# Dual Nickel/Photoredox‐Catalyzed Regio‐ and Enantioselective Alkenylation of Epoxides

**DOI:** 10.1002/advs.77326

**Published:** 2026-08-29

**Authors:** Hongyan Lan, Shengzhou Jin, Zhao‐Dong Xu, Guigen Li, Dingyi Wang

**Affiliations:** ^1^ College of Sciences Northeastern University Shenyang China; ^2^ Hubei Key Laboratory of Pollutant Analysis & Reuse Technology College of Chemistry and Chemical Engineering Hubei Normal University Huangshi China; ^3^ School of Civil Engineering Southeast University Nanjing China; ^4^ Department of Chemistry and Biochemistry Texas Tech University Lubbock Texas USA

**Keywords:** alkenylation, cross‐electrophile coupling, dual metal catalysis, epoxides, nickel catalysis

## Abstract

*β*‐Chiral phenethyl alcohols represent a privileged scaffold in bioactive molecules, chemical probes, and functional materials, serving as versatile synthons in organic synthesis. Herein, we report the first Ni/photoredox‐catalyzed stereoconvergent C(sp^3^)−C(sp^2^) cross‐electrophile coupling of racemic epoxides with alkenyl bromides, delivering *β*‐alkenyl phenylethanol with high enantioselectivity (up to 95% ee), excellent *E*‐stereoselectivity, and complete regiocontrol. The enantioenriched products serve as versatile intermediates for diverse chiral synthons, enabling further applications. Mechanistic studies suggest a stereoconvergent pathway involving regioselective halide ring‐opening of epoxides, followed by enantioselective coupling of the generated *β*‐hydroxy benzyl halides with alkenyl bromides.

## Graphical abstract

Ni/photoredox‐catalyzed stereoconvergent cross‐electrophile coupling of racemic epoxides with alkenyl bromides is described, affording *β*‐alkenyl products with up to 95% *ee*, excellent E‐selectivity, and complete regiocontrol. These versatile intermediates enable diverse chiral synthons; mechanistic studies suggest a halide ring‐opening followed by enantioselective coupling.

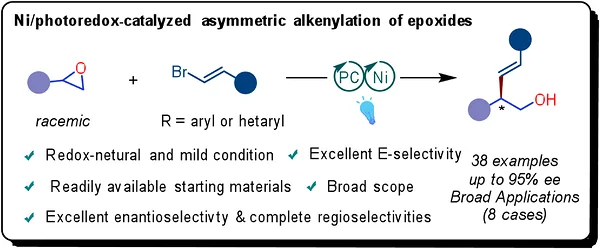

## 1 Introduction

Epoxides are among the most versatile building blocks in synthetic chemistry due to their high ring‐strain and energy [1, 2, 3, 4, 5, 6, 7, 8, 9, 10, 11, 12, 13]. In asymmetric synthesis, catalytic asymmetric ring‐opening of epoxides with nucleophiles offers a powerful strategy to access enantiomerically enriched alcohols (Scheme 1a) [14, 15, 16, 17]. These chiral alcohols, upon further polymerization or cross‑linking, hold promise for the fabrication of novel viscoelastic materials, thereby offering a pathway to improve their vibration mitigation capacity. Although well‐established, this approach relies on stereospecific ring‐opening via kinetic resolution of racemic epoxides or desymmetrization of meso‐epoxides using soft, heteroatom‐centered nucleophiles (e.g., cyanide, water, and azide) [18, 19, 20, 21, 22]. These methods suffer from limited substrate availability, harsh reaction conditions, and poor functional group tolerance. To address these limitations, developing chiral catalyst‐controlled stereoconvergent C─C bond‐forming reactions of racemic epoxides to access chiral *β*‐phenylethanols would be highly valuable (Scheme 1b) [23, 24, 25, 26, 27, 28, 29, 30, 31, 32, 33, 34, 35, 36, 37, 38]. In 2017, Yamamoto and co‐workers developed a nickel‐catalyzed enantio‐ and diastereoselective cross‐coupling of 3,4‐epoxyalcohols, employing a hydroxy directing group to synthesize 4,4‐diarylalkanes bearing additional 1,3‐diol functionality [24]. Subsequently, the Doyle group achieved asymmetric cross‐electrophile coupling of styrene oxides with aryl iodides via cooperative nickel photocatalysis, yielding enantioenriched 2,2‐diarylalcohols [25]. In 2024, the Guo group introduced a mild method for the regio‐ and enantioselective alkynylation of epoxides with terminal alkynes, enabling the synthesis of chiral β‐homopropargyl alcohols through combined copper and photoredox catalysis [26].

**SCHEME 1:**
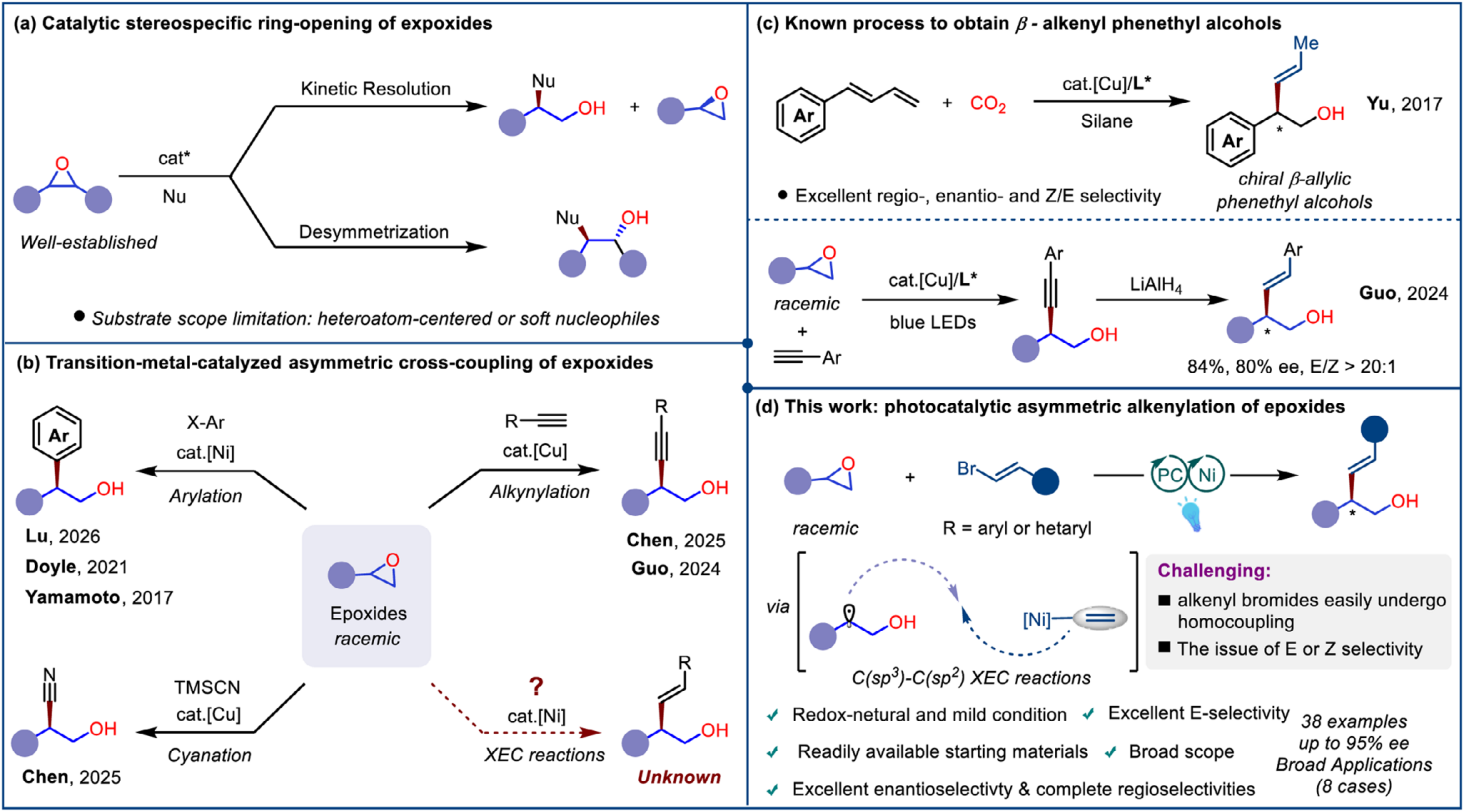
Strategies for enantioselective functionalization of epoxides.

In 2025, Chen reported a photoinduced Cu‐catalyzed cyanation/alkynylation of epoxides, affording cross‐coupled products with excellent enantioselectivity [27]. The carbon‐carbon double bond is a pivotal functional group owing to its versatility in chemical transformations, rendering it highly valuable in pharmaceuticals, agrochemicals, and materials science [28, 29, 30, 31, 32]. Consequently, incorporating alkenyl functionalities into the phenethyl alcohol scaffold to construct valuable chiral alkenyl alcohols is of substantial synthetic importance. Such compounds would serve as advanced intermediates, bearing a versatile synthetic handle for enabling rapid diversification into a wide array of valuable derivatives. Despite this potential, a general and efficient asymmetric strategy to access these structures constitutes a notable synthetic void. To the best of our knowledge, only Yu and coworkers have reported a singular example of copper‐catalyzed reductive hydroxymethylation of 1,3‐dienes, although this method is restricted to the synthesis of chiral *β*‐allylic phenethyl alcohols (Scheme 1c, top) [33, 34]. Alternatively, Guo et al. reported the hydrogenation of chiral *β*‐homopropargyl alcohols with lithium aluminium hydride, affording the corresponding alkenylethyl alcohols with moderate enantioselectivity (Scheme 1c, bottom) [26]. In this context, we report the first enantioconvergent C(sp^3^)−C(sp^2^) cross‐electrophile coupling (XEC) between racemic epoxides and alkenyl bromides. This method enables the efficient synthesis of structurally diverse chiral *β*‐alkenyl phenylethanol through cooperative nickel/photoredox dual catalysis under mild conditions, achieving good‐to‐excellent enantioselectivity with complete regiocontrol and high *E*‐selectivity (Scheme 1d). This mild protocol exhibits broad applicability, enabling the late‐stage functionalization of complex pharmacophores. The resultant *β*‐alkenyl phenolethanol serves as a pivotal intermediate, enabling the rapid assembly of a wide range of structurally complex, chiral frameworks.

Our investigation into the Ni/photoredox‐catalyzed enantioselective alkenylation of epoxides using racemic styrene oxides and alkenyl bromides as model substrates to synthesize chiral *β*‐alkenyl alcohols by using a variety of chiral ligands with Mg salts and bases (Table 1). Optimization revealed that the reaction employing 2.0 mol% 4CzIPN, 10 mol% NiBr_2_·DME, 12 mol% chiral Biimidazoline (BiIM) ligands **L1**, 25 mol% MgCl_2_, and 5.0 equiv of Et_3_N in THF under 425 nm blue LEDs irradiation, the desired alkenylation product **3aa** could be obtained in 72% isolated yield with 93% ee. The reaction exhibited excellent stereoselectivity, affording exclusively E‐product **1** regardless of whether Z‐ or E/Z‐mixed *β*‐bromostyrenes were employed as starting materials. Smaller aryl substituents on BiIM ligands (**L2** and **L3**) lowered enantioselectivity. The use of other chiral bidentate ligands (e.g., pyridine‐oxazoline, bisoxazoline) (**L4**‐**L7**) led to significantly lower yield and enantioselectivity. Different nickel catalysts, such as NiBr_2_ and NiCl_2_·DME afforded the corresponding product with lower yields but similar ee (entries 2–3). The yield was significantly decreased by using MgI_2_ instead of MgCl_2_ as an additive (entry 4). This may be attributed to the lower stability of the iodo‑substituted analogue formed during epoxide ring‑opening under the reaction conditions, compared to the corresponding chloride intermediate. Solvent screening showed that 1,4‐dioxane or DME was less effective than THF (entries 5–6). Other common organic bases, including DBU and DABCO, failed to afford the target product when used as additives (entries 7–8). The use of DIPEA as a reductant afforded only a small amount of product with low enantioselectivity. Additionally, light wavelength proved crucial; switching to 456 or 400 nm blue LEDs gave inferior results (entries 10–11). Control experiments confirmed the necessity of the photocatalyst, Ni salt, ligand, MgCl_2_, NEt_3_, and light are required for this transformation (entry 12).

**TABLE 1:** Optimization of Reaction Conditions ^a^.

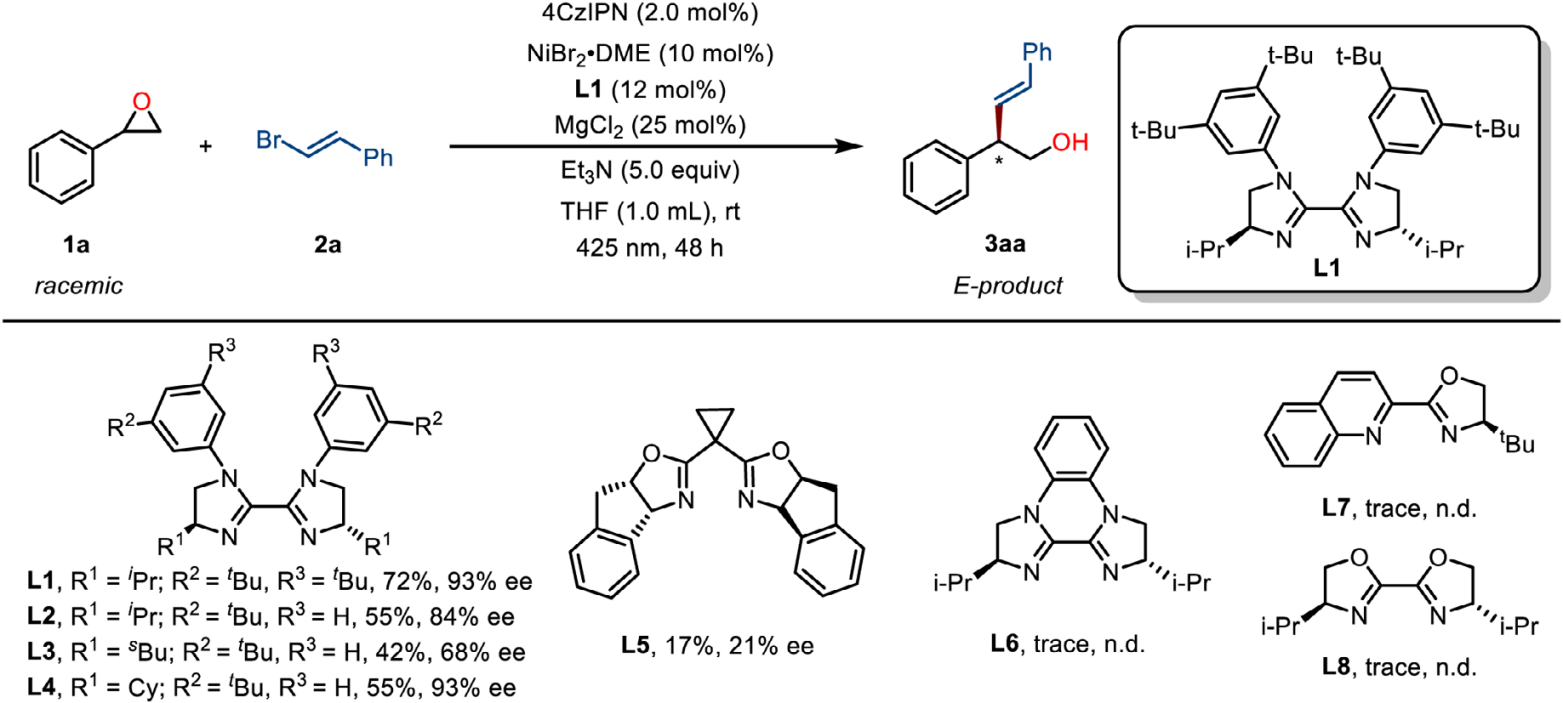			
Entry	Derivation of the optimal condition	Yield^b^ (%)	ee^c^ (%)
1	None	72	93
2	NiBr_2_ instead of NiBr_2_·DME	58	91
3	NiCl_2_·DME instead of NiBr_2_·DME	67	93
4	MgI_2_ instead of MgCl_2_	30	91
5	1,4‐dioxane instead of THF	62	82
6	DME instead of THF	64	85
7	DBU instead of Et_3_N	N.R.	—
8	DABCO instead of Et_3_N	N.R.	—
9	DIPEA instead of Et_3_N	31	70
10	456 nm instead of 425 nm	41	93
11	400 nm instead of 425 nm	37	94
12	w/o 4CzIPN, Ni, **L1**, light, Et_3_N or MgCl_2_	< 5	—

^a^ Reaction conditions: epoxides **1a** (0.1 mmol), alkenyl bromides **2a** (3.0 equiv), photocatalyst (2.0 mol%), NiBr_2_·DME (10 mol %), **L1** (12 mol %), MgCl_2_ (25 mol%), Et_3_N (5.0 equiv), THF (1.0 mL), 425 nm, rt, 48 h, N_2_.

^b^ Isolated yields.

^c^ Determined by HPLC on a chiral stationary phase. DBU = 1,8‐Diazabicyclo [5.4.0]‐7‐Undecene; DABCO = Triethylenediamine; DIPEA = *N*‐Ethyldiisopropylamine.

## 2 Results and Discussion

Having established optimal conditions, we explored the substrate scope of the photoredox/Ni‐catalyzed enantioselective epoxide alkenylation (Scheme 2). Styrene oxides with *para*‐ or *meta*‐substituents—including electron‐donating (−Me, −*t*Bu), electron‐withdrawing (CF_3_, COOMe), and halogen (F, Cl, Br) groups—coupled efficiently with alkenyl bromides to afford chiral *β*‐alkenyl phenylethanol **3aa**‐**3ja** in moderate‐to‐good yields (47%–74%) with excellent enantioselectivity. *Ortho*‐substituted substrates provided a lower yield (37%) and enantioselectivity (87% ee), revealing steric hindrance on both reactivity and stereocontrol. Additionally, biphenyl‐type epoxides are perfectly compatible, and the ring‐open product (**3la** and **3ma**) was generated in good yield with a high ee value. Furthermore, the reaction of furan‐ ‐substituted epoxides resulted in the formation of alkenylation products (**3na**) with good yields and enantioselectivities. However, epoxides bearing substituents at their terminal positions did not yield the corresponding alkenylation products (**3oa**) under the standard conditions. This is likely due to the significant steric encumbrance of such substituents, which impedes productive substrate engagement.

**SCHEME 2:**
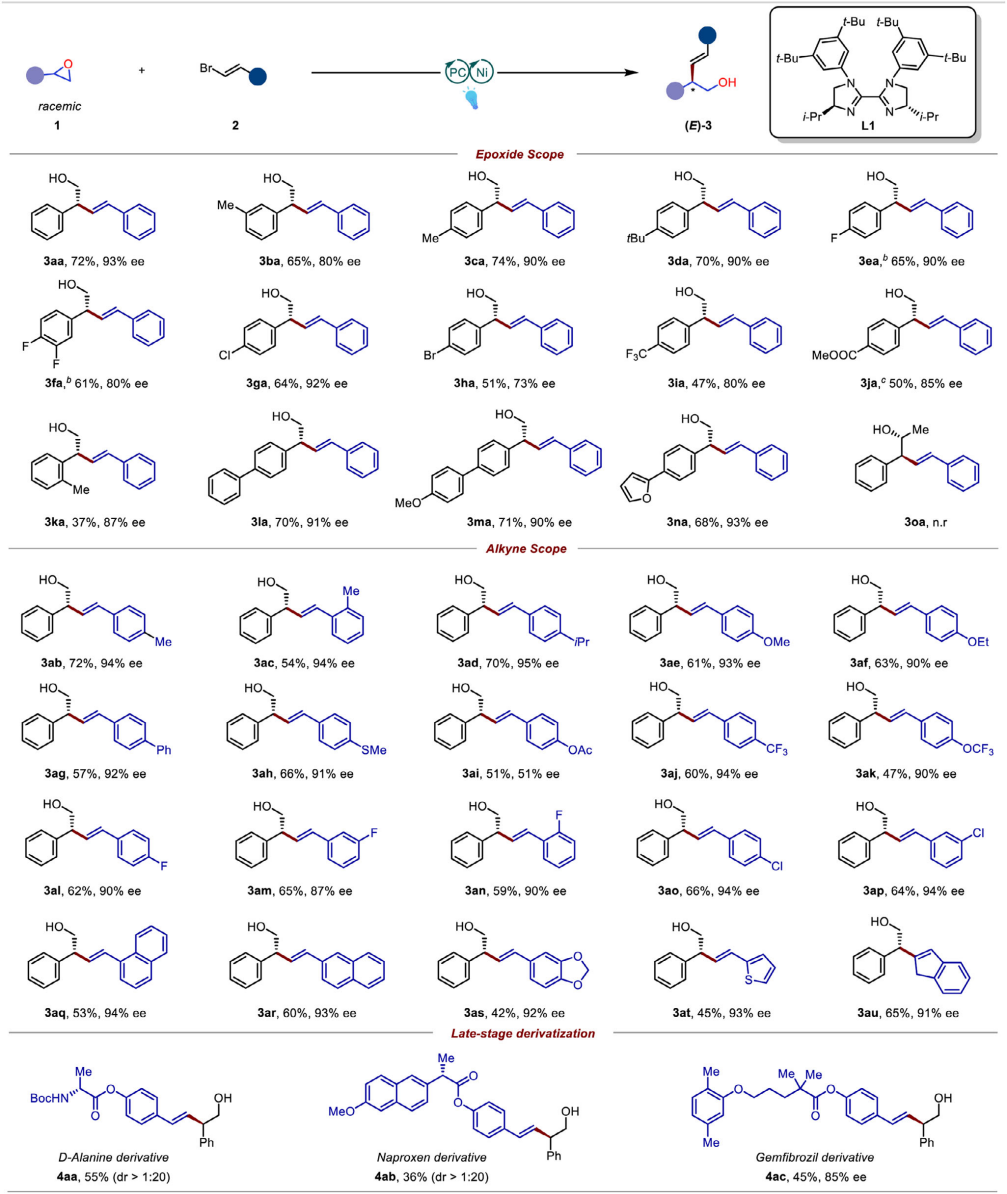
Substrate Scope of Ni/Photoredox‐Catalyzed Alkenylation of Epoxides. *^a^* Reaction conditions: epoxides **1a** (0.1 mmol), alkenyl bromides **2a** (E/Z > 20:1, 3.0 equiv), photocatalyst (2.0 mol%), NiBr_2_·DME (10 mol %), **L1** (0.012 mmol, 12 mol %), MgCl_2_ (25 mol%), Et_3_N (5.0 equiv), THF (1.0 mL), 425 nm, rt, 48 h, and N_2_. Isolated yields. Determined by HPLC on a chiral stationary phase. *^b^* **L1** (0.018 mmol, 18 mol %), and *^c^* **L4** (0.012 mmol, 12 mol %).

Next, we turned our attention to exploring the versatility of alkenyl bromides. Styrenyl bromides bearing diverse substituents—including alkyl (**3ab**‐**3ad**), alkoxy (**3ae**‐**3af**), phenyl (**3ag**), methylthio (**3ah**), acetyloxy (**3ai**), trifluoromethyl (**3aj**), trifluoromethoxy (**3ak**), and halogen (**3al**‐**3ap**)‐ all provided the corresponding products in good yields. Compared with epoxides, ortho‐substituted allyl bromides similarly afforded lower yields but maintained high diastereoselectivity (e.g., **3ac**, **3an**). Furthermore, vinyl bromides bearing an acetoxy group exhibited significantly diminished enantioselectivity. This decrease is likely attributable to the coordinating nature of the acetoxy group, which may compete with the chiral ligand for coordination sites on the nickel center. To our delight, alkenyl bromides bearing naphthalene, 1,3‐benzodioxole, and thiophene rings were tolerated as well, and the desired alkenylation products were obtained with good yields and excellent enantioselectivities (**3aq**‐**3at**). Remarkably, 2‐bromo‐1H‐indene—a cyclic disubstituted alkenyl bromide that is typically challenging in asymmetric alkenylation reactions—served as a competent substrate under the standard conditions, affording product **3au** in good yield with excellent enantioselectivity. The synthetic utility of this photo‐nickel dual catalytic system was further demonstrated through the late‐stage functionalization of diverse natural products and pharmaceutical derivatives. Specifically, allyl bromides derived from D‐alanine, naproxen, and gemfibrozil were incorporated, affording the corresponding chiral *β*‐alkenyl ethyl alcohols **4aa**–**4ac**.

To demonstrate the synthetic utility of this enantioselective protocol, we applied it to prepare key synthetic intermediates, as outlined in Scheme 3. First, the value‐added epoxide **5aa** was synthesized via a straightforward*m*‐CPBA‐mediated epoxidation of **3aa**, obtaining the product in 76% yield with a 1:1 dr. Moreover, a stereoselective Ti(Oi‐Pr)_4_‐catalyzed heterocyclization with benzhydrol in HFIP afforded valuable saturated chiral *O*‐heterocycles **5ab**. Additionally, the alkenylation products underwent diastereoselective iodocyclization in the presence of I_2_ and NaHCO_3_ to deliver enantioenriched iodosubstituted pyrrolidine (**5ac**), containing three contiguous chiral centers. Importantly, subsequent tosylation of the alcohol provided the chiral sulfonate **5ad**, which served as a versatile intermediate, undergoing nucleophilic substitution with various nucleophiles to afford chiral products in good yields with retained enantiopurity. For instance, the treatment of **5ad** with LiAlH_4_ afforded the corresponding dehydroxylated product **5ae** in good yield (91%) with good enantiomeric ratio (95:5 er). The substitution with CBr_4_ or NaI afforded the chiral halides **5af** and **5ag** in 95% yield with 93% ee and 88% yield with 91% ee, respectively. The installation of a thioether group was achieved using PhSNa, providing **5ah** in 90% yield with 91% ee. The above synthetic applications demonstrate the significant potential of the synthesized *β*‐chiral phenethyl alcohols for constructing complex molecular architectures.

**SCHEME 3:**
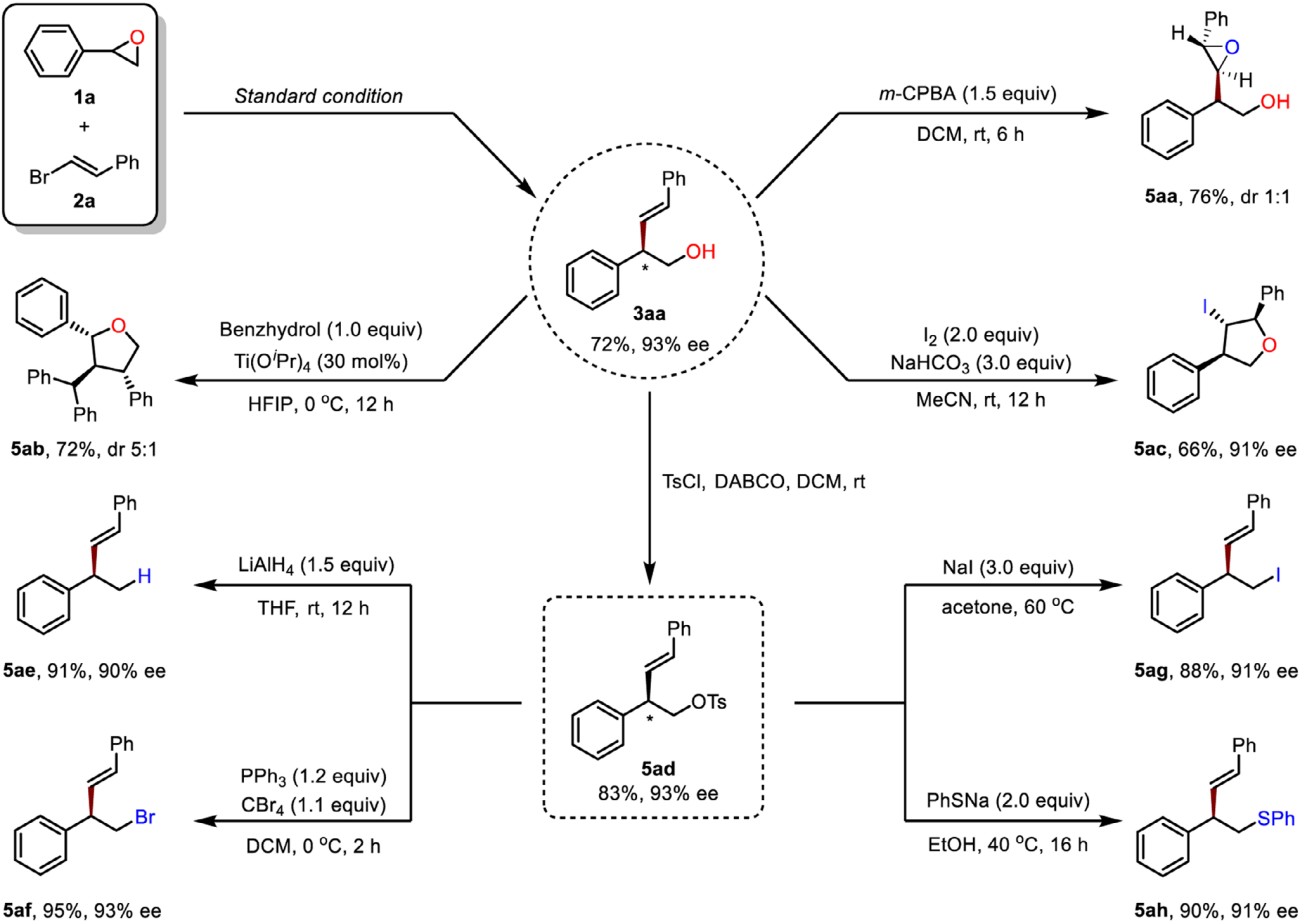
Downstream Transformations.

A combination of control experiments and cyclic voltammetry (CV) studies was performed to elucidate the mechanism of the asymmetric alkenylation. The transformation proceeds stereoconvergently, as product **3aa** was obtained with similar enantioselectivity from both racemic and enantioenriched styrene oxide substrates (Scheme 4a). Monitoring the reaction at different conversions revealed a constant enantiomeric excess for product **3aa**, whereas the recovered substrate **1a** was consistently racemic (Scheme 4b). This result confirms an enantioconvergent pathway that bypasses kinetic resolution of the aziridine precursors. We then used high‐resolution mass spectrometry (HR‐MS) to probe the ring‐opening step and identify halohydrin intermediates (Scheme 4c). Indeed, benzyl chloride **6aa** was detected when MgCl_2_ (1.0 equiv) was present. In contrast, **6aa** was not detected when NiBr_2_·DME was removed. Furthermore, *β*‐bromophenethyl alcohol **7aa** was not identified by HR‐MS in the absence of MgCl_2_ under otherwise standard conditions. These HR‐MS results, along with prior literature, indicate that MgCl_2_ acts as a source of Cl(‐) nucleophile for ring‐opening, while the nickel catalyst serves as a Lewis acid. We next investigated whether the halohydrins **6aa** were competent reaction intermediates. Subjecting them to the standard reaction conditions instead of styrene oxide **1a** afforded product **3aa** in 67% with a 92% ee value (Scheme 4d). The reaction proceeded efficiently with Ni(cod)_2_ as the catalyst (66% yield, 90% ee) (Scheme 4e). In addition, when the nickel complex Ni‐1 was used as a substrate in place of the alkenyl bromide, the desired product was obtained. Moreover, employing Ni‐1 directly as the catalyst also afforded the target product in 54% yield (Please see the Supporting Information). These results support a pathway in which Ni(0) first undergoes oxidative addition with the alkenyl bromide to generate a Ni(II) species, which then reacts with the benzyl radical intermediate. This result supports a pathway involving the direct oxidative addition of alkenyl bromides to a Ni(0) species. Radical trapping experiments with TEMPO afforded the TEMPO‐benzyl adduct **8aa**, which was detected by HR‐MS (Scheme 4f). In contrast, the target adducts were not observed in the absence of both MgCl_2_ and NiBr_2_·DME, which excludes a pathway involving direct single‐electron reduction of the epoxide. Cyclic voltammetry (CV) of the NiBr_2_(dme)/**L1** complex revealed two irreversible reduction waves, with onset potentials at *E*_onset_(Ni^I^/Ni^0^) = −1.40 V vs. Ag/AgCl in THF and *E*_onset_(Ni^II^/Ni^I^) = −0.81 vs. Ag/AgCl in THF (Scheme 4 g). These potentials are consistent with the reduction of the Ni(II) precatalyst in situ to a Ni(I) species by the photocatalyst. The observation of a linear correlation in the nonlinear effect study points to a monomeric nickel complex, coordinated by a bidentate ligand, as the enantioselectivity‐governing species (Scheme 4 h).

**SCHEME 4:**
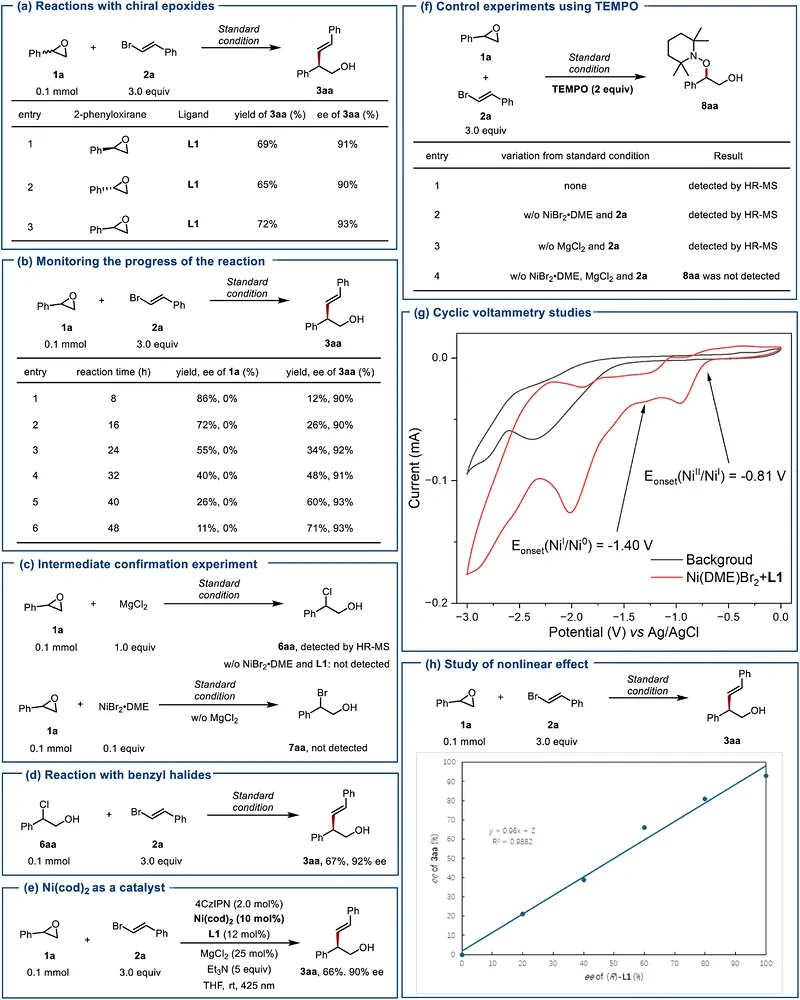
Mechanistic investigations.

Integrating our mechanistic findings with prior literature [14, 15, 16], we propose the catalytic cycle depicted in Scheme 5 for the enantioselective synthesis of chiral *β*‐alkenyl alcohols. The catalytic cycle commences with the oxidative addition of alkenyl bromide **2** to the Ni(0) species **A**, generating Ni(II) intermediate **B**. Concurrently, the regioselective ring‐opening of epoxides **1** affords racemic benzyl chloride intermediate **C**. This intermediate then undergoes halogen atom abstraction (HAA) or single‐electron transfer (SET) to produce the key benzylic radical **D**. Radical **D** is then captured by Ni(II) complex **B**, yielding Ni(III) species **E**. Subsequent reductive elimination from **E** affords the desired *β*‐alkenyl phenylethanol **3** and regenerates the Ni(I) species **F**. Finally, the active Ni(0) catalyst **A** is regenerated via reduction of Ni(I) species **F** by the photocatalytically generated 4CzIPN radical anion. This reduced photocatalyst is formed by reductive quenching of the excited‐state photocatalyst by triethylamine.

**SCHEME 5:**
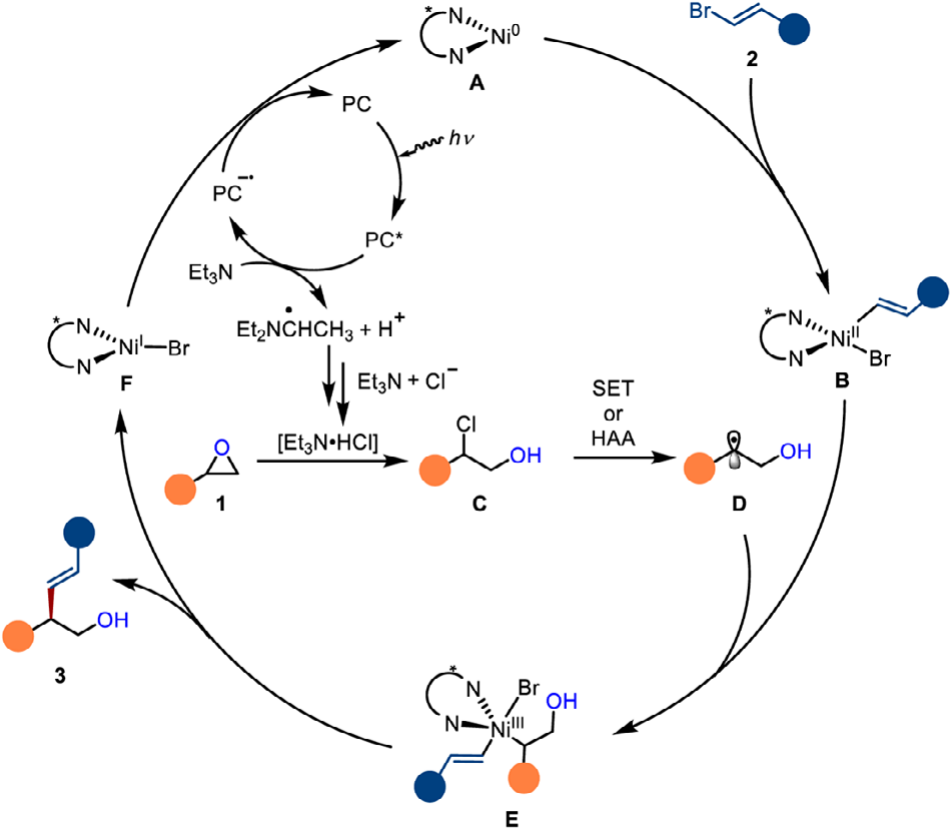
Proposed Reaction Mechanism.

## 3 Conclusion

In summary, we have developed the first nickel/photoredox‐catalyzed enantioselective cross‐electrophile coupling of racemic epoxides with alkenyl bromides. This method provides efficient and modular access to enantioenriched *β*‐alkenyl phenylethanols with high enantioselectivity (up to 95% ee) and complete regiocontrol and excellent E/Z selectivity. The reaction features a broad substrate scope, accommodating a wide range of both epoxides and alkenyl bromides, including those bearing pharmacologically relevant motifs. The resultant (*E*)‐*β*‐alkenyl phenolethanol serves as a pivotal intermediate, enabling the rapid assembly of a wide range of structurally complex, chiral frameworks. Mechanistic investigations elucidated a stereoconvergent radical pathway, which was supported by control experiments and electrochemical studies.

## Author Contributions

**Hongyan Lan**: investigation, validation, Writing – original draft. **Zhao‐Dong Xu**: resources, supervision, writing – review and editing. **Shengzhou Jin**: investigation, validation. **Dingyi Wang**: investigation, conceptualization, methodology, funding acquisition, resources, writing – review and editing, supervision, validation. **Guigen Li**: supervision, writing – review and editing, resources, investigation.

## Conflicts of Interest

The authors declare no conflicts of interest.

## Supporting information




**Supporting File**: advs77326‐sup‐0001‐SuppMat.pdf.

## Data Availability

The data that support the findings of this study are available in the Supporting Information of this article.
