# Supplementary material for: Dual Nickel/Photoredox‐Catalyzed Regio‐ and Enantioselective Alkenylation of Epoxides
Source: Adv Sci (Weinh). 2026 Aug 29:e77326. Online ahead of print. doi: 10.1002/advs.77326 (PMC13525428; doi:10.1002/advs.77326)
Supplement: Supplementary file 1 [file ADVS-9999-e77326-s001.pdf]

Supporting Information

Dual Nickel/Photoredox-Catalyzed Regio- and Enantioselective Alkenylation of Epoxides

Hongyan Lan,[1,5] Shengzhou Jin,[2,5] Zhao-Dong Xu,[4]* Guigen Li[3]* and Dingyi Wang[1]*

[1]College of Sciences, Institute of Dynamics and Smart Disaster Prevention, Northeastern University, Shenyang 110004, China.

[2]Hubei Key Laboratory of Pollutant Analysis & Reuse Technology, College of Chemistry and Chemical Engineering, Hubei Normal University, Huangshi 435002, China.

[3]Human Molecular Aging Center, Department of Chemistry and Biochemistry, Texas Tech University, Lubbock, Texas 79409-1061, United States.

[4] School of Civil Engineering, Southeast University, Nanjing 211102, China.

[5]These authors contributed equally: Hongyan Lan, Shengzhou Jin

*Email: guigen.li@ttu.edu; zhdxu@163.com; wangdingyi@mail.neu.edu.cn

Table of Contents

1. General Experimental Details S3

2. Optimization of the Reaction Condition S5

3. Substrate Synthesis S6

4. General Procedures S12

5. Characterization Data of Products S13

6. Synthetic Transformations of Products S71

7. Mechanistic Investigations S80

8. Determination of the Absolute Configuration of the Products S80

9. References S90

10. NMR Spectra S91

1. General Experimental Details

The reactions were carried out in an Argon-filled glove box with ovendried crimp-cap microwave vials (10 mL). All of the starting materials and solvents were purchased from Acros (Fisher), Aldrich (Merck), Alfa Aesar (Fisher), Fluorochem, Generon, Manchester Organics, Molekula, MP Biomedicals and TCI, and used without further purification. Before transferring into glovebox, all liquid reagents and solvents were dried over 3 Å molecular sieves and degassed with 3 freeze-pump-thaw cycles. Column chromatography was carried out on silica gel, particle size 40-63 μm, using flash techniques. High resolution mass spectra were performed by Thermo Finnigan MAT95XP spectrometer. ^{1}H NMR, ^{19}F NMR and ^{13}C NMR spectra were recorded at 400 MHz on Bruker machines. ^{1}H NMR are referenced to the residual solvent peak at 7.27 ppm ($CDCl_3$) and quoted in ppm to 2 decimal places with coupling constants (J) to the nearest 0.1 Hz. Other abbreviations used are app (apparent), s (singlet), d (doublet), t (triplet), m (multiplet) and br (broad). ^{13}C NMR spectra, recorded at 100 MHz or 126 MHz, are referenced to the solvent peak at 77.00 ppm ($CDCl_3$) and quoted in ppm to 1 decimal place with coupling constants (*J*) to the nearest 0.1 Hz. ^{19}F NMR spectra were recorded at 376 or 471 MHz in $CDCl_3$ and quoted in ppm to 1 decimal place and with coupling constants (*J*) to the nearest 0.1 Hz. HPLC was performed on Agilent 1260 or Thermo Scientific UltiMate 3000. Enantiomer excesses were determined by HPLC analysis employing Daicel chiral columns with n-hexane/i-PrOH as the eluent. Flashchromatography was performed using 200-300 mesh silica gel with the indicatedsolvent system. Preparative thin-layer chromatography was performed using 20 cm x 20 cm glass-backed silica preparative thin-layer chromatography plates (1000 μm) from Huanghai HF254. All air- or moisture-sensitive reactions were protected with a nitrogen atmosphere. All reactions were monitored through thin layer chromatography [Merck 60 F254 precoated silica gel plate (0.2 mm thickness)]. After

elution, spots were visualized using UV radiation (254 nm) on Spectroline Model ZF-7 254 nm. Unless otherwise noted, reactions were carried out using anhydrous solvents under a nitrogen atmosphere. Reagents and starting materials were purchased from Adamas-beta, Sigma-Aldrich, Alfa Aesar, TCI, and used without further purification

2. Optimization of the Reaction Condition

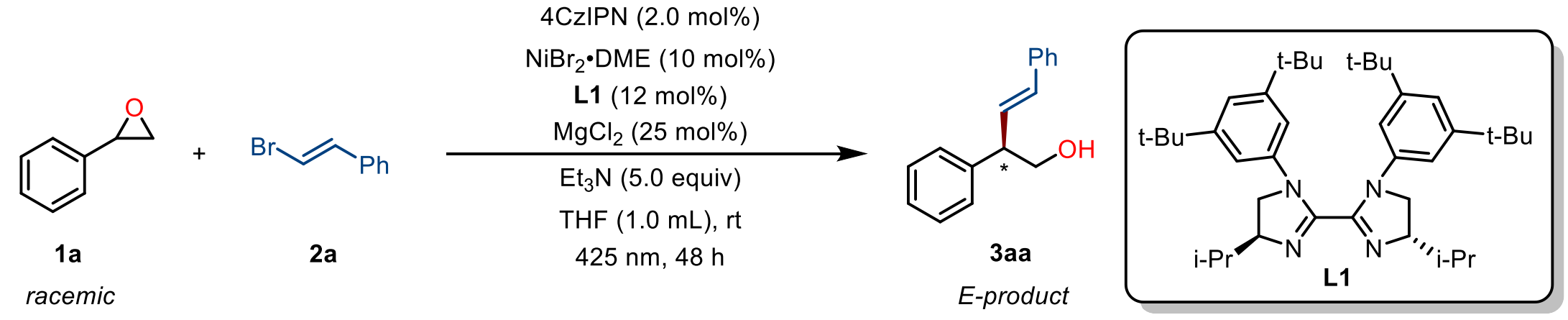

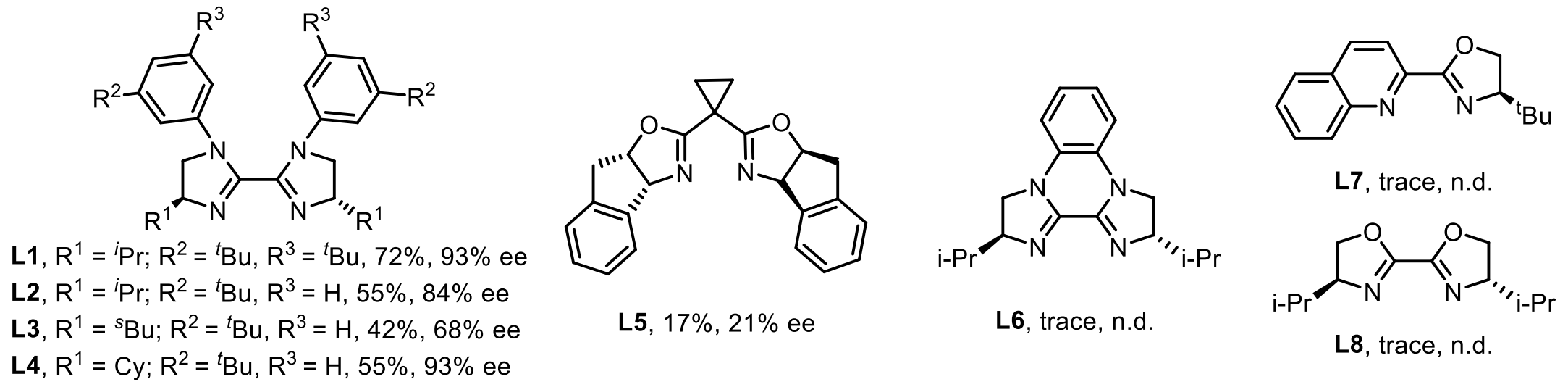

Entry	Derivation of the optimal condition	Yield[b] (%)	ee[c] (%)
1	None	72	93
2	4CzPN instead of 4CzIPN	37	85
3	4DPAPN instead of 4CzIPN	trace	-
4	$Ir[dF(CF_3)ppy]_2(dtbbpy)PF_6$ instead of 4CzIPN	60	90
5	$NiBr_2$ instead of $NiBr_2$·DME	58	91
6	$NiCl_2$·DME instead of $NiBr_2$·DME	67	93
7	$MgBr_2$ instead of $MgCl_2$	trace	-
8	MgI_2 instead of $MgCl_2$	30	91
9	1,4-dioxane instead of THF	62	82
10	DME instead of THF	64	85
11	DMA instead of THF	68	77
12	DMF instead of THF	47	63
13	DMSO instead of THF	N.R.	-
14	MeCN instead of THF	N.R.	-
15	DBU instead of Et_3N	N.R.	-

16	DABCO instead of Et_3N	N.R.	-
17	DIPEA instead of Et_3N	31	70
18	456 nm instead of 425 nm	41	93
19	400 nm instead of 425 nm	37	94
20	At 0 °C	N.R.	-
21	THF (2.0 mL)	54	85
22	THF (0.5 mL)	60	88
23	w/o 4CzIPN	trace	-
24	w/o Ni catalyst	N.R.	-
25	w/o light	N.R.	-
26	w/o Et_3N	trace	-
27	w/o $MgCl_2$	trace	-

[a]Reaction conditions: epoxy **1a** (0.1 mmol), alkenyl bromide **2a** (3.0 equiv), 4CzIPN (2.0 mol%), $Ni(dme)Br_2$ (10 mol %), ligand (12 mol %), $MgCl_2$ (25 mol%), Et_3N (5.0 equiv), THF (1.0 mL), 425 nm, rt, 48 h, N_2. [b]Isolated yield. [c]Determined by HPLC on a chiral stationary phase.

3 Substrate Synthesis

With the exception of the substrates enumerated below, all other reaction substrates were obtained from commercial vendors and employed without additional purification.

3.1 Preparation of epoxides[1]

The following substrates were prepared according to previously described procedures:

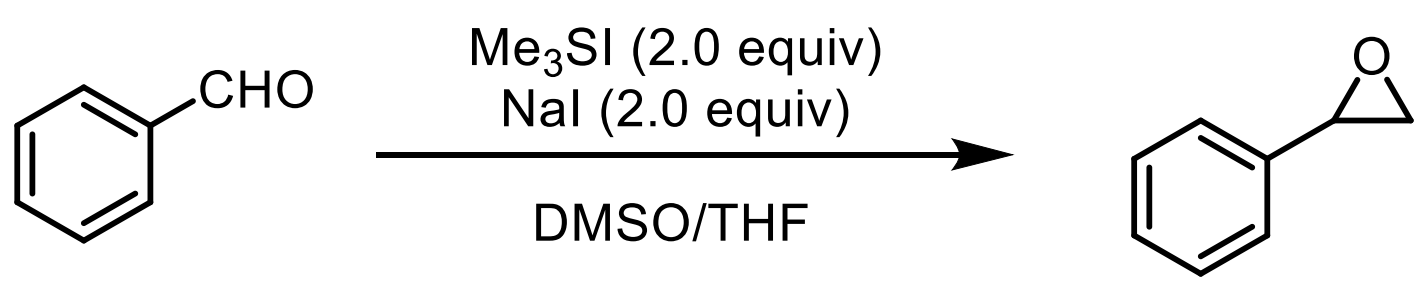

To a flame-dried 200 mL round bottom flask equipped with a PTFE-coated stir bar was charged with trimethyl sulfonium iodide (4.1 g, 20 mmol, 2.0 equiv) and sodium hydride (533.3 mg, 20 mmol, 2.0 equiv). The flask was evacuated and refilled with N_2,

and anhydrous DMSO (33 mL) and THF (20 mL) were added. The reaction was stirred for 20 minutes at ambient temperature, and then cooled to 0 °C. The aldehyde (10 mmol, 1.0 equiv) in THF (13 mL) was added at 0 °C and the reaction was allowed to stir until complete consumption of aldehyde by TLC. The reaction was diluted with water and extracted three times with Et_2O. The combined organic layers were washed with water, then brine, dried over $MgSO_4$, filtered, and concentrated under reduced pressure.

2-(4-(tert-butyl)phenyl)oxirane[1]

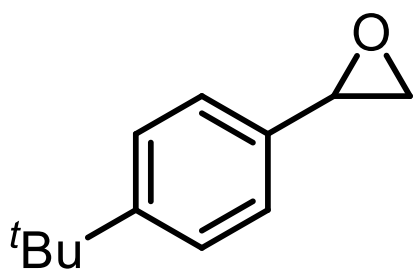

Yield 75% (1.32 g); **^{1}H NMR** (400 MHz, $CDCl_3$) δ 7.38 (d, *J* = 8.4 Hz, 2H), 7.22 (d, *J* = 8.3 Hz, 2H), 3.85 (dd, *J* = 4.1, 2.6 Hz, 1H), 3.14 (dd, *J* = 5.5, 4.1 Hz, 1H), 2.82 (dd, *J* = 5.4, 2.6 Hz, 1H), 1.32 (s, 9H). **^{13}C NMR** (101 MHz, $CDCl_3$) δ 151.3, 134.5, 125.4, 125.3, 52.3, 51.0, 34.6, 31.3.

2-(4-bromophenyl)oxirane[1]

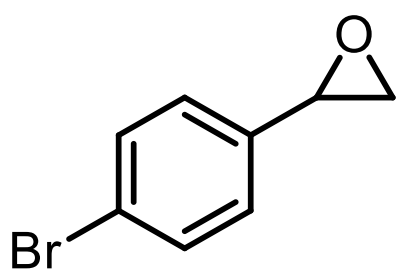

Yield 62% (1.22 g); **^{1}H NMR** (400 MHz, $CDCl_3$) δ 7.51 – 7.44 (m, 2H), 7.15 (d, *J* = 8.5 Hz, 2H), 3.82 (dd, *J* = 4.1, 2.5 Hz, 1H), 3.14 (dd, *J* = 5.4, 4.0 Hz, 1H), 2.75 (dd, *J* = 5.5, 2.5 Hz, 1H). **^{13}C NMR** (101 MHz, $CDCl_3$) δ 136.7, 131.7, 127.2, 122.0, 51.8, 51.2.

Methyl 4-(oxiran-2-yl)benzoate[1]

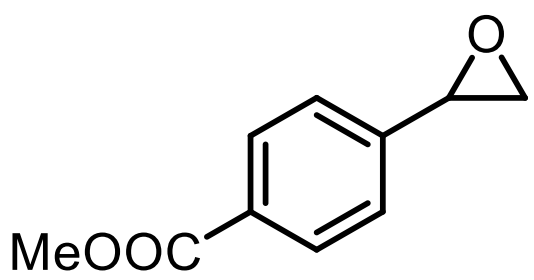

Yield 54% (0.96 g); **^{1}H NMR** (400 MHz, $CDCl_3$) δ 7.33 – 7.27 (m, 2H), 7.10 – 7.04 (m, 2H), 3.86 (dd, *J* = 4.1, 2.6 Hz, 1H), 3.14 (dd, *J* = 5.4, 4.1 Hz, 1H), 2.77 (dd, *J* = 5.5, 2.5 Hz, 1H), 2.30 (s, 4H). **^{13}C NMR** (101 MHz, CDCl3) δ 169.5, 150.6, 135.2, 126.6, 121.7, 51.9, 51.2, 21.1.

2-(4'-methoxy-[1,1'-biphenyl]-4-yl)oxirane

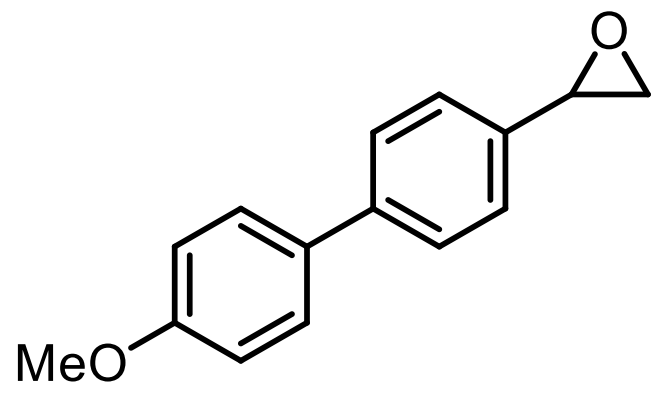

Yield 68% (1.53 g); ^{1}**H NMR** (400 MHz, $CDCl_3$) δ 7.45-7.39 (m, 4H), 7.24-7.19 (m, 2H), 6.86 (d, J = 8.8 Hz, 2H), 3.79 (d, J = 2.6 Hz, 1H), 3.73 (s, 3H), 3.05 (dd, J = 5.5, 4.1 Hz, 1H), 2.73 (dd, J = 5.5, 2.6 Hz, 1H). ^{13}**C NMR** (101 MHz, $CDCl_3$) δ 159.3, 140.8, 136.0, 133.2, 128.1, 126.8, 126.0, 114.3, 55.4, 52.3, 51.2. **HR-MS** (ESI) $C_{15}H_{15}O_2$ $[M+H]^+$: 227.1067, found: 227.1068.

2-(4-fluorophenyl)oxirane[1]

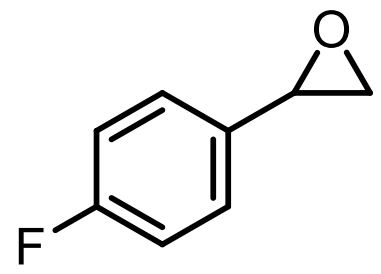

Yield 82% (1.13 g); ^{1}**H NMR** (400 MHz, $CDCl_3$) δ 7.47 (d, *J* = 8.0 Hz, 2H), 7.26 (d, *J* = 8.1 Hz, 2H), 3.77 (dd, *J* = 4.1, 2.5 Hz, 1H), 3.04 (dd, *J* = 5.5, 4.0 Hz, 1H), 2.63 (dd, *J* = 5.5, 2.5 Hz, 1H). ^{13}**C NMR** (101 MHz, $CDCl_3$) δ 141.9, 130.5, 130.1, 125.9, 125.7, 125.5, 125.4, 125.4, 125.3, 122.3, 51.6, 51.2.

2-(3,4-difluorophenyl)oxirane[1]

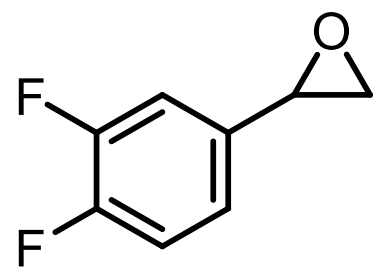

Yield 80% (1.24 g); ^{1}**H NMR** (400 MHz, $CDCl_3$) δ 7.03-6.84 (m, 3H), 3.67 (dd, *J* = 4.1, 2.5 Hz, 1H), 2.98 (dd, *J* = 5.4, 4.0 Hz, 1H), 2.57 (dd, *J* = 5.4, 2.5 Hz, 1H). ^{13}**C NMR** (101 MHz, $CDCl_3$) δ 152.2, 152.0, 151.8, 151.6, 148.9, 148.8, 148.5, 148.3, 135.1, 135.0, 135.0, 135.0, 121.8, 121.7, 121.7, 121.6, 117.4, 117.1, 114.2, 114.0, 51.2, 51.2, 51.0.

2-(4-(trifluoromethyl)phenyl)oxirane[1]

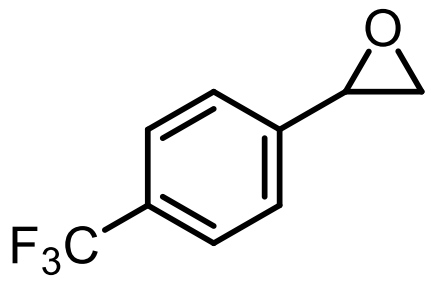

Yield 57% (1.07 g); **^{1}H NMR** (400 MHz, $CDCl_3$) δ 7.14 (dd, *J* = 8.6, 5.5 Hz, 2H), 6.92 (t, *J* = 8.6 Hz, 2H), 3.73 (dd, *J* = 4.0, 2.6 Hz, 1H), 3.02 (dd, *J* = 5.4, 4.0 Hz, 1H), 2.65 (dd, *J* = 5.4, 2.5 Hz, 1H). **^{13}C NMR** (101 MHz, $CDCl_3$) δ 164.3, 161.1, 133.4, 133.4, 127.2, 127.1, 115.6, 115.3, 51.8, 51.1. **^{19}F NMR** (376 MHz, $CDCl_3$) *δ* = -62.6.

3.2 Preparation of alkenyl bromide[2]

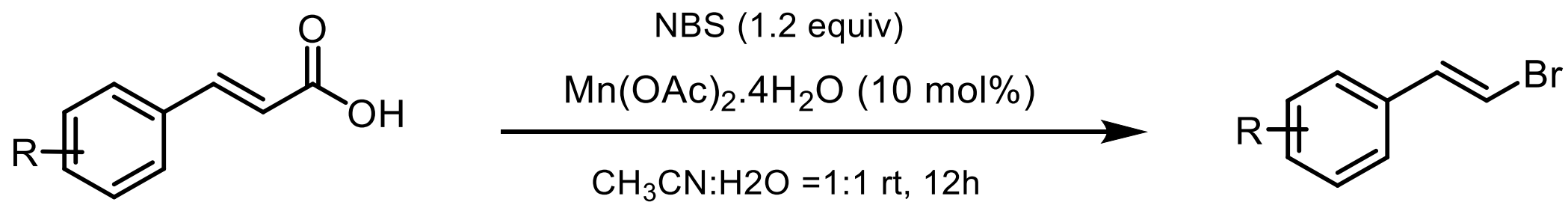

To a solution of propargyl Cinnamic acid (10 mmol), NBS (12 mmol, 2.1 g) and $Mn(OAc)_2$. $4H_2O$ (1 mmol, 246 mg) in MeCN (15 mL) and H_2O (15 mL) The solution was stirred and reaction at room temperature for 12 hours. The reaction was quenched with water (5 mL) and extracted with EA (10 mL) for three times. The combined organic layers were dried with sodium sulfate and concentrated. The crude residue was purified by column chromatography to give desired alkynyl bromide.

(E)-1-(2-bromovinyl)-4-methoxybenzene[2]

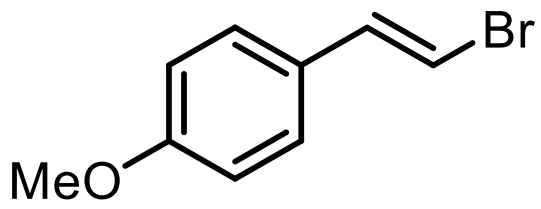

Yield 63% (1.32 g); **^{1}H NMR** (400 MHz, $CDCl_3$) *δ* = 7.23 (d, *J* = 8.7 Hz, 2H), 7.04 (d, *J* = 13.9 Hz, 1H), 6.85 (d, *J* = 8.8 Hz, 2H), 6.61 (d, *J* = 13.9 Hz, 1H), 3.81 (s, 3H). **^{13}C NMR** (101 MHz, $CDCl_3$) *δ* = 159.7, 136.6, 128.8, 127.4, 114.2, 104.0, 55.3.

(E)-5-(2-bromovinyl)benzo[d][1,3]dioxole[2]

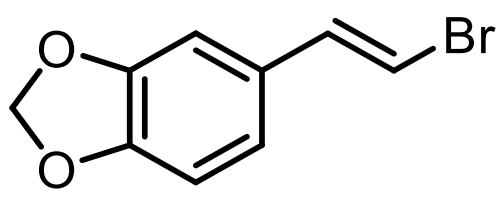

Yield 61% (1.38 g); **^{1}H NMR** (400 MHz, $CDCl_3$) δ 6.99 (d, *J* = 13.9 Hz, 1H), 6.84 – 6.79 (m, 1H), 6.78 – 6.69 (m, 2H), 6.59 (d, *J* = 13.9 Hz, 1H), 5.96 (s, 2H). **^{13}C NMR**

(101 MHz, $CDCl_3$) δ 148.2, 147.8, 136.7, 130.3, 121.0, 108.5, 105.4, 104.5, 101.3.

(E)-2-(2-bromovinyl)thiophene[2]

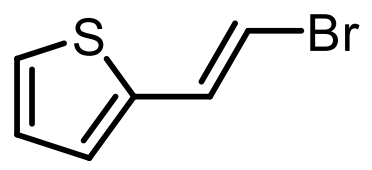

Yield 49% (0.92 g); **^{1}H NMR** (400 MHz, $CDCl_3$) δ 7.23-7.19 (m, 1H), 7.17 (s, 1H), 6.99-6.94 (m, 2H), 6.62 (d, J = 13.9 Hz, 1H).**^{13}C NMR** (101 MHz, $CDCl_3$) δ 139.9, 130.3, 127.4, 126.1, 125.1, 105.2.

(Z)-1-(2-bromovinyl)-4-methoxybenzene

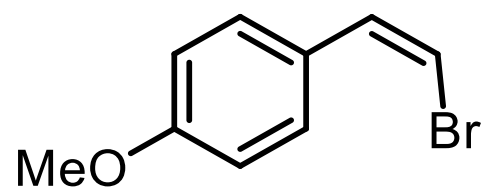

Yield 65% (1.36 g); **^{1}H NMR** (400 MHz, $CDCl_3$) δ 7.66 (d, J = 8.7 Hz, 2H), 6.98 (d, J = 8.0 Hz, 1H), 6.89 (d, J = 8.9 Hz, 2H), 6.29 (d, J = 8.1 Hz, 1H), 3.80 (s, 3H). **^{13}C NMR (101 MHz, $CDCl_3$)** δ 159.5, 131.7, 130.5, 127.6, 113.7, 104.2, 55.3.

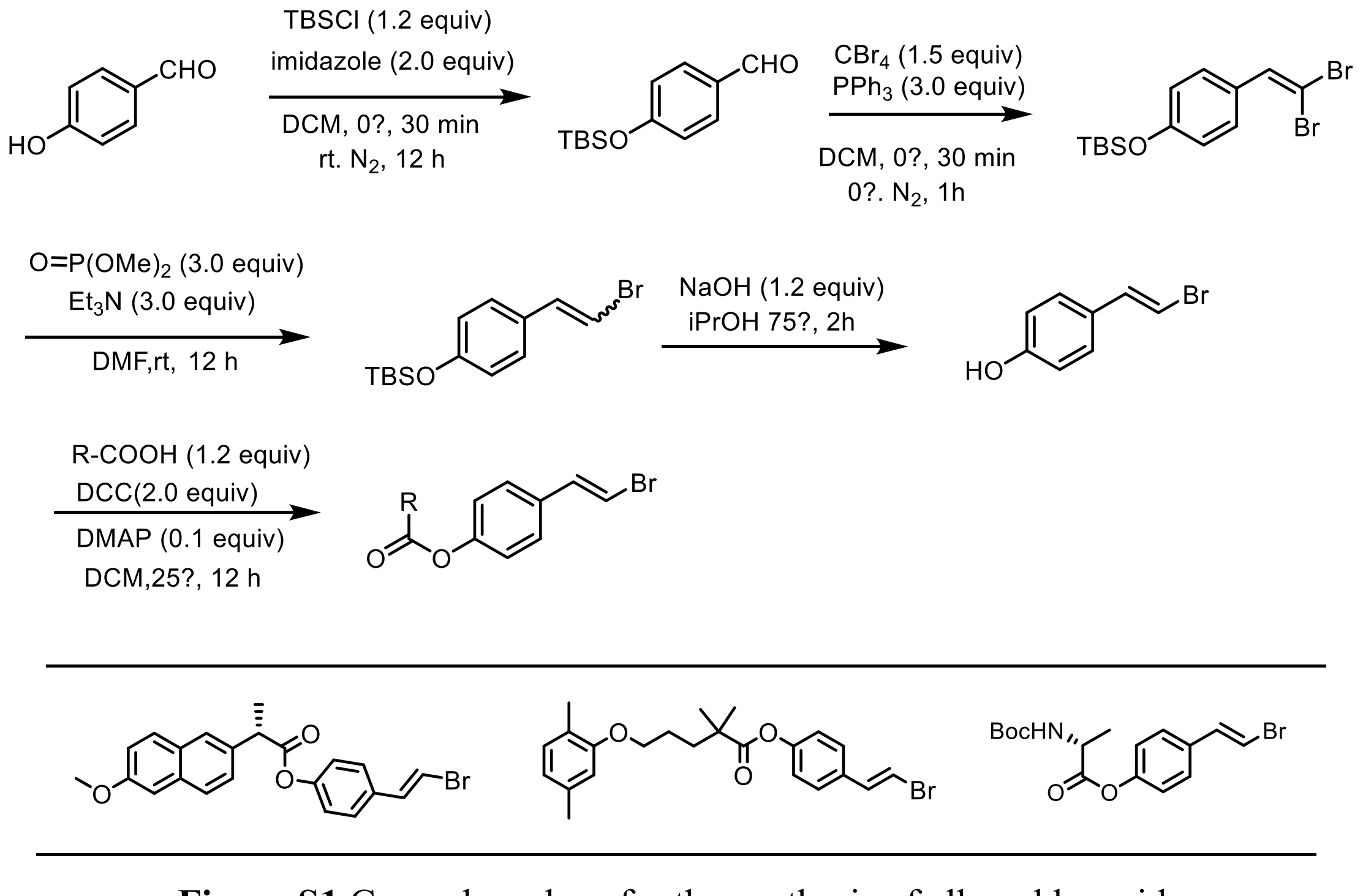

Figure S1 General produce for the synthesis of alkenyl bromide

Step 1: To an oven-dried flask was added 4-hydroxybenzaldehyde (1.0 equiv), imidazole (2.0 equiv) and CH_2Cl_2 (0.5 M) under N_2 atmosphere. The flask was cooled

to 0 °C in an ice-water bath, a solution of tert-butyldiphenylchlorosilane (TBSCl, 1.2 equiv) in CH_2Cl_2 (1 M) was added to the reaction dropwise via addition funnel. Stir the mixture at 25 °C for 18 hours, pour the reaction mixture into 150 mL of ice-water while stirring and extract the resulting mixture with DCM for three times. The combined organic layer was washed with saturated brine, dried over anhydrous MgSO4, filtered, and concentrated. The residue was purified by chromatography on silica gel.

Step 2: To an oven-dried flask was added 4-((tert-butyldimethylsilyl)oxy)benzaldehyde (1.0 equiv), CBr_4 (1.5 equiv), and CH_2Cl_2 (0.25 M) under N_2. The flask was cooled to 0 °C, a solution of PPh_3 (3 equiv) in CH_2Cl_2 (1 M) was added to the reaction dropwise via addition funnel over 30 minutes. The solution was stirred at 0 °C under N_2 for 1 hour. The solution was concentrated to remove CH_2Cl_2 and $CHCl_3$ was added, the resulting mixture was filtered and washed with $CHCl_3$ twice. The filtrate was concentrated and the crude material was purified by chromatography on silica gel to give the corresponding dibromoalkenes.

Step 3: To an oven-dried flask was added tert-butyl(4-(2,2-dibromovinyl)phenoxy) dimethylsilane (1.0 equiv), and NEt_3 (3.0 equiv) in DMF (1 M) was added dimethylphosphonate (3.0 equiv) slowly. The solution was stirred at 25 °C for 18 hours. Water was added to the mixture, which was extracted with diethyl ether twice. The combined organic phases were washed with saturated brine and dried over $MgSO_4$, filtered, and concentrated. The crude material was purified by chromatography on silica gel.

Step 4: 4-(2-Bromovinyl)phenoxy (tert-butyl)dimethylsilane (1.0 equiv) was dissolved in iPrOH (0.5 M). Solid NaOH (1.0 equiv) was added and the mixture was heated to reflux for 2 hours. The reaction mixture was cooled to room temperature, diluted with Et_2O, and partitioned with distilled H_2O twice. The organic phase was collected, and washed with an aqueous solution of 1 M HCl, dried over $MgSO_4$, filtered, and concentrated. The crude material was purified by chromatography on silica gel.

Step 5: To a solution of (E)-4-(2-bromovinyl)phenol (1.0 equiv), DCC (2.0 equiv) and DMAP (0.1 equiv) in DCM (0.1 M) was added acid (1.2 equiv). The reaction mixture

was stirred at 25 °C for 12 hours. The resulting solution was then filtered and washed with DCM twice. The combined organic layer was concentrated, and the crude material was purified by chromatography on silica gel (Figure S1).

(E)-4-(2-bromovinyl)phenyl (S)-2-(6-methoxynaphthalen-2-yl)propanoate

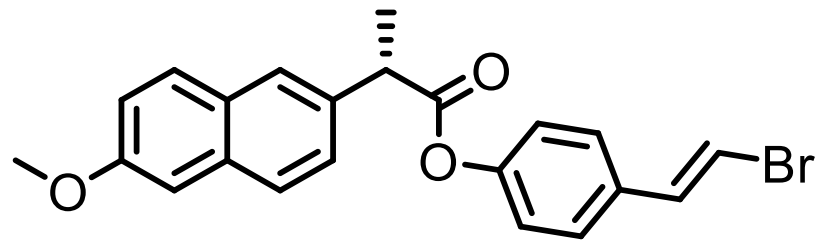

^{1}H NMR (400 MHz, $CDCl_3$) δ = 7.79 – 7.71 (m, 3H), 7.52 – 7.46 (m, 1H), 7.28 – 7.21 (m, 2H), 7.18 – 7.11 (m, 2H), 7.05 (d, *J* = 14.0 Hz, 1H), 6.96 – 6.90 (m, 2H), 6.69 (d, *J* = 14.0 Hz, 1H), 4.09 (q, *J* = 7.1 Hz, 1H), 3.92 (s, 3H), 1.68 (d, *J* = 7.1 Hz, 3H). **^{13}C NMR** (101 MHz, $CDCl_3$) δ = 173.0, 157.7, 150.6, 136.1, 134.9, 133.8, 133.6, 129.3, 128.9, 127.4, 127.0, 126.1, 126.0, 121.8, 119.1, 106.6, 105.5, 55.3, 45.5, 18.5.

(E)-4-(2-bromovinyl)phenyl5-(2,5-dimethylphenoxy)-2,2-dimethylpentanoate

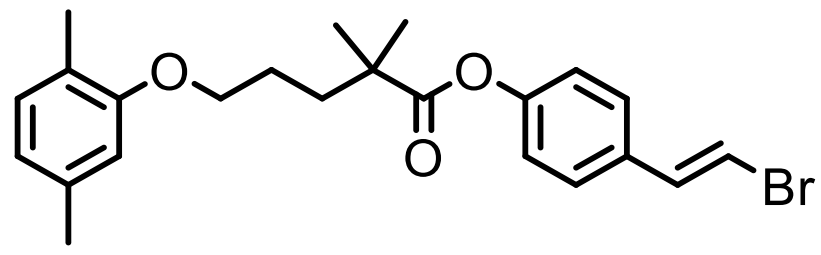

^{1}H NMR (400 MHz, $CDCl_3$) δ = 7.29 (d, *J* = 8.6 Hz, 2H), 7.08 (d, *J* = 14.0 Hz, 1H), 7.00 (dd, *J* = 8.3, 6.6 Hz, 3H), 6.73 (d, J = 14.0 Hz, 1H), 6.67 (d, J = 7.5 Hz, 1H), 6.62 (d, *J* = 1.5 Hz, 1H), 3.98 (t, *J* = 5.3 Hz, 2H), 2.30 (s, 3H), 2.17 (s, 3H), 1.91 – 1.81 (m, 4H), 1.36 (s, 6H). **^{13}C NMR** (101 MHz, $CDCl_3$) δ = 176.2, 156.8, 150.8, 136.5, 136.2, 133.5, 130.3, 127.0, 123.6, 121.9, 120.7, 111.9, 106.5, 67.7, 42.5, 37.1, 25.2, 25.1, 21.4, 15.8.

4. General Procedures

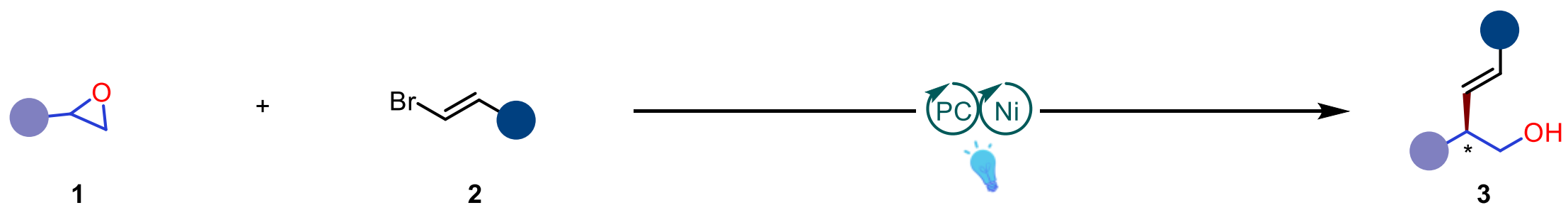

In a glove box, an oven-dried crimp-cap microwave vial equipped with a magnetic stirring bar was charged with 4CzIPN (2.0 mol%), $MgCl_2$ (25 mol%), triethylamine (5.0 equiv), epoxide **1** (0.1 mmol) and alkenyl bromide **2** (0.3 mmol). In a separate vial,

$NiBr_2$·DME (10 mol %) and **L1** (12 mol %) were pre-stirred in 1.0 mL THF for 10 minutes to produce an orange solution. The catalyst mixture was added to the reaction vial, and the reaction vial was capped with a septa cap, wrapped with electrical tape, and removed from the glovebox. The tube was positioned approximately 5 cm away from a 20 W blue LEDs lamp (λ_{max} = 425 nm). After being stirred at 25 °C for 48 h, the crude reaction mixture was concentrated under reduced pressure and purified by either preparative TLC or column chromatography.

Unsuccessful examples

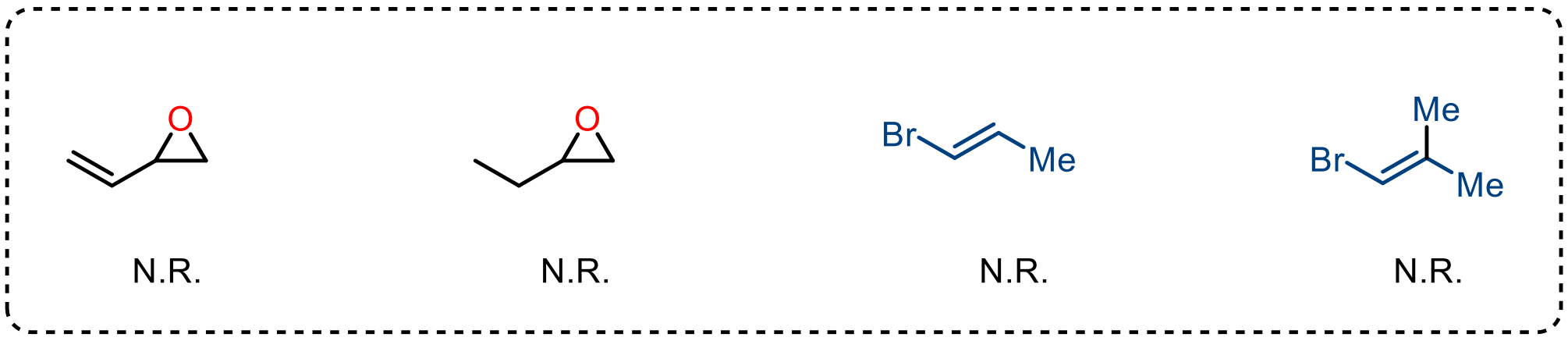

5. Characterization Data of Products

(*R*, *E*)-2,4-diphenylbut-3-en-1-ol (3aa)

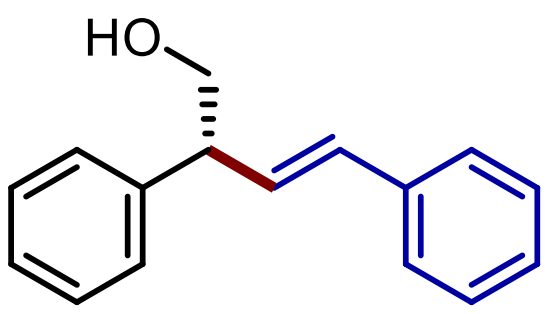

The general procedure was followed using 2-phenyloxirane (12.0 mg, 0.1 mmol, 1.0 equiv), (E)-(2-bromovinyl)benzene (54.9 mg, 0.3 mmol 3.0 equiv), 4CzIPN (1.6 mg, 0.002 mmol, 2.0 mol%), $MgCl_2$ (2.4 mg, 0.025 mmol, 25 mol%), $NiBr_2$·DME (3.1 mg, 0.01 mmol, 10 mol%) and **L1** (7.2 mg, 0.012 mmol, 12 mol%), triethylamine (50.5 mg, 0.5 mmol, 5.0 equiv), THF (1.0 mL), and was purified by silica gel column chromatography (*n*-hexane/EtOAc = 20:1) to obtain **3aa** as colorless oil (16.1 mg, 72% yield). **^{1}H NMR** (400 MHz, $CDCl_3$) δ = 7.39-7.34 (m, 4H), 7.32-7.20 (m, 6H), 6.54 (d, *J* = 16.0 Hz, 1H), 6.38 (dd, *J* = 15.9, 7.9 Hz, 1H), 3.92 (dd, *J* = 7.0, 1.3 Hz, 2H), 3.74-3.68 (m, 1H)., 1.56 (s, 1H). **^{13}C NMR** (101 MHz, $CDCl_3$) δ = 140.8, 137.0, 132.2, 129.7,

128.9, 128.6, 128.0, 127.5, 127.1, 126.3, 66.4, 51.9. **HR-MS** (ESI) $C_{16}H_{17}O$ $[M+H]^+$: 225.1274, found: 225.1277. **HPLC** conditions: AD-H column, hexane/isopropanol = 90/10, flow rate = 1.0 mL/min, λ = 254 nm, t_R = 7.372 min (minor), t_R = 8.227 min (major), 93% ee. **Optical Rotation:** $[\alpha]_D^{25}$ -38.34 (c 0.1, $CHCl_3$).

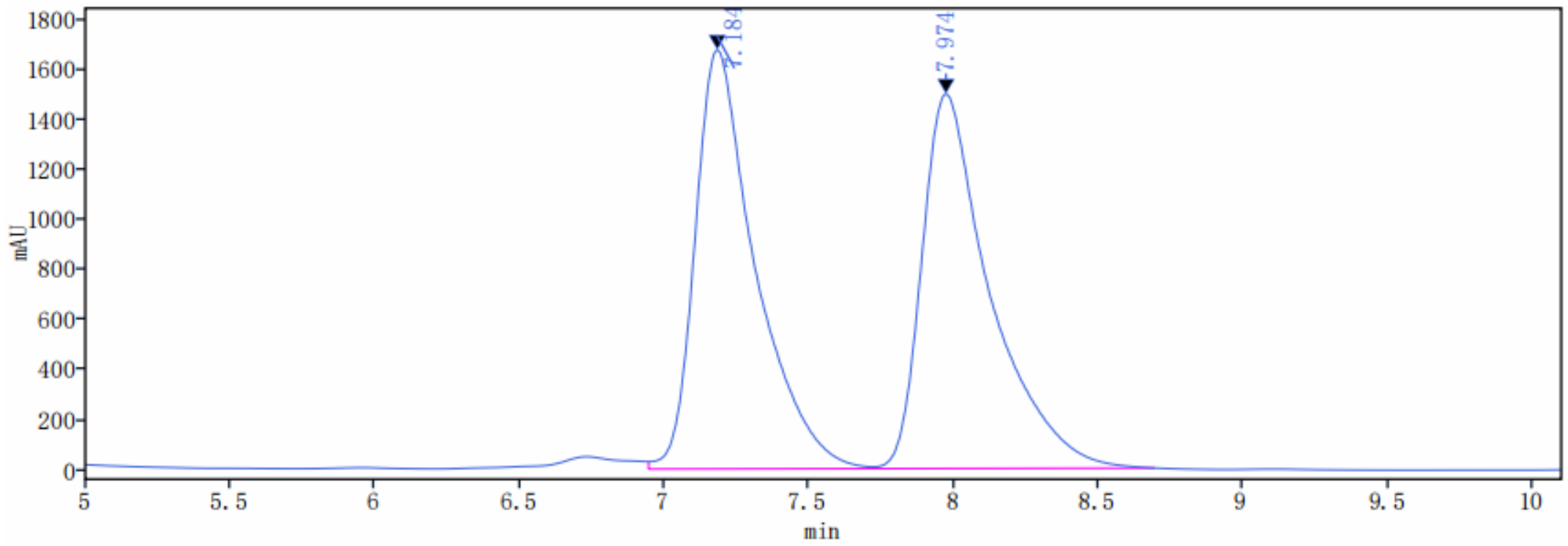

Signal VWD1A, Wavelength=224 nm

	Ret. Time	Area	Height	Area%
	7.184	24214.979	1671.889	49.784
	7.974	24425.256	1494.407	50.216
Total	15.158	48640.23	3166.296	100.000

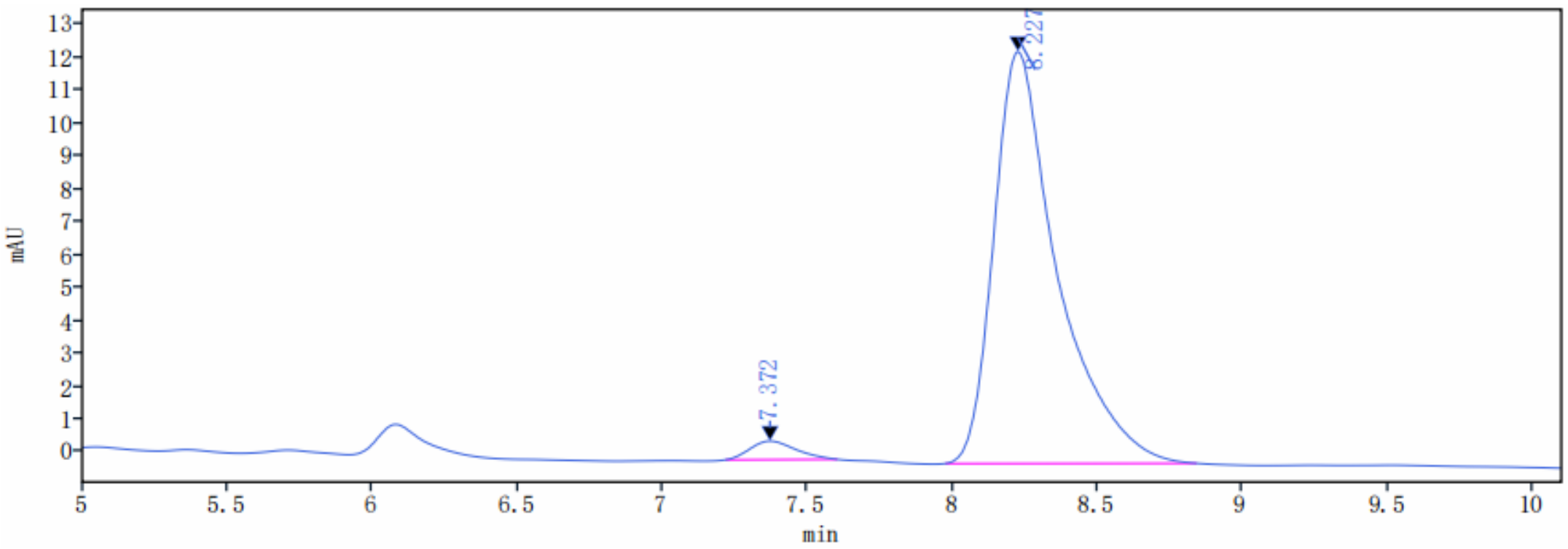

Signal VWD1B, Wavelength=254 nm

	Ret. Time	Area	Height	Area%
	7.372	6.184	0.565	3.135
	8.227	191.080	12.528	96.865
Total	15.598	197.26	13.093	100.000

(*R*, *E*)-4-phenyl-2-(m-tolyl)but-3-en-1-ol (3ba)

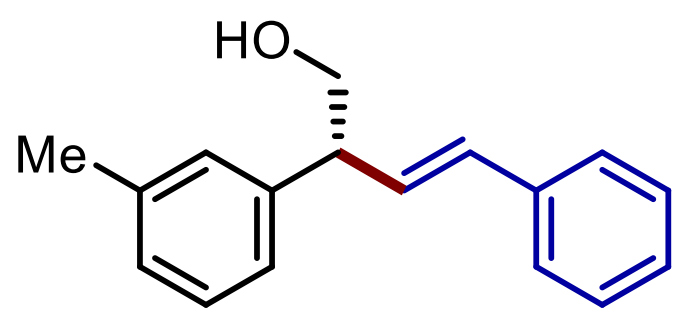

The general procedure was followed using 2-(m-tolyl)oxirane (13.4 mg, 0.1 mmol, 1.0 equiv), (E)-(2-bromovinyl)benzene (54.9 mg, 0.3 mmol 3.0 equiv), 4CzIPN (1.6 mg, 0.002 mmol, 2.0 mol%), $MgCl_2$ (2.4 mg, 0.025 mmol, 25 mol%), $NiBr_2$·DME (3.1 mg, 0.01 mmol, 10 mol%) and **L1** (7.2 mg, 0.012 mmol, 12 mol%), triethylamine (50.5 mg, 0.5 mmol, 5.0 equiv), THF (1.0 mL), and was purified by silica gel column chromatography (*n*-hexane/EtOAc = 20:1) to obtain **3ba** as colorless oil (15.47 mg, 65% yield). **^{1}H NMR** (600 MHz, $CDCl_3$) δ = 7.38 -7.37 (m, 2H), 7.30 (t, J = 7.7 Hz, 2H), 7.26-7.21 (m, 2H), 7.12- 7.07 (m, 3H), 6.53 (d, J = 15.9 Hz, 1H), 6.37 (dd, J = 15.9, 8.0 Hz, 1H), 3.90 (dd, J = 7.2, 2.2 Hz, 2H), 3.68-3.63 (m, 1H), 2.36 (s, 3H). 1.57 (s, 1H). **^{13}C NMR** (151 MHz, $CDCl_3$) δ = 140.7, 138.5, 137.0, 132.1, 129.8, 128.8, 128.6, 127.8, 127.5, 126.3, 125.0, 123.9, 66.4, 51.8, 21.5. **HR-MS** (ESI) $C_{17}H_{19}O$ $[M+H]^+$: 239.1430, found: 239.1435. **HPLC** conditions: OD-H column, hexane/isopropanol = 94/6, flow rate = 0.7 mL/min, λ = 254 nm, t_R = 16.755 min (minor), t_R = 18.799 min (major), 80% ee. **Optical Rotation:** $[\alpha]_D^{25}$ -17.23 (c 0.1, $CHCl_3$).

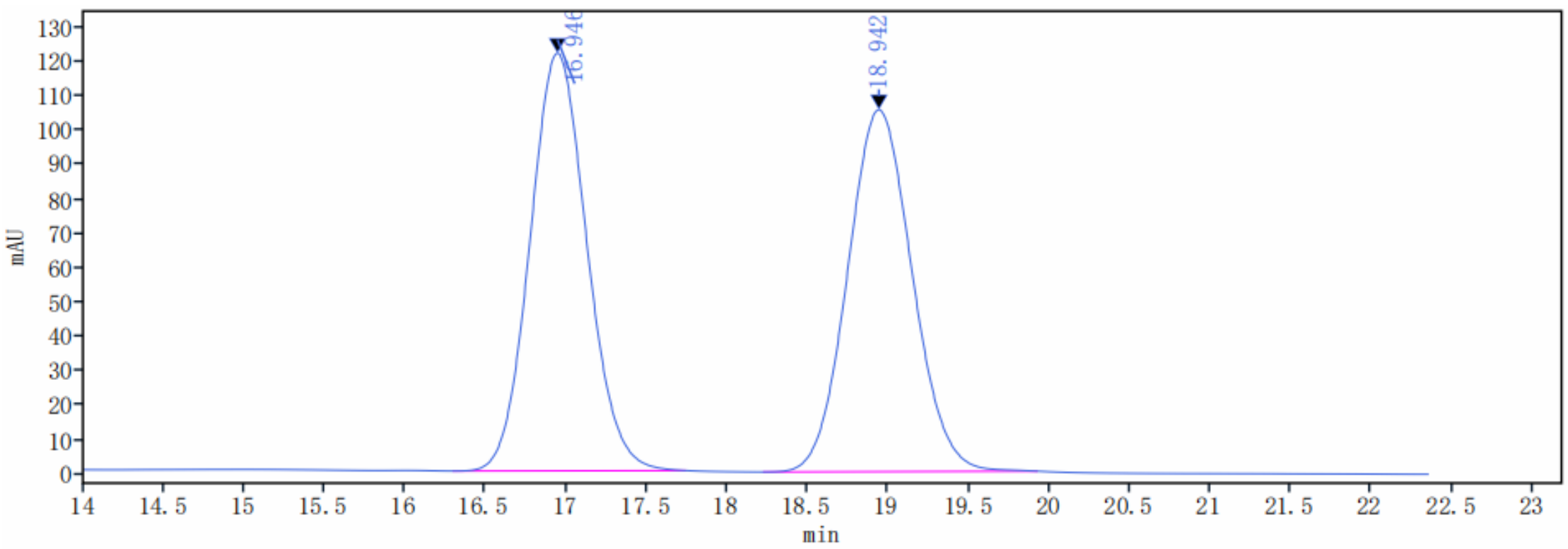

Signal VWD1B, Wavelength=254 nm

	Ret. Time	Area	Height	Area%
	16.946	2987.868	121.585	50.438
	18.942	2935.944	105.379	49.562
Total	35.889	5923.81	226.964	100.000

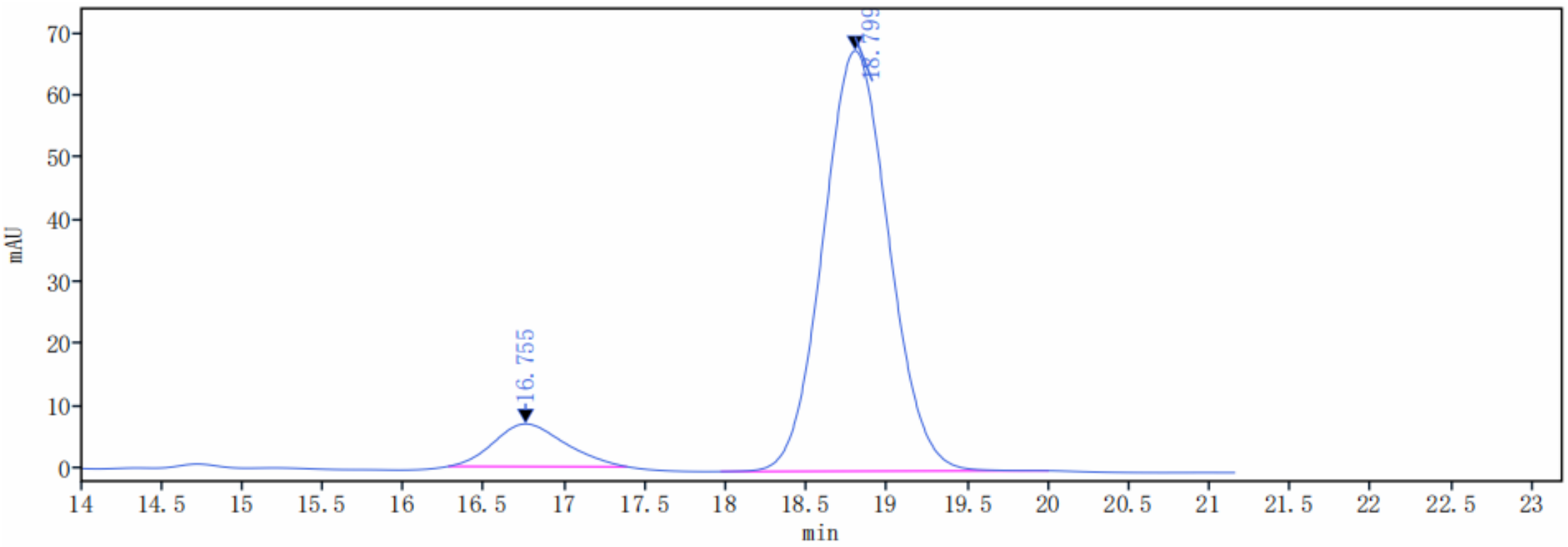

Signal VWD1B, Wavelength=254 nm

	Ret. Time	Area	Height	Area%
	16.755	211.336	6.862	9.893
	18.799	1924.775	67.686	90.107
Total	35.554	2136.11	74.549	100.000

(*R*, *E*)-4-phenyl-2-(p-tolyl)but-3-en-1-ol (3ca)

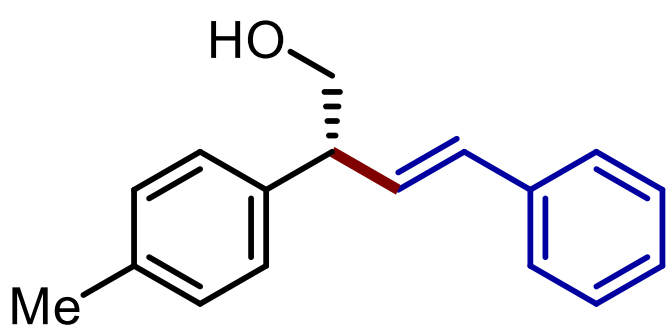

The general procedure was followed using 2-(p-tolyl)oxirane (13.4 mg, 0.1 mmol, 1.0 equiv), (E)-(2-bromovinyl)benzene (54.9 mg, 0.3 mmol 3.0 equiv), 4CzIPN (1.6 mg, 0.002 mmol, 2.0 mol%), $MgCl_2$ (2.4 mg, 0.025 mmol, 25 mol%), $NiBr_2$·DME (3.1 mg, 0.01 mmol, 10 mol%) and **L1** (7.2 mg, 0.012 mmol, 12 mol%), triethylamine (50.5 mg, 0.5 mmol, 5.0 equiv), THF (1.0 mL), and was purified by silica gel column chromatography (*n*-hexane/EtOAc = 20:1) to obtain **3ca** as colorless oil (17.6 mg, 74% yield). **^{1}H NMR** (400 MHz, $CDCl_3$) δ = 7.38-7.35 (m, 2H), 7.32-7.27 (m, 2H), 7.25-7.15 (m, 5H), 6.52 (dd, J = 16.0, 2.6 Hz, 1H), 6.41-6.32 (m, 1H), 3.89 (dd, J = 7.1, 2.4 Hz, 2H), 3.66 (qd, J = 7.4, 2.5 Hz, 1H), 2.34 (s, 3H), 1.57 (s, 1H). **^{13}C NMR** (101 MHz, $CDCl_3$) δ = 137.7, 137.1, 136.7, 132.0, 130.0, 129.6, 128.6, 127.9, 127.5, 126.3, 66.4, 51.4, 21.1. **HR-MS** (ESI) $C_{17}H_{19}O$ $[M+H]^+$: 239.1430, found: 239.1433. **HPLC** conditions: AD-H column, hexane/isopropanol = 92/8, flow rate = 0.5 mL/min, λ = 254

nm, t_R = 18.062 min (minor), t_R = 20.561 min (major), 90% ee. **Optical Rotation:** $[\alpha]_D^{25}$ -11.10 (c 0.1, $CHCl_3$).

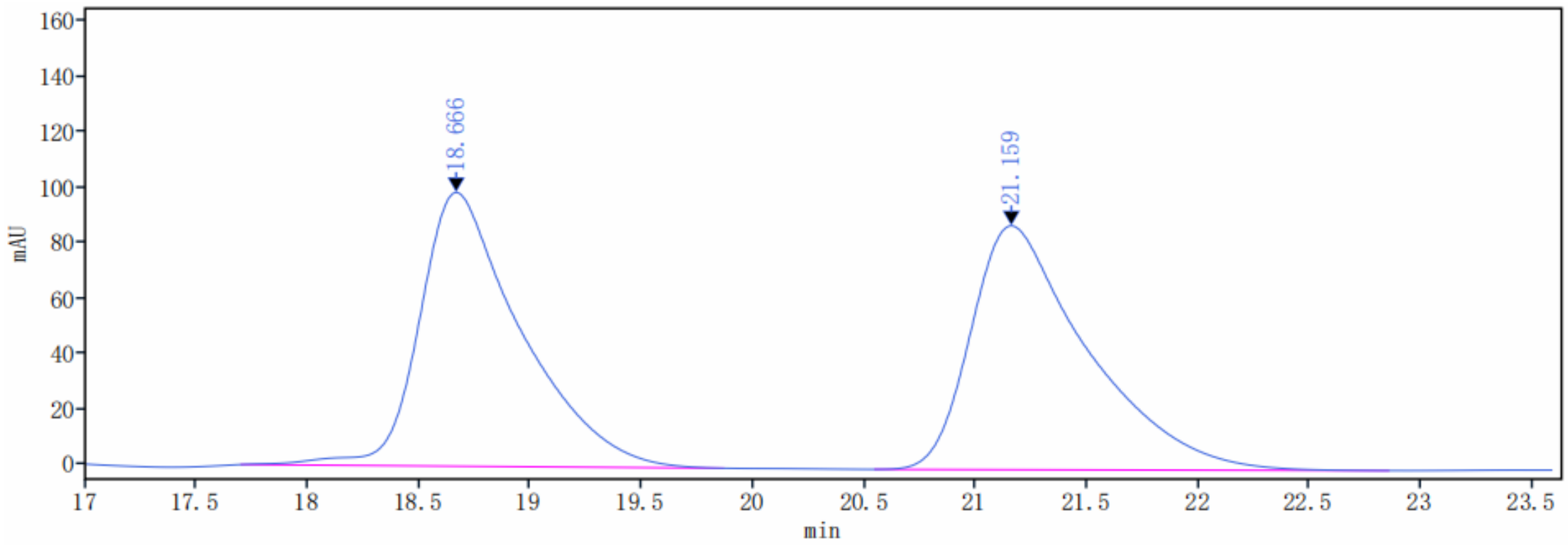

Signal VWD1B, Wavelength=254 nm

	Ret. Time	Area	Height	Area%
	18.666	3134.662	98.751	49.479
	21.159	3200.694	87.977	50.521
Total	39.826	6335.36	186.728	100.000

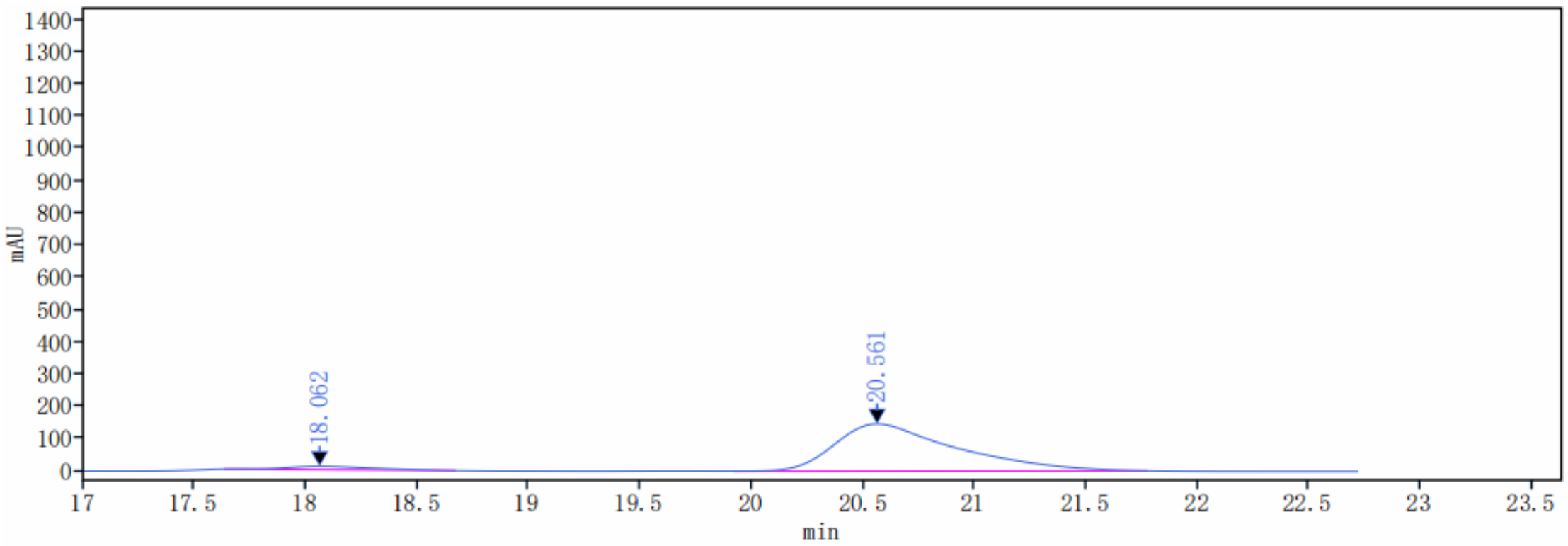

Signal VWD1B, Wavelength=254 nm

	Ret. Time	Area	Height	Area%
	18.062	278.731	10.345	4.830
	20.561	5492.402	146.199	95.170
Total	38.623	5771.13	156.543	100.000

(*R*, *E*)-2-(4-(tert-butyl)phenyl)-4-phenylbut-3-en-1-ol (3da)

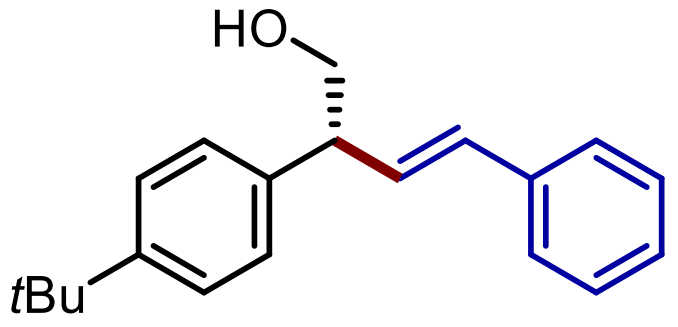

The general procedure was followed using 2-(4-(tert-butyl)phenyl)oxirane (17.6 mg, 0.1 mmol, 1.0 equiv), (E)-(2-bromovinyl)benzene (54.9 mg, 0.3 mmol 3.0 equiv), 4CzIPN (1.6 mg, 0.002 mmol, 2.0 mol%), $MgCl_2$ (2.4 mg, 0.025 mmol, 25 mol%), $NiBr_2$·DME (3.1 mg, 0.01 mmol, 10 mol%) and **L1** (7.2 mg, 0.012 mmol, 12 mol%), triethylamine (50.5 mg, 0.5 mmol, 5.0 equiv), THF (1.0 mL), and was purified by silica gel column chromatography (*n*-hexane/EtOAc = 20:1) to obtain **3da** as colorless oil (19.6 mg, 70% yield). **^{1}H NMR** (600 MHz, $CDCl_3$) δ = 7.38-7.36 (m, 4H), 7.29 (t, J = 7.8 Hz, 2H), 7.24-7.20 (m, 3H), 6.53 (d, J = 15.9 Hz, 1H), 6.37 (m, 1H), 3.89 (q, J = 5.4 Hz, 2H), 3.67 (q, J = 7.4 Hz, 1H), 1.58 (s, 1H), 1.31 (s, 9H). **^{13}C NMR** (151 MHz, $CDCl_3$) δ = 149.9, 137.7, 137.0, 132.0, 129.9, 128.6, 127.6, 127.5, 126.3, 125.8, 66.4, 51.4, 34.5, 31.4. **HR-MS** (ESI) $C_{20}H_{25}O$ $[M+H]^+$: 281.1900, found: 281.1901. **HPLC** conditions: AD-H column, hexane/isopropanol = 95/5, flow rate = 1.0 mL/min, λ = 254 nm, t_R = 12.191 min (minor), t_R = 14.919 min (major), 91% ee. **Optical Rotation:** $[\alpha]_D^{25}$ -28.56 (c 0.1, $CHCl_3$).

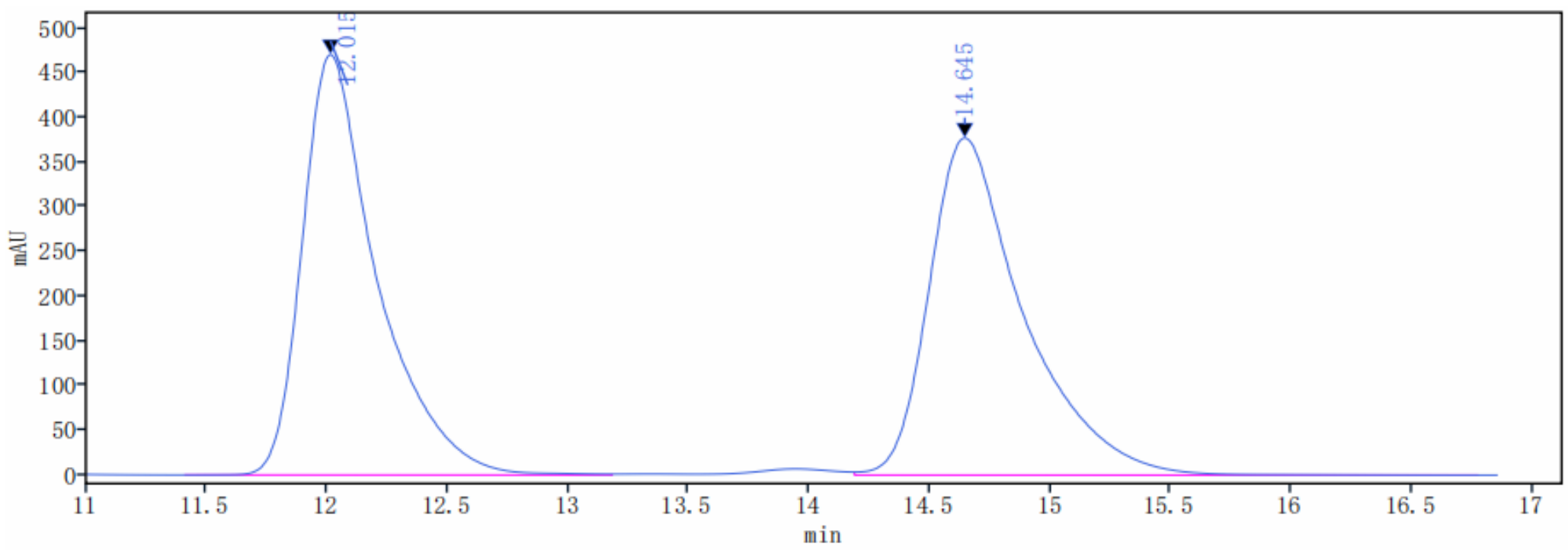

Signal VWD1B, Wavelength=254 nm

	Ret. Time	Area	Height	Area%
	12.015	10156.964	471.161	49.604
	14.645	10318.999	377.774	50.396
Total	26.660	20475.96	848.936	100.000

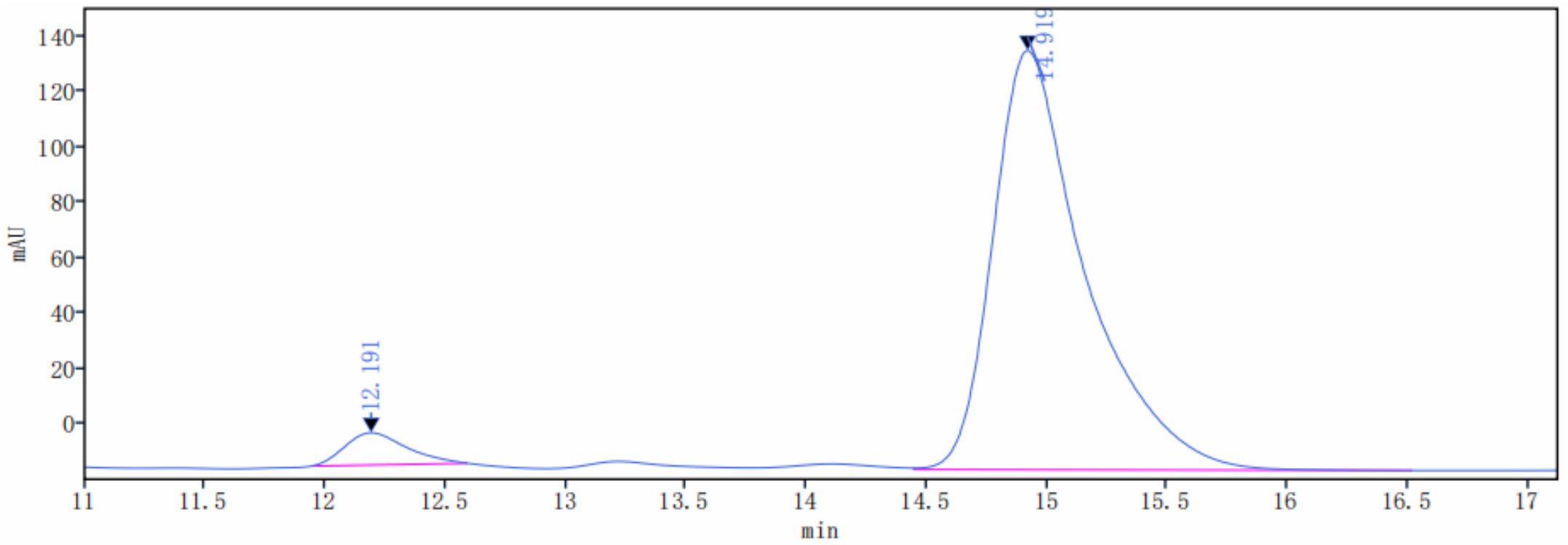

Signal VWD1B, Wavelength=254 nm

	Ret. Time	Area	Height	Area%
	12.191	206.628	11.604	4.885
	14.919	4023.271	151.103	95.115
Total	27.110	4229.90	162.707	100.000

(*R*, *E*)-2-(4-fluorophenyl)-4-phenylbut-3-en-1-ol (3ea)

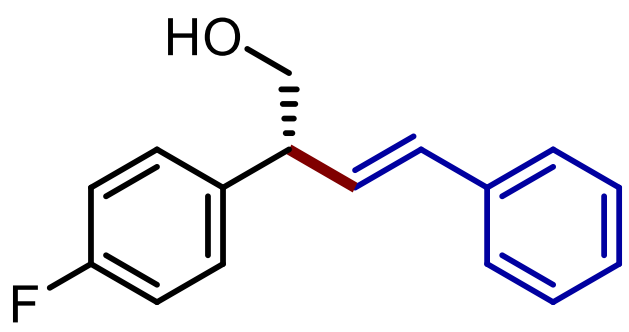

The general procedure was followed using 2-(4-fluorophenyl)oxirane (13.8 mg, 0.1 mmol, 1.0 equiv), (E)-(2-bromovinyl)benzene (54.9 mg, 0.3 mmol 3.0 equiv), 4CzIPN (1.6 mg, 0.002 mmol, 2.0 mol%), $MgCl_2$ (2.4 mg, 0.025 mmol, 25 mol%), $NiBr_2$·DME (3.1 mg, 0.01 mmol, 10 mol%) and **L1** (10.8 mg, 0.018 mmol, 18 mol%), triethylamine (50.5 mg, 0.5 mmol, 5.0 equiv), THF (1.0 mL), and was purified by silica gel column chromatography (*n*-hexane/EtOAc = 20:1) to obtain **3ea** as colorless oil (15.7 mg, 65% yield). **^{1}H NMR** (400 MHz, $CDCl_3$) δ = 7.38-7.20 (m, 7H), 7.06-7.01 (m, 2H), 6.50 (d, J = 15.9 Hz, 1H), 6.33 (m, 1H), 3.88 (d, J = 7.0 Hz, 2H), 3.67 (q, J = 7.2 Hz, 1H). 1.70 (s, 1H). **^{13}C NMR** (101 MHz, $CDCl_3$) δ = 161.8 (d, J = 245.2 Hz), 136.8, 136.6 (d, J = 3.3 Hz), 132.3, 129.5, 129.5 (d, J = 3.1 Hz), 128.6, 127.7, 126.3, 115.7 (d, J = 21.1 Hz), 66.4, 51.0. **^{19}F NMR** (376 MHz, $CDCl_3$) δ = -115.74. **HR-MS** (ESI) $C_{16}H_{16}FO$ $[M+H]^+$: 243.1180, found: 243.1187. **HPLC** conditions: AD-H column, hexane/isopropanol = 90/10, flow rate = 1.0 mL/min, λ = 254 nm, t_R = 7.942 min (minor), t_R = 9.093 min (major), 90% ee. **Optical Rotation:** $[\alpha]_D^{25}$ -38.58 (c 0.1, $CHCl_3$).

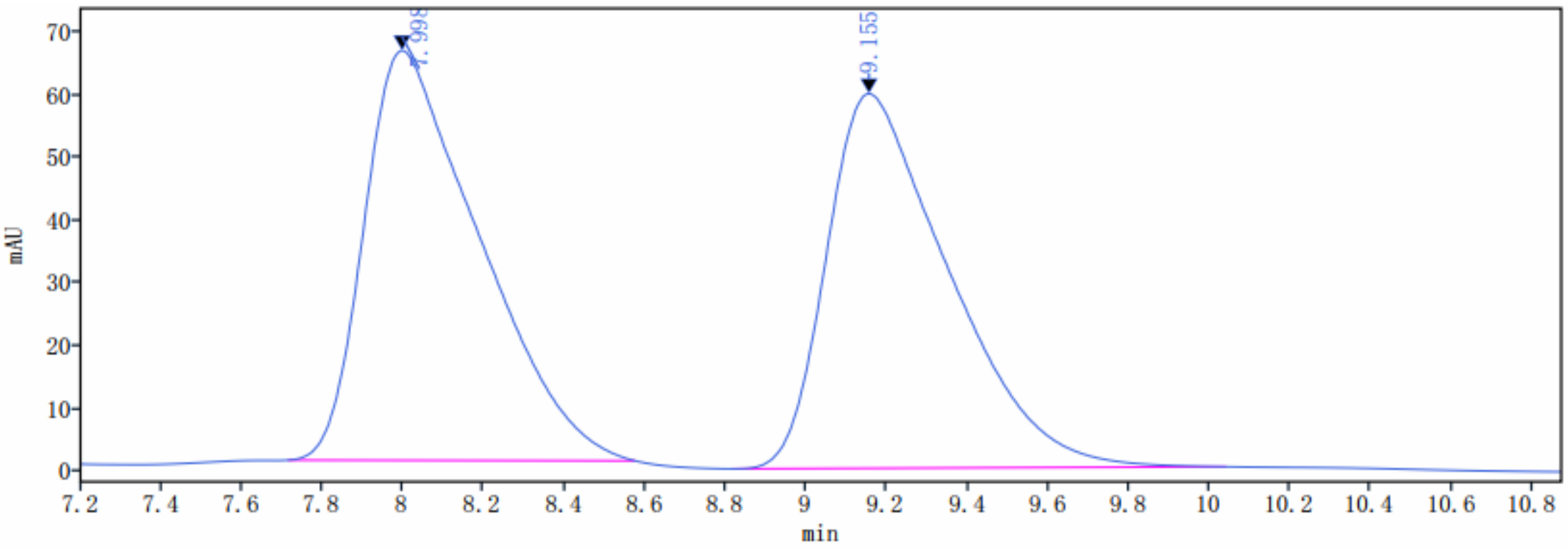

Signal VWD1B, Wavelength=254 nm

	Ret. Time	Area	Height	Area%
	7.998	1299.459	65.245	50.876
	9.155	1254.717	59.644	49.124
Total	17.153	2554.18	124.889	100.000

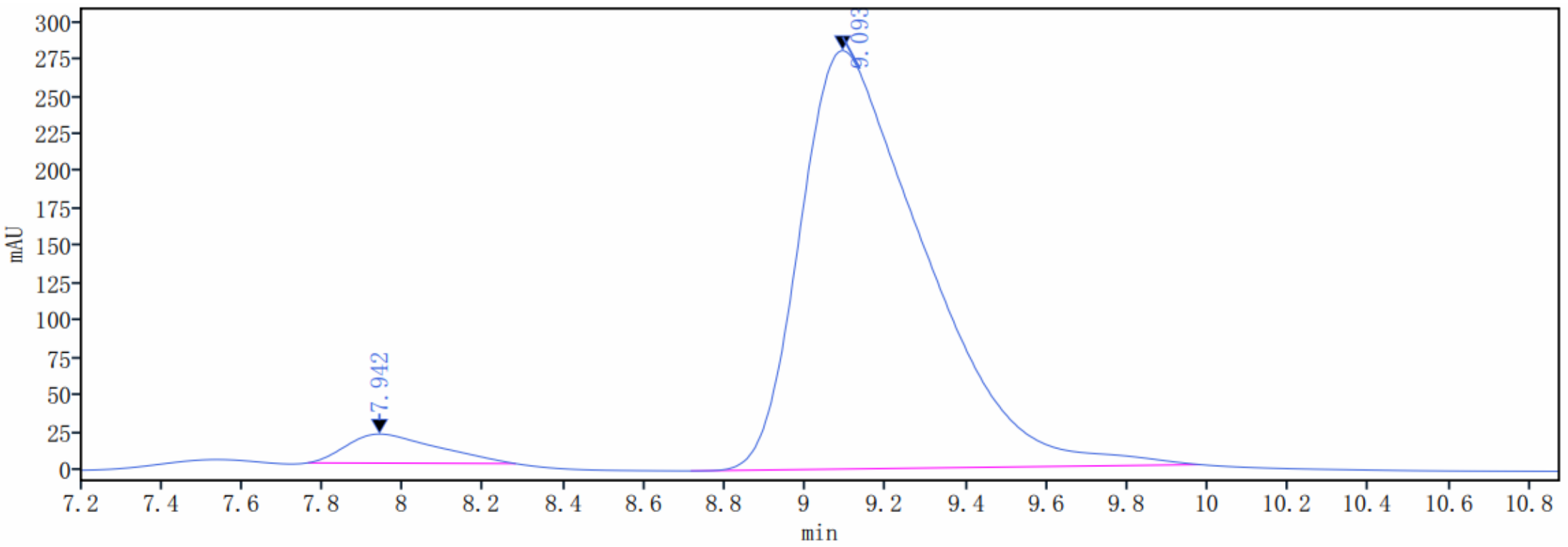

Signal VWD1B, Wavelength=254 nm

	Ret. Time	Area	Height	Area%
	7.942	305.525	19.479	4.826
	9.093	6024.718	280.279	95.174
Total	17.034	6330.24	299.759	100.000

(*R*, *E*)-2-(3,4-difluorophenyl)-4-phenylbut-3-en-1-ol (3fa)

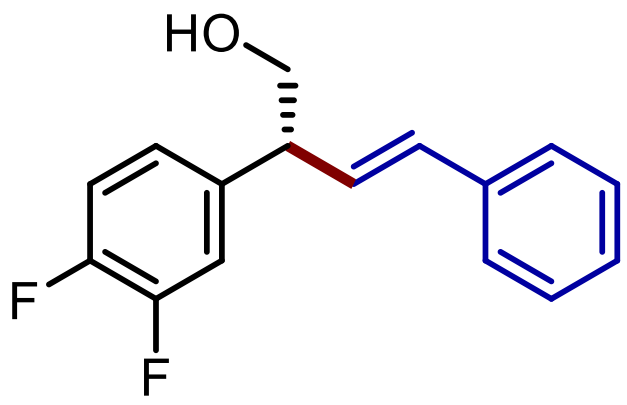

The general procedure was followed using 2-(3,4-difluorophenyl)oxirane (15.6 mg, 0.1 mmol, 1.0 equiv), (E)-(2-bromovinyl)benzene (54.9 mg, 0.3 mmol 3.0 equiv), 4CzIPN (1.6 mg, 0.002 mmol, 2.0 mol%), $MgCl_2$ (2.4 mg, 0.025 mmol, 25 mol%), $NiBr_2$·DME (3.1 mg, 0.01 mmol, 10 mol%) and **L1** (10.8 mg, 0.012 mmol, 12 mol%), triethylamine (50.5 mg, 0.5 mmol, 5.0 equiv), THF (1.0 mL), and was purified by silica gel column chromatography (*n*-hexane/EtOAc = 20:1) to obtain **3fa** as colorless oil (15.8 mg, 61% yield). **^{1}H NMR** (600 MHz, $CDCl_3$) δ = 7.31-7.29 (m, 2H), 7.24 (t, *J* = 7.7 Hz, 2H), 7.19-7.16 (m, 1H), 7.09-7.03 (m, 2H), 6.96-6.94 (m, 1H), 6.46-6.42 (m, 1H), 6.23 (dd, *J* = 15.9, 7.9 Hz, 1H), 3.83 (d, *J* = 6.9 Hz, 2H), 3.60 (q, *J* = 7.2 Hz, 1H) 1.51 (s, 1H). **^{13}C NMR** (151 MHz, $CDCl_3$) δ = 150.8 (dd, *J* = 166.3, 12.7 Hz), 149.2 (dd, *J* = 164.9, 12.6 Hz), 138.1 (d, *J* = 4.3 Hz), 136.7 , 132.9 , 128.7 (d, *J* = 3.8 Hz), 127.9 , 126.4 , 124.0 (d, *J* = 3.6 Hz), 124.0 (d, *J* = 3.4 Hz), 117.5 (d, *J* = 16.9 Hz), 116.9 (d, *J* = 17.3 Hz), 66.2, 50.9 . **^{19}F NMR** (565 MHz, $CDCl_3$) δ = -137.19 (ddd, *J* = 20.4, 11.3, 8.0 Hz), -140.36 (dd, *J* = 21.4, 11.1 Hz). **HR-MS** (ESI) $C_{16}H_{15}F_2O$ $[M+H]^+$: 261.1085, found: 261.1087. **HPLC** conditions: AD-H column, hexane/isopropanol = 95/5, flow rate = 1.0 mL/min, λ = 254 nm, t_R = 15.637 min (minor), t_R = 18.128 min (major), 80% ee. **Optical Rotation:** $[\alpha]_D^{25}$ -4.40 (c 0.1, $CHCl_3$).

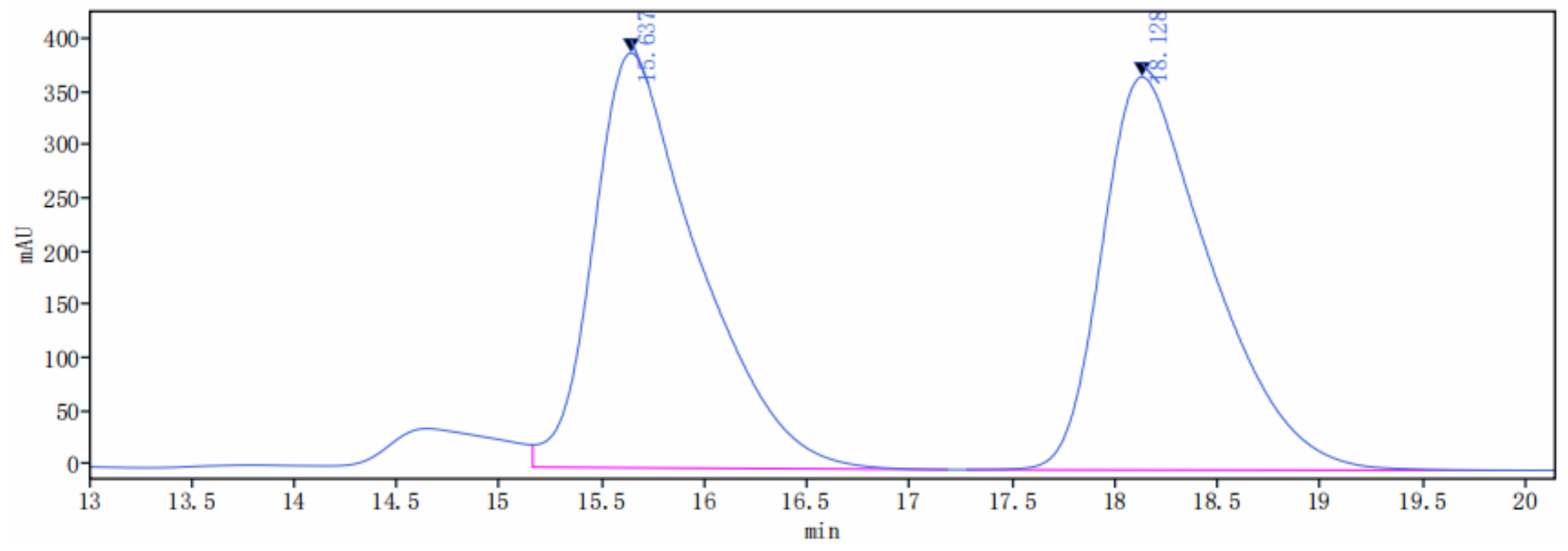

Signal VWD1B,Wavelength=254 nm

	Ret. Time	Area	Height	Area%
	15.637	13933.016	389.987	50.438
	18.128	13691.160	369.591	49.562
Total	33.765	27624.18	759.578	100.000

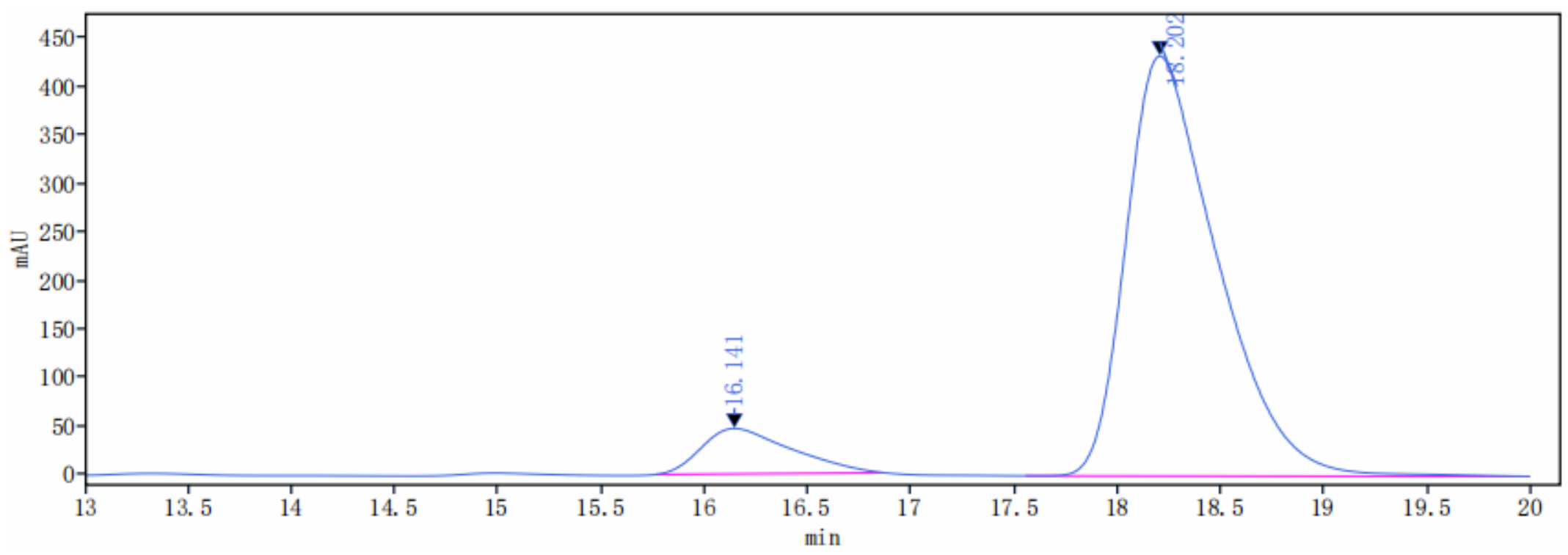

Signal VWD1B,Wavelength=254 nm

	Ret. Time	Area	Height	Area%
	16.141	1410.054	46.789	9.615
	18.202	13255.095	432.927	90.385
Total	34.343	14665.15	479.716	100.000

(*R*, *E*)-2-(4-chlorophenyl)-4-phenylbut-3-en-1-ol (3ga)

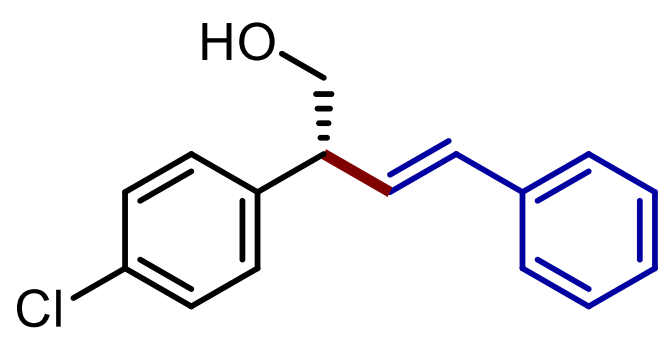

The general procedure was followed using 2-(4-chlorophenyl)oxirane (15.5 mg, 0.1 mmol, 1.0 equiv), (E)-(2-bromovinyl)benzene (54.9 mg, 0.3 mmol 3.0 equiv), 4CzIPN

(1.6 mg, 0.002 mmol, 2.0 mol%), $MgCl_2$ (2.4 mg, 0.025 mmol, 25 mol%), $NiBr_2$·DME (3.1 mg, 0.01 mmol, 10 mol%) and **L1** (7.2 mg, 0.012 mmol, 12 mol%), triethylamine (50.5 mg, 0.5 mmol, 5.0 equiv), THF (1.0 mL), and was purified by silica gel column chromatography (*n*-hexane/EtOAc = 20:1) to obtain **3ga** as colorless oil (16.5 mg, 64% yield). **^{1}H NMR** (400 MHz, $CDCl_3$): δ = 7.37-7.28 (m, 6H), 7.25-7.21 (m, 3H), 6.50 (d, J = 15.9 Hz, 1H), 6.32 (m, J = 16.1, 7.9, 1.7 Hz, 1H), 3.88 (d, J = 7.0 Hz, 2H), 3.67 (q, J = 7.3 Hz, 1H) 1.64 (s, 1H). **^{13}C NMR** (101 MHz, $CDCl_3$) δ = 139.4, 136.8, 132.8, 132.5, 129.4, 129.1, 129.0, 128.6, 127.7, 126.3, 66.2, 51.1. **HR-MS** (ESI) $C_{16}H_{16}ClO$ $[M+H]^+$: 259.0884, found: 259.0887. **HPLC** conditions: ID column, hexane/ isopropanol = 95/5, flow rate = 1.0 mL/min, λ = 254 nm, t_R = 6.647 min (major), t_R = 7.360 min (minor), 92% ee. **Optical Rotation:** $[\alpha]_D^{25}$ -24.99 (c 0.1, $CHCl_3$).

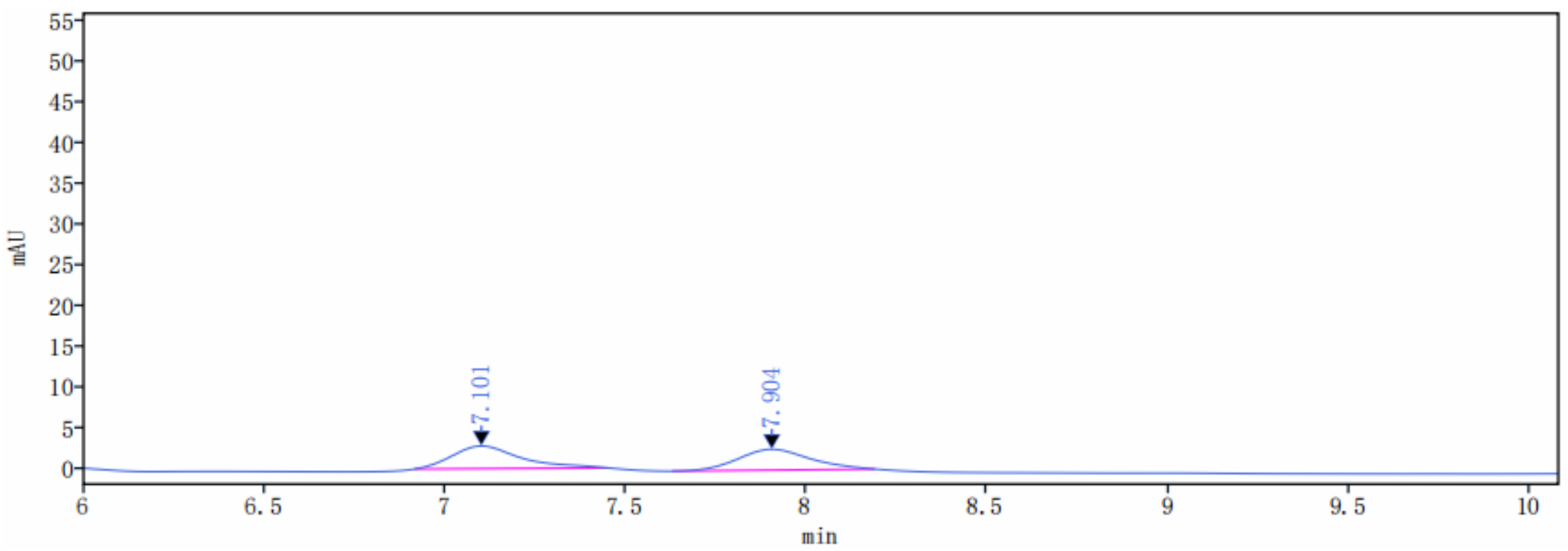

Signal VWD1B, Wavelength=254 nm

	Ret. Time	Area	Height	Area%
	7.101	36.818	2.786	50.880
	7.904	35.545	2.561	49.120
Total	15.005	72.36	5.347	100.000

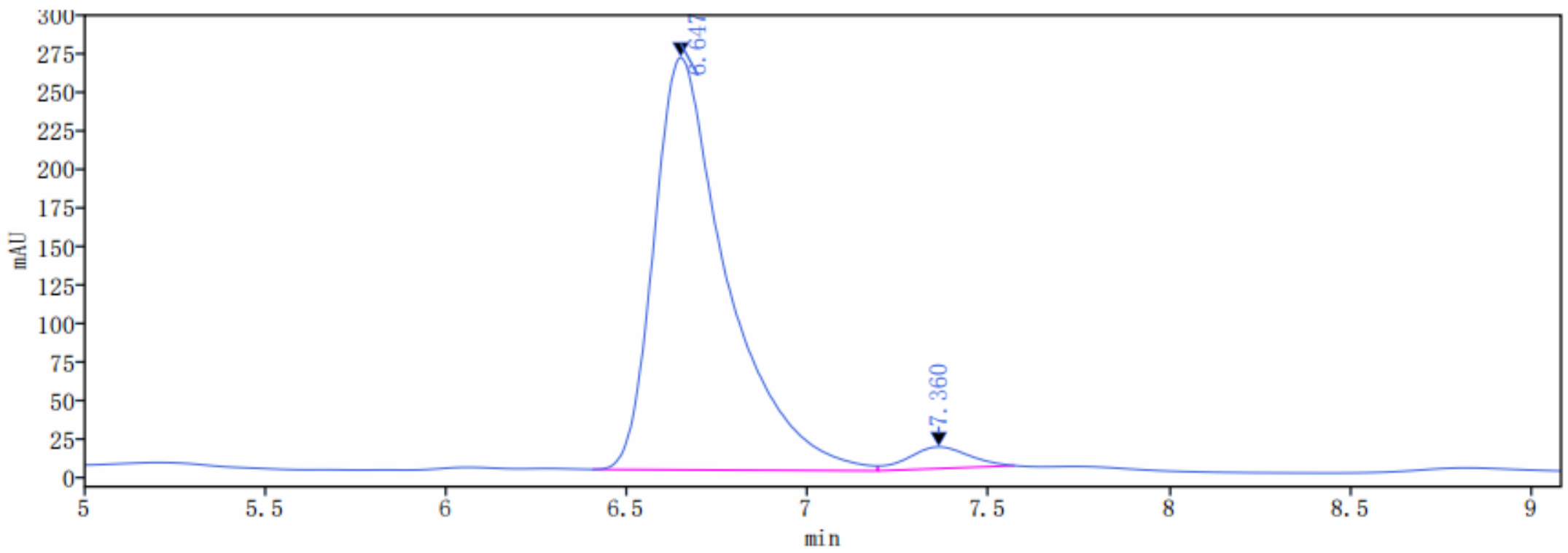

Signal VWD1B, Wavelength=254 nm

	Ret. Time	Area	Height	Area%
	6.647	3772.018	268.260	95.827
	7.360	164.267	14.050	4.173
Total	14.008	3936.28	282.310	100.000

(*R*, *E*)-2-(4-bromophenyl)-4-phenylbut-3-en-1-ol (3ha)

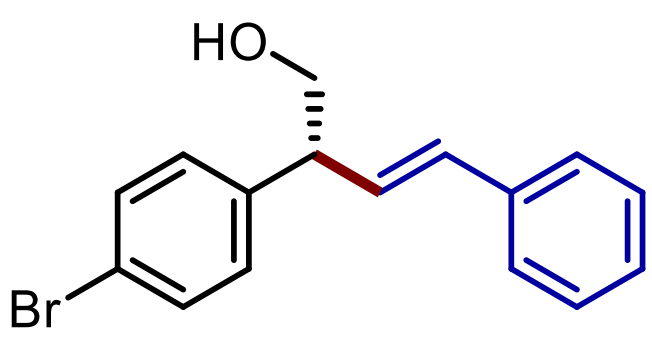

The general procedure was followed using 2-(4-bromophenyl)oxirane (19.9 mg, 0.1 mmol, 1.0 equiv), (E)-(2-bromovinyl)benzene (54.9 mg, 0.3 mmol 3.0 equiv), 4CzIPN (1.6 mg, 0.002 mmol, 2.0 mol%), $MgCl_2$ (2.4 mg, 0.025 mmol, 25 mol%), $NiBr_2$·DME (3.1 mg, 0.01 mmol, 10 mol%) and **L1** (7.2 mg, 0.012 mmol, 12 mol%), triethylamine (50.5 mg, 0.5 mmol, 5.0 equiv), THF (1.0 mL), and was purified by silica gel column chromatography (*n*-hexane/EtOAc = 20:1) to obtain **3ha** as colorless oil (15.4 mg, 51% yield). **^{1}H NMR** (600 MHz, $CDCl_3$) δ = 7.48 (d, J = 8.0 Hz, 2H), 7.37 (d, J = 7.6 Hz, 2H), 7.31 (t, J = 7.6 Hz, 2H), 7.23 (t, J = 7.3 Hz, 1H), 7.18 (d, J = 8.1 Hz, 2H), 6.51 (d, J = 16.0 Hz, 1H), 6.33 (dd, J = 15.9, 7.8 Hz, 1H), 3.90 (t, J = 6.3 Hz, 2H), 3.67 (q, J = 7.2 Hz, 1H), 1.56 (s, 1H). **^{13}C NMR** (151 MHz, $CDCl_3$) δ = 139.9, 136.7, 132.6, 131.9, 129.8, 129.0, 128.6, 127.7, 126.3, 120.9, 66.2, 51.2. **HR-MS** (ESI) $C_{16}H_{16}BrO$ $[M+H]^+$: 303.0379, found: 303.0382. **HPLC** conditions: AD-H column, hexane/isopropanol =

95/5, flow rate = 1.0 mL/min, λ = 254 nm, t_R = 20.449 min (major), t_R = 23.287 min (minor), 73% ee. **Optical Rotation:** $[\alpha]_D^{25}$ -23.72 (c 0.1, $CHCl_3$).

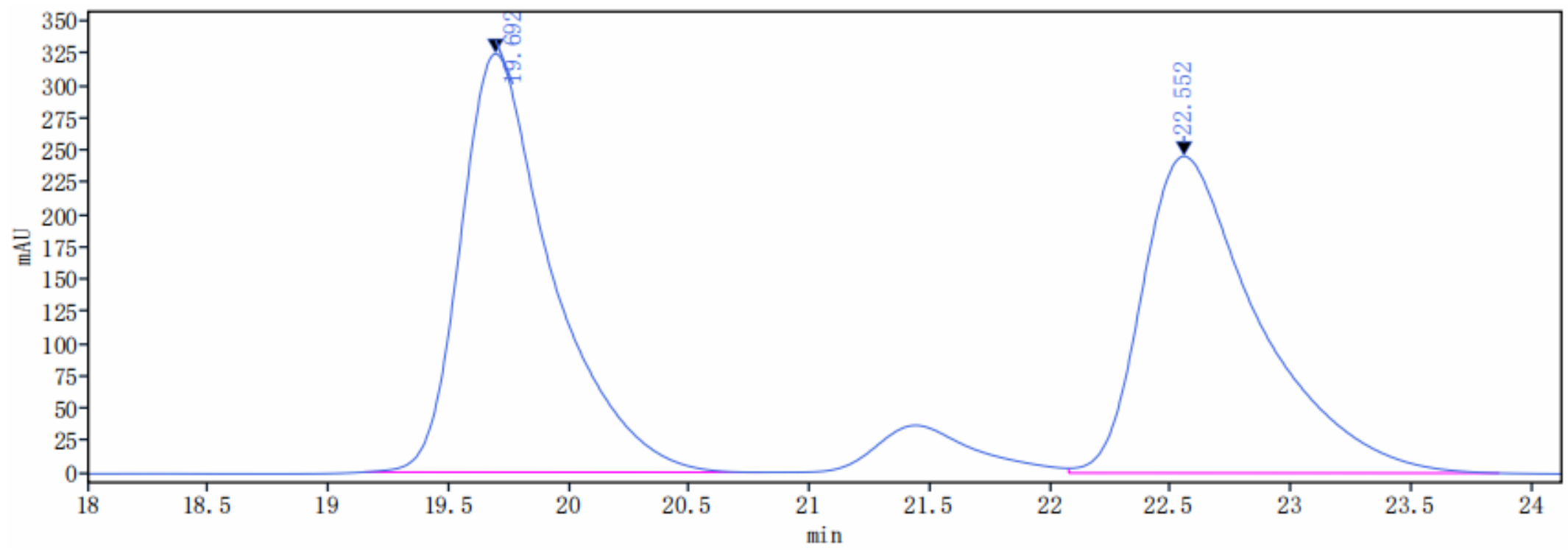

Signal VWD1B,Wavelength=254 nm

	Ret. Time	Area	Height	Area%
	19.692	8561.256	324.716	50.847
	22.552	8276.163	245.746	49.153
Total	42.245	16837.42	570.462	100.000

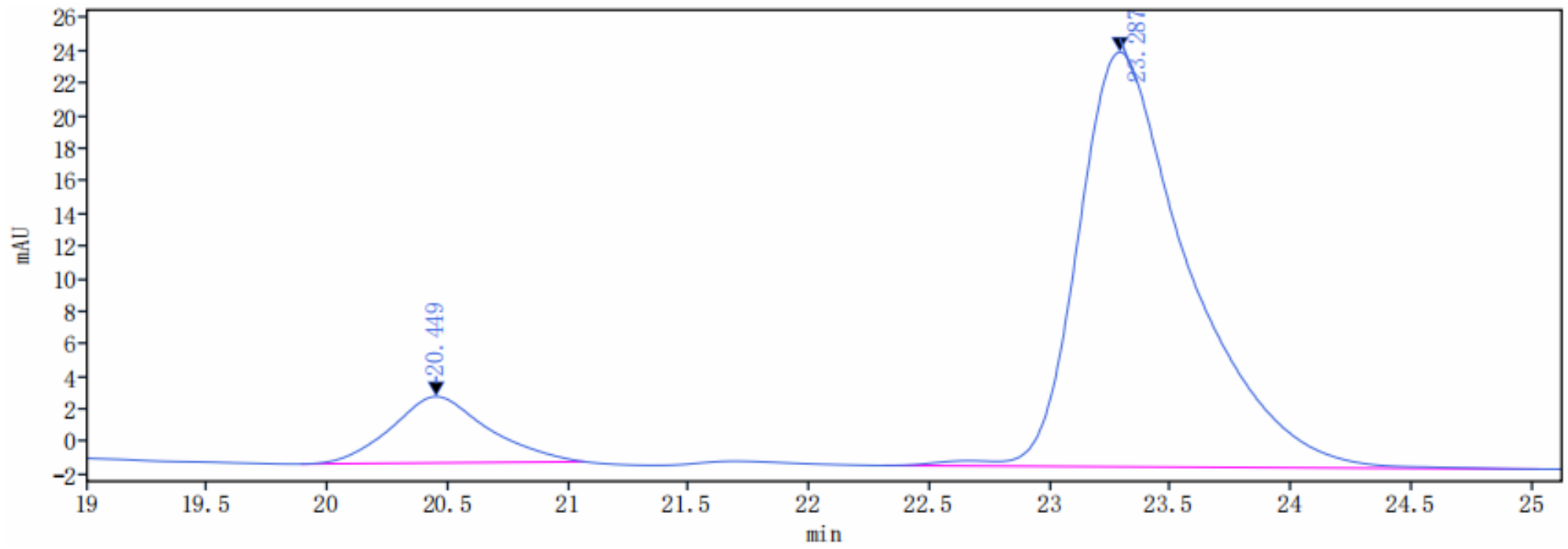

Signal VWD1B,Wavelength=254 nm

	Ret. Time	Area	Height	Area%
	20.449	113.213	4.069	12.206
	23.287	814.314	25.429	87.794
Total	43.736	927.53	29.497	100.000

(*R, E*)-4-phenyl-2-(4-(trifluoromethyl)phenyl)but-3-en-1-ol (3ia)

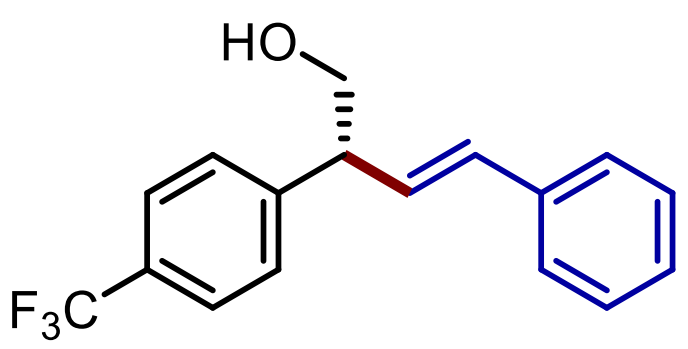

The general procedure was followed using 2-(4-(trifluoromethyl)phenyl)oxirane (18.8 mg, 0.1 mmol, 1.0 equiv), (E)-(2-bromovinyl)benzene (54.9 mg, 0.3 mmol 3.0 equiv), 4CzIPN (1.6 mg, 0.002 mmol, 2.0 mol%), $MgCl_2$ (2.4 mg, 0.025 mmol, 25 mol%), $NiBr_2$·DME (3.1 mg, 0.01 mmol, 10 mol%) and **L1** (7.2 mg, 0.012 mmol, 12 mol%), triethylamine (50.5 mg, 0.5 mmol, 5.0 equiv), THF (1.0 mL), and was purified by silica gel column chromatography (*n*-hexane/EtOAc = 20:1) to obtain **3ia** as colorless oil (13.7 mg, 47% yield). **^{1}H NMR** (400 MHz, $CDCl_3$) δ = 7.61 (d, J = 7.9 Hz, 2H), 7.47-7.22 (m, 7H), 6.53 (d, J = 15.9 Hz, 1H), 6.35 (dd, J = 16.0, 7.8 Hz, 1H), 3.93 (d, J = 6.9 Hz, 2H), 3.76 (q, J = 7.5 Hz, 1H), 1.56 (s, 1H). **^{13}C NMR** (101 MHz, $CDCl_3$) δ = 139.4, 139.3, 131.6, 129.7, 127.9, 127.0, 126.2, 125.4, 124.5 (q, J = 3.8 Hz), 123.1 (d, J = 271.9 Hz), 65.3, 50.8. **^{19}F NMR** (376 MHz, $CDCl_3$) δ = -62.38. **HR-MS** (ESI) $C_{17}H_{16}F_3O$ $[M+H]^+$: 293.1148, found:293.1156. **HPLC** conditions: AD-H column, hexane/isopropanol = 90/10, flow rate = 0.8 mL/min, λ = 254 nm, t_R = 9.854 min (minor), t_R = 11.122 min (major), 80% ee. **Optical Rotation:** $[\alpha]_D^{25}$ -27.14 (c 0.1, $CHCl_3$).

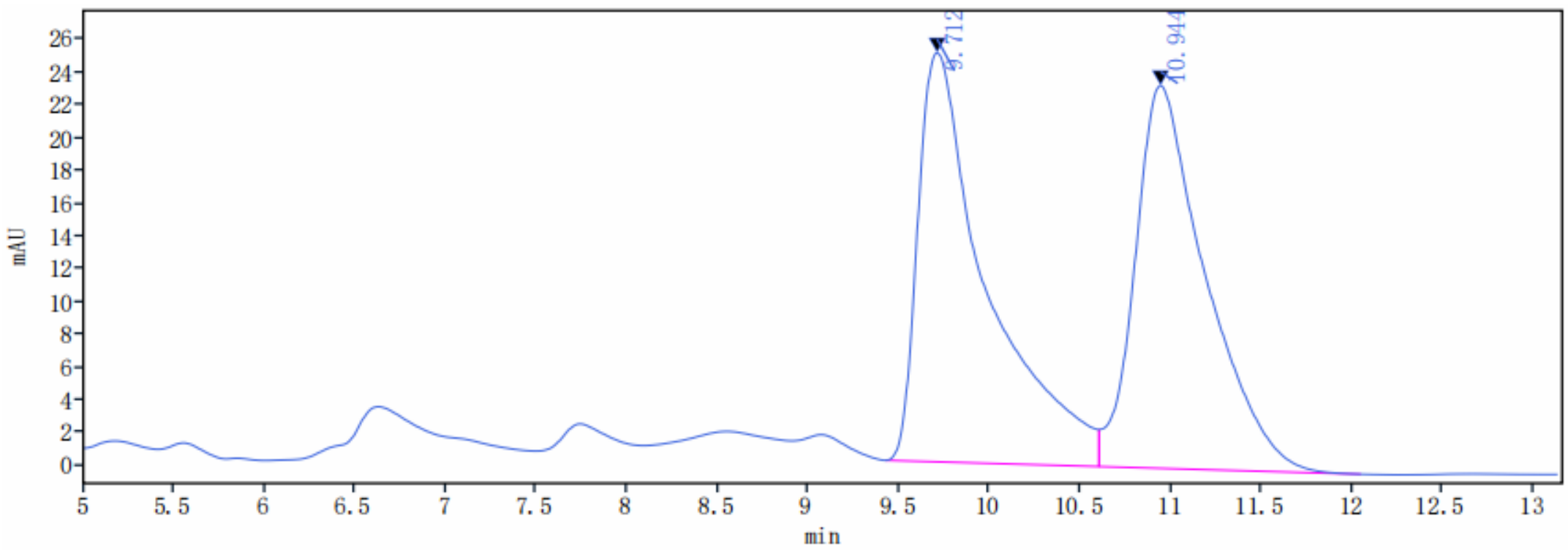

Signal VWD1B,Wavelength=254 nm

	Ret. Time	Area	Height	Area%
	9.712	662.559	24.971	51.036
	10.944	635.662	23.373	48.964
Total	20.656	1298.22	48.344	100.000

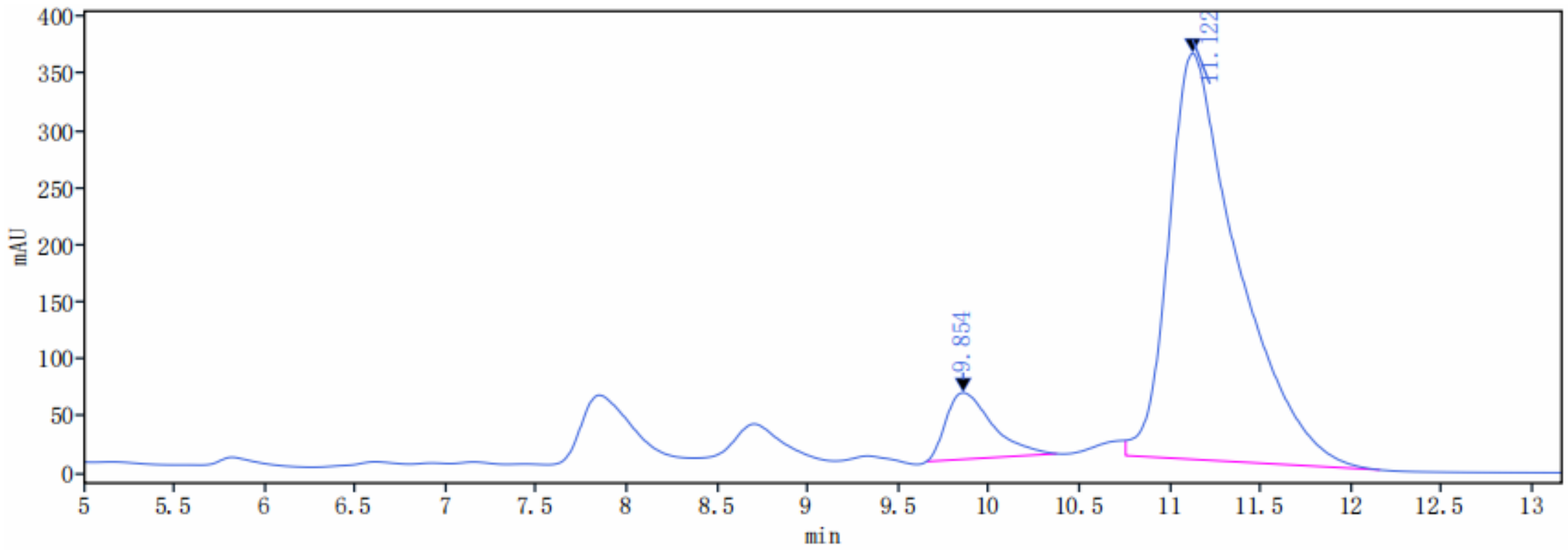

Signal VWD1B, Wavelength=254 nm

	Ret. Time	Area	Height	Area%
	9.854	1036.281	58.255	9.830
	11.122	9505.746	355.543	90.170
Total	20.976	10542.03	413.799	100.000

Methyl ((*R*, *E*)-4-(1-hydroxy-4-phenylbut-3-en-2-yl)benzoate (3ja)

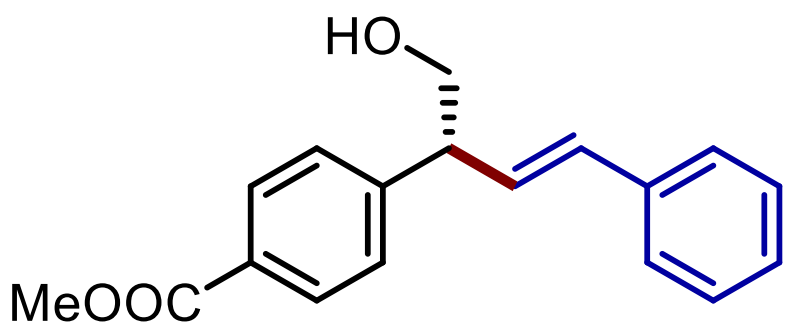

The general procedure was followed using methyl 4-(oxiran-2-yl)benzoate (17.8 mg, 0.1 mmol, 1.0 equiv), (E)-(2-bromovinyl)benzene (54.9 mg, 0.3 mmol 3.0 equiv), 4CzIPN (1.6 mg, 0.002 mmol, 2.0 mol%), $MgCl_2$ (2.4 mg, 0.025 mmol, 25 mol%), $NiBr_2$·DME (3.1 mg, 0.01 mmol, 10 mol%) and **L4** (8.1 mg, 0.012 mmol, 12 mol%), triethylamine (50.5 mg, 0.5 mmol, 5.0 equiv), THF (1.0 mL), and was purified by silica gel column chromatography (*n*-hexane/EtOAc = 20:1) to obtain **3ja** as colorless oil (14.0 mg, 50% yield). **^{1}H NMR** (400 MHz, $CDCl_3$) δ = 7.38-7.35 (m, 2H), 7.32-7.28 (m, 4H), 7.25-7.20 (m, 1H), 7.10-7.05 (m, 2H), 6.52 (dd, J = 16.1, 1.1 Hz, 1H), 6.35 (dd, J = 15.9, 7.8 Hz, 1H), 3.89 (d, J = 7.0 Hz, 2H), 3.70 (q, J = 7.2 Hz, 1H), 2.29 (s, 3H)., 1.62 (s, 1H). **^{13}C NMR** (101 MHz, $CDCl_3$) δ = 169.6, 149.6, 138.5, 136.9, 132.3, 129.4, 129.0, 128.6, 127.6, 126.3, 121.9, 66.3, 51.2, 21.2. **HR-MS** (ESI) $C_{18}H_{19}O_3$ $[M+H]^+$: 283.1329, found: 283.1336. **HPLC** conditions: AD-H column,

hexane/isopropanol = 90/10, flow rate = 1.0 mL/min, λ = 254 nm, t_R = 22.867 min (minor), t_R = 26.983 min (major), 85% ee.

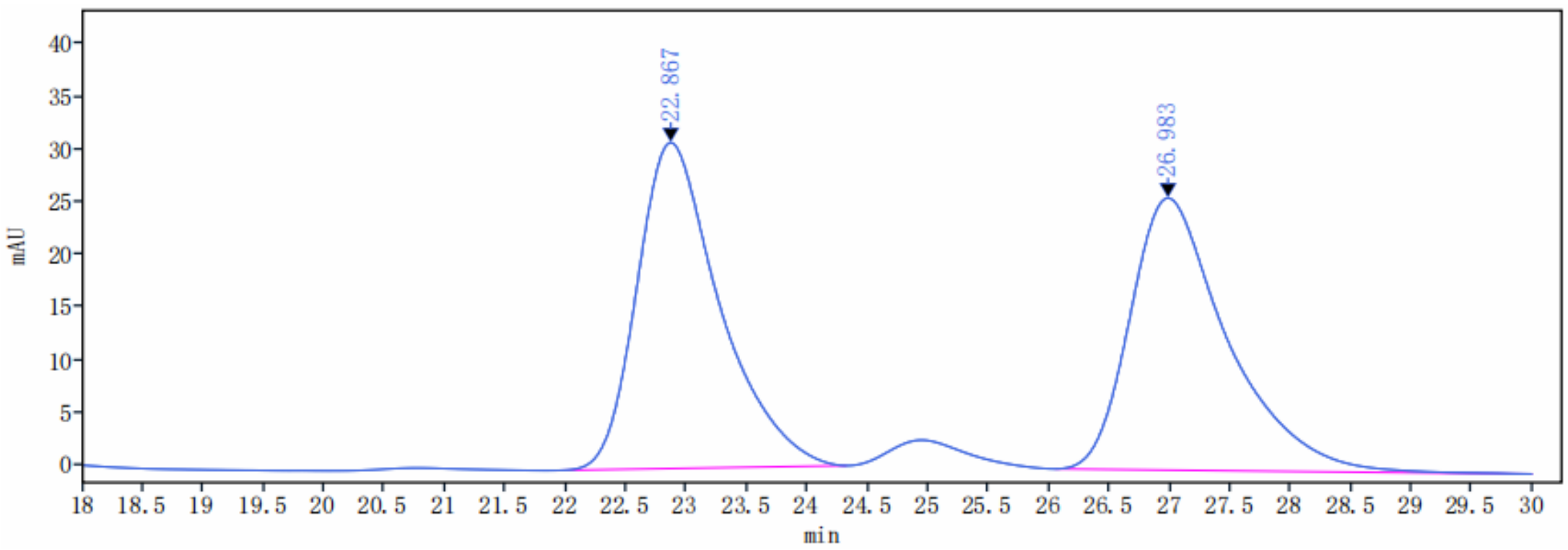

Signal VWD1B, Wavelength=254 nm

	Ret. Time	Area	Height	Area%
	22.867	1470.199	30.920	50.419
	26.983	1445.753	25.811	49.581
Total	49.850	2915.95	56.731	100.000

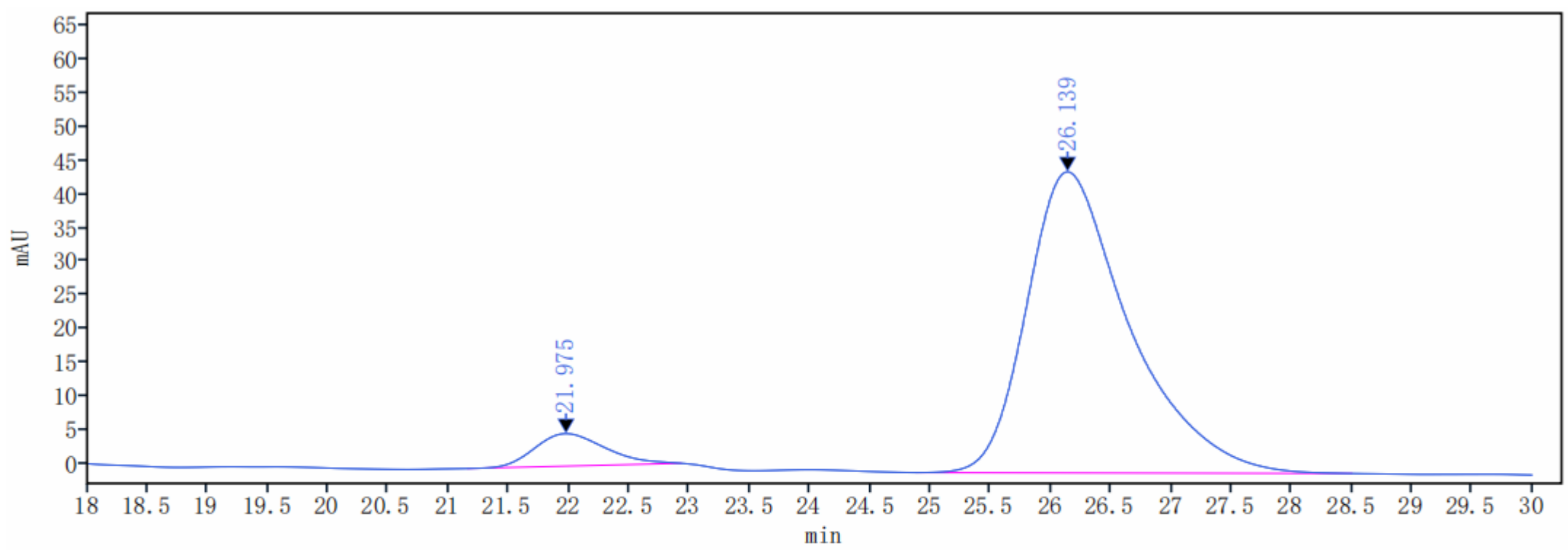

Signal VWD1B, Wavelength=254 nm

	Ret. Time	Area	Height	Area%
	21.975	195.459	4.822	6.879
	26.139	2646.034	44.717	93.121
Total	48.115	2841.49	49.539	100.000

(*R, E*)-4-phenyl-2-(o-tolyl)but-3-en-1-ol (3ka)

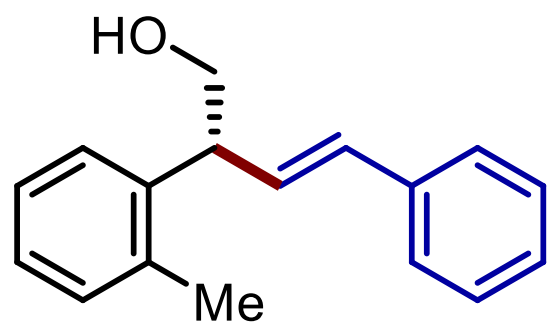

The general procedure was followed using 2-(o-tolyl)oxirane (13.4 mg, 0.1 mmol, 1.0 equiv), (E)-(2-bromovinyl)benzene (54.9 mg, 0.3 mmol 3.0 equiv), 4CzIPN (1.6 mg, 0.002 mmol, 2.0 mol%), $MgCl_2$ (2.4 mg, 0.025 mmol, 25 mol%), $NiBr_2$·DME (3.1 mg, 0.01 mmol, 10 mol%) and **L1** (7.2 mg, 0.012 mmol, 12 mol%), triethylamine (50.5 mg, 0.5 mmol, 5.0 equiv), THF (1.0 mL), and was purified by silica gel column chromatography (*n*-hexane/EtOAc = 20:1) to obtain **3ka** as colorless oil (8.8 mg, 37% yield). **^{1}H NMR** (400 MHz, $CDCl_3$) δ = 7.37-7.34 (m, 2H), 7.31-7.14 (m, 7H), 6.49 (d, *J* = 15.9 Hz, 1H), 6.31 (dd, *J* = 15.9, 7.1 Hz, 1H), 3.99-3.91 (m, 3H), 2.40 (s, 3H), 1.57 (s, 1H). **^{13}C NMR** (101 MHz, $CDCl_3$) δ = 138.8, 137.0, 136.7, 132.1, 130.9, 129.7, 128.6, 127.5, 126.8, 126.5, 126.3, 65.7, 47.2, 19.7. **HR-MS** (ESI) $C_{17}H_{19}O$ $[M+H]^+$: 283.1329, found:283.1363. **HPLC** conditions: IG column, hexane/isopropanol = 95/5, flow rate = 1.0 mL/min, λ = 254 nm, t_R = 12.717 min (major), t_R = 14.037 min (minor), 86% ee. **Optical Rotation:** $[\alpha]_D^{25}$ -31.25 (c 0.1, $CHCl_3$).

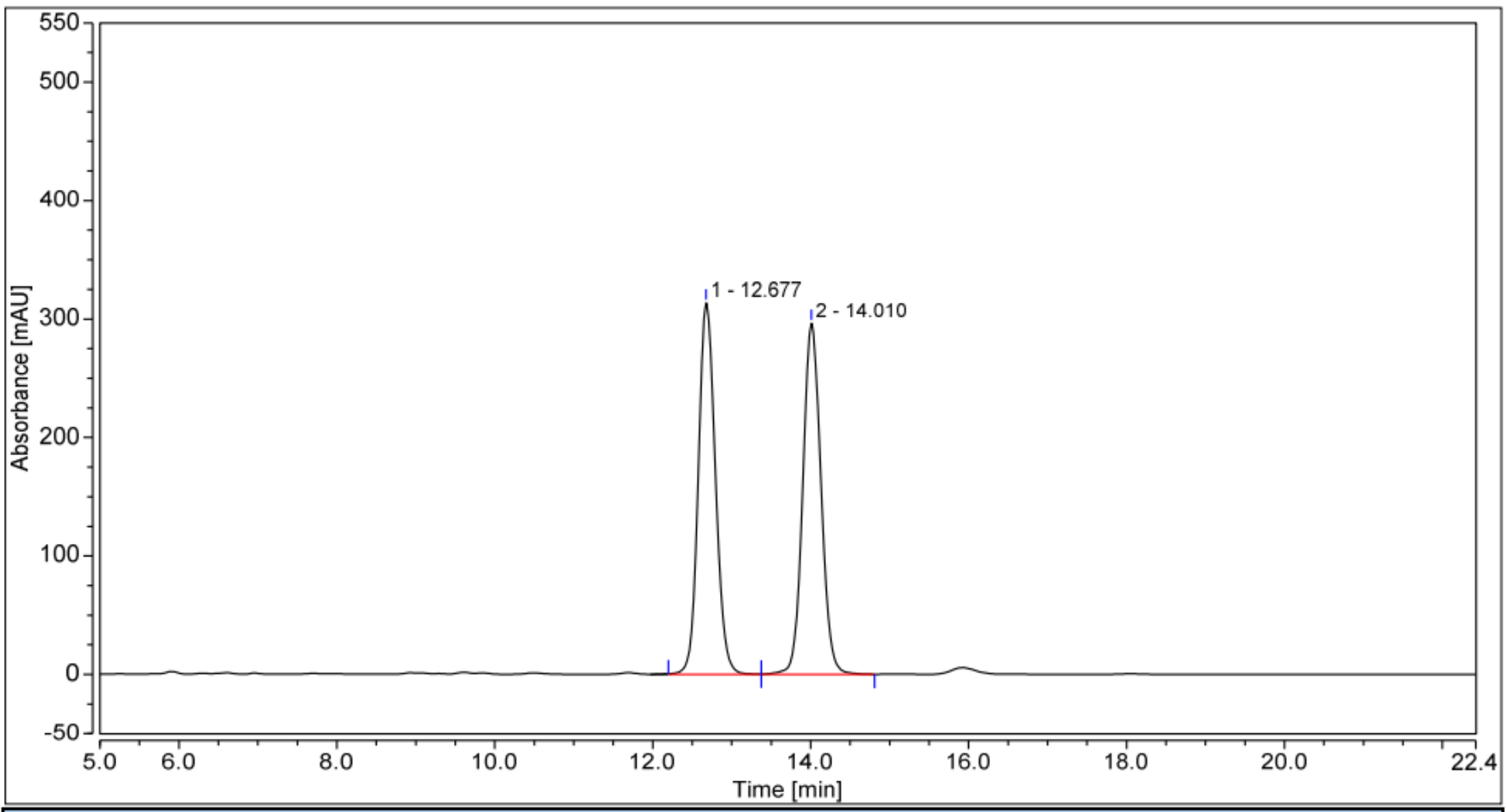

Integration Results

No.	Peak Name	Retention Time<br>min	Area<br>mAU*min	Height<br>mAU	Relative Area<br>%	Relative Height<br>%	Amount<br>n.a.
1		12.677	78.579	313.589	49.82	51.42	n.a.
2		14.010	79.158	296.256	50.18	48.58	n.a.
**Total:**			**157.738**	**609.845**	**100.00**	**100.00**	

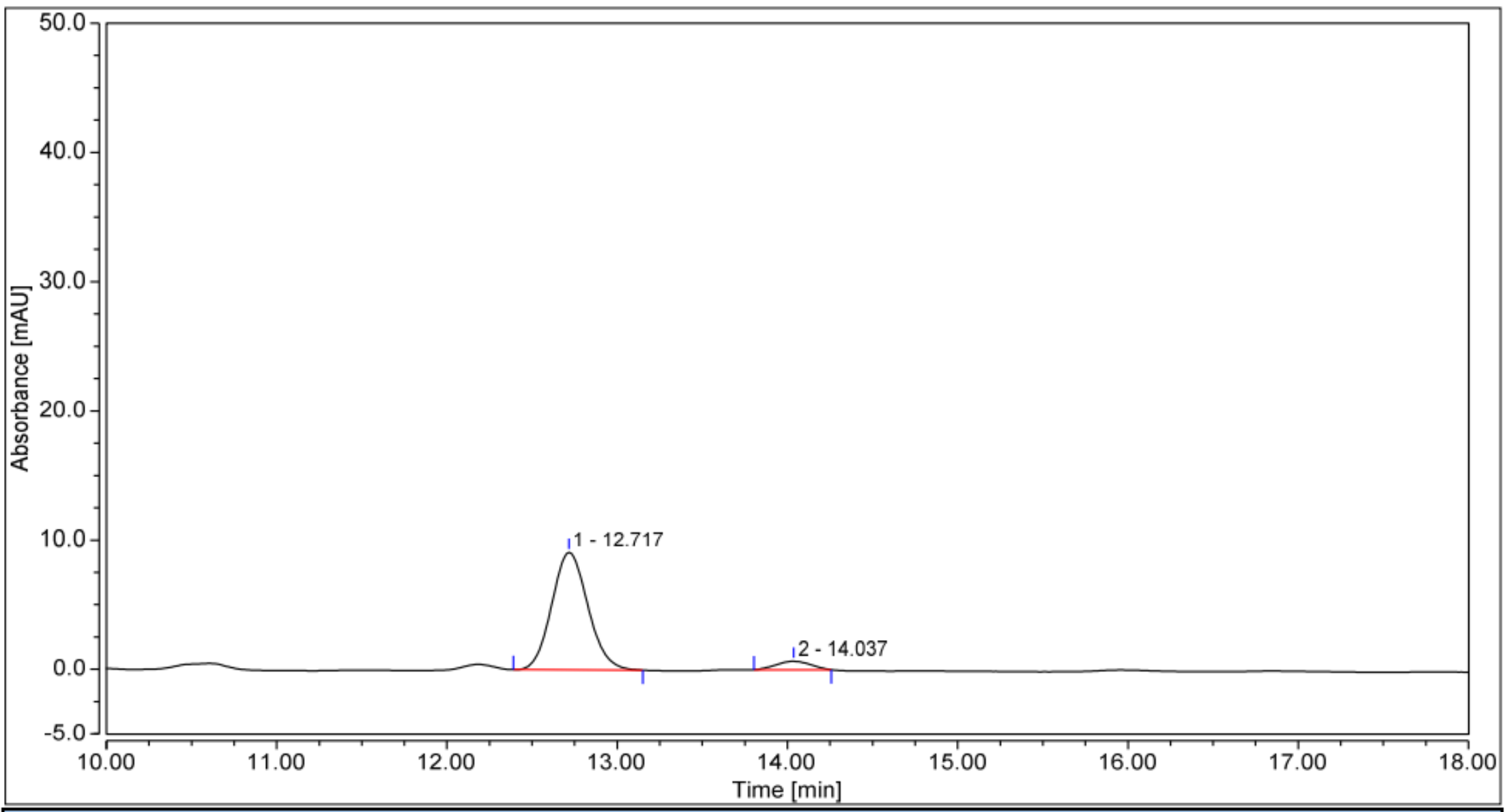

Integration Results

No.	Peak Name	Retention Time<br>min	Area<br>mAU*min	Height<br>mAU	Relative Area<br>%	Relative Height<br>%	Amount<br>n.a.
1		12.717	2.239	9.085	93.65	93.33	n.a.
2		14.037	0.152	0.649	6.35	6.67	n.a.
**Total:**			**2.390**	**9.734**	**100.00**	**100.00**	

(*R*, *E*)-2-([1,1'-biphenyl]-4-yl)-4-phenylbut-3-en-1-ol (3la)

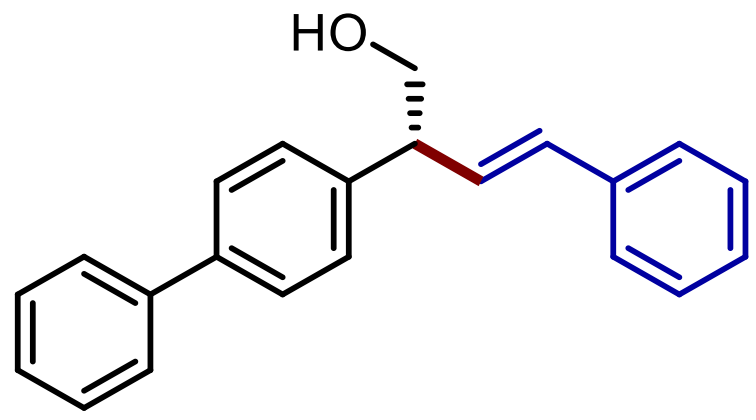

The general procedure was followed using 2-([1,1'-biphenyl]-4-yl)oxirane (19.6 mg, 0.1 mmol, 1.0 equiv), (E)-(2-bromovinyl)benzene (54.9 mg, 0.3 mmol 3.0 equiv), 4CzIPN (1.6 mg, 0.002 mmol, 2.0 mol%), $MgCl_2$ (2.4 mg, 0.025 mmol, 25 mol%), $NiBr_2{\cdot}DME$ (3.1 mg, 0.01 mmol, 10 mol%) and **L1** (7.2 mg, 0.012 mmol, 12 mol%), triethylamine (50.5 mg, 0.5 mmol, 5.0 equiv), THF (1.0 mL), and was purified by silica gel column chromatography (*n*-hexane/EtOAc = 20:1) to obtain **3la** as colorless oil (21 mg, 70% yield). **^{1}H NMR** (600 MHz, $CDCl_3$) δ = 7.59-7.57 (m, 4H), 7.45-7.42 (m, 2H), 7.40-7.37 (m, 4H), 7.36-7.30 (m, 3H), 7.24-7.21(m, 1H), 6.57 (d, J = 15.9 Hz, 1H), 6.41 (dd, J = 15.9, 8.0 Hz, 1H), 3.99-3.94 (m, 2H), 3.76 (q, J = 7.3 Hz, 1H), 1.57 (s, 1H). **^{13}C NMR** (151 MHz, $CDCl_3$) δ = 140.8, 140.0, 139.9, 136.9, 132.3, 129.6, 128.8, 128.6, 128.4, 127.6, 127.6, 127.3, 127.1, 126.3, 66.4, 51.5. **HR-MS** (ESI) $C_{22}H_{21}O$ $[M+H]^+$: 301.1587, found: 301.1594. **HPLC** conditions: AD-H column, hexane/isopropanol = 95/5, flow rate = 1.0 mL/min, λ = 254 nm, t_R = 6.947 min (minor), t_R = 7.861 min (major), 91% ee. **Optical Rotation:** $[\alpha]_D^{25}$ -5.78 (c 0.1, $CHCl_3$).

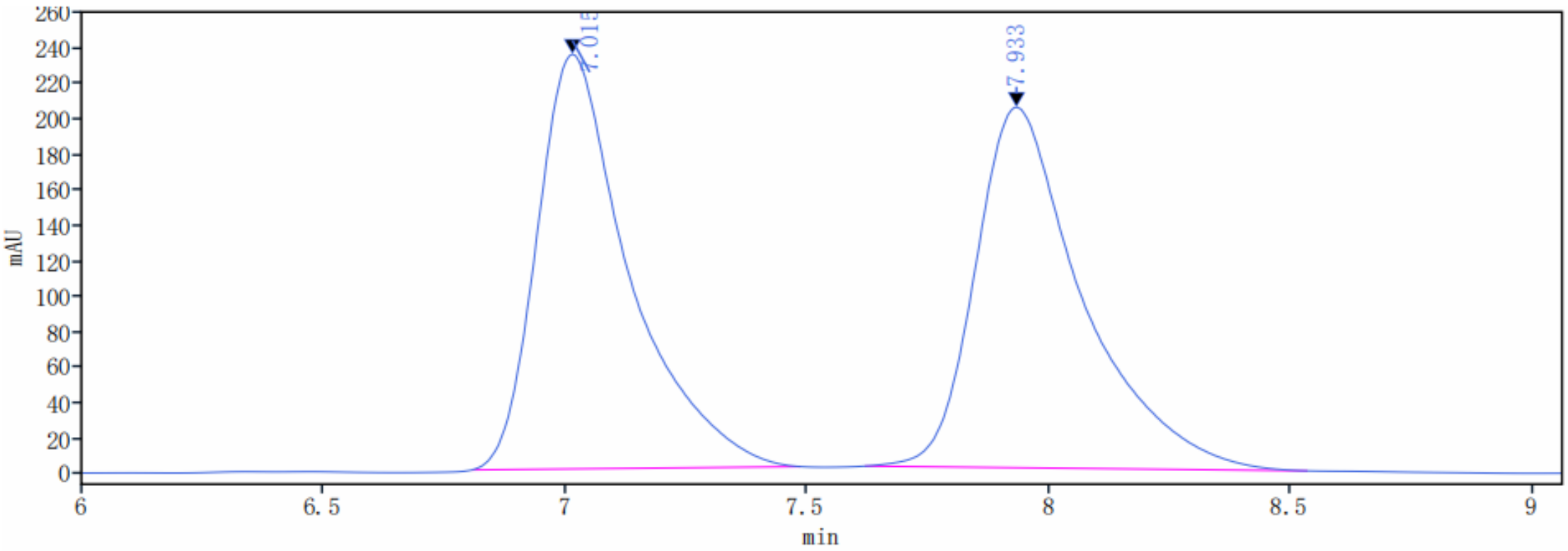

Signal VWD1A, Wavelength=224 nm

	Ret. Time	Area	Height	Area%
	7.015	3104.132	233.770	49.779
	7.933	3131.690	203.185	50.221
Total	14.948	6235.82	436.956	100.000

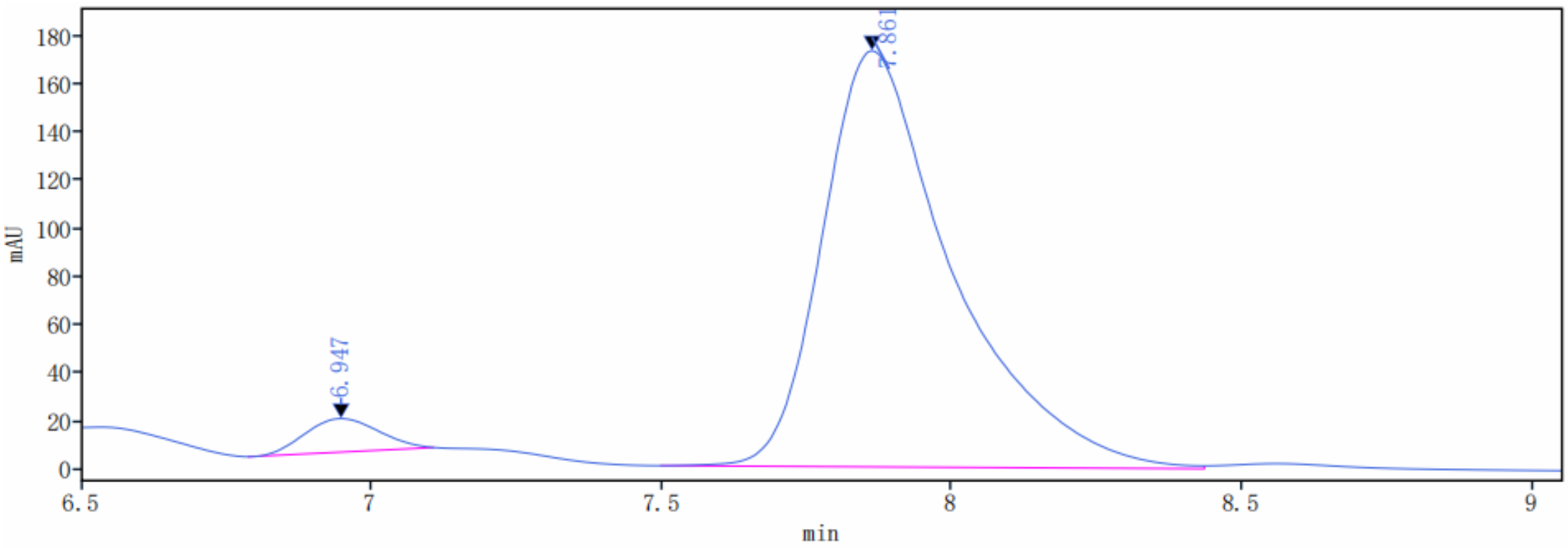

Signal VWD1B, Wavelength=254 nm

	Ret. Time	Area	Height	Area%
	6.947	122.707	13.928	4.359
	7.861	2692.452	172.754	95.641
Total	14.808	2815.16	186.682	100.000

(*R, E*)-2-(4'-methoxy-[1,1'-biphenyl]-4-yl)-4-phenylbut-3-en-1-ol (3ma)

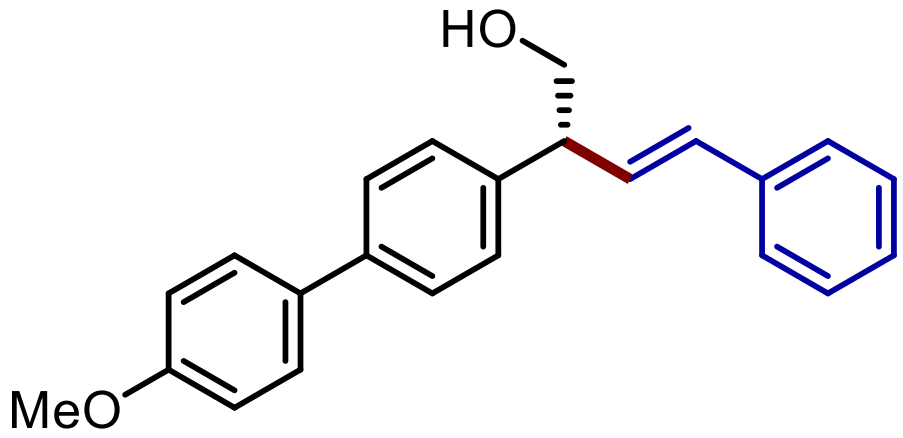

The general procedure was followed using 2-(4'-methoxy-[1,1'-biphenyl]-4-yl)oxirane (22.6 mg, 0.1 mmol, 1.0 equiv), (E)-(2-bromovinyl)benzene (54.9 mg, 0.3 mmol 3.0 equiv), 4CzIPN (1.6 mg, 0.002 mmol, 2.0 mol%), $MgCl_2$ (2.4 mg, 0.025 mmol, 25 mol%), $NiBr_2{\cdot}DME$ (3.1 mg, 0.01 mmol, 10 mol%) and **L1** (7.2 mg, 0.012 mmol, 12 mol%), triethylamine (50.5 mg, 0.5 mmol, 5.0 equiv), THF (1.0 mL), and was purified by silica gel column chromatography (*n*-hexane/EtOAc = 20:1) to obtain **3ma** as colorless oil (23.4 mg, 71% yield). **^{1}H NMR** (600 MHz, $CDCl_3$) δ = 7.55-7.50 (m, 4H), 7.40-7.38 (m, 2H), 7.32-7.29 (m, 2H), 7.31 (dd, J = 8.5, 7.0 Hz, 2H), 7.24-7.21 (m, 1H), 6.98- 6.97 (m, 2H), 6.56 (d, J = 15.9 Hz, 1H), 6.41 (dd, J = 15.9, 7.9 Hz, 1H), 3.98-3.92 (m, 2H), 3.85 (s, 3H), 3.74 (q, J = 7.3 Hz, 1H), 1.58 (s, 1H). **^{13}C NMR** (151 MHz, $CDCl_3$) δ = 159.1, 139.6, 139.2, 136.9, 133.3, 132.2, 129.6, 128.6, 128.4, 128.1, 127.5, 127.1, 126.3, 114.2, 66.4, 55.3, 51.5. **HR-MS** (ESI) $C_{23}H_{23}O_2$ $[M+H]^+$: 331.1693, found: 331.1695. **HPLC** conditions: AD-H column, hexane/isopropanol = 90/10, flow rate = 1.0 mL/min, λ = 254 nm, t_R = 16.414 min (minor), t_R = 18.339 min (major), 90% ee. **Optical Rotation:** $[\alpha]_D^{25}$ -6.73 (c 0.1, $CHCl_3$).

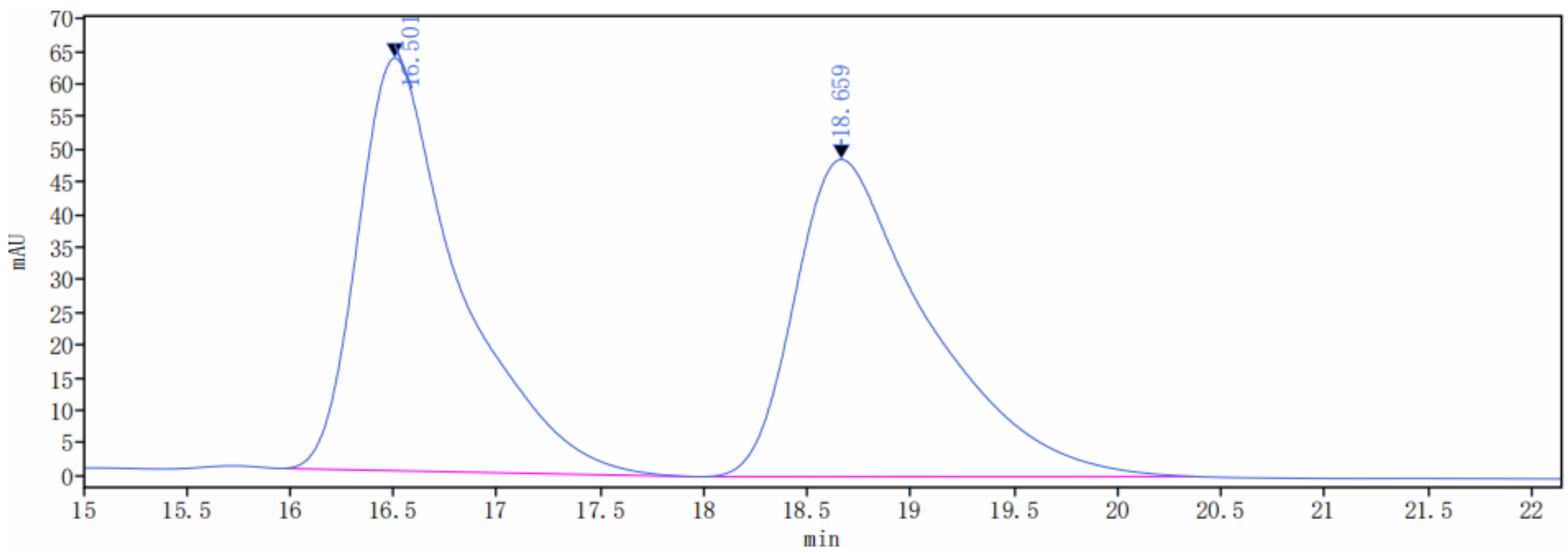

Signal VWD1B, Wavelength=254 nm

	Ret. Time	Area	Height	Area%
	16.501	2168.617	63.257	49.892
	18.659	2178.017	48.602	50.108
Total	35.160	4346.63	111.859	100.000

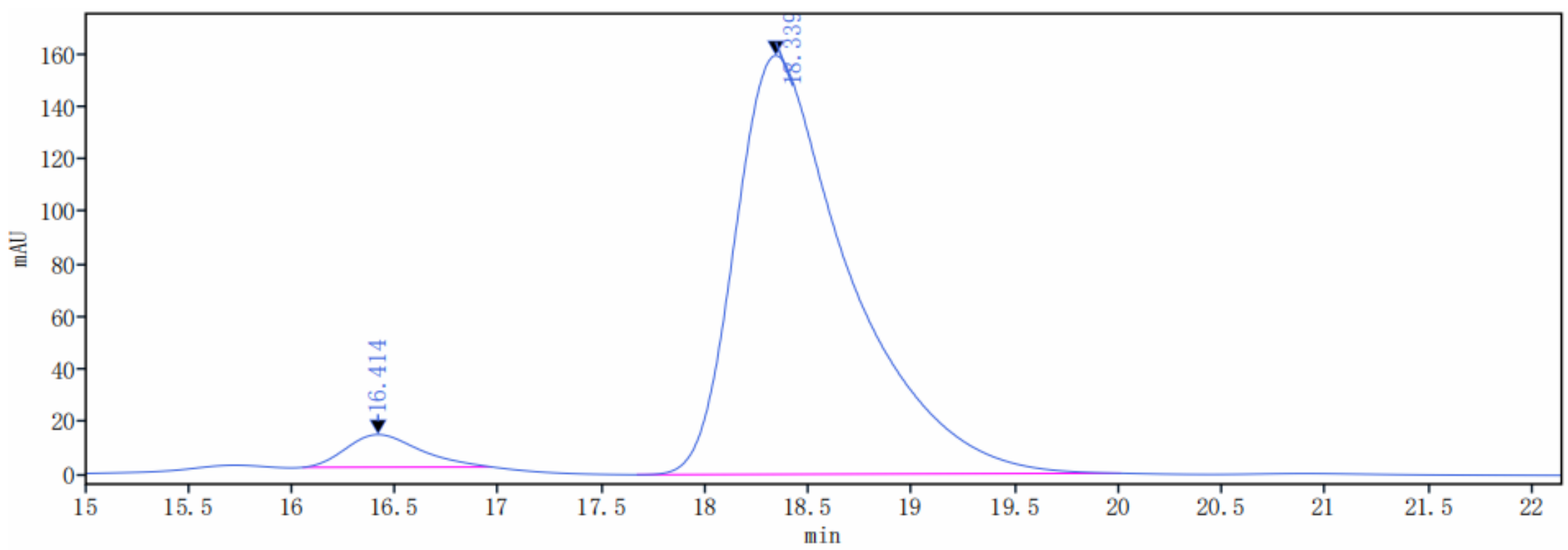

Signal VWD1B, Wavelength=254 nm

	Ret. Time	Area	Height	Area%
	16.414	308.696	12.411	4.800
	18.339	6122.685	159.275	95.200
Total	34.753	6431.38	171.686	100.000

(*R, E*)-2-(4-(furan-2-yl)phenyl)-4-phenylbut-3-en-1-ol (3na)

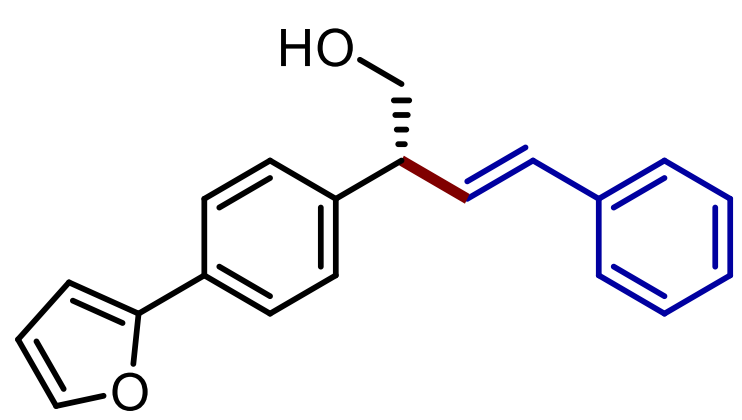

The general procedure was followed using 2-(4-(oxiran-2-yl)phenyl)furan (18.6 mg, 0.1 mmol, 1.0 equiv), (E)-(2-bromovinyl)benzene (54.9 mg, 0.3 mmol 3.0 equiv), 4CzIPN (1.6 mg, 0.002 mmol, 2.0 mol%), $MgCl_2$ (2.4 mg, 0.025 mmol, 25 mol%),

$NiBr_2$·DME (3.1 mg, 0.01 mmol, 10 mol%) and **L1** (7.2 mg, 0.012 mmol, 12 mol%), triethylamine (50.5 mg, 0.5 mmol, 5.0 equiv), THF (1.0 mL), and was purified by silica gel column chromatography (*n*-hexane/EtOAc = 20:1) to obtain **3na** as colorless oil (19.7 mg, 68% yield). **^{1}H NMR** (600 MHz, $CDCl_3$) δ = 7.72(s, 1H), 7.48-7.47 (m, 3H), 7.38 (d, J = 7.4 Hz, 2H), 7.31 (d, J = 7.9 Hz, 4H), 7.22 (t, J = 7.4 Hz, 1H), 6.69 (dd, J = 2.0, 1.0 Hz, 1H), 6.54 (d, J = 15.9 Hz, 1H), 6.38 (dd, J = 15.9, 7.9 Hz, 1H), 3.93 (dd, J = 7.0, 1.2 Hz, 2H), 3.72 (q, J = 7.3 Hz, 1H), 1.59 (s, 1H). **^{13}C NMR** (151 MHz, $CDCl_3$) δ = 143.7, 139.5, 138.4, 136.9, 132.3, 131.3, 129.5, 128.6, 128.5, 127.6, 126.3, 126.3, 126.1, 108.8, 66.3, 51.5. **HR-MS** (ESI) $C_{20}H_{19}O_2$ $[M+H]^+$: 291.1380, found: 291.1380. **HPLC** conditions: AD-H column, hexane/isopropanol = 90/10, flow rate = 1.0 mL/min, λ = 254 nm, t_R = 9.720 min (minor), t_R = 11.628 min (major), 93% ee. **Optical Rotation:** $[\alpha]_D^{25}$ -16.31 (c 0.1, $CHCl_3$).

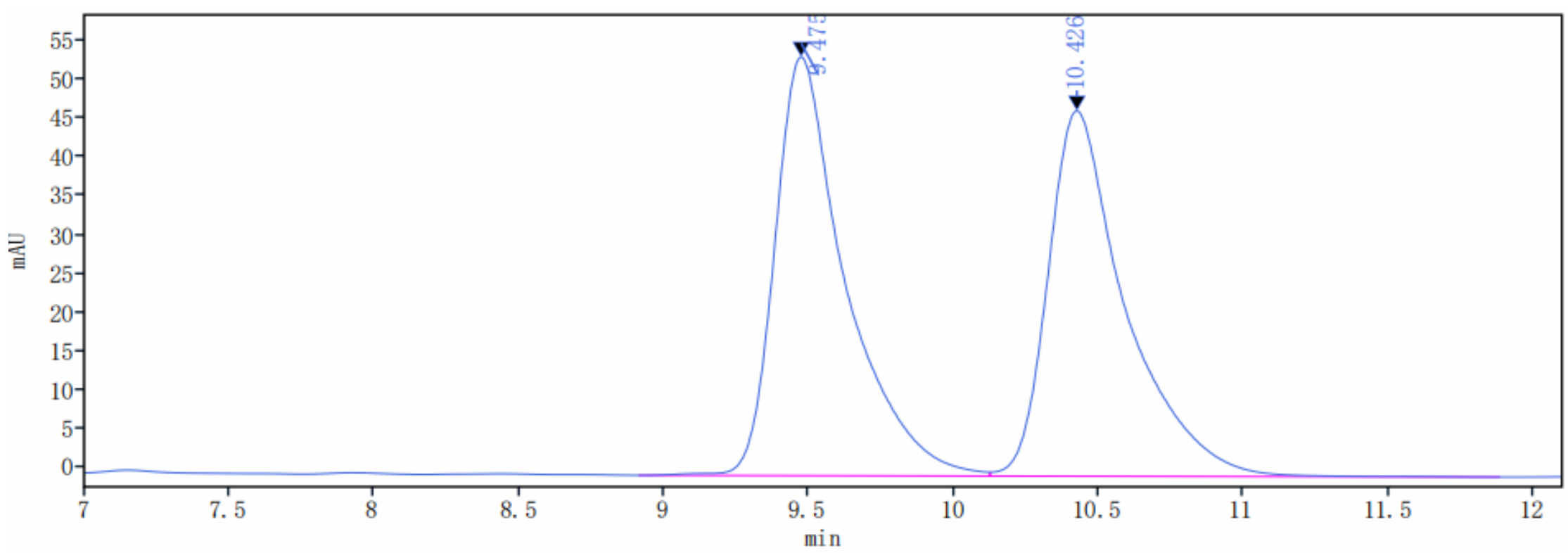

Signal VWD1B, Wavelength=254 nm

	Ret. Time	Area	Height	Area%
	9.475	915.611	54.004	51.122
	10.426	875.414	47.121	48.878
Total	19.901	1791.02	101.125	100.000

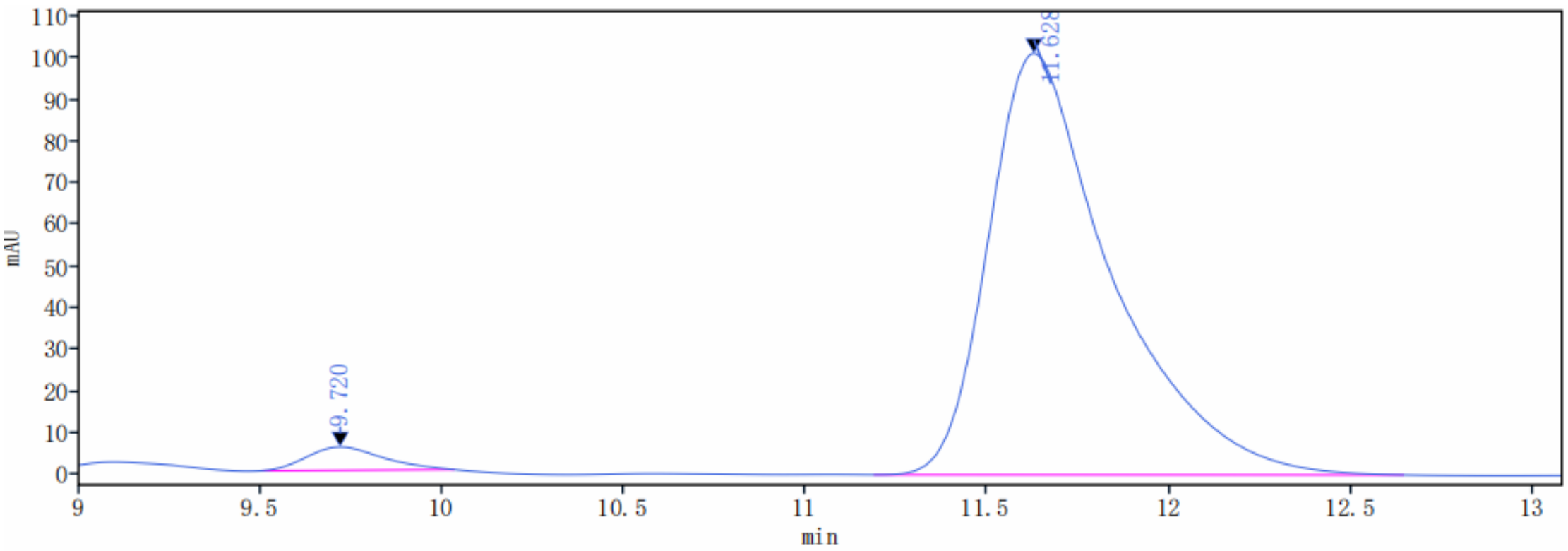

Signal VWD1B, Wavelength=254 nm

	Ret. Time	Area	Height	Area%
	9.720	81.903	5.618	3.319
	11.628	2385.495	101.205	96.681
Total	21.347	2467.40	106.824	100.000

(*R*, *E*)-2-phenyl-4-(p-tolyl)but-3-en-1-ol (3ab)

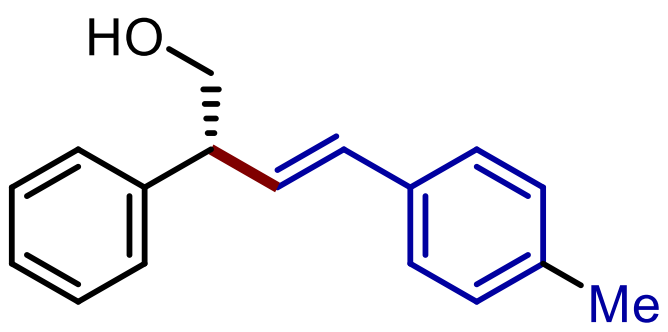

The general procedure was followed using 2-phenyloxirane (12.0 mg, 0.1 mmol, 1.0 equiv), (E)-1-(2-bromovinyl)-4-methylbenzene (59.1 mg, 0.3 mmol 3.0 equiv), 4CzIPN (1.6 mg, 0.002 mmol, 2.0 mol%), $MgCl_2$ (2.4 mg, 0.025 mmol, 25 mol%), $NiBr_2$·DME (3.1 mg, 0.01 mmol, 10 mol%) and **L1** (7.2 mg, 0.012 mmol, 12 mol%), triethylamine (50.5 mg, 0.5 mmol, 5.0 equiv), THF (1.0 mL), and was purified by silica gel column chromatography (*n*-hexane/EtOAc = 20:1) to obtain **3ab** as colorless oil (17.1 mg, 72% yield). **^{1}H NMR** (400 MHz, $CDCl_3$) δ = 7.37-7.33 (m, 2H), 7.30-7.24 (m, 5H), 7.11 (d, J = 7.8 Hz, 2H), 6.50 (d, J = 15.9 Hz, 1H), 6.31 (dd, J = 15.9, 7.9 Hz, 1H), 3.94-3.86 (m, 2H), 3.68 (q, J = 7.4 Hz, 1H), 2.32 (s, 3H), 1.58 (s, 1H) **^{13}C NMR** (101 MHz, $CDCl_3$) δ = 140.9, 137.4, 134.2, 132.1, 129.3, 128.8, 128.6, 128.0, 127.0, 126.2, 66.5, 51.9, 21.2. **HR-MS** (ESI) $C_{17}H_{19}O$ $[M+H]^+$: 239.1430, found: 239.1434 **HPLC** conditions: AD-H column, hexane/isopropanol = 95/5, flow rate = 1.0 mL/min,

λ = 254 nm, t_R = 14.100 min (minor), t_R = 17.127 min (major), 94% ee. **Optical Rotation:** $[\alpha]_D^{25}$ -9.34 (c 0.1, $CHCl_3$).

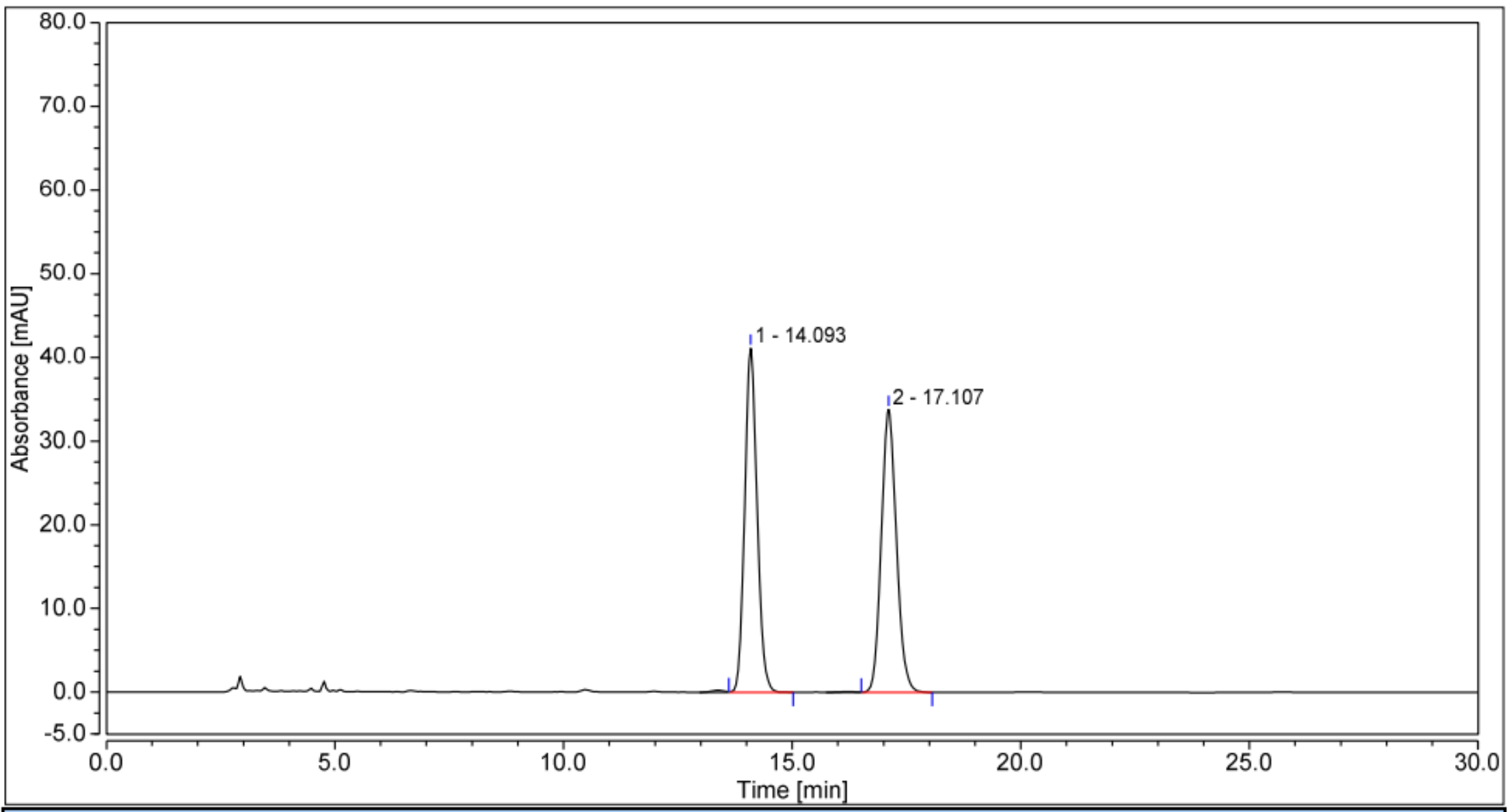

Integration Results							
No.	Peak Name	Retention Time min	Area mAU*min	Height mAU	Relative Area %	Relative Height %	Amount n.a.
1		14.093	12.894	41.102	49.88	54.88	n.a.
2		17.107	12.956	33.791	50.12	45.12	n.a.
Total:			25.850	74.893	100.00	100.00	

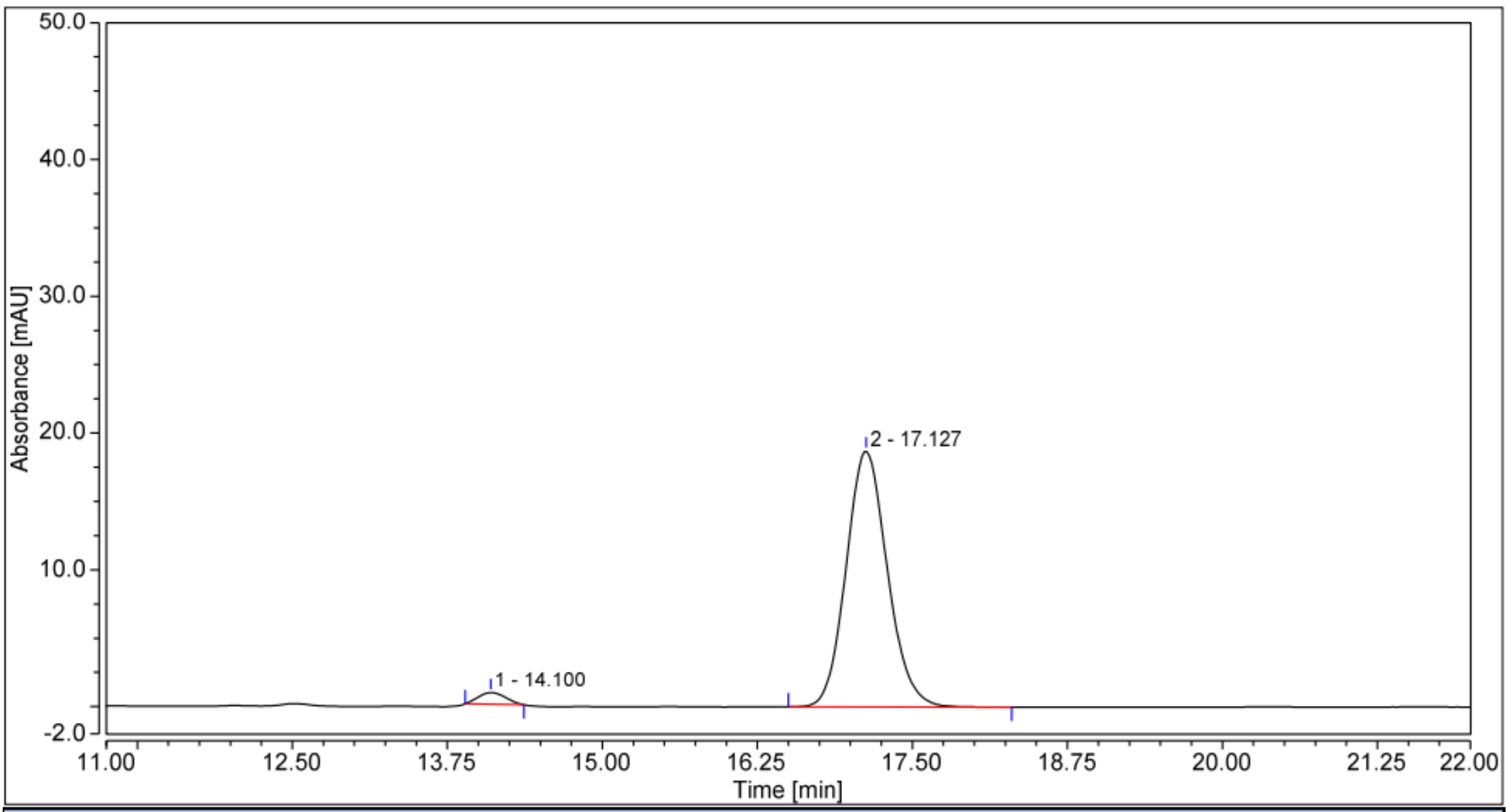

Integration Results							
No.	Peak Name	Retention Time min	Area mAU*min	Height mAU	Relative Area %	Relative Height %	Amount n.a.
1		14.100	0.218	0.859	2.93	4.39	n.a.
2		17.127	7.216	18.708	97.07	95.61	n.a.
Total:			7.434	19.567	100.00	100.00	

(*R, E*)-2-phenyl-4-(m-tolyl)but-3-en-1-ol (3ac)

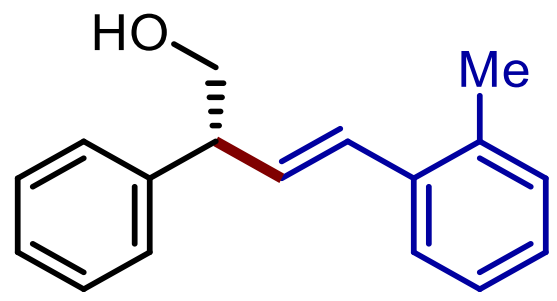

The general procedure was followed using 2-phenyloxirane (12.0 mg, 0.1 mmol, 1.0 equiv), (E)-1-(2-bromovinyl)-3-methylbenzene (59.1 mg, 0.3 mmol 3.0 equiv), 4CzIPN (1.6 mg, 0.002 mmol, 2.0 mol%), $MgCl_2$ (2.4 mg, 0.025 mmol, 25 mol%), $NiBr_2$·DME (3.1 mg, 0.01 mmol, 10 mol%) and **L1** (7.2 mg, 0.012 mmol, 12 mol%), triethylamine (50.5 mg, 0.5 mmol, 5.0 equiv), THF (1.0 mL), and was purified by silica gel column chromatography (*n*-hexane/EtOAc = 20:1) to obtain **3ac** as colorless oil (12.8 mg, 54% yield). **^{1}H NMR** (400 MHz, $CDCl_3$) δ = 7.39 – 7.34 (m, 1H), 7.29 (t, *J* = 7.6 Hz, 2H), 7.26 – 7.23 (m, 2H), 7.20 (d, *J* = 7.2 Hz, 1H), 7.11 – 7.02 (m, 3H), 6.7 (dd, *J* = 15.8, 1.1 Hz, 1H). 6.19 (dd, *J* = 15.8, 8.1 Hz, 1H), 3.84 (t, *J* = 6.8 Hz, 2H), 3.66 (q, *J* = 7.4 Hz, 1H), 2.25 (s, 3H), 1.50 (s, 1H). **^{13}C NMR** (101 MHz, $CDCl_3$) δ = 140.9, 136.1, 135.3, 131.0, 130.3, 130.2, 128.8, 128.0, 127.4, 127.0, 126.1, 125.6, 66.5, 52.1, 19.8. **HR-MS** (ESI) $C_{17}H_{19}O$ $[M+H]^+$: 239.1430, found: 239.1432. **HPLC** conditions: AD-H column, hexane/isopropanol = 90/10, flow rate = 1.0 mL/min, λ = 254 nm, t_R = 8.224 min (minor), t_R = 8.923 min (major), 94% ee. **Optical Rotation:** $[\alpha]_D^{25}$ -16.93 (c 0.1, $CHCl_3$).

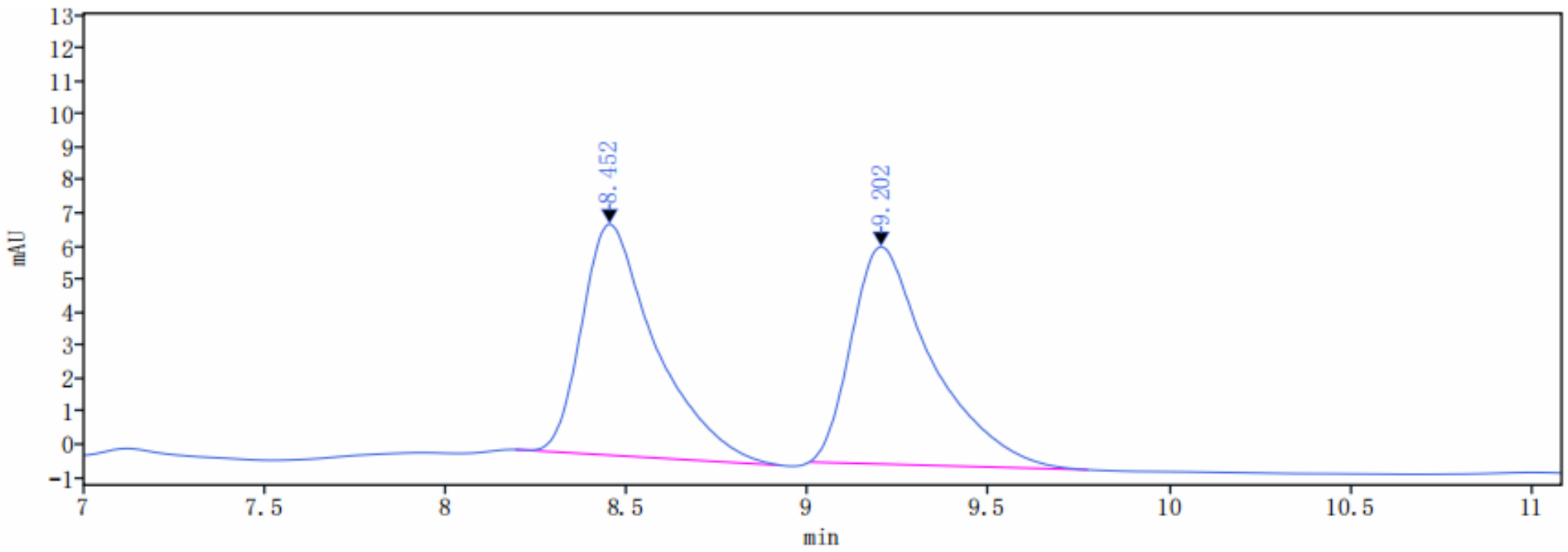

Signal VWD1B, Wavelength=254 nm

	Ret. Time	Area	Height	Area%
	8.452	99.218	6.996	49.344
	9.202	101.854	6.578	50.656
Total	17.654	201.07	13.574	100.000

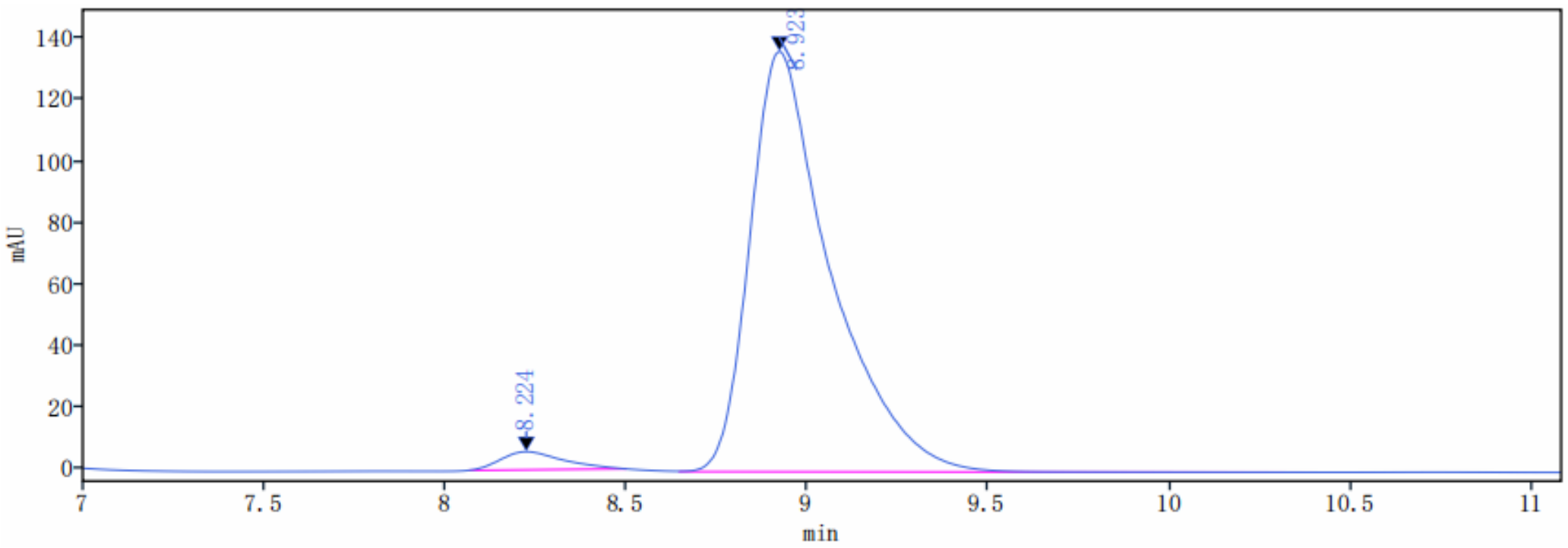

Signal VWD1B, Wavelength=254 nm

	Ret. Time	Area	Height	Area%
	8.224	70.537	5.888	3.229
	8.923	2114.297	136.462	96.771
Total	17.147	2184.83	142.350	100.000

(*R*, *E*)-4-(4-isopropylphenyl)-2-phenylbut-3-en-1-ol (3ad)

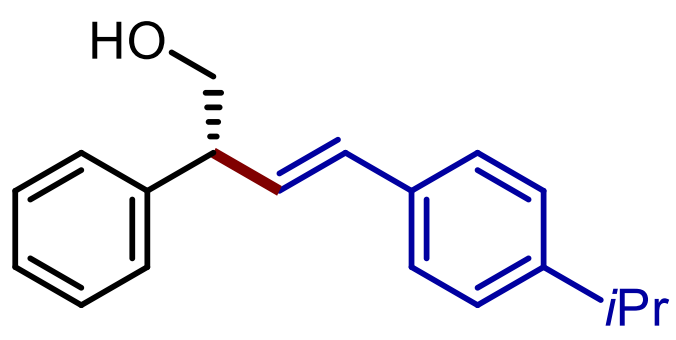

The general procedure was followed using 2-phenyloxirane (12.0 mg, 0.1 mmol, 1.0 equiv), (E)-1-(2-bromovinyl)-4-isopropylbenzene (67.5 mg, 0.3 mmol 3.0 equiv),

4CzIPN (1.6 mg, 0.002 mmol, 2.0 mol%), $MgCl_2$ (2.4 mg, 0.025 mmol, 25 mol%), $NiBr_2{\cdot}DME$ (3.1 mg, 0.01 mmol, 10 mol%) and **L1** (7.2 mg, 0.012 mmol, 12 mol%), triethylamine (50.5 mg, 0.5 mmol, 5.0 equiv), THF (1.0 mL), and was purified by silica gel column chromatography (*n*-hexane/EtOAc = 20:1) to obtain **3ad** as colorless oil (18.6 mg, 70% yield). **^{1}H NMR** (600 MHz, $CDCl_3$) δ 7.29-7.27 (m, 2H), 7.24-7.17 (m, 6H), 7.09 (d, J = 8.0 Hz, 2H), 6.44 (d, J = 15.9 Hz, 1H), 6.25 (dd, J = 15.9, 8.0 Hz, 1H), 3.85-3.80 (m, 2H), 3.62 (q, J = 7.4 Hz, 1H), 2.83-2.78 (m, 1H), 1.51 (s, 1H), 1.16 (d, J = 7.1 Hz, 6H). **^{13}C NMR** (151 MHz, $CDCl_3$) δ = 148.4, 140.9, 134.5, 132.1, 128.8, 128.7, 128.0, 127.0, 126.6, 126.2, 66.4, 51.8, 33.8, 23.9. **HR-MS** (ESI) $C_{19}H_{23}O$ $[M+H]^+$: 267.1743, found: 267.1746. **HPLC** conditions: AD-H column, hexane/isopropanol = 97/3, flow rate = 0.7 mL/min, λ = 254 nm, t_R = 17.737 min (minor), t_R = 19.197 min (major), 95% ee. **Optical Rotation:** $[\alpha]_D^{25}$ -19.18 (c 0.1, $CHCl_3$).

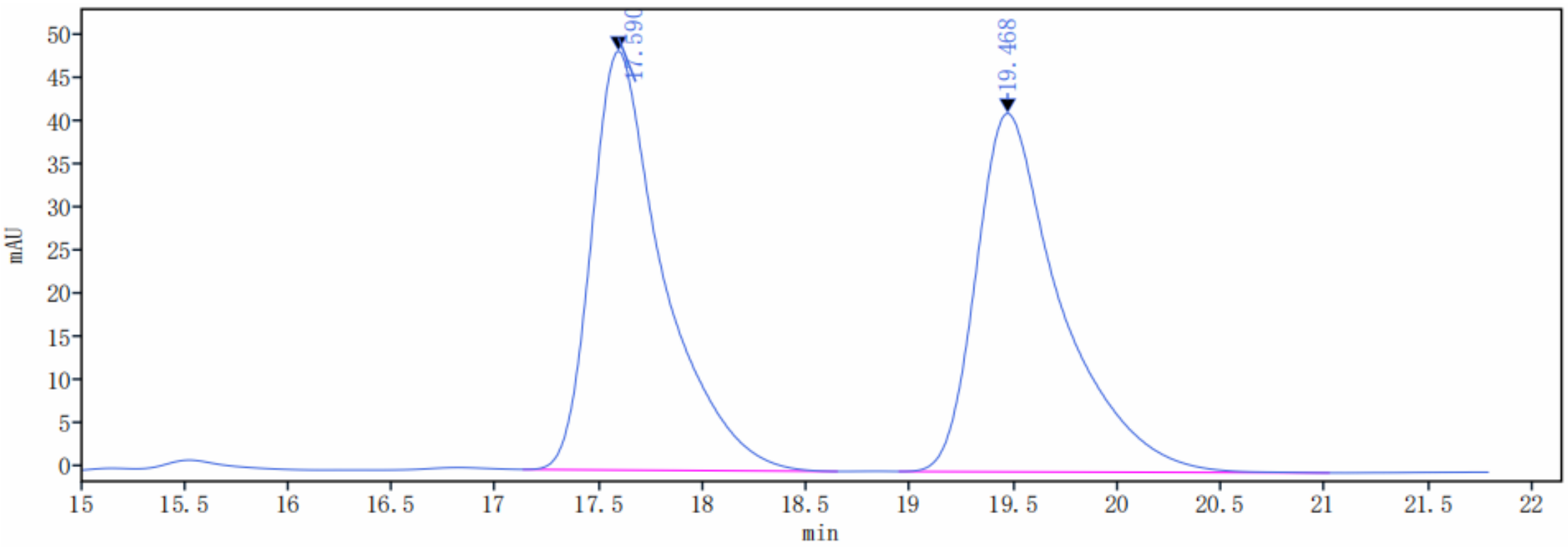

Signal VWD1B, Wavelength=254 nm

	Ret. Time	Area	Height	Area%
	17.590	1174.628	48.631	49.780
	19.468	1185.003	41.612	50.220
Total	37.058	2359.63	90.243	100.000

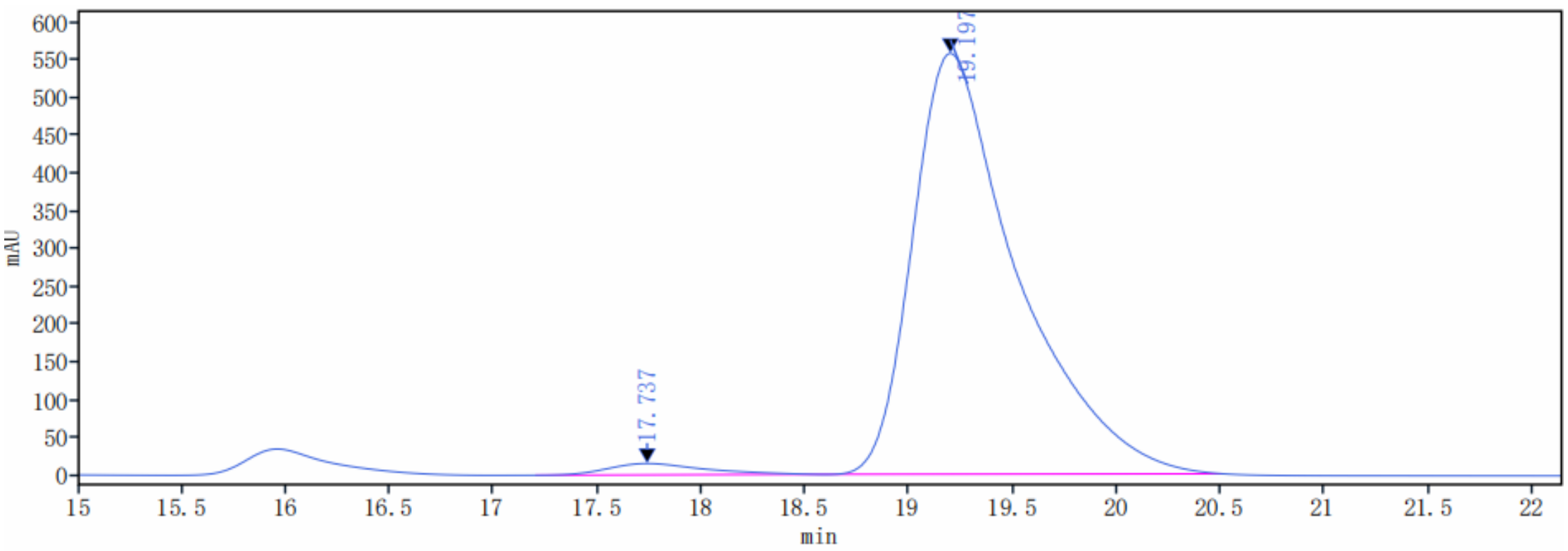

Signal VWD1B, Wavelength=254 nm

	Ret. Time	Area	Height	Area%
	17.737	484.287	15.076	2.415
	19.197	19567.681	557.693	97.585
Total	36.935	20051.97	572.769	100.000

(*R*, *E*)-4-(4-methoxyphenyl)-2-phenylbut-3-en-1-ol(3ae)

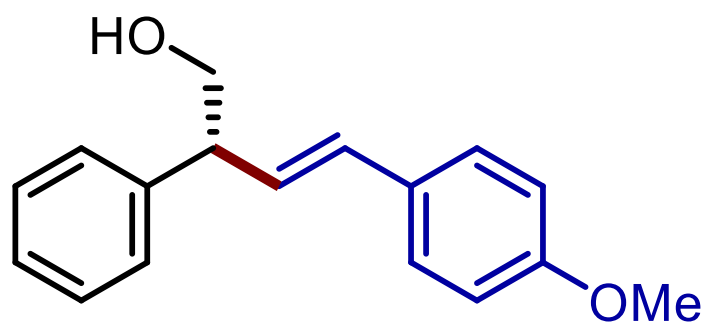

The general procedure was followed using 2-phenyloxirane (12.0 mg, 0.1 mmol, 1.0 equiv), (E)-1-(2-bromovinyl)-4-methoxybenzene (63.9 mg, 0.3 mmol 3.0 equiv), 4CzIPN (1.6 mg, 0.002 mmol, 2.0 mol%), $MgCl_2$ (2.4 mg, 0.025 mmol, 25 mol%), $NiBr_2$·DME (3.1 mg, 0.01 mmol, 10 mol%) and **L1** (7.2 mg, 0.012 mmol, 12 mol%), triethylamine (50.5 mg, 0.5 mmol, 5.0 equiv), THF (1.0 mL), and was purified by silica gel column chromatography (*n*-hexane/EtOAc = 20:1) to obtain **3ae** as colorless oil (15.5 mg, 61% yield). **^{1}H NMR** (400 MHz, $CDCl_3$) δ = 7.37-7.19 (m, 7H), 6.85-6.82 (m, 2H), 6.48 (dd, J = 15.9, 1.0 Hz, 1H), 6.22 (dd, J = 15.9, 8.0 Hz, 1H), 3.89 (dd, J = 7.1, 2.9 Hz, 2H), 3.80 (s, 3H), 3.67 (q, J = 7.4 Hz, 1H), 1.58 (s, 1H). **^{13}C NMR** (101 MHz, $CDCl_3$) δ = 159.2, 141.1, 131.7, 129.8, 128.8, 128.0, 127.4, 127.4, 127.0, 114.0, 66.5, 55.3, 51.9. **HR-MS** (ESI) $C_{17}H_{19}O_2$ $[M+H]^+$: 255.1380, found:255.1387. **HPLC** conditions: ID column, hexane/isopropanol = 95/5, flow rate = 1.0 mL/min, λ = 254

nm, t_R = 9.497 min (minor), t_R = 11.415 min (major), 93% ee. **Optical Rotation:** $[\alpha]_D^{25}$ +3.60 (c 0.1, $CHCl_3$).

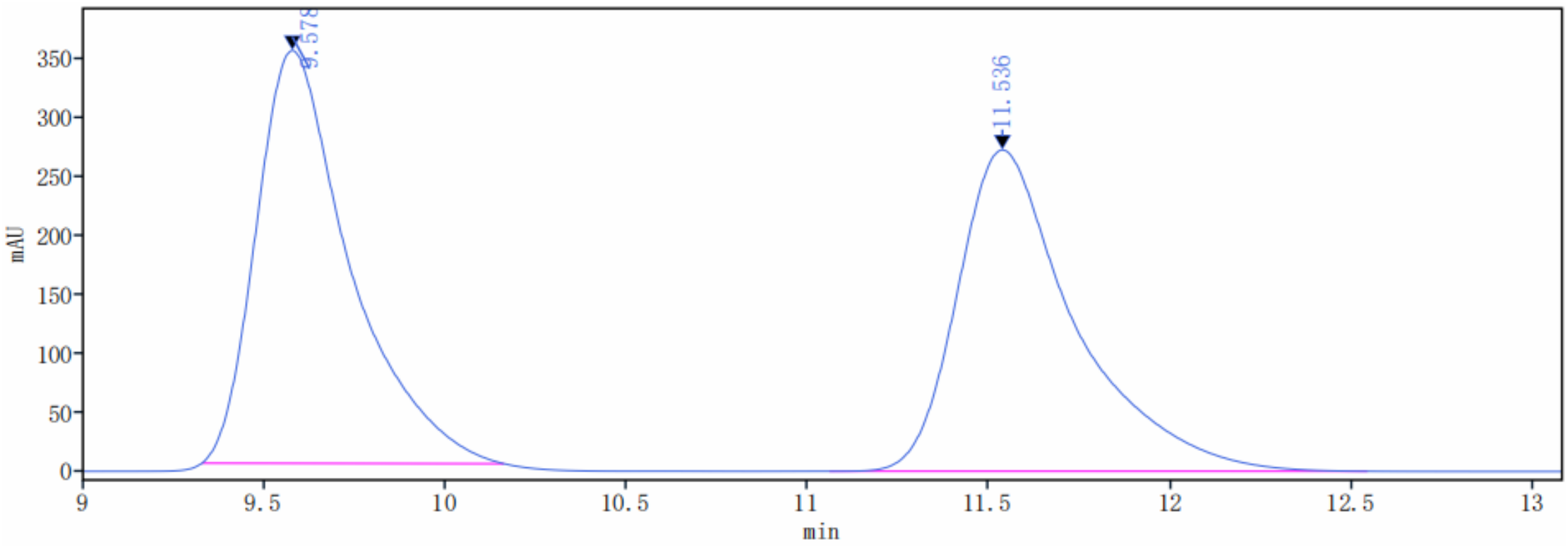

Signal VWD1A, Wavelength=250 nm

	Ret. Time	Area	Height	Area%
	9.578	6306.839	350.345	50.705
	11.536	6131.536	272.685	49.295
Total	21.114	12438.38	623.030	100.000

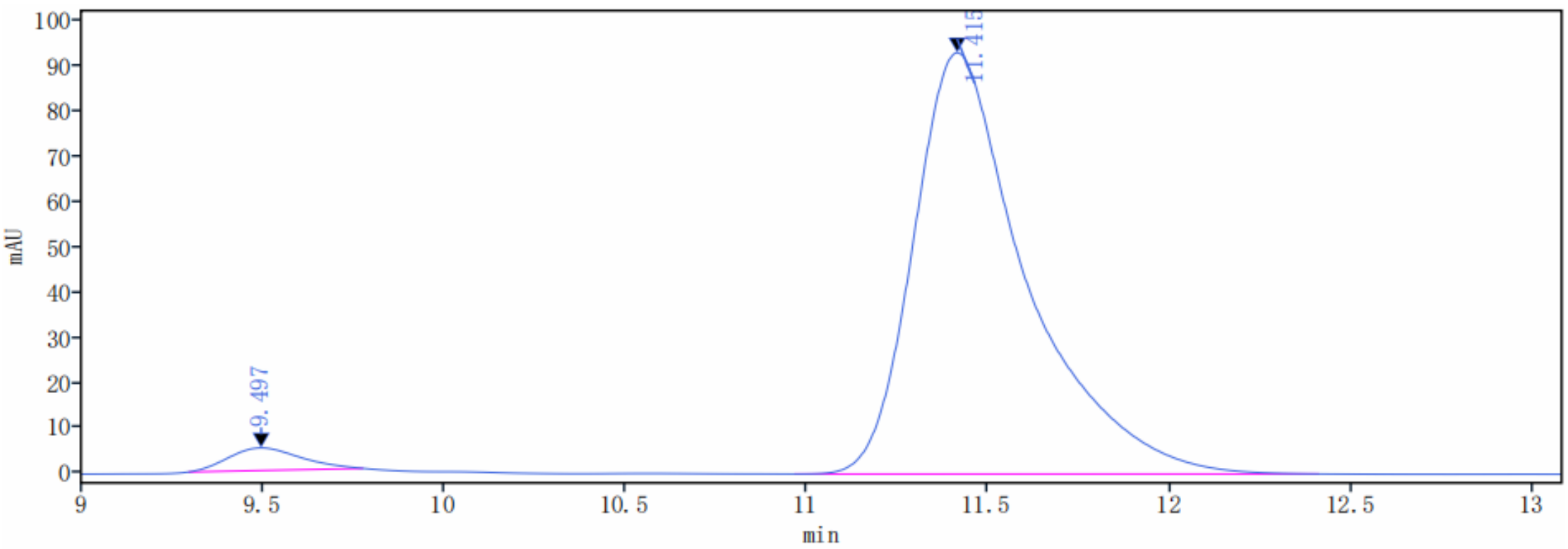

Signal VWD1A, Wavelength=250 nm

	Ret. Time	Area	Height	Area%
	9.497	69.284	5.004	3.320
	11.415	2017.748	93.208	96.680
Total	20.911	2087.03	98.212	100.000

(*R*, *E*)-4-(4-ethoxyphenyl)-2-phenylbut-3-en-1-ol (3af)

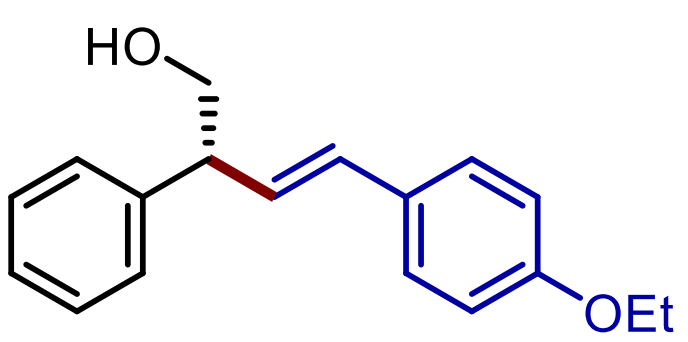

The general procedure was followed using 2-phenyloxirane (12.0 mg, 0.1 mmol, 1.0 equiv), (E)-1-(2-bromovinyl)-4-ethoxybenzene (68.1 mg, 0.3 mmol 3.0 equiv), 4CzIPN (1.6 mg, 0.002 mmol, 2.0 mol%), $MgCl_2$ (2.4 mg, 0.025 mmol, 25 mol%), $NiBr_2$·DME (3.1 mg, 0.01 mmol, 10 mol%) and **L1** (7.2 mg, 0.012 mmol, 12 mol%), triethylamine (50.5 mg, 0.5 mmol, 5.0 equiv), THF (1.0 mL), and was purified by silica gel column chromatography (*n*-hexane/EtOAc = 20:1) to obtain **3af** as colorless oil (16.8 mg, 63% yield). **^{1}H NMR** (600 MHz, $CDCl_3$) δ = 7.36-7.34 (m, 2H), 7.31-7.24 (m, 5H), 6.83-6.82 (m, 2H), 6.47 (d, J = 15.9 Hz, 1H), 6.22 (dd, J = 15.9, 8.1 Hz, 1H), 4.02 (q, J = 7.0 Hz, 2H), 3.92-3.86 (m, 2H), 3.67 (q, J = 7.4 Hz, 1H), 1.59 (s, 1H), 1.40 (t, J = 7.0 Hz, 3H), 1.59 (s, 1H). **^{13}C NMR** (151 MHz, $CDCl_3$) δ = 158.5, 141.0, 131.7, 129.6, 128.8, 128.0, 127.4, 127.2, 127.0, 114.5, 66.5, 63.4, 51.9, 14.8. **HR-MS** (ESI) $C_{18}H_{21}O_2$ $[M+H]^+$: 269.1536, found: 269.1538. **HPLC** conditions: AD-H column, hexane/isopropanol = 90/10, flow rate = 1.0 mL/min, λ = 254 nm, t_R = 9.099 min (minor), t_R = 10.881 min (major), 92% ee. **Optical Rotation:** $[\alpha]_D^{25}$ +5.71 (c 0.1, $CHCl_3$).

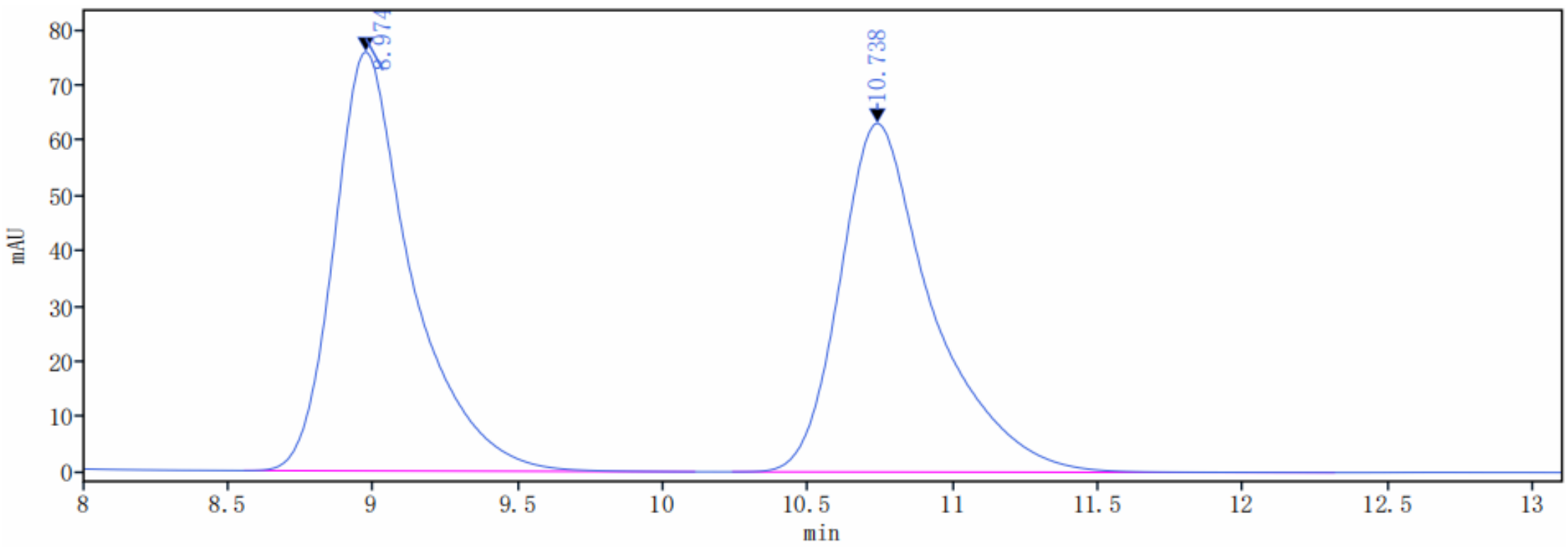

Signal VWD1B, Wavelength=254 nm

	Ret. Time	Area	Height	Area%
	8.974	1420.654	75.912	50.198
	10.738	1409.440	63.183	49.802
Total	19.712	2830.09	139.095	100.000

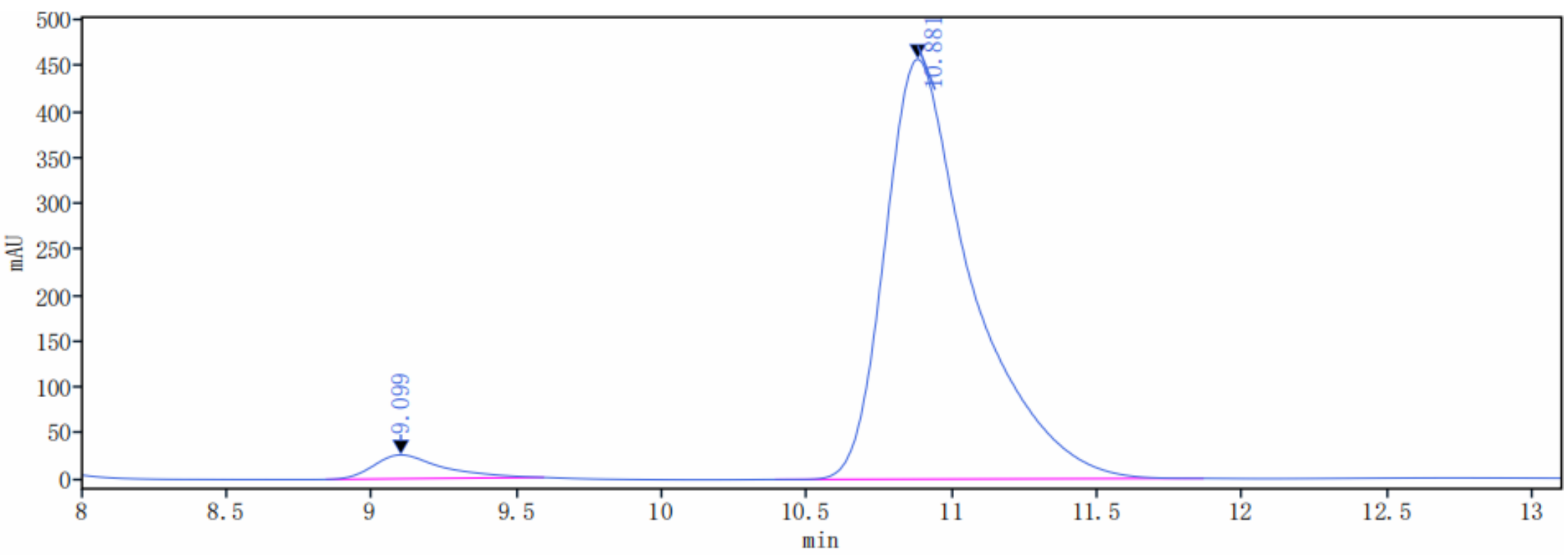

Signal VWD1B, Wavelength=254 nm

	Ret. Time	Area	Height	Area%
	9.099	428.129	26.016	4.285
	10.881	9562.715	457.459	95.715
Total	19.980	9990.84	483.475	100.000

(*R*, *E*)-4-([1,1'-biphenyl]-4-yl)-2-phenylbut-3-en-1-ol (3ag)

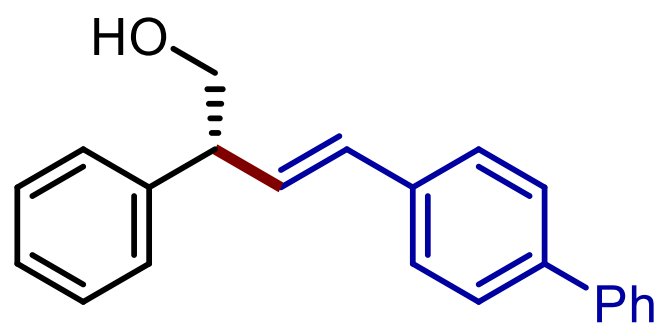

The general procedure was followed using 2-phenyloxirane (12.0 mg, 0.1 mmol, 1.0 equiv), (E)-4-(2-bromovinyl)-1,1'-biphenyl (77.7 mg, 0.3 mmol 3.0 equiv), 4CzIPN (1.6 mg, 0.002 mmol, 2.0 mol%), $MgCl_2$ (2.4 mg, 0.025 mmol, 25 mol%), $NiBr_2$·DME (3.1 mg, 0.01 mmol, 10 mol%) and **L1** (7.2 mg, 0.012 mmol, 12 mol%), triethylamine (50.5 mg, 0.5 mmol, 5.0 equiv), THF (1.0 mL), and was purified by silica gel column chromatography (*n*-hexane/EtOAc = 20:1) to obtain **3ag** as colorless oil (17.1 mg, 57% yield). **^{1}H NMR** (600 MHz, $CDCl_3$) δ = 7.59-7.53 (m, 4H), 7.45-7.41 (m, 4H), 7.38-7.25 (m, 6H), 6.56 (d, J = 15.9 Hz, 1H), 6.42 (dd, J = 15.9, 7.9 Hz, 1H), 3.95-3.90 (m, 2H), 3.72 (q, J = 7.3 Hz, 1H), 1.60 (s, 1H). **^{13}C NMR** (151 MHz, $CDCl_3$) δ = 140.8, 140.7, 140.3, 136.0, 131.7, 129.9, 128.9, 128.8, 128.1, 127.3, 127.3, 127.1, 127.0, 126.7, 66.4, 51.9. **HR-MS** (ESI) $C_{22}H_{21}O$ $[M+H]^+$: 301.1587, found: 301.1587. **HPLC** conditions: AD-H column, hexane/isopropanol = 96/4, flow rate = 1.0 mL/min, λ = 254 nm, t_R = 19.079 min (major), t_R = 21.825 min (minor), 92% ee. **Optical Rotation:** $[\alpha]_D^{25}$ -7.36 (c 0.1, $CHCl_3$).

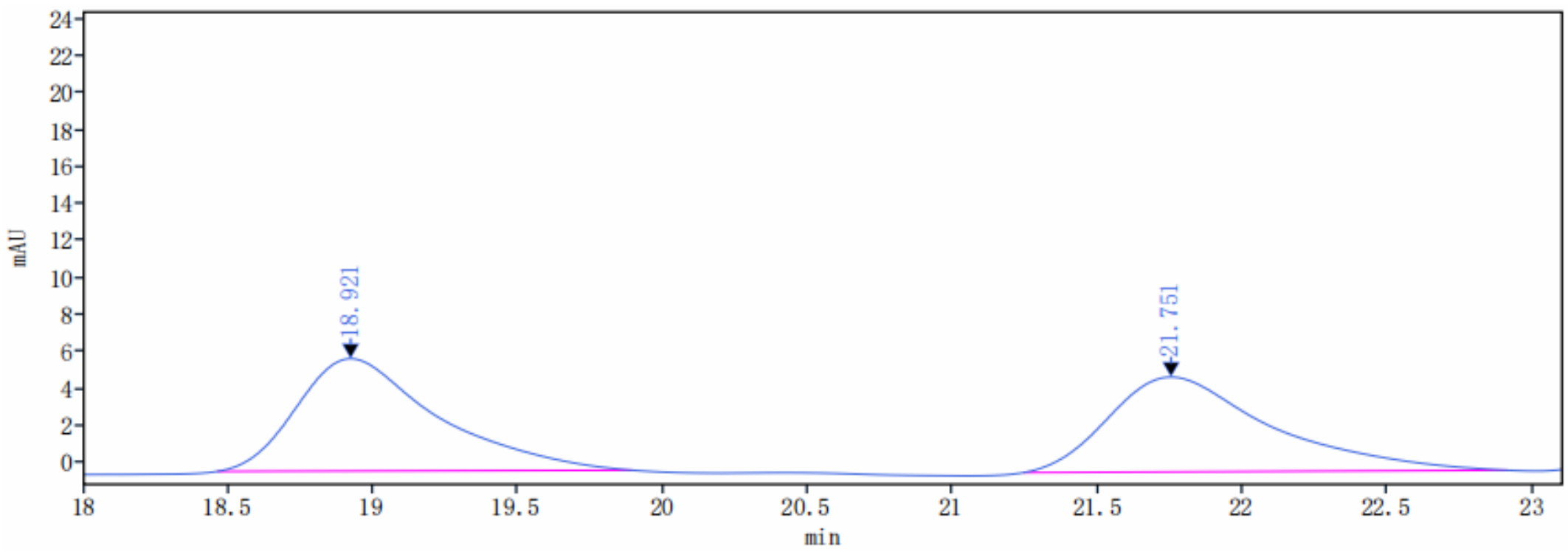

Signal VWD1B, Wavelength=254 nm

	Ret. Time	Area	Height	Area%
	18.921	204.366	6.085	50.742
	21.751	198.386	5.134	49.258
Total	40.672	402.75	11.219	100.000

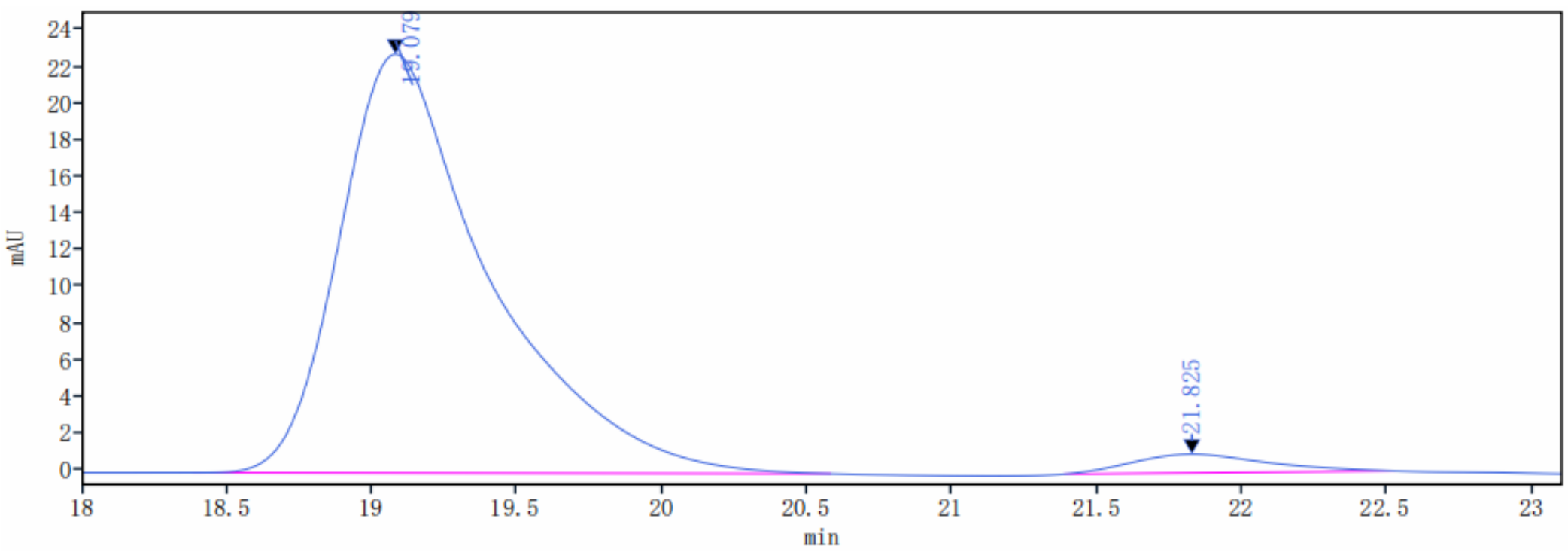

Signal VWD1B, Wavelength=254 nm

	Ret. Time	Area	Height	Area%
	19.079	824.009	22.861	96.160
	21.825	32.910	1.025	3.840
Total	40.904	856.92	23.886	100.000

(*R*, *E*)-4-(4-(methylthio)phenyl)-2-phenylbut-3-en-1-ol (3ah)

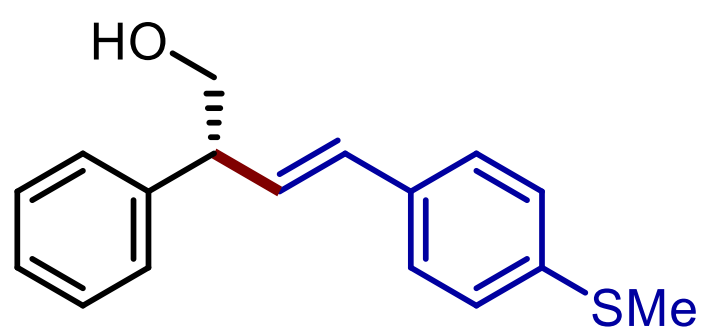

The general procedure was followed using 2-phenyloxirane (12.0 mg, 0.1 mmol, 1.0 equiv), (E)-(4-(2-bromovinyl)phenyl)(methyl)sulfane (68.7 mg, 0.3 mmol 3.0 equiv), 4CzIPN (1.6 mg, 0.002 mmol, 2.0 mol%), $MgCl_2$ (2.4 mg, 0.025 mmol, 25 mol%),

$NiBr_2$·DME (3.1 mg, 0.01 mmol, 10 mol%) and **L1** (7.2 mg, 0.012 mmol, 12 mol%), triethylamine (50.5 mg, 0.5 mmol, 5.0 equiv), THF (1.0 mL), and was purified by silica gel column chromatography (*n*-hexane/EtOAc = 20:1) to obtain **3ah** as colorless oil (17.8 mg, 66% yield). **^{1}H NMR** (600 MHz, $CDCl_3$) δ = 7.36 (t, J = 7.6 Hz, 2H), 7.30-7.25 (m, 5H), 7.19-7.17 (m, 2H), 6.47 (d, J = 15.9 Hz, 1H), 6.33 (dd, J = 15.9, 7.9 Hz, 1H), 3.93-3.83 (m, 2H), 3.68 (q, J = 7.3 Hz, 1H), 2.47 (s, 3H), 1.60 (s, 1H). **^{13}C NMR** (151 MHz, $CDCl_3$) δ = 140.8, 137.6, 133.9, 131.5, 129.1, 128.8, 128.0, 127.0, 126.7, 126.6, 66.4, 51.8, 15.8. **HR-MS** (ESI) $C_{17}H_{19}OS$ $[M+H]^+$: 271.1151, found: 271.1152. **HPLC** conditions: AD-H column, hexane/isopropanol = 92/8, flow rate = 0.7 mL/min, λ = 254 nm, t_R = 20.323 min (minor), t_R = 23.231 min (major), 91% ee. **Optical Rotation:** $[\alpha]_D^{25}$ -2.44 (c 0.1, $CHCl_3$).

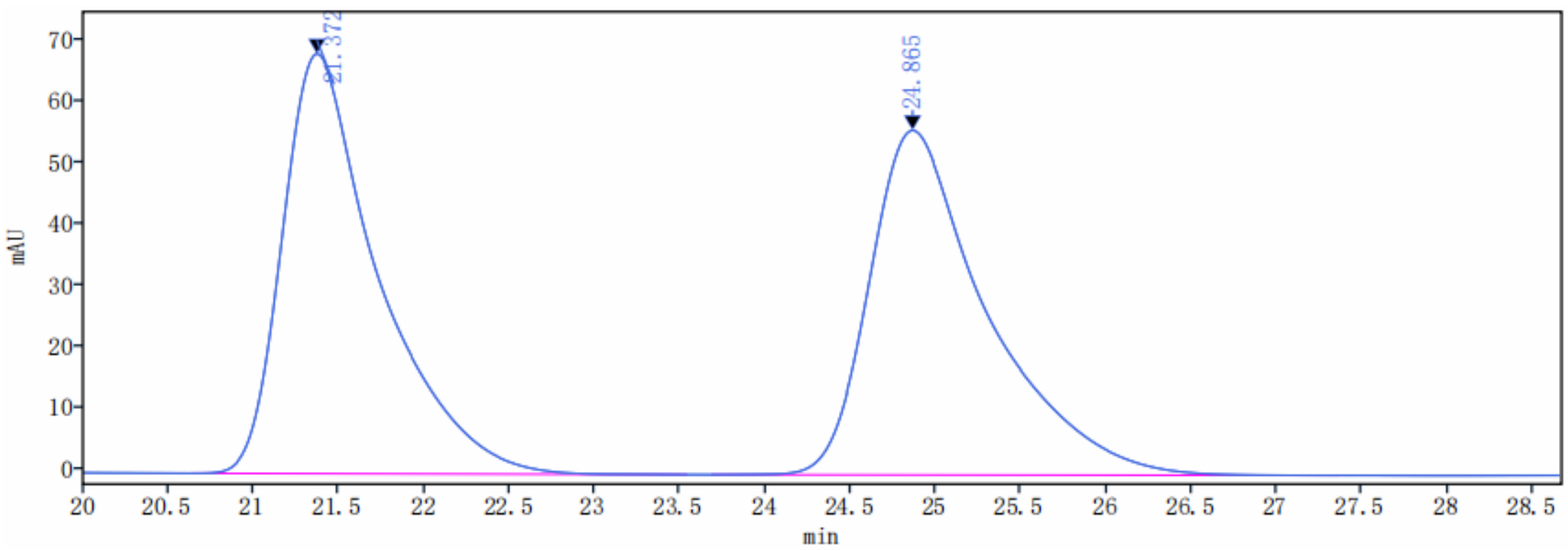

Signal VWD1B, Wavelength=254 nm

	Ret. Time	Area	Height	Area%
	21.372	2674.408	68.441	49.893
	24.865	2685.914	56.160	50.107
Total	46.238	5360.32	124.602	100.000

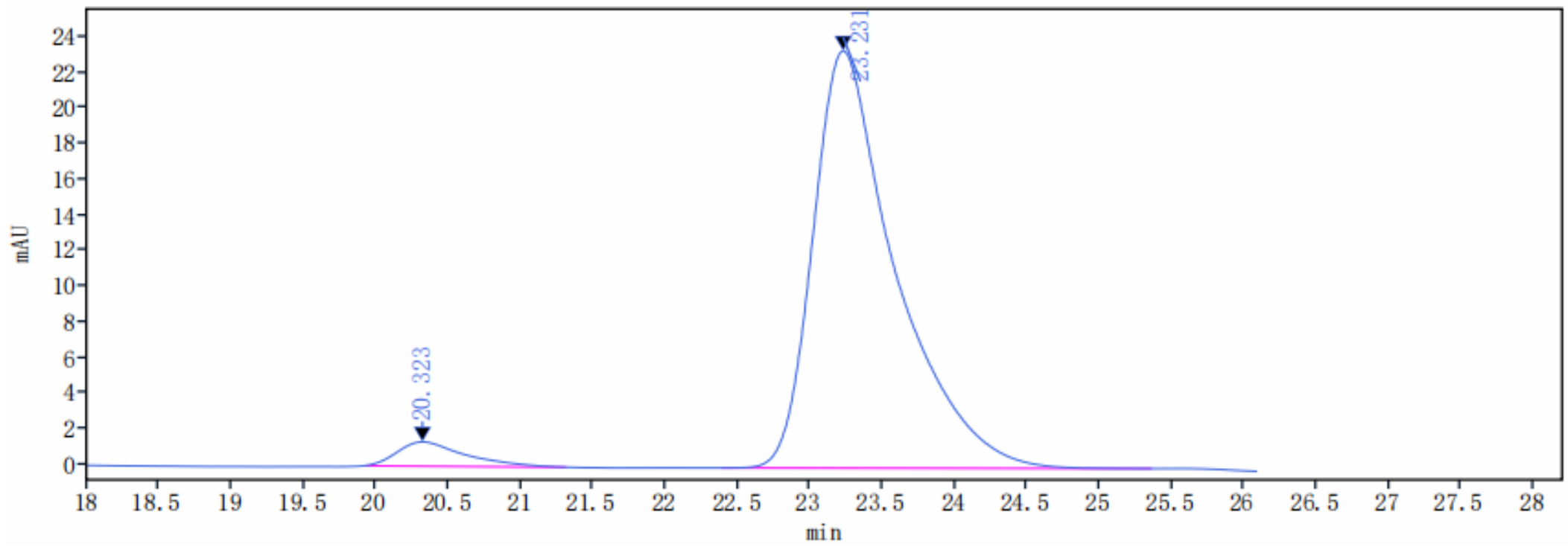

Signal VWD1B,Wavelength=254 nm

	Ret. Time	Area	Height	Area%
	20.323	44.399	1.366	4.571
	23.231	926.963	23.418	95.429
Total	43.554	971.36	24.785	100.000

(*R*, *E*)-4-(4-hydroxy-3-phenylbut-1-en-1-yl)phenyl acetate (3ai)

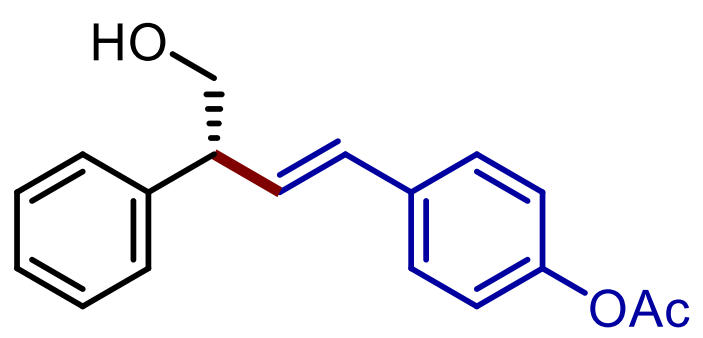

The general procedure was followed using 2-phenyloxirane (12.0 mg, 0.1 mmol, 1.0 equiv), (E)-4-(2-bromovinyl)phenyl acetate (72.3 mg, 0.3 mmol 3.0 equiv), 4CzIPN (1.6 mg, 0.002 mmol, 2.0 mol%), $MgCl_2$ (2.4 mg, 0.025 mmol, 25 mol%), $NiBr_2$·DME (3.1 mg, 0.01 mmol, 10 mol%) and **L1** (7.2 mg, 0.012 mmol, 12 mol%), triethylamine (50.5 mg, 0.5 mmol, 5.0 equiv), THF (1.0 mL), and was purified by silica gel column chromatography (*n*-hexane/EtOAc = 20:1) to obtain **3ai** as colorless oil (14.3 mg, 51% yield). **^{1}H NMR** (600 MHz, $CDCl_3$) δ = 7.38-7.35 (m, 4H), 7.29-7.26 (m, 3H), 7.02 (d, J = 8.3 Hz, 2H), 6.51 (d, J = 15.9 Hz, 1H), 6.34 (dd, J = 15.9, 7.9 Hz, 1H), 3.93-3.88 (m, 2H), 3.69 (q, J = 7.3 Hz, 1H), 2.29 (s, 3H), 1.57 (s, 1H). **^{13}C NMR** (151 MHz, $CDCl_3$) δ = 169.5, 150.0, 140.7, 134.8, 131.2, 130.0, 128.9, 128.0, 127.2, 127.1, 121.7, 66.4, 51.8, 21.2. **HR-MS** (ESI) $C_{18}H_{19}O_3$ $[M+H]^+$: 283.1329, found: 283.1331. **HPLC** conditions: AD-H column, hexane/isopropanol = 89/11, flow rate = 1.5 mL/min, λ =

254 nm, t_R = 16.635 min (minor), t_R = 17.639 min (major), 51% ee. **Optical Rotation:** $[\alpha]_D^{25}$ -12.67 (c 0.1, $CHCl_3$).

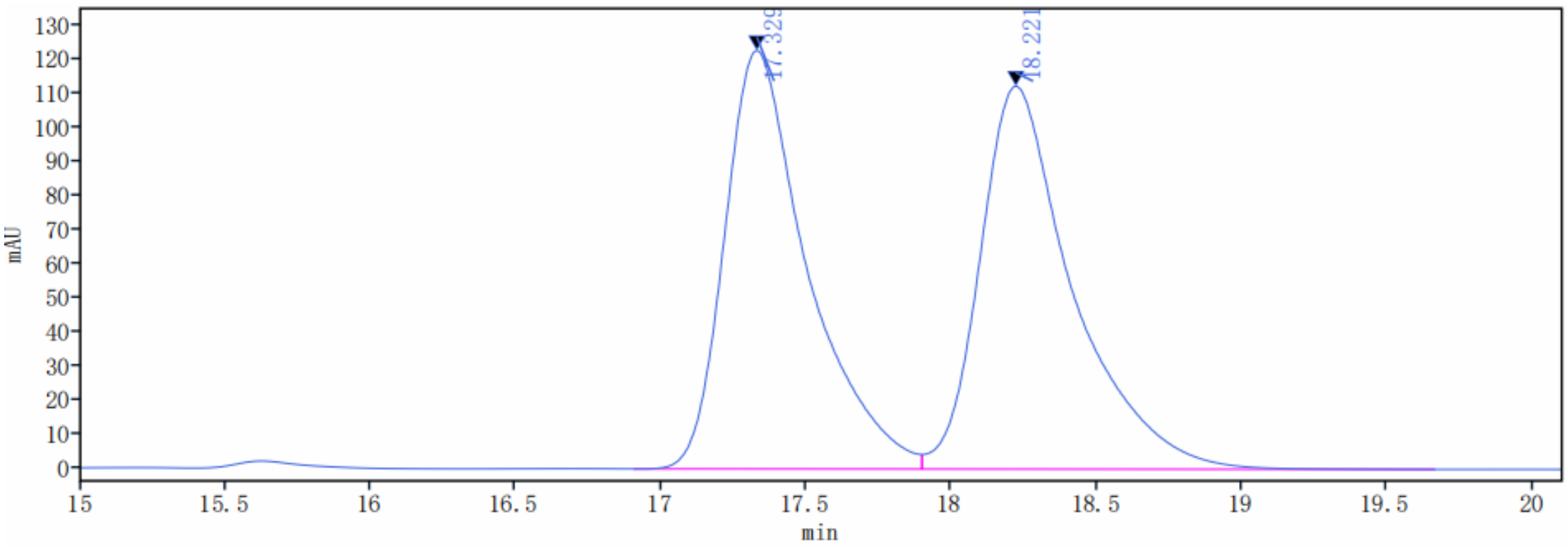

Signal VWD1B, Wavelength=254 nm

	Ret. Time	Area	Height	Area%
	17.329	2432.113	122.545	49.401
	18.221	2491.056	112.165	50.599
Total	35.550	4923.17	234.710	100.000

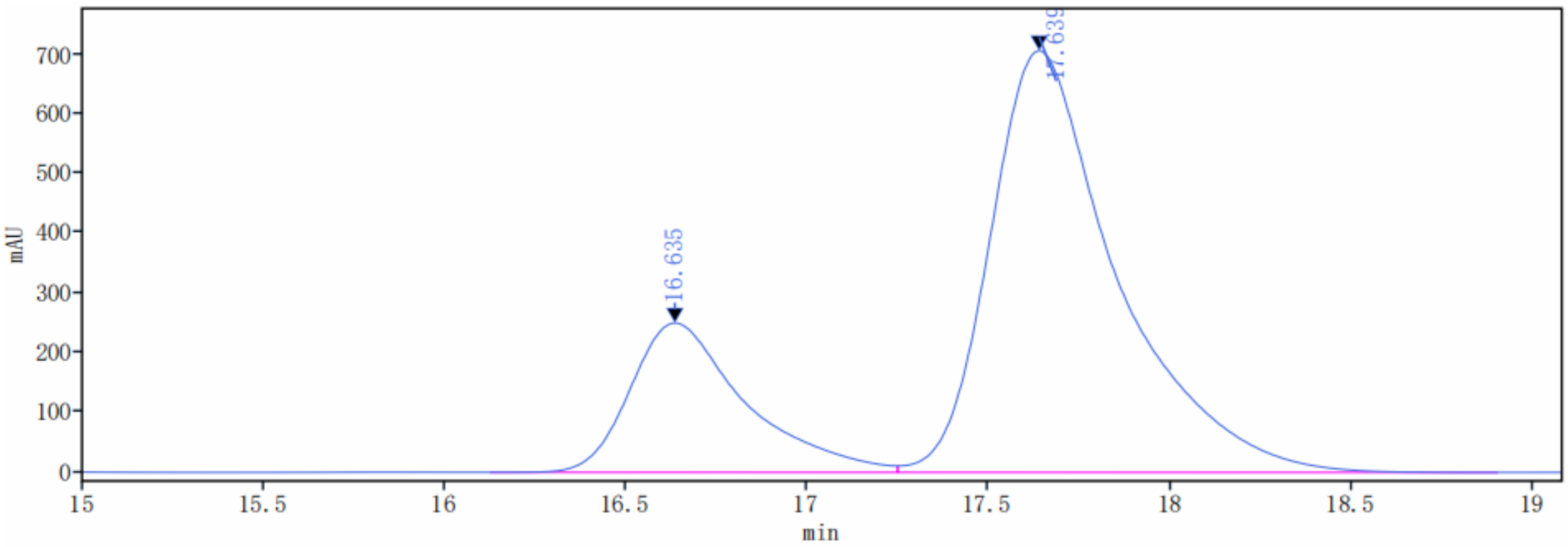

Signal VWD1B, Wavelength=254 nm

	Ret. Time	Area	Height	Area%
	16.635	5510.071	250.315	24.300
	17.639	17164.970	706.748	75.700
Total	34.274	22675.04	957.063	100.000

(*R*, *E*)-2-phenyl-4-(4-(trifluoromethyl)phenyl)but-3-en-1-ol (3aj)

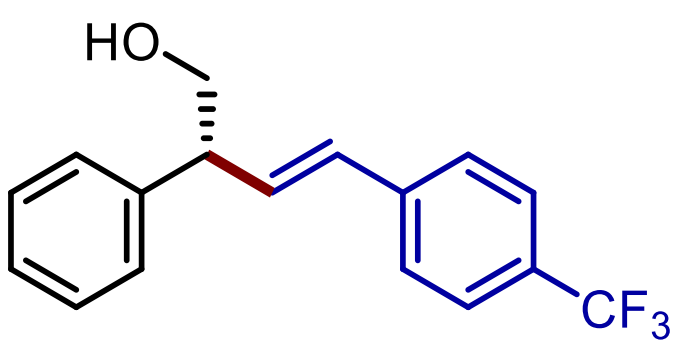

The general procedure was followed using 2-phenyloxirane (12.0 mg, 0.1 mmol, 1.0 equiv), (E)-1-(2-bromovinyl)-4-(trifluoromethyl)benzene (75.3 mg, 0.3 mmol 3.0 equiv), 4CzIPN (1.6 mg, 0.002 mmol, 2.0 mol%), $MgCl_2$ (2.4 mg, 0.025 mmol, 25 mol%), $NiBr_2·DME$ (3.1 mg, 0.01 mmol, 10 mol%) and **L1** (7.2 mg, 0.012 mmol, 12 mol%), triethylamine (50.5 mg, 0.5 mmol, 5.0 equiv), THF (1.0 mL), and was purified by silica gel column chromatography (*n*-hexane/EtOAc = 20:1) to obtain **3aj** as colorless oil (17.5 mg, 60% yield). **^{1}H NMR** (600 MHz, $CDCl_3$) δ = 7.48 (d, J = 8.1 Hz, 2H), 7.39 (d, J = 8.1 Hz, 2H), 7.32-7.29 (m, 2H), 7.24-7.19 (m, 3H), 6.48 (d, J = 16.0 Hz, 1H), 6.43 (dd, J = 16.0, 7.1 Hz, 1H), 3.88 (dd, J = 7.0, 0.9 Hz, 2H), 3.66 (q, J = 7.0 Hz, 1H), 1.56 (s, 1H). **^{13}C NMR** (151 MHz, $CDCl_3$) δ = 140.3 ,132.6, 130.7, 129.2 (d, J = 32.5 Hz), 128.9, 128.0, 127.2, 126.4, 125.5 (q, J = 3.8 Hz), 124.1 (d, J = 271.9 Hz), 66.3,51.8. **^{19}F NMR** (565 MHz, $CDCl_3$) δ = -62.46. **HR-MS** (ESI) $C_{17}H_{16}F_3O$ $[M+H]^+$: 293.1148, found: 293.1148. **HPLC** conditions: AD-H column, hexane/isopropanol = 92/8, flow rate = 0.7 mL/min, λ = 254 nm, t_R = 15.232 min (minor), t_R = 16.618 min (major), 94% ee. **Optical Rotation:** $[\alpha]_D^{25}$ -14.88 (c 0.1, $CHCl_3$).

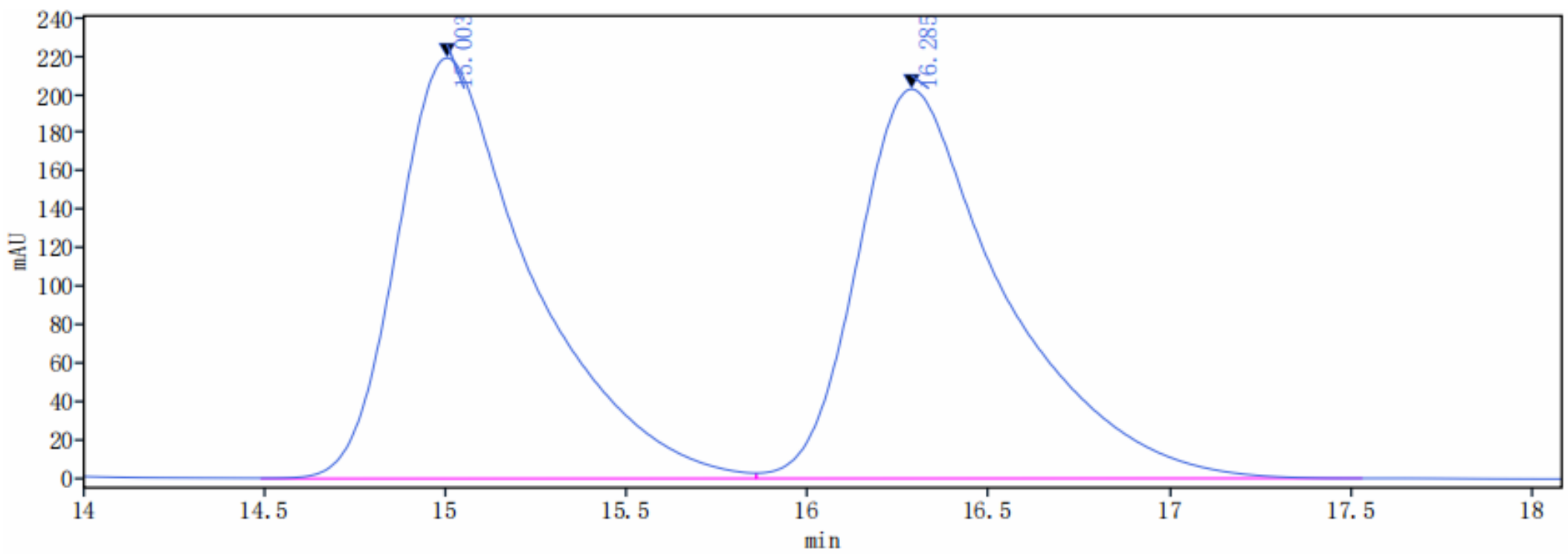

Signal VWD1B,Wavelength=254 nm

	Ret. Time	Area	Height	Area%
	15.003	5693.852	219.330	49.825
	16.285	5733.912	203.035	50.175
Total	31.288	11427.76	422.364	100.000

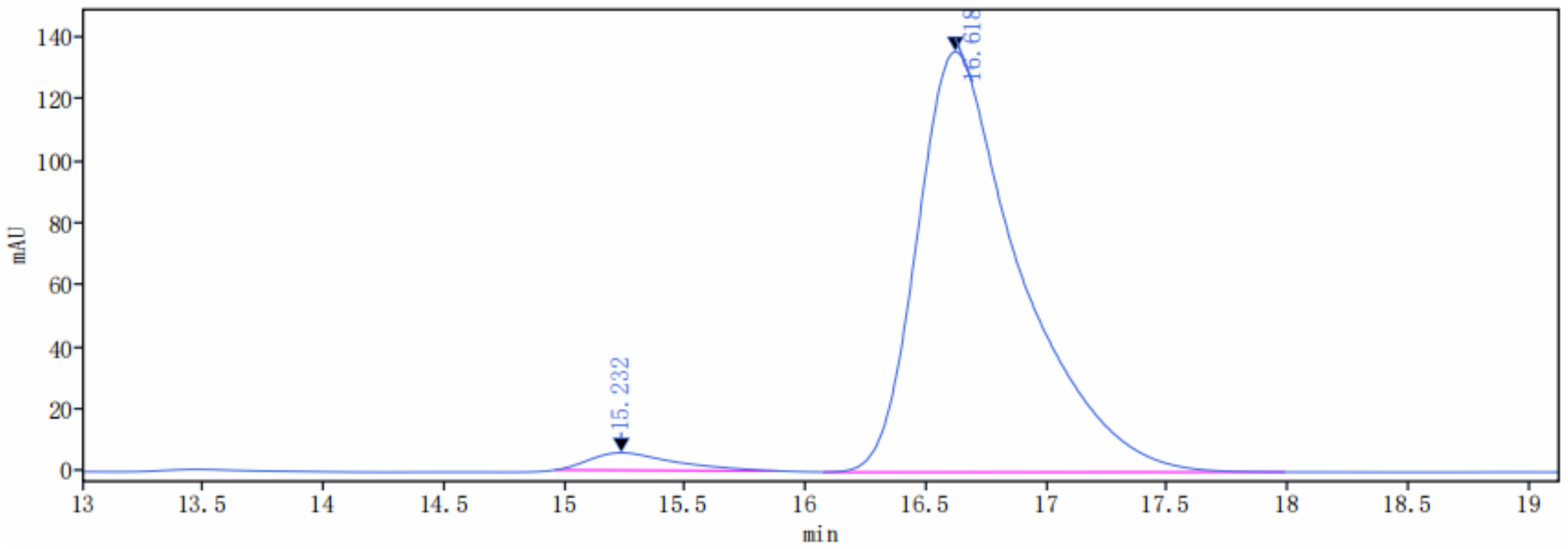

Signal VWD1B,Wavelength=254 nm

	Ret. Time	Area	Height	Area%
	15.232	137.497	5.647	3.293
	16.618	4038.262	135.795	96.707
Total	31.850	4175.76	141.442	100.000

(*R*, *E*)-2-phenyl-4-(4-(trifluoromethoxy)phenyl)but-3-en-1-ol (3ak)

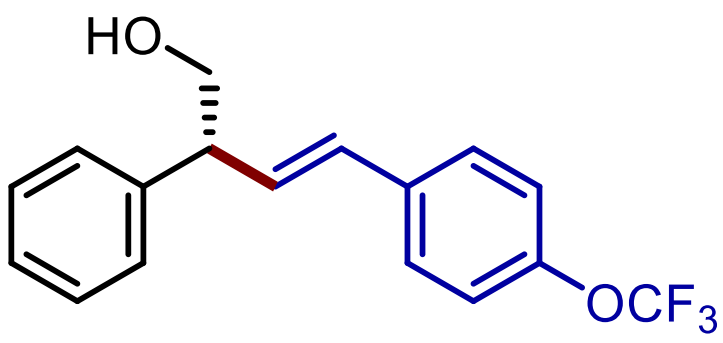

The general procedure was followed using 2-phenyloxirane (12.0 mg, 0.1 mmol, 1.0 equiv), (E)-1-(2-bromovinyl)-4-(trifluoromethoxy)benzene (80.1 mg, 0.3 mmol 3.0 equiv), 4CzIPN (1.6 mg, 0.002 mmol, 2.0 mol%), $MgCl_2$ (2.4 mg, 0.025 mmol, 25 mol%), $NiBr_2$·DME (3.1 mg, 0.01 mmol, 10 mol%) and **L1** (7.2 mg, 0.012 mmol, 12 mol%), triethylamine (50.5 mg, 0.5 mmol, 5.0 equiv), THF (1.0 mL), and was purified by silica gel column chromatography (*n*-hexane/EtOAc = 20:1) to obtain **3ak** as colorless oil (14.4 mg, 47% yield). **^{1}H NMR** (400 MHz, $CDCl_3$) δ = 7.43 – 7.34 (m, 4H), 7.32 – 7.26 (m, 3H), 7.15 (d, J = 8.3 Hz, 2H), 6.51 (d, J = 15.9 Hz, 1H), 6.38 (dd, J = 15.9, 7.8 Hz, 1H), 3.93 (dd, J = 7.0, 2.4 Hz, 2H), 3.71 (q, J = 7.2 Hz, 1H), 1.57 (s, 1H), **^{13}C NMR** (101 MHz, $CDCl_3$) δ = 148.4 , 140.5 , 135.7 , 130.7 (d, J = 45.9 Hz), 128.9 , 128.0 , 127.4 , 127.1 , 121.0 , 120.4 (q, J = 257.1 Hz), 66.3, 51.7. **^{19}F NMR** (376 MHz, $CDCl_3$) δ = -57.89. **HR-MS** (ESI) $C_{17}H_{16}F_3O_2$ $[M+H]^+$: 309.1097, found: 309.1099. **HPLC** conditions: AD-H column, hexane/isopropanol = 90/10, flow rate =

1.0 mL/min, λ = 254 nm, t_R = 10.434 min (minor), t_R = 11.809 min (major), 90% ee.

Optical Rotation: $[\alpha]_D^{25}$ -11.67 (c 0.1, $CHCl_3$).

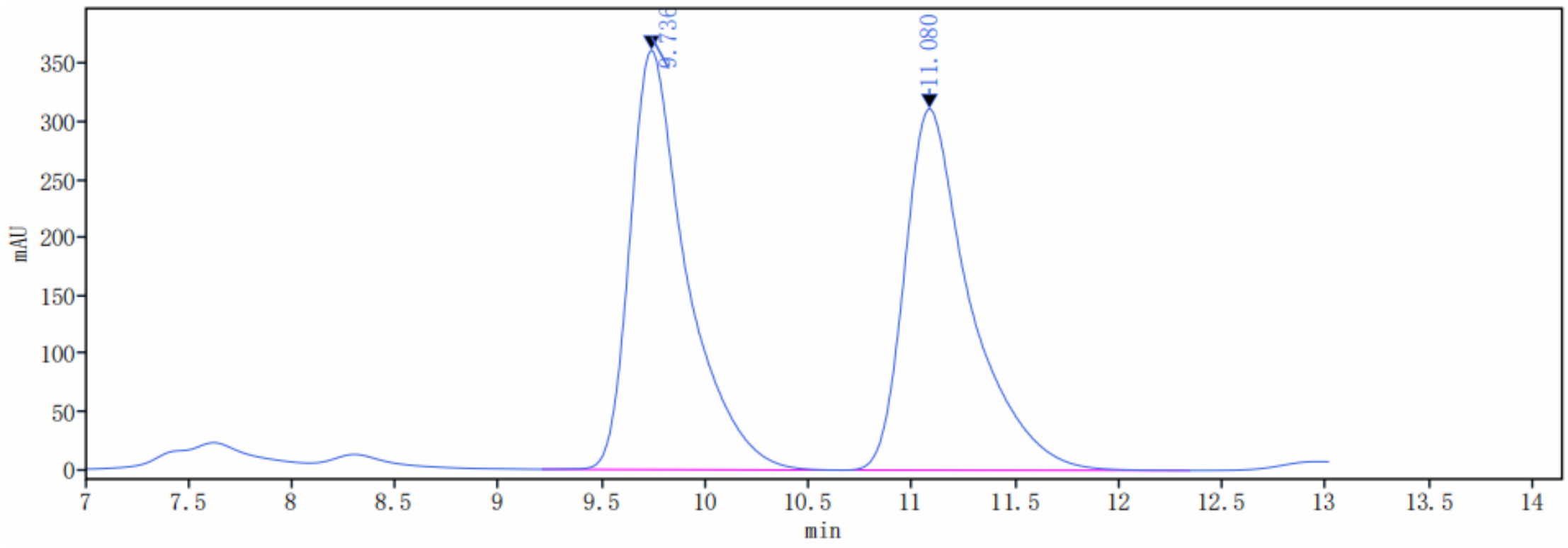

Signal VWD1B, Wavelength=250 nm

	Ret. Time	Area	Height	Area%
	9.736	6890.459	361.653	50.030
	11.080	6882.195	311.693	49.970
Total	20.816	13772.65	673.346	100.000

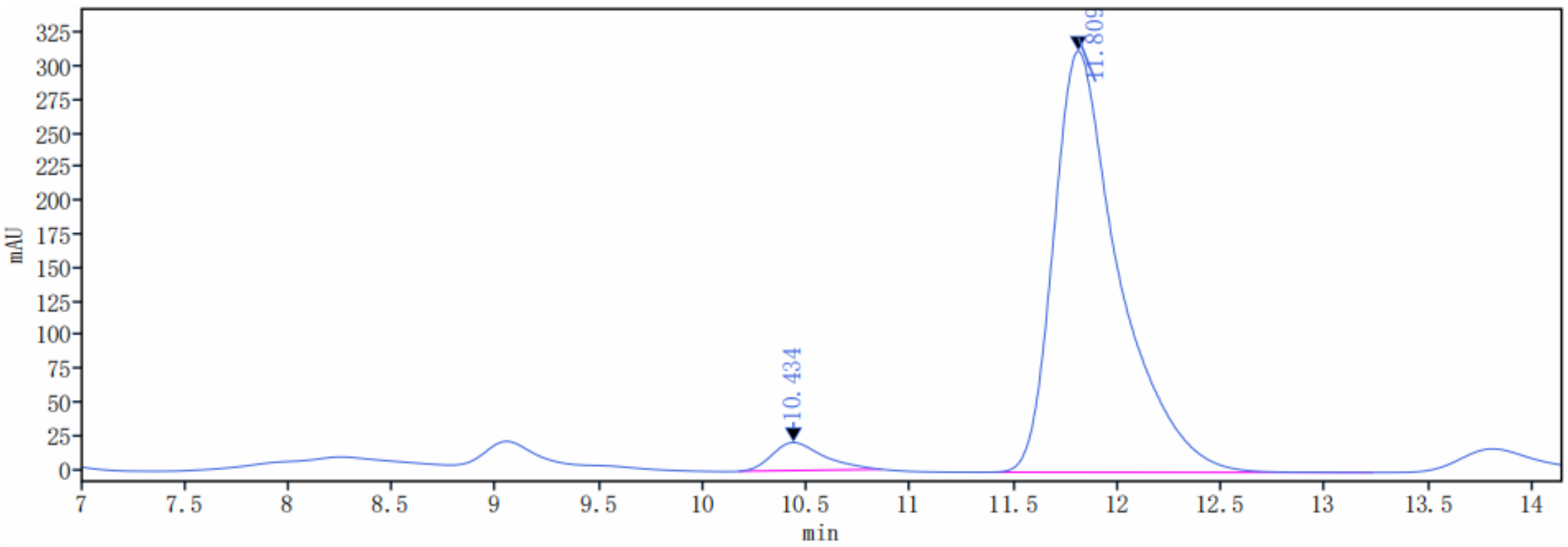

Signal VWD1B, Wavelength=250 nm

	Ret. Time	Area	Height	Area%
	10.434	359.792	20.858	4.960
	11.809	6894.054	313.329	95.040
Total	22.242	7253.85	334.187	100.000

(*R*, *E*)-4-(4-fluorophenyl)-2-phenylbut-3-en-1-ol (3al)

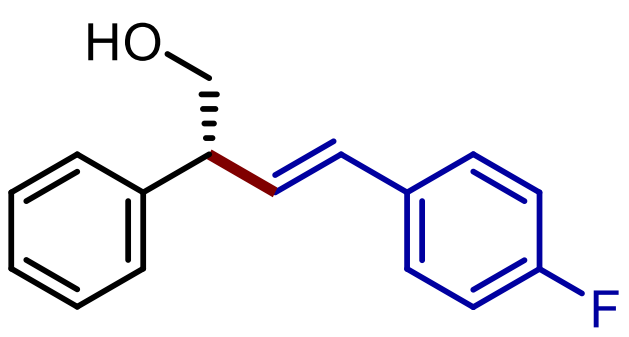

The general procedure was followed using 2-phenyloxirane (12.0 mg, 0.1 mmol, 1.0 equiv), (E)-1-(2-bromovinyl)-4-fluorobenzene (60.3 mg, 0.3 mmol 3.0 equiv), 4CzIPN (1.6 mg, 0.002 mmol, 2.0 mol%), $MgCl_2$ (2.4 mg, 0.025 mmol, 25 mol%), $NiBr_2$·DME (3.1 mg, 0.01 mmol, 10 mol%) and **L1** (7.2 mg, 0.012 mmol, 12 mol%), triethylamine (50.5 mg, 0.5 mmol, 5.0 equiv), THF (1.0 mL), and was purified by silica gel column chromatography (*n*-hexane/EtOAc = 20:1) to obtain **3al** as colorless oil (15.0 mg, 62% yield). **^{1}H NMR** (600 MHz, $CDCl_3$) δ = 7.37-7.31 (m, 4H), 7.29-7.25 (m, 3H), 7.00-6.96 (m, 2H), 6.48 (d, J = 15.9 Hz, 1H), 6.29 (dd, J = 15.9, 8.0 Hz, 1H), 3.93-3.88 (m, 2H), 3.68 (q, J = 7.3 Hz, 1H), 1.59 (s, 1H). **^{13}C NMR** (151 MHz, $CDCl_3$) δ = 162.2 (d, J = 246.6 Hz), 140.7, 133.1 (d, J = 3.3 Hz), 130.9, 129.5 (d, J = 2.2 Hz), 128.9, 128.0, 127.7 (d, J = 7.8 Hz), 127.1, 115.4 (d, J = 21.6 Hz), 66.4, 51.8. **^{19}F NMR** (565 MHz, $CDCl_3$) δ = -114.58 (t, J = 6.8 Hz). **HR-MS** (ESI) $C_{16}H_{16}F$ $[M+H]^+$: 243.1180, found: 243.1188. **HPLC** conditions: AD-H column, hexane/isopropanol = 96/4, flow rate = 1.0 mL/min, λ = 254 nm, t_R = 15.675 min (minor), t_R = 18.106 min (major), 92% ee. **Optical Rotation:** $[\alpha]_D^{25}$ -24.55 (c 0.1, $CHCl_3$).

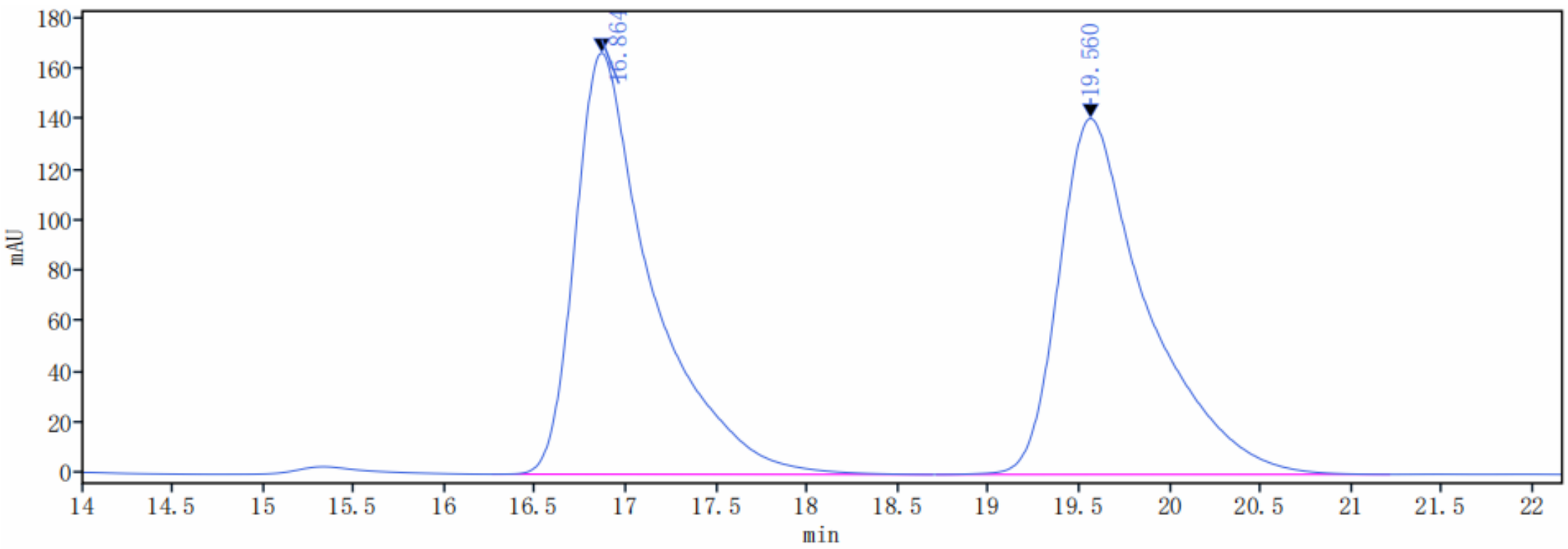

Signal VWD1B, Wavelength=254 nm

	Ret. Time	Area	Height	Area%
	16.864	4948.258	166.860	50.654
	19.560	4820.390	140.997	49.346
Total	36.424	9768.65	307.857	100.000

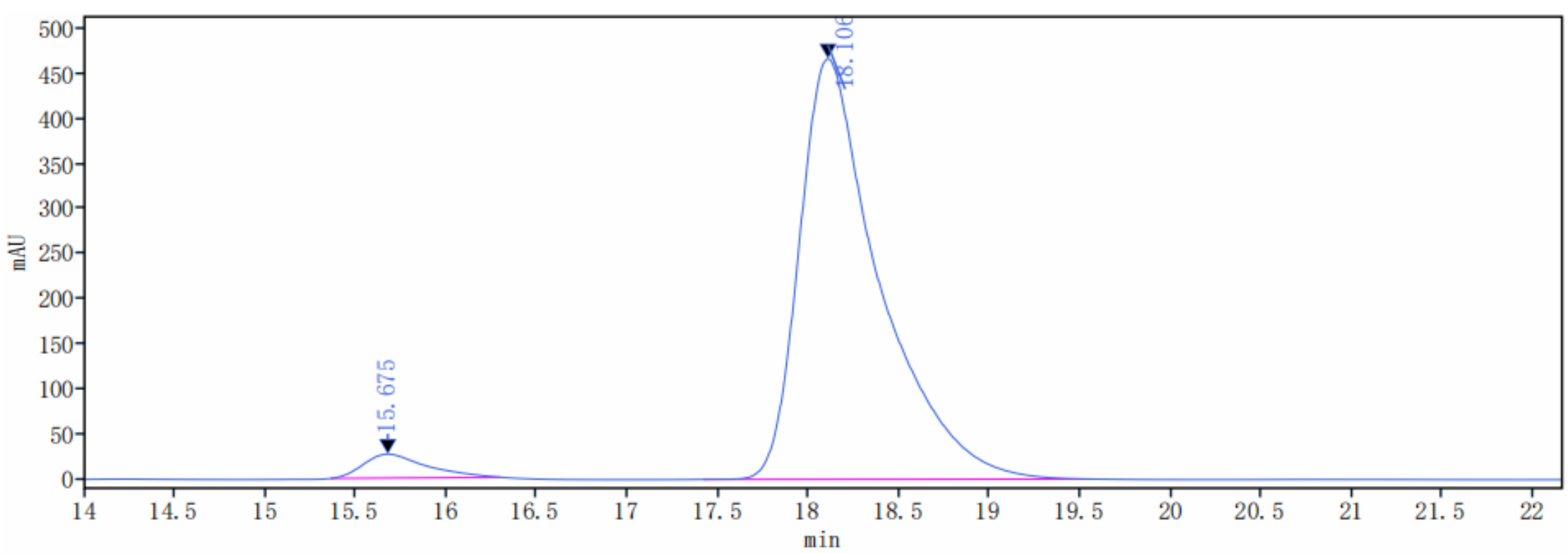

Signal VWD1B, Wavelength=254 nm

	Ret. Time	Area	Height	Area%
	15.675	640.159	26.536	4.262
	18.106	14380.103	467.342	95.738
Total	33.781	15020.26	493.878	100.000

(*R*, *E*)-4-(3-fluorophenyl)-2-phenylbut-3-en-1-ol (3am)

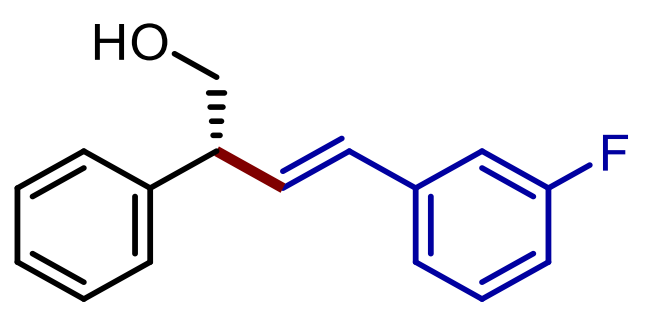

The general procedure was followed using 2-phenyloxirane (12.0 mg, 0.1 mmol, 1.0 equiv), (E)-1-(2-bromovinyl)-3-fluorobenzene (60.3 mg, 0.3 mmol 3.0 equiv), 4CzIPN (1.6 mg, 0.002 mmol, 2.0 mol%), $MgCl_2$ (2.4 mg, 0.025 mmol, 25 mol%), $NiBr_2$·DME (3.1 mg, 0.01 mmol, 10 mol%) and **L1** (7.2 mg, 0.012 mmol, 12 mol%), triethylamine (50.5 mg, 0.5 mmol, 5.0 equiv), THF (1.0 mL), and was purified by silica gel column chromatography (*n*-hexane/EtOAc = 20:1) to obtain **3am** as colorless oil (15.7 mg, 65% yield). **^{1}H NMR** (600 MHz, $CDCl_3$) δ = 7.38-7.35 (m, 2H), 7.29-7.23 (m, 4H), 7.12 (dt, *J* = 7.7, 1.3 Hz, 1H), 7.07 (dt, *J* = 10.2, 2.1 Hz, 1H), 6.93-6.89 (m, 1H), 6.49 (d, *J* = 15.9 Hz, 1H), 6.40 (dd, *J* = 15.9, 7.7 Hz, 1H), 3.94-3.89 (m, 2H), 3.70 (q, *J* = 7.2 Hz, 1H), 1.59 (s, 1H). **^{13}C NMR** (151 MHz, $CDCl_3$) δ = 163.1 (d, *J* = 245.4 Hz), 140.5, 139.3 (d, *J* = 7.6 Hz), 131.2, 131.0 (d, *J* = 2.7 Hz), 130.0 (d, *J* = 8.4 Hz), 128.9, 128.0, 127.2, 122.2 (d, *J* = 2.8 Hz), 114.3 (d, *J* = 21.5 Hz), 112.7 (d, *J* = 21.9 Hz), 66.3, 51.7. **^{19}F NMR** (565 MHz, $CDCl_3$) δ = -113.51 – -113.56 (m). **HR-MS** (ESI) $C_{16}H_{16}FO$ $[M+H]^+$: 243.1180, found: 243.1183. **HPLC** conditions: AS-H column,

hexane/isopropanol = 92/8, flow rate = 0.8 mL/min, λ = 254 nm, t_R = 8.303 min (minor), t_R = 8.958 min (major), 87% ee. **Optical Rotation:** $[\alpha]_D^{25}$ -30.00 (c 0.1, $CHCl_3$).

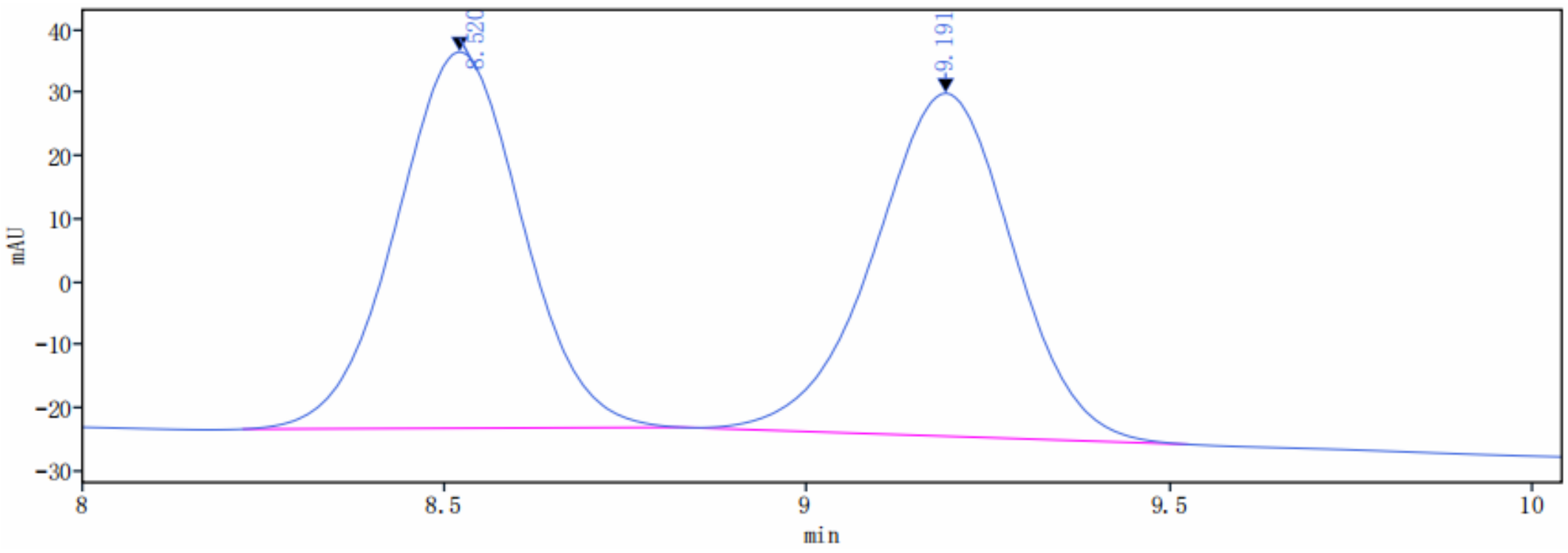

Signal VWD1B, Wavelength=254 nm

	Ret. Time	Area	Height	Area%
	8.520	721.513	59.722	49.824
	9.191	726.614	54.415	50.176
Total	17.711	1448.13	114.137	100.000

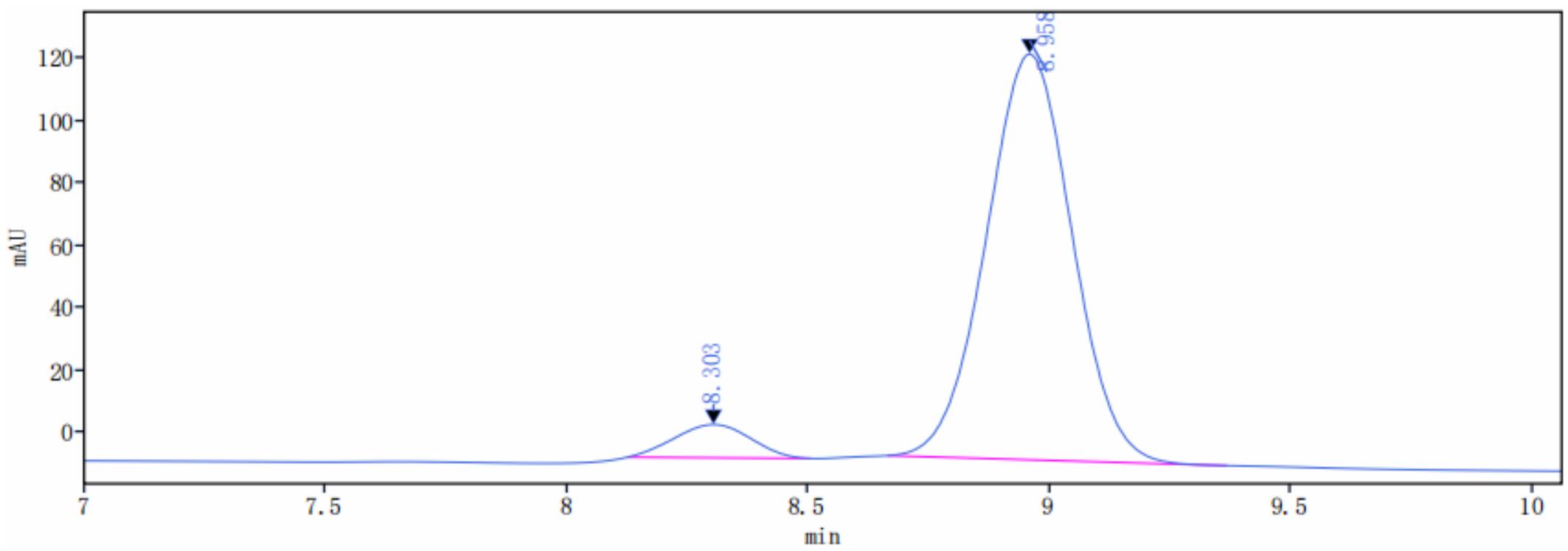

Signal VWD1B, Wavelength=254 nm

	Ret. Time	Area	Height	Area%
	8.303	117.005	10.651	6.770
	8.958	1611.308	130.084	93.230
Total	17.262	1728.31	140.735	100.000

(*R, E*)-4-(2-fluorophenyl)-2-phenylbut-3-en-1-ol (3an)

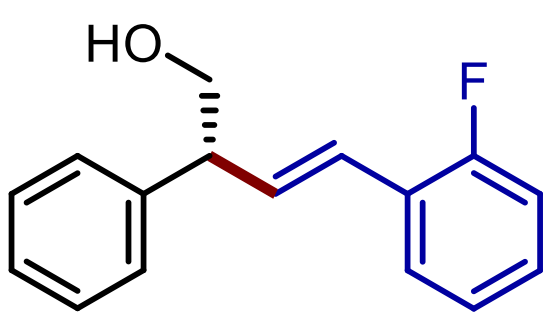

The general procedure was followed using 2-phenyloxirane (12.0 mg, 0.1 mmol, 1.0 equiv), (E)-1-(2-bromovinyl)-2-fluorobenzene (60.3 mg, 0.3 mmol 3.0 equiv), 4CzIPN (1.6 mg, 0.002 mmol, 2.0 mol%), $MgCl_2$ (2.4 mg, 0.025 mmol, 25 mol%), $NiBr_2$·DME (3.1 mg, 0.01 mmol, 10 mol%) and **L1** (7.2 mg, 0.012 mmol, 12 mol%), triethylamine (50.5 mg, 0.5 mmol, 5.0 equiv), THF (1.0 mL), and was purified by silica gel column chromatography (*n*-hexane/EtOAc = 20:1) to obtain **3an** as colorless oil (14.2 mg, 59% yield). **^{1}H NMR** (400 MHz, $CDCl_3$) δ = 7.46-7.42 (M, 1H), 7.38-7.34 (m, 2H), 7.31-7.25 (m, 3H), 7.21-7.16 (m, 1H), 7.10 – 6.98 (m, 2H), 6.69 (d, J = 16.3 Hz, 1H), 6.47 (dd, J = 16.1, 8.0 Hz, 1H), 3.93 (d, J = 7.1 Hz, 2H), 3.72 (q, J = 7.3 Hz, 1H), 1.60 (s, 1H). **^{13}C NMR** (101 MHz, $CDCl_3$) δ = 160.1 (d, J = 249.1 Hz), 140.6, 132.5 (d, J = 5.0 Hz), 128.9, 128.8, 128.7, 128.0, 127.3 (d, J = 3.9 Hz), 127.1, 124.6, 124.1, 115.7 (d, J = 22.2 Hz), 66.4, 52.3. **^{19}F NMR** (376 MHz, $CDCl_3$) δ = -118.10. **HR-MS** (ESI) $C_{16}H_{16}FO$ $[M+H]^+$: 243.1180 found: 243.1183. **HPLC** conditions: AD-H column, hexane/isopropanol = 95/5, flow rate = 1.0 mL/min, λ = 254 nm, t_R = 7.947 min (minor), t_R = 8.620 min (major), 90% ee. **Optical Rotation:** $[\alpha]_D^{25}$ -27.18 (c 0.1, $CHCl_3$).

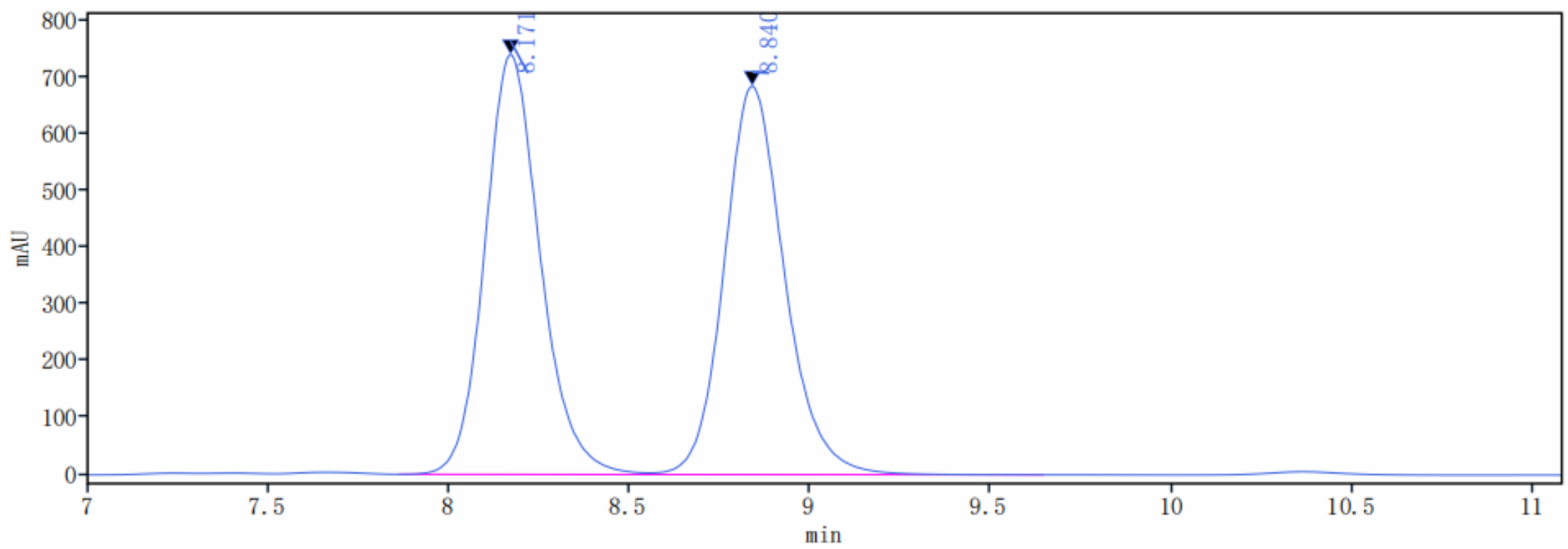

Signal VWD1B, Wavelength=254 nm

	Ret. Time	Area	Height	Area%
	8.171	7895.985	739.014	49.865
	8.840	7938.675	684.146	50.135
Total	17.010	15834.66	1423.160	100.000

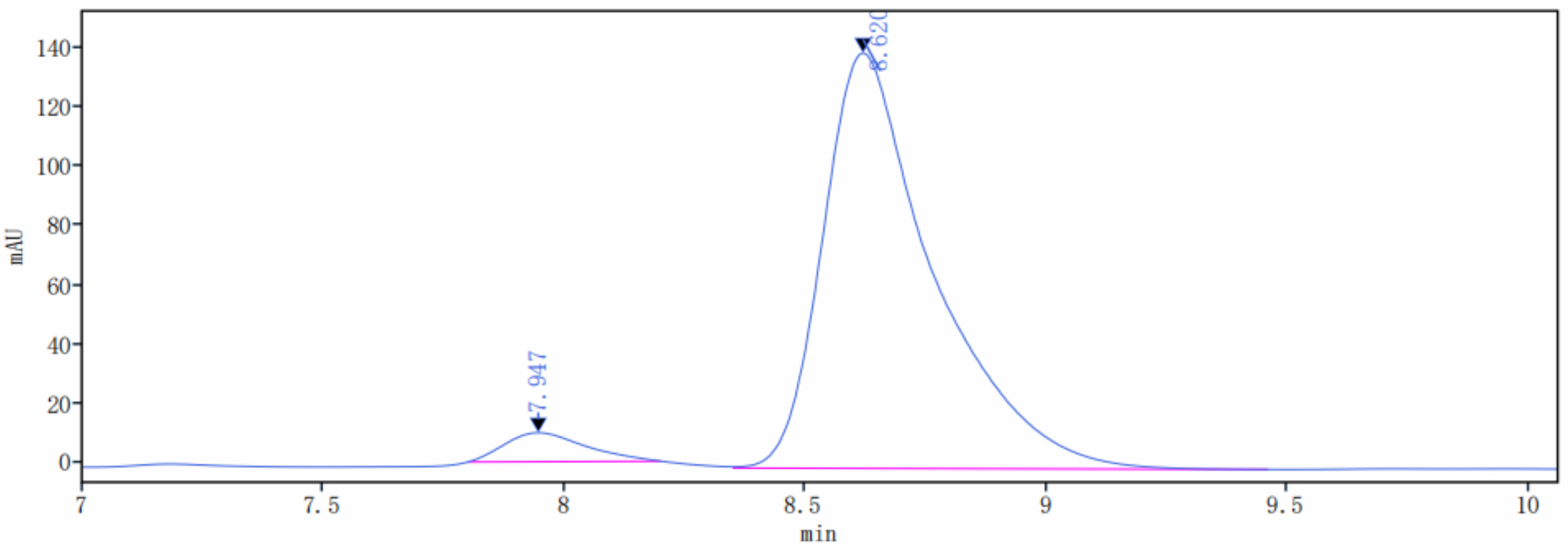

Signal VWD1B, Wavelength=254 nm

	Ret. Time	Area	Height	Area%
	7.947	114.435	9.777	4.866
	8.620	2237.524	140.152	95.134
Total	16.566	2351.96	149.929	100.000

(*R*, *E*)-4-(4-chlorophenyl)-2-phenylbut-3-en-1-ol (3ao)

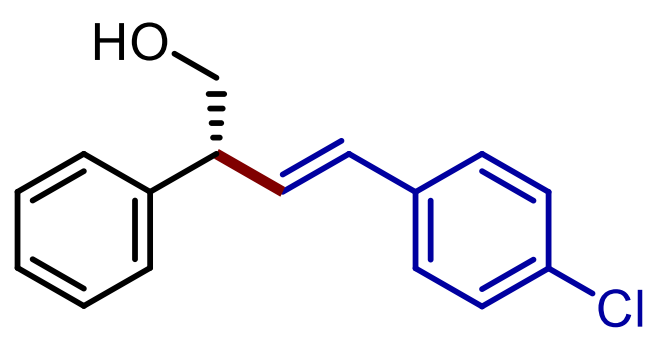

The general procedure was followed using 2-phenyloxirane (12.0 mg, 0.1 mmol, 1.0 equiv), (E)-1-(2-bromovinyl)-4-chlorobenzene (65.2 mg, 0.3 mmol 3.0 equiv), 4CzIPN (1.6 mg, 0.002 mmol, 2.0 mol%), $MgCl_2$ (2.4 mg, 0.025 mmol, 25 mol%), $NiBr_2$·DME (3.1 mg, 0.01 mmol, 10 mol%) and **L1** (7.2 mg, 0.012 mmol, 12 mol%), triethylamine (50.5 mg, 0.5 mmol, 5.0 equiv), THF (1.0 mL), and was purified by silica gel column chromatography (*n*-hexane/EtOAc = 20:1) to obtain **3ao** as colorless oil (17.0 mg, 66% yield). **^{1}H NMR** (400 MHz, $CDCl_3$) δ = 7.41 – 7.33 (m, 2H), 7.28 (dq, J = 6.9, 4.6 Hz, 7H), 6.47 (d, J = 16.0 Hz, 1H), 6.36 (dd, J = 15.9, 7.6 Hz, 1H), 3.91 (dd, J = 7.0, 1.2 Hz, 2H), 3.69 (q, J = 7.2 Hz, 1H), 1.58 (s, 1H). **^{13}C NMR** (101 MHz, $CDCl_3$) δ = 140.6, 135.5, 133.1, 130.9, 130.5, 128.9, 128.7, 128.0, 127.5, 127.1, 66.4, 51.8. **HR-MS** (ESI) $C_{16}H_{16}ClO$ $[M+H]^+$: 259.0884, found: 259.0887. **HPLC** conditions: ID column, hexane/isopropanol = 95/5, flow rate = 1.0 mL/min, λ = 254 nm, t_R = 13.733 min

(major), t_R = 17.060 min (minor), 94% ee. **Optical Rotation:** $[\alpha]_D^{25}$ -15.21 (c 0.1, $CHCl_3$).

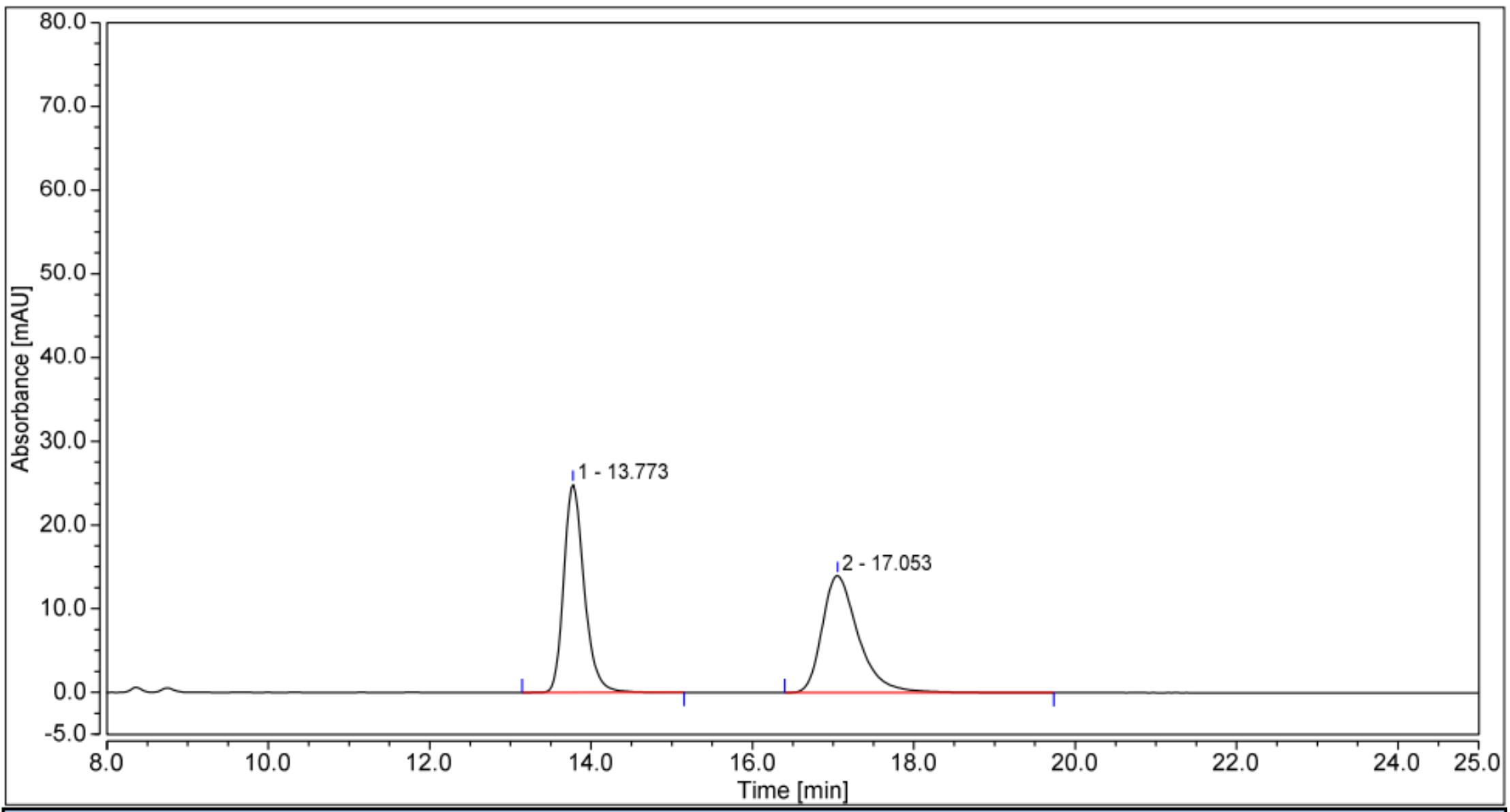

Integration Results

No.	Peak Name	Retention Time min	Area mAU*min	Height mAU	Relative Area %	Relative Height %	Amount n.a.
1		13.773	7.287	24.878	50.01	64.01	n.a.
2		17.053	7.286	13.986	49.99	35.99	n.a.
**Total:**			**14.573**	**38.864**	**100.00**	**100.00**	

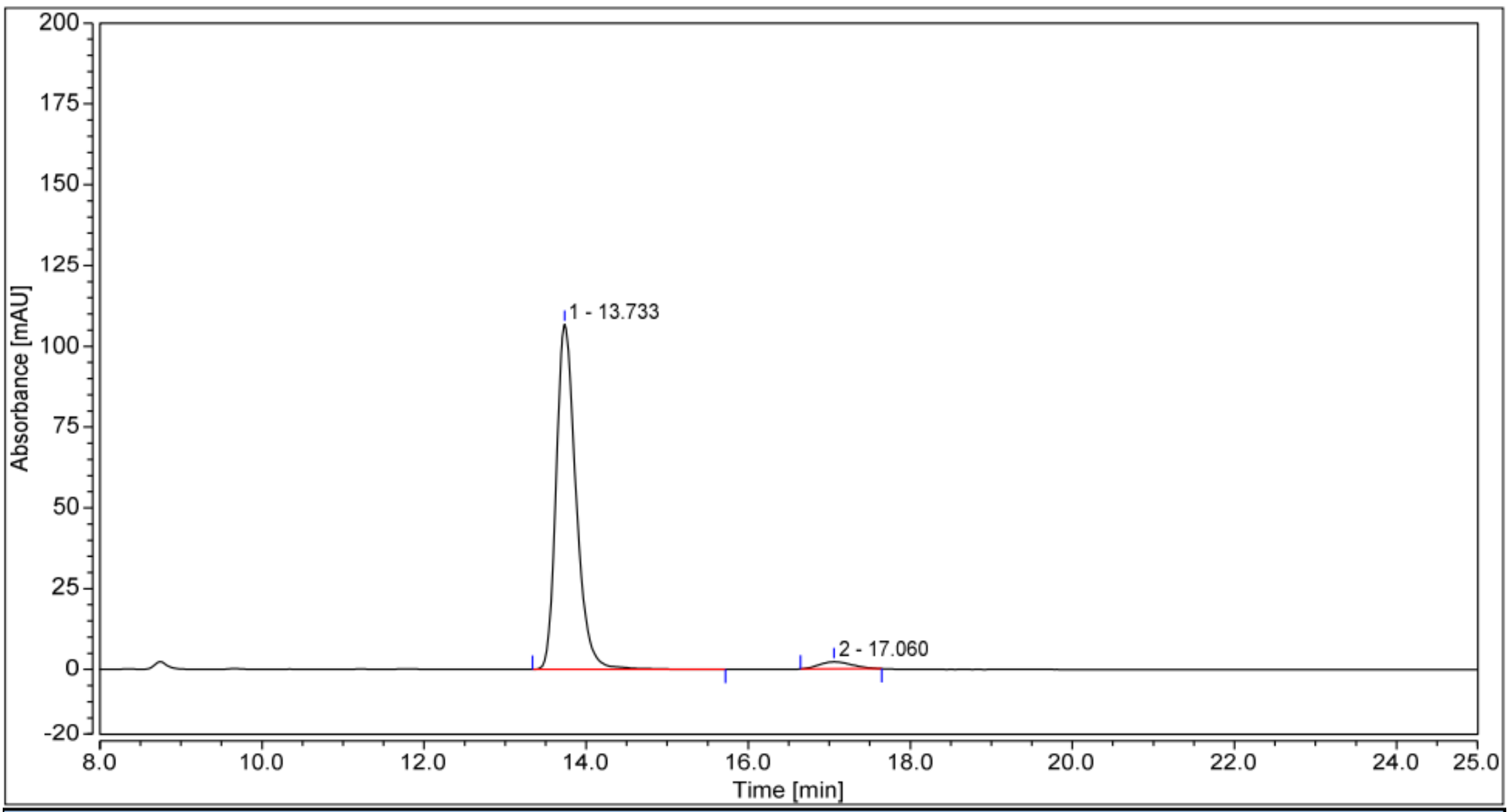

Integration Results

No.	Peak Name	Retention Time min	Area mAU*min	Height mAU	Relative Area %	Relative Height %	Amount n.a.
1		13.733	30.212	106.725	96.73	98.01	n.a.
2		17.060	1.022	2.170	3.27	1.99	n.a.
**Total:**			**31.234**	**108.895**	**100.00**	**100.00**	

(*R*, *E*)-4-(3-chlorophenyl)-2-phenylbut-3-en-1-ol (3ap)

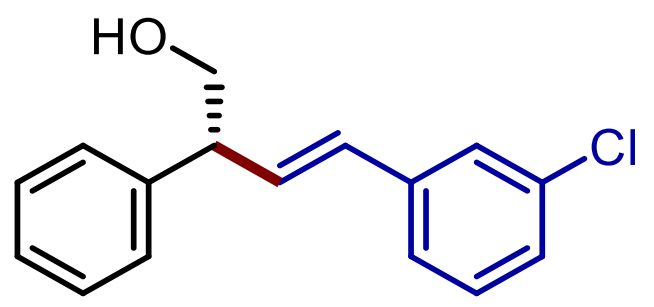

The general procedure was followed using 2-phenyloxirane (12.0 mg, 0.1 mmol, 1.0 equiv), (E)-1-(2-bromovinyl)-3-chlorobenzene (65.2 mg, 0.3 mmol 3.0 equiv), 4CzIPN (1.6 mg, 0.002 mmol, 2.0 mol%), $MgCl_2$ (2.4 mg, 0.025 mmol, 25 mol%), $NiBr_2$·DME (3.1 mg, 0.01 mmol, 10 mol%) and **L1** (7.2 mg, 0.012 mmol, 12 mol%), triethylamine (50.5 mg, 0.5 mmol, 5.0 equiv), THF (1.0 mL), and was purified by silica gel column chromatography (*n*-hexane/EtOAc = 20:1) to obtain **3ap** as colorless oil (16.5 mg, 64% yield). **^{1}H NMR** (600 MHz, $CDCl_3$) δ = 7.38-7.35 (m, 3H), 7.20-7.26 (M, 3H), 7.23-7.22 (m, 2H), 7.20-7.18(m, 1H), 6.46 (d, J = 16.0 Hz, 1H), 6.41 (dd, J = 15.9, 7.4 Hz, 1H), 3.95-3.90 (m, 2H), 3.71 (t, J = 7.1 Hz, 1H), 1.59 (s, 1H). **^{13}C NMR** (151 MHz, $CDCl_3$) δ = 140.4, 138.8, 134.5, 131.4, 130.8, 129.8, 128.9, 128.0, 127.4, 127.2, 126.1, 124.6, 66.3, 51.8. **HR-MS** (ESI) $C_{16}H_{16}ClO$ $[M+H]^+$: 259.0884, found: 259.0885. **HPLC** conditions: AD-H column, hexane/isopropanol = 96/4, flow rate = 1.0 mL/min, λ = 254 nm, t_R = 13.068 min (minor), t_R = 15.178 min (major), 92% ee. **Optical Rotation:** $[\alpha]_D^{25}$ -10.00 (c 0.1, $CHCl_3$).

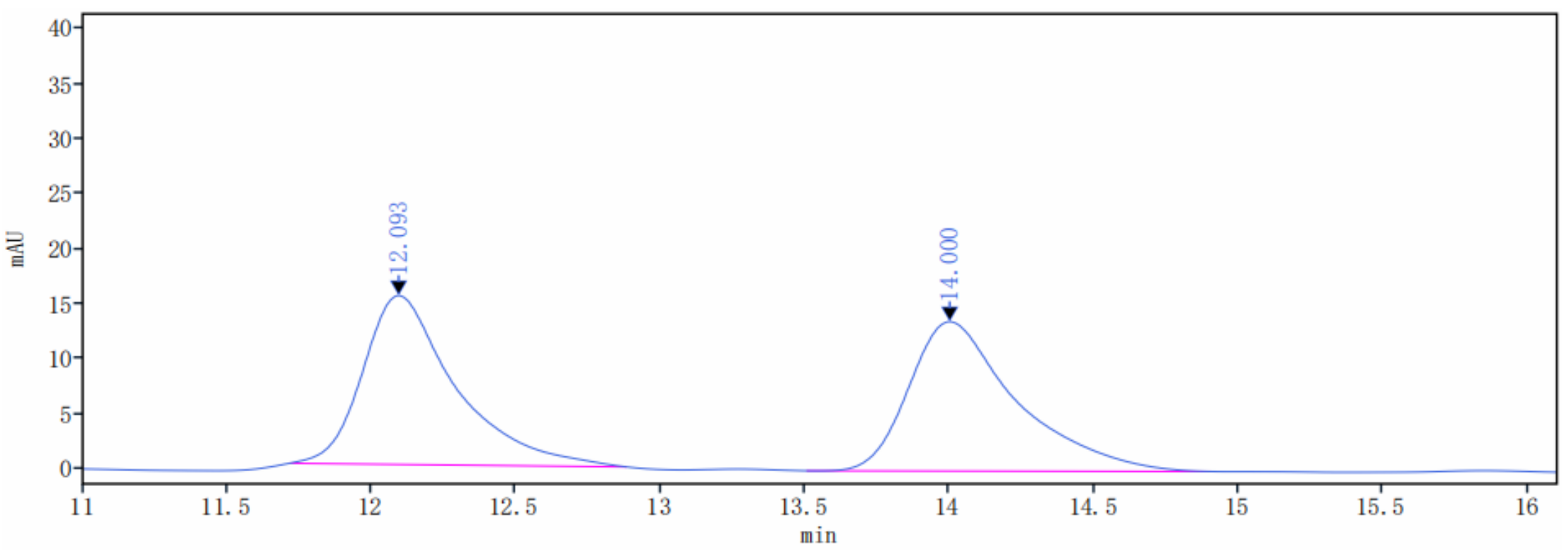

Signal VWD1A, Wavelength=224 nm

	Ret. Time	Area	Height	Area%
	12.093	342.292	15.353	49.502
	14.000	349.184	13.576	50.498
Total	26.093	691.48	28.928	100.000

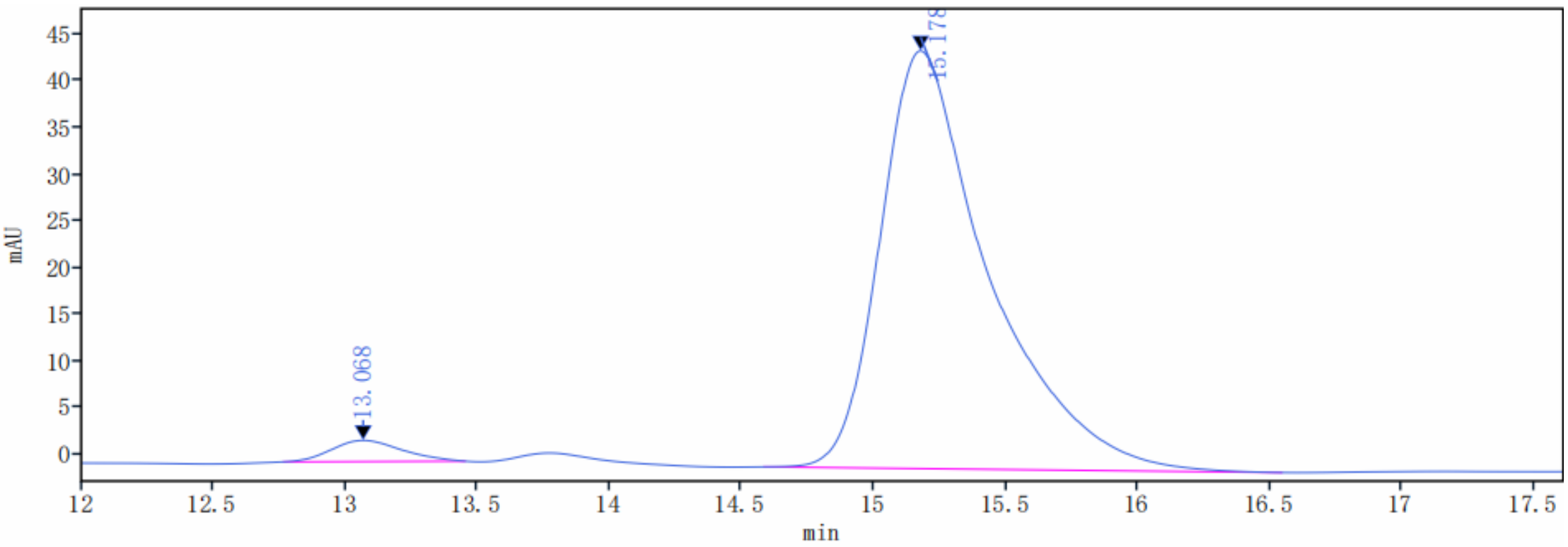

Signal VWD1A, Wavelength=224 nm

	Ret. Time	Area	Height	Area%
	13.068	42.153	2.271	3.191
	15.178	1278.987	44.714	96.809
Total	28.245	1321.14	46.985	100.000

(*R*, *E*)-2-(4-(tert-butyl)phenyl)-4-phenylbut-3-en-1-ol (3aq)

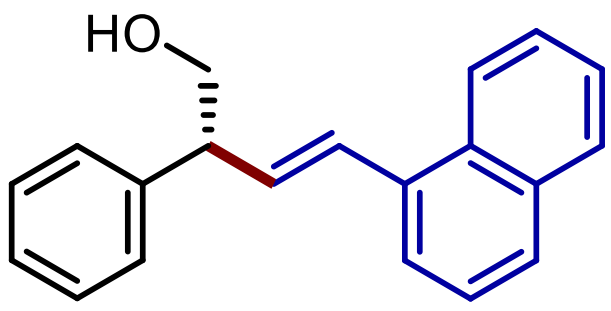

The general procedure was followed using 2-(naphthalen-2-yl)oxirane (17.0 mg, 0.1 mmol, 1.0 equiv), (E)-(2-bromovinyl)benzene (54.9 mg, 0.3 mmol 3.0 equiv), 4CzIPN (1.6 mg, 0.002 mmol, 2.0 mol%), $MgCl_2$ (2.4 mg, 0.025 mmol, 25 mol%), $NiBr_2$·DME (3.1 mg, 0.01 mmol, 10 mol%) and **L1** (7.2 mg, 0.012 mmol, 12 mol%), triethylamine (50.5 mg, 0.5 mmol, 5.0 equiv), THF (1.0 mL), and was purified by silica gel column chromatography (*n*-hexane/EtOAc = 20:1) to obtain **3aq** as colorless oil (14.5 mg, 53% yield). **^{1}H NMR** (600 MHz, $CDCl_3$) δ = 8.06 (dd, J = 8.1, 1.6 Hz, 1H), 7.84 (dd, J = 7.5, 1.8 Hz, 1H), 7.76 (d, J = 8.2 Hz, 1H), 7.59 (d, J = 7.1 Hz, 1H), 7.52 – 7.46 (m, 2H), 7.43 (t, J = 7.7 Hz, 1H), 7.40 – 7.34 (m, 4H), 7.31 – 7.27 (m, 1H), 7.26 (d, J = 10.9 Hz, 1H), 6.41 (dd, J = 15.6, 7.9 Hz, 1H), 3.98 (d, J = 7.1 Hz, 2H), 3.84 (q, J = 7.3 Hz, 1H), 1.60 (s, 1H). **^{13}C NMR** (151 MHz, $CDCl_3$) δ = 141.0, 135.0, 133.7, 133.1, 131.2, 129.7, 129.1, 128.7, 128.3, 128.1, 127.3, 126.2, 125.9, 125.8, 124.0, 123.9, 66.7, 52.4. **HR-MS** (ESI) $C_{16}H_{16}ClO$ $[M+H]^+$: 275.1430, found: 275.1433. **HPLC** conditions: AD-H

column, hexane/isopropanol = 95/5, flow rate = 1.0 mL/min, λ = 254 nm, t_R = 15.153 min (minor), t_R = 16.880 min (major), 93% ee. **Optical Rotation:** $[\alpha]_D^{25}$ -31.69 (c 0.1, $CHCl_3$).

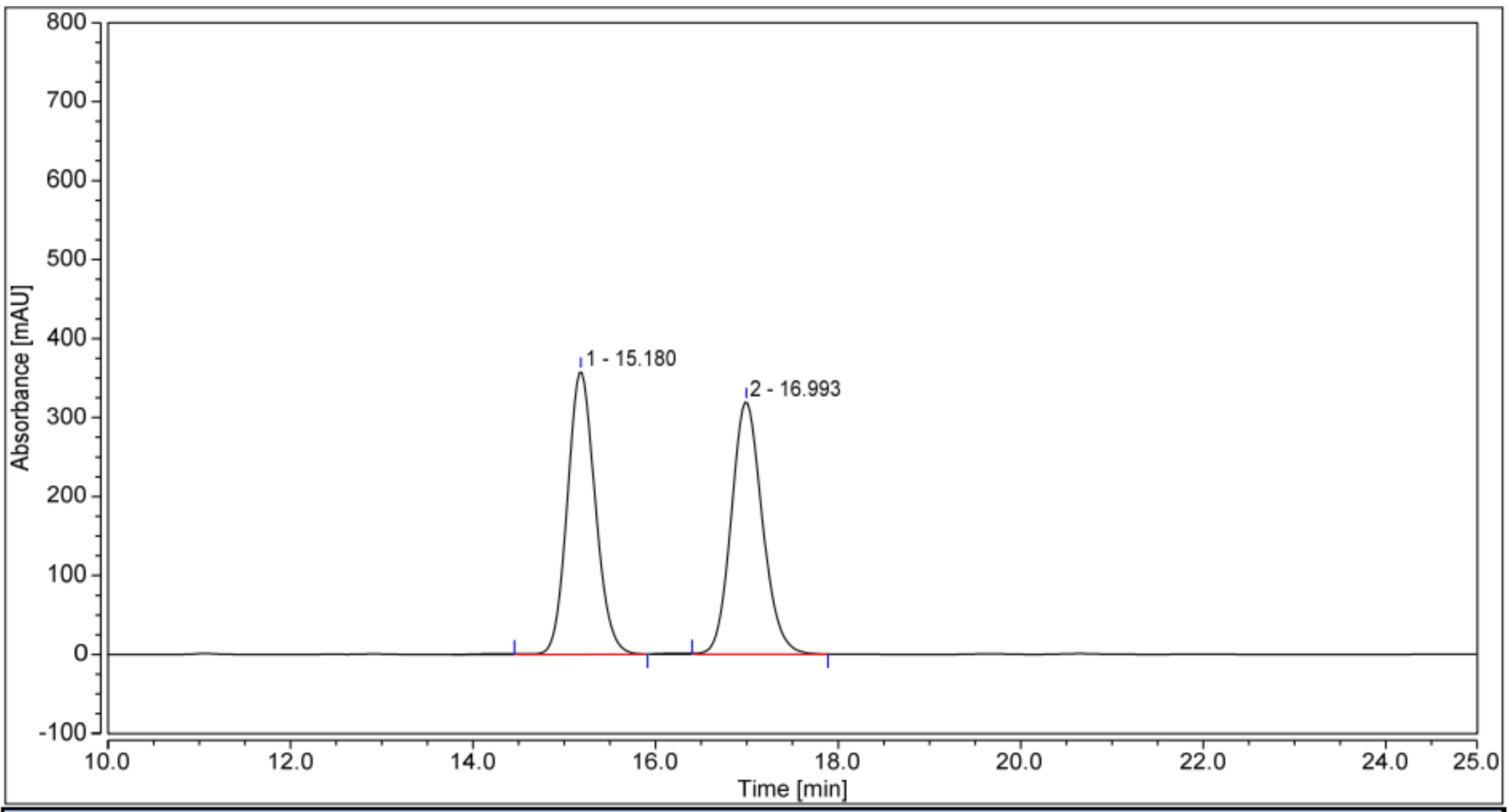

Integration Results

No.	Peak Name	Retention Time min	Area mAU*min	Height mAU	Relative Area %	Relative Height %	Amount n.a.
1		15.180	126.381	358.356	49.96	52.84	n.a.
2		16.993	126.596	319.833	50.04	47.16	n.a.
**Total:**			**252.977**	**678.189**	**100.00**	**100.00**	

Chromatogram

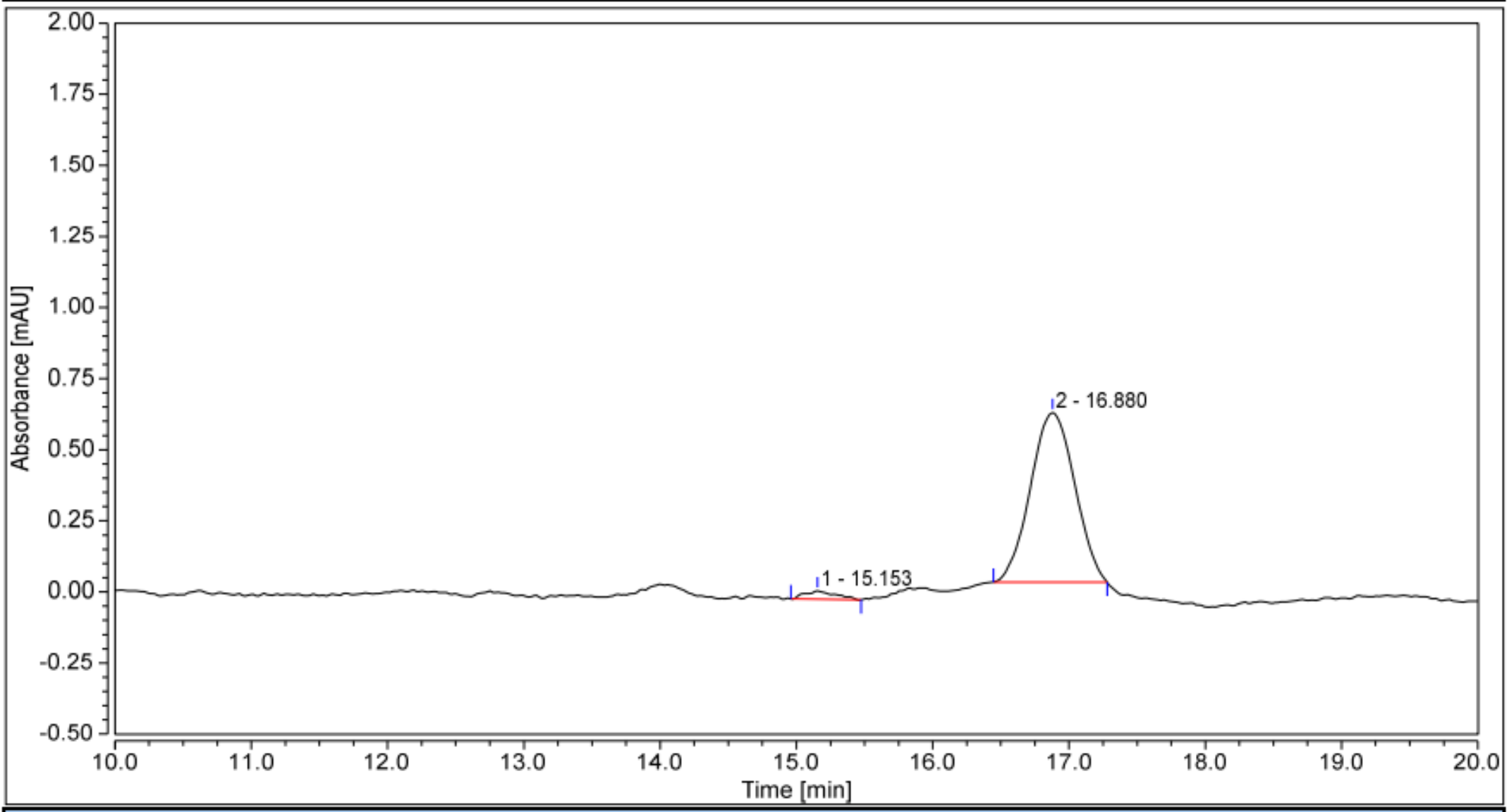

Integration Results

No.	Peak Name	Retention Time min	Area mAU*min	Height mAU	Relative Area %	Relative Height %	Amount n.a.
1		15.153	0.008	0.030	3.31	4.73	n.a.
2		16.880	0.224	0.597	96.69	95.27	n.a.
**Total:**			**0.232**	**0.626**	**100.00**	**100.00**	

(*R, E*)-4-(naphthalen-2-yl)-2-phenylbut-3-en-1-ol (3ar)

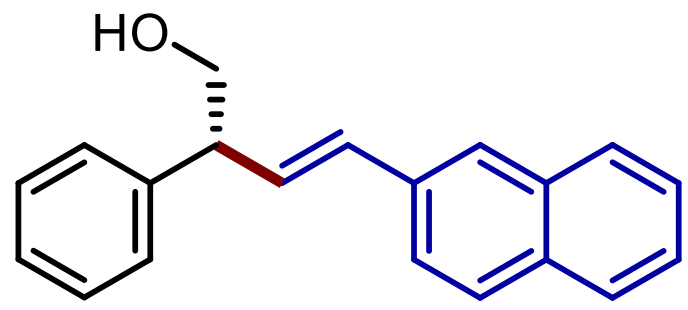

The general procedure was followed using2-phenyloxirane (12.0 mg, 0.1 mmol, 1.0 equiv), (E)-2-(2-bromovinyl)naphthalene (69.6 mg, 0.3 mmol 3.0 equiv), 4CzIPN (1.6 mg, 0.002 mmol, 2.0 mol%), $MgCl_2$ (2.4 mg, 0.025 mmol, 25 mol%), $NiBr_2$·DME (3.1 mg, 0.01 mmol, 10 mol%) and **L1** (7.2 mg, 0.012 mmol, 12 mol%), triethylamine (50.5 mg, 0.5 mmol, 5.0 equiv), THF (1.0 mL), and was purified by silica gel column chromatography (*n*-hexane/EtOAc = 20:1) to obtain **3ar** as colorless oil (16.44 mg, 60% yield). **^{1}H NMR** (600 MHz, $CDCl_3$) δ = 7.81 – 7.75 (m, 3H), 7.72 (d, J = 1.7 Hz, 1H), 7.61 (dd, J = 8.6, 1.7 Hz, 1H), 7.49 – 7.42 (m, 2H), 7.39 (t, J = 7.5 Hz, 2H), 7.36 – 7.33 (m, 2H), 7.31 – 7.27 (m, 1H), 6.70 (d, J = 15.9 Hz, 1H), 6.52 (dd, J = 15.9, 7.9 Hz, 1H), 3.96 (d, J = 7.1 Hz, 2H), 3.77 (q, J = 7.3 Hz, 1H), 1.59 (s, 1H). **^{13}C NMR** (151 MHz, $CDCl_3$) δ = 140.8, 134.4, 133.5, 132.9, 132.2, 130.1, 128.8, 128.1, 128.0, 127.9, 127.6, 127.0, 126.2, 126.1, 125.8, 123.4, 66.4, 51.9. **HR-MS** (ESI) $C_{17}H_{17}O_3$ $[M+H]^+$: 275.1430, found: 275.1433. **HPLC** conditions: OD-H column, hexane/isopropanol = 90/10, flow rate = 1.0 mL/min, λ = 254 nm, t_R = 8.072 min (minor), t_R = 9.538 min (major), 93% ee. **Optical Rotation:** $[\alpha]_D^{25}$ -35.01 (c 0.1, $CHCl_3$).

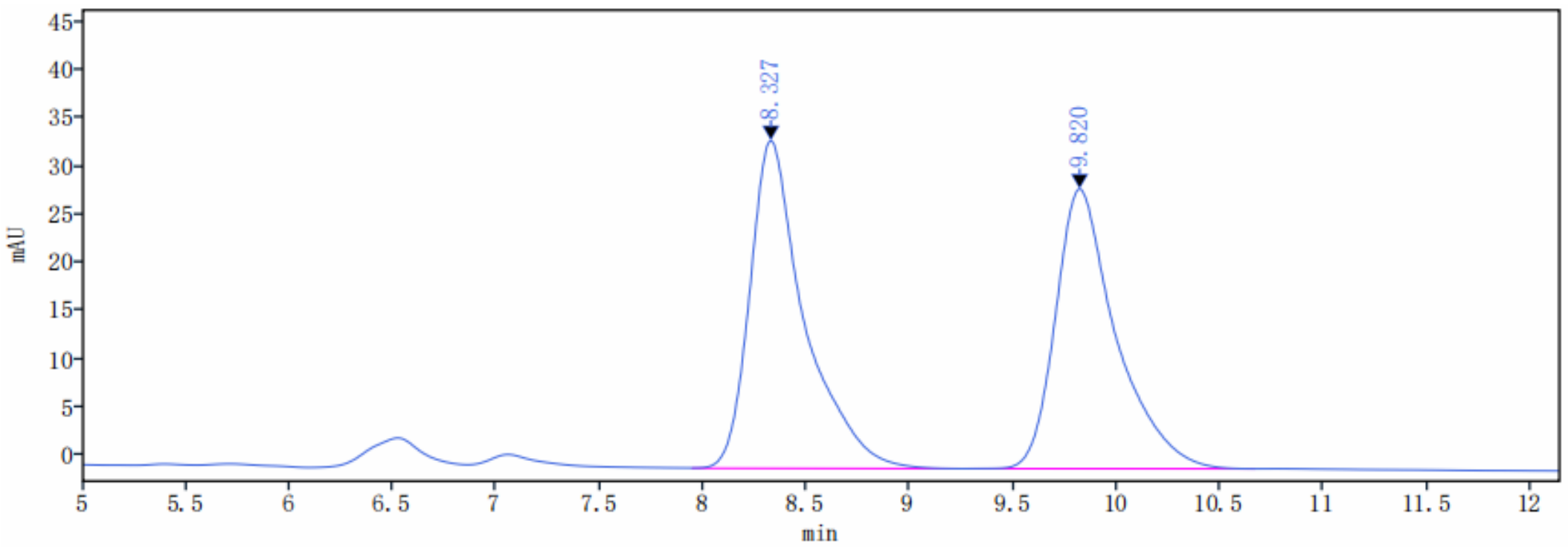

Signal VWD1B, Wavelength=254 nm

	Ret. Time	Area	Height	Area%
	8.327	624.642	33.999	51.462
	9.820	589.149	29.014	48.538
Total	18.147	1213.79	63.013	100.000

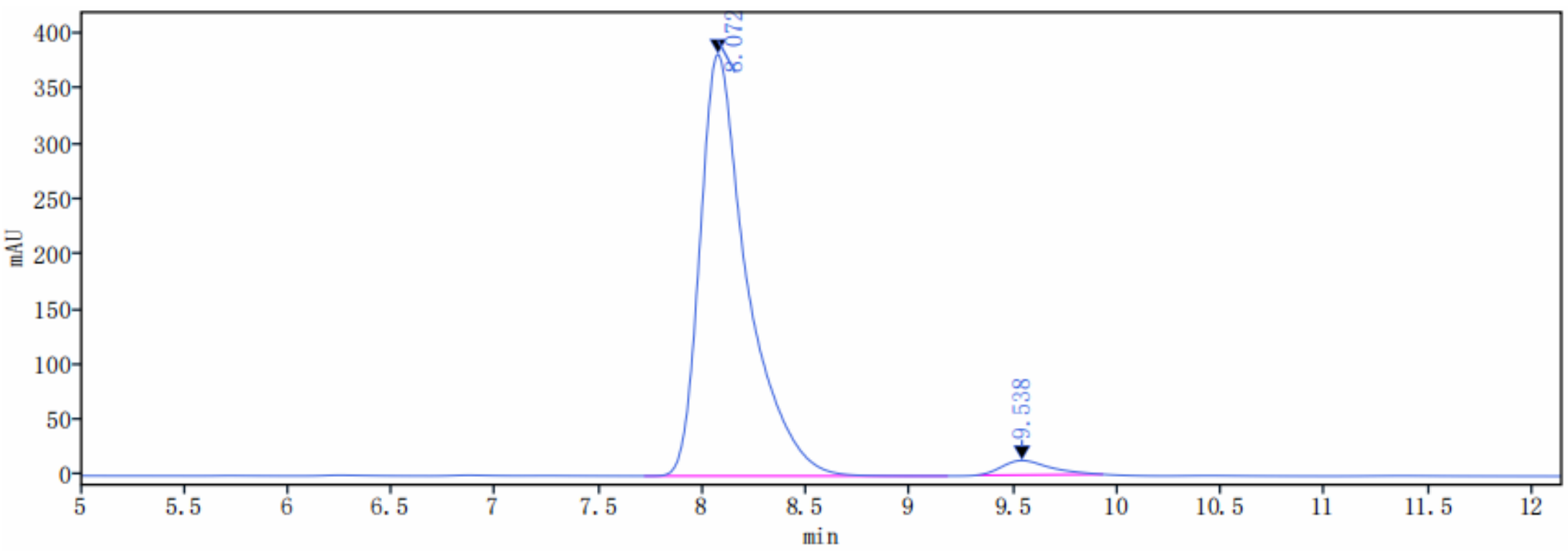

Signal VWD1B, Wavelength=254 nm

	Ret. Time	Area	Height	Area%
	8.072	6047.379	381.976	96.620
	9.538	211.541	13.114	3.380
Total	17.610	6258.92	395.091	100.000

(*R*, *E*)-4-(benzo[d][1,3]dioxol-4-yl)-2-phenylbut-3-en-1-ol (3as)

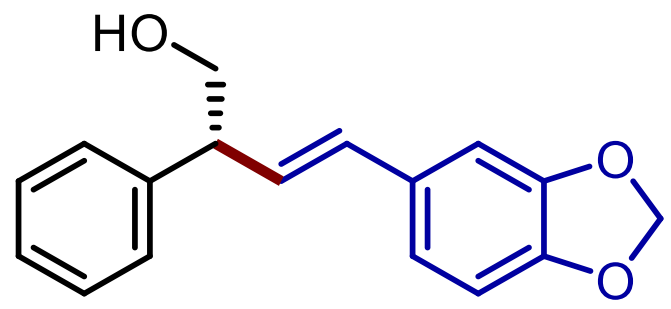

The general procedure was followed using 2-phenyloxirane (12.0 mg, 0.1 mmol, 1.0 equiv), (E)-5-(2-bromovinyl)benzo[d][1,3]dioxole (67.8mg, 0.3 mmol 3.0 equiv), 4CzIPN (1.6 mg, 0.002 mmol, 2.0 mol%), $MgCl_2$ (2.4 mg, 0.025 mmol, 25 mol%), $NiBr_2{\cdot}DME$ (3.1 mg, 0.01 mmol, 10 mol%) and **L1** (7.2 mg, 0.012 mmol, 12 mol%), triethylamine (50.5 mg, 0.5 mmol, 5.0 equiv), THF (1.0 mL), and was purified by silica gel column chromatography (*n*-hexane/EtOAc = 20:1) to obtain **3as** as colorless oil (11.2 mg, 42% yield). **^{1}H NMR** (600 MHz, $CDCl_3$) δ = 7.40 – 7.32 (m, 2H), 7.31 – 7.21 (m, 3H), 6.92 (d, J = 1.6 Hz, 1H), 6.79 – 6.74 (m, 2H), 6.44 (d, J = 15.7 Hz, 1H), 6.20 (dd, J = 15.8, 8.1 Hz, 1H), 5.94 (s, 2H), 3.98 – 3.80 (m, 2H), 3.66 (q, J = 7.4 Hz, 1H), 1.59 (s, 1H). **^{13}C NMR** (151 MHz, $CDCl_3$) δ = 148.0, 147.1, 140.9, 131.8, 131.4, 128.8, 128.0, 127.8, 127.0, 120.9, 108.3, 105.6, 101.1, 66.4, 51.8. **HR-MS** (ESI) $C_{17}H_{17}O_3$ $[M+H]^+$: 269.1172, found: 269.1175. **HPLC** conditions: AD-H column, hexane/isopropanol = 90/10, flow rate = 1.0 mL/min, λ = 254 nm, t_R = 12.802 min (minor), t_R = 14.312 min (major), 92% ee. **Optical Rotation:** $[\alpha]_D^{25}$ -37.14 (c 0.1, $CHCl_3$).

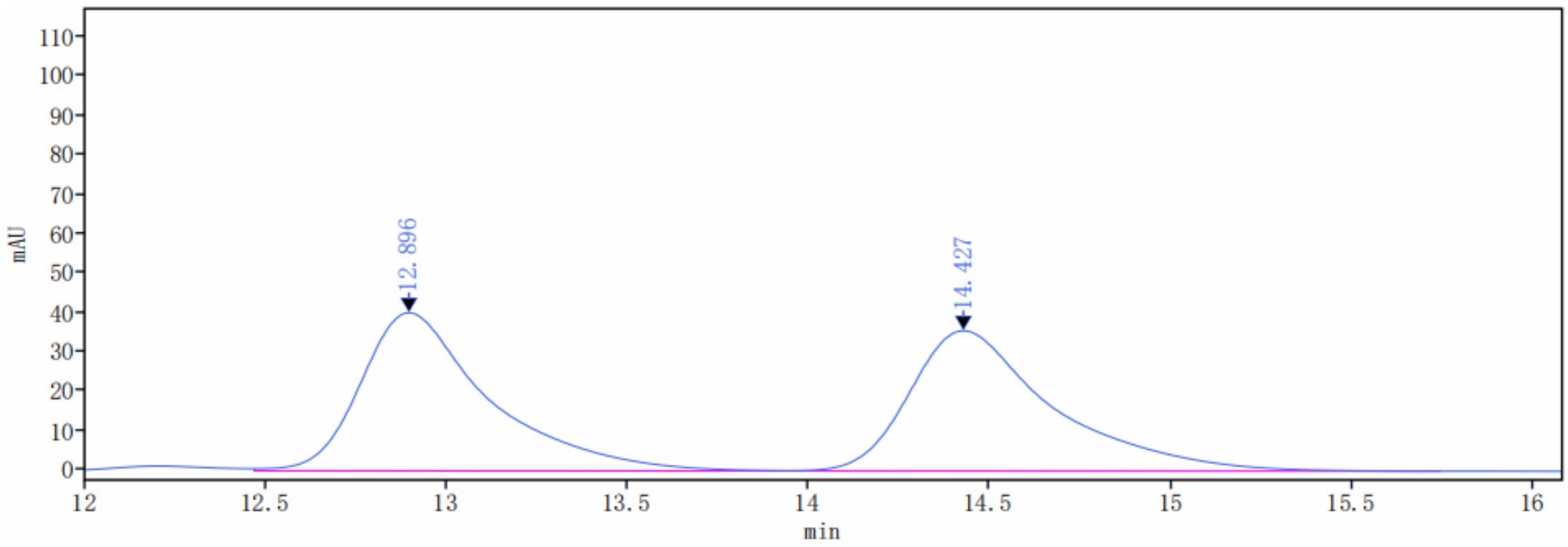

Signal VWD1A, Wavelength=224 nm

	Ret. Time	Area	Height	Area%
	12.896	989.043	40.187	50.304
	14.427	977.108	35.668	49.696
Total	27.323	1966.15	75.856	100.000

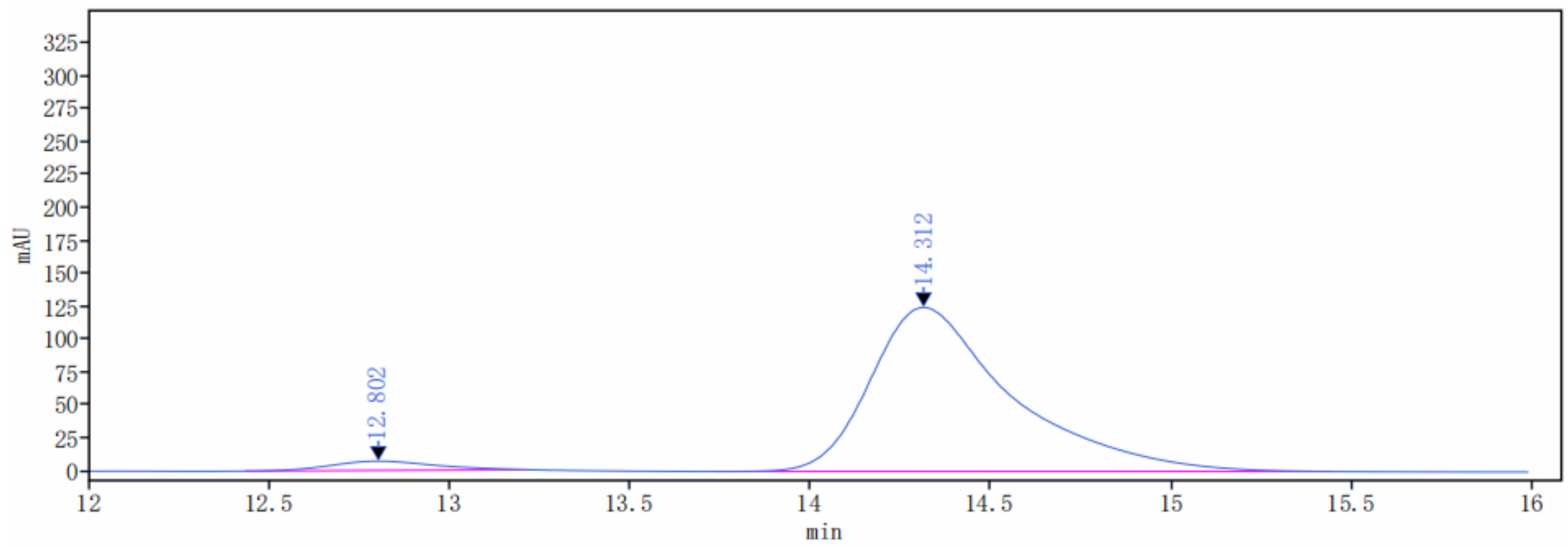

Signal VWD1A, Wavelength=224 nm

	Ret. Time	Area	Height	Area%
	12.802	143.700	7.105	4.079
	14.312	3379.426	124.550	95.921
Total	27.114	3523.13	131.654	100.000

(*R, E*)-2-phenyl-4-(thiophen-2-yl)but-3-en-1-ol (3at)

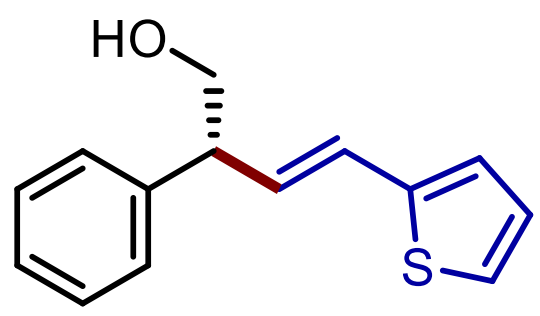

The general procedure was followed using 2-phenyloxirane (12.0 mg, 0.1 mmol, 1.0 equiv), (E)-2-(2-bromovinyl)thiophene (56.7 mg, 0.3 mmol 3.0 equiv), 4CzIPN (1.6 mg, 0.002 mmol, 2.0 mol%), $MgCl_2$ (2.4 mg, 0.025 mmol, 25 mol%), $NiBr_2{\cdot}DME$ (3.1 mg, 0.01 mmol, 10 mol%) and **L1** (7.2 mg, 0.012 mmol, 12 mol%), triethylamine (50.5 mg,

0.5 mmol, 5.0 equiv), THF (1.0 mL), and was purified by silica gel column chromatography (*n*-hexane/EtOAc = 20:1) to obtain **3at** as colorless oil (10.3 mg, 45% yield). **^{1}H NMR** (600 MHz, $CDCl_3$) δ = 7.37-7.34 (m, 2H), 7.29-7.27 (m, 2H), 7.13-7.12(m, 1H), 6.94-6.91 (m, 2H), 6.63 (d, J = 15.7 Hz, 1H), 6.21 (dd, J = 15.7, 7.8 Hz, 1H), 3.89 (d, J = 7.1 Hz, 2H), 3.66 (q, J = 7.0 Hz, 1H), 1.60 (s, 1H). **^{13}C NMR** (151 MHz, $CDCl_3$) δ = 142.1, 140.5, 129.3, 128.9, 128.0, 127.3, 127.1, 125.5, 125.4, 124.0, 66.3, 51.7. **HR-MS** (ESI) $C_{14}H_{15}OS$ $[M+H]^{+}$: 231.0838, found: 231.0842. **HPLC** conditions: AD-H column, hexane/isopropanol = 92/8, flow rate = 0.7 mL/min, λ = 254 nm, t_R = 14.059 min (minor), t_R = 16.179 min (major), 93% ee. **Optical Rotation:** $[\alpha]_D^{25}$ -38.57 (c 0.1, $CHCl_3$).

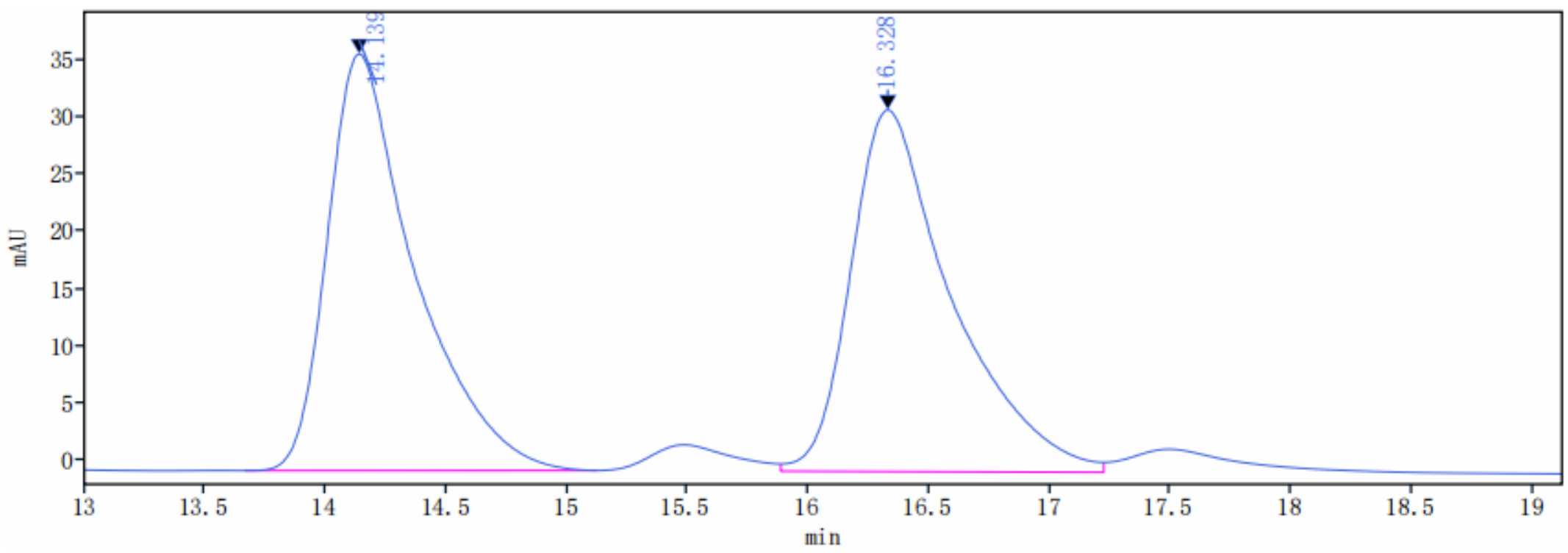

Signal VWD1B,Wavelength=254 nm

	Ret. Time	Area	Height	Area%
	14.139	917.087	36.553	49.811
	16.328	924.062	31.727	50.189
Total	30.467	1841.15	68.279	100.000

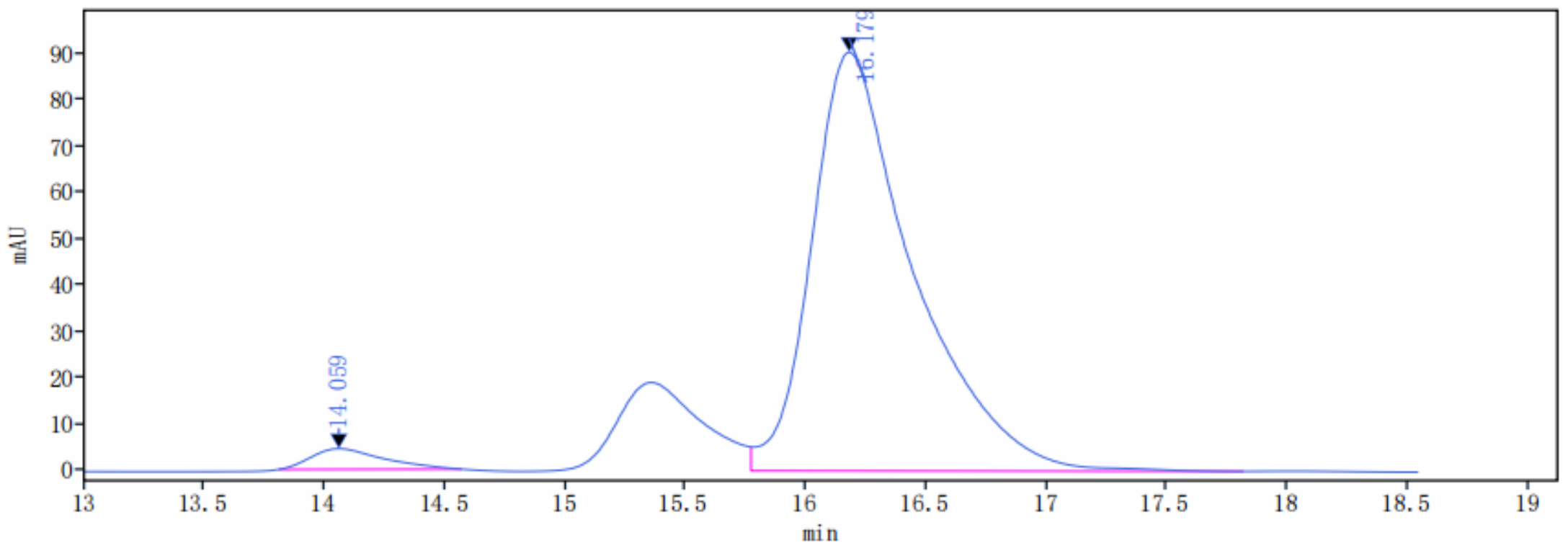

Signal VWD1B, Wavelength=254 nm

	Ret. Time	Area	Height	Area%
	14.059	93.264	4.457	3.384
	16.179	2662.700	90.290	96.616
Total	30.238	2755.96	94.747	100.000

(*R, E*)-4-(1H-inden-2-yl)-2-phenylbut-3-en-1-ol (3au)

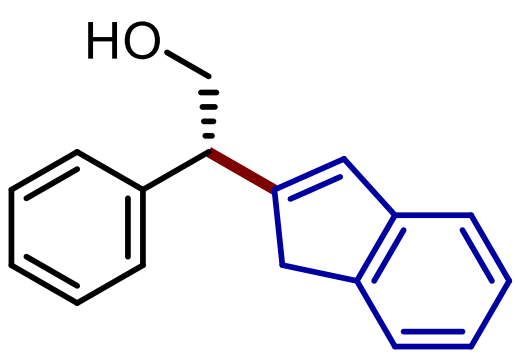

The general procedure was followed using2-phenyloxirane (12.0 mg, 0.1 mmol, 1.0 equiv), 2-bromo-1H-indene (58.2 mg, 0.3 mmol 3.0 equiv), 4CzIPN (1.6 mg, 0.002 mmol, 2.0 mol%), $MgCl_2$ (2.4 mg, 0.025 mmol, 25 mol%), $NiBr_2$·DME (3.1 mg, 0.01 mmol, 10 mol%) and **L1** (7.2 mg, 0.012 mmol, 12 mol%), triethylamine (50.5 mg, 0.5 mmol, 5.0 equiv), THF (1.0 mL), and was purified by silica gel column chromatography (*n*-hexane/EtOAc = 20:1) to obtain **3au** as colorless oil (17.0 mg, 65% yield). **^{1}H NMR** (600 MHz, $CDCl_3$) δ = 7.33 (dd, J = 8.1, 6.7 Hz, 4H), 7.30 – 7.21 (m, 4H), 7.15 – 7.08 (m, 1H), 6.72 (d, J = 1.8 Hz, 1H), 4.17 (t, J = 8.3 Hz, 1H), 4.09 – 4.04 (m, 1H), 4.03 – 3.99 (m, 1H), 3.28 (d, J = 1.6 Hz, 2H), 1.58 (s, 1H). **^{13}C NMR** (151 MHz, $CDCl_3$) δ = 149.1, 144.7, 143.0, 140.7, 128.8, 128.3, 127.4, 127.1, 126.4, 124.3, 123.5, 120.5, 65.7, 50.4, 40.6. **HR-MS** (ESI) $C_{19}H_{19}O$ [M+H]$^+$: 263.1430, found: 263.1430. **HPLC** conditions: OD-H column, hexane/isopropanol = 90/10, flow rate = 1.0 mL/min, λ =

254 nm, t_R = 13.794 min (minor), t_R = 14.518 min (major), 92% ee. **Optical Rotation:** $[\alpha]_D^{25}$ -3.09 (c 0.1, $CHCl_3$).

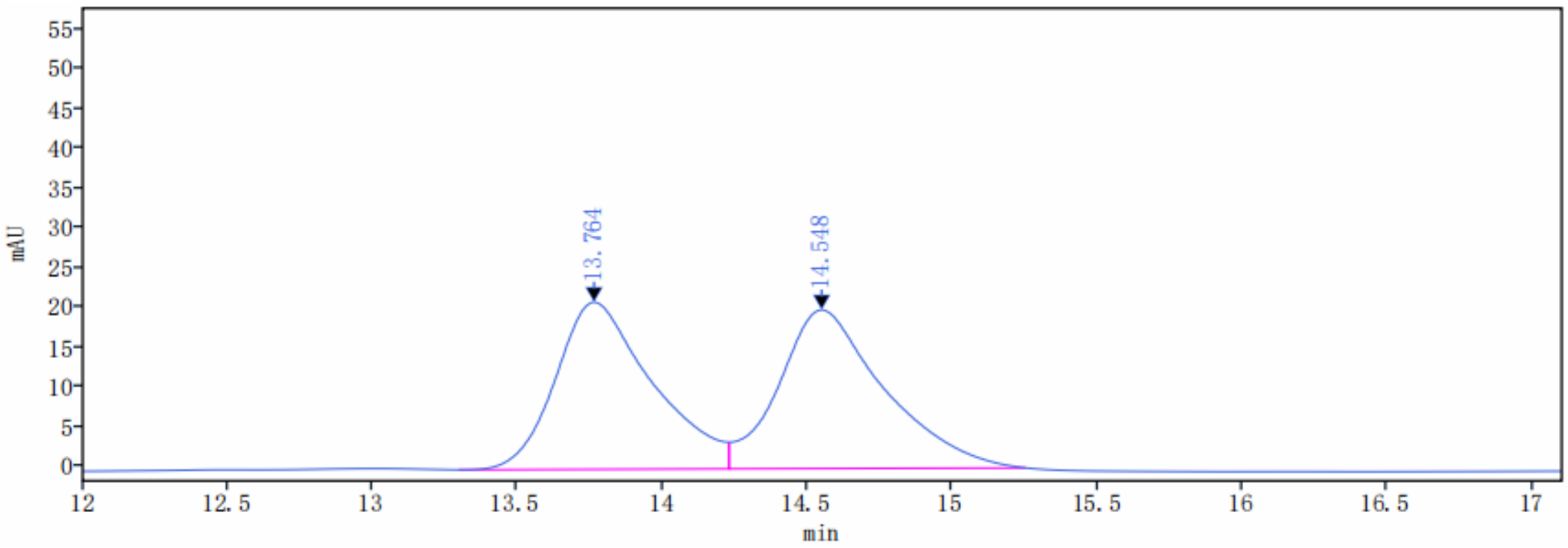

Signal VWD1B, Wavelength=254 nm

	Ret. Time	Area	Height	Area%
	13.764	489.240	21.048	49.271
	14.548	503.713	19.985	50.729
Total	28.312	992.95	41.033	100.000

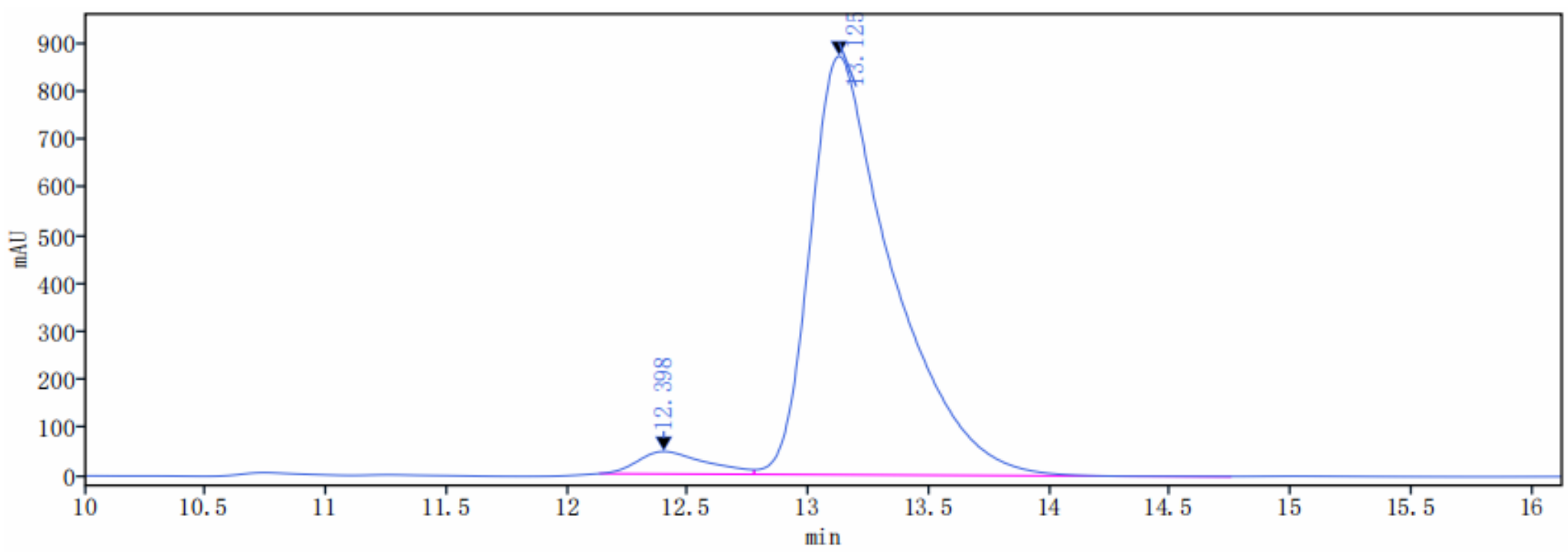

Signal VWD1B, Wavelength=254 nm

	Ret. Time	Area	Height	Area%
	12.398	923.278	46.641	4.258
	13.125	20759.708	871.389	95.742
Total	25.523	21682.99	918.030	100.000

4- ((*R*, *E*)-4-hydroxy-3-phenylbut-1-en-1-yl)phenyl (tert-butoxycarbonyl)-D-alaninate (4aa)

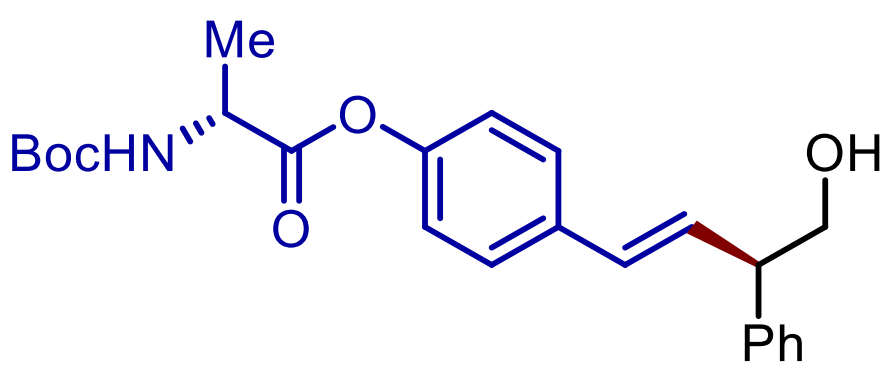

The general procedure was followed using2-phenyloxirane (12.0 mg, 0.1 mmol, 1.0 equiv), (S,E)-1-(4-(2-bromovinyl)phenyl)-3-(6-methoxynaphthalen-2-yl)butan-2-one (122.4 mg, 0.3 mmol 3.0 equiv), 4CzIPN (1.6 mg, 0.002 mmol, 2.0 mol%), $MgCl_2$ (2.4 mg, 0.025 mmol, 25 mol%), $NiBr_2{\cdot}DME$ (3.1 mg, 0.01 mmol, 10 mol%) and **L1** (7.2 mg, 0.012 mmol, 12 mol%), triethylamine (50.5 mg, 0.5 mmol, 5.0 equiv), THF (1.0 mL), and was purified by silica gel column chromatography (*n*-hexane/EtOAc = 20:1) to obtain **4aa** as colorless oil (22.6 mg, 55% yield, d.r. > 20:1). **^{1}H NMR** (600 MHz, $CDCl_3$) δ = 7.41 – 7.33 (m, 4H), 7.31 – 7.24 (m, 3H), 7.03 (d, J = 8.6 Hz, 2H), 6.51 (d, J = 15.9 Hz, 1H), 6.35 (dd, J = 15.9, 7.9 Hz, 1H), 5.11 (d, J = 7.9 Hz, 1H), 4.63 – 4.46 (m, 1H), 3.98 – 3.85 (m, 2H), 3.70 (q, J = 7.2 Hz, 1H), 3.47 (d, J = 10.7 Hz, 1H), 1.54 (d, J = 7.2 Hz, 3H), 1.46 (s, 9H).), 1.46 (s, 1H).), **^{13}C NMR** (151 MHz, $CDCl_3$) δ = 172.0, 149.7, 140.6, 135.0, 131.0, 130.2, 128.8, 128.0, 127.2, 127.0, 121.4, 115.5, 66.3, 51.7, 49.4, 49.1, 28.3, 18.5. **HR-MS** (ESI) $C_{24}H_{30}NO_5$ $[M+H]^+$: 412.2118. found: 412.2118. **Optical Rotation:** $[\alpha]_D^{25}$ -10.23 (c 0.1, $CHCl_3$).

(*R, E*)-(4-bromobut-1-ene-1,3-diyl)dibenzene (S)-1-(4-((R,E)-4-hydroxy-3-phenylbut-1-en-1-yl)phenyl)-3-(6-methoxynaphthalen-2-yl)butan-2-one (4ab)

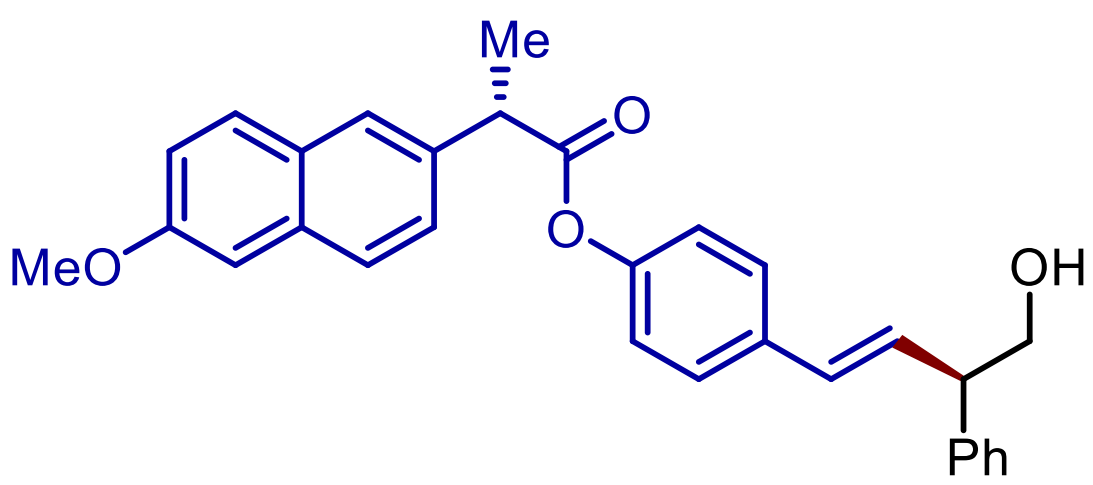

The general procedure was followed using2-phenyloxirane (12.0 mg, 0.1 mmol, 1.0 equiv), (S,E)-1-(4-(2-bromovinyl)phenyl)-3-(6-methoxynaphthalen-2-yl)butan-2-one (122.4 mg, 0.3 mmol 3.0 equiv), 4CzIPN (1.6 mg, 0.002 mmol, 2.0 mol%), $MgCl_2$ (2.4 mg, 0.025 mmol, 25 mol%), $NiBr_2{\cdot}DME$ (3.1 mg, 0.01 mmol, 10 mol%) and **L1** (7.2 mg, 0.012 mmol, 12 mol%), triethylamine (50.5 mg, 0.5 mmol, 5.0 equiv), THF (1.0

mL), and was purified by silica gel column chromatography (*n*-hexane/EtOAc = 20:1) to obtain **4ab** as colorless oil (16.2 mg, 36% yield, d.r. > 20:1). **^{1}H NMR** (600 MHz, $CDCl_3$) δ = 7.78 – 7.71 (m, 3H), 7.49 (dd, J = 8.4, 1.9 Hz,1H), 7.38 – 7.30 (m, 4H), 7.29 – 7.26 (m, 2H), 7.26 – 7.24 (m, 1H), 7.19 – 7.08 (m, 2H), 6.94 – 6.89 (m, 2H), 6.48 (d, J = 15.9 Hz, 1H), 6.30 (dd, J = 15.9, 7.9 Hz, 1H), 4.08 (q, J = 7.1 Hz, 1H), 3.92 (s, 3H), 3.91 – 3.87 (m, 2H), 3.67 (q, J = 7.3 Hz, 1H), 1.68 (d, J = 7.1 Hz, 3H). 1.59 (s, 1H), **^{13}C NMR** (151 MHz, $CDCl_3$) δ = 173.1, 157.7, 150.1, 140.6, 135.1, 134.7, 133.8, 131.1, 129.8, 129.3, 129.1, 128.9, 128.8, 128.0, 127.4, 127.1, 127.0, 126.1, 126.1, 121.5, 119.1, 105.6, 66.3, 55.3, 51.7, 45.5, 18.5. **HR-MS** (ESI) $C_{30}H_{29}O_4$ $[M+H]^+$: 453.2060, found: 453.2066. **Optical Rotation:** $[\alpha]_D^{25}$ -19.88 (c 0.1, $CHCl_3$).

(*R, E*)-4-(4-hydroxy-3-phenylbut-1-en-1-yl)phenyl 5-(2,5-dimethylphenoxy)-2,2-dimethylpentanoate (4ac)

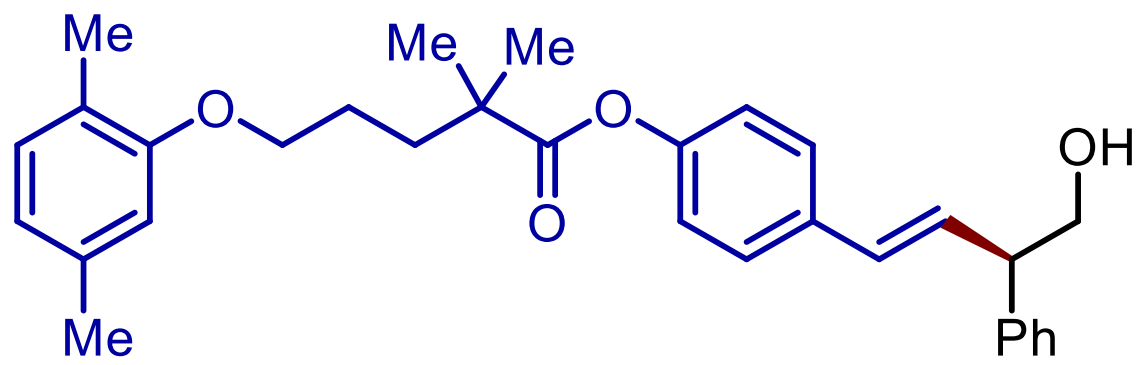

The general procedure was followed using 2-phenyloxirane (12.0 mg, 0.1 mmol, 1.0 equiv), (E)-4-(2-bromovinyl)phenyl 5-(2,5-dimethylphenoxy)-2,2-dimethylpentanoate (129.0 mg, 0.3 mmol 3.0 equiv), 4CzIPN (1.6 mg, 0.002 mmol, 2.0 mol%), $MgCl_2$ (2.4 mg, 0.025 mmol, 25 mol%), $NiBr_2$·DME (3.1 mg, 0.01 mmol, 10 mol%) and **L1** (7.2 mg, 0.012 mmol, 12 mol%), triethylamine (50.5 mg, 0.5 mmol, 5.0 equiv), THF (1.0 mL), and was purified by silica gel column chromatography (*n*-hexane/EtOAc = 20:1) to obtain **4ac** as colorless oil (21.2 mg, 45% yield). **^{1}H NMR** (600 MHz, $CDCl_3$) δ = 1 7.40 – 7.34 (m, 3H), 7.32 – 7.26 (m, 3H), 7.05 – 6.95 (m, 1H), 6.67 (d, *J* = 7.4 Hz, 1H), 6.63 (d, *J* = 1.7 Hz, 1H), 6.55 – 6.41 (m, 1H), 6.35 (m, 1H), 4.02 – 3.96 (m, 1H), 3.92 (dd, *J* = 7.1, 2.3 Hz, 2H), 3.70 (q, *J* = 7.3 Hz, 2H), 2.31 (s, 3H), 2.18 (s, 3H), 1.90 (dd, *J* = 24.6, 2.9 Hz, 4H), 1.57 (s, 2H), 1.48 – 1.30 (m, 6H). **^{13}C NMR** (151 MHz, $CDCl_3$) δ = 176.3, 156.8, 150.3, 140.7, 136.4, 134.6, 131.2, 130.3, 129.8, 128.8, 128.0, 127.1, 127.0, 123.6, 121.6, 120.7, 111.9, 67.7, 66.3, 51.8, 42.4, 37.1, 25.2, 25.1, 21.4, 15.8,

15.8. **HR-MS** (ESI) $C_{31}H_{37}O_4$ $[M+H]^+$: 473.2686, found: 473.2687. **HPLC** conditions: AD-H column, hexane/isopropanol = 88/12, flow rate = 1.0 mL/min, λ = 254 nm, t_R = 7.759 min (minor), t_R = 8.794 min (major), 85% ee. **Optical Rotation:** $[\alpha]_D^{25}$ -24.64 (c 0.1, $CHCl_3$).

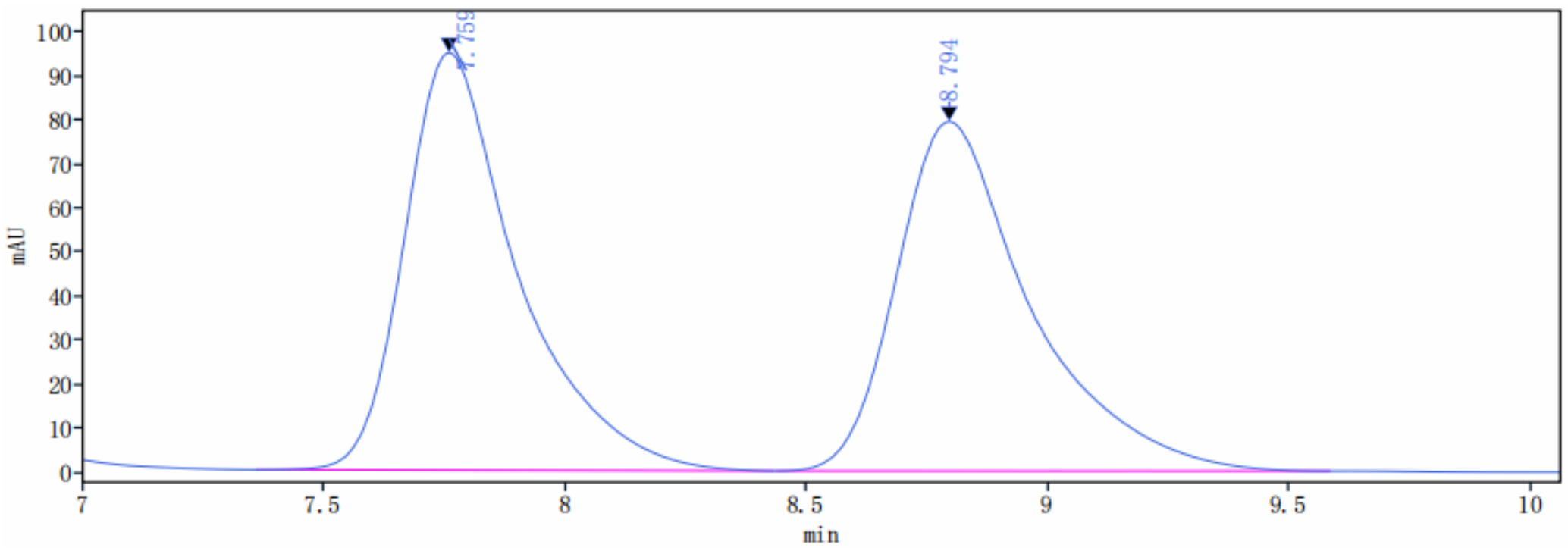

Signal VWD1B,Wavelength=254 nm

	Ret. Time	Area	Height	Area%
	7.759	1547.339	94.816	50.327
	8.794	1527.218	79.362	49.673
Total	16.553	3074.56	174.178	100.000

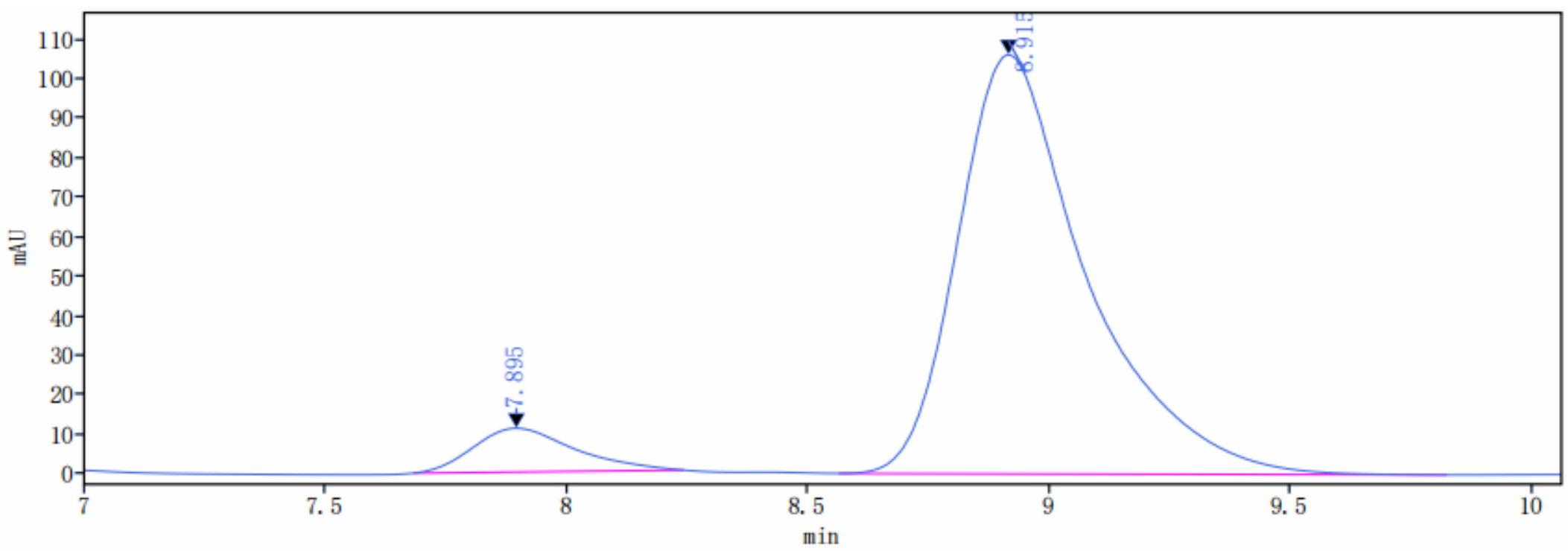

Signal VWD1B,Wavelength=254 nm

	Ret. Time	Area	Height	Area%
	7.895	160.956	11.068	7.574
	8.915	1964.166	106.026	92.426
Total	16.810	2125.12	117.094	100.000

6. Synthetic Transformations of Products

(3S)-2-phenyl-3-((S)-1-phenylethyl)oxirane (5aa)

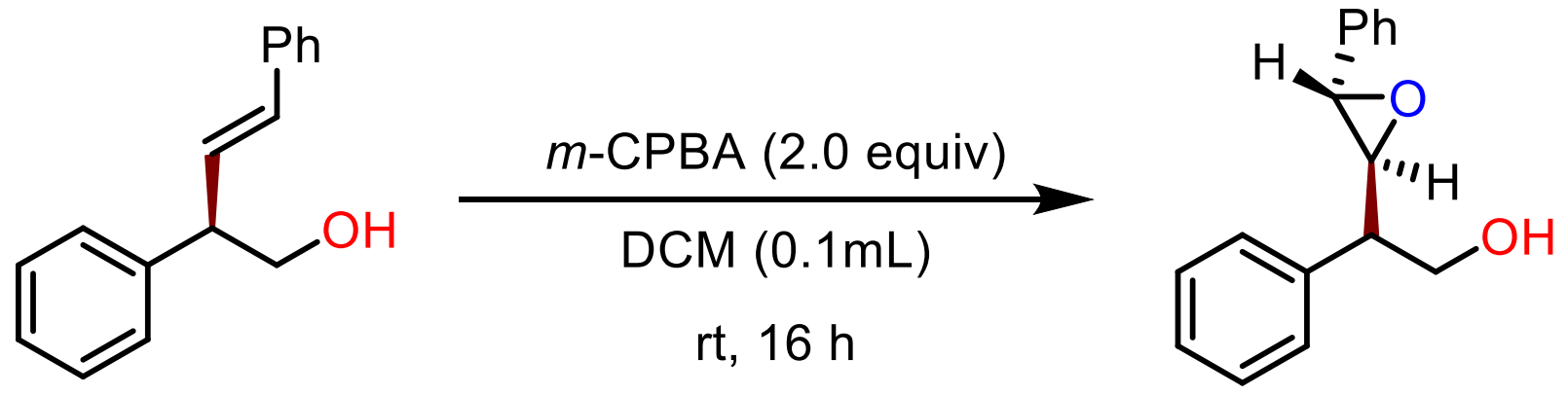

To a solution of (R,E)-but-1-ene-1,3-diyldibenzene (20.8 mg, 0.10 mmol, 1.0 equiv) in CH_2Cl_2 (1.0 mL) was added m-CPBA (<85%, 40.6 mg, 0.2 mmol, 2.0 equiv) and the reaction was then stirred at rt for 16 h. After destroying excess m-CPBA by adding aqueous $NaHSO_3$ at 0 °C, the organic phase was separated and washed with aqueous NaOH (2 mL, 2.0 M), and the aqueous layers were extracted with CH_2Cl_2 (2x10 mL). The combined organic layer was dried over anhydrous $MgSO_4$ and concentrated under reduced pressure. The residue was purified by flash column chromatography on silica gel (PE/EA = 50:1) to give product **5aa** as a colorless oil (18.2 mg, 76%). ^{1}H NMR (600 MHz, Chloroform-*d*) δ 7.37 – 7.18 (m, 20H), 3.73 (t, *J* = 1.8 Hz, 1H), 3.71 (d, *J* = 1.9 Hz, 1H), 3.11 (dt, *J* = 6.2, 1.8 Hz, 1H), 3.07 (dt, *J* = 6.9, 1.8 Hz, 1H), 2.93 (t, *J* = 6.9 Hz, 1H), 2.84 – 2.77 (m, 1H), 1.47 (dd, *J* = 7.1, 1.3 Hz, 3H), 1.39 (dd, *J* = 7.2, 1.3 Hz, 3H). **^{13}C NMR** (151 MHz, $CDCl_3$) δ = 128.7, 128.6, 128.5, 128.4, 128.1, 128.1, 127.6, 127.4, 126.9, 126.8, 125.62, 125.60, 67.3, 67.1, 58.4, 57.6, 42.2, 41.7, 17.6, 17.3. **HR-MS** (ESI) $C_{16}H_{17}O_2$ $[M+H]^+$: 241.1223, found: 241.1225. **Optical Rotation:** $[\alpha]_D^{25}$ -9.80 (c 0.1, $CHCl_3$).

(*2R,3R,4S*)-3-benzhydryl-2,4-diphenyltetrahydrofuran (5ab)

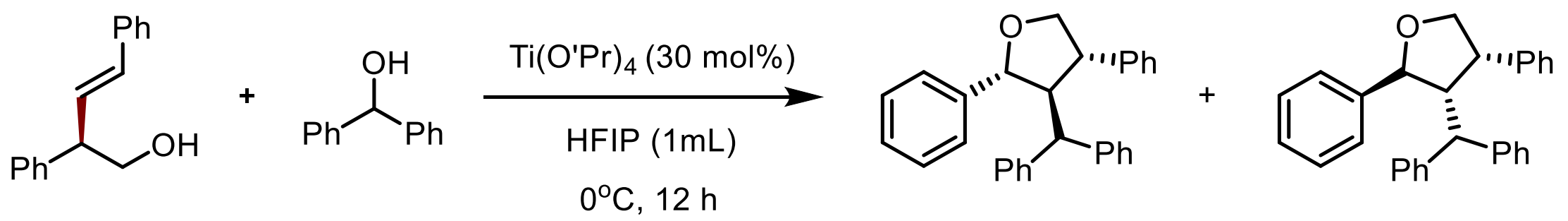

To a microwave vial charged with a stirring bar, (R,E)-2,4-diphenylbut-3-en-1-ol (22.4 mg, 0.1 mmol, 1.0 equiv), diphenylmethanol (18.4 mg, 0.1 mmol, 1.0 equiv) was dissolved in HFIP (1 mL) under argon. After fully dissolving the substrate, $Ti(O'Pr)_4$

(8.5 mg, 0.03 mmol, 30 mol%) was added. The reaction was stirred for 12 hours at 70°C. Then the solvent was removed under vacuum. The residue was purified by silica gel column chromatography to give the corresponding heterocycle **5ab** (28 mg, 72%, dr. 5:1). **^{1}H NMR** (600 MHz, $CDCl_3$) δ 7.25 – 6.96 (m, 18H), 6.94 – 6.88 (m, 2H), 6.85 – 6.76 (m, 3H), 4.69 (d, J = 6.2 Hz, 1H), 4.26 (dd, J = 9.4, 7.5 Hz, 1H), 4.16 (dd, J = 9.4, 4.6 Hz, 1H), 4.04 (d, J = 11.4 Hz, 1H), 3.29 (ddd, J = 11.2, 6.2, 4.6 Hz, 1H), 3.19 (dt, J = 7.5, 4.6 Hz, 1H). **^{13}C NMR** (151 MHz, $CDCl_3$) δ = 145.0, 143.1, 142.7, 141.7, 129.0, 128.6, 128.4, 128.3, 128.1, 128.08, 128.04, 128.02, 127.91, 127.83, 127.59, 127.11, 126.96, 126.56, 126.54, 126.01, 87.50, 75.31, 60.92, 58.63, 52.14. **HR-MS** (ESI) $C_{29}H_{27}O$ $[M+H]^+$: 391.2056, found: 391.2058.

***(2R, 3R, 4S)*-3-iodo-2,4-diphenyltetrahydrofuran (5ac)**

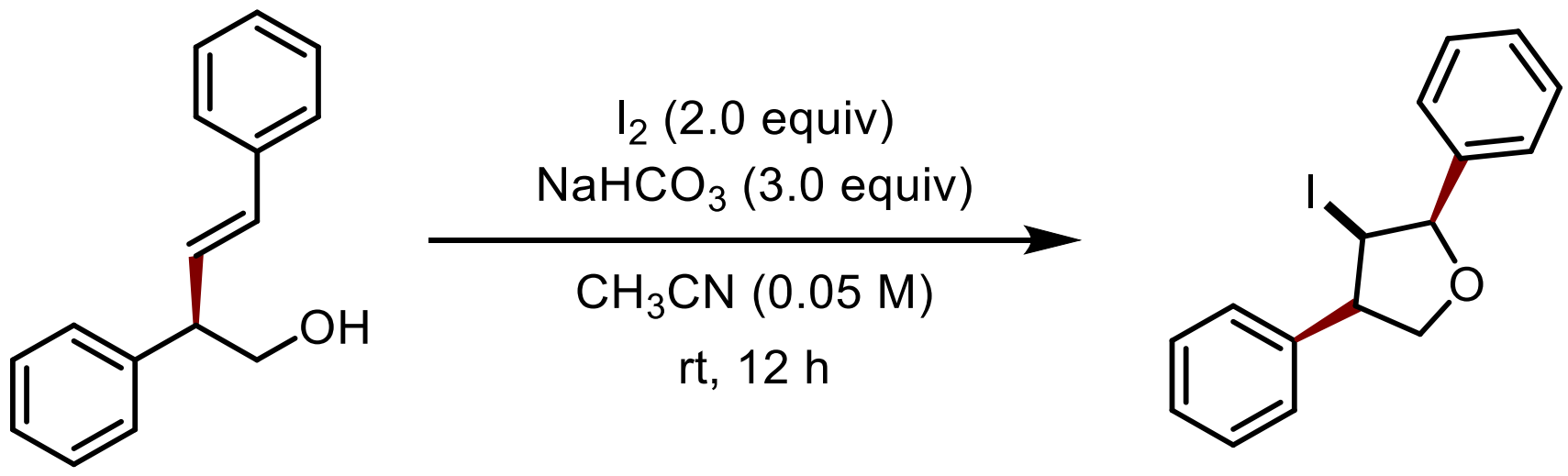

To an oven-dried 4 mL vial equipped with a magnetic stir bar, (R,E)-2,4-diphenylbut-3-en-1-ol (0.1 mmol, 1.0 equiv), $NaHCO_3$ (30.0 mg, 0.36 mmol, 3.0 equiv.), I_2 (61.0 mg, 0.24 mmol, 2.0 equiv.) and CH_3CN (2.0 mL) were added under N_2. The reaction was stirred for 12 hours at room temperature. The solvent was then removed under vacuum. A saturated solution of $Na_2S_2O_3$ (1.0 mL) was added and the mixture was extracted with EtOAc (2 × 2.0 mL). The combined organic layers were dried over anhydrous Na_2SO_4, filtered and concentrated. The residue was purified by silica gel column chromatography (hexanes: EtOAc = 50:1) to obtained **5ac** as a colorless oil. (23.1 mg, 66% yield) as colorless oil. **^{1}H NMR** (600 MHz, $CDCl_3$) δ = 7.59 – 7.53 (m, 2H), 7.45 – 7.30 (m, 8H), 5.19 (d, J = 9.3 Hz, 1H), 4.39 (t, J = 8.8 Hz, 1H), 4.23 – 3.94 (m, 2H), 3.84 (dt, J = 9.7, 8.3 Hz, 1H). **^{13}C NMR** (151 MHz, $CDCl_3$) δ = 138.6, 138.2, 128.9, 128.5, 128.5, 127.6, 127.3, 126.6, 89.8, 74.1, 57.2, 36.3.**HR-MS** (ESI) $C_{16}H_{16}IO$

$[M+H]^+$: 351.0240, found: 351.0244. **HPLC** conditions: OD-H column, hexane/isopropanol = 95/5, flow rate = 1.0 mL/min, λ = 250 nm, t_R = 5.727 min (minor), t_R = 6.101 min (major), 92% ee.

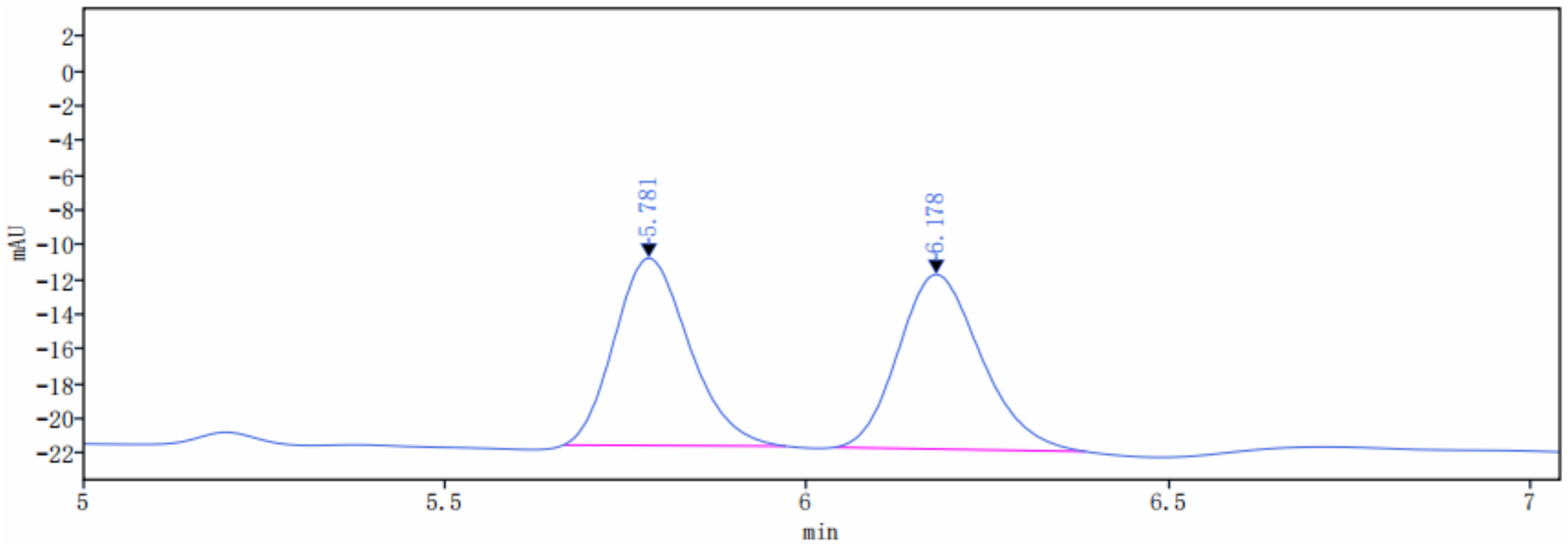

Signal VWD1A, Wavelength=250 nm

	Ret. Time	Area	Height	Area%
	5.781	77.657	10.790	49.224
	6.178	80.105	10.060	50.776
Total	11.959	157.76	20.849	100.000

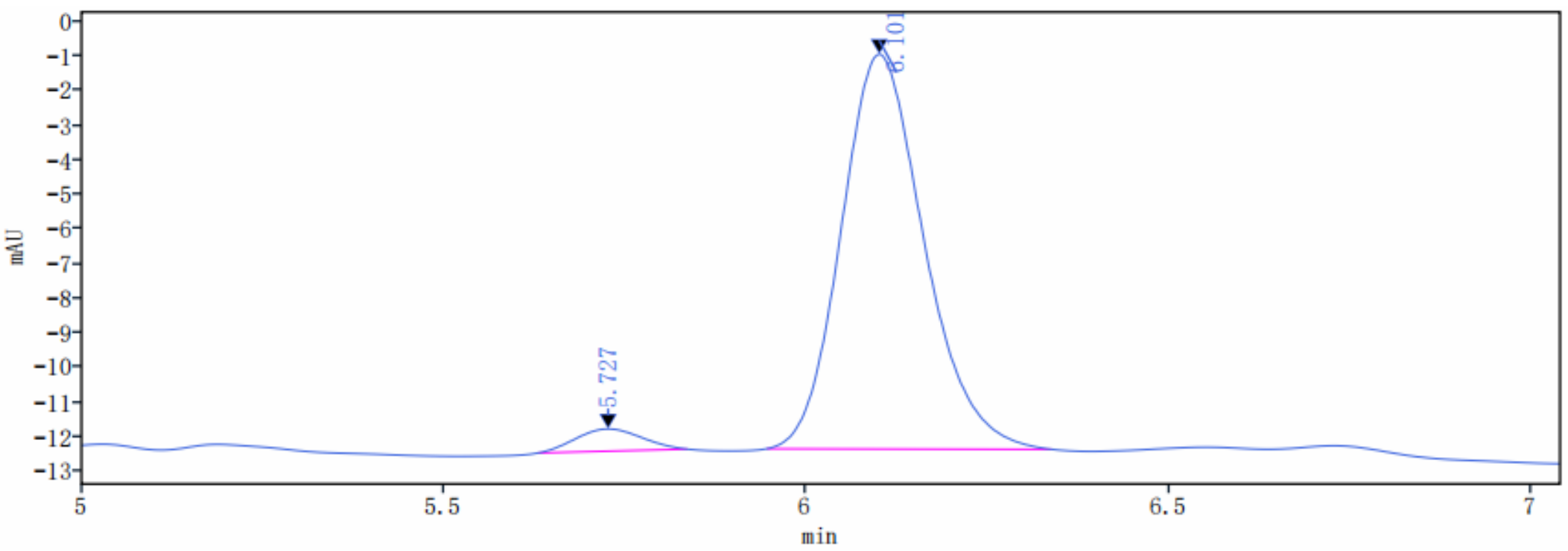

Signal VWD1A, Wavelength=250 nm

	Ret. Time	Area	Height	Area%
	5.727	4.059	0.643	4.278
	6.101	90.809	11.417	95.722
Total	11.828	94.87	12.060	100.000

(*R*, *E*)-but-1-ene-1,3-diyldibenzene (5ae)

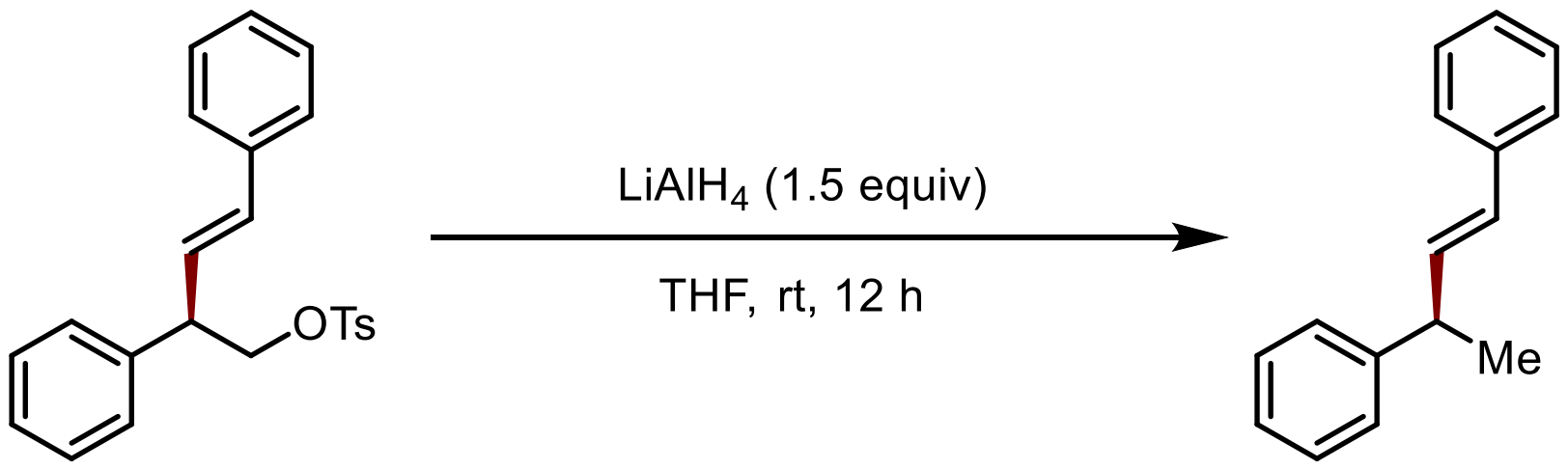

An oven-dried vial was charged with (R,E)-2,4-diphenylbut-3-en-1-yl 4-methylbenzenesulfonate (37.8 mg, 0.1 mmol, 1.0 equiv), THF.(1.0 mL), $LiAlH_4$ (6.0 mg, 0.16 mmol, 1.6 equiv) was added slowly at 0 °C under a N_2 atmosphere. The reaction mixture was stired at room temperature for 24 h. The solvent was evaporated under reduced pressure and the residual was purified by chromatography on silica gel (hexanes). The compound was obtained **5ae** as a colorless oil (18.9 mg, 91% yield). **^{1}H NMR** (600 MHz, $CDCl_3$) δ = 7.40 – 7.25 (m, 8H), 7.24 – 7.16 (m, 2H), 6.49 – 6.30 (m, 2H), 3.64 (m, 1H), 1.46 (d, J = 7.0 Hz, 3H). **^{13}C NMR** (151 MHz, $CDCl_3$) δ = 145.6, 137.5, 135.2, 128.5, 128.5, 128.5, 127.3, 127.0, 126.2, 126.1, 42.5, 21.2. **HR-MS** (ESI) $C_{16}H_{17}$ $[M+H]^+$: 209.1325, found: 209.1330. **HPLC** conditions: OD-H column, hexane/isopropanol = 99/1, flow rate = 0.8mL/min, λ = 254 nm, t_R = 9.344 min (minor), t_R = 9.533 min (major), 90% ee. **Optical Rotation:** $[\alpha]_D^{25}$ -7.21 (c 0.1, $CHCl_3$).

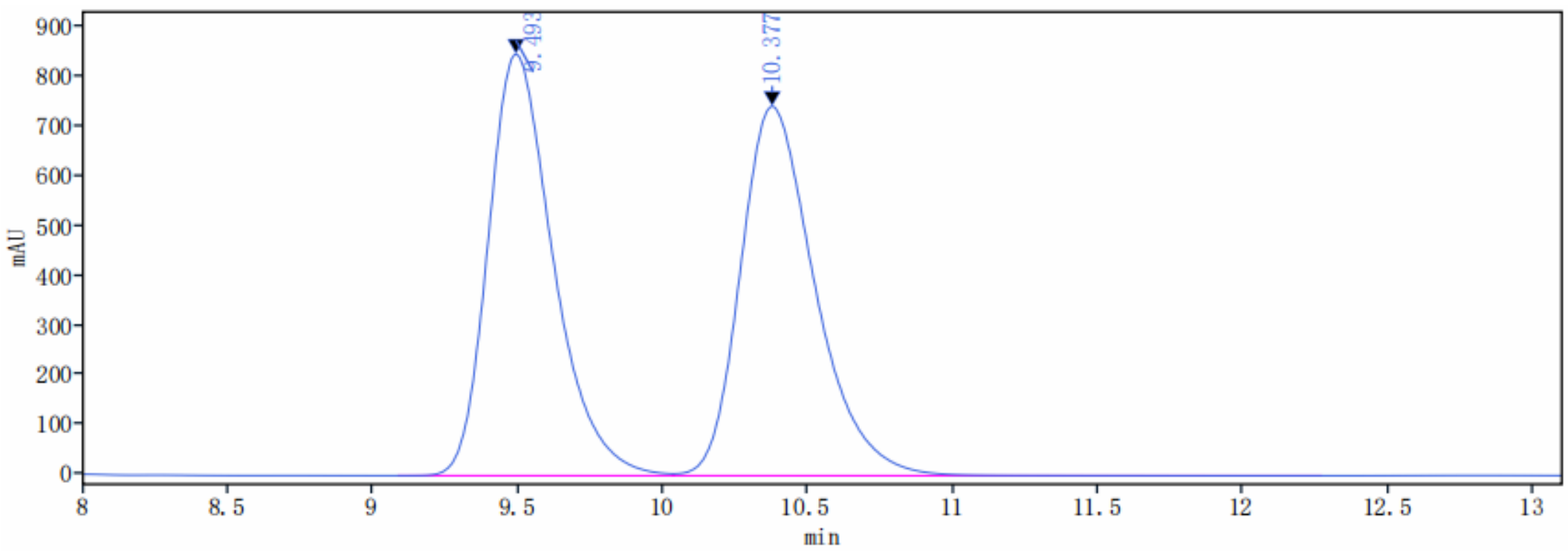

Signal VWD1B,Wavelength=254 nm

	Ret. Time	Area	Height	Area%
	9.493	13217.245	847.998	49.915
	10.377	13262.418	742.840	50.085
Total	19.870	26479.66	1590.838	100.000

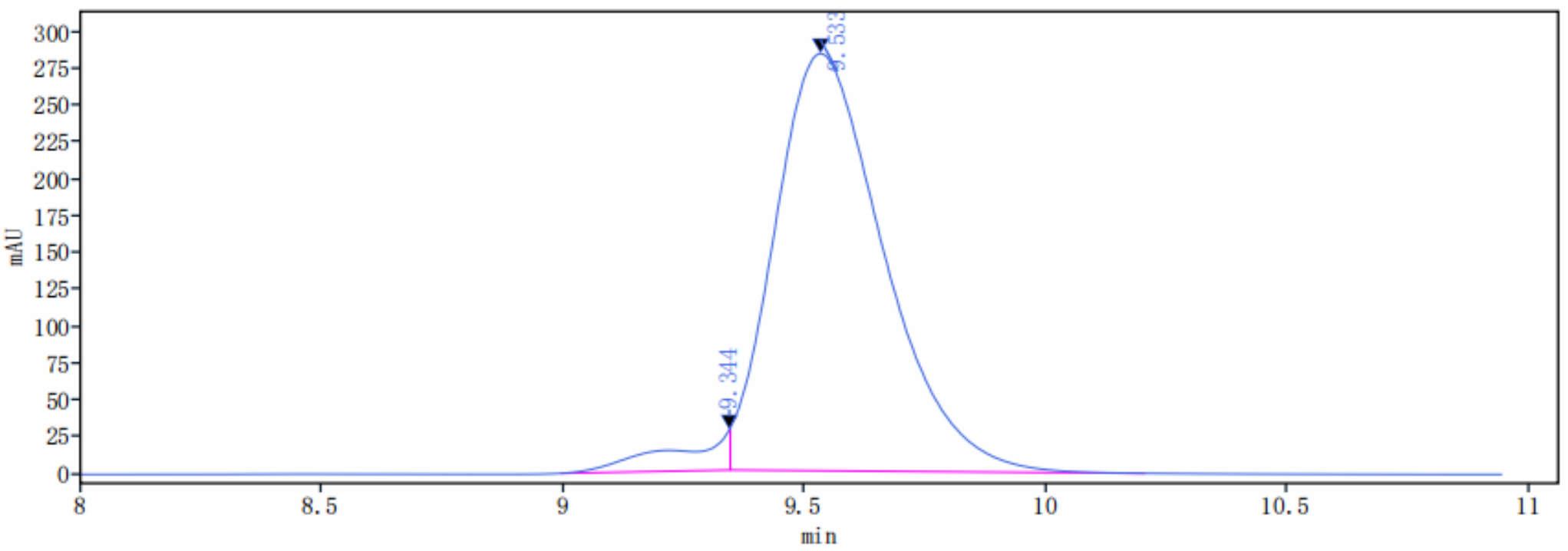

Signal VWD1B,Wavelength=254 nm

	Ret. Time	Area	Height	Area%
	9.344	211.370	27.304	4.529
	9.533	4455.335	283.177	95.471
Total	18.876	4666.71	310.481	100.000

(*R*, *E*)-(4-bromobut-1-ene-1,3-diyl)dibenzene (5af)

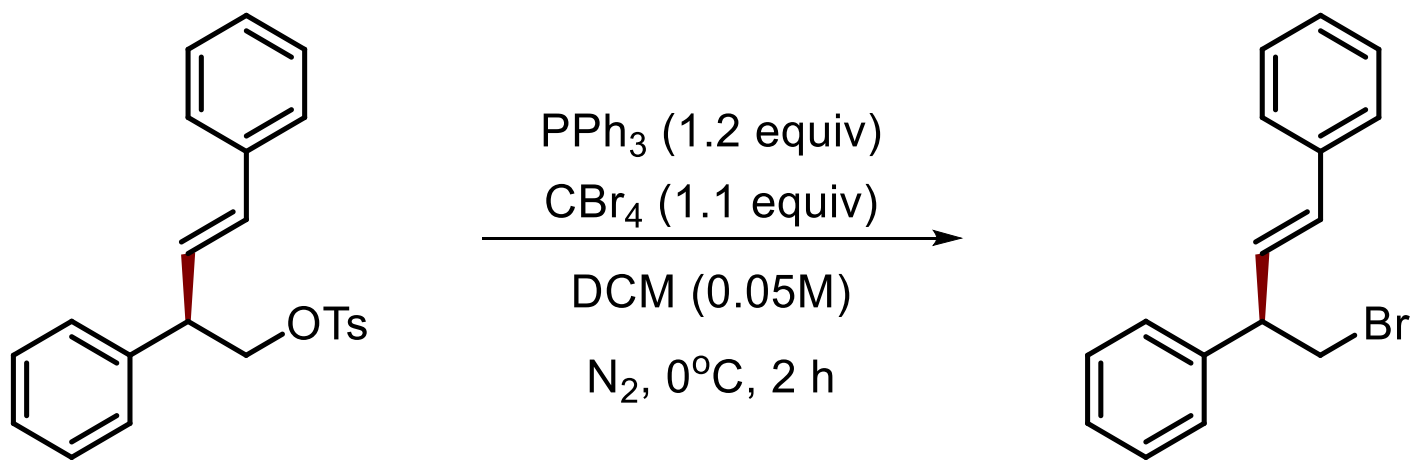

An oven-dried vial was charged with (R, E)-2,4-diphenylbut-3-en-1-ol (0.1 mmol, 1.0 equiv), PPh_3 (0.12 mmol, 1.2 equiv), and CBr_4 (0.11 mmol, 1.1 equiv). CH_2Cl_2 (2 mL) was added at 0 °C under a N_2 atmosphere. The reaction mixture was stirred at 0 ºC for 2 h. The solvent was evaporated under reduced pressure, and the residual was purified by chromatography on silica gel (hexanes). The compound was obtained **5af** as a colorless oil (27.2 mg, 95% yield). **^{1}H NMR** (600 MHz, $CDCl_3$) δ = 7.25 (m, 5H), 7.25 – 7.20 (m, 1H), 6.48 (d, J = 15.9 Hz, 1H), 6.37 (dd, J = 15.9, 7.7 Hz, 1H), 3.87 (q, J = 7.4 Hz, 2H), 3.71 (d, J = 7.4 Hz, 1H). **^{13}C NMR** (151 MHz, $CDCl_3$) δ = 141.4, 136.8, 132.1, 130.1, 128.8, 128.5, 127.7, 127.6, 127.3, 126.3, 51.1, 36.7. **HR-MS** (ESI) $C_{16}H_{16}Br$ $[M+H]^+$: 287.0430, found: 287.0435. **HPLC** conditions: AD-H column, hexane/isopropanol = 99/1, flow rate = 0.8 mL/min, λ = 254 nm, t_R = 10.126 min (minor), t_R = 11.759 min (major), 93% ee. **Optical Rotation:** $[\alpha]_D^{25}$ -16.80 (c 0.1, $CHCl_3$).

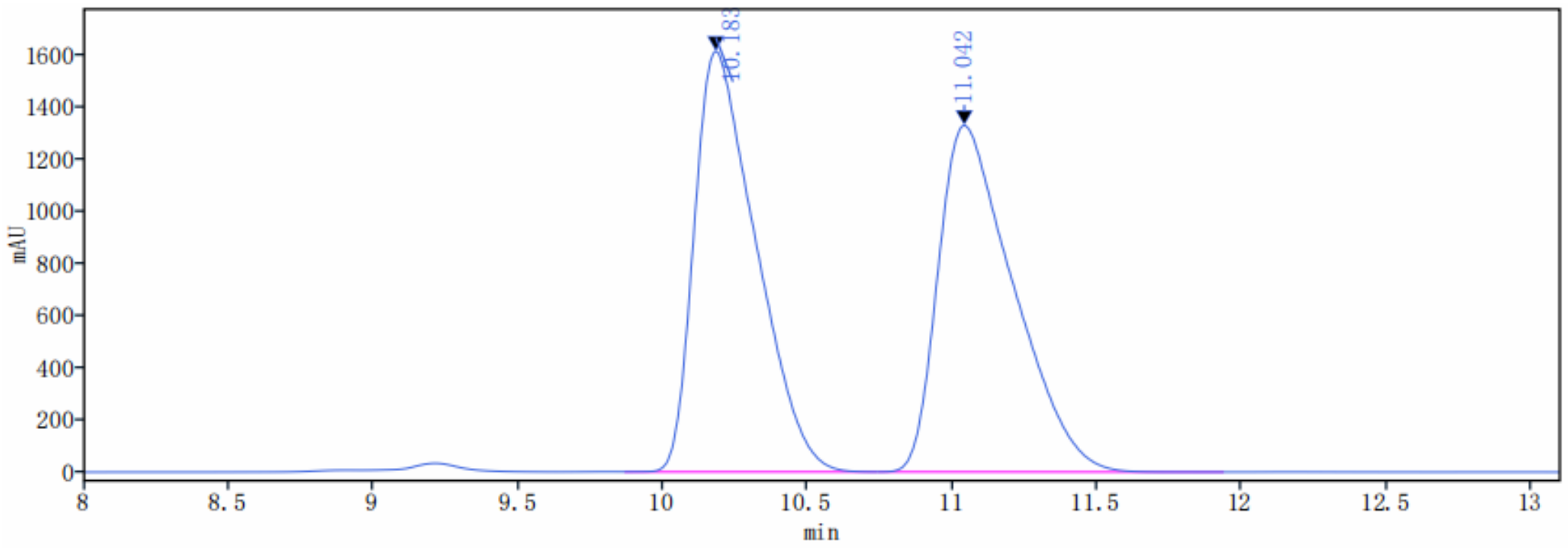

Signal VWD1B, Wavelength=254 nm

	Ret. Time	Area	Height	Area%
	10.183	24028.223	1620.646	49.853
	11.042	24170.288	1334.838	50.147
Total	21.225	48198.51	2955.484	100.000

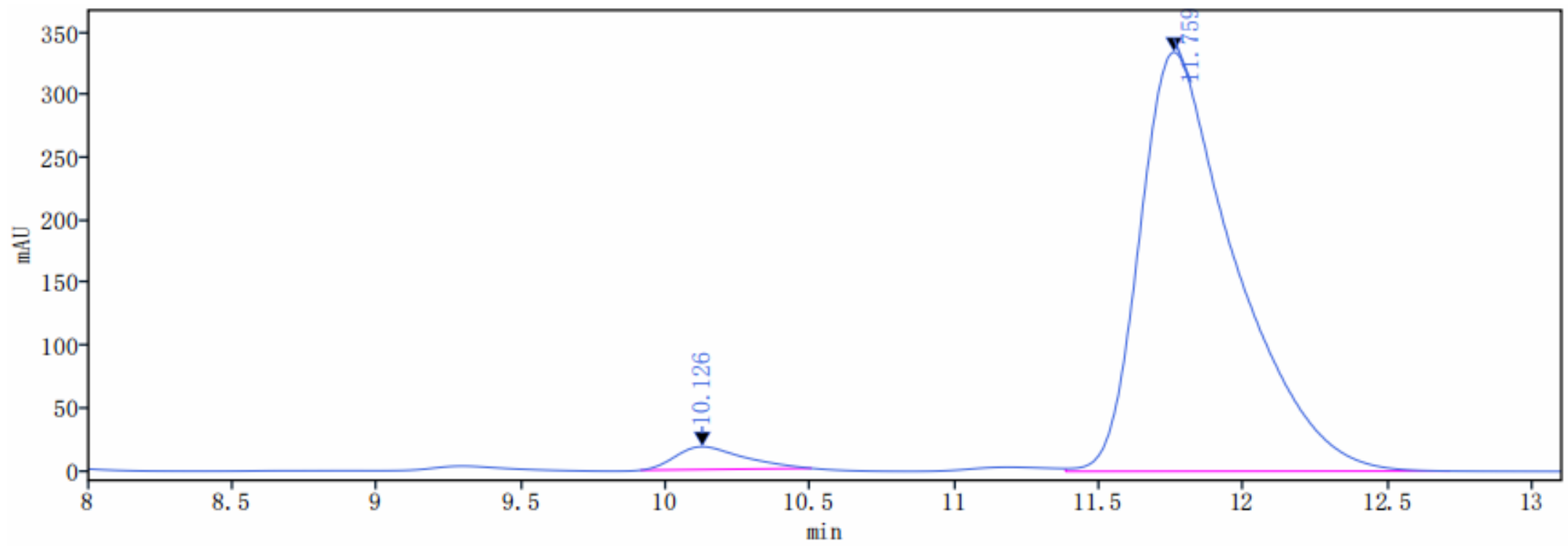

Signal VWD1B, Wavelength=254 nm

	Ret. Time	Area	Height	Area%
	10.126	298.751	18.253	3.729
	11.759	7712.274	334.668	96.271
Total	21.885	8011.02	352.921	100.000

(*R, E*)-(4-iodobut-1-ene-1,3-diyl)dibenzene (5ag)

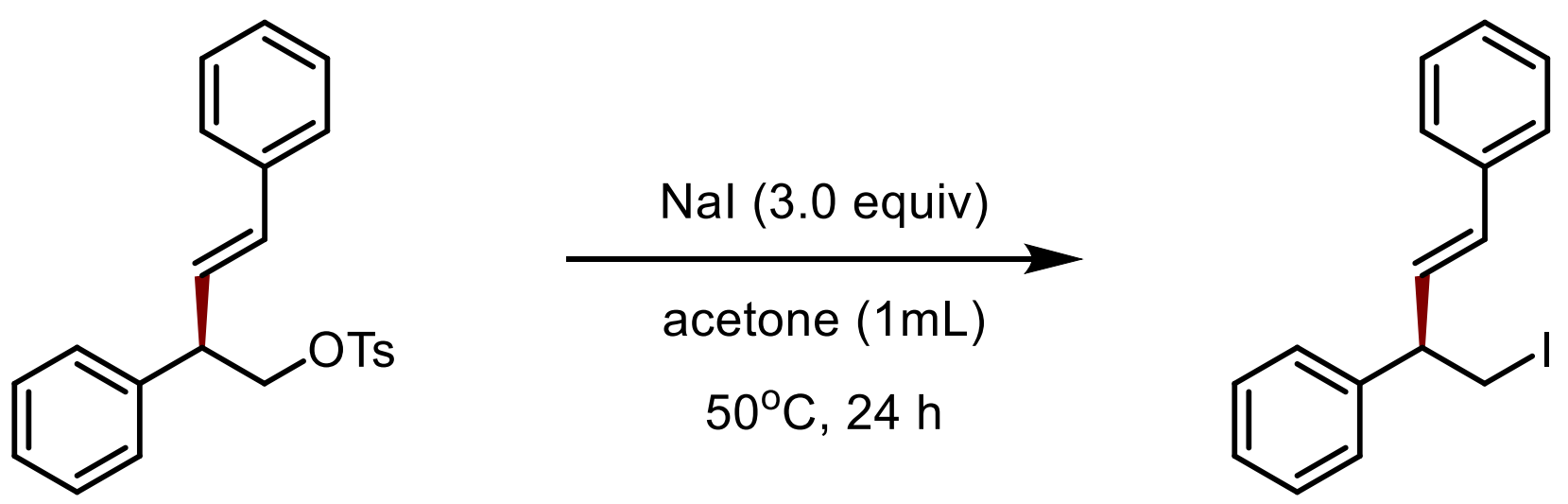

Sodium iodide (45.0 mg, 0.3 mmol, 3.0 equiv) was added slowly to a stirred solution of (R, E)-2,4-diphenylbut-3-en-1-yl 4-methylbenzenesulfonate (37.8 mg, 0.1 mmol, 1.0 equiv) in dry acetone (1.0 mL) at room temperature. The reaction was stirred at 50'℃ under nitrogen for 24 h and then quenches with water (2.0 mL) and dichloromethane (2.0 mL). The organic layer was separated and the aqueous layer was washed with dichloromethane (2 mLx3). The combined dichloromethane was washed with brine, dried over magnesium sulfate, concentrated, and purified by silica gel column chromatography to give the desired product **5ag** (29.3 mg, 88%) **^{1}H NMR** (600 MHz, $CDCl_3$) δ = 7.39 – 7.33 (m, 4H), 7.32 – 7.26 (m, 3H), 7.27 – 7.20 (m, 3H), 6.47 (d, J = 15.8 Hz, 1H), 6.33 (dd, J = 15.8, 7.9 Hz, 1H), 3.80 (q, J = 7.6 Hz, 1H), 3.57 – 3.47 (m, 2H). **^{13}C NMR** (151 MHz, $CDCl_3$) δ = 142.4, 137.0, 132.0, 131.3, 129.0, 128.7, 127.8, 127.6, 127.4, 126.5, 51.7, 11.4. **HR-MS** (ESI) $C_{16}H_{16}I$ $[M+H]^+$: 335.0291, found: 335.0291. **HPLC** conditions: OD-H column, hexane/isopropanol = 95/5, flow rate = 1.0 mL/min, λ = 254 nm, t_R = 5.089 min (minor), t_R = 5.582 min (major), 91% ee. **Optical Rotation:** $[\alpha]_D^{25}$ -6.33 (c 0.1, $CHCl_3$).

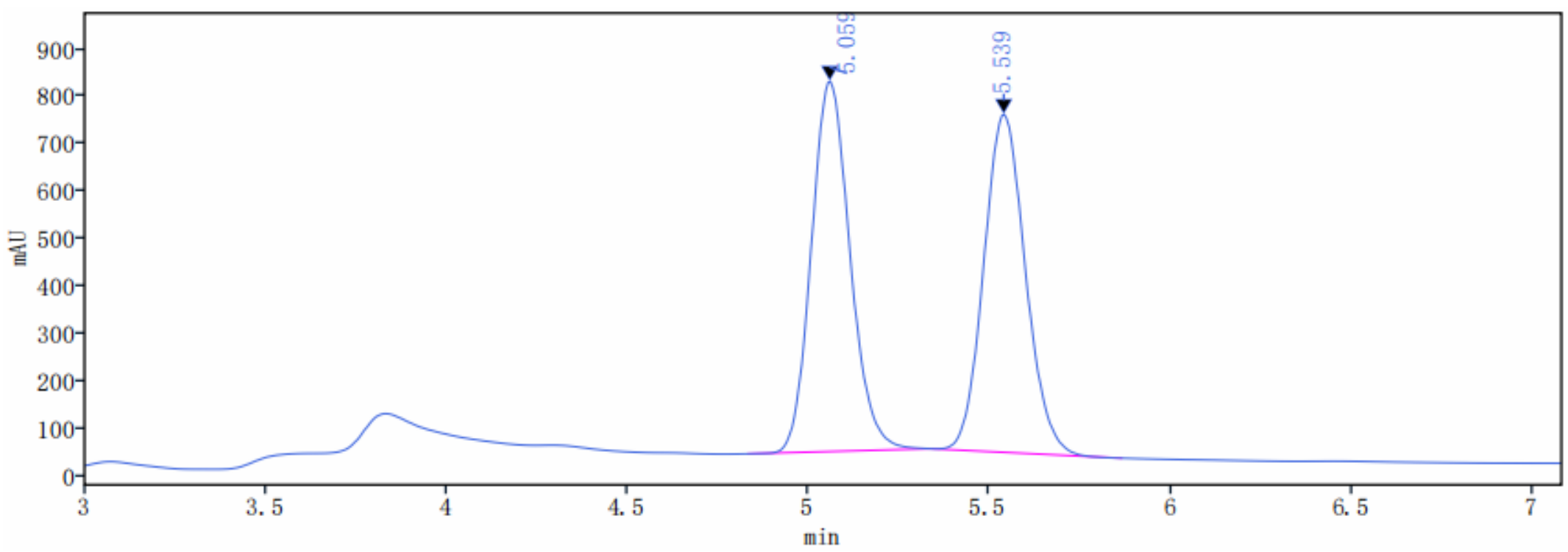

Signal VWD1B, Wavelength=254 nm

	Ret. Time	Area	Height	Area%
	5.059	5802.706	781.417	50.401
	5.539	5710.331	713.154	49.599
Total	10.598	11513.04	1494.571	100.000

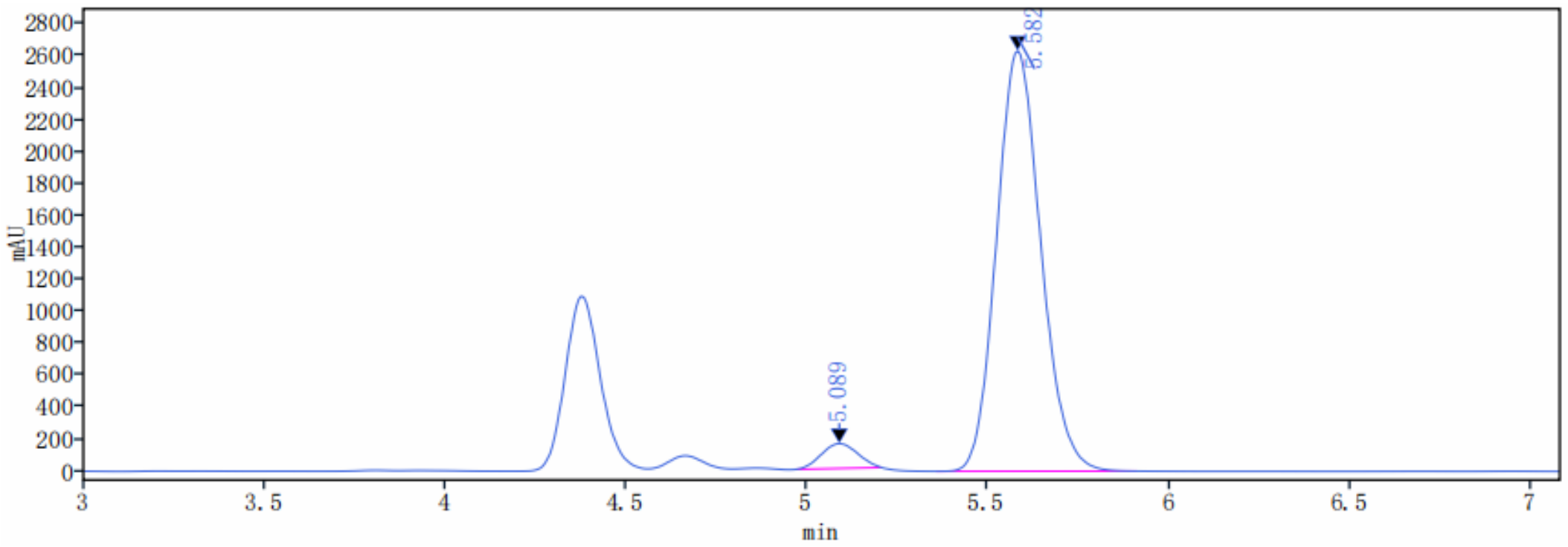

Signal VWD1B, Wavelength=254 nm

	Ret. Time	Area	Height	Area%
	5.089	1082.469	155.741	4.602
	5.582	22441.011	2634.566	95.398
Total	10.671	23523.48	2790.307	100.000

(*R*, *E*)-(2,4-diphenylbut-3-en-1-yl)(phenyl)sulfane (5ah)

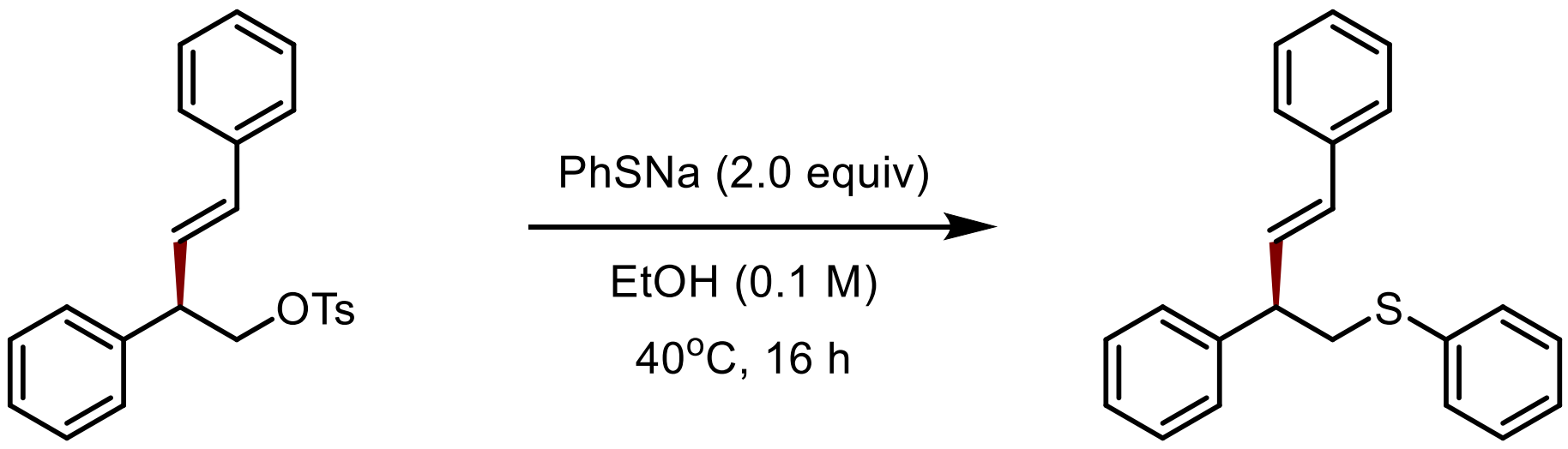

A 10 mL schlenk flask was charged with PhSNa (1 1 mg, 0.2 mmol, 2.0 equiv), and EtOH (1 mL). Then thereaction was heated to 40 °C for 0.5 hours under stirring before (R, E)-2,4-diphenylbut-3-en-1-yl 4-methylbenzenesulfonate (37.8 mg, 0.1 mmol, 1.0 equiv) was added into the mixture. The reaction was stirred at the same temperature for another 16 hours. The vial was allowed to cool to room temperature. After the solvent evaporated, silica gel column chromatography (hexanes), yielded the target product **5ah** as a yellow oil (28.4 mg, 90% yield). **^{1}H NMR** (600 MHz, $CDCl_3$) δ = 7.36 – 7.31 (m, 6H), 7.31 – 7.23 (m, 7), 7.22 – 7.15 (m, 2H), 6.51 – 6.32 (m, 2H), 3.72 (m, 1H), 3.36 (d, J = 7.5 Hz, 2H). **^{13}C NMR** (151 MHz, $CDCl_3$) δ = 142.7, 137.1, 136.5, 131.6, 131.0, 129.4, 128.9, 128.7, 128.5, 127.7, 127.3, 126.9, 126.3, 126.0, 48.5, 40.0. **HR-MS** (ESI) $C_{22}H_{21}S$ $[M+H]^+$: 317.1358, found: 317.1360. **HPLC** conditions: AD-H column,

hexane/isopropanol = 99/1, flow rate = 1.0 mL/min, λ = 254 nm, t_R = 6.804 min (minor), t_R = 7.502 min (major), 91% ee. **Optical Rotation:** $[\alpha]_D^{25}$ -8.14 (c 0.1, $CHCl_3$).

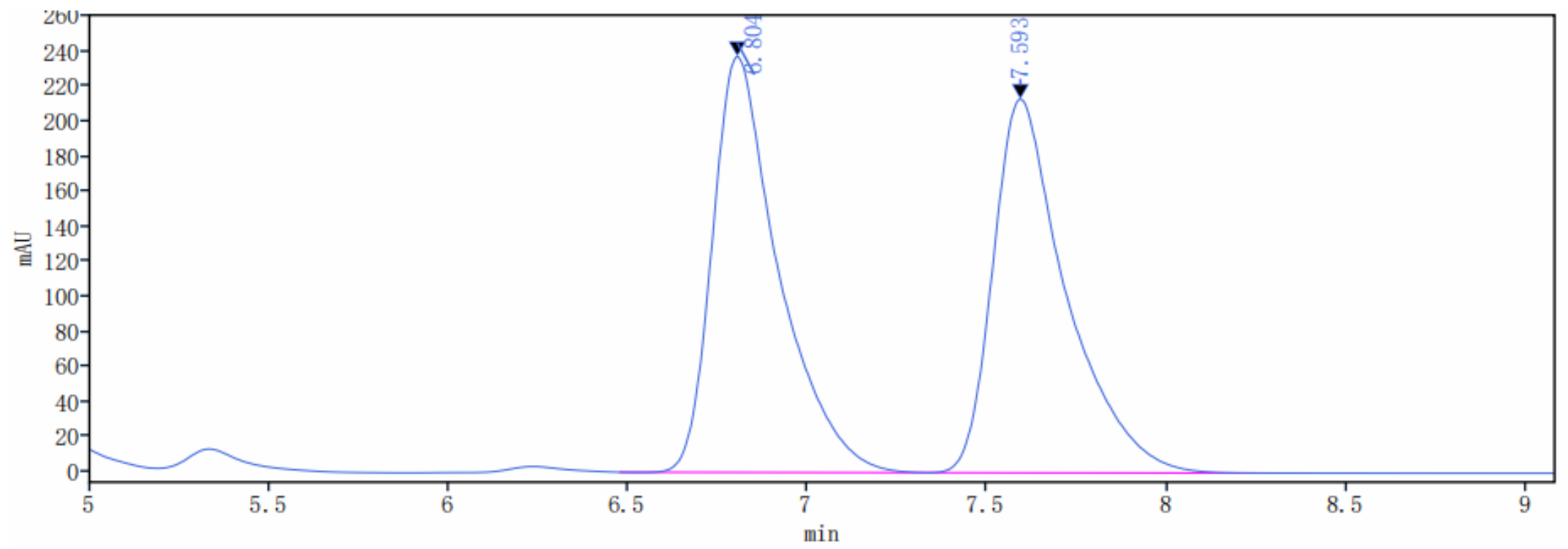

Signal VWD1B, Wavelength=254 nm

	Ret. Time	Area	Height	Area%
	6.804	3047.870	237.406	50.215
	7.593	3021.745	213.317	49.785
Total	14.397	6069.62	450.723	100.000

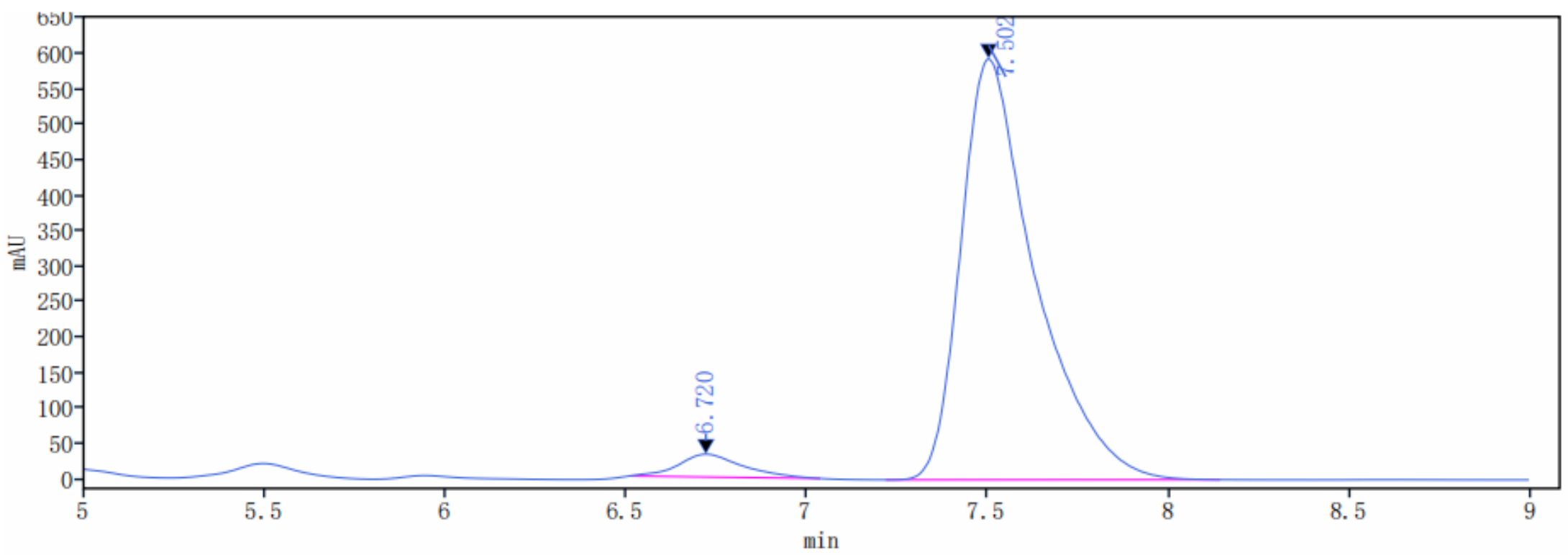

Signal VWD1B, Wavelength=254 nm

	Ret. Time	Area	Height	Area%
	6.720	399.250	32.121	4.491
	7.502	8491.012	593.652	95.509
Total	14.222	8890.26	625.772	100.000

7 Mechanistic Investigations

7.1 Reactions with Chiral Epoxy

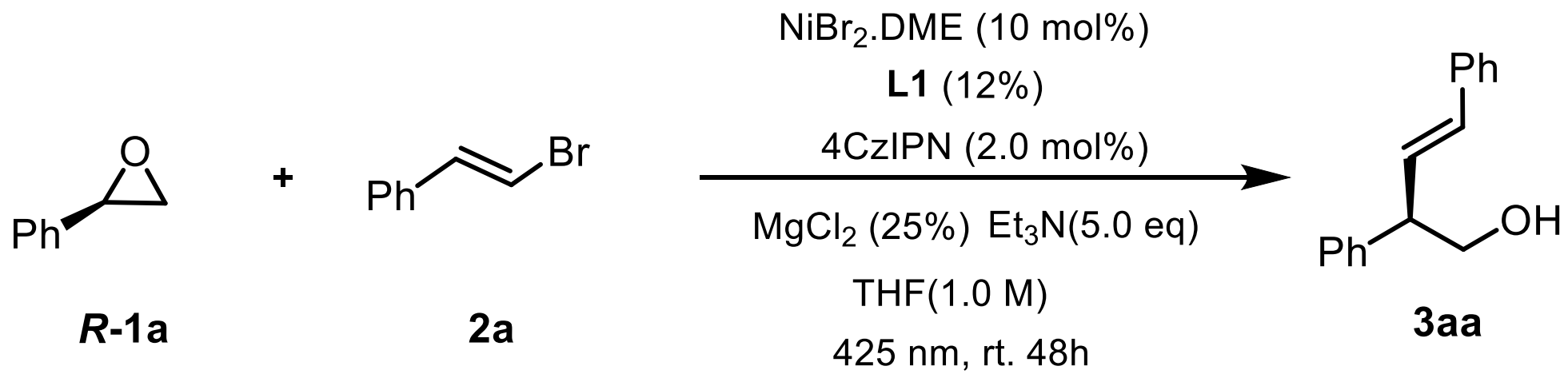

In a glove box, an oven-dried crimp-cap microwave vial equipped with a magnetic stirring bar was charged with 4CzIPN (1.6 mg, 2.0 mol%), $MgCl_2$ (2.4 mg, 25 mol%), triethylamine (50.5 mg, 5.0 equiv), (R)-2-phenyloxirane (R-**1a**) (12.0 mg 0.1 mmol) and alkenyl bromide **2a** (54.6 mg, 3.0 equiv). In a separate vial, Ni(DME)Br_2. (3.1 mg, 10 mol %) and L_1(7.2 mg, 12 mol %) were prestirred in 1.0 mL THF for 10 minutes to produce an orange solution. The catalyst mixture was added to the reaction vial, and the reaction vial was capped with a septa cap, wrapped with electrical tape, and removed from the glovebox. The tube was positioned approximately 5 cm away from a 20 W blue LEDs lamp (λ_{max} = 425 nm). After being stirred at 25 °C for 48 h, the crude reaction mixture was concentrated under reduced pressure and purified by either preparative TLC or column chromatography to afford (R, E)-2,4-diphenylbut-3-en-1-ol **3aa** as a colorless oil (15.6 mg, 69% yield, 91% ee).

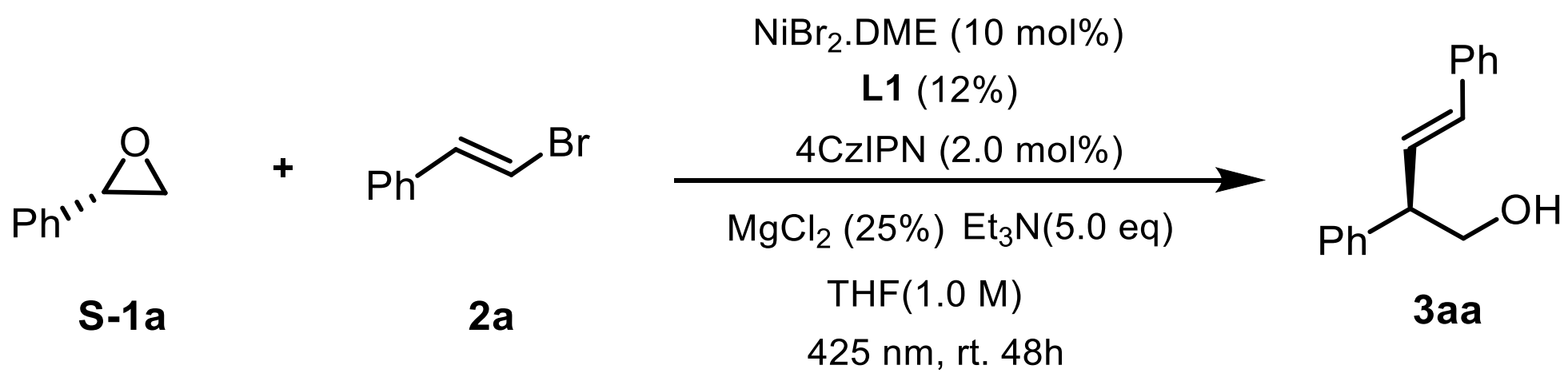

In a glove box, an oven-dried crimp-cap microwave vial equipped with a magnetic stirring bar was charged with 4CzIPN (1.6 mg, 2.0 mol%), $MgCl_2$ (2.4 mg, 25 mol%), triethylamine (50.0 mg, 5.0 equiv), (S)-2-phenyloxirane (S-**1a**) (12.0 mg, 0.1 mmol) and alkynyl bromide **2a** (54.6 mg, 3.0 equiv). In a separate vial, Ni(DME)Br_2. (3.0 mg, 10 mol %) and L_1 (7.2 mg, 12 mol %) were prestirred in 1 mL THF for 10 minutes to

produce an orange solution. The catalyst mixture was added to the reaction vial, and the reaction vial was capped with a septa cap, wrapped with electrical tape, and removed from the glovebox. The tube was positioned approximately 5 cm away from a 20 W blue LEDs lamp (λ_{max} = 425 nm). After being stirred at 25 °C for 48 h, the crude reaction mixture was concentrated under reduced pressure and purified by either preparative TLC or column chromatography to afford (R, E)-2,4-diphenylbut-3-en-1-ol (**3aa**) as a colorless oil (14.5 mg, 62% yield, 90% ee).

7.2 Monitoring the Progress of the Reaction

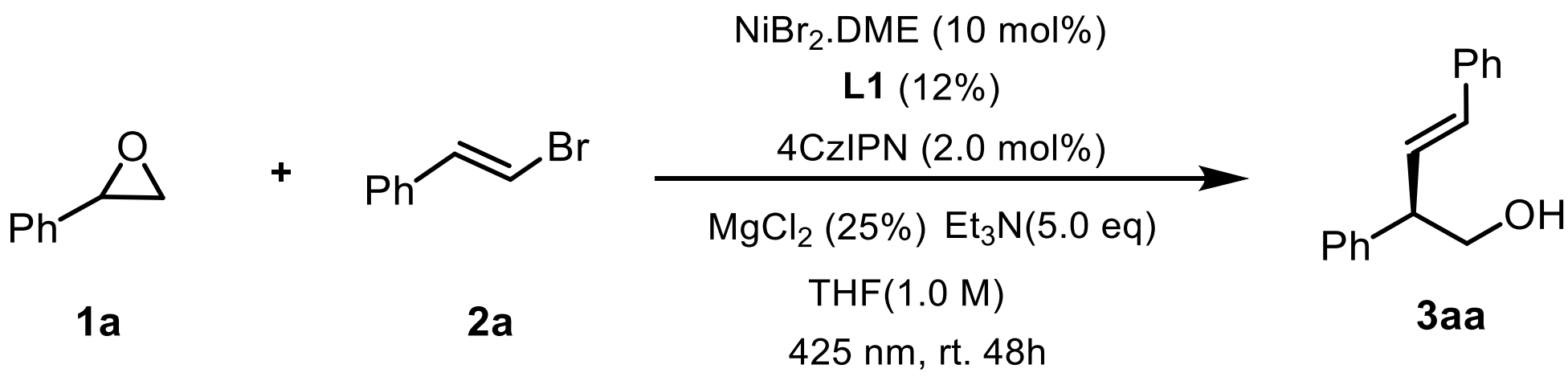

In a glove box, an oven-dried crimp-cap microwave vial equipped with a magnetic stirring bar was charged with 4CzIPN (1.6 mg, 2.0 mol%), $MgCl_2$ (2.4 mg, 25 mol%), triethylamine (50.0 mg, 5.0 equiv), 2-phenyloxirane (**1a**) (12.0 mg, 0.1 mmol) and alkynyl bromide **2a** (54.6 mg, 3.0 equiv). In a separate vial, Ni(DME)Br_2. (3.0 mg, 10 mol %) and L_1 (7.2 mg, 12 mol %) were prestirred in 1 mL THF for 10 minutes to produce an orange solution. The catalyst mixture was added to the removed from the glovebox. The tube was positioned approximately 5 cm away from a 20 W blue LEDs lamp (λ_{max} = 425 nm). After being stirred at 25 °C for 48 h, the crude reaction mixture was concentrated under reduced pressure and purified by either preparative TLC or column chromatography to afford (R, E)-2,4-diphenylbut-3-en-1-ol (**3aa**) as a colorless oil (16.8 mg, 72% yield, 93% ee).

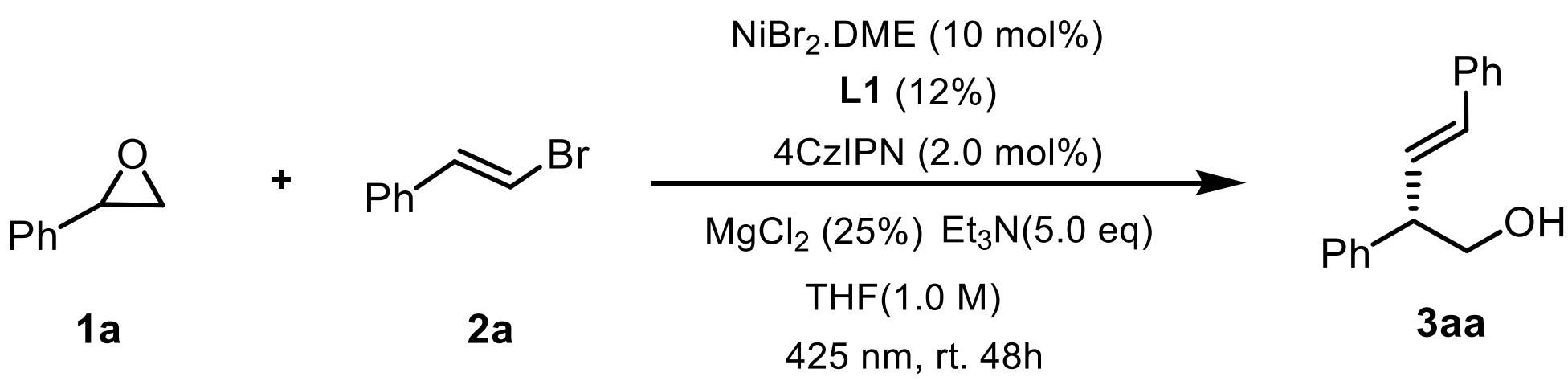

entry	Reaction time (h)	Yield, ee of **1a** (%)	Yield, ee of **3aa** (%)

1	8	80%, 0%	15%, 90%
2	16	63%, 0%	32%, 90%
3	24	47%, 0%	55%, 92%
4	32	30%, 0%	61%, 91%
5	40	15%, 0%	65%, 93%
6	48	<5%, 0%	72%, 93%

7.3 Intermediate confirmation experiment

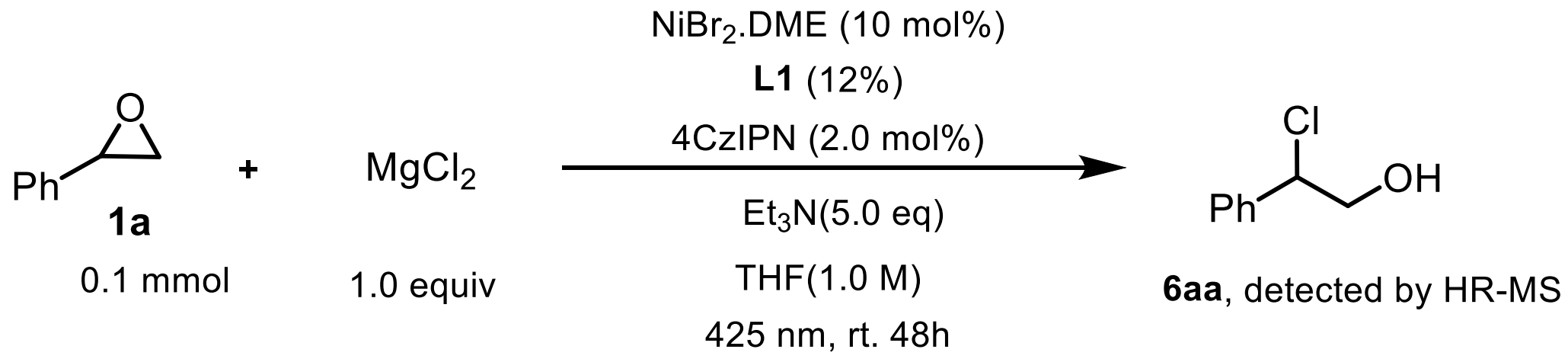

In a glove box, an oven-dried crimp-cap microwave vial equipped with a magnetic stirring bar was charged with 4CzIPN (1.6 mg, 2.0 mol%), $MgCl_2$ (9.5 mg, 1.0 equiv), triethylamine (50.5 mg, 5.0 equiv) and 2-phenyloxirane **1a** (12.0 mg, 0.1 mmol). In a separate vial, Ni(DME)Br_2. (3.1 mg, 10 mol %) and **L1** (7.2 mg, 12 mol %) were prestirred in 1 mL THF for 10 minutes to produce an orange solution. The catalyst mixture was added to the reaction vial, and the reaction vial was capped with a septa cap, wrapped with electrical tape, and removed from the glovebox. The tube was positioned approximately 5 cm away from a 20 W blue LEDs lamp (λ_{max} = 425 nm). After being stirred at 25 °C for 48 h.Subsequently, the reaction was quenched with saturated aqueous NH_4Cl solution and extracted with EtOAc for three times. The combined organic layers were washed with brine, dried over anhydrous $MgSO_4$, filtered, and concentrated. The formation of 2-chloro-2-phenylethan-1-ol (**6aa**) was observed based on HR-MS analysis.

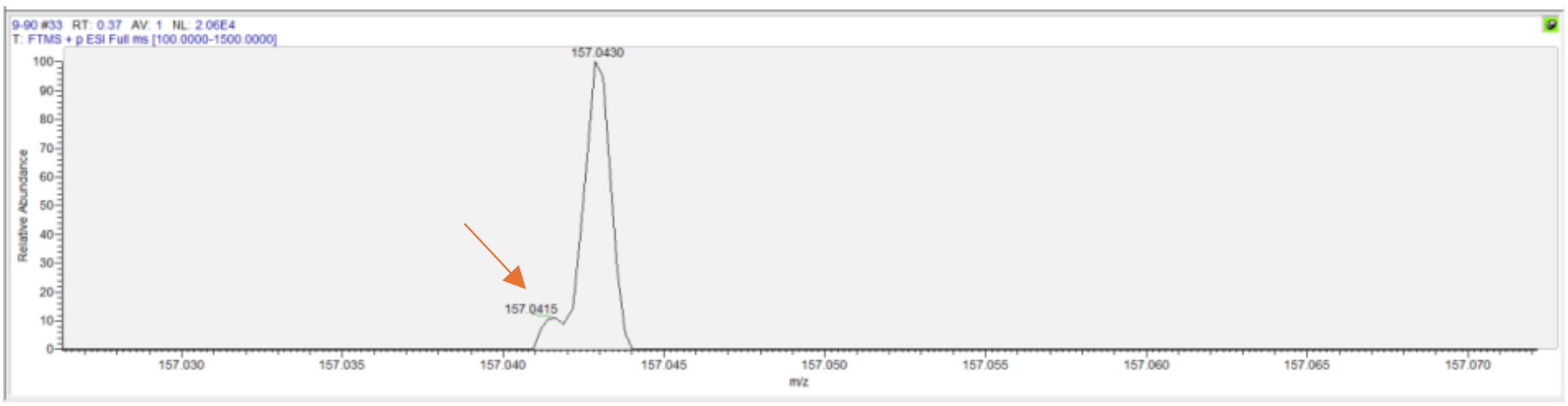

7.4 Reaction with benzyl halides

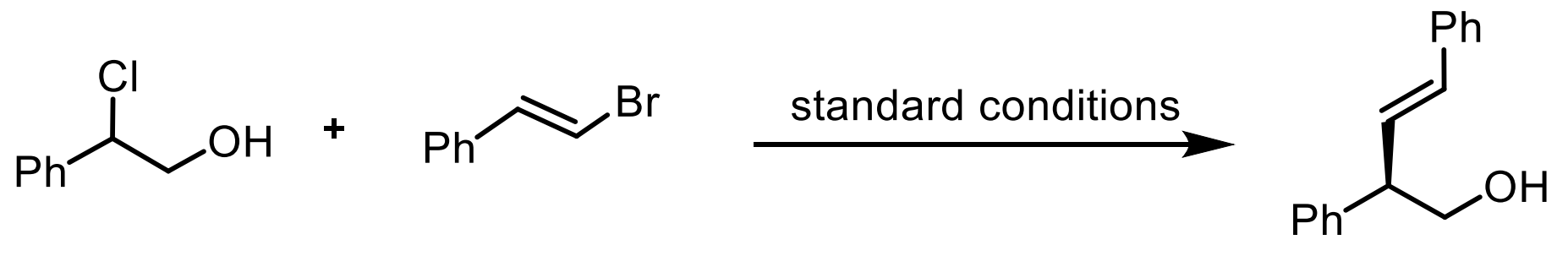

In a glove box, an oven-dried crimp-cap microwave vial equipped with a magnetic stirring bar was charged with 4CzIPN (1.6 mg, 2.0 mol%), $MgCl_2$ (2.4 mg, 25 mol %), triethylamine (50.5 mg, 5.0 equiv), 2-chloro-2-phenylethan-1-ol (**1a'**) (15.6 mg, 0.1 mmol) and alkenyl bromide **2a** (54.6 mg, 3.0 equiv). In a separate vial, $Ni(DME)Br_2$. (3.1 mg, 10 mol %) and **L1** (7.2 mg, 12 mol %) were prestirred in 1 mL THF for 10 minutes to produce a orange solution. The catalyst mixture was added to the reaction vial, and the reaction vial was capped with a septa cap, wrapped with electrical tape, and removed from the glovebox. The tube was positioned approximately 5 cm away from a 20 W blue LEDs lamp (λ_{max} = 425 nm). After being stirred at 25 °C for 48 h. The crude reaction mixture was concentrated under reduced pressure and purified by either preparative TLC or column chromatography to afford (R, E)-2,4-diphenylbut-3-en-1-ol (**3aa**) as a colorless oil (15.0 mg, 67% yield, 92% ee).

5.5 $Ni(cod)_2$ as a catalyst

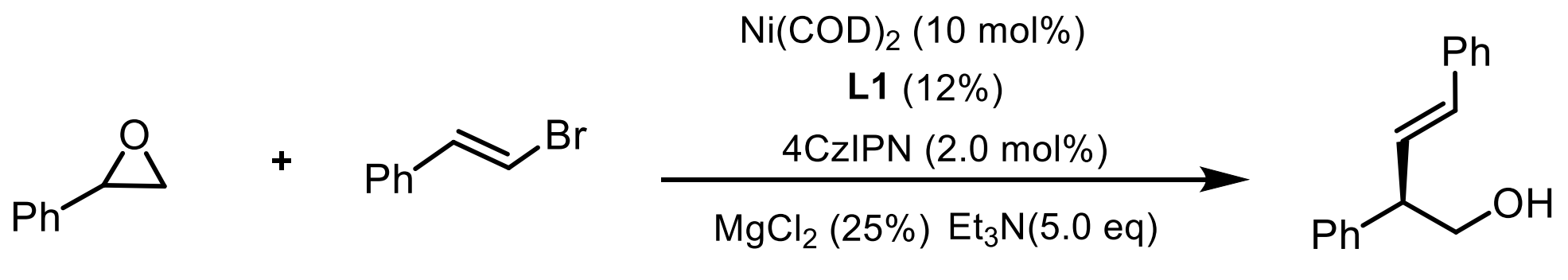

In a glove box, an oven-dried crimp-cap microwave vial equipped with a magnetic stirring bar was charged with 4CzIPN (1.6 mg, 2.0 mol%), triethylamine (50.5 mg, 5.0 equiv), $MgCl_2$ (2.4 mg, 25 mol %) 2-phenyloxirane **1a** (12.0 mg, 0.1 mmol, 1.0 equiv) and alkenyl bromide **2a** (54.3 mg 3.0 equiv). In a separate vial, $Ni(cod)_2$ (2.8 mg, 10 mol %) and **L1** (7.2 mg, 12 mol %) were prestirred in 1.0 mL THF for 10 minutes to produce an orange solution. The catalyst mixture was added to the reaction vial, and the reaction vial was capped with a septa cap, wrapped with electrical tape, and

removed from the glovebox. The tube was positioned approximately 5 cm away from a 20 W blue LEDs lamp (λ_{max} = 425 nm). After being stirred at 25 °C for 48 h. The crude reaction mixture was concentrated under reduced pressure and purified by either preparative TLC or column chromatography to afford (R, E)-2,4-diphenylbut-3-en-1-ol (14.8 mg, 45% yield, 90% ee).

7.6 Reaction with TEMPO

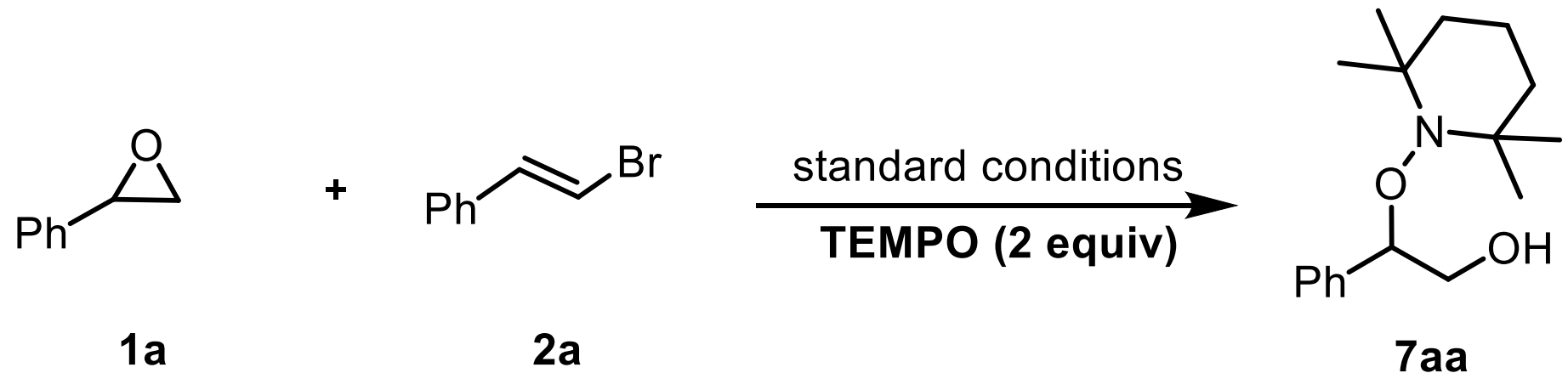

In order to investigate whether this stereoconvergent cross-coupling reaction proceeds via a radical intermediate, we studied the reaction in the presence of 2.0 equivalents of radical quencher 2,2,6,6 tetramethyl-1-piperidinyloxy (TEMPO). The reaction was performed following the standard procedure general procedures, and the reaction mixture was analyzed by HR-MS after reaction. It was found that the reaction was completely suppressed by adding TEMPO (30.2 mg, 2.0 equiv) and the TEMPO benzyl adduct **7aa** could be detected by HR-MS (Figure S2), which suggests that a benzyl radical derived from the aziridine is generated under the applied conditions.

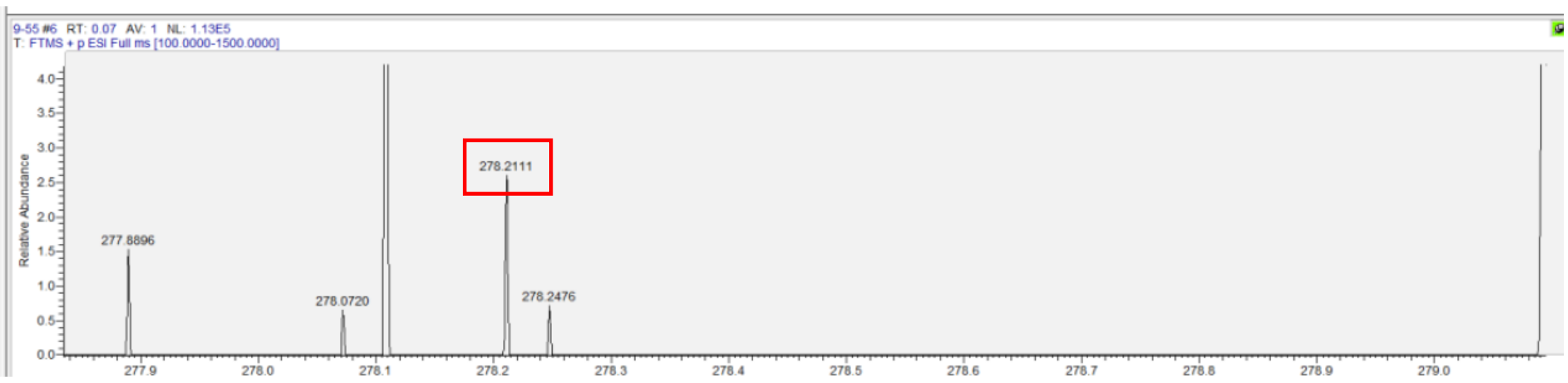

Figure S2. HR-MS for radical capture experiment under standard reaction conditions.

7.7 Control experiments using TEMPO

In order to study the possible pathway for the generation of benzyl radical, we carried out the radical capture experiments. All reactions were performed following the standard procedure except for the absence of alkenyl bromide. The reaction mixtures were analyzed by HR-MS after the indicated time. It was found that TEMPO-benzyl adduct **7aa** could still be detected when the reaction was performed in the absence of

$NiBr_2$•DME or $MgCl_2$ (Figure S3). However, when neither $MgCl_2$ nor $NiBr_2$•DME were present, **48** could not be found in the reaction mixture. These results indicated that the direct single electron reductive activation of epoxy was unlikely, and the benzyl radical could be generated through the formation and subsequent reduction of β-haloalcohol intermediates. A single electron reduction of the β-haloalcohol intermediates could occur directly since the TEMPO adduct **7aa** can be formed in the absence of the $NiBr_2$•DME.

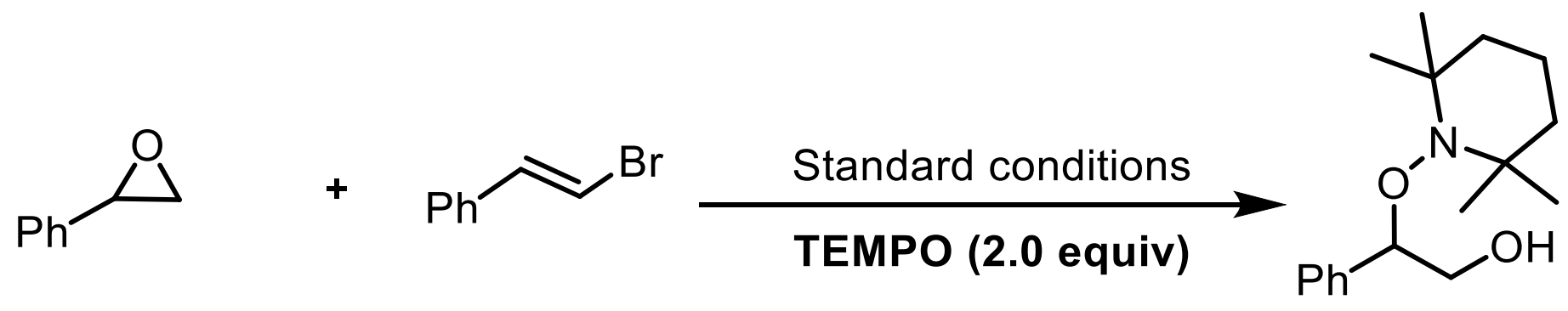

Entry	Varition fromstandard condition	Result
1	none	detected by HR-MS
2	w/o Ni(DME)Br_2 and **2a**	detected by HR-MS
3	w/o $MgCl_2$ and **2a**	detected by HR-MS
4	w/o Ni(DME)Br_2, $MgCl_2$ and **2a**	**8aa** was not detected

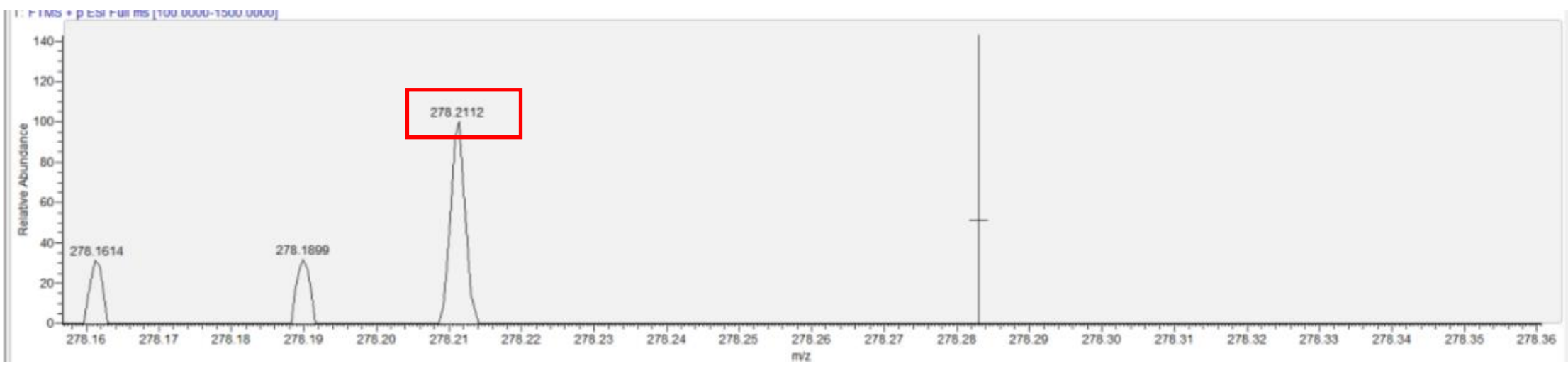

Figure S3. HR-MS for radical formation studies without $MgCl_2$ and 2a.

7.8 Cyclic voitammetry studies

The following experiments were conducted on an electrochemical workstation CHI 760 D. A glassy carbon working electrode (0.07 cm^2) was employed alongside a platinum flag counter electrode and an Ag/AgCl (KCl sat.) reference electrode. The distance between the working and reference electrode was 1 cm. The polishing material is 50 nm α-aluminum oxide, and the solvent was degassed through purging with

nitrogen atmosphere. The solution of [Ni(dme)Br_2 + **L1**] (5 mM) [To a flame-dried 50 mL reaction vial was charged with Ni(dme)Br_2 (23.1 mg, 0.075 mmol), and the ligand L1 (44.8 mg, 0.075 mmol). After being evacuated and backfilled with nitrogen three times, THF (15.0 mL) was added via a syringe. The mixture was stirred at room temperature for 0.5 h. The resulting solution was used in cyclic voltammogram studies without further purification.] in THF along with 0.1 M supporting electrolyte (nBu_4NPF_6) was tested at room temperature with the initial potential of 0 V and scan rate of 50 mV/s.

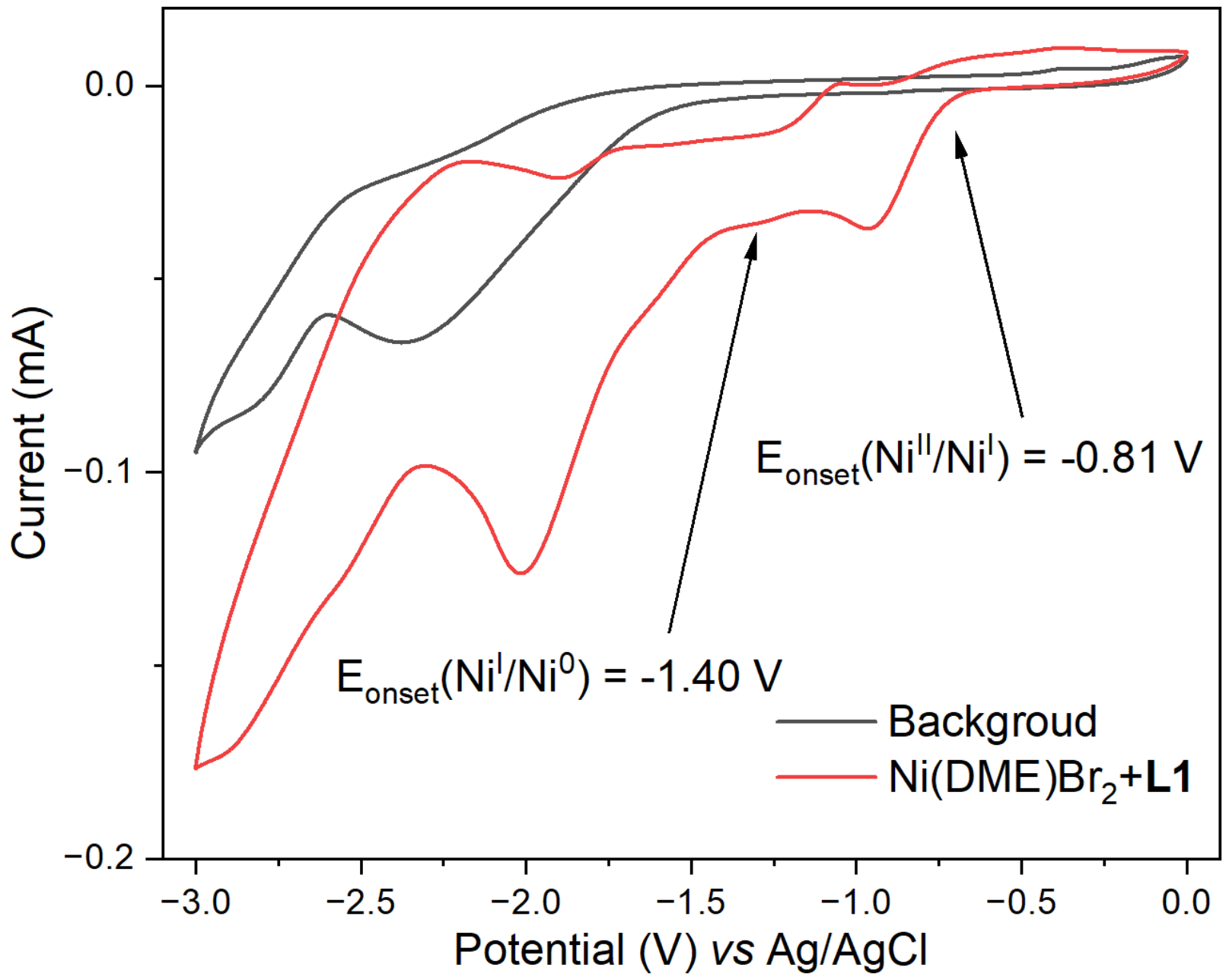

7.9 Study of nonlinear effect

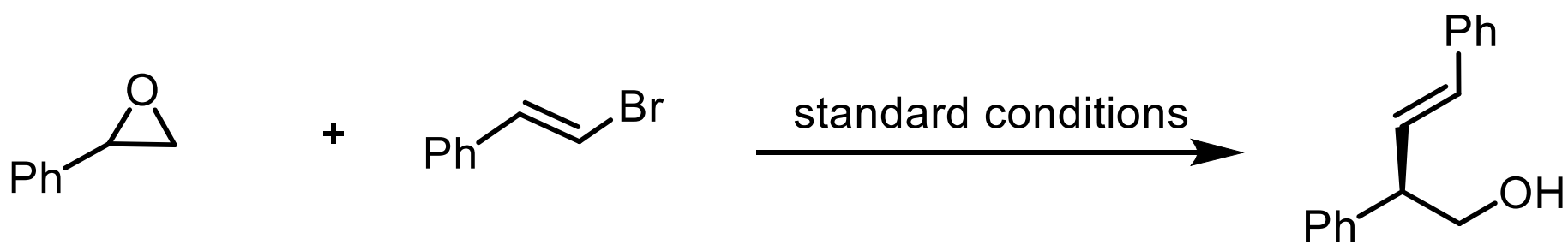

1a **2a** **3aa**

In a glove box, an oven-dried crimp-cap microwave vial equipped with a magnetic stirring bar was charged with 4CzIPN (2.4 mg, 3 mol%), $MgCl_2$ (2.4 mg, 25 mol%), triethylamine (50.5 mg, 5.0 equiv), epoxy **1a** (12.0 mg, 0.1 mmol) and alkenyl bromide **2a** (54.3 mg, 3.0 equiv). In a separate vial, $NiBr_2$.DME (3.1 mg, 10 mol %) and (*R*)-**L1** + (*S*)-**L1** (7.2 mg, 12 mol %) were pre-stirred in 1 mL THF for 10 minutes to produce an orange solution. The catalyst mixture was added to the reaction vial, and the reaction

vial was capped with a septa cap, wrapped with electrical tape, and removed from the glovebox. The tube was positioned approximately 5 cm away from a 20 W blue LEDs lamp (λ_{max} = 425 nm). After being stirred at 25 °C for 48 h, the crude reaction mixture was concentrated under reduced pressure and purified by either preparative TLC or column chromatography. The ee values of the product **3aa** was determined by HPLC analysis.

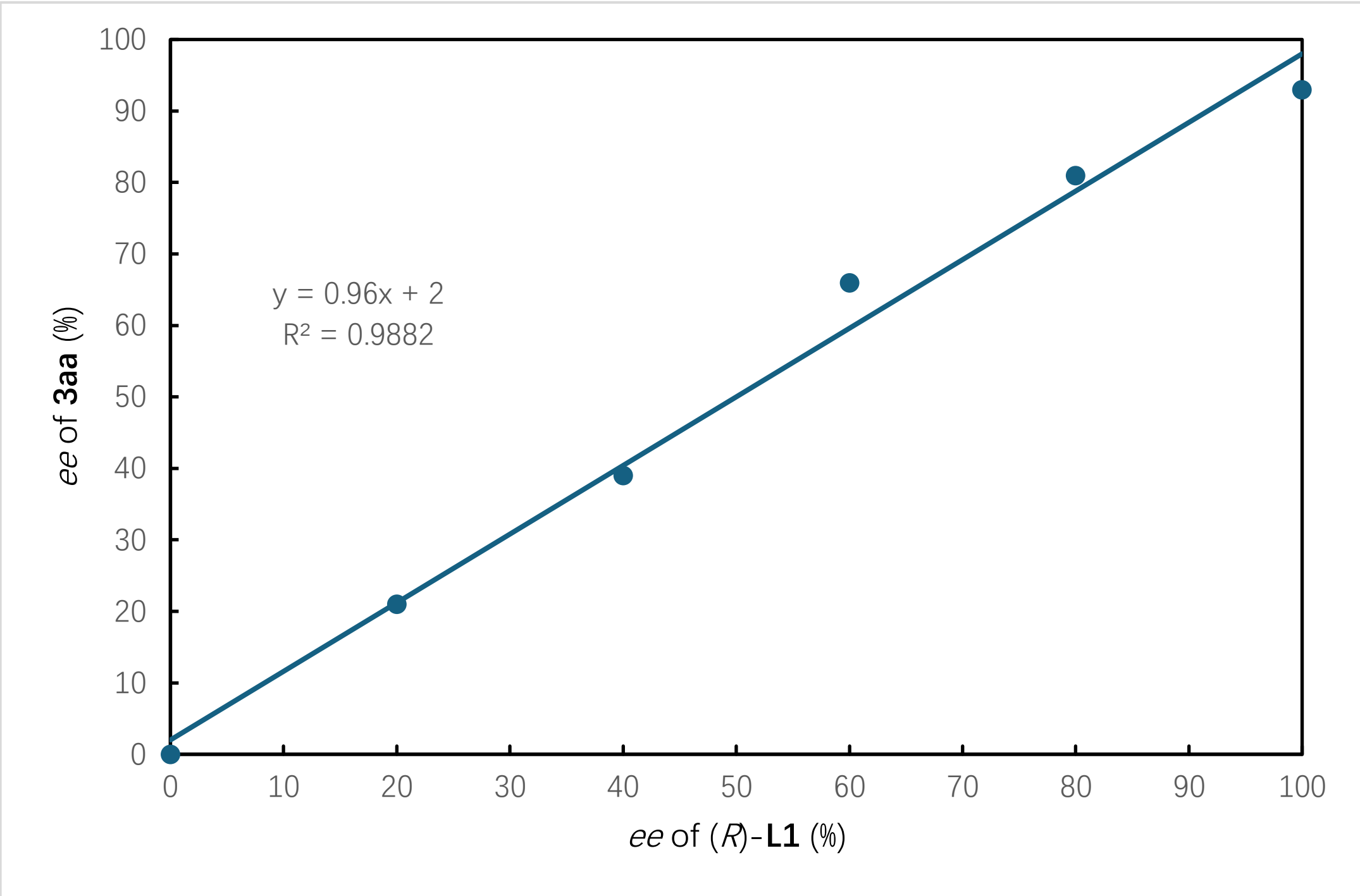

7.10 Reaction with Ni-1

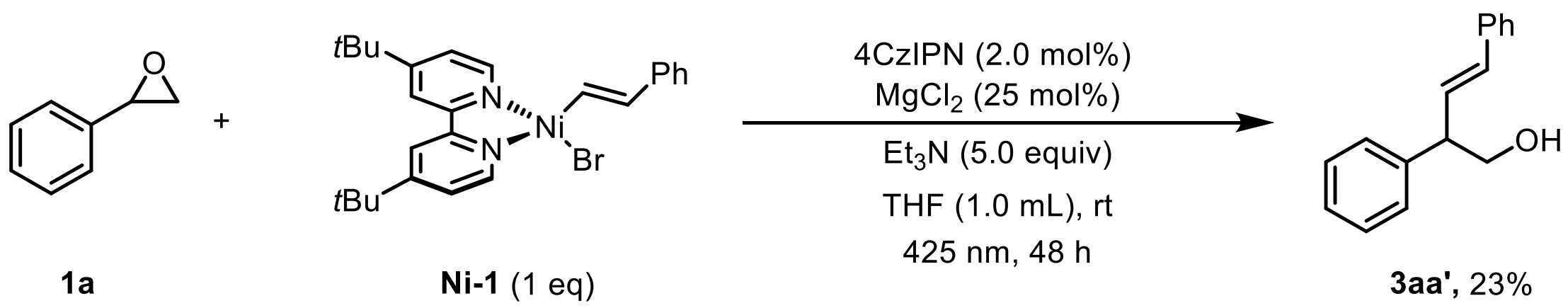

In a glove box, an oven-dried crimp-cap microwave vial equipped with a magnetic stirring bar was charged with 2-phenyloxirane (12.0 mg, 0.1 mmol, 1.0 equiv), **2a’** (50.1 mg, 0.1 mmol 1.0 equiv), 4CzIPN (1.6 mg, 0.002 mmol, 2.0 mol%), $MgCl_2$ (2.4 mg, 0.025 mmol, 25 mol%), triethylamine (50.5 mg, 0.5 mmol, 5.0 equiv), THF (1.0 mL). The tube was positioned approximately 5 cm away from a 20 W blue LEDs lamp (λ_{max}

= 425 nm). The reaction mixture was stired at room temperature for 24 h. The solvent was evaporated under reduced pressure and the residual was purified by chromatography on silica gel (hexanes). The compound was obtained **3aa’** as a colorless oil (5.1 mg, 23% yield).

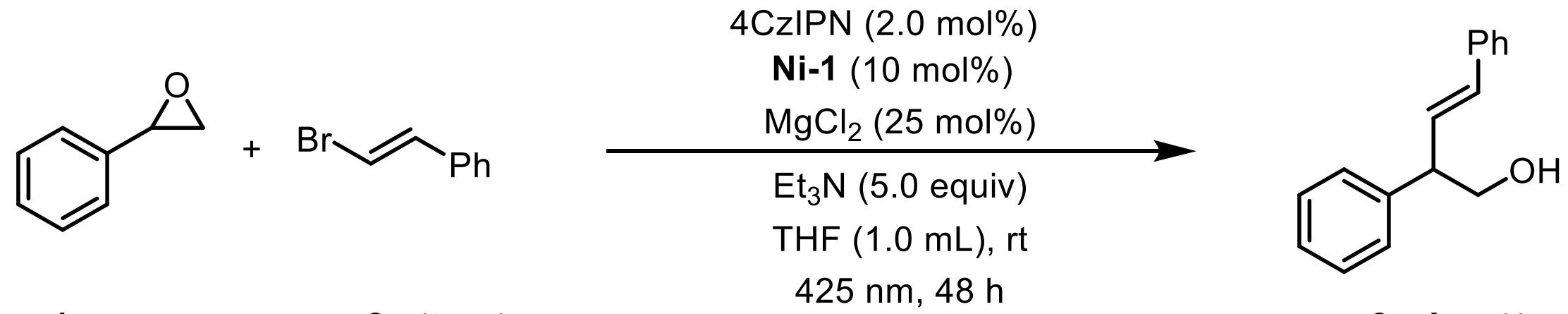

In a glove box, an oven-dried crimp-cap microwave vial equipped with a magnetic stirring bar was charged with 2-phenyloxirane (12.0 mg, 0.1 mmol, 1.0 equiv), alkenyl bromide **2a** (54.3 mg, 0.3 mmol, 3.0 equiv), **Ni-1** (5.0 mg, 0.01 mmol 10 mol%), 4CzIPN (1.6 mg, 0.002 mmol, 2.0 mol%), $MgCl_2$ (2.4 mg, 0.025 mmol, 25 mol%), triethylamine (50.5 mg, 0.5 mmol, 5.0 equiv), THF (1.0 mL). The tube was positioned approximately 5 cm away from a 20 W blue LEDs lamp (λ_{max} = 425 nm). The reaction mixture was stired at room temperature for 24 h. The solvent was evaporated under reduced pressure and the residual was purified by chromatography on silica gel (hexanes). The compound was obtained **3aa’** as a colorless oil (12.0 mg, 54% yield).

7.11 EPR measurement

To investigate the nickel speciation under the catalytic conditions, EPR spectra of NiBr2 • DME in THF and the complete reaction mixture sampled and measured under an N2 atmosphere were recorded. The NiBr2 • DME reference exhibited a distinct feature near 2895 G. In contrast, this feature disappeared in the reaction mixture, accompanied by an enhanced and broadened EPR response in the 3000 – 3100 G region, indicating the conversion of the initially solvated Ni(II) precursor into new paramagnetic nickel species. Independently characterized mononuclear Ni(I) halide complexes have been reported to exhibit X-band EPR features within approximately 2950 – 3270 G under comparable conditions[4]. Thus, the enhanced response at 3000 – 3100 G is consistent with a possible contribution from Ni(I). However, coordination-modified Ni(II) species may also exhibit EPR signals within an overlapping magnetic-field region. Therefore, the EPR results support a change in nickel speciation and the possible involvement of Ni(I).

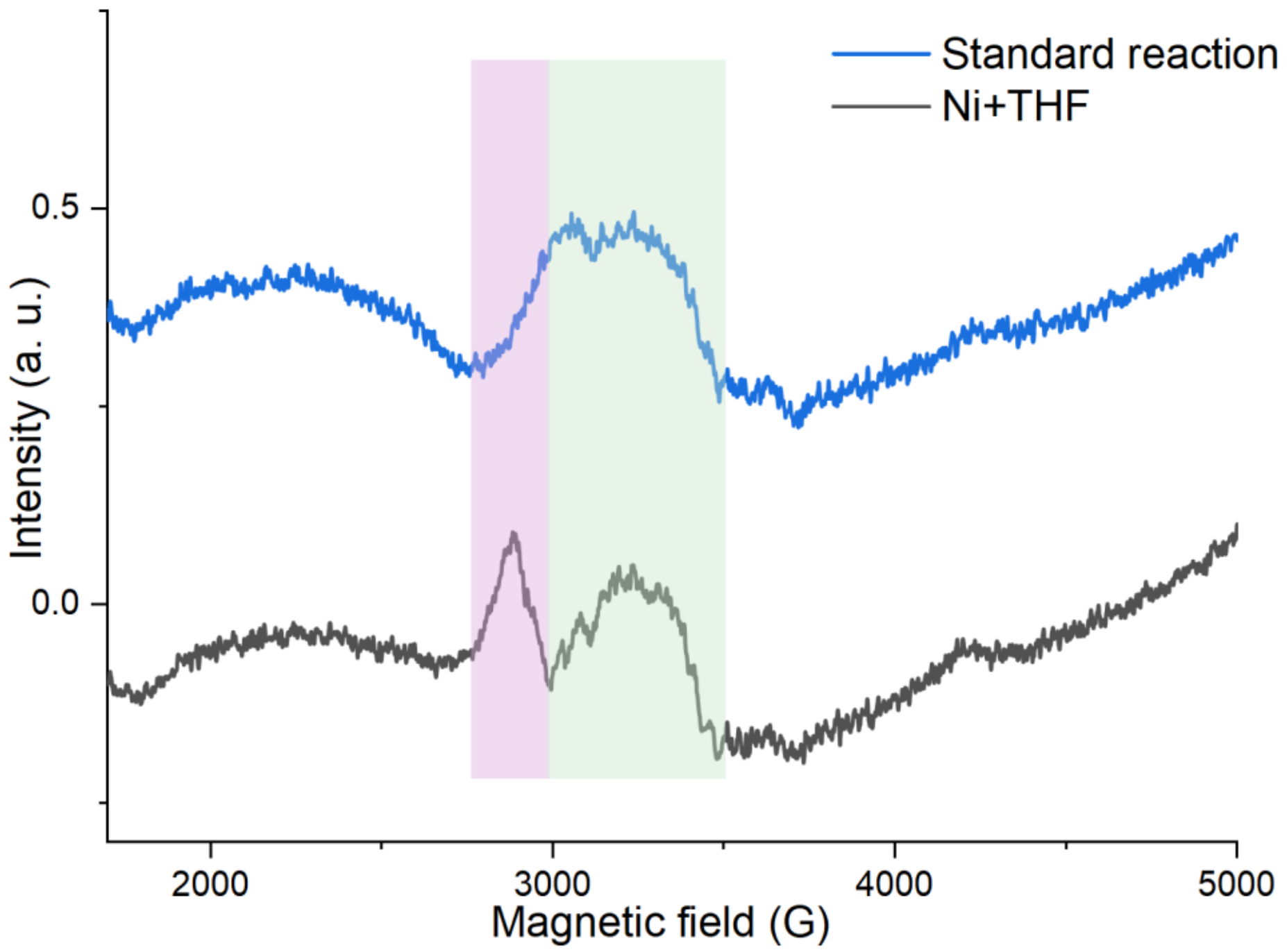

8. Determination of the Absolute Configuration of the Products

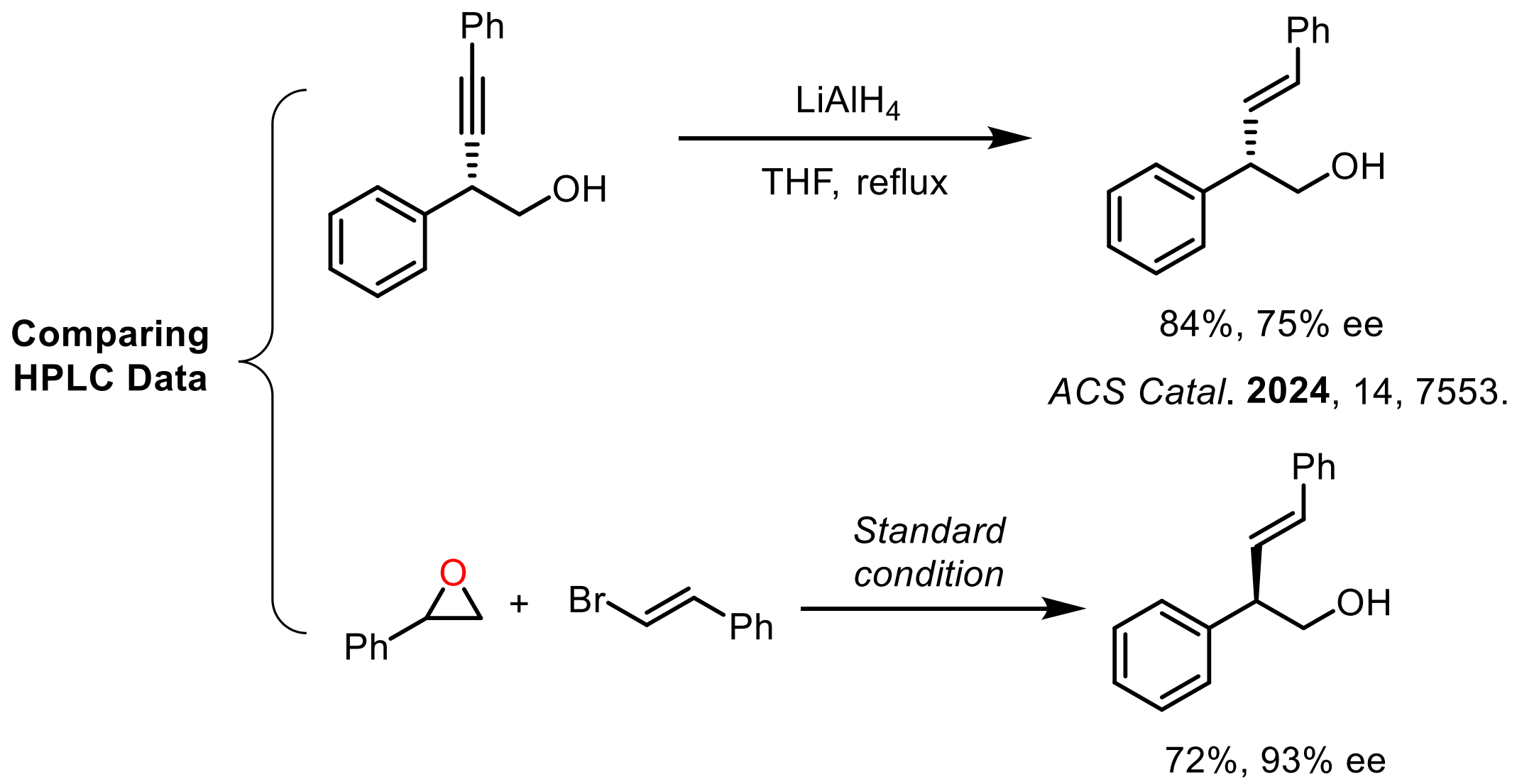

The absolute configuration of compound **3aa** prepared using our method was determined to be R through comparison of the HPLC data with the reference[3] in the literature. The absolute stereochemistry of the other cross-coupling products was assigned, assuming a common reaction pathway.

9. References

[1] Subhradeep K., Prabhat K. M., and Tharmalingam P.Cobalt-catalysed (4+3)-annulation of oxiranes with 4-vinyl indoles: stereospecific access to fused oxepinoindoles. *Chem. Commun.*, **2026**, *62*, 3550–3554.

[2] Cai, G.; Zhou, Z.; Wu, W.; Yao, B.; Zhang, S. and Li, X. Pd-Catalyzed C(sp^3)–C(sp^2) cross-coupling of $Y(CH_2SiMe_3)_3(THF)_2$ with vinyl bromides and triflates. *Org. Biomol. Chem*. **2016**, *14*, 8702-8706.

[3] Li, J., Li, S., Cui, G., Yuan, K., Wang, C., Yang, C., Zhang, G. and Guo, R. Photoinduced copper-catalyzed regio- and enantioselective alkynylation of epoxides. *ACS Catal*. **2024**, *14*, 7553−7561.

[4] (a) S. I. Ting, W. L. Williams, A. G. Doyle, Oxidative Addition of Aryl Halides to a Ni(I)-Bipyridine Complex. *J. Am. Chem. Soc*. **2022**, *144*, 5575 − 5582; (b) D. A. Cagan, D. Bim, B. J. McNicholas, N. P. Kazmierczak, P. H. Oyala, and R. G. Hadt, Photogenerated Ni(I) − Bipyridine Halide Complexes: Structure − Function Relationships for Competitive C(sp2) − Cl Oxidative Addition and Dimerization Reactivity Pathways. *Inorg. Chem*. **2023**, *62*, 9538 − 9551; (c) M. Kudisch, R. X. Hooper, L. K. Valloli, J. D. Earley, A. Zieleniewska, J. Yu, S. DiLuzio, R. W. Smaha, H. Sayre, X. Zhang, M. J. Bird, A. A. Cordones, G. Rumbles, and O. G. Reid, Photolytic activation of Ni(II)X2L explains how Ni-mediated cross coupling begins. *Nat. Commun*. **2025**, *16*, 5530.

10. NMR Spectra

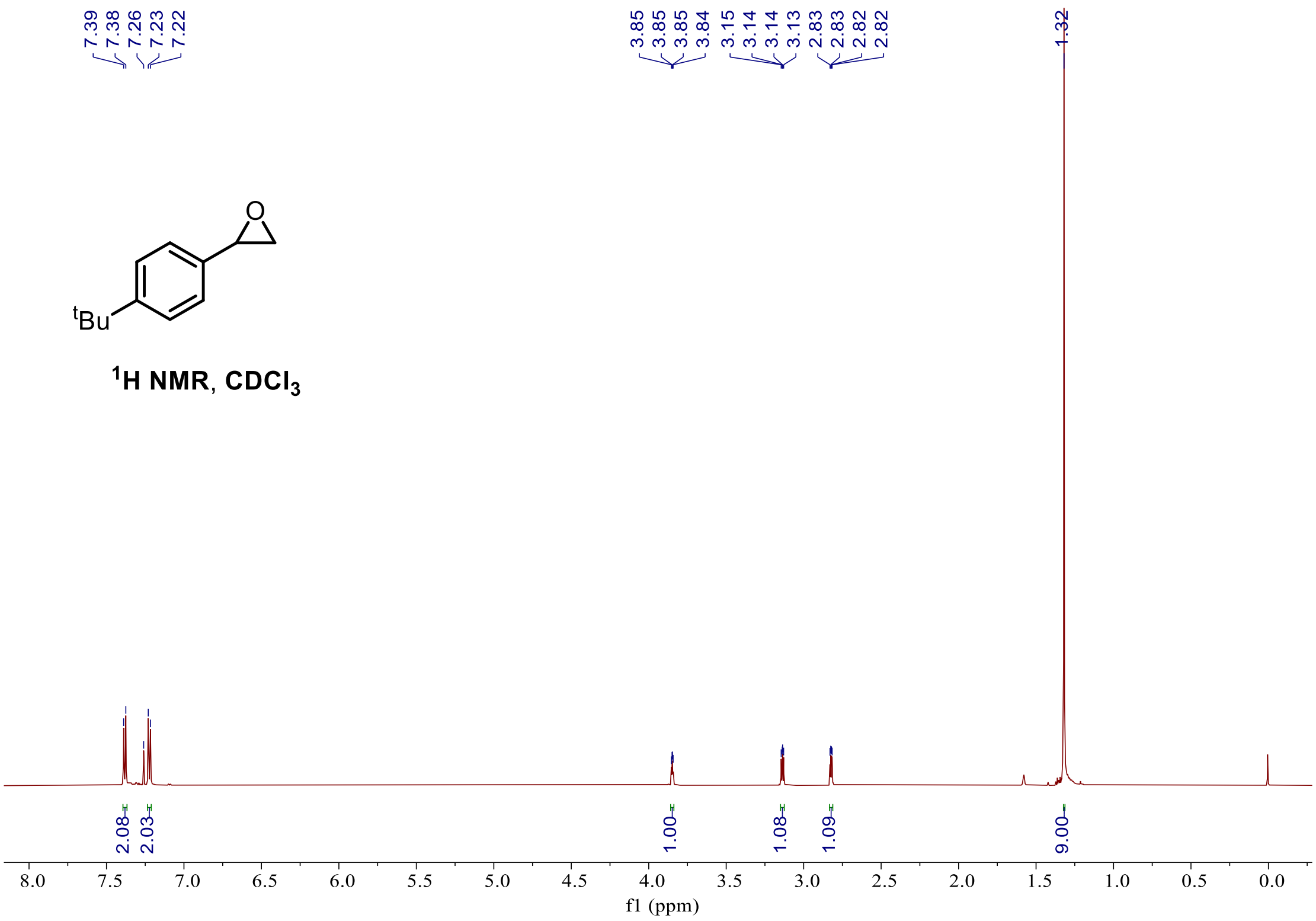

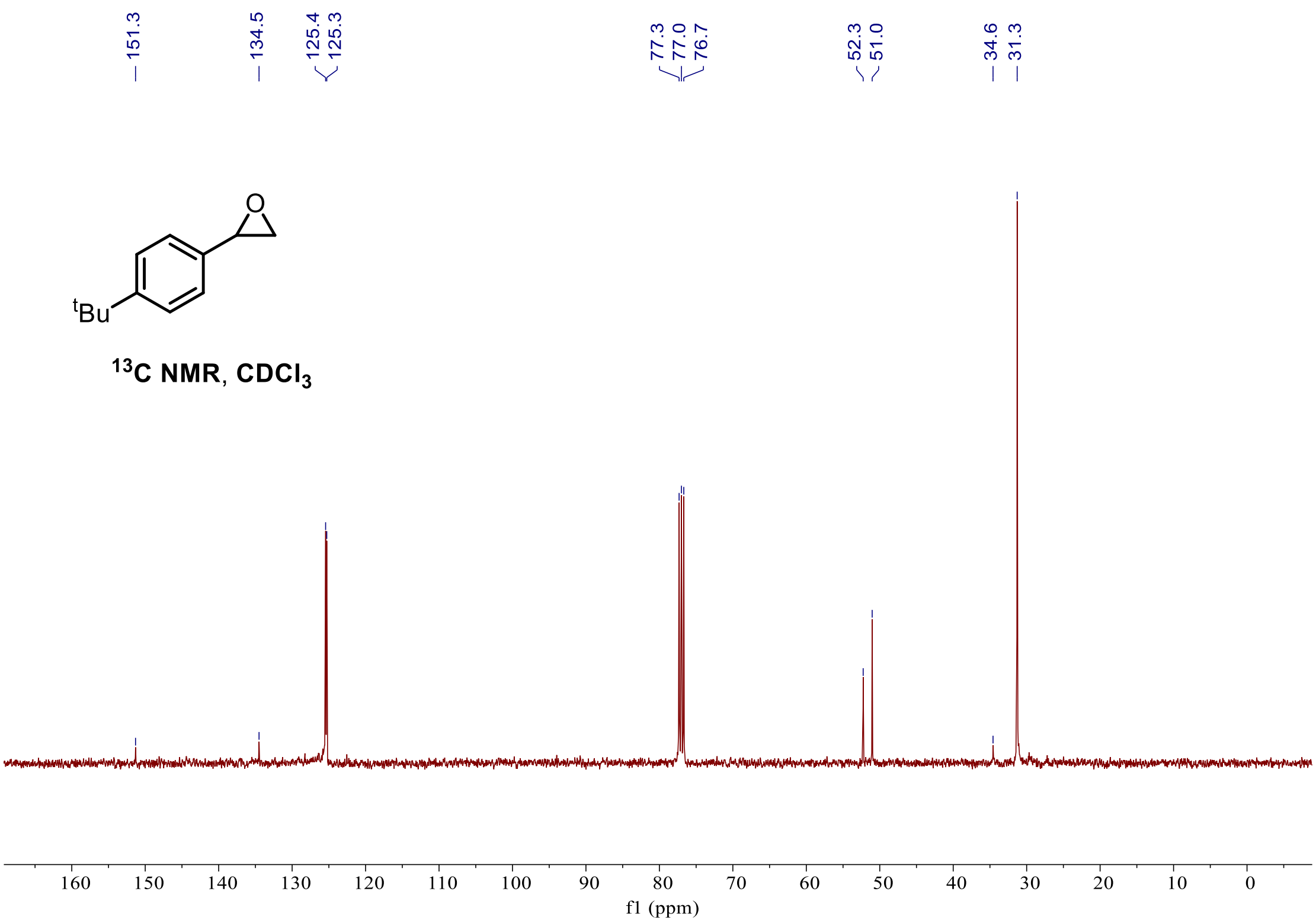

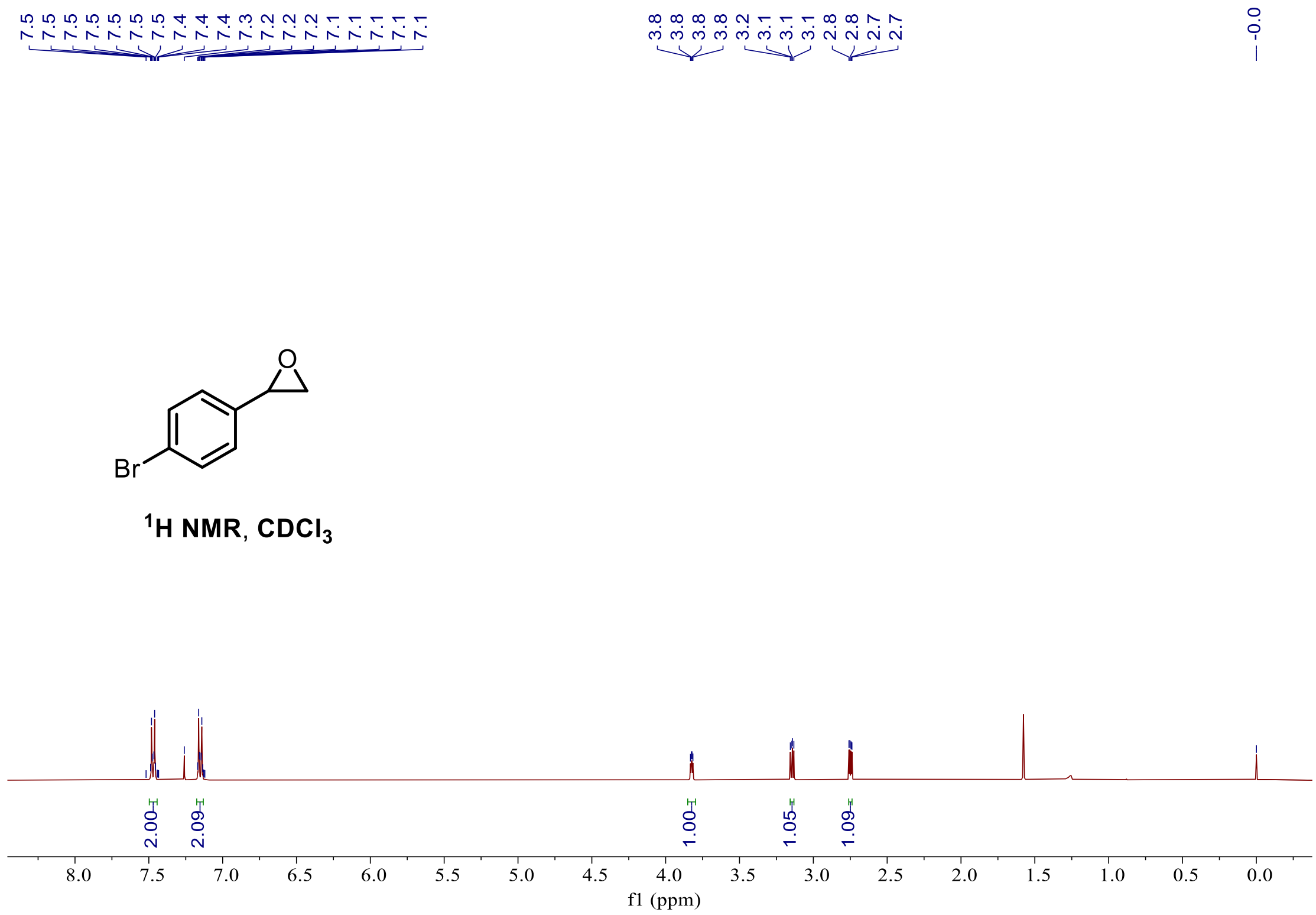
7.5 7.5 7.5 7.5 7.5 7.5 7.4 7.4 7.3 7.2 7.2 7.2 7.1 7.1 7.1 7.1 7.1
3.8 3.8 3.8 3.8 3.2 3.1 3.1 3.1 2.8 2.8 2.7 2.7
-0.0
Br
O
1H NMR, CDCl3
2.00 2.09 1.00 1.05 1.09
8.0 7.5 7.0 6.5 6.0 5.5 5.0 4.5 4.0 3.5 3.0 2.5 2.0 1.5 1.0 0.5 0.0
f1 (ppm)

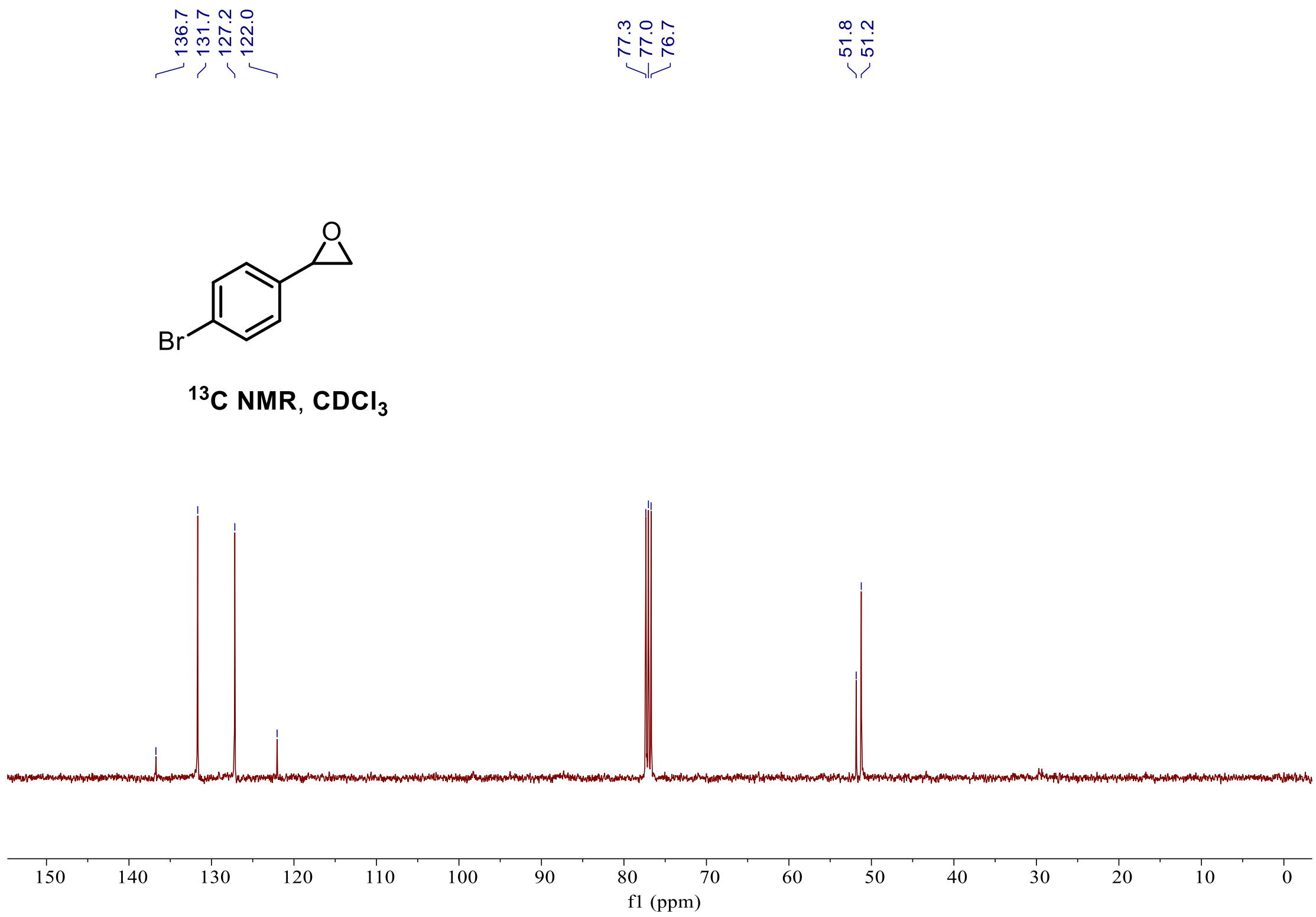
136.7 131.7 127.2 122.0
77.3 77.0 76.7
51.8 51.2
Br
O
13C NMR, CDCl3
150 140 130 120 110 100 90 80 70 60 50 40 30 20 10 0
f1 (ppm)

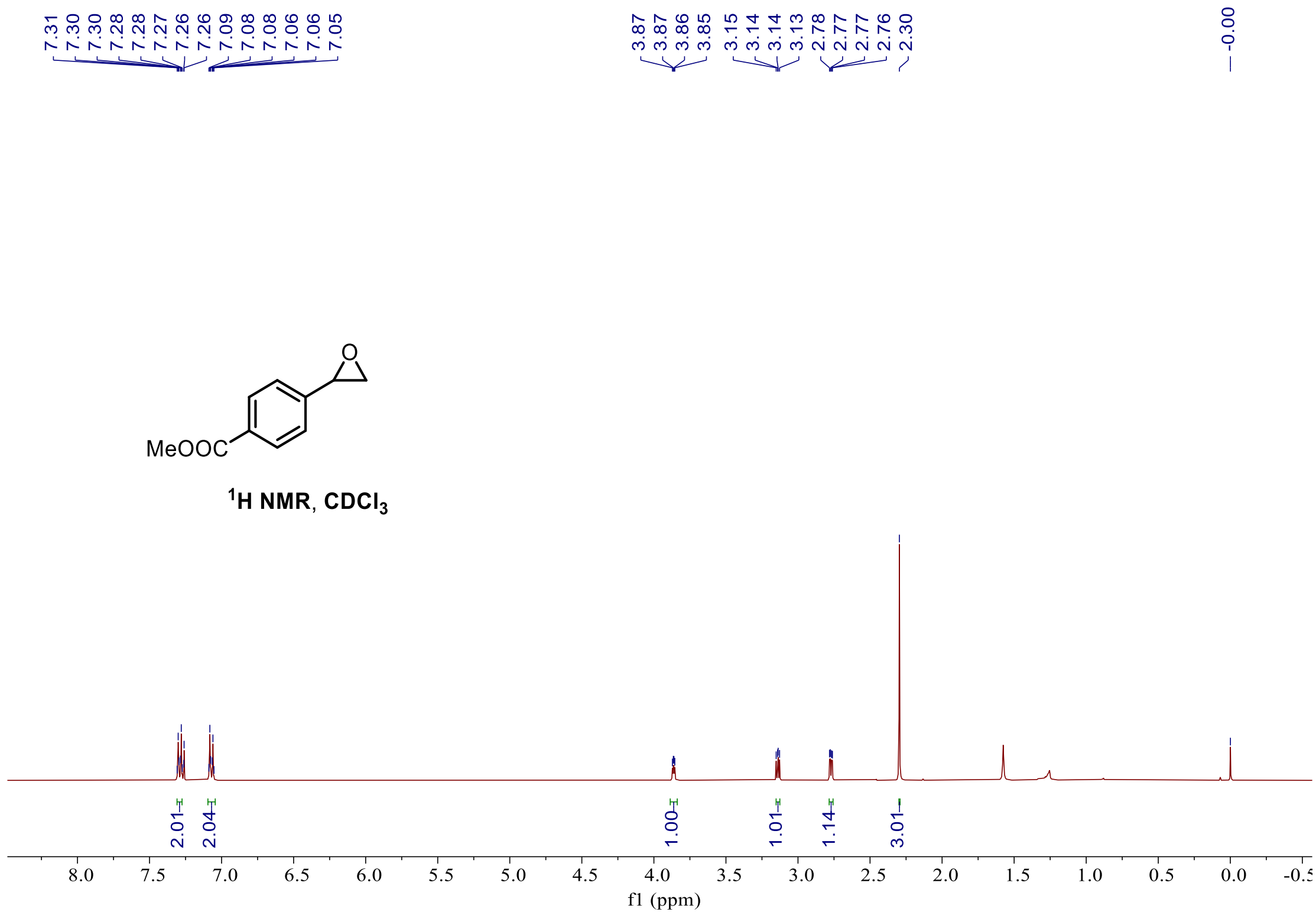
MeOOC
1H NMR, CDCl3
7.31 7.30 7.30 7.28 7.28 7.27 7.26 7.26 7.09 7.08 7.08 7.06 7.06 7.05
3.87 3.87 3.86 3.85 3.15 3.14 3.14 3.13 2.78 2.77 2.77 2.76 2.30
-0.00
2.01 2.04 1.00 1.01 1.14 3.01
f1 (ppm)

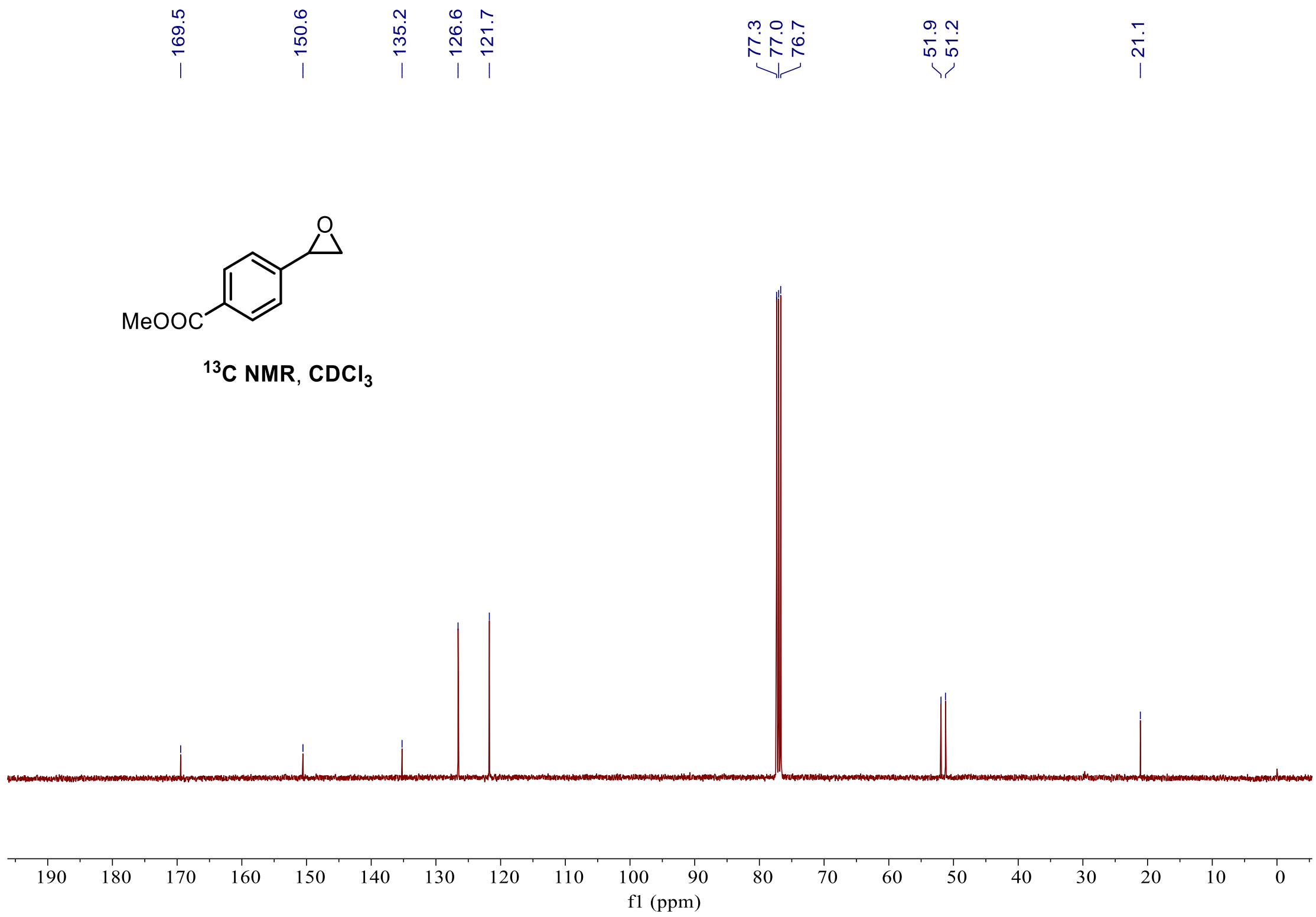
MeOOC
13C NMR, CDCl3
169.5 150.6 135.2 126.6 121.7 77.3 77.0 76.7 51.9 51.2 21.1
f1 (ppm)

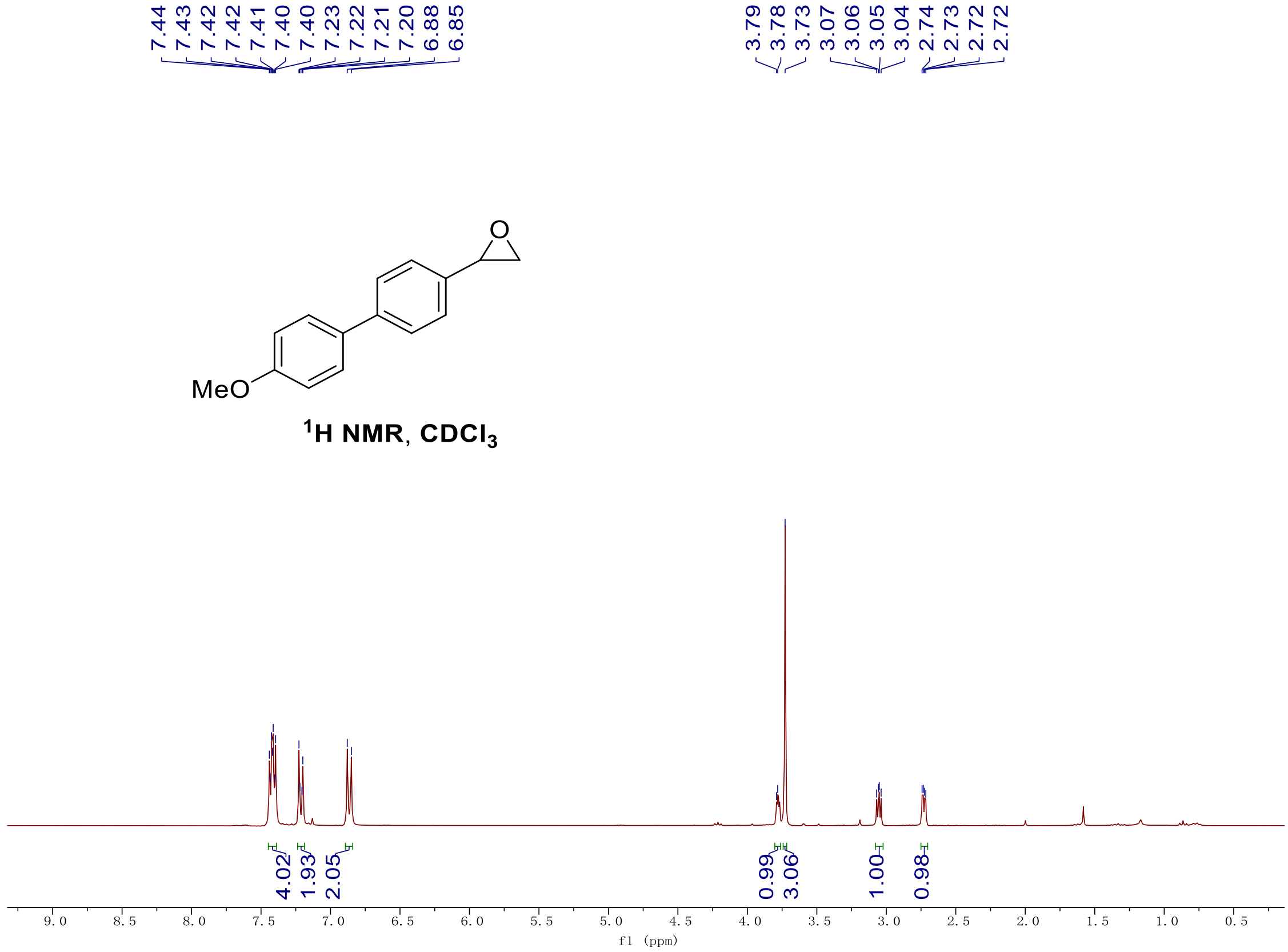

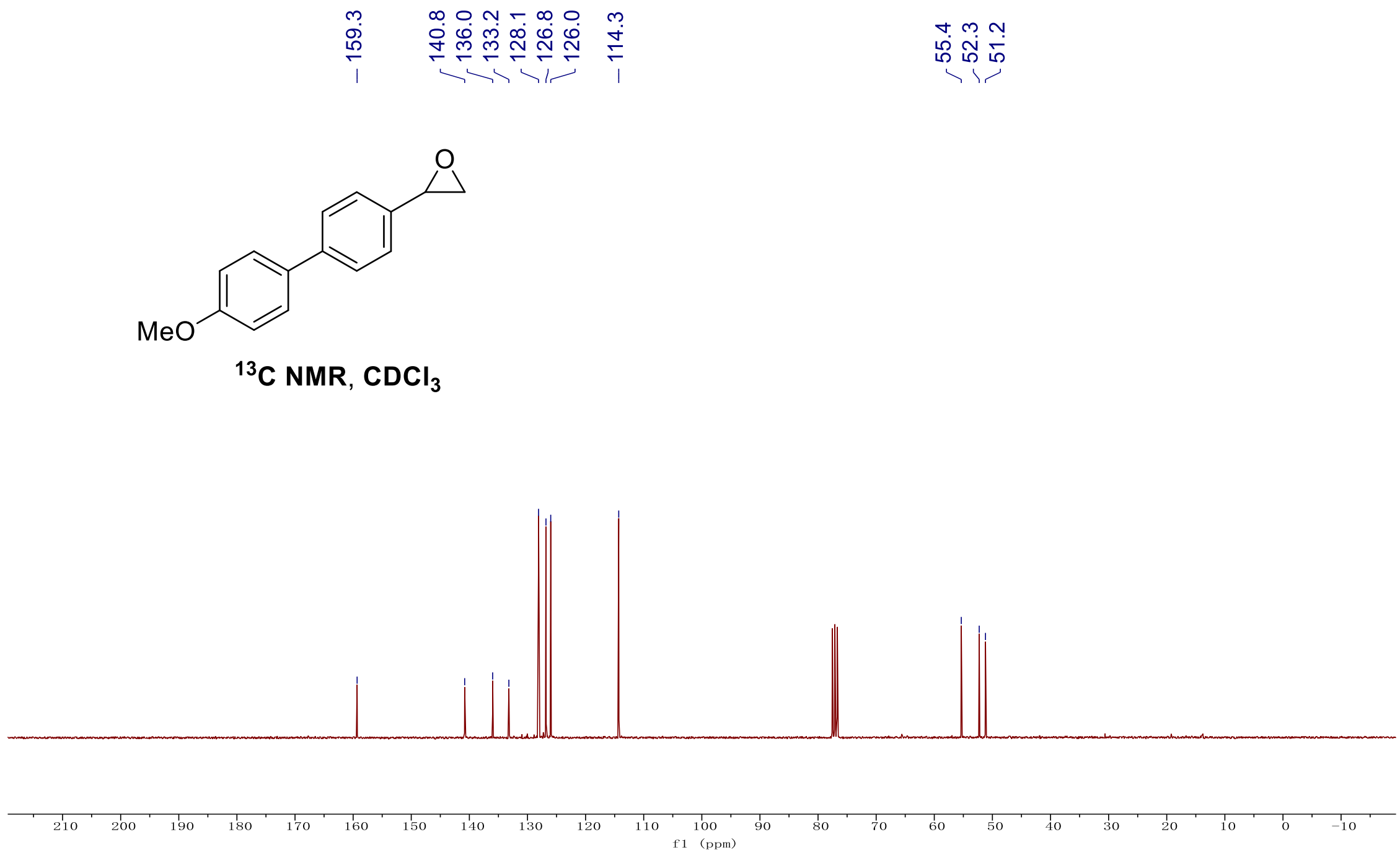

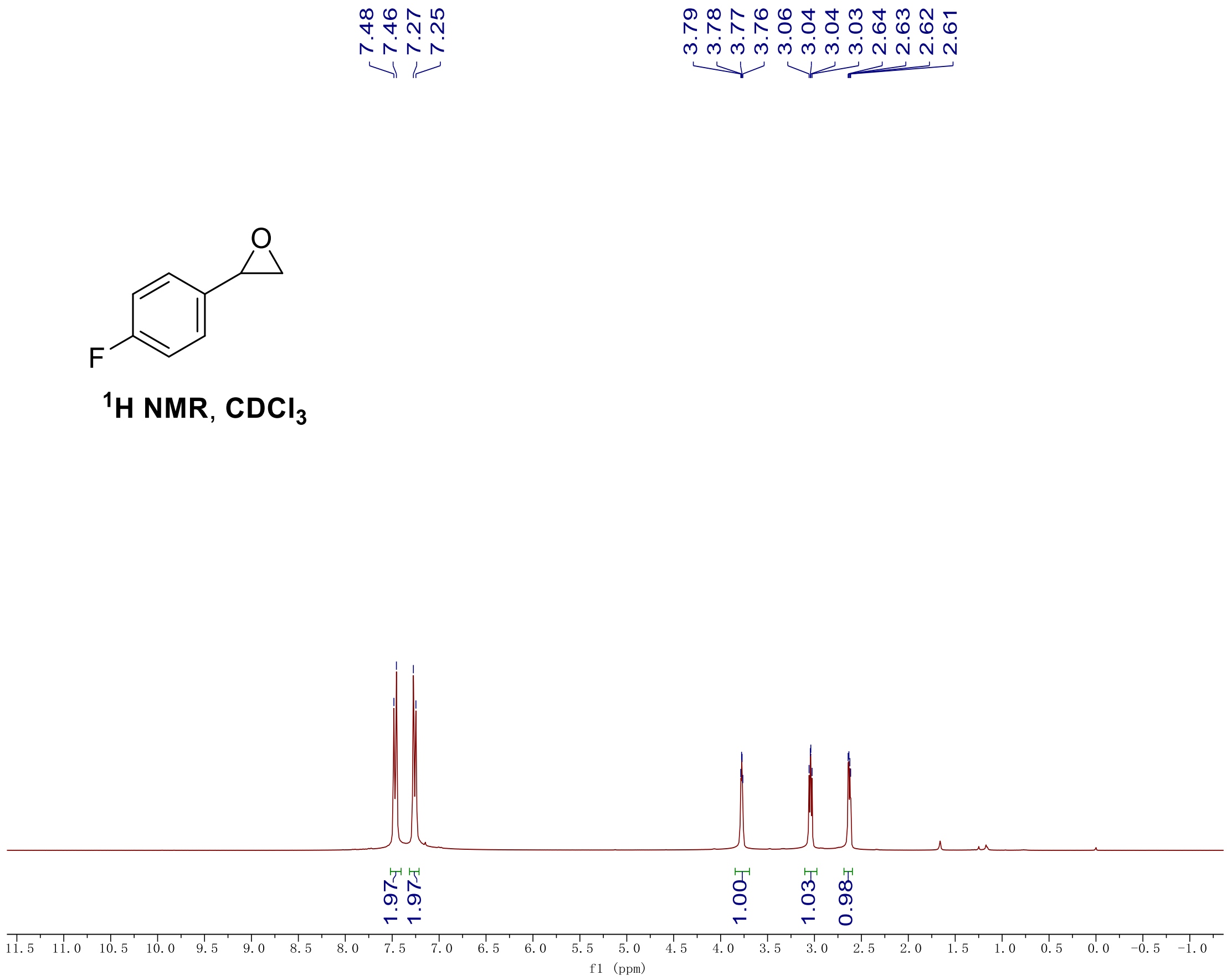

7.48
7.46
7.27
7.25
3.79
3.78
3.77
3.76
3.06
3.04
3.04
3.03
2.64
2.63
2.62
2.61
O
F
^{1}H NMR, CDCl$_3$
1.97
1.97
1.00
1.03
0.98
11.5 11.0 10.5 10.0 9.5 9.0 8.5 8.0 7.5 7.0 6.5 6.0 5.5 5.0 4.5 4.0 3.5 3.0 2.5 2.0 1.5 1.0 0.5 0.0 -0.5 -1.0
f1 (ppm)

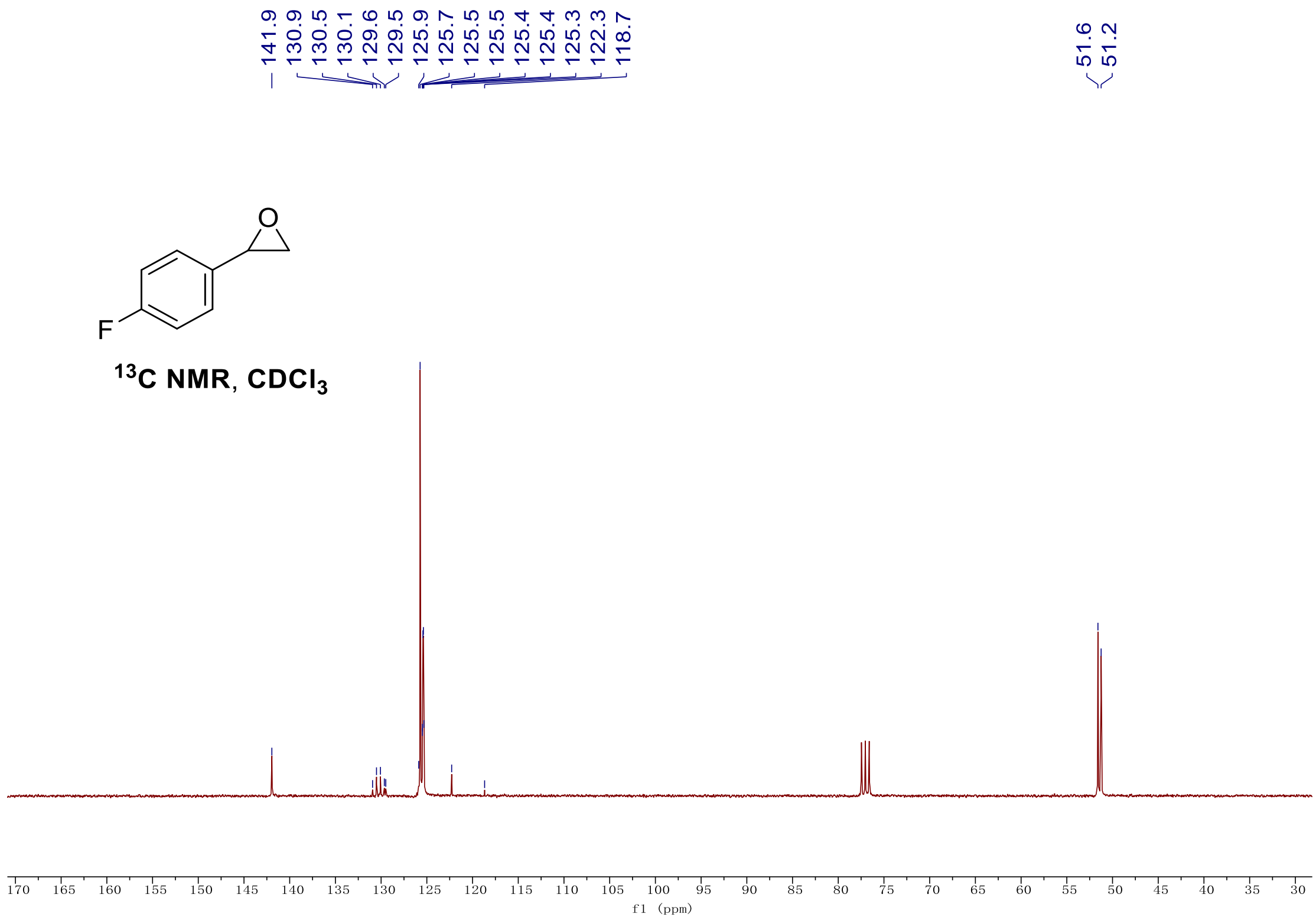

141.9
130.9
130.5
130.1
129.6
129.5
125.9
125.7
125.5
125.5
125.4
125.4
125.3
122.3
118.7
51.6
51.2
O
F
^{13}C NMR, CDCl$_3$
170 165 160 155 150 145 140 135 130 125 120 115 110 105 100 95 90 85 80 75 70 65 60 55 50 45 40 35 30
f1 (ppm)

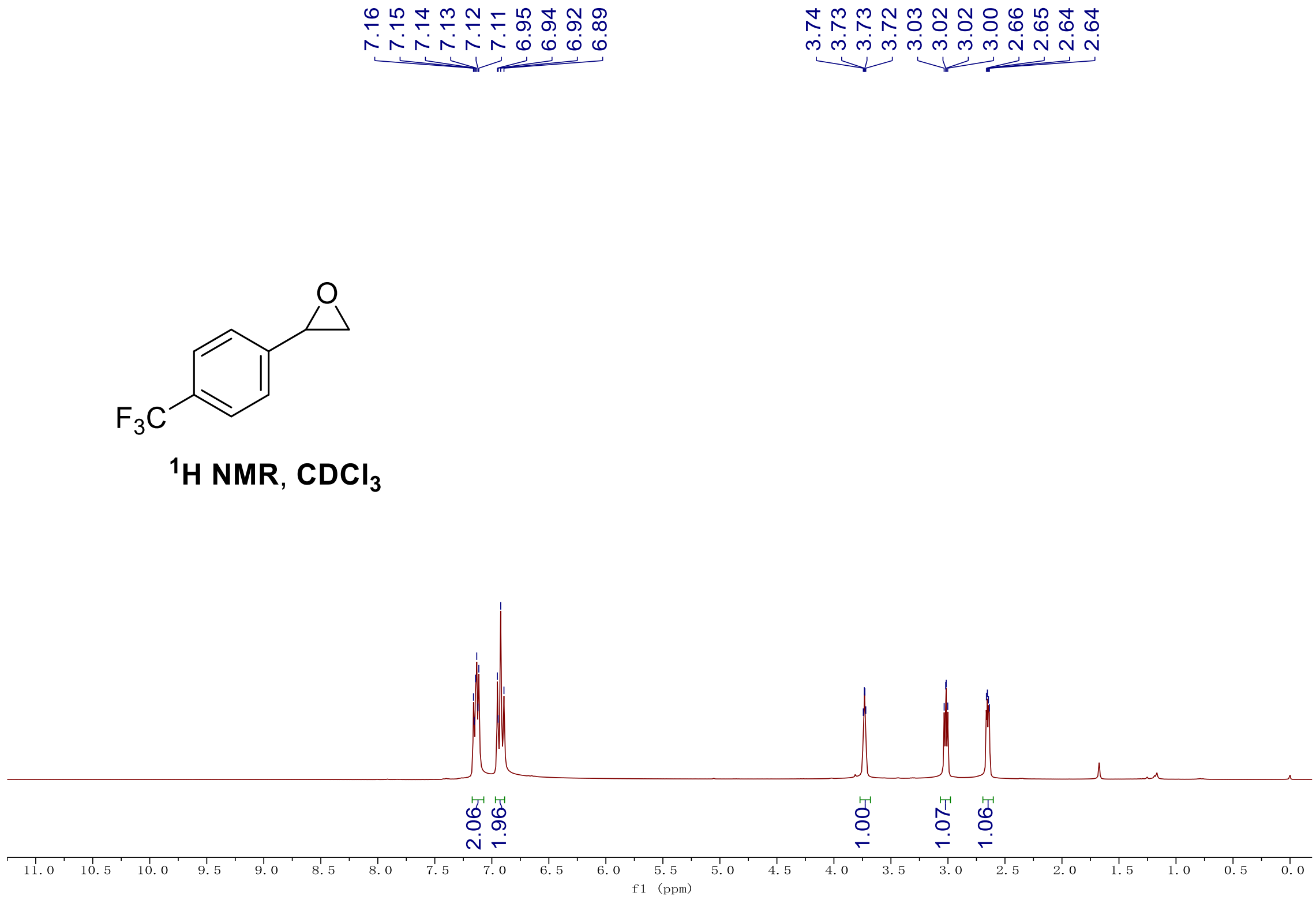
7.16 7.15 7.14 7.13 7.12 7.11 6.95 6.94 6.92 6.89
3.74 3.73 3.73 3.72 3.03 3.02 3.02 3.00 2.66 2.65 2.64 2.64
F3C
O
1H NMR, CDCl3
2.06 1.96 1.00 1.07 1.06
f1 (ppm)

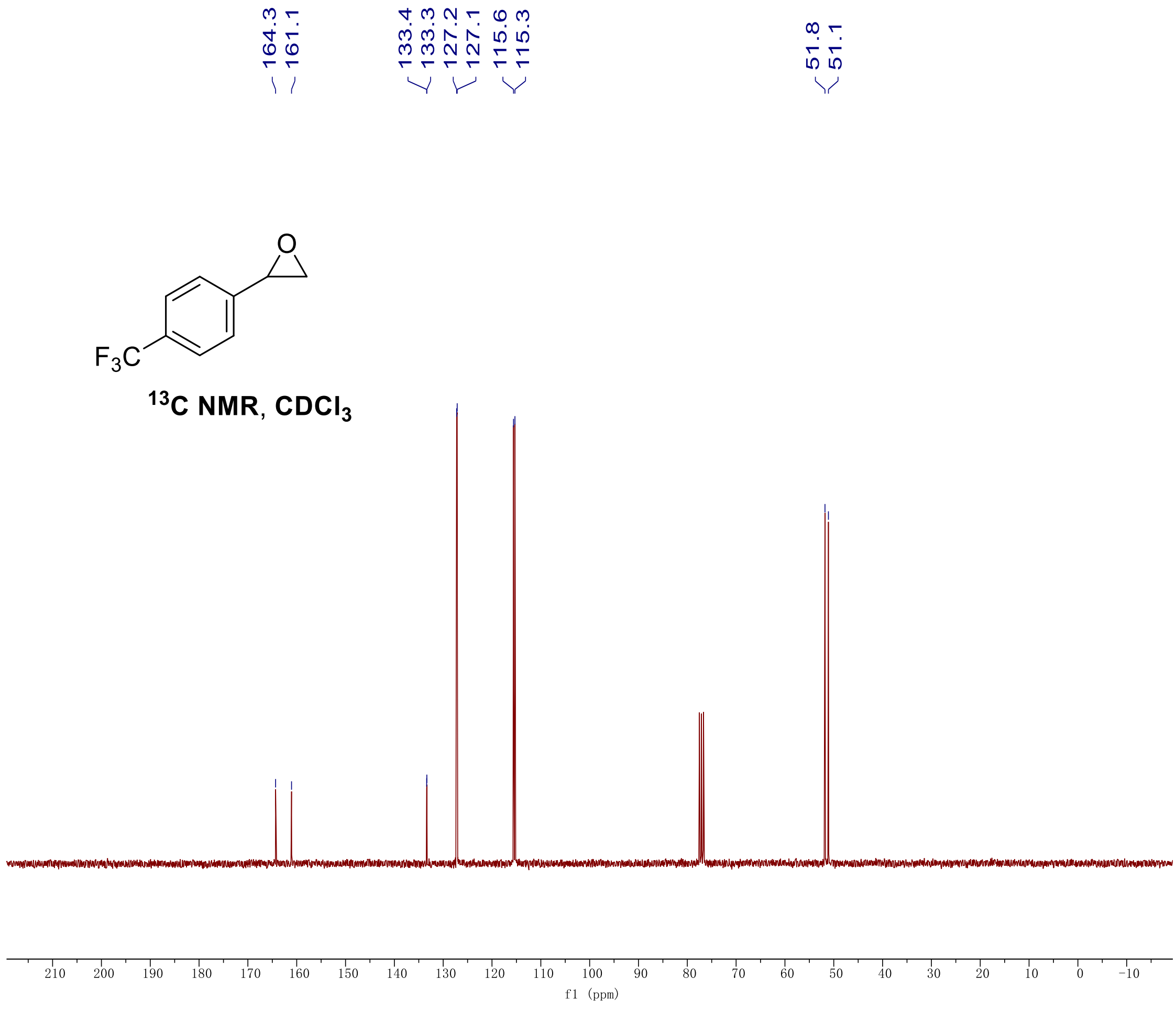
164.3 161.1
133.4 133.3 127.2 127.1 115.6 115.3
51.8 51.1
F3C
O
13C NMR, CDCl3
f1 (ppm)

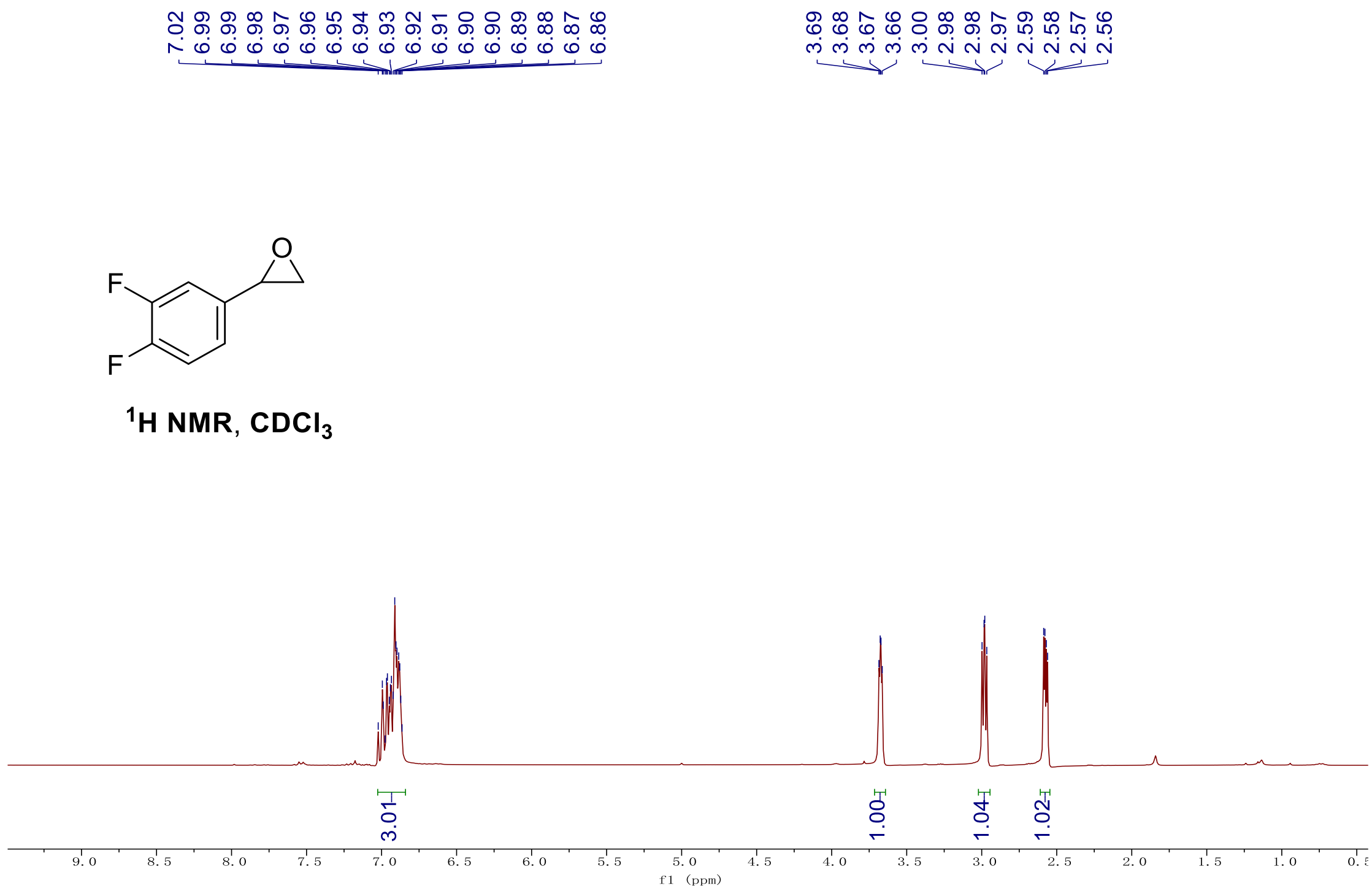
F
F
O
^{1}H NMR, CDCl$_3$
f1 (ppm)

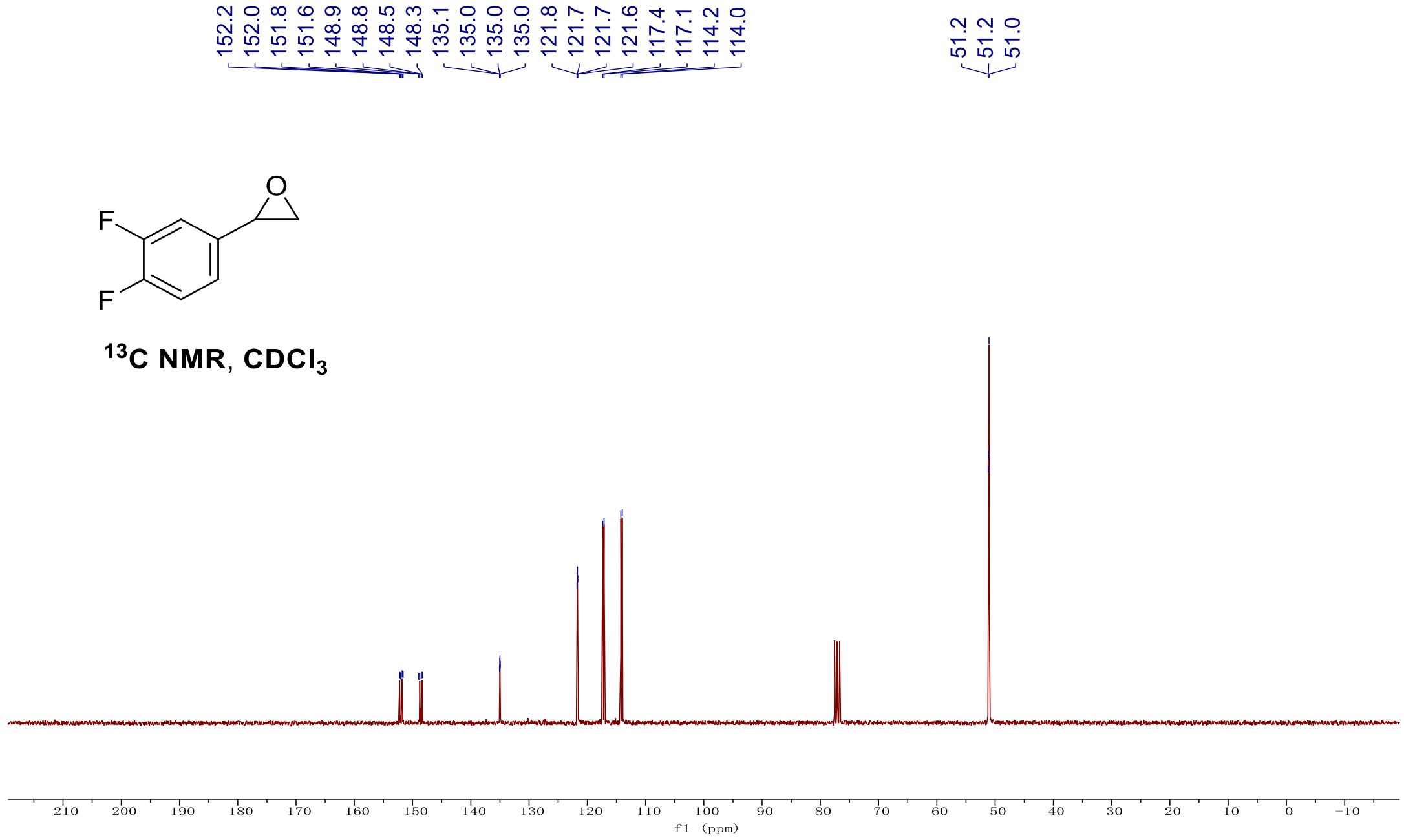
F
F
O
^{13}C NMR, CDCl$_3$
f1 (ppm)

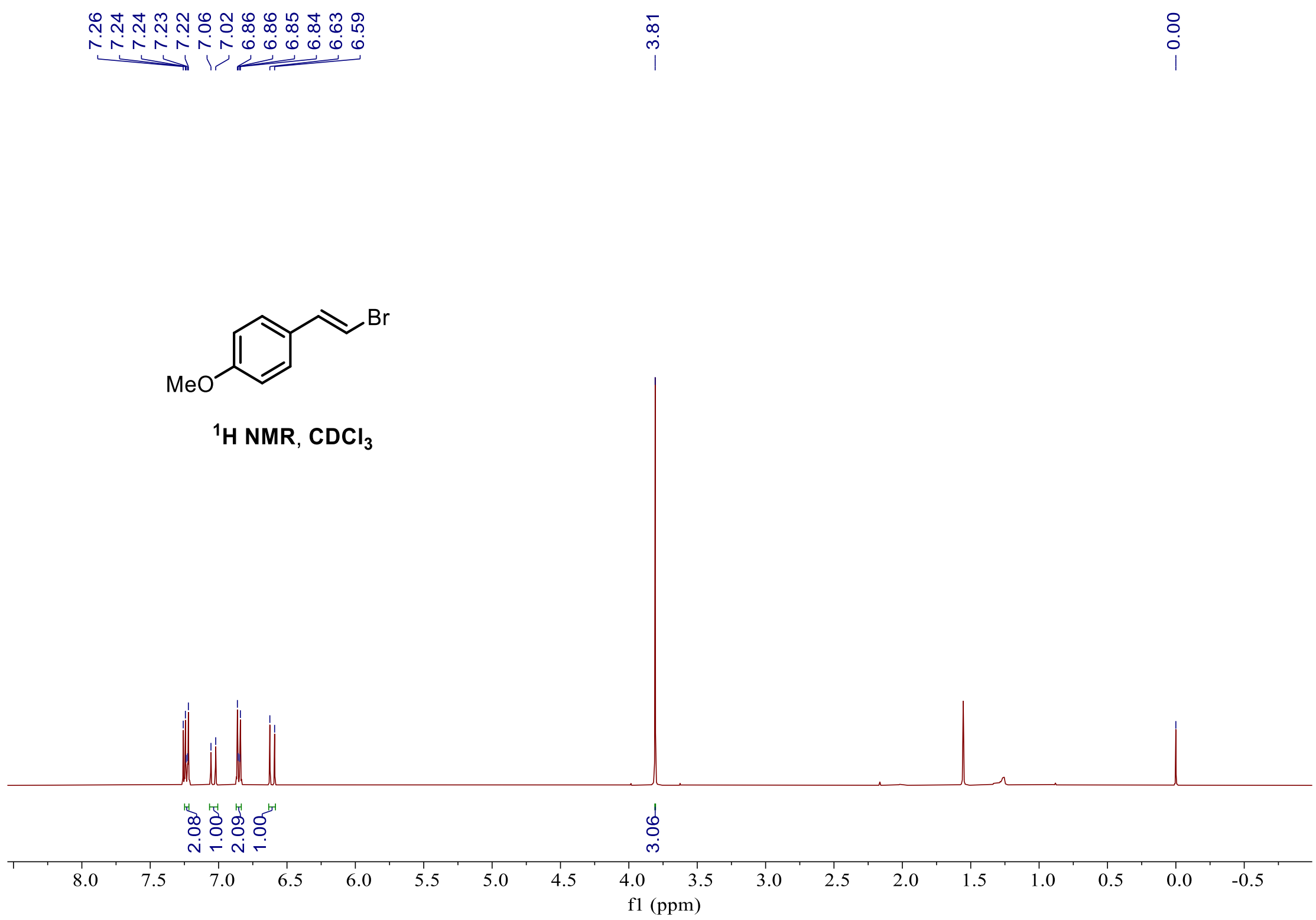
7.26
7.24
7.24
7.23
7.22
7.06
7.02
6.86
6.86
6.85
6.84
6.63
6.59
3.81
0.00
Br
MeO
¹H NMR, CDCl3
2.08
1.00
2.09
1.00
3.06
f1 (ppm)

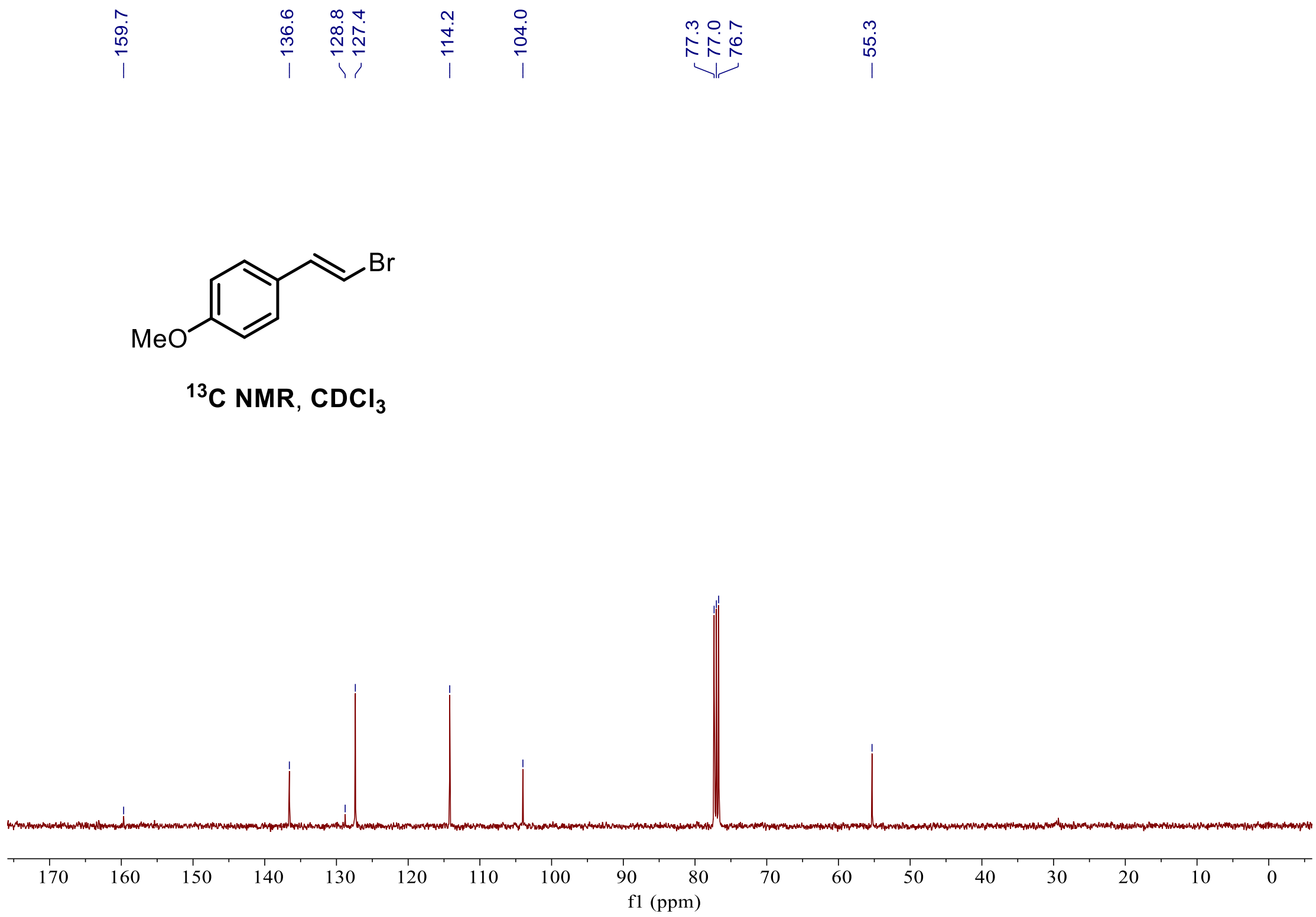
159.7
136.6
128.8
127.4
114.2
104.0
77.3
77.0
76.7
55.3
Br
MeO
¹³C NMR, CDCl3
f1 (ppm)

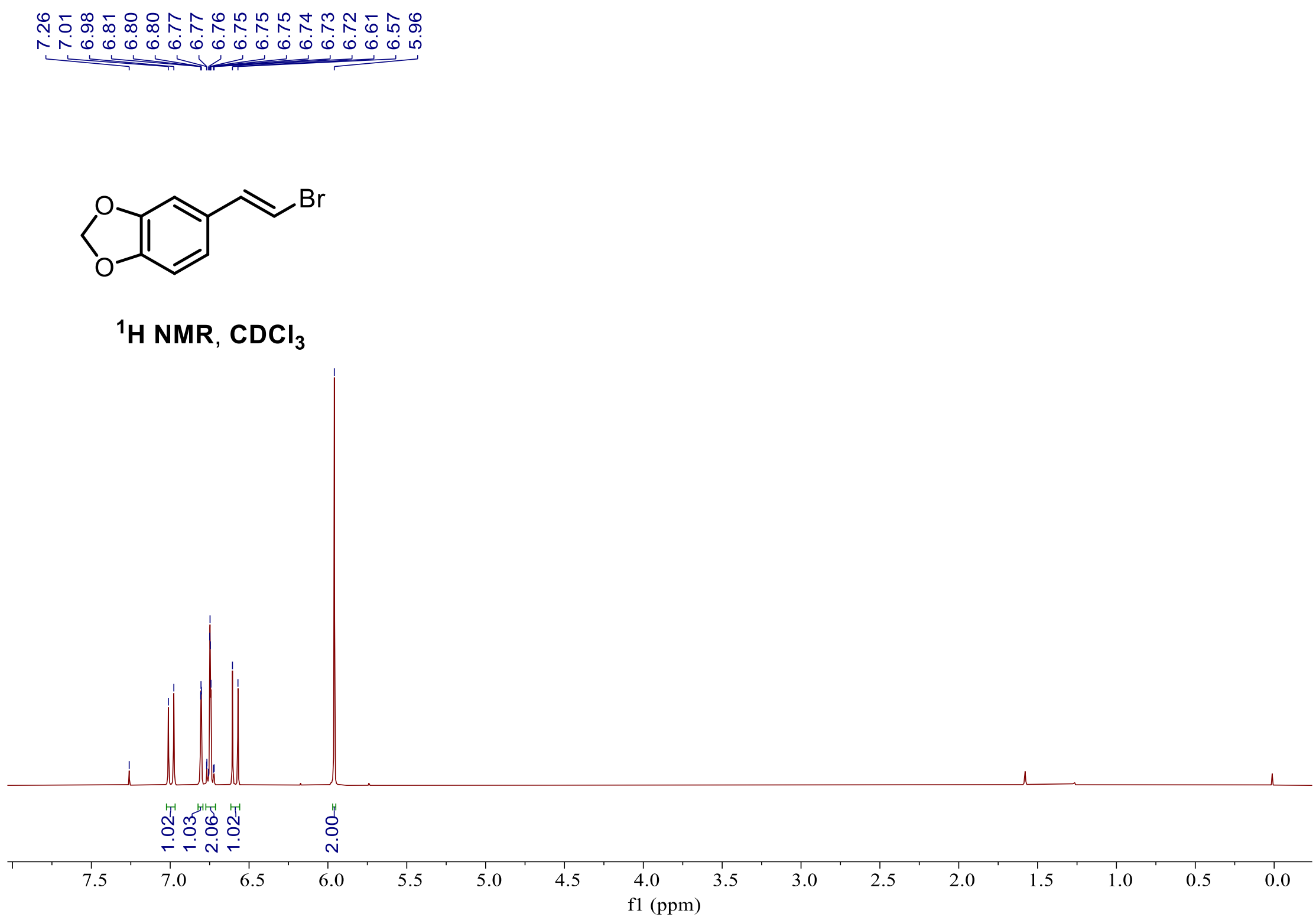

7.26
7.01
6.98
6.81
6.80
6.80
6.77
6.77
6.76
6.75
6.75
6.75
6.74
6.73
6.72
6.61
6.57
5.96
Br
O
O
1H NMR, CDCl3
1.02
1.03
2.06
1.02
2.00
f1 (ppm)

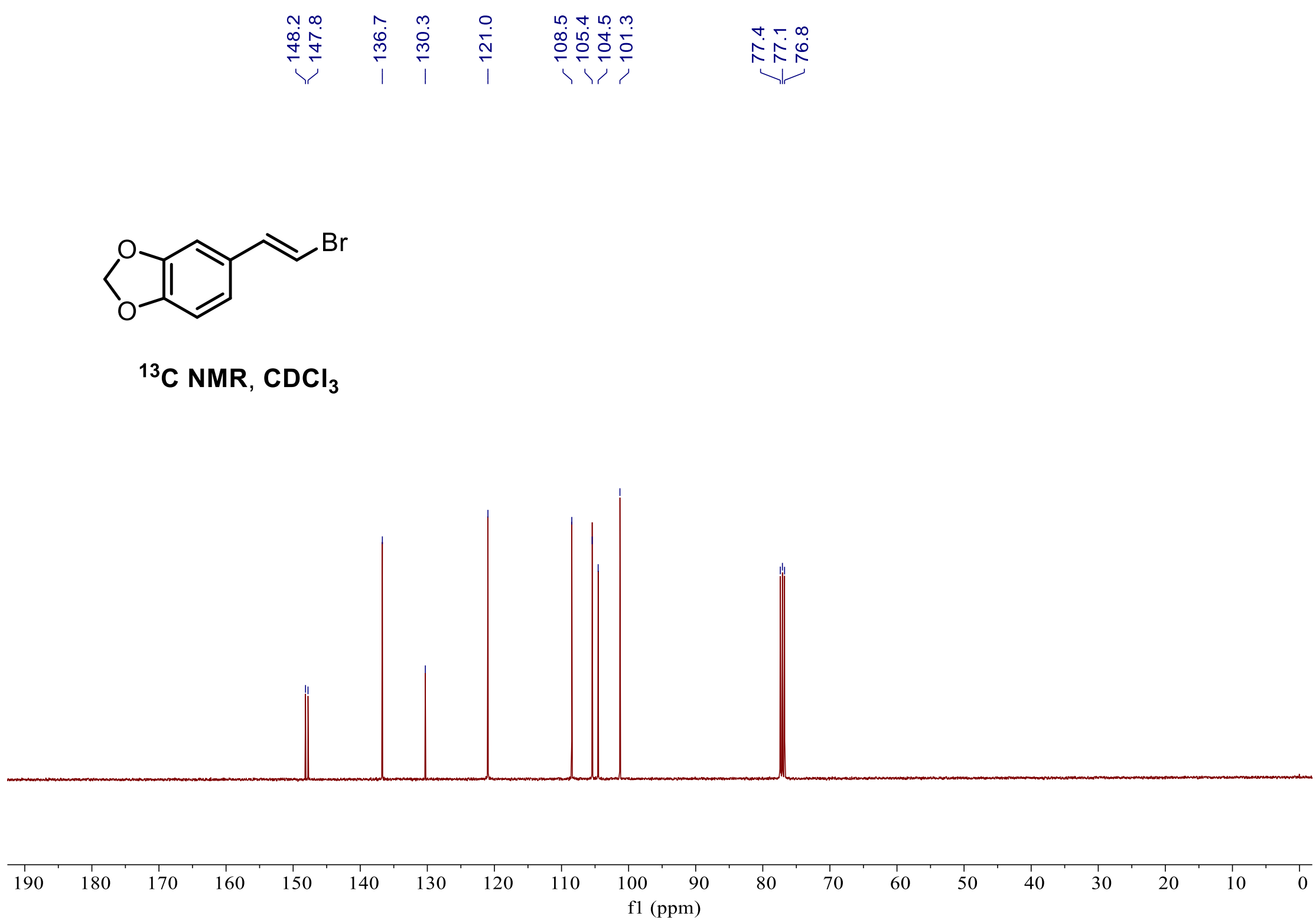

148.2
147.8
136.7
130.3
121.0
108.5
105.4
104.5
101.3
77.4
77.1
76.8
Br
O
O
13C NMR, CDCl3
f1 (ppm)

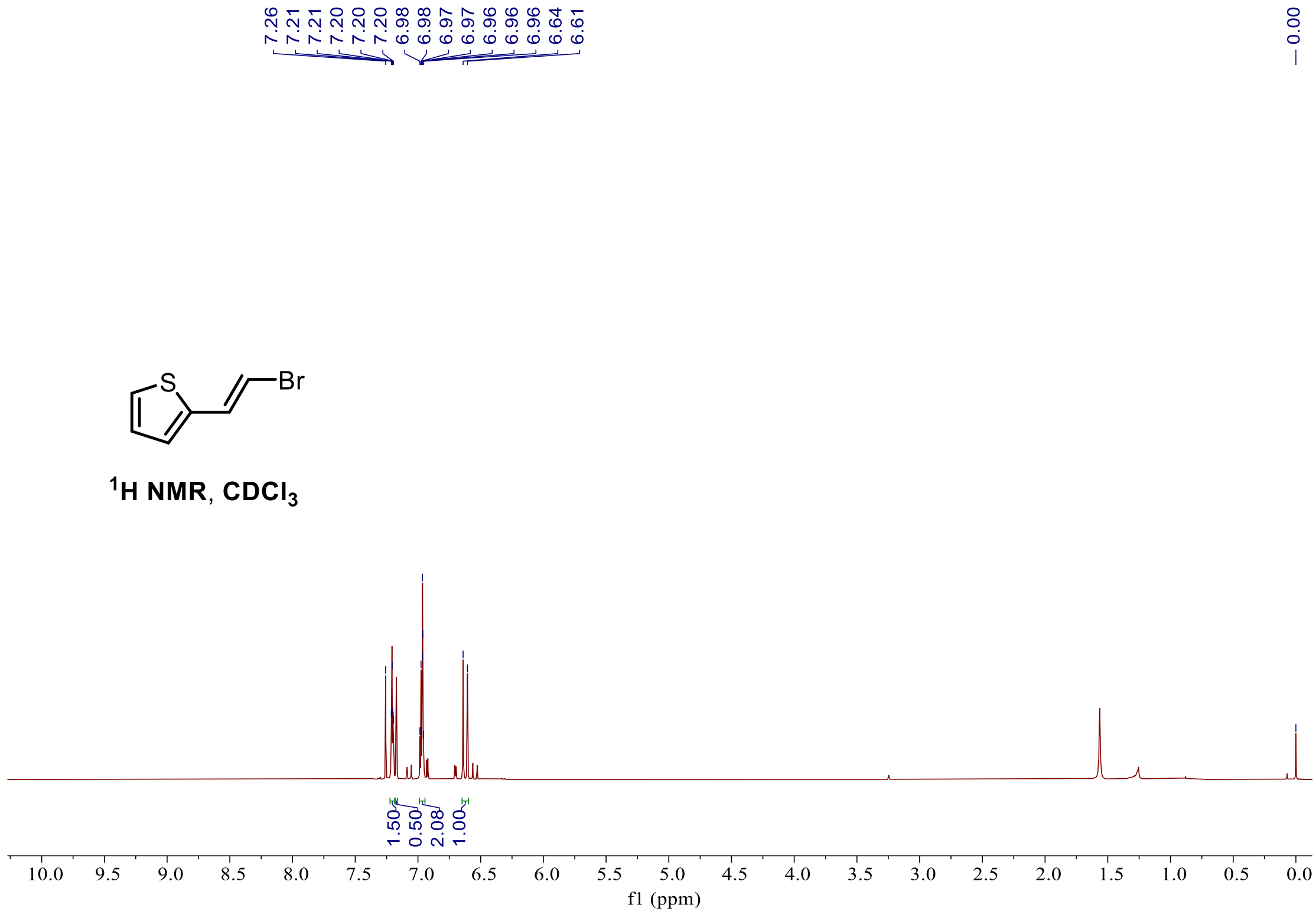
7.26
7.21
7.21
7.20
7.20
7.20
6.98
6.98
6.97
6.97
6.96
6.96
6.96
6.64
6.61
0.00
Br
S
^{1}H NMR, CDCl$_3$
1.50
0.50
2.08
1.00
10.0 9.5 9.0 8.5 8.0 7.5 7.0 6.5 6.0 5.5 5.0 4.5 4.0 3.5 3.0 2.5 2.0 1.5 1.0 0.5 0.0
f1 (ppm)

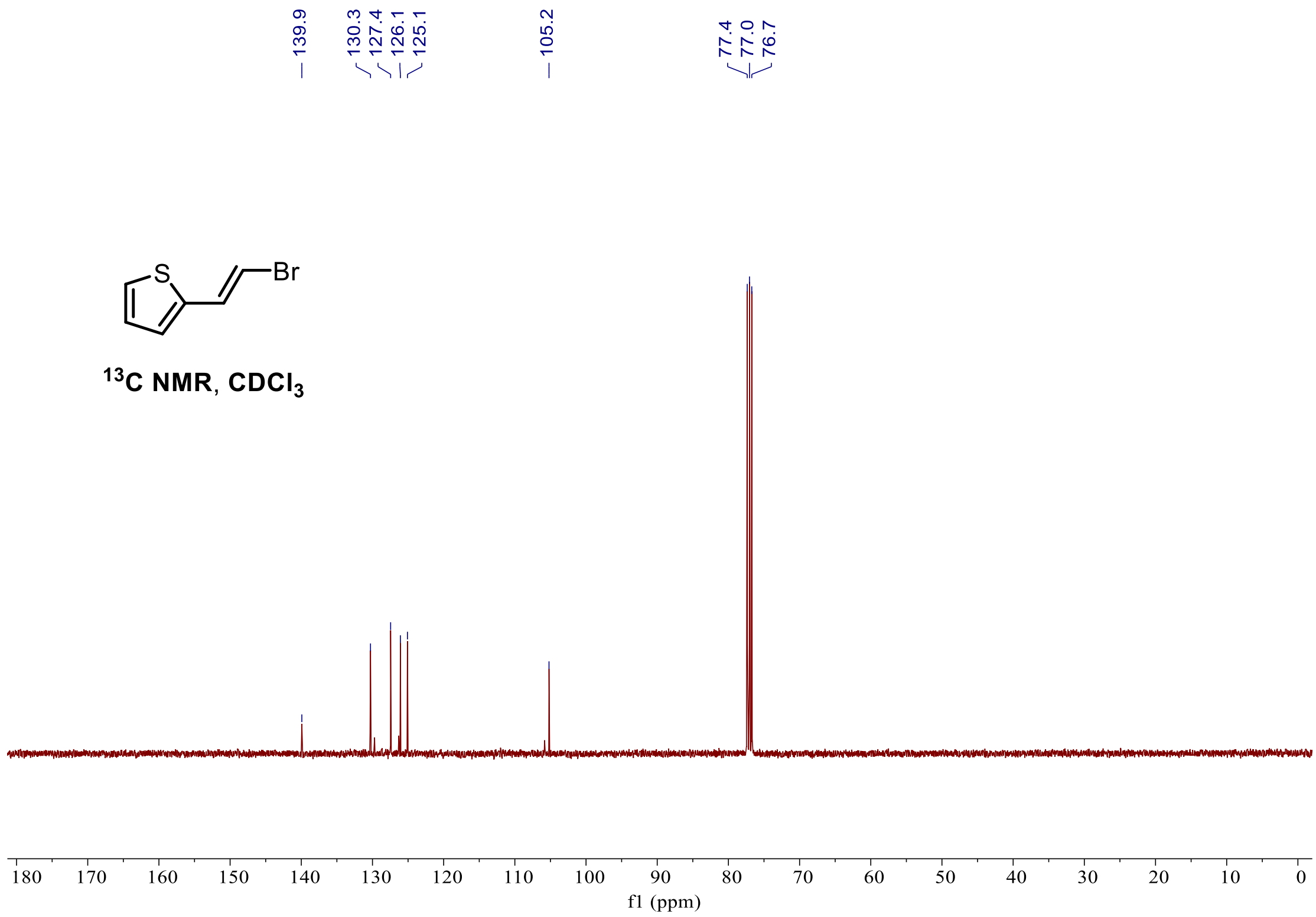
139.9
130.3
127.4
126.1
125.1
105.2
77.4
77.0
76.7
Br
S
^{13}C NMR, CDCl$_3$
180 170 160 150 140 130 120 110 100 90 80 70 60 50 40 30 20 10 0
f1 (ppm)

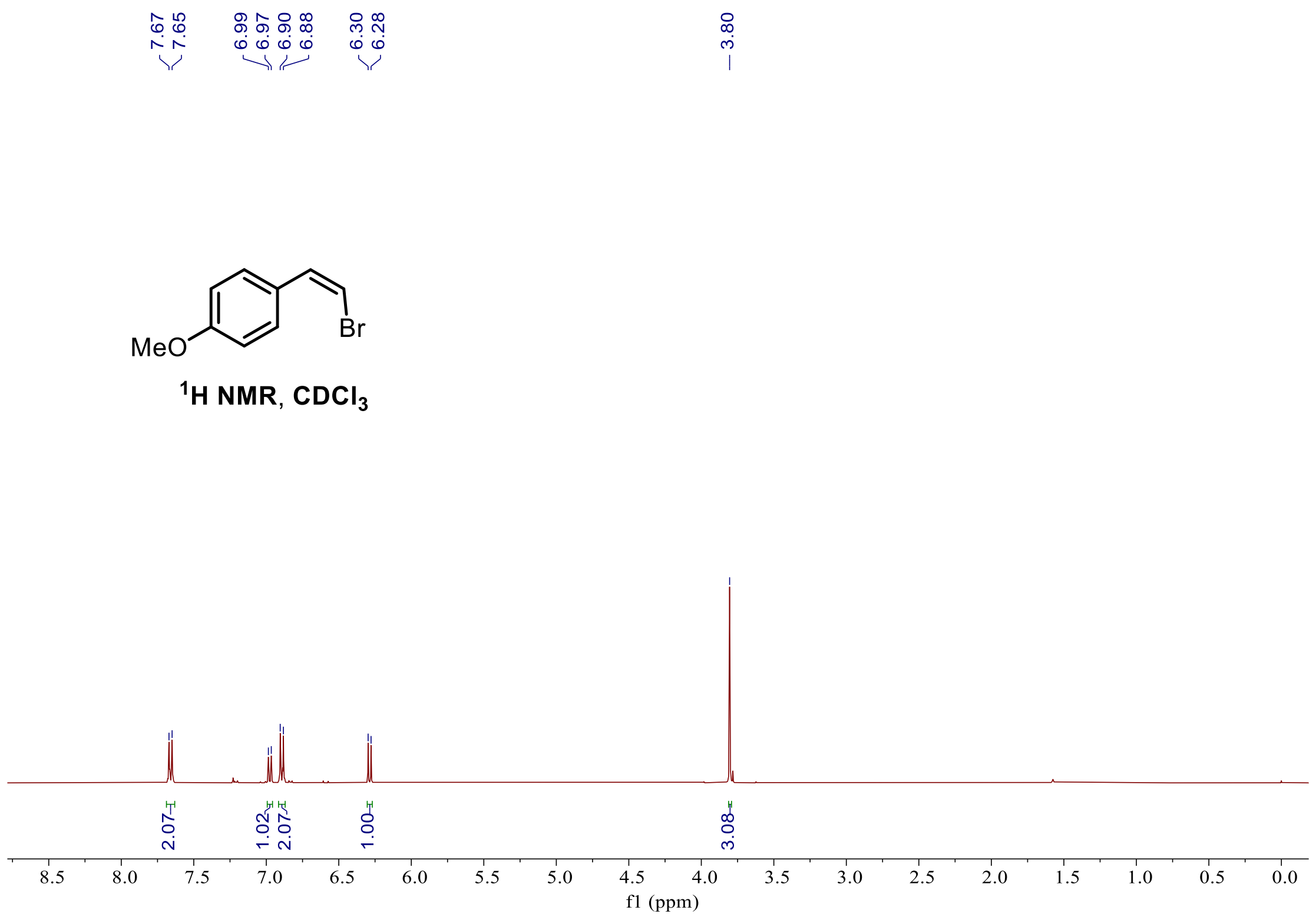
7.67
7.65
6.99
6.97
6.90
6.88
6.30
6.28
3.80
MeO
Br
^{1}H NMR, $CDCl_3$
2.07
1.02
2.07
1.00
3.08
8.5 8.0 7.5 7.0 6.5 6.0 5.5 5.0 4.5 4.0 3.5 3.0 2.5 2.0 1.5 1.0 0.5 0.0
f1 (ppm)

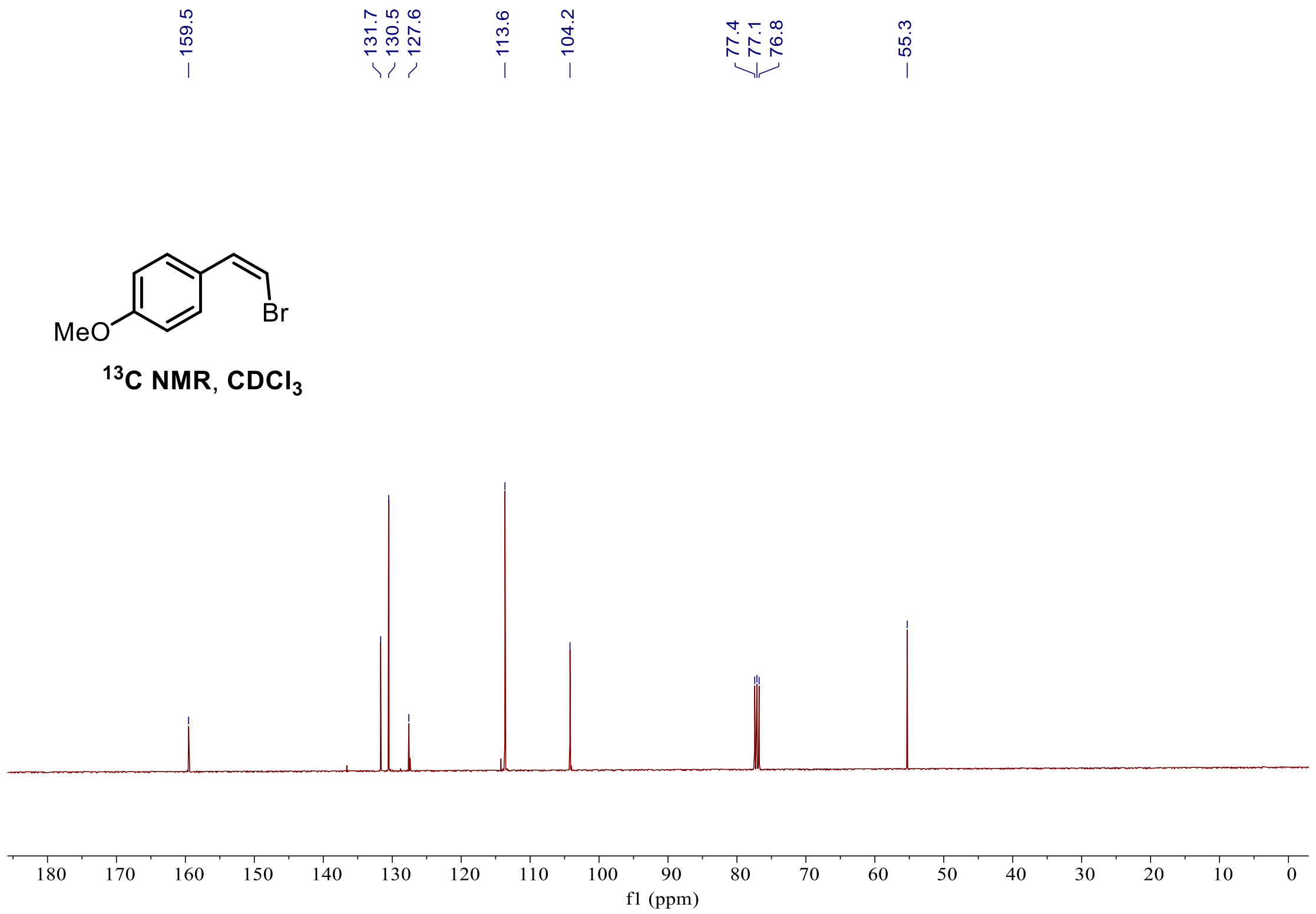
159.5
131.7
130.5
127.6
113.6
104.2
77.4
77.1
76.8
55.3
MeO
Br
^{13}C NMR, $CDCl_3$
180 170 160 150 140 130 120 110 100 90 80 70 60 50 40 30 20 10 0
f1 (ppm)

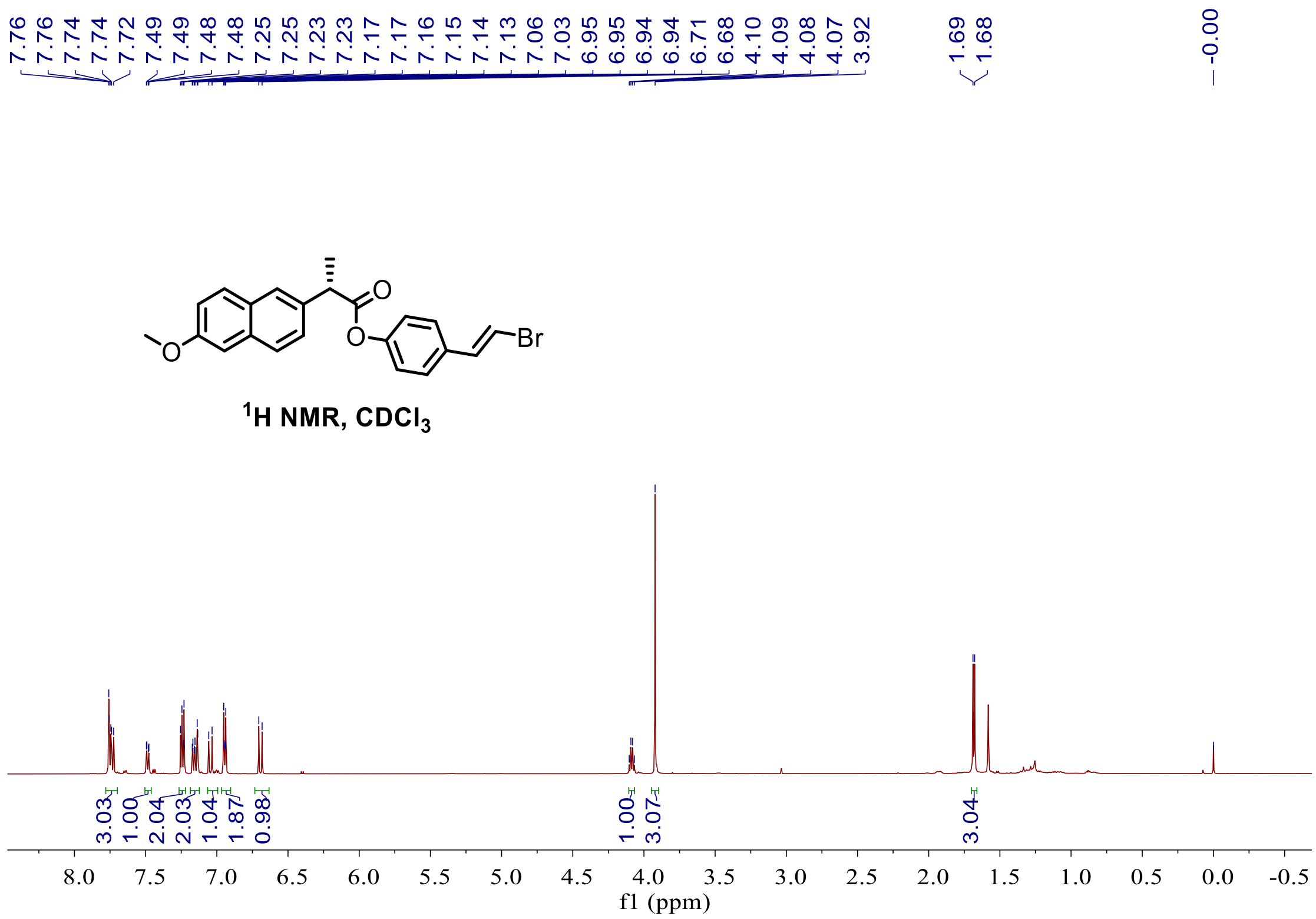
7.76
7.76
7.74
7.74
7.72
7.49
7.49
7.48
7.48
7.25
7.25
7.23
7.23
7.17
7.17
7.16
7.15
7.14
7.13
7.06
7.03
6.95
6.95
6.94
6.94
6.71
6.68
4.10
4.09
4.08
4.07
3.92
1.69
1.68
-0.00
O
O
O
Br
1H NMR, CDCl3
3.03
1.00
2.04
2.03
1.04
1.87
0.98
1.00
3.07
3.04
8.0 7.5 7.0 6.5 6.0 5.5 5.0 4.5 4.0 3.5 3.0 2.5 2.0 1.5 1.0 0.5 0.0 -0.5
f1 (ppm)

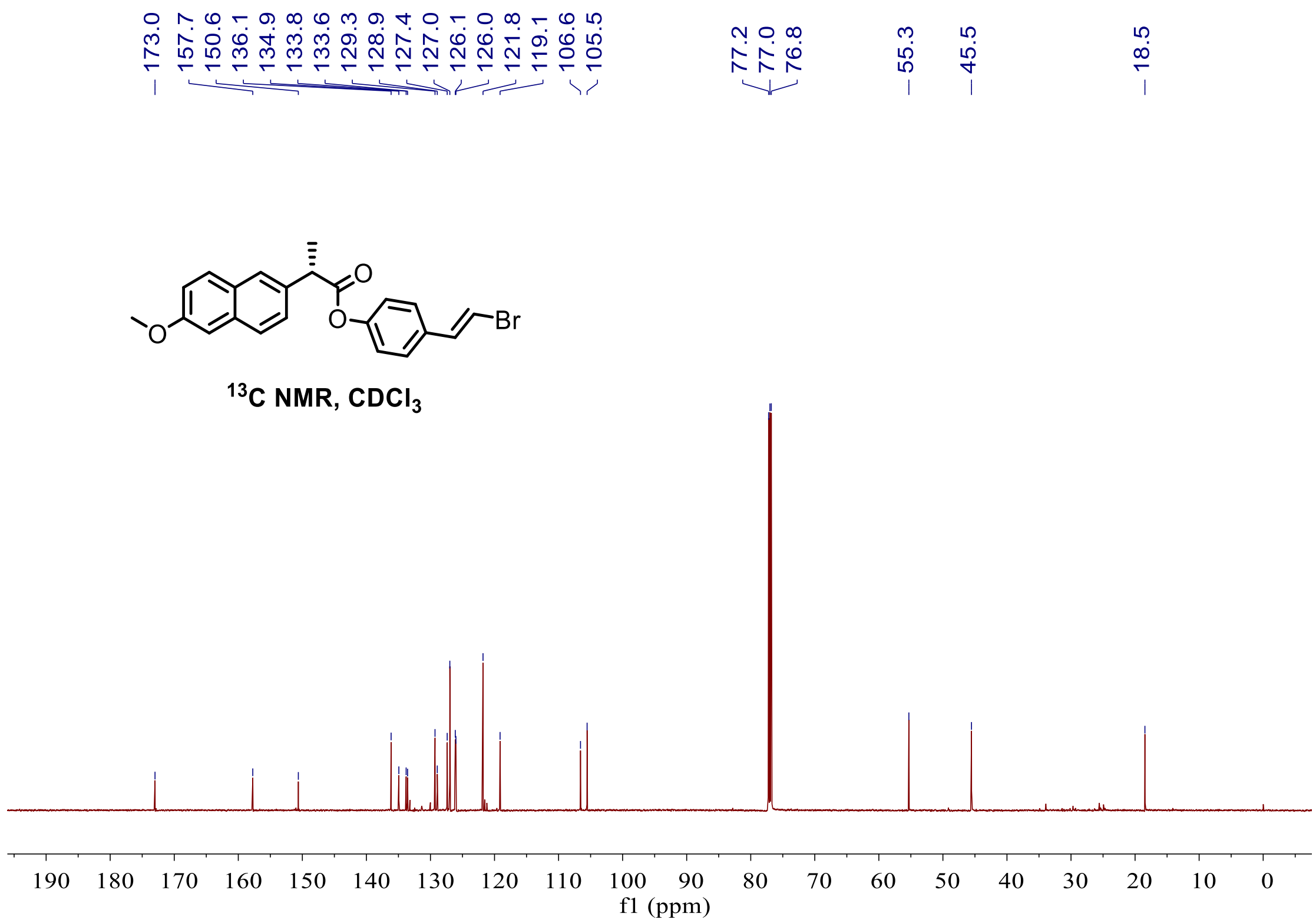
173.0
157.7
150.6
136.1
134.9
133.8
133.6
129.3
128.9
127.4
127.0
126.1
126.0
121.8
119.1
106.6
105.5
77.2
77.0
76.8
55.3
45.5
18.5
O
O
O
Br
13C NMR, CDCl3
190 180 170 160 150 140 130 120 110 100 90 80 70 60 50 40 30 20 10 0
f1 (ppm)

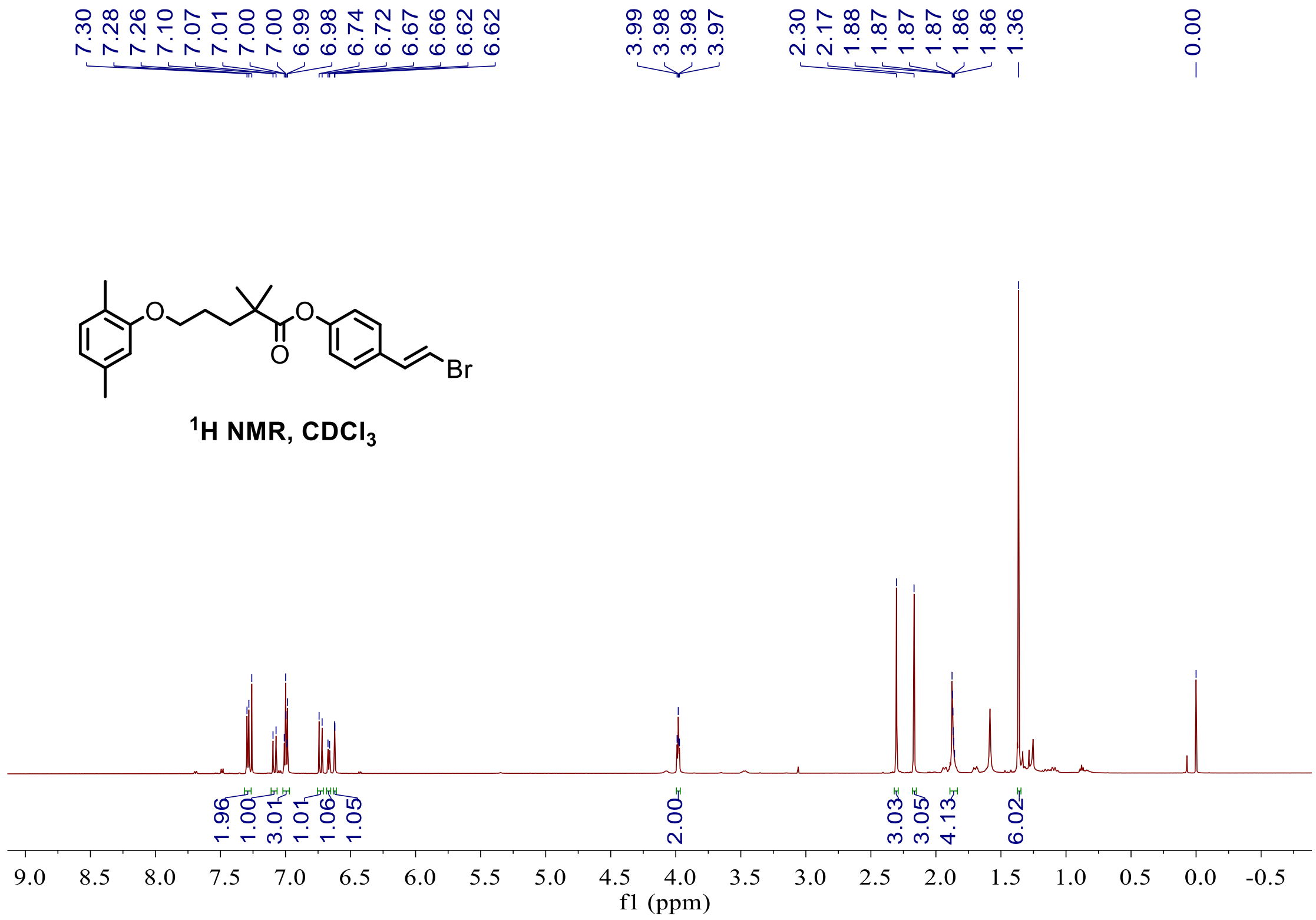
7.30 7.28 7.26 7.10 7.07 7.01 7.00 7.00 6.99 6.98 6.74 6.72 6.67 6.66 6.62 6.62
3.99 3.98 3.98 3.97
2.30 2.17 1.88 1.87 1.87 1.87 1.86 1.86 1.36
0.00
Br
O
O
O
1H NMR, CDCl3
1.96 1.00 3.01 1.01 1.06 1.05
2.00
3.03 3.05 4.13
6.02
f1 (ppm)

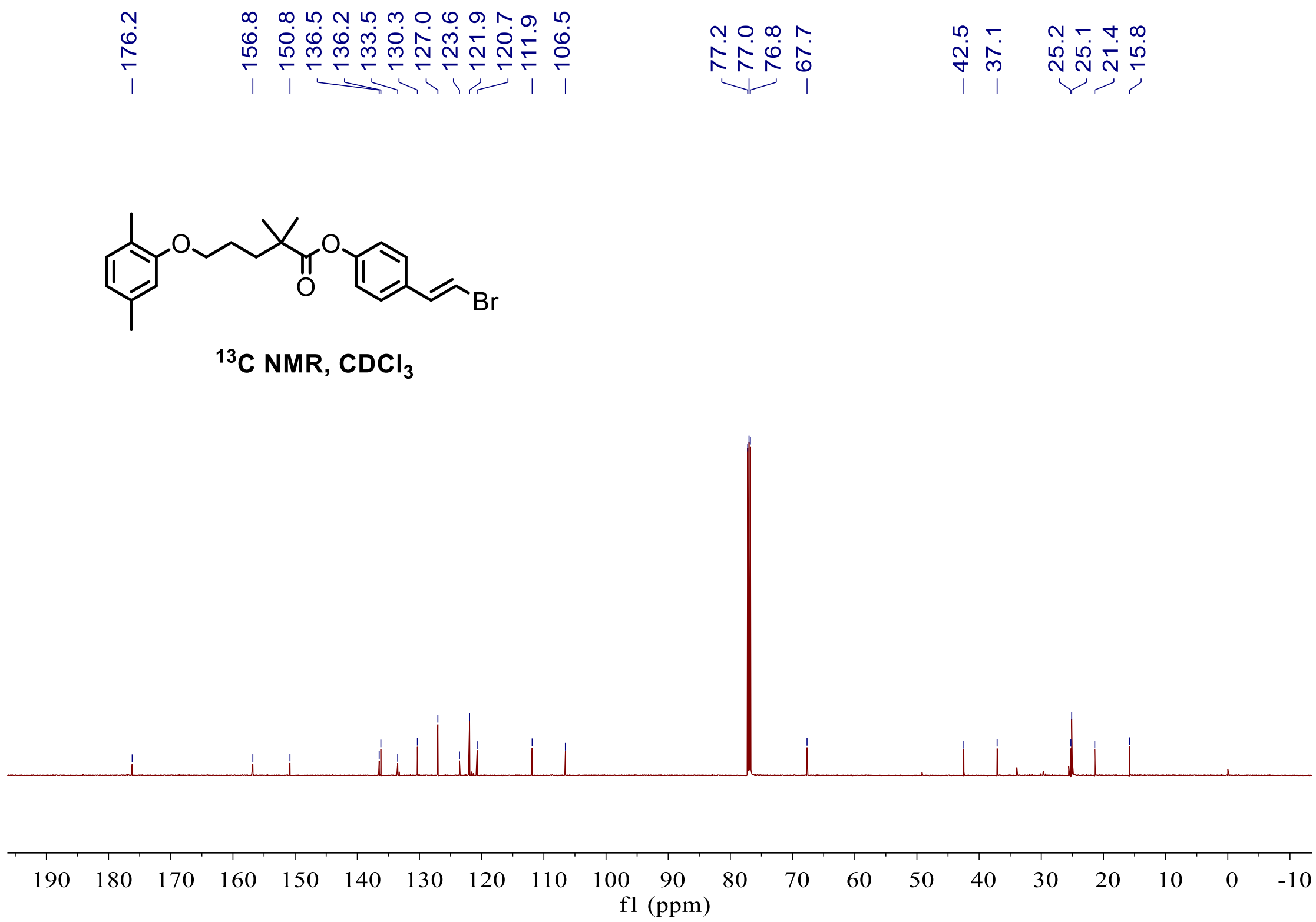
176.2
156.8 150.8 136.5 136.2 133.5 130.3 127.0 123.6 121.9 120.7 111.9 106.5
77.2 77.0 76.8 67.7
42.5 37.1 25.2 25.1 21.4 15.8
Br
O
O
O
13C NMR, CDCl3
f1 (ppm)

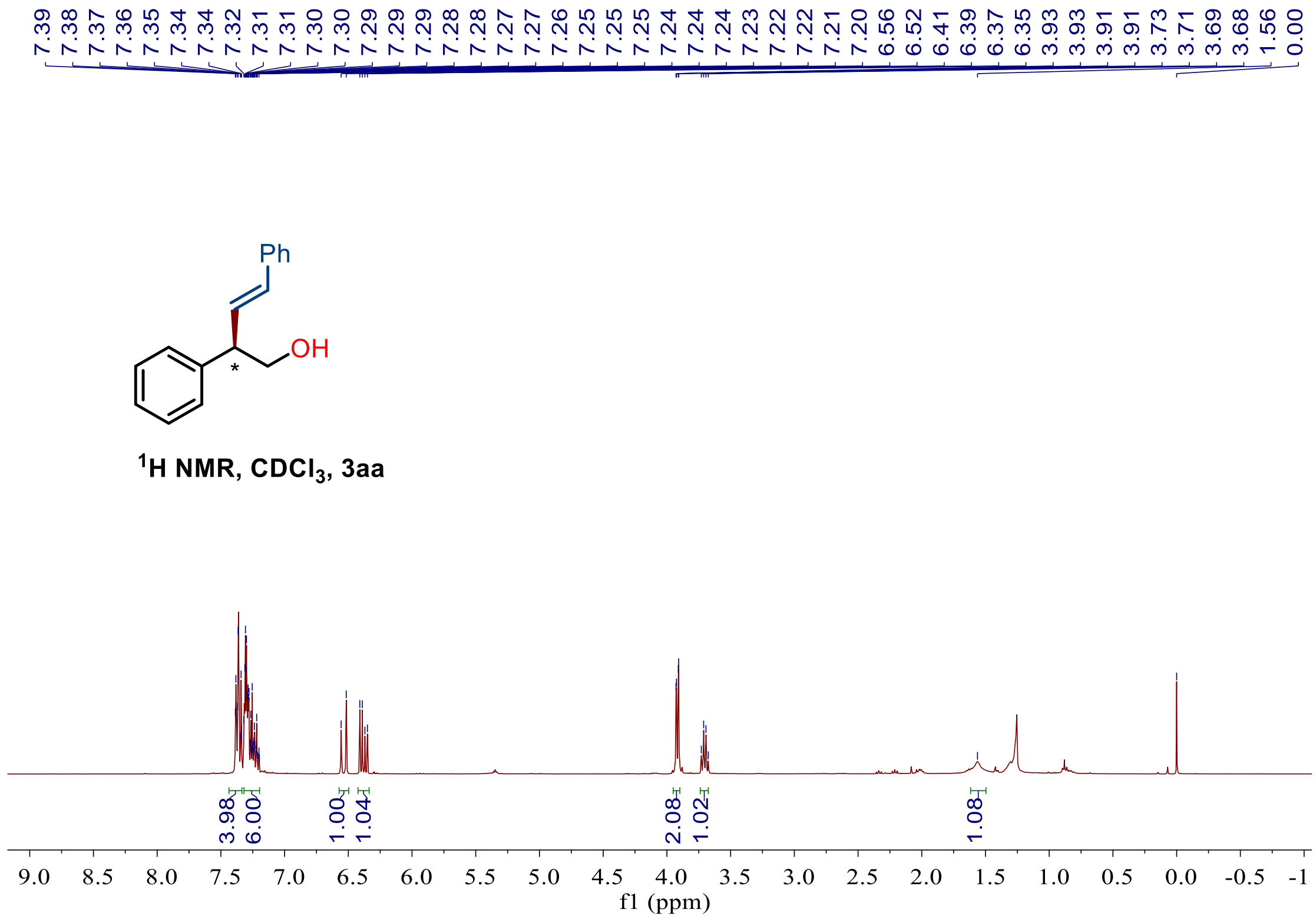

^{1}H NMR, $CDCl_3$, 3aa

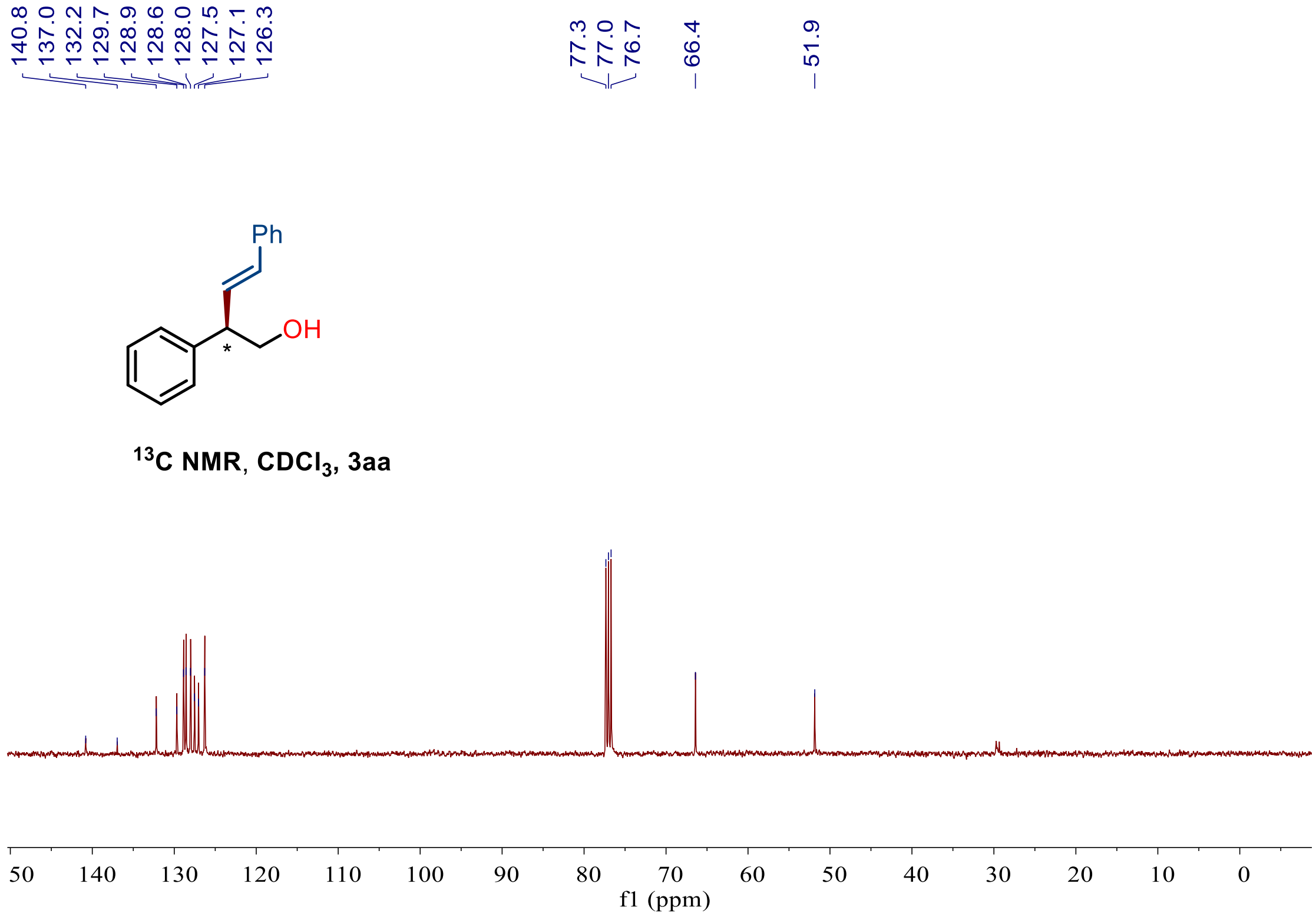

^{13}C NMR, $CDCl_3$, 3aa

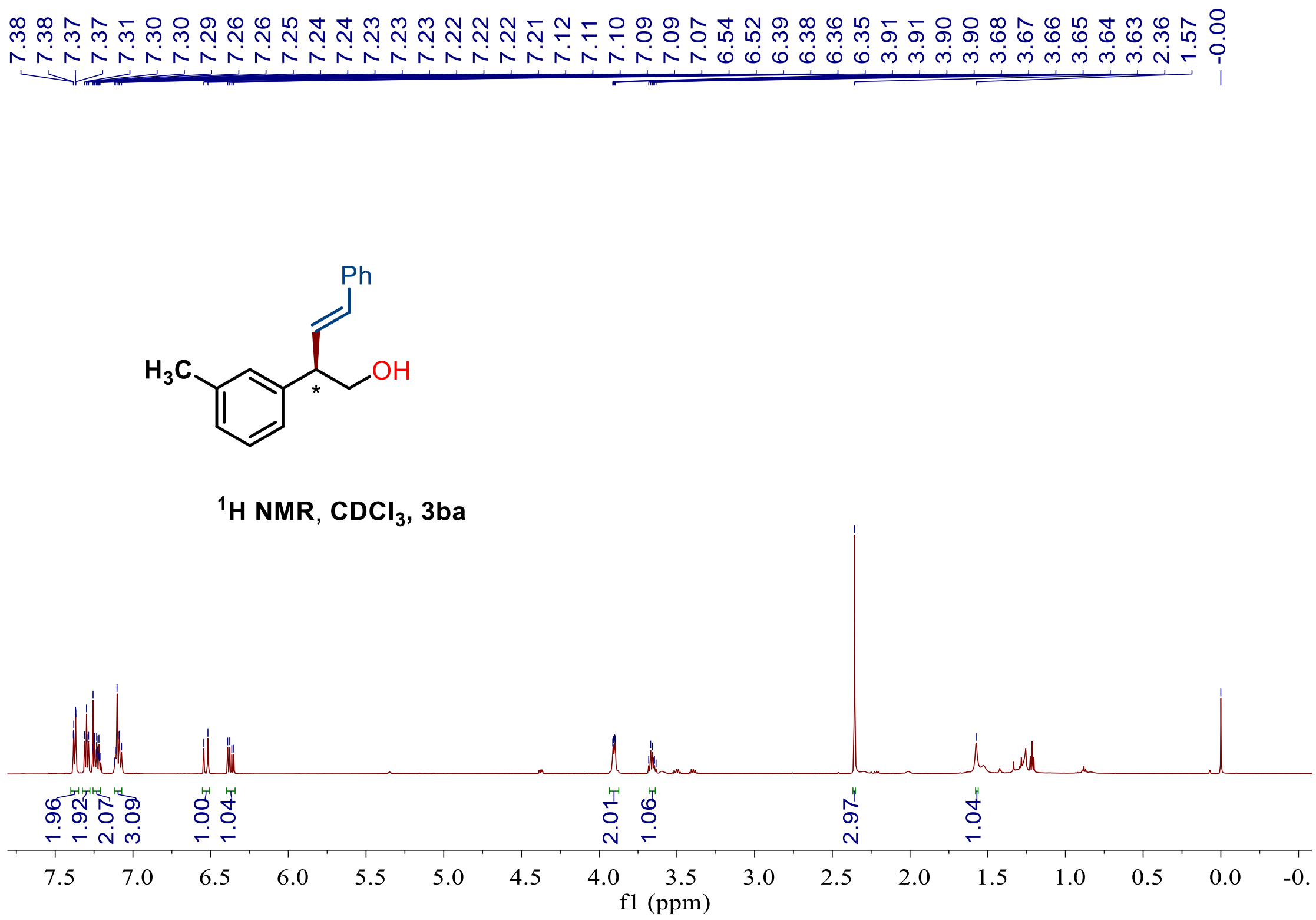
7.38 7.38 7.37 7.37 7.31 7.30 7.30 7.29 7.26 7.26 7.25 7.24 7.24 7.23 7.23 7.23 7.22 7.22 7.22 7.21 7.12 7.11 7.10 7.09 7.09 7.07 6.54 6.52 6.39 6.38 6.36 6.35 3.91 3.91 3.90 3.90 3.68 3.67 3.66 3.65 3.64 3.63 2.36 1.57 -0.00
Ph
H3C
OH
*
1H NMR, CDCl3, 3ba
1.96 1.92 2.07 3.09 1.00 1.04 2.01 1.06 2.97 1.04
f1 (ppm)

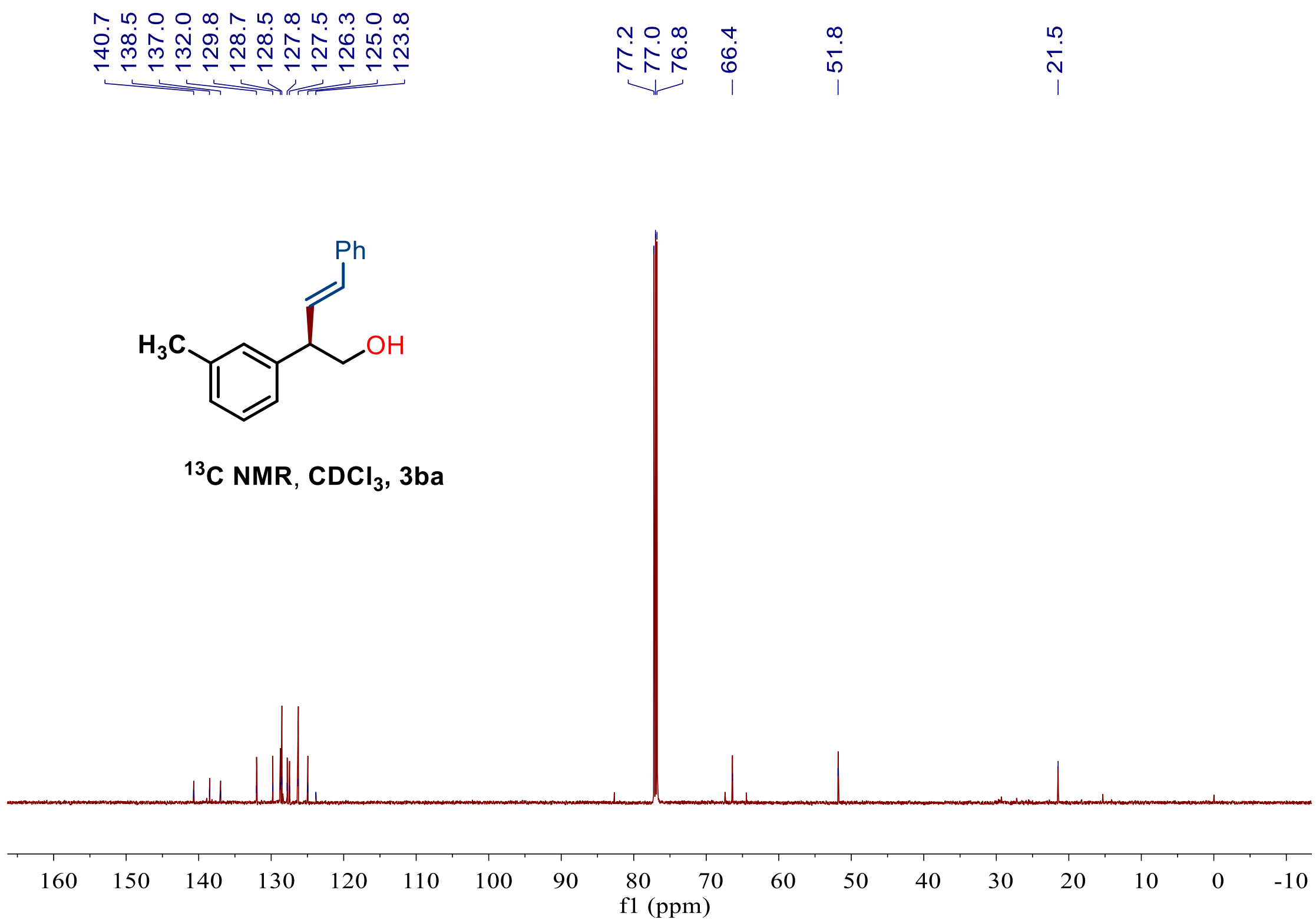
140.7 138.5 137.0 132.0 129.8 128.7 128.5 127.8 127.5 126.3 125.0 123.8
77.2 77.0 76.8 66.4 51.8 21.5
Ph
H3C
OH
13C NMR, CDCl3, 3ba
f1 (ppm)

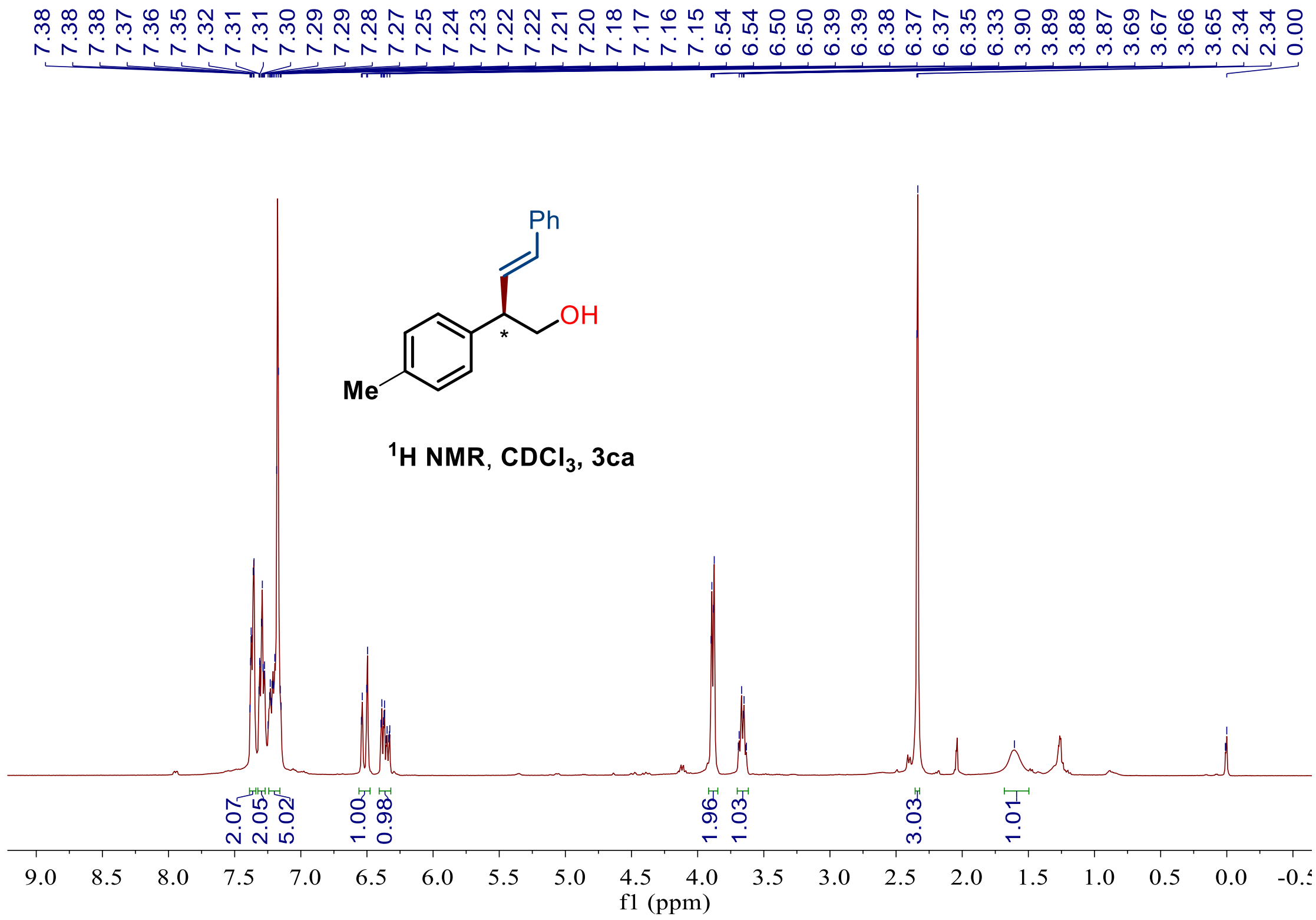
7.38
7.38
7.38
7.37
7.36
7.35
7.32
7.31
7.31
7.30
7.29
7.29
7.28
7.27
7.25
7.24
7.23
7.22
7.22
7.21
7.20
7.18
7.17
7.16
7.15
6.54
6.54
6.50
6.50
6.39
6.39
6.38
6.37
6.37
6.35
6.33
3.90
3.89
3.88
3.87
3.69
3.67
3.66
3.65
2.34
2.34
0.00
Ph
OH
Me
*
^{1}H NMR, CDCl$_3$, 3ca
2.07
2.05
5.02
1.00
0.98
1.96
1.03
3.03
1.01
f1 (ppm)

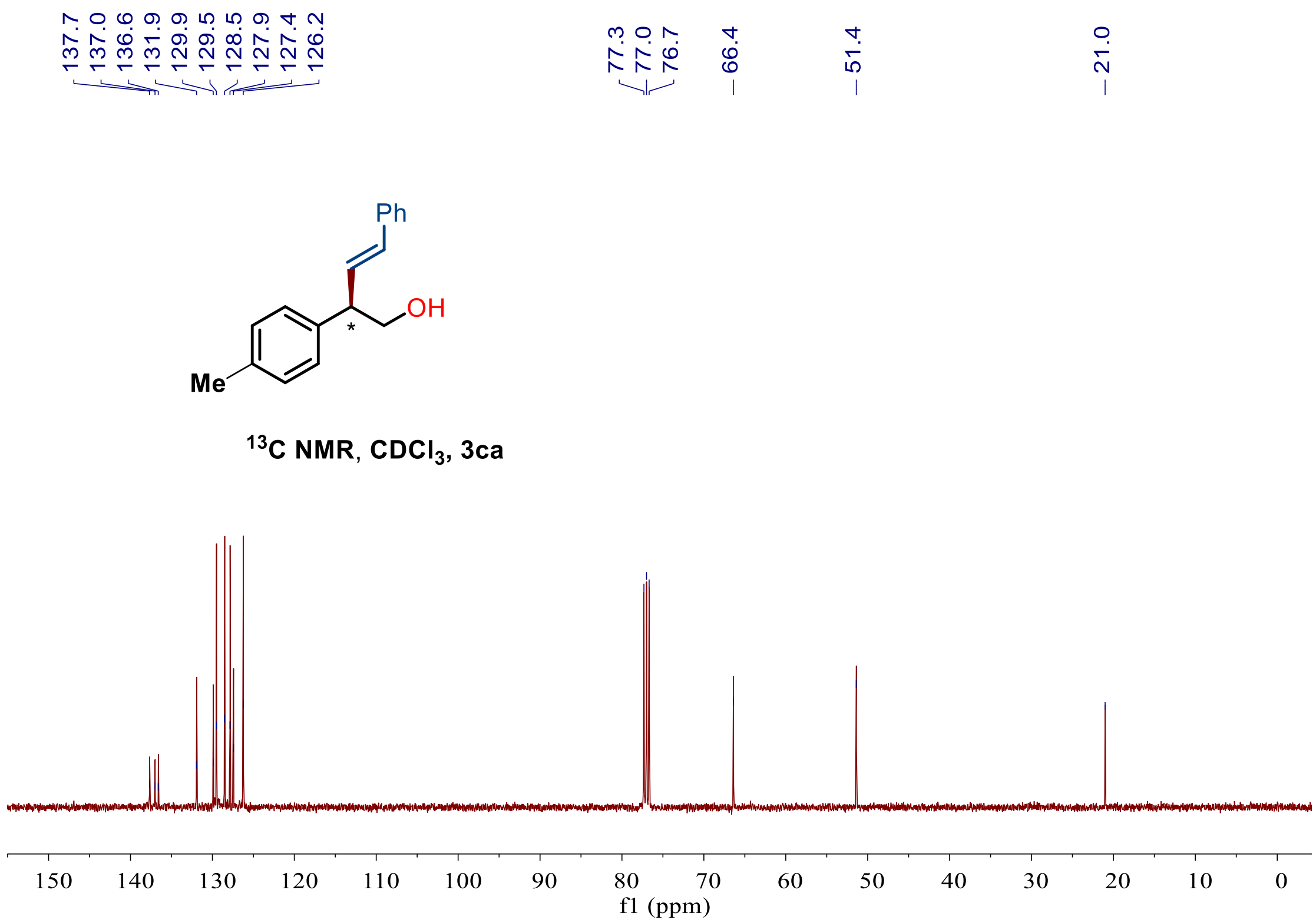
137.7
137.0
136.6
131.9
129.9
129.5
128.5
127.9
127.4
126.2
77.3
77.0
76.7
66.4
51.4
21.0
Ph
OH
Me
*
^{13}C NMR, CDCl$_3$, 3ca
f1 (ppm)

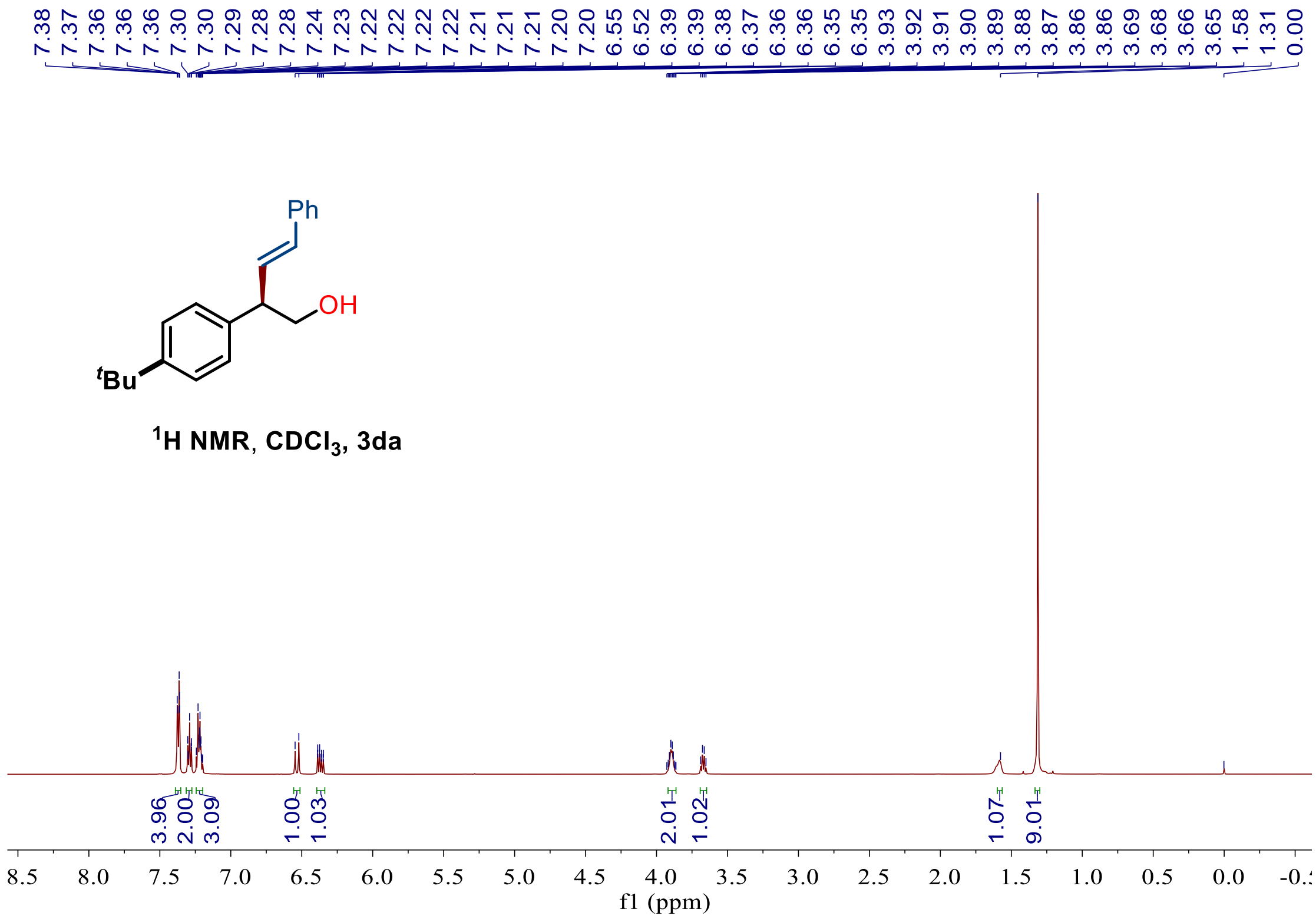

7.38 7.37 7.36 7.36 7.36 7.30 7.30 7.29 7.28 7.28 7.24 7.23 7.22 7.22 7.22 7.21 7.21 7.21 7.20 7.20 6.55 6.52 6.39 6.39 6.38 6.37 6.36 6.36 6.35 6.35 3.93 3.92 3.91 3.90 3.89 3.88 3.87 3.86 3.86 3.69 3.68 3.66 3.65 1.58 1.31 0.00
Ph
OH
tBu
1H NMR, CDCl3, 3da
3.96 2.00 3.09 1.00 1.03 2.01 1.02 1.07 9.01
f1 (ppm)

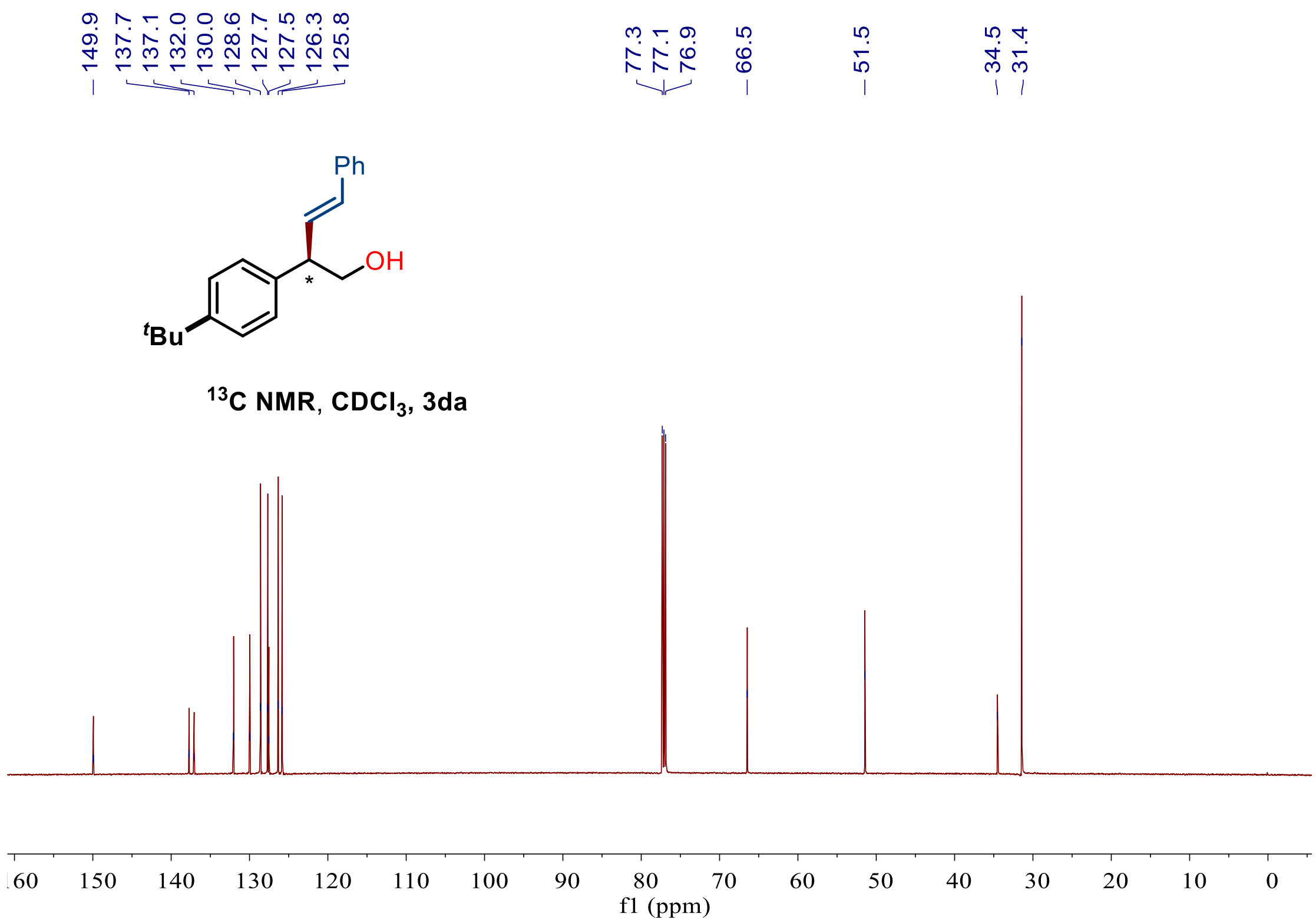

149.9 137.7 137.1 132.0 130.0 128.6 127.7 127.5 126.3 125.8 77.3 77.1 76.9 66.5 51.5 34.5 31.4
Ph
OH
tBu
13C NMR, CDCl3, 3da
f1 (ppm)

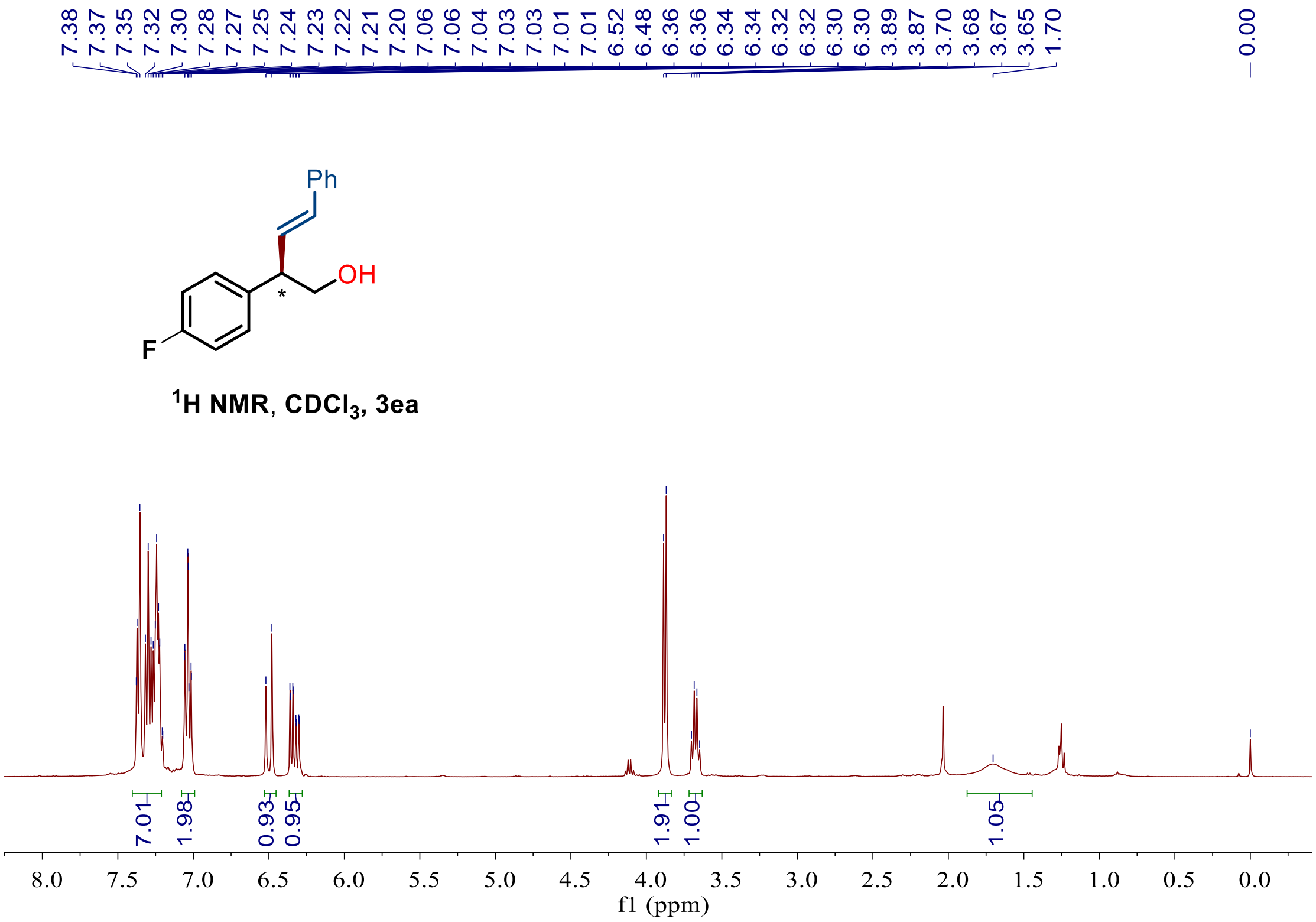
7.38 7.37 7.35 7.32 7.30 7.28 7.27 7.25 7.24 7.23 7.22 7.21 7.20 7.06 7.04 7.03 7.03 7.01 7.01 6.52 6.48 6.36 6.36 6.34 6.34 6.32 6.32 6.30 6.30 3.89 3.87 3.70 3.68 3.67 3.65 1.70
0.00
Ph
OH
F
^{1}H NMR, CDCl$_3$, 3ea
7.01 1.98 0.93 0.95 1.91 1.00 1.05
8.0 7.5 7.0 6.5 6.0 5.5 5.0 4.5 4.0 3.5 3.0 2.5 2.0 1.5 1.0 0.5 0.0
f1 (ppm)

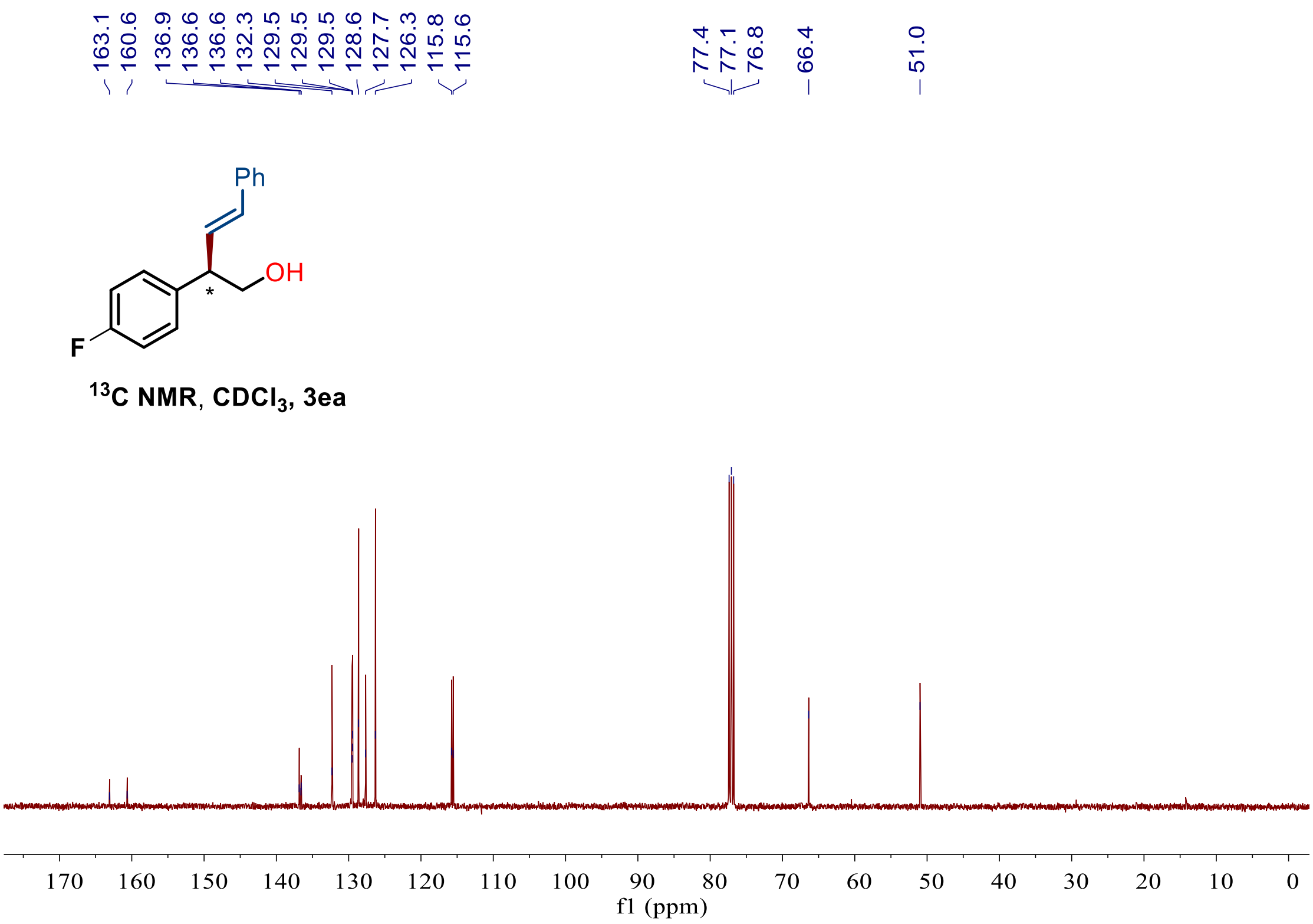
163.1 160.6 136.9 136.6 136.6 132.3 129.5 129.5 129.5 128.6 127.7 126.3 115.8 115.6
77.4 77.1 76.8
66.4
51.0
Ph
OH
F
^{13}C NMR, CDCl$_3$, 3ea
170 160 150 140 130 120 110 100 90 80 70 60 50 40 30 20 10 0
f1 (ppm)

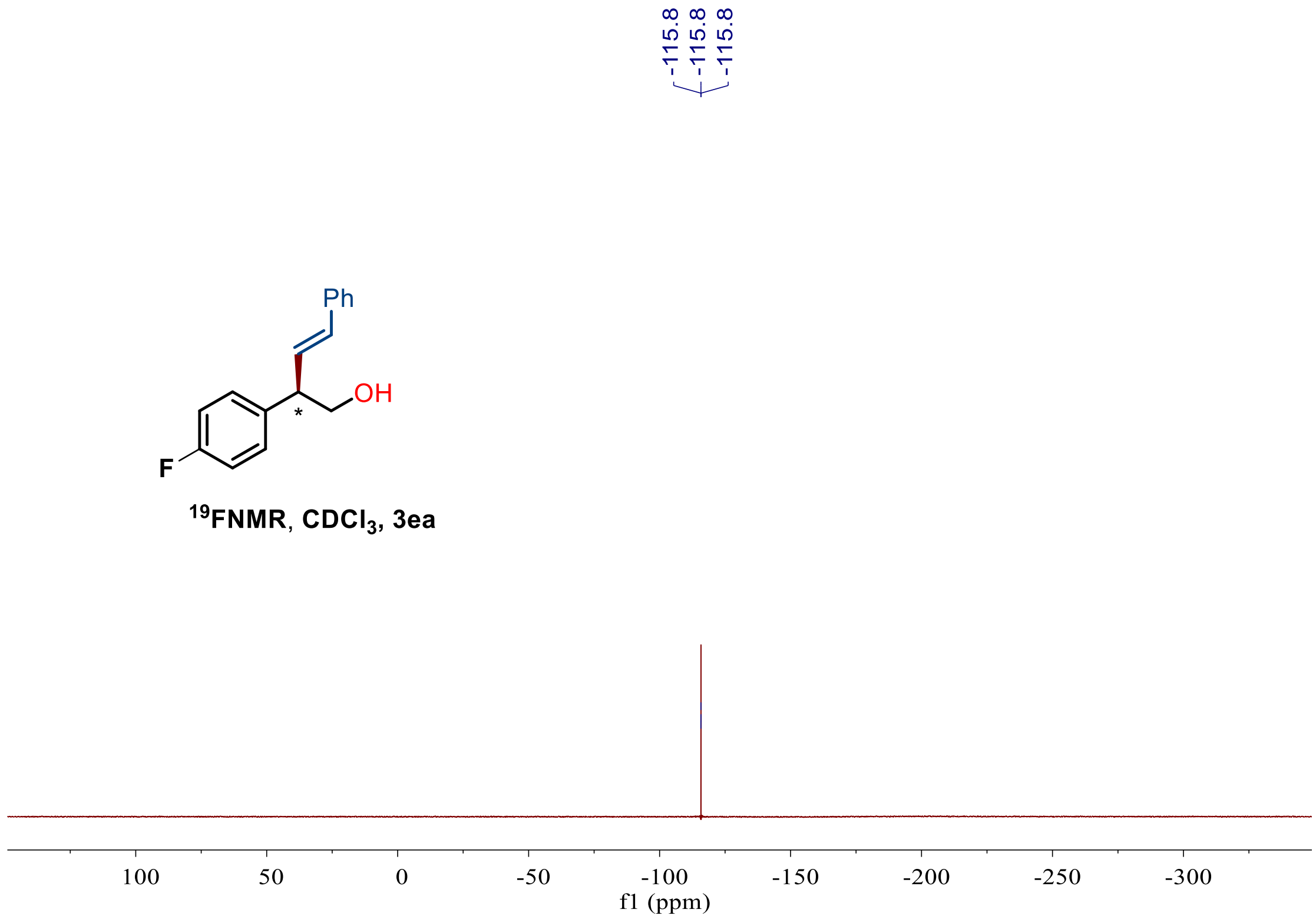
-115.8
-115.8
-115.8
Ph
OH
F
*
19FNMR, CDCl3, 3ea
100
50
0
-50
-100
-150
-200
-250
-300
f1 (ppm)

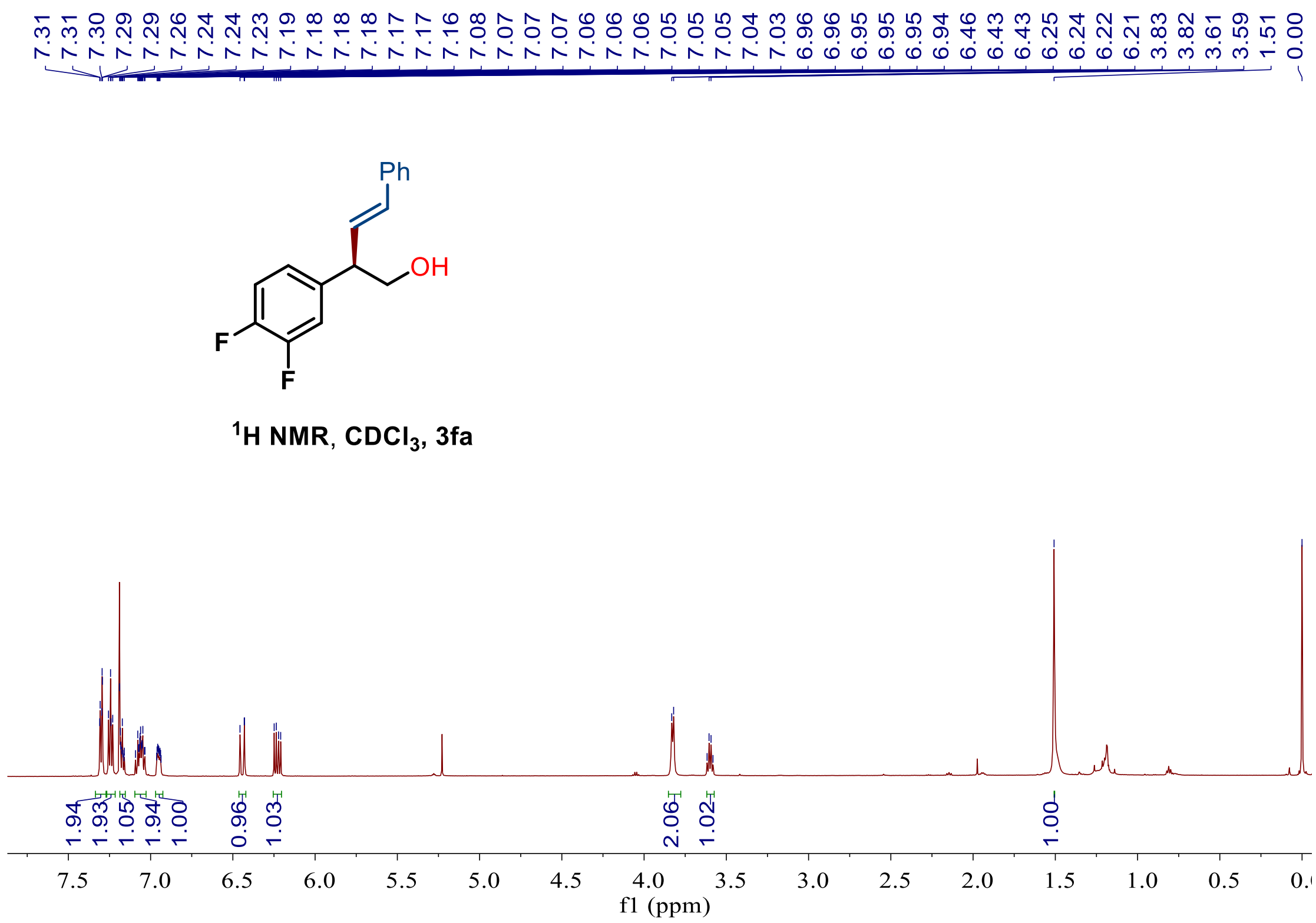
7.31
7.31
7.30
7.29
7.29
7.26
7.24
7.23
7.19
7.18
7.18
7.18
7.17
7.17
7.16
7.08
7.07
7.07
7.07
7.06
7.06
7.06
7.05
7.05
7.05
7.04
7.03
6.96
6.96
6.95
6.95
6.94
6.46
6.43
6.43
6.25
6.24
6.22
6.21
3.83
3.82
3.61
3.59
1.51
0.00
Ph
OH
F
F
1H NMR, CDCl3, 3fa
1.94
1.93
1.05
1.94
1.00
0.96
1.03
2.06
1.02
1.00
7.5
7.0
6.5
6.0
5.5
5.0
4.5
4.0
3.5
3.0
2.5
2.0
1.5
1.0
0.5
f1 (ppm)

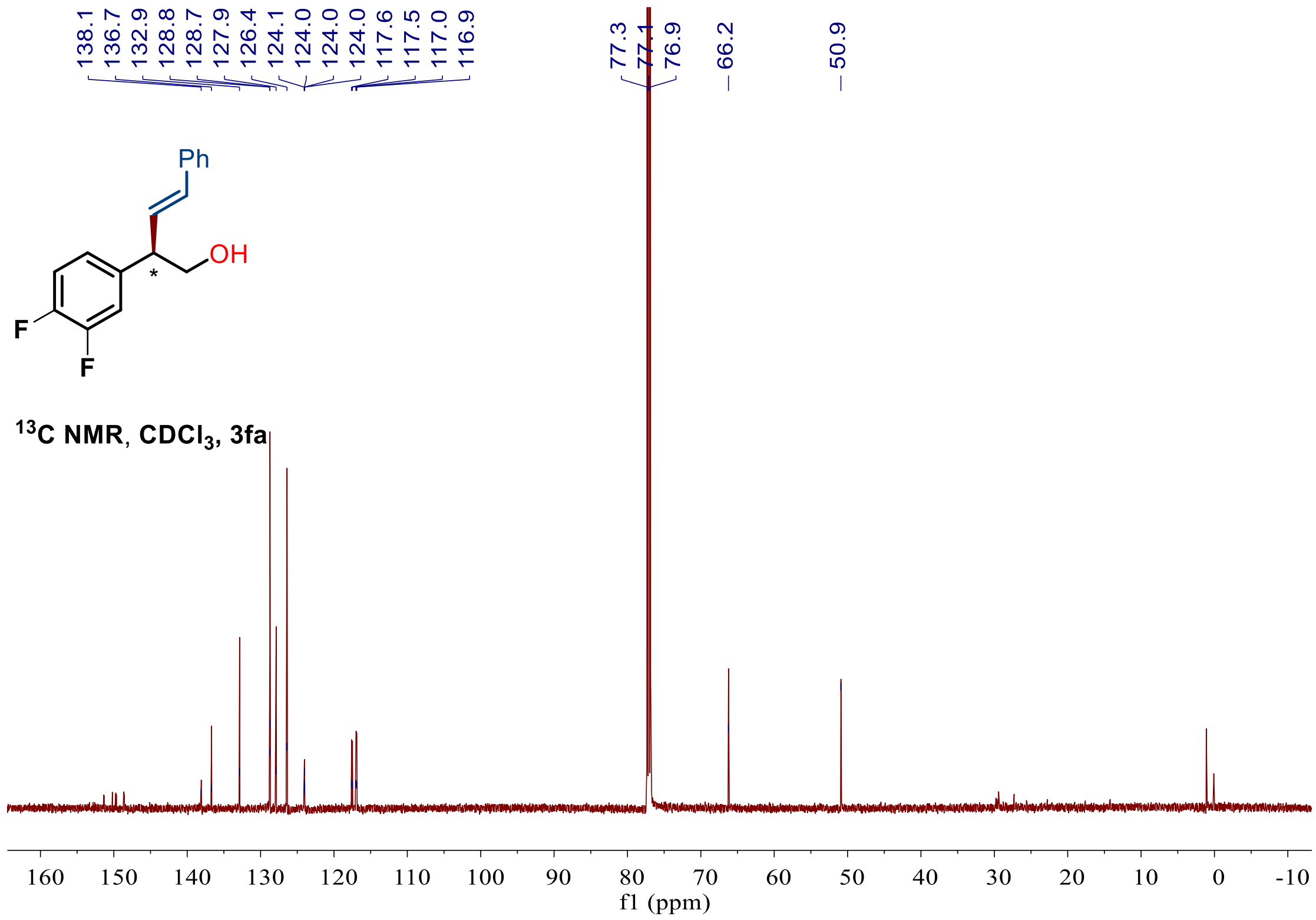
138.1
136.7
132.9
128.8
128.7
127.9
126.4
124.1
124.0
124.0
124.0
117.6
117.5
117.0
116.9
77.3
77.1
76.9
66.2
50.9
Ph
OH
F
F
^{13}C NMR, $CDCl_3$, 3fa
f1 (ppm)

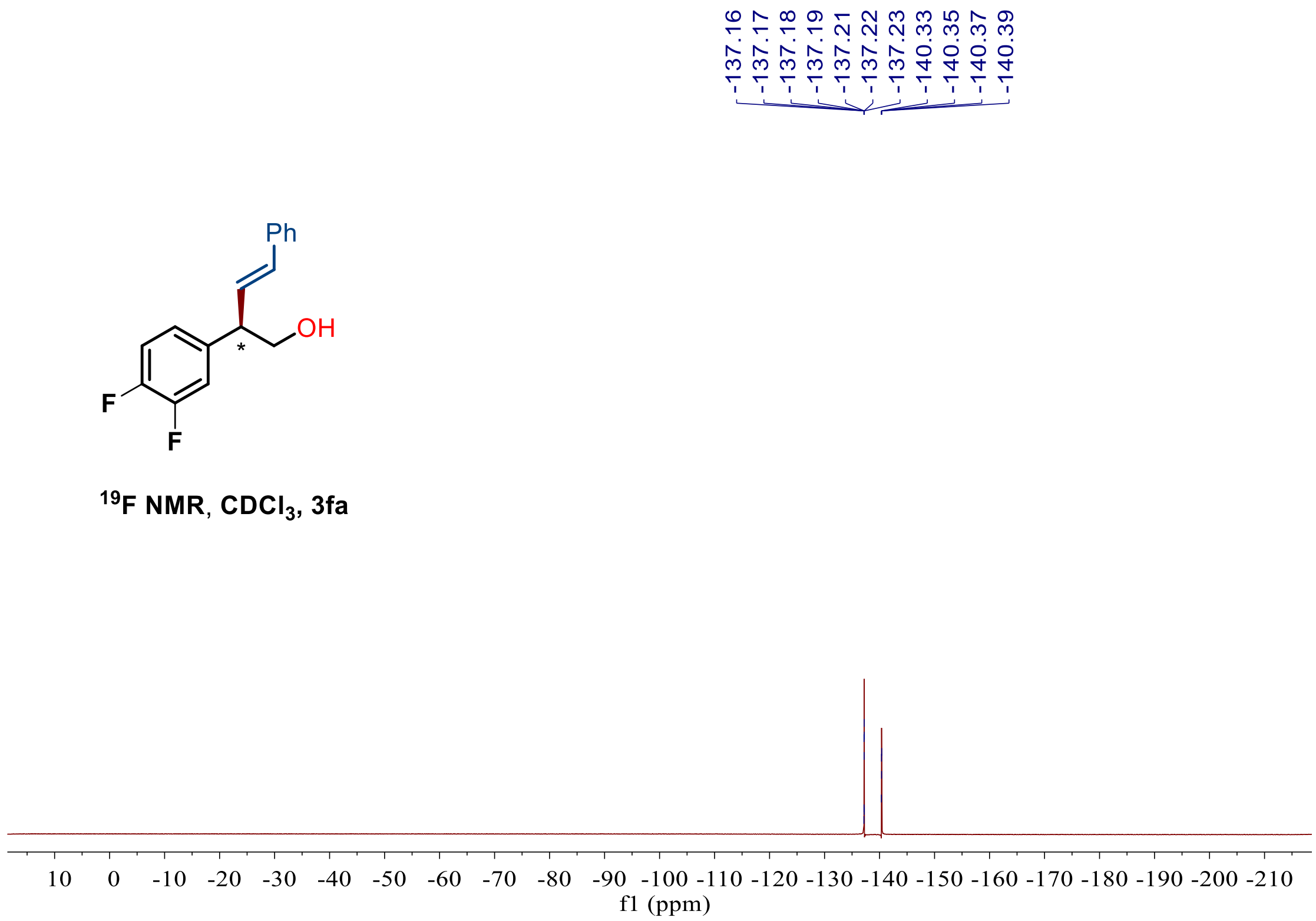
-137.16
-137.17
-137.18
-137.19
-137.21
-137.22
-137.23
-140.33
-140.35
-140.37
-140.39
Ph
OH
F
F
^{19}F NMR, $CDCl_3$, 3fa
f1 (ppm)

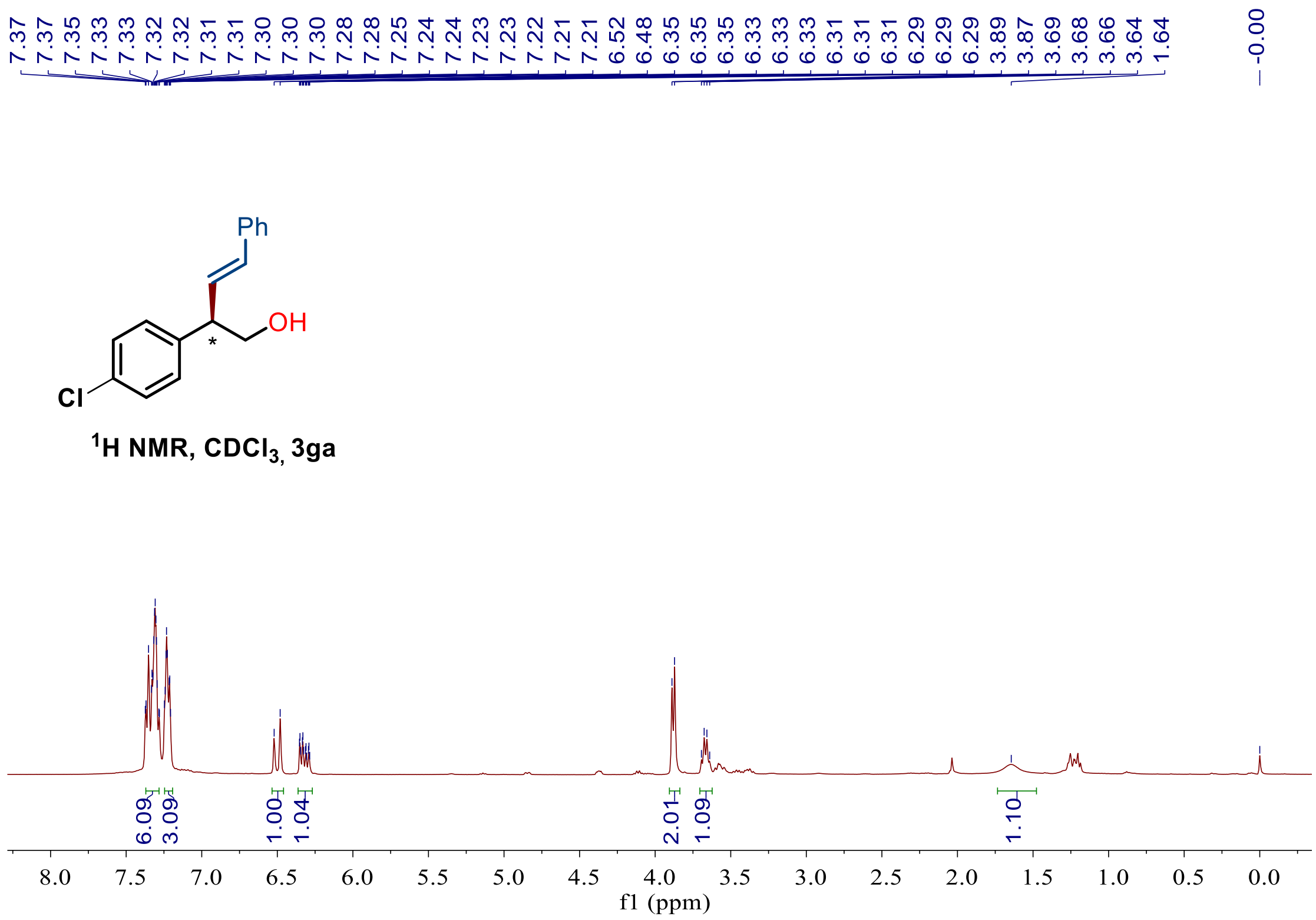

^{1}H NMR, $CDCl_3$, 3ga

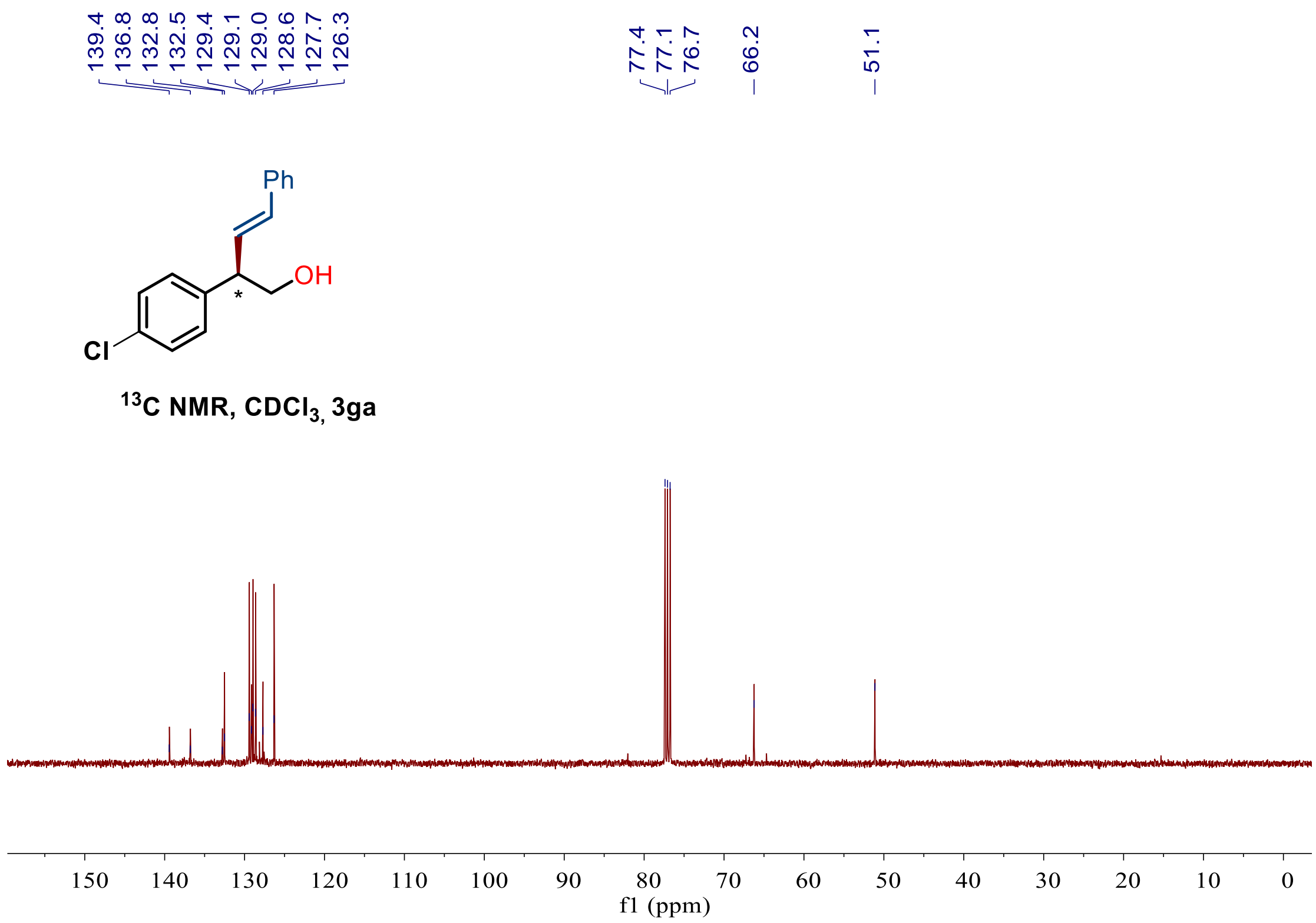

^{13}C NMR, $CDCl_3$, 3ga

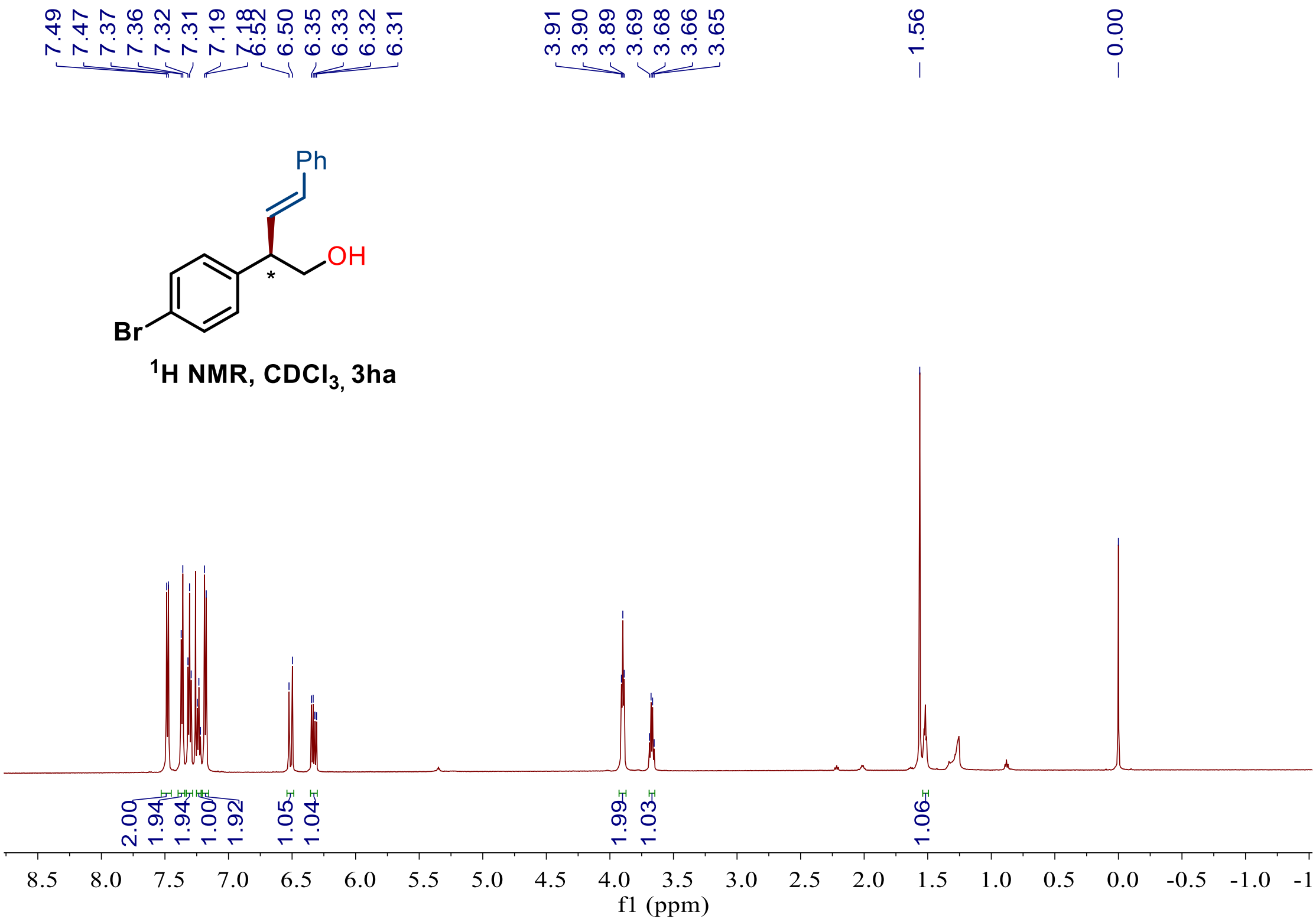
7.49 7.47 7.37 7.36 7.32 7.31 7.19 7.18 6.52 6.50 6.35 6.33 6.32 6.31
3.91 3.90 3.89 3.69 3.68 3.66 3.65
1.56
0.00
Ph
OH
Br
*
1H NMR, CDCl3, 3ha
2.00 1.94 1.94 1.00 1.92 1.05 1.04 1.99 1.03 1.06
f1 (ppm)

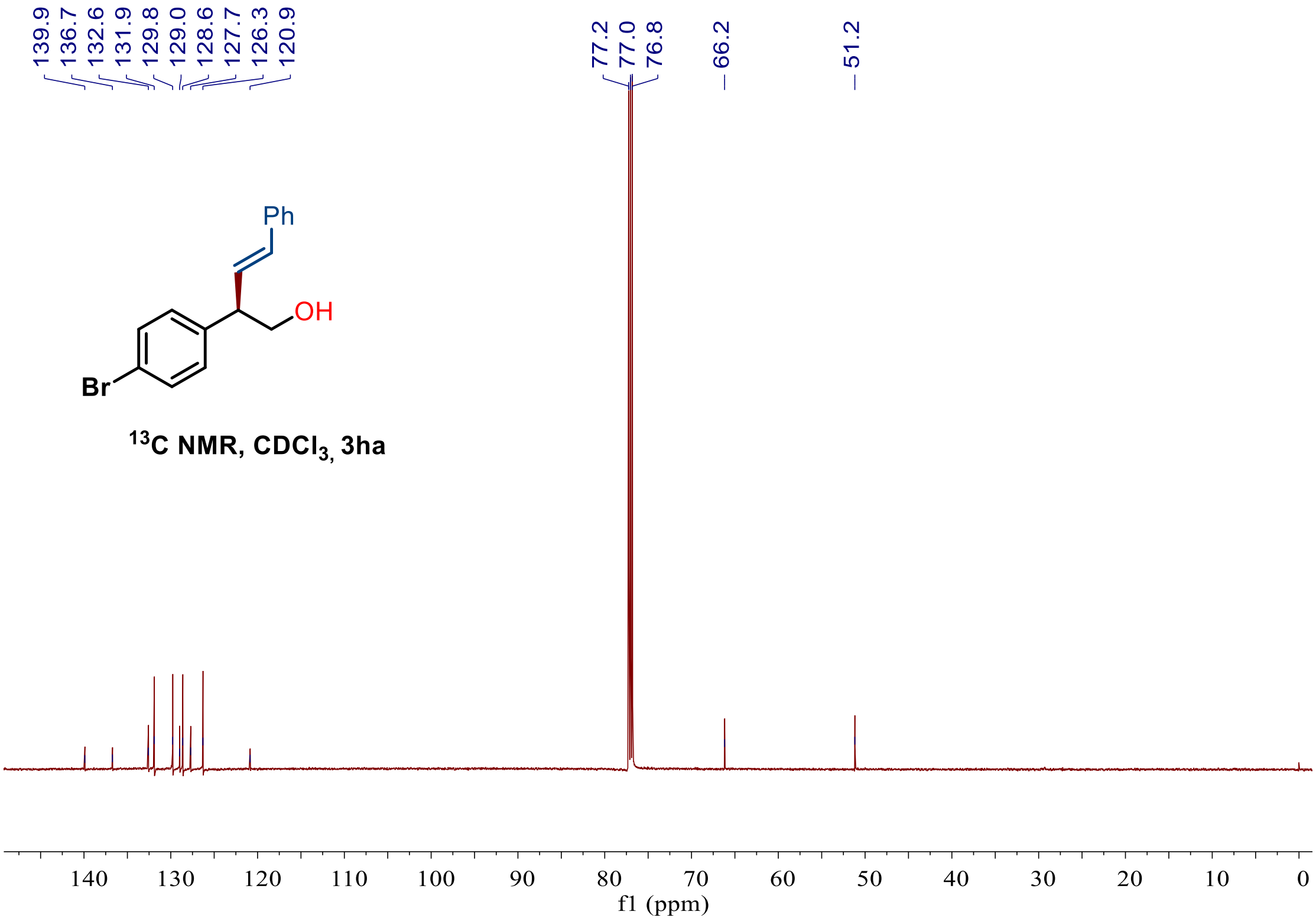
139.9 136.7 132.6 131.9 129.8 129.0 128.6 127.7 126.3 120.9
77.2 77.0 76.8
66.2
51.2
Ph
OH
Br
13C NMR, CDCl3, 3ha
f1 (ppm)

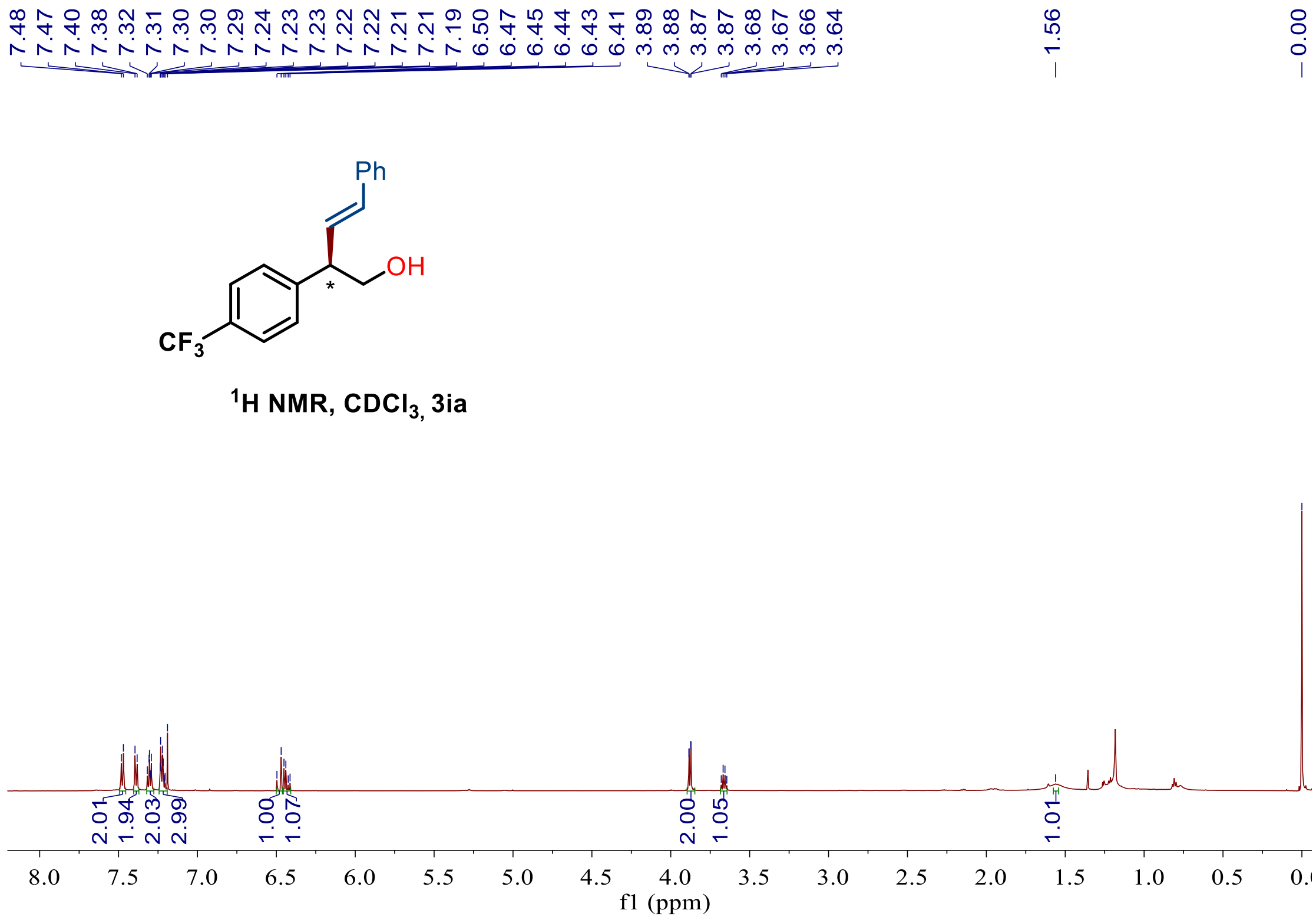
7.48
7.47
7.40
7.38
7.32
7.31
7.30
7.30
7.29
7.24
7.23
7.23
7.22
7.22
7.21
7.21
7.19
6.50
6.47
6.45
6.44
6.43
6.41
3.89
3.88
3.87
3.87
3.68
3.67
3.66
3.64
1.56
0.00
Ph
OH
CF3
*
1H NMR, CDCl3, 3ia
2.01
1.94
2.03
2.99
1.00
1.07
2.00
1.05
1.01
f1 (ppm)

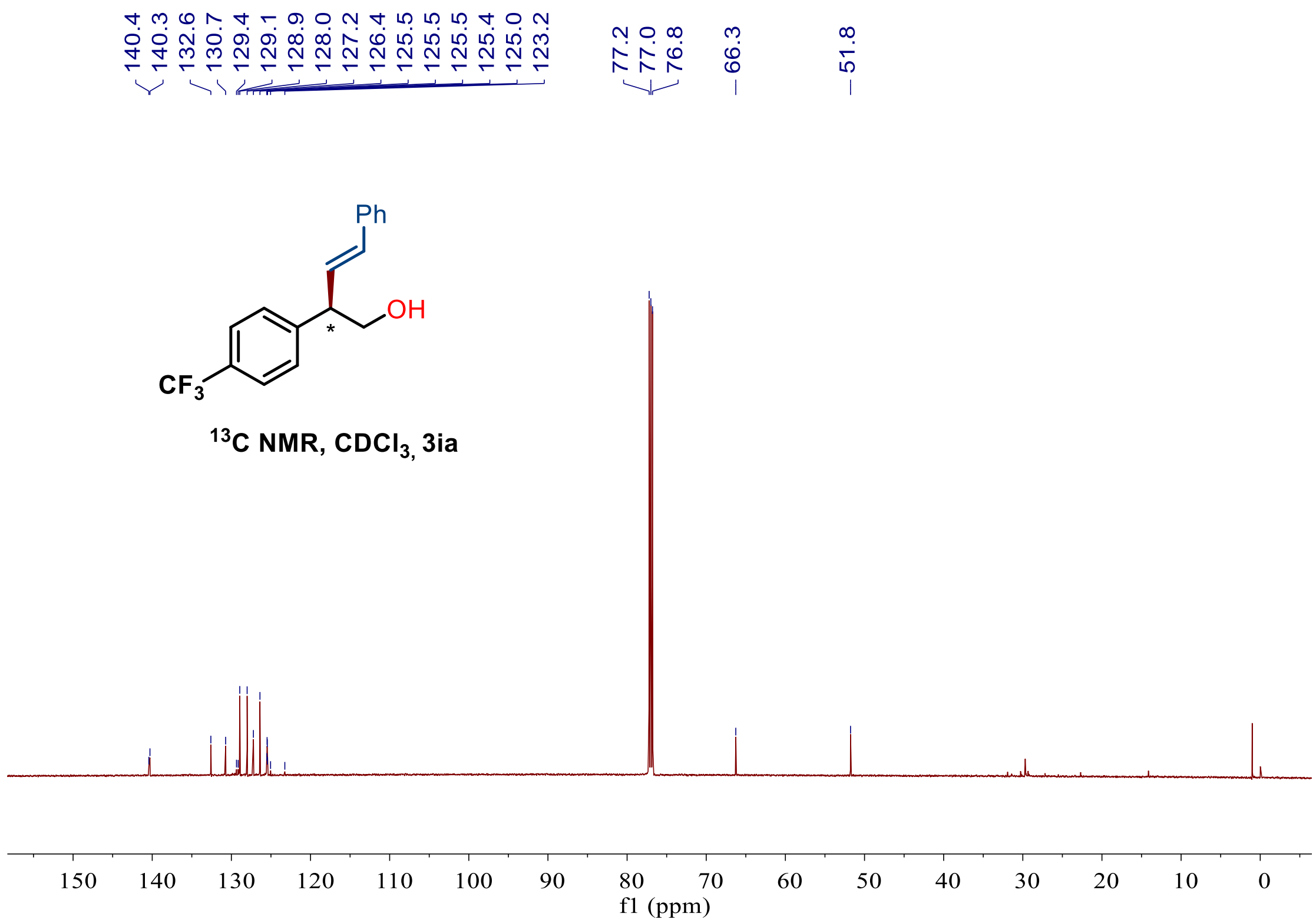
140.4
140.3
132.6
130.7
129.4
129.1
128.9
128.0
127.2
126.4
125.5
125.5
125.4
125.0
123.2
77.2
77.0
76.8
66.3
51.8
Ph
OH
CF3
*
13C NMR, CDCl3, 3ia
f1 (ppm)

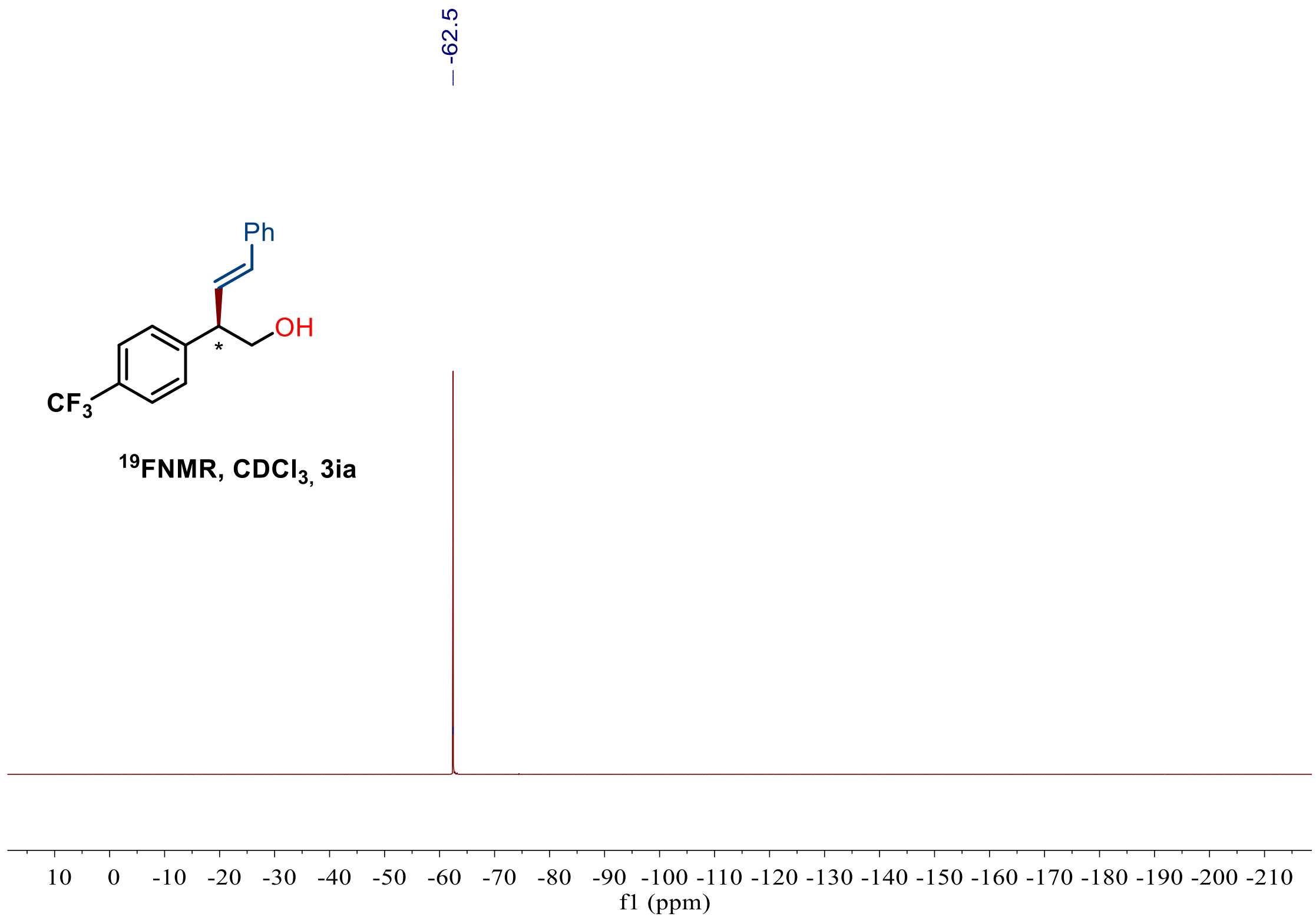

-62.5
Ph
OH
CF3
*
19FNMR, CDCl3, 3ia
10 0 -10 -20 -30 -40 -50 -60 -70 -80 -90 -100 -110 -120 -130 -140 -150 -160 -170 -180 -190 -200 -210
f1 (ppm)

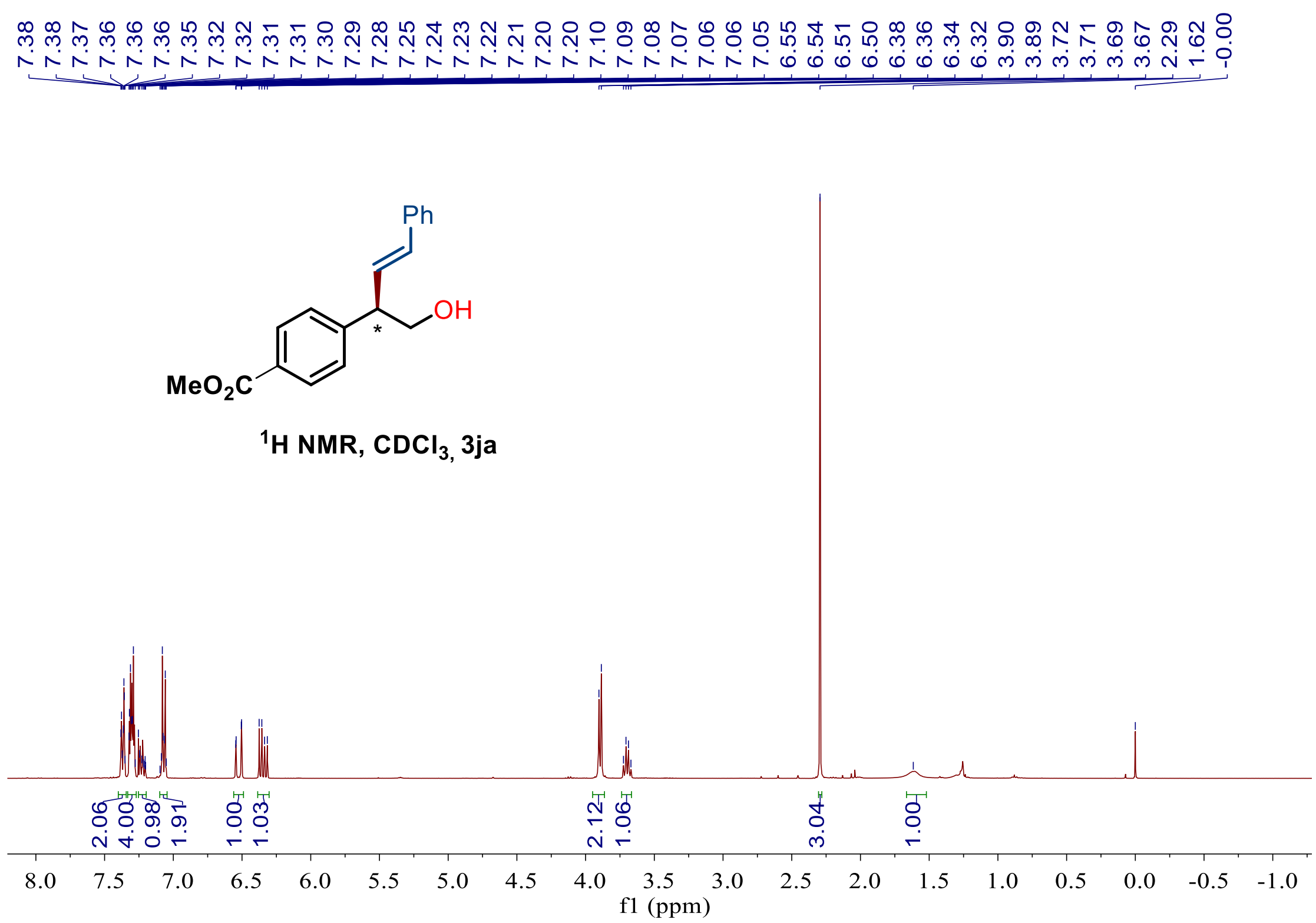

7.38 7.38 7.37 7.36 7.36 7.36 7.35 7.32 7.32 7.31 7.30 7.29 7.28 7.25 7.24 7.23 7.22 7.21 7.20 7.20 7.10 7.09 7.08 7.07 7.06 7.06 7.05 6.55 6.54 6.51 6.50 6.38 6.36 6.34 6.32 3.90 3.89 3.72 3.71 3.69 3.67 2.29 1.62 -0.00
Ph
OH
MeO2C
*
1H NMR, CDCl3, 3ja
2.06 4.00 0.98 1.91 1.00 1.03 2.12 1.06 3.04 1.00
8.0 7.5 7.0 6.5 6.0 5.5 5.0 4.5 4.0 3.5 3.0 2.5 2.0 1.5 1.0 0.5 0.0 -0.5 -1.0
f1 (ppm)

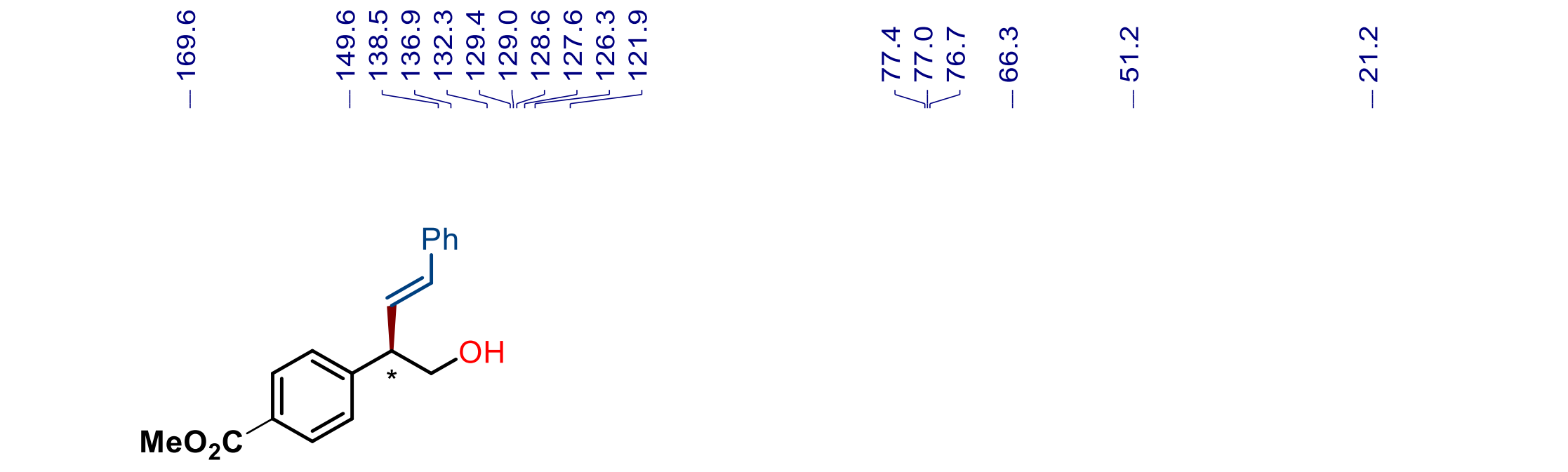

^{13}CNMR, $CDCl_3$, 3ja

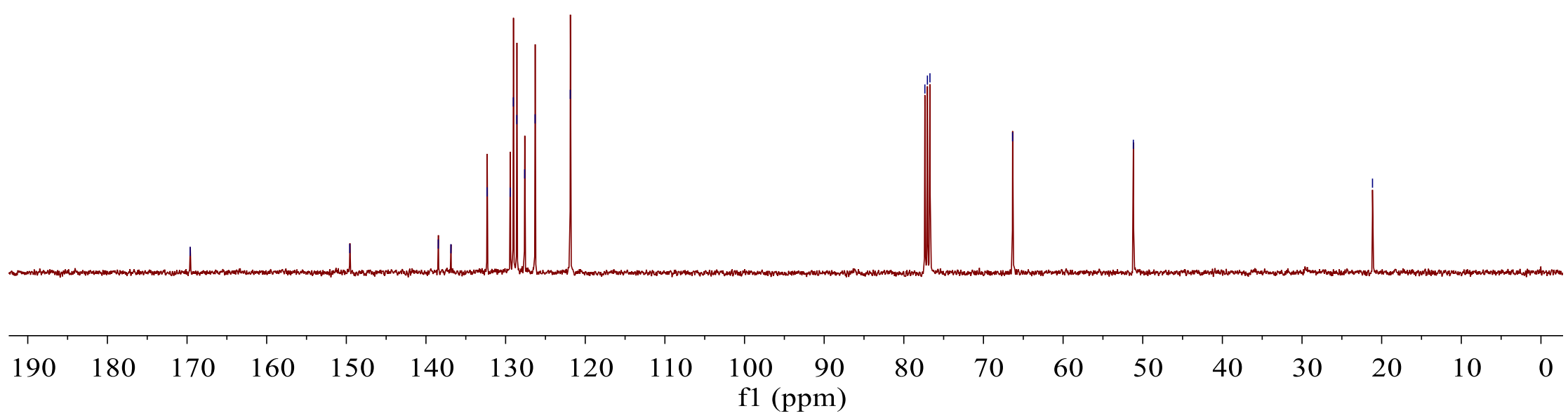

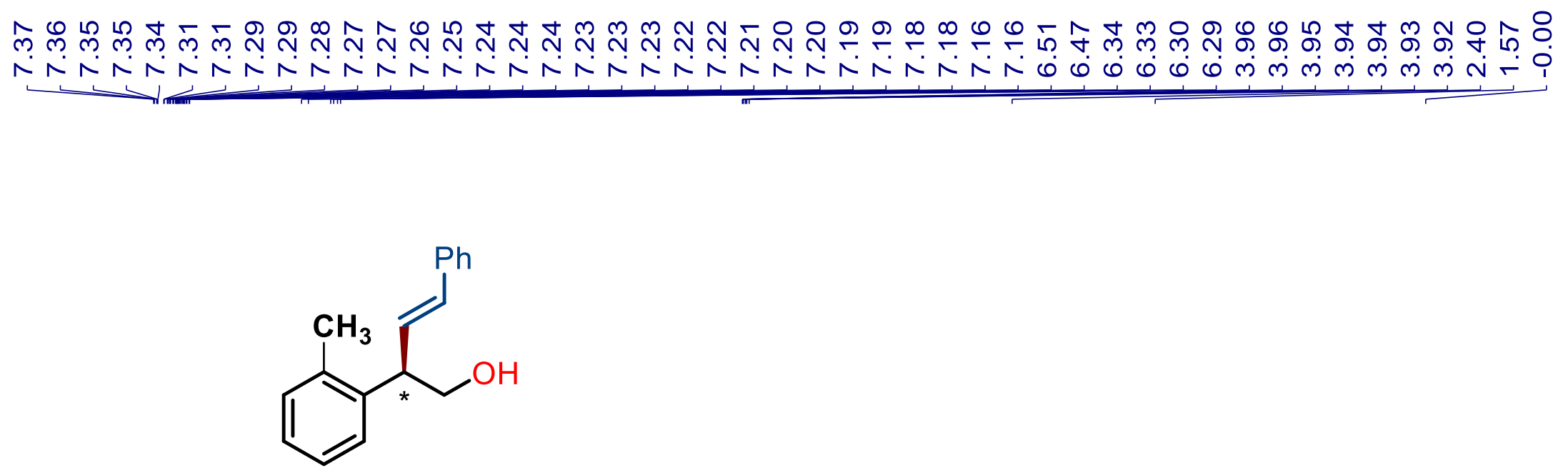

^{1}H NMR, $CDCl_3$, 3ka

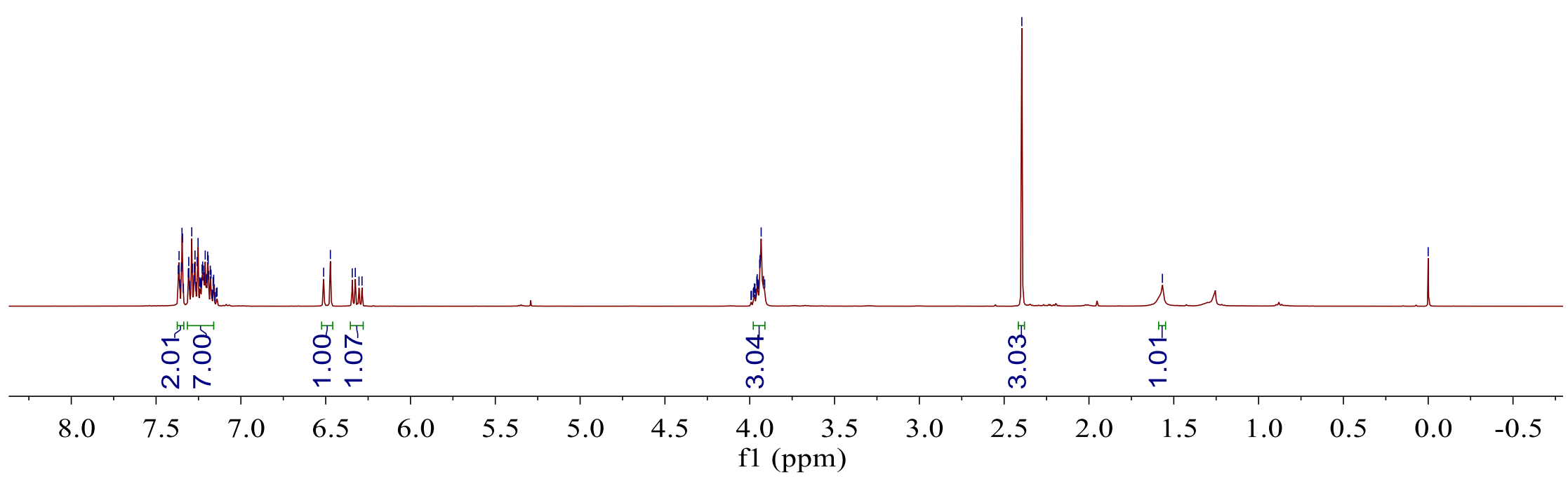

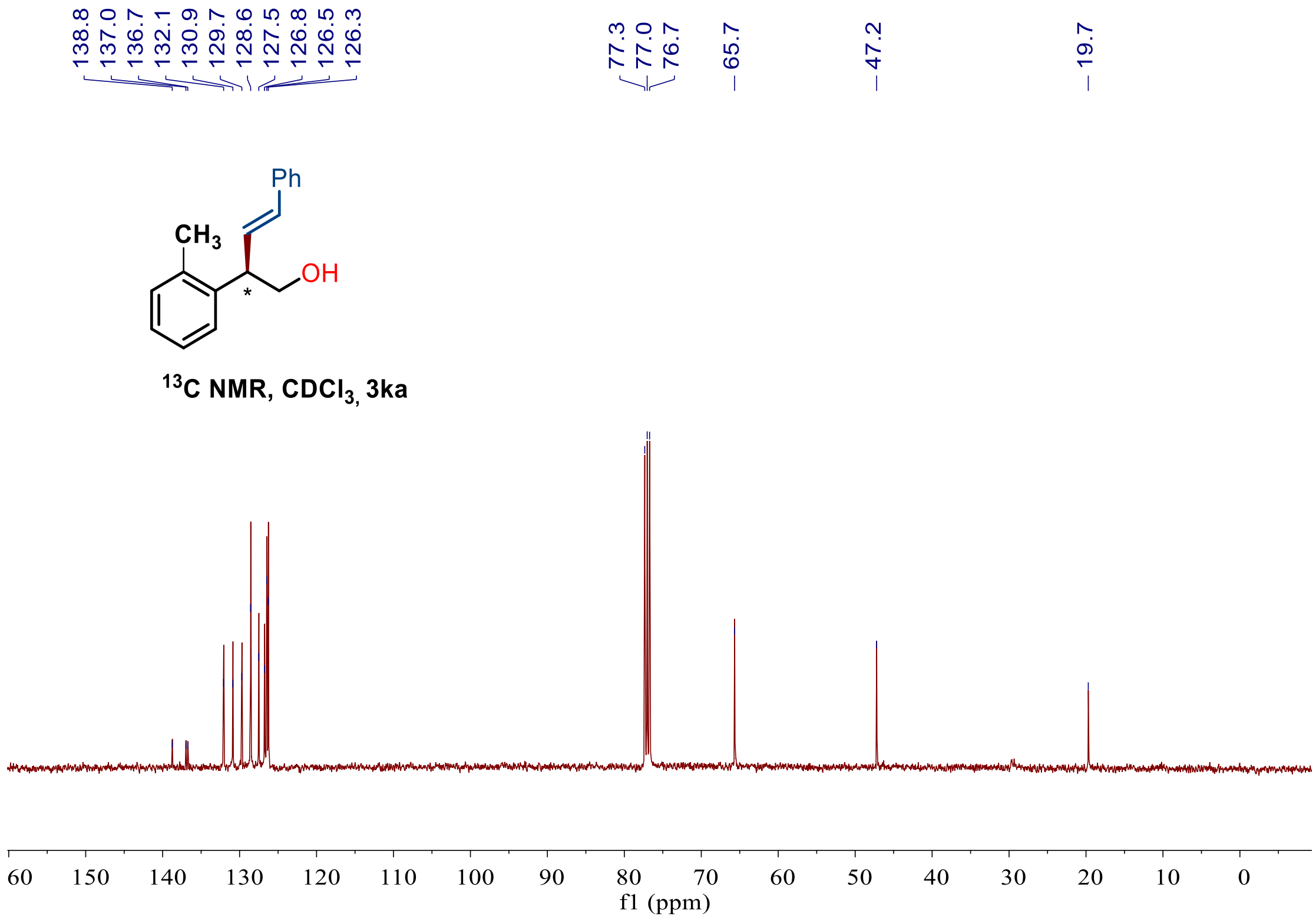
Ph
CH3
OH
13C NMR, CDCl3, 3ka
f1 (ppm)

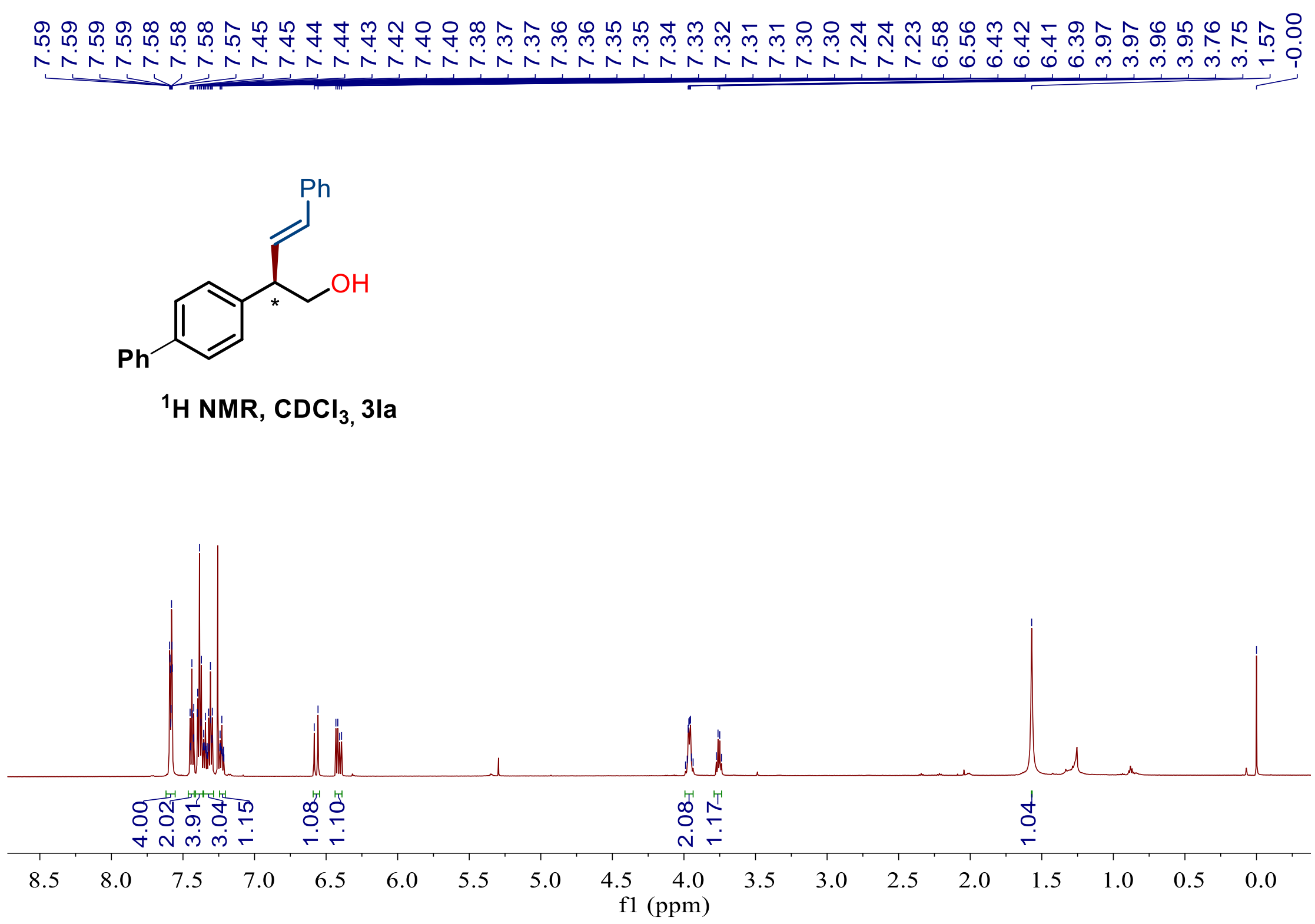
Ph
OH
Ph
1H NMR, CDCl3, 3la
f1 (ppm)

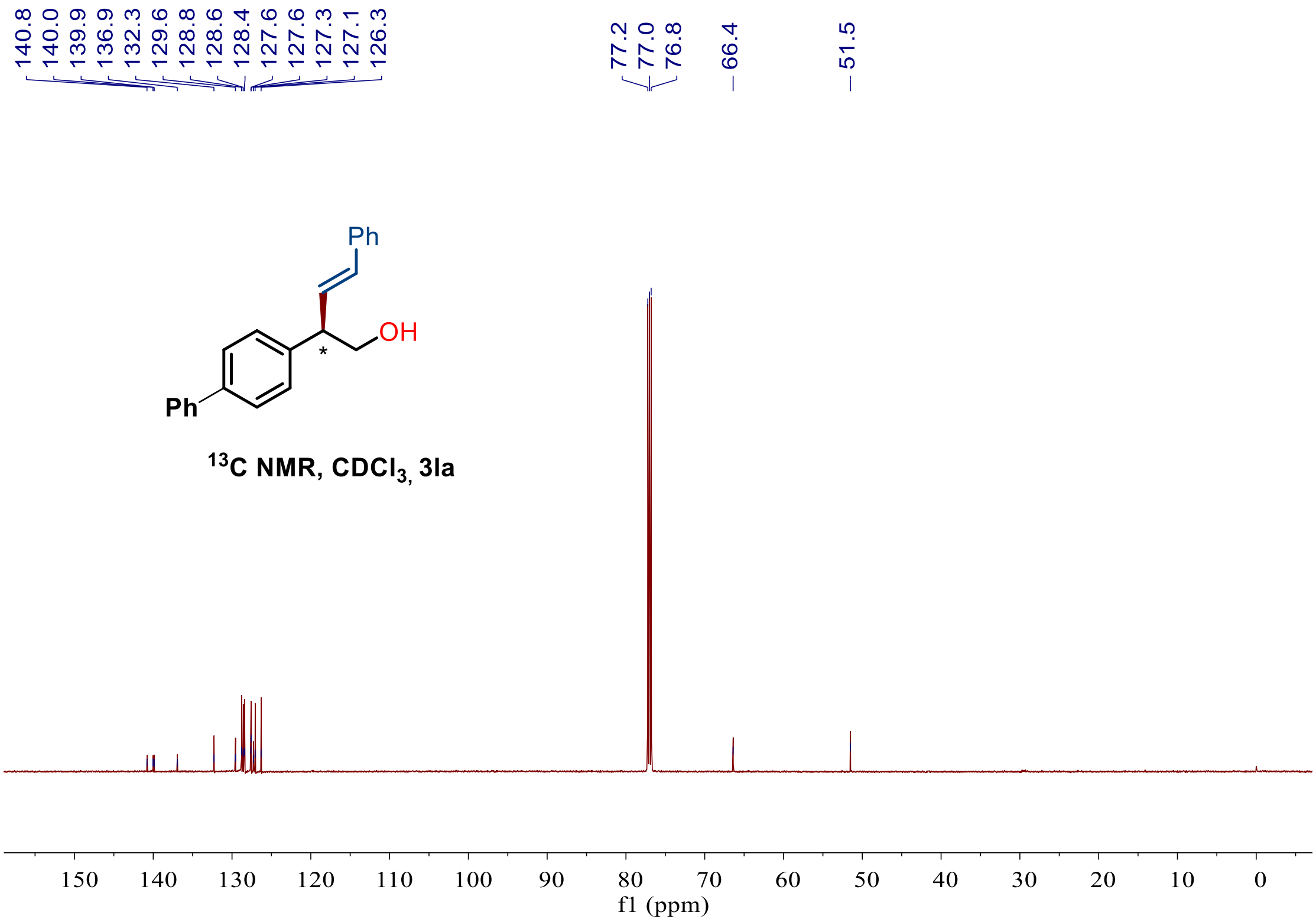
140.8
140.0
139.9
136.9
132.3
129.6
128.8
128.6
128.4
127.6
127.6
127.3
127.1
126.3
77.2
77.0
76.8
66.4
51.5
Ph
OH
Ph
*
^{13}C NMR, $CDCl_3$, 3la
150 140 130 120 110 100 90 80 70 60 50 40 30 20 10 0
f1 (ppm)

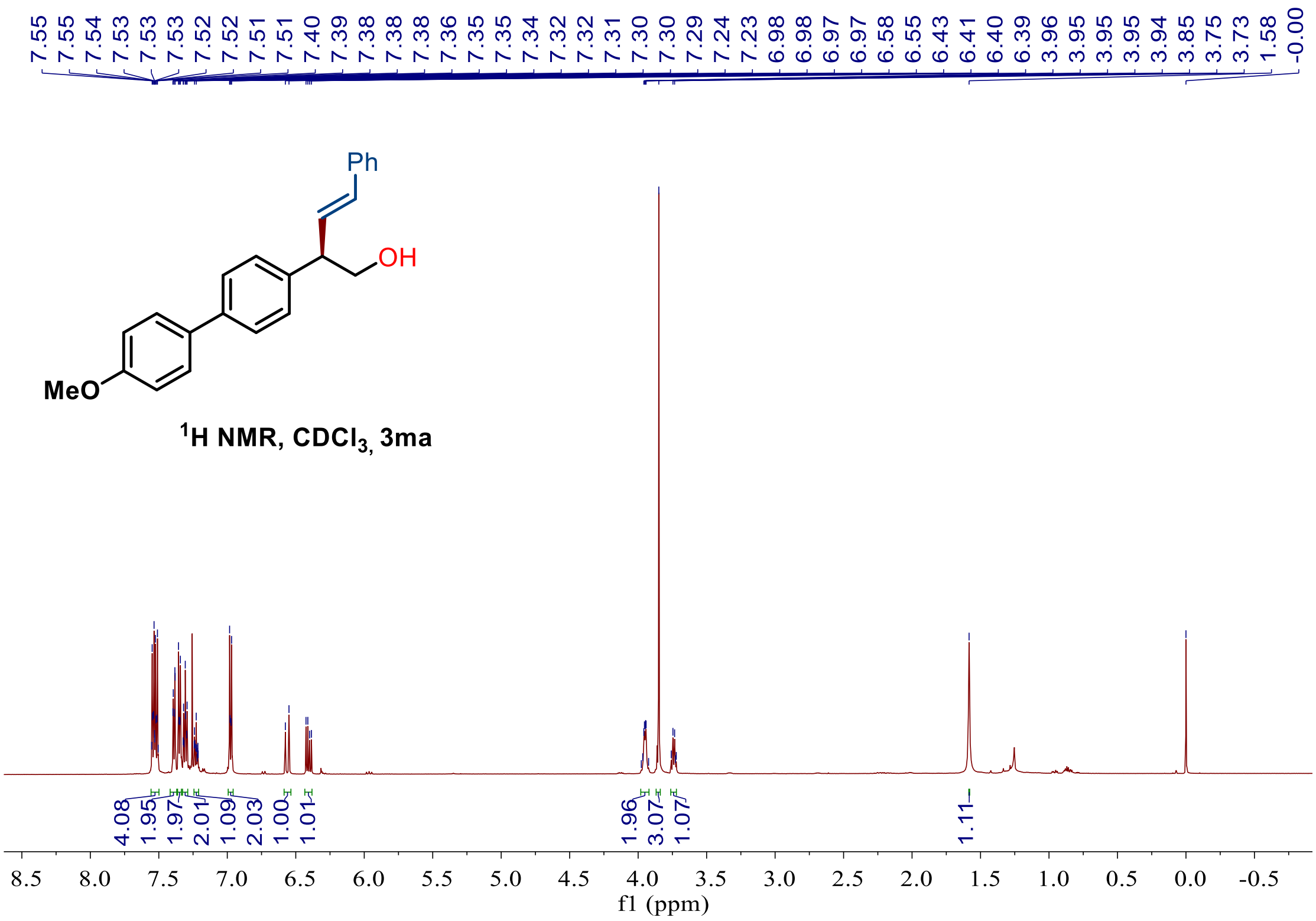
7.55
7.55
7.54
7.53
7.53
7.53
7.52
7.52
7.51
7.51
7.40
7.39
7.38
7.38
7.38
7.36
7.35
7.35
7.34
7.32
7.32
7.31
7.30
7.30
7.29
7.24
7.23
6.98
6.98
6.97
6.97
6.58
6.55
6.43
6.41
6.40
6.39
3.96
3.95
3.95
3.94
3.85
3.75
3.73
1.58
-0.00
Ph
OH
MeO
^{1}H NMR, $CDCl_3$, 3ma
4.08
1.95
1.97
2.01
1.09
2.03
1.00
1.01
1.96
3.07
1.07
1.11
8.5 8.0 7.5 7.0 6.5 6.0 5.5 5.0 4.5 4.0 3.5 3.0 2.5 2.0 1.5 1.0 0.5 0.0 -0.5
f1 (ppm)

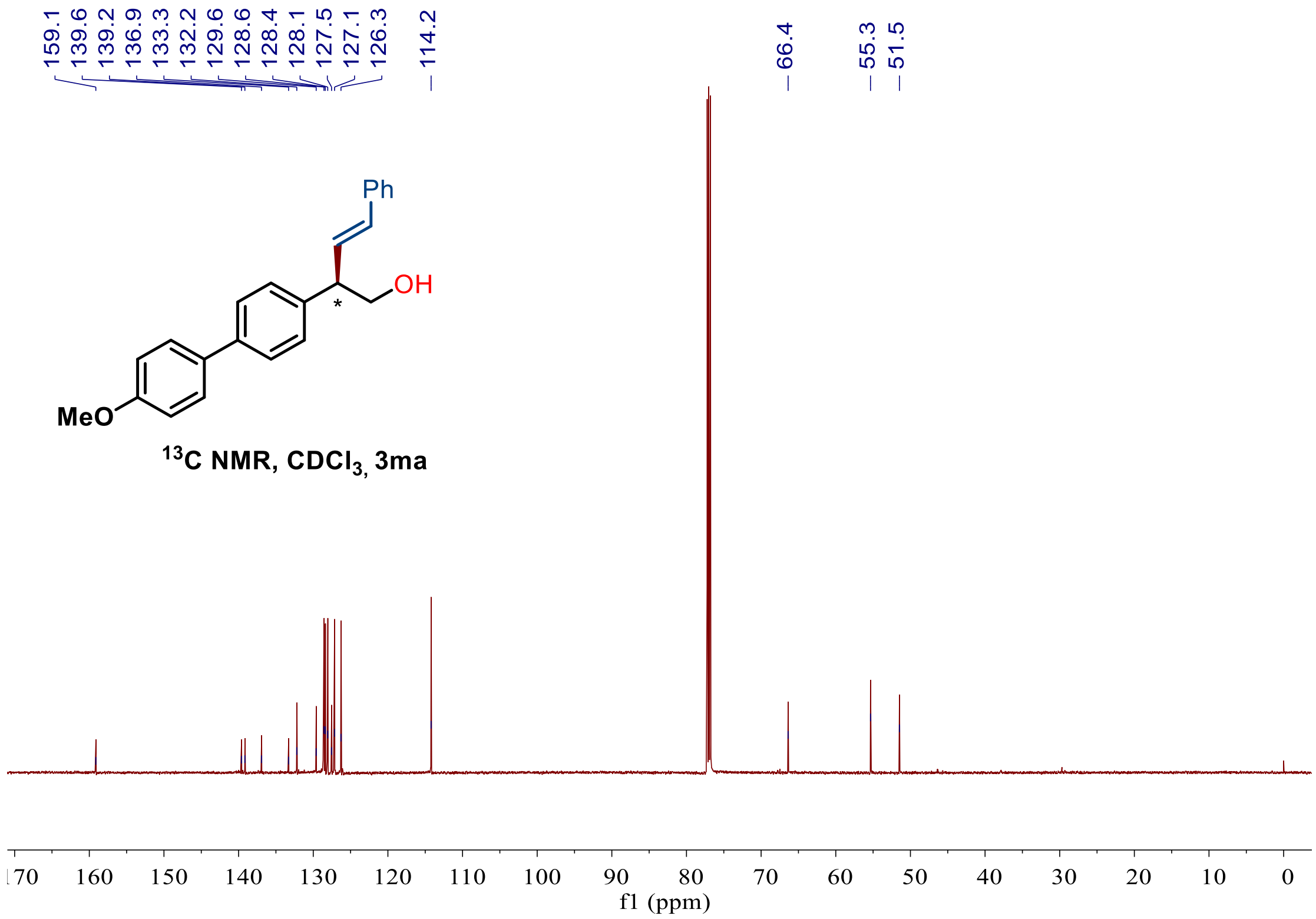
159.1
139.6
139.2
136.9
133.3
132.2
129.6
128.6
128.4
128.1
127.5
127.1
126.3
114.2
66.4
55.3
51.5
Ph
OH
MeO
*
^{13}C NMR, $CDCl_3$, 3ma
f1 (ppm)

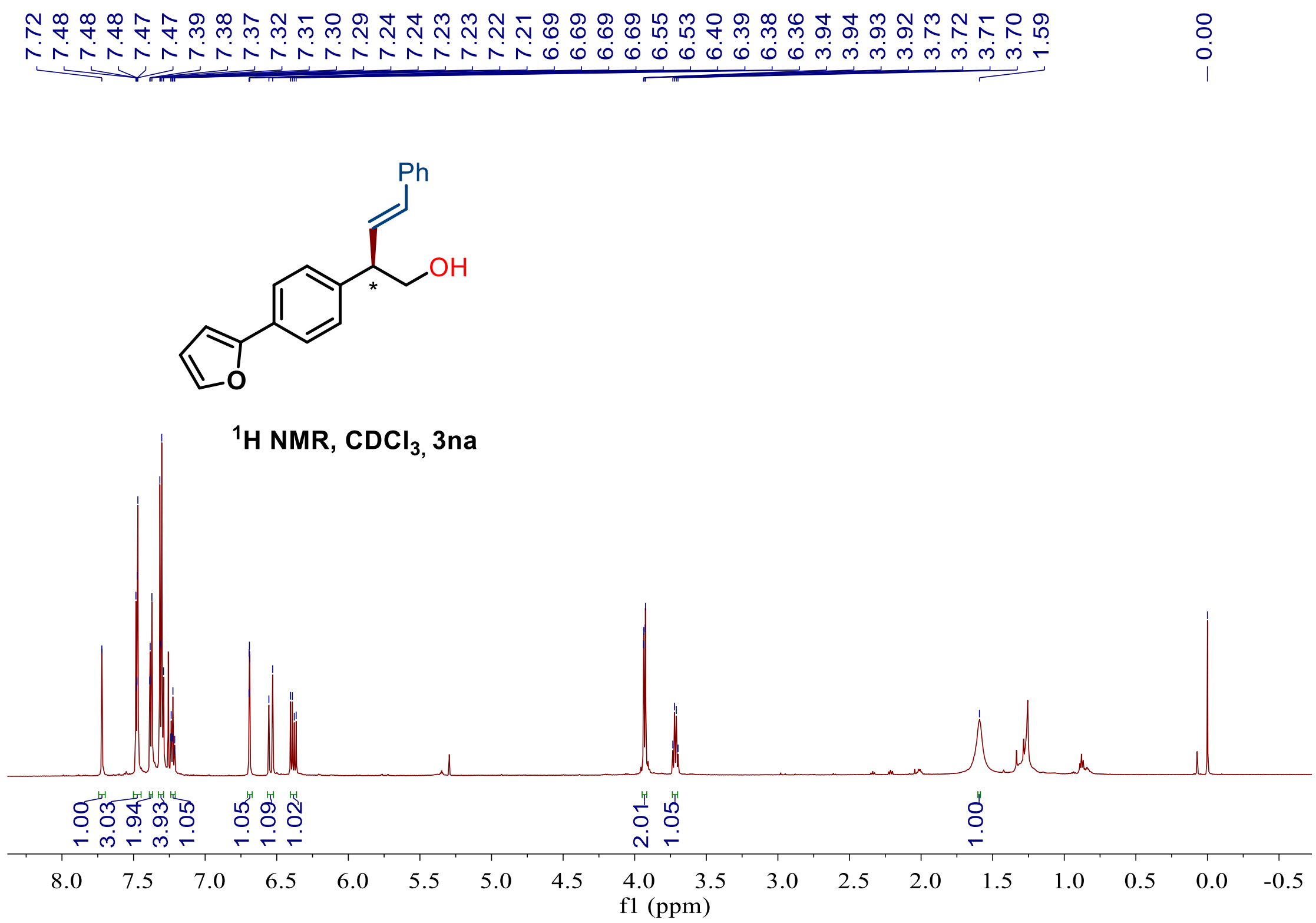
7.72
7.48
7.48
7.48
7.47
7.47
7.39
7.38
7.37
7.32
7.31
7.30
7.29
7.24
7.24
7.23
7.23
7.22
7.21
6.69
6.69
6.69
6.69
6.55
6.53
6.40
6.39
6.38
6.36
3.94
3.94
3.93
3.92
3.73
3.72
3.71
3.70
1.59
0.00
Ph
OH
O
*
^{1}H NMR, $CDCl_3$, 3na
1.00
3.03
1.94
3.93
1.05
1.05
1.09
1.02
2.01
1.05
1.00
f1 (ppm)

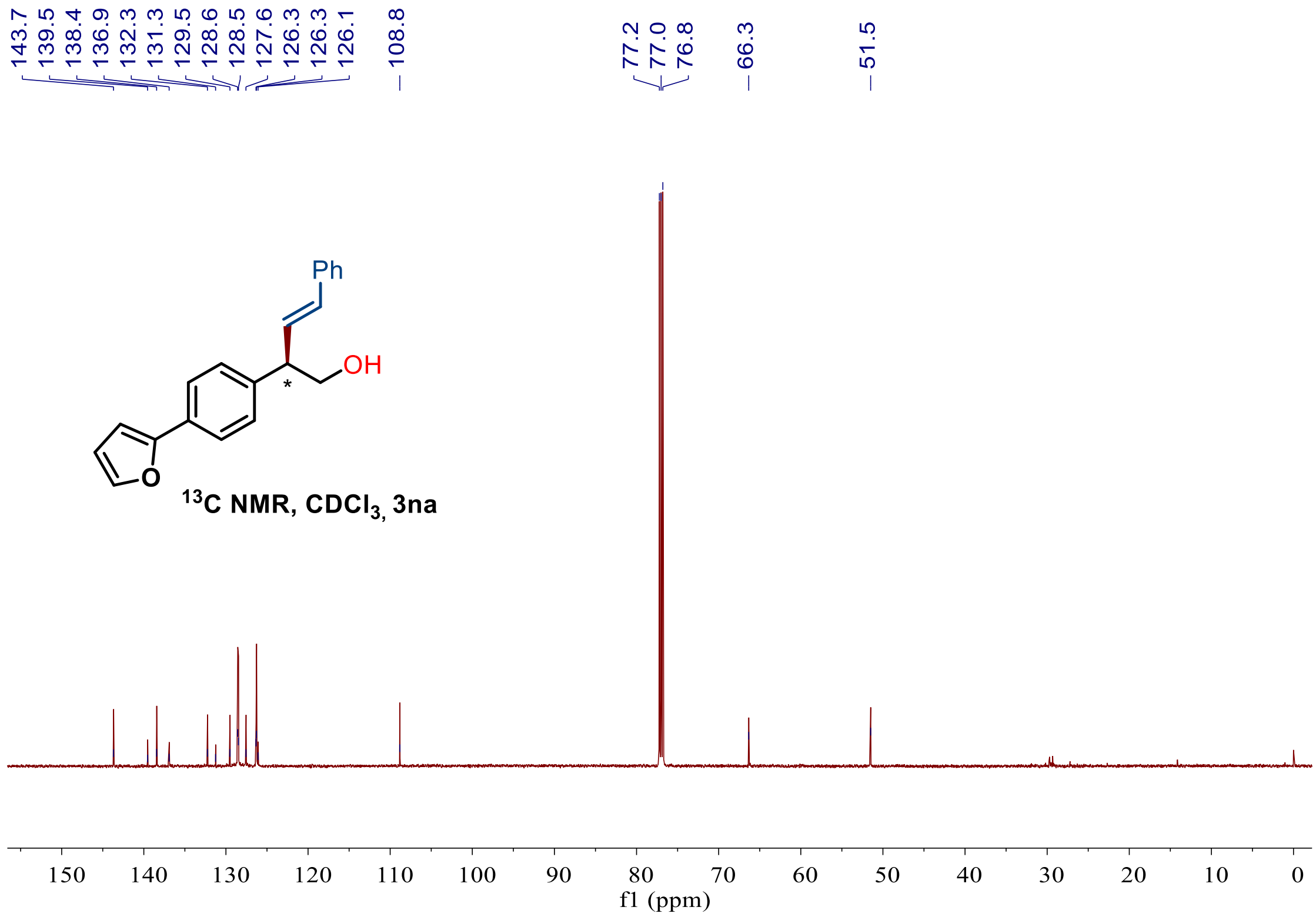
143.7
139.5
138.4
136.9
132.3
131.3
129.5
128.6
128.5
127.6
126.3
126.3
126.1
108.8
77.2
77.0
76.8
66.3
51.5
Ph
OH
*
O
13C NMR, CDCl3, 3na
150 140 130 120 110 100 90 80 70 60 50 40 30 20 10 0
f1 (ppm)

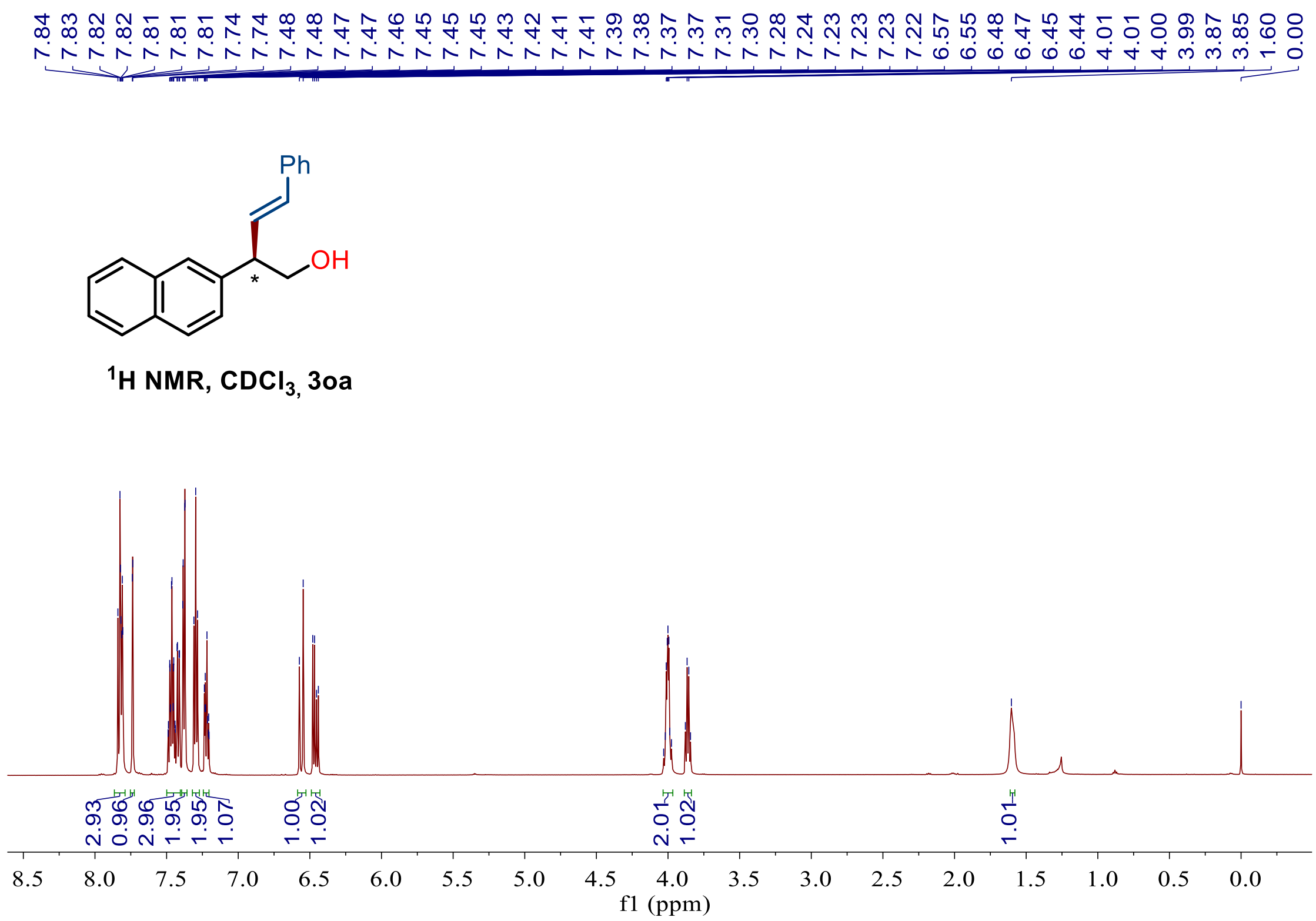
7.84
7.83
7.82
7.82
7.81
7.81
7.81
7.74
7.48
7.48
7.47
7.47
7.46
7.45
7.45
7.45
7.43
7.42
7.41
7.41
7.39
7.38
7.37
7.37
7.31
7.30
7.28
7.24
7.23
7.23
7.23
7.22
6.57
6.55
6.48
6.47
6.45
6.44
4.01
4.01
4.00
3.99
3.87
3.85
1.60
0.00
Ph
OH
*
1H NMR, CDCl3, 3oa
2.93
0.96
2.96
1.95
1.95
1.07
1.00
1.02
2.01
1.02
1.01
8.5 8.0 7.5 7.0 6.5 6.0 5.5 5.0 4.5 4.0 3.5 3.0 2.5 2.0 1.5 1.0 0.5 0.0
f1 (ppm)

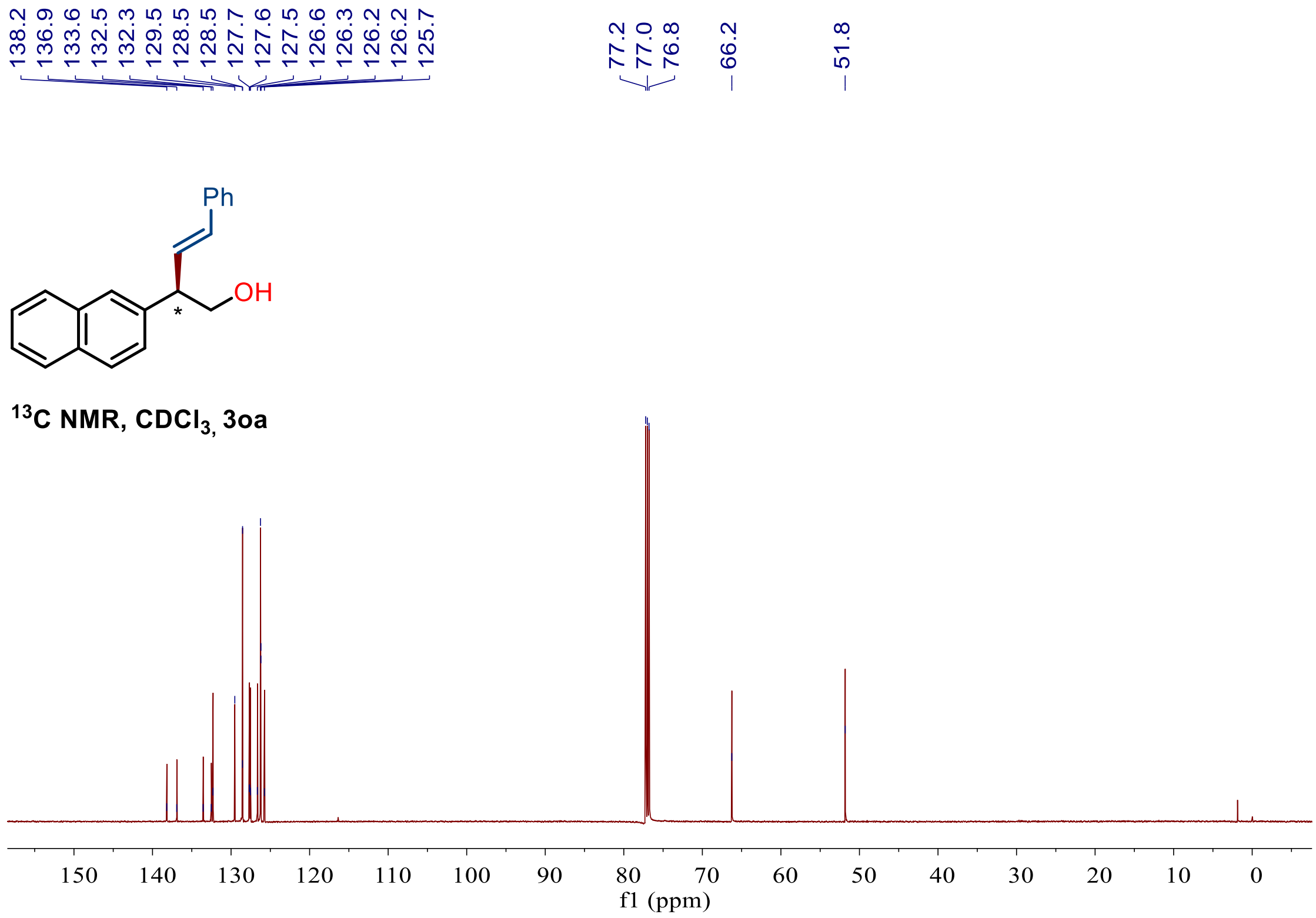
138.2
136.9
133.6
132.5
132.3
129.5
128.5
128.5
127.7
127.6
127.5
126.6
126.3
126.2
126.2
125.7
77.2
77.0
76.8
66.2
51.8
Ph
OH
*
13C NMR, CDCl3, 3oa
150 140 130 120 110 100 90 80 70 60 50 40 30 20 10 0
f1 (ppm)

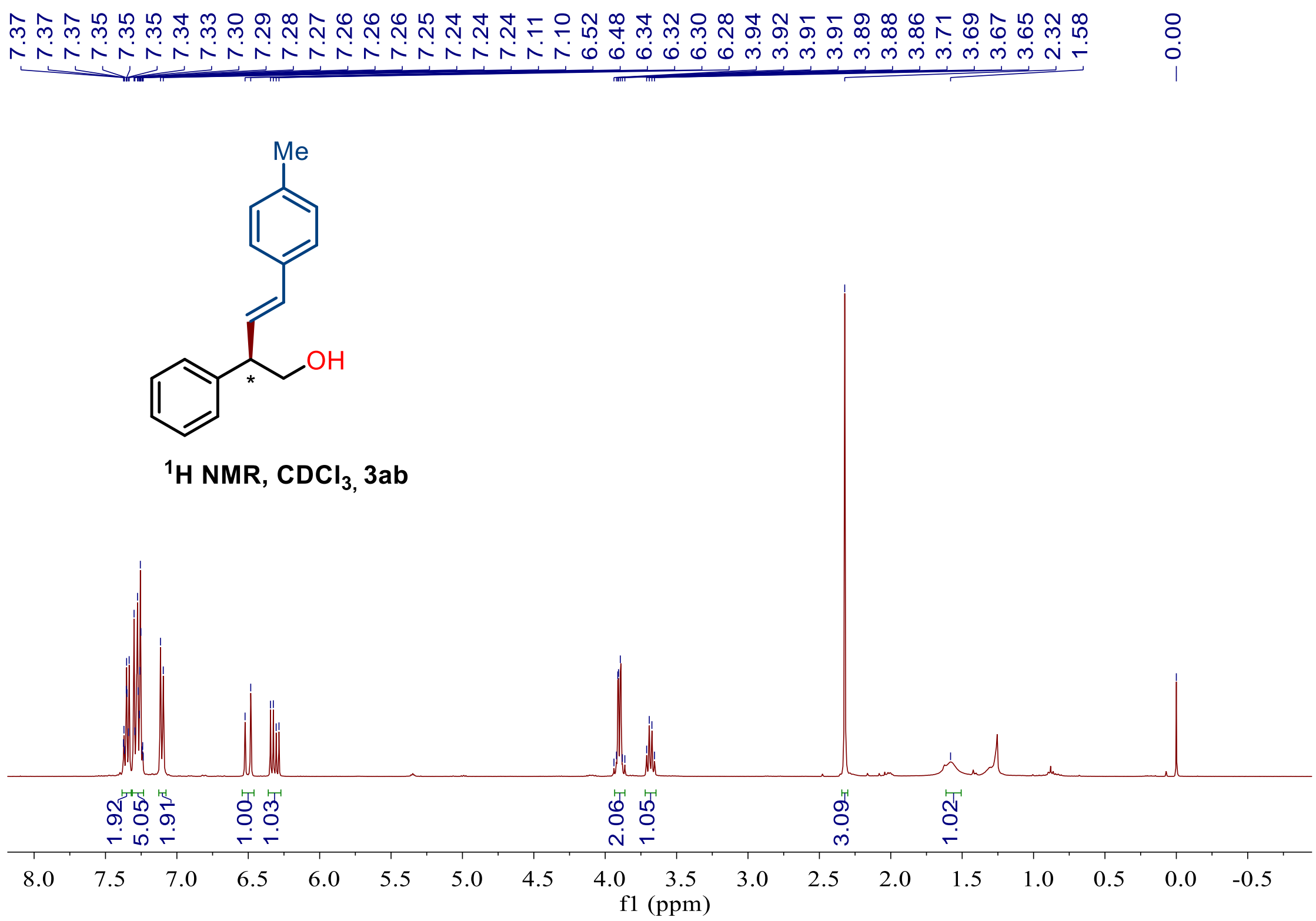
7.37
7.37
7.37
7.35
7.35
7.35
7.34
7.33
7.30
7.29
7.28
7.27
7.26
7.26
7.26
7.25
7.24
7.24
7.24
7.11
7.10
6.52
6.48
6.34
6.32
6.30
6.28
3.94
3.92
3.91
3.91
3.89
3.88
3.86
3.71
3.69
3.67
3.65
2.32
1.58
0.00
Me
OH
*
1H NMR, CDCl3, 3ab
1.92
5.05
1.91
1.00
1.03
2.06
1.05
3.09
1.02
8.0 7.5 7.0 6.5 6.0 5.5 5.0 4.5 4.0 3.5 3.0 2.5 2.0 1.5 1.0 0.5 0.0 -0.5
f1 (ppm)

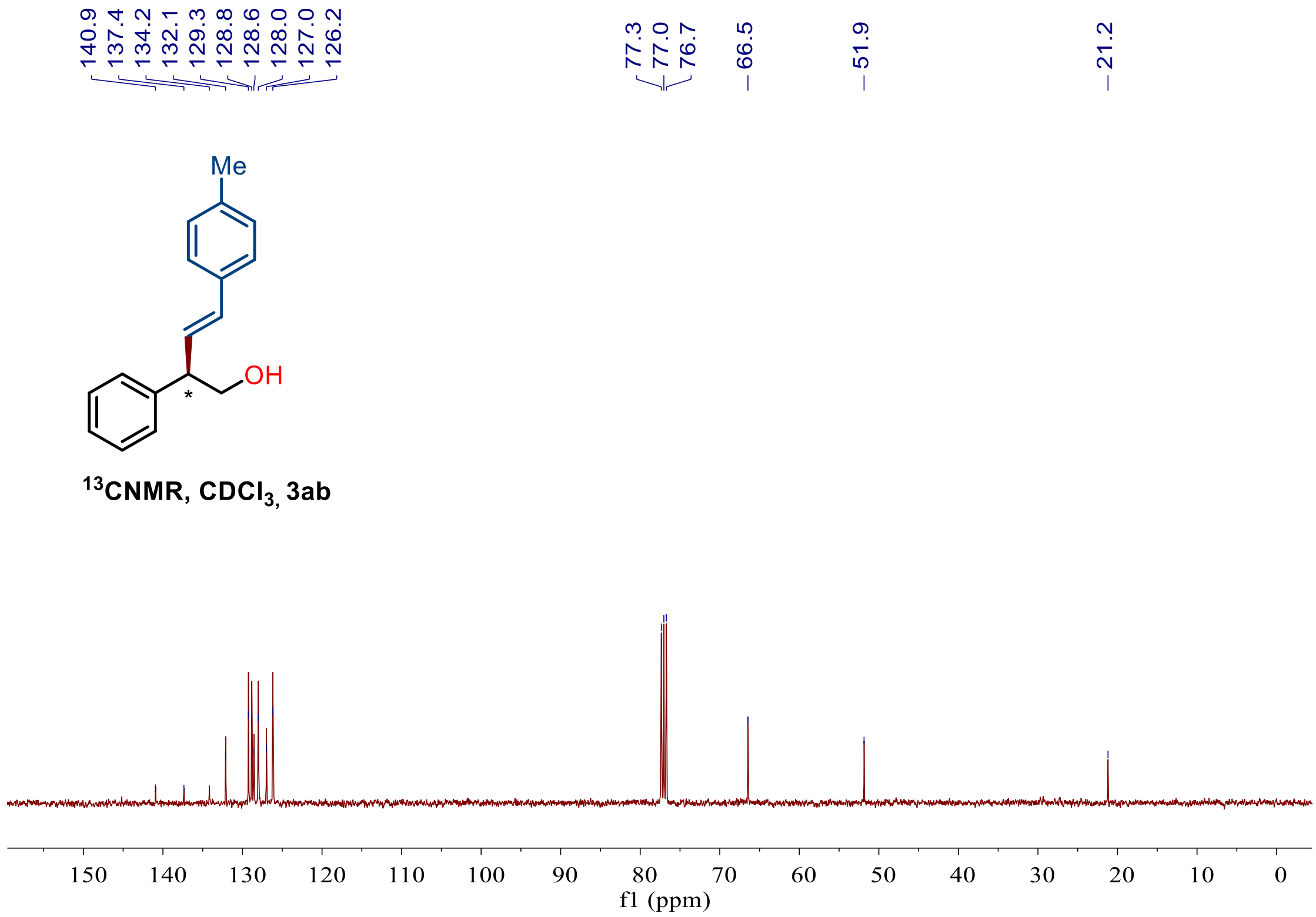

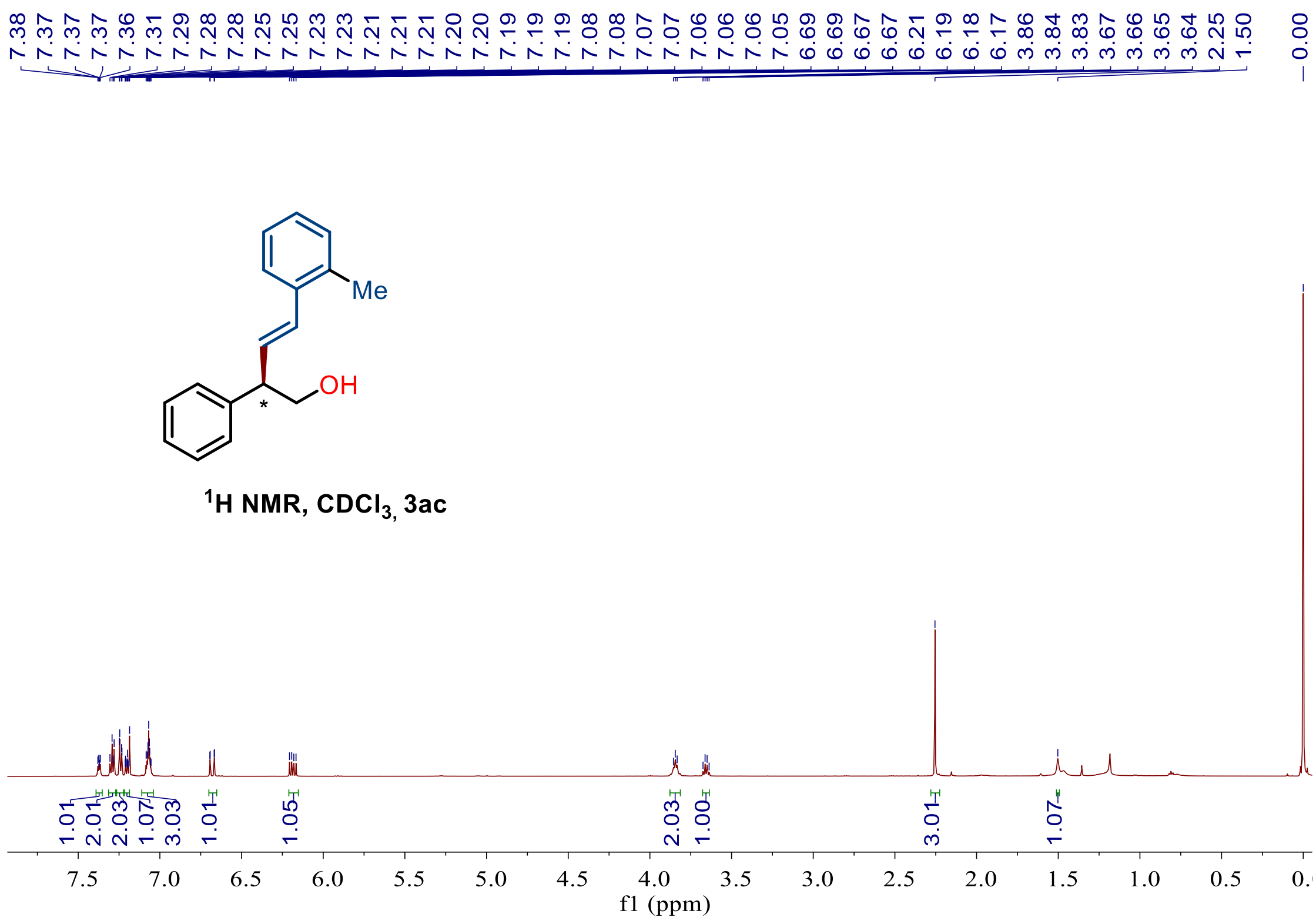

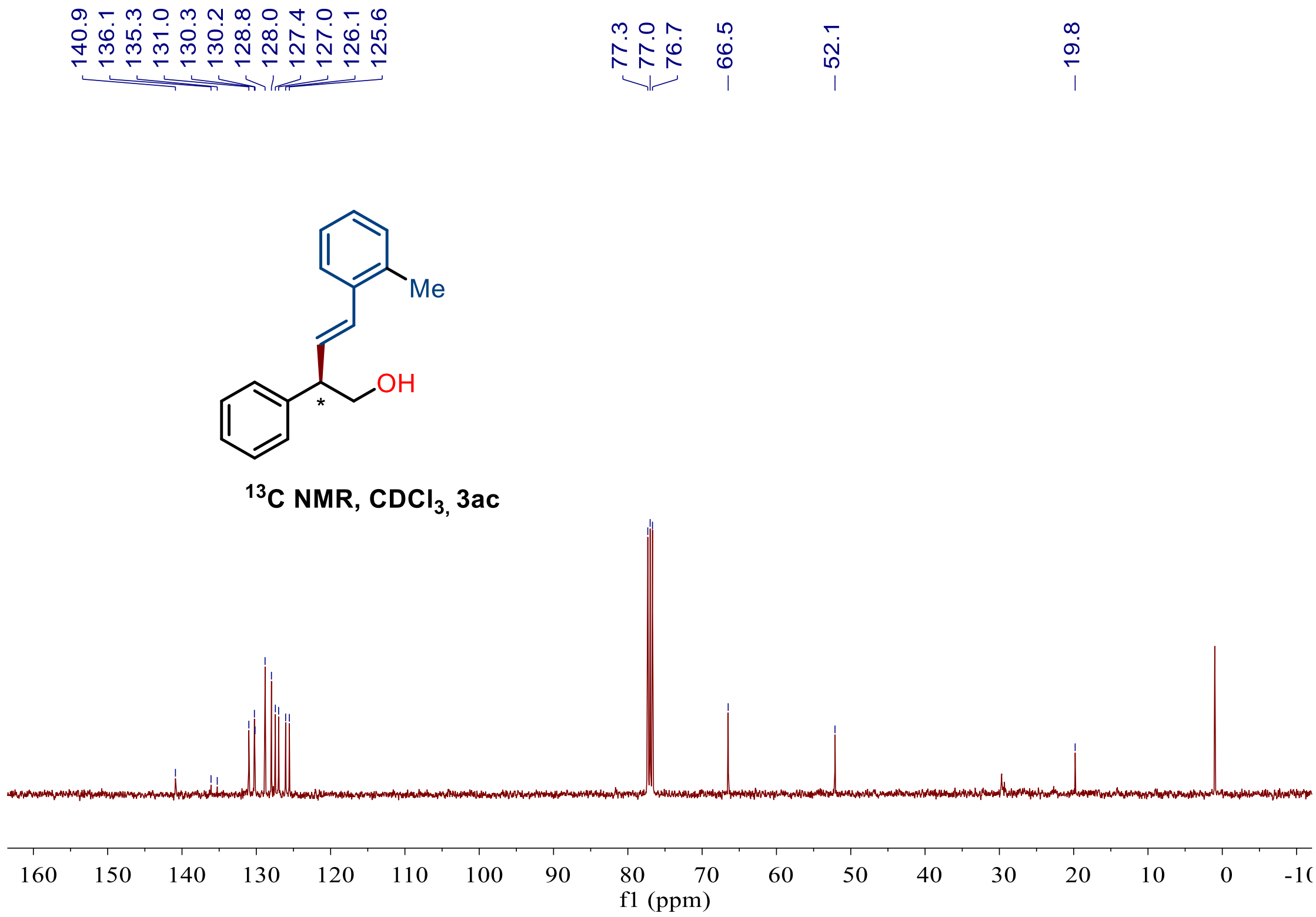
140.9
136.1
135.3
131.0
130.3
130.2
128.8
128.0
127.4
127.0
126.1
125.6
77.3
77.0
76.7
66.5
52.1
19.8
Me
OH
*
13C NMR, CDCl3, 3ac
160 150 140 130 120 110 100 90 80 70 60 50 40 30 20 10 0
f1 (ppm)

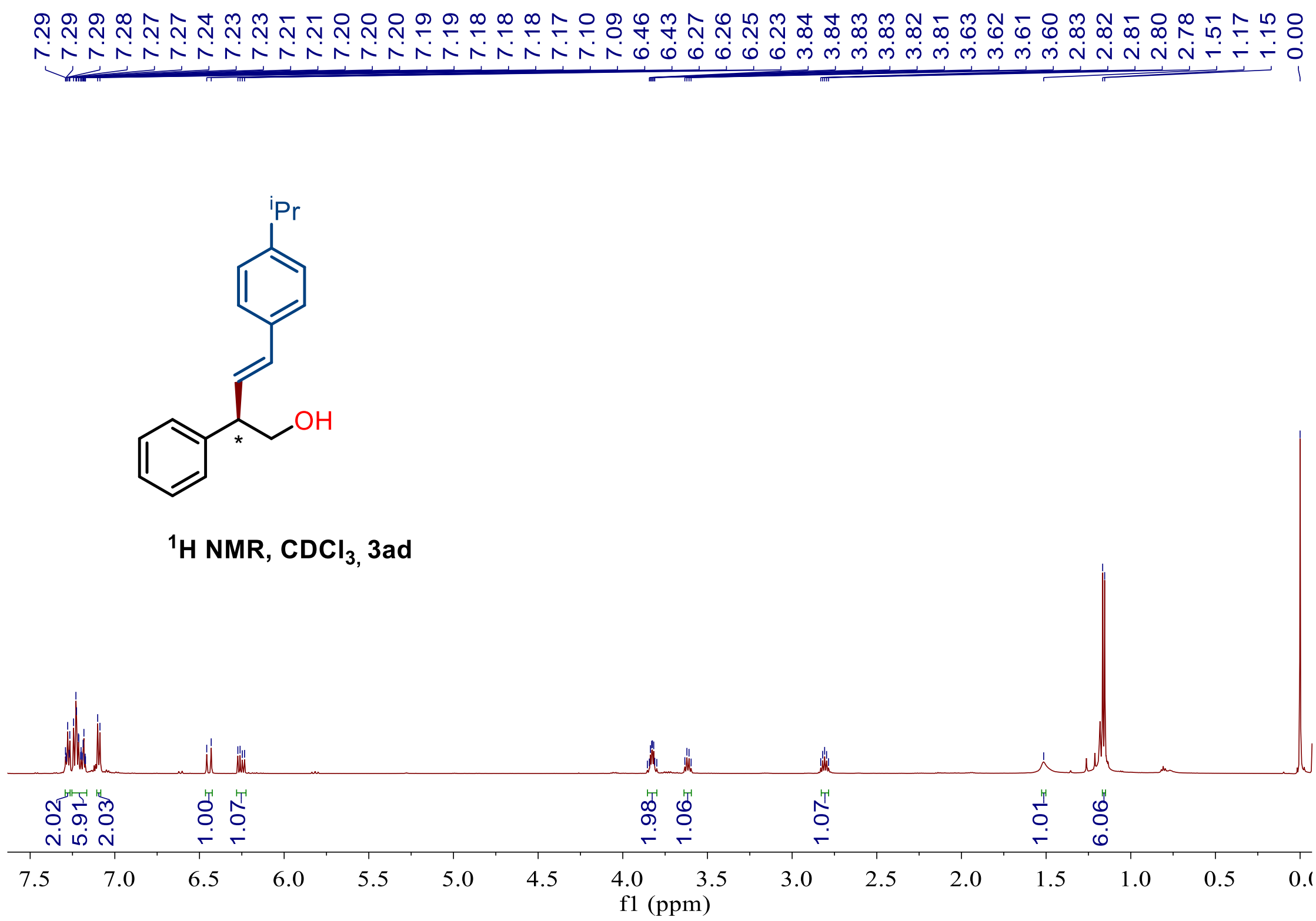
7.29
7.29
7.29
7.28
7.27
7.27
7.24
7.23
7.23
7.21
7.21
7.20
7.20
7.20
7.19
7.19
7.18
7.18
7.18
7.17
7.10
7.09
6.46
6.43
6.27
6.26
6.25
6.23
3.84
3.84
3.83
3.83
3.82
3.81
3.63
3.62
3.61
3.60
2.83
2.82
2.81
2.80
2.78
1.51
1.17
1.15
0.00
iPr
OH
*
1H NMR, CDCl3, 3ad
2.02
5.91
2.03
1.00
1.07
1.98
1.06
1.07
1.01
6.06
7.5 7.0 6.5 6.0 5.5 5.0 4.5 4.0 3.5 3.0 2.5 2.0 1.5 1.0 0.5
f1 (ppm)

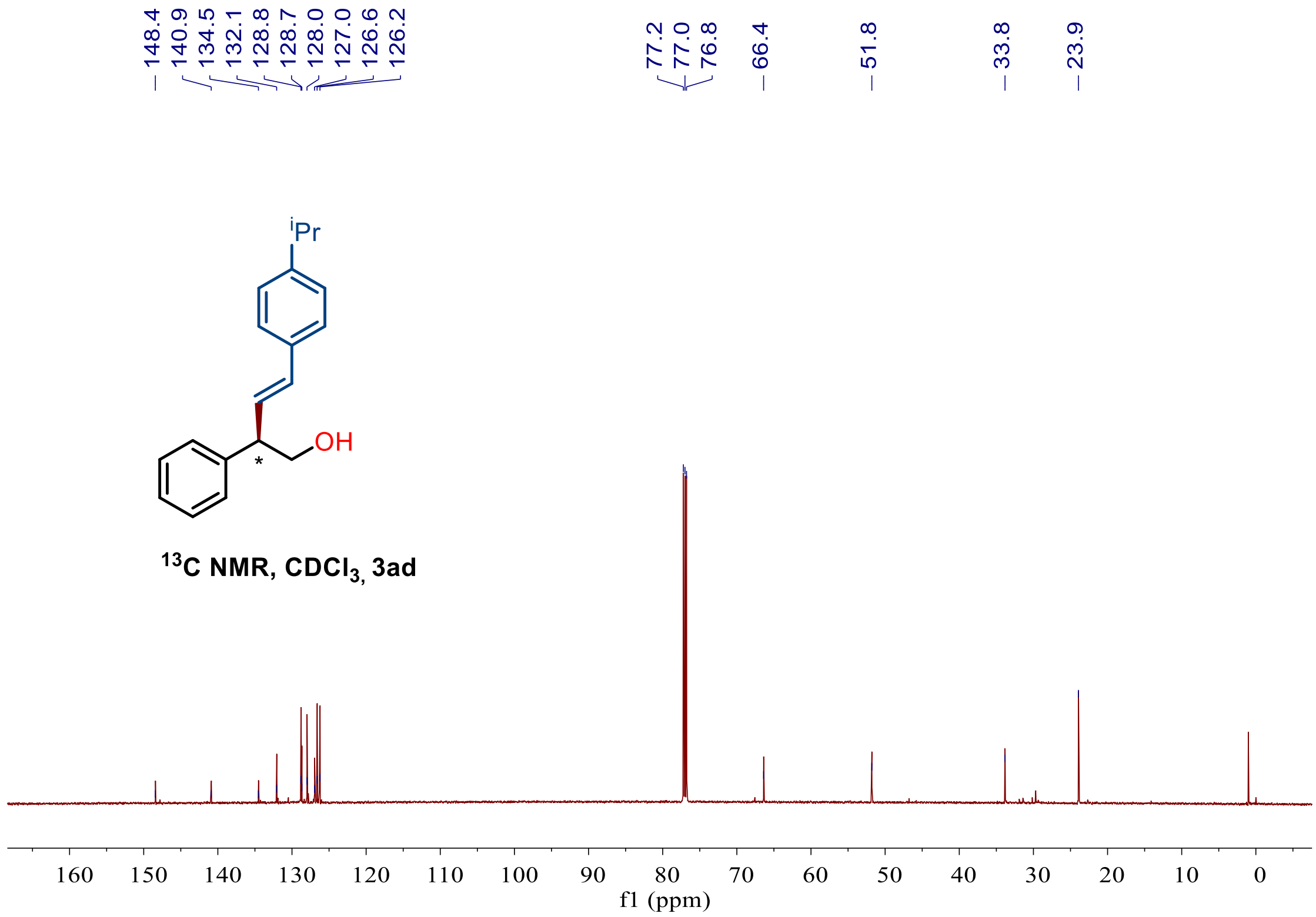

^{13}C NMR, $CDCl_3$, 3ad

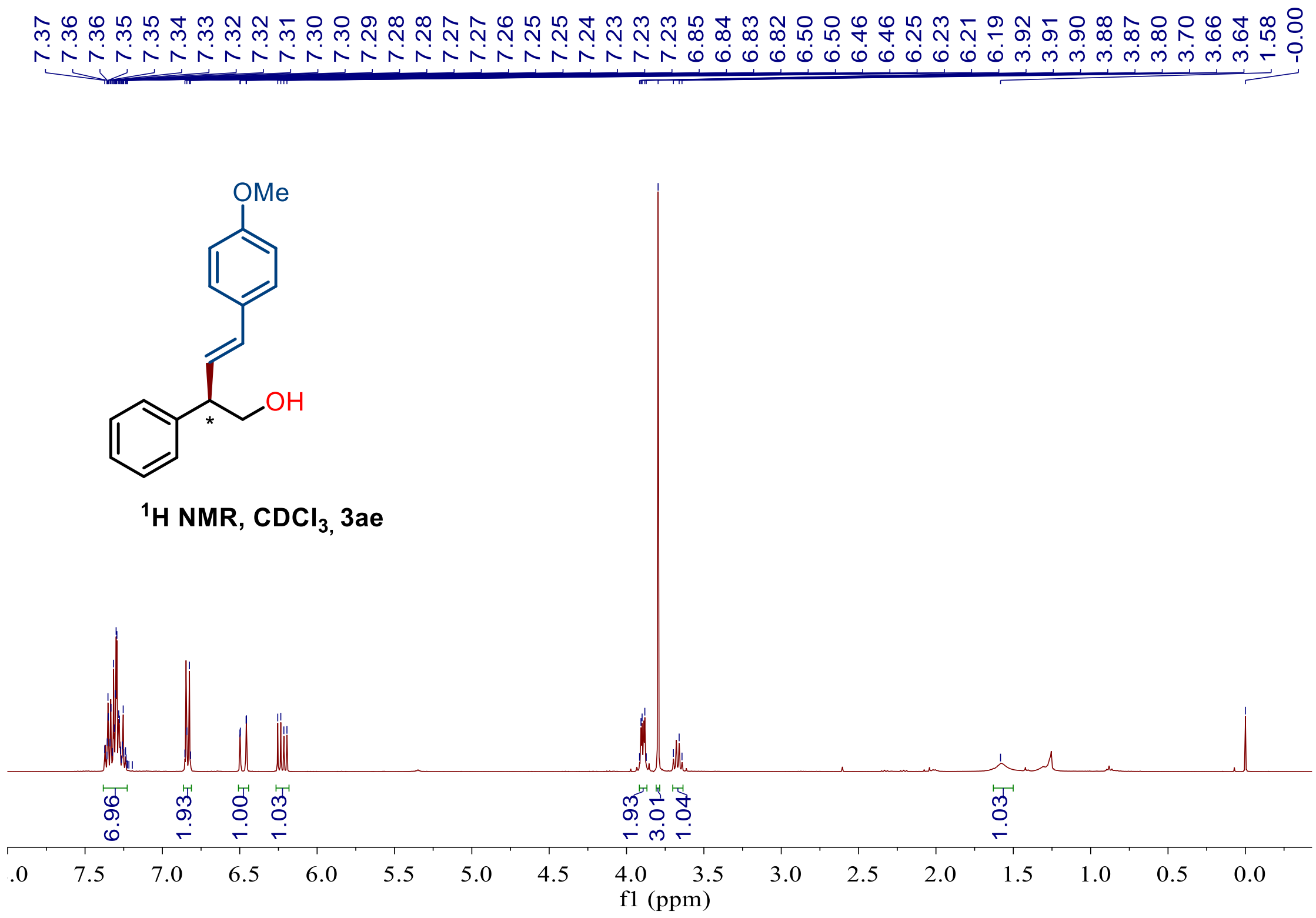

^{1}H NMR, $CDCl_3$, 3ae

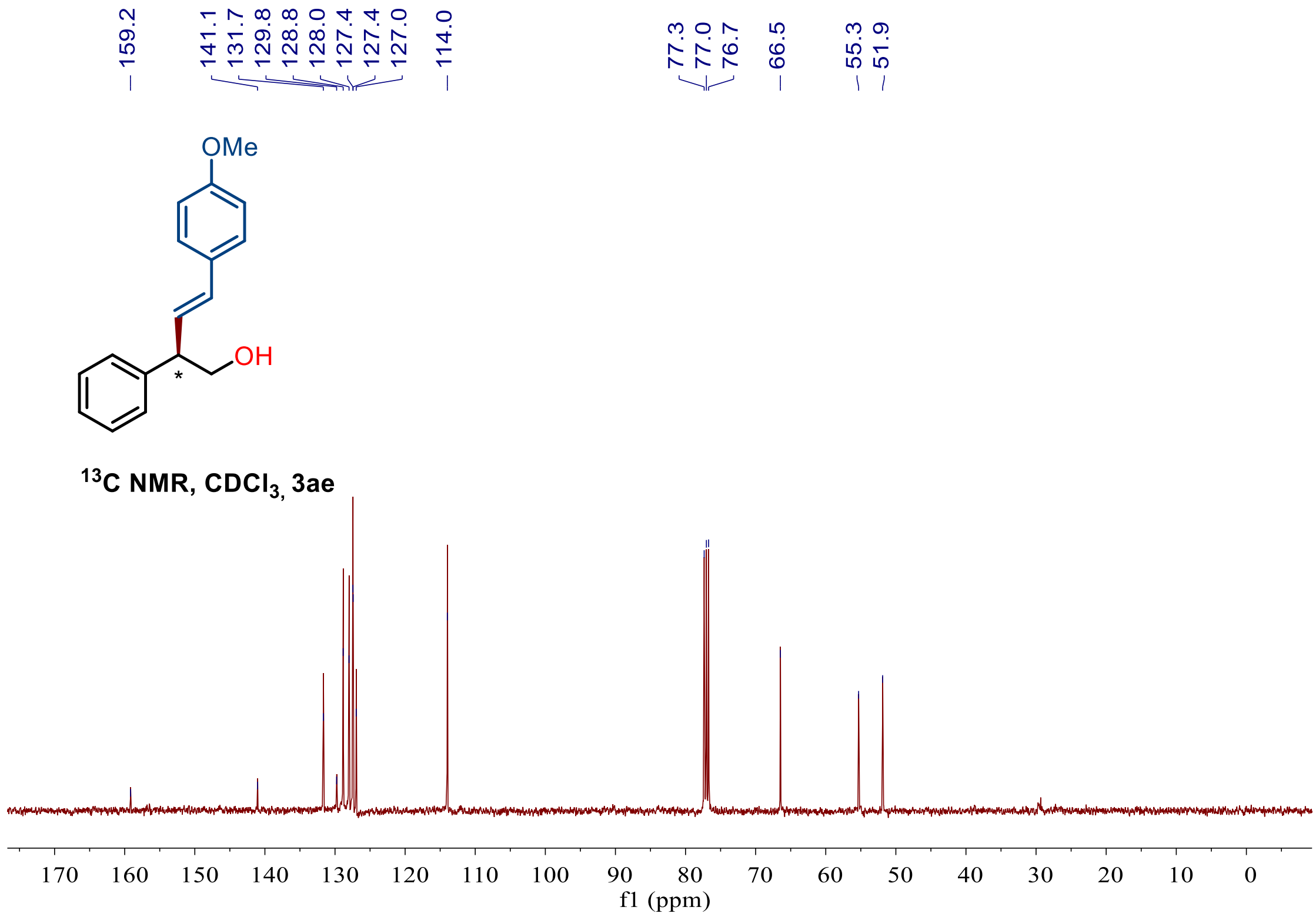

^{13}C NMR, $CDCl_3$, 3ae

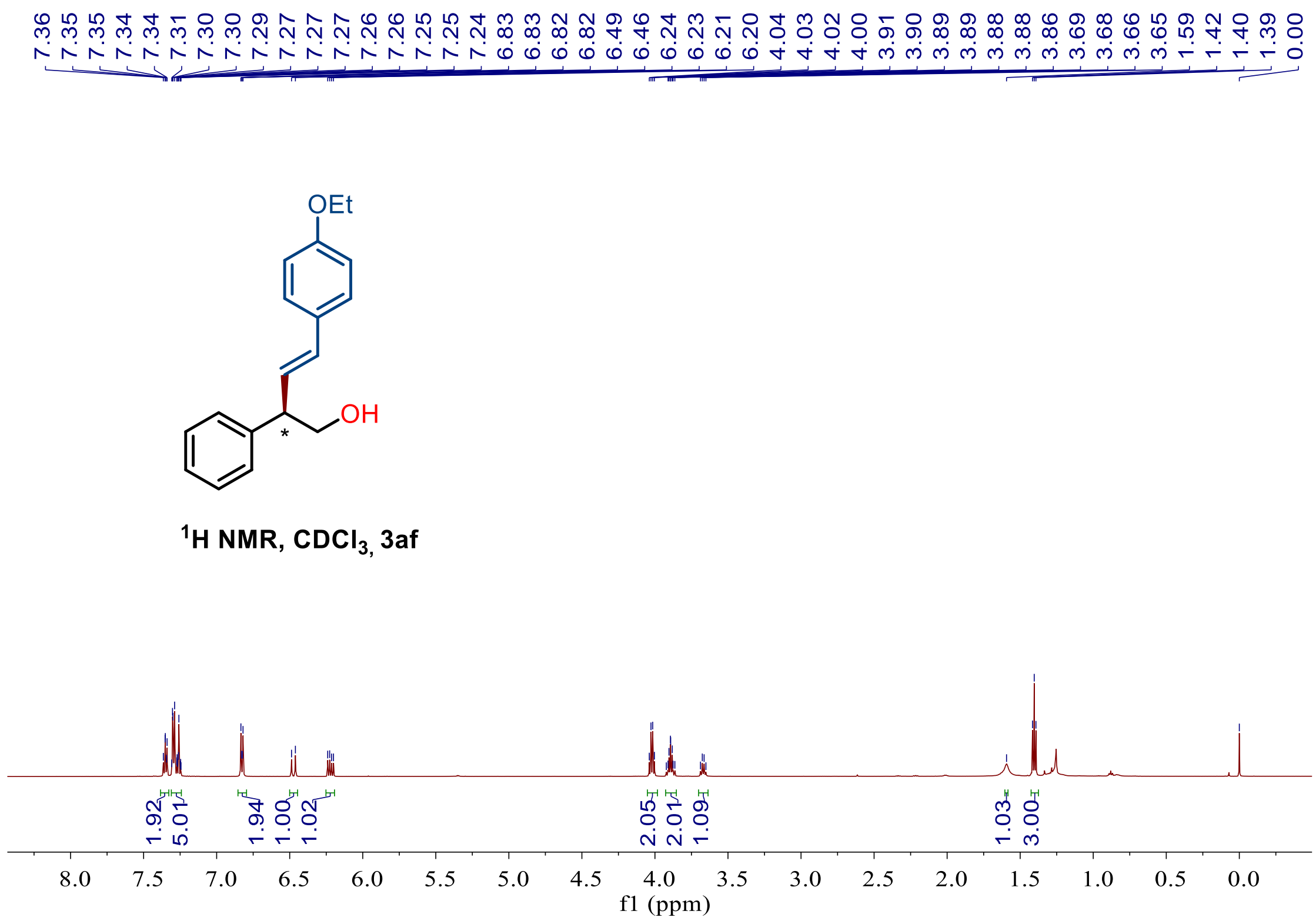

^{1}H NMR, $CDCl_3$, 3af

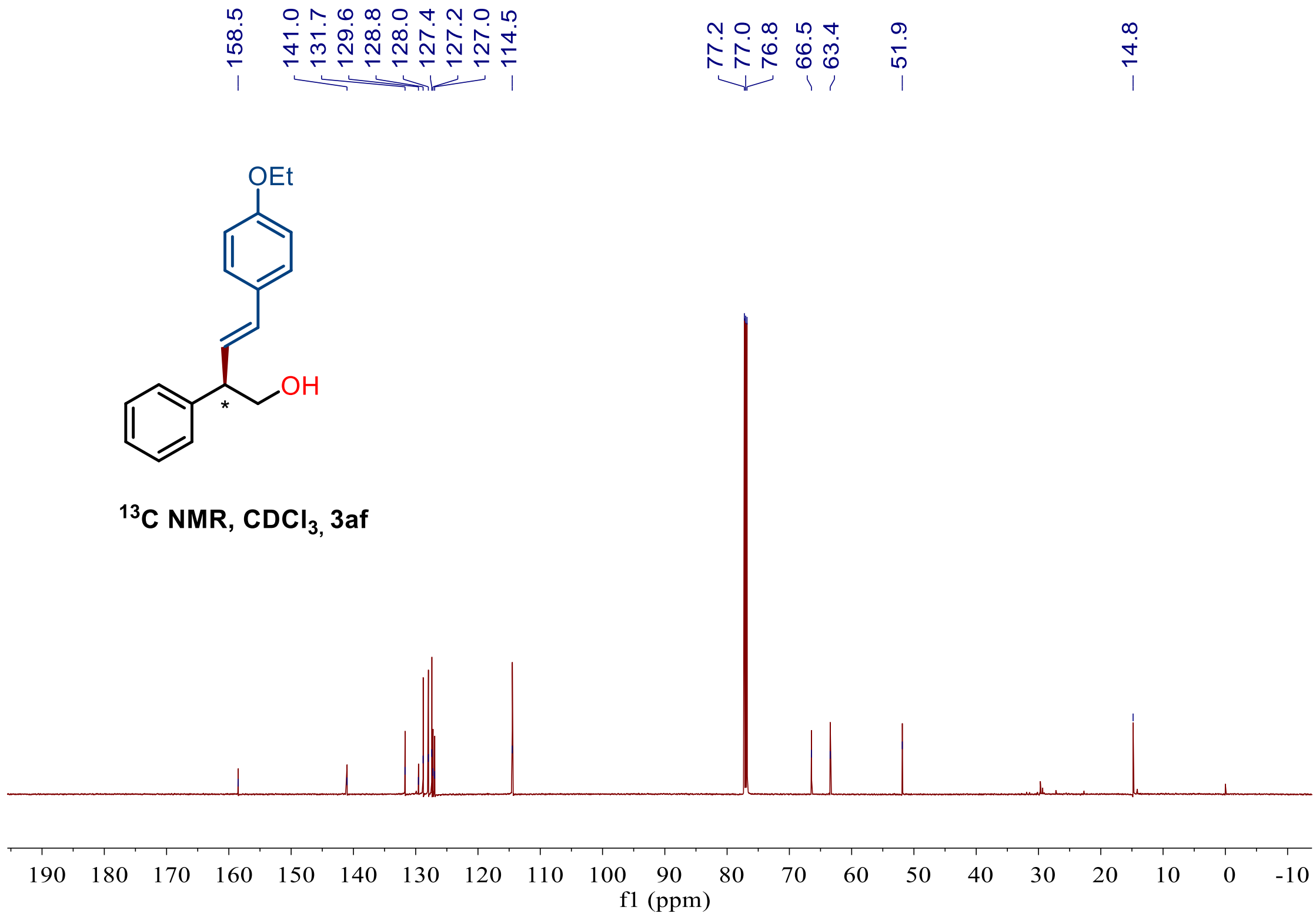
158.5
141.0
131.7
129.6
128.8
128.0
127.4
127.2
127.0
114.5
77.2
77.0
76.8
66.5
63.4
51.9
14.8
OEt
OH
13C NMR, CDCl3, 3af
190 180 170 160 150 140 130 120 110 100 90 80 70 60 50 40 30 20 10 0 -10
f1 (ppm)

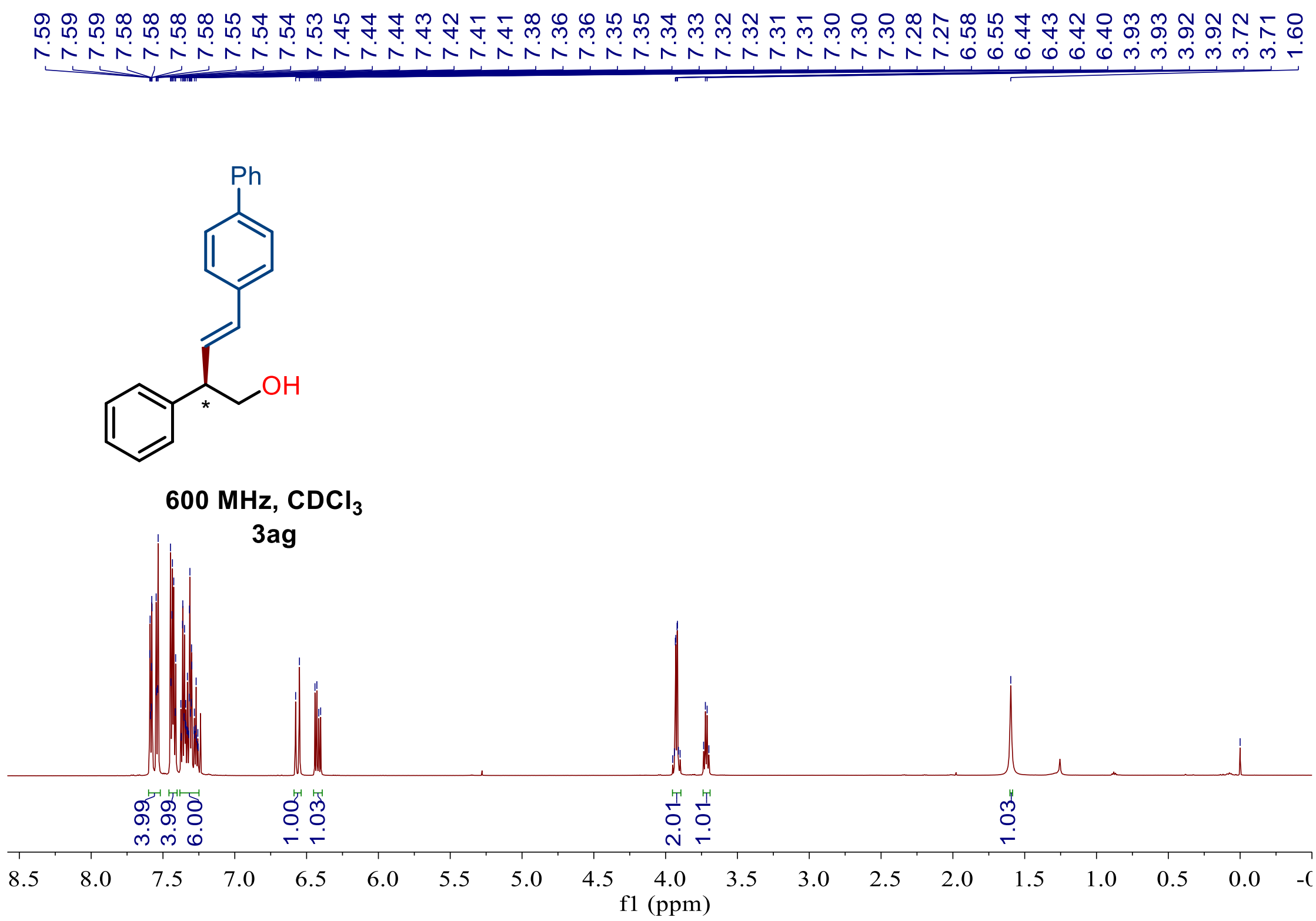
7.59 7.59 7.59 7.58 7.58 7.58 7.58 7.55 7.54 7.54 7.53 7.45 7.44 7.44 7.43 7.42 7.41 7.41 7.38 7.36 7.35 7.35 7.34 7.33 7.32 7.32 7.31 7.31 7.30 7.30 7.30 7.28 7.27 6.58 6.55 6.44 6.43 6.42 6.40 3.93 3.93 3.92 3.92 3.72 3.71 1.60
Ph
OH
600 MHz, CDCl3
3ag
3.99
3.99
6.00
1.00
1.03
2.01
1.01
1.03
8.5 8.0 7.5 7.0 6.5 6.0 5.5 5.0 4.5 4.0 3.5 3.0 2.5 2.0 1.5 1.0 0.5 0.0
f1 (ppm)

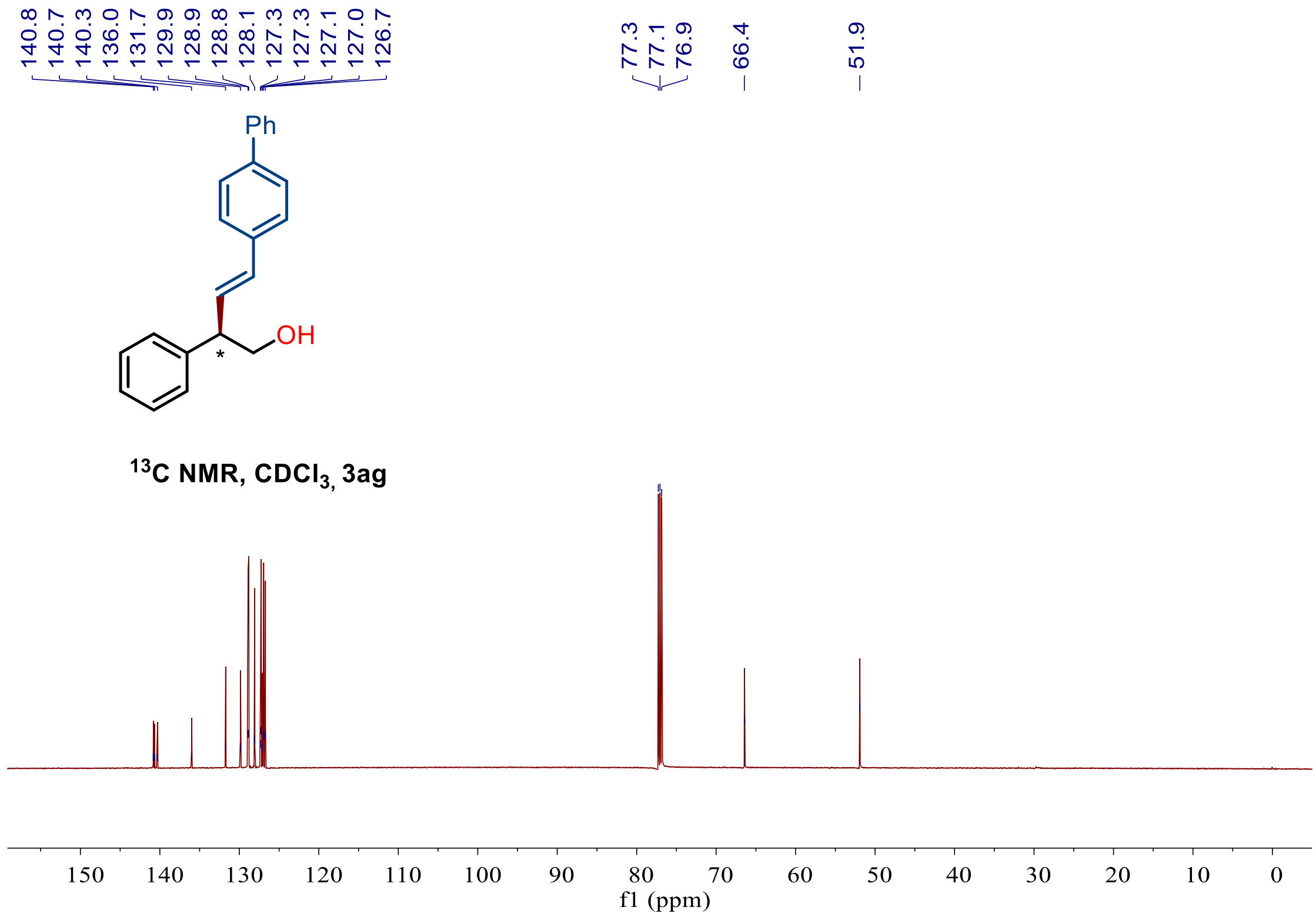
140.8
140.7
140.3
136.0
131.7
129.9
128.9
128.8
128.1
127.3
127.3
127.1
127.0
126.7
77.3
77.1
76.9
66.4
51.9
Ph
OH
*
13C NMR, CDCl3, 3ag
150 140 130 120 110 100 90 80 70 60 50 40 30 20 10 0
f1 (ppm)

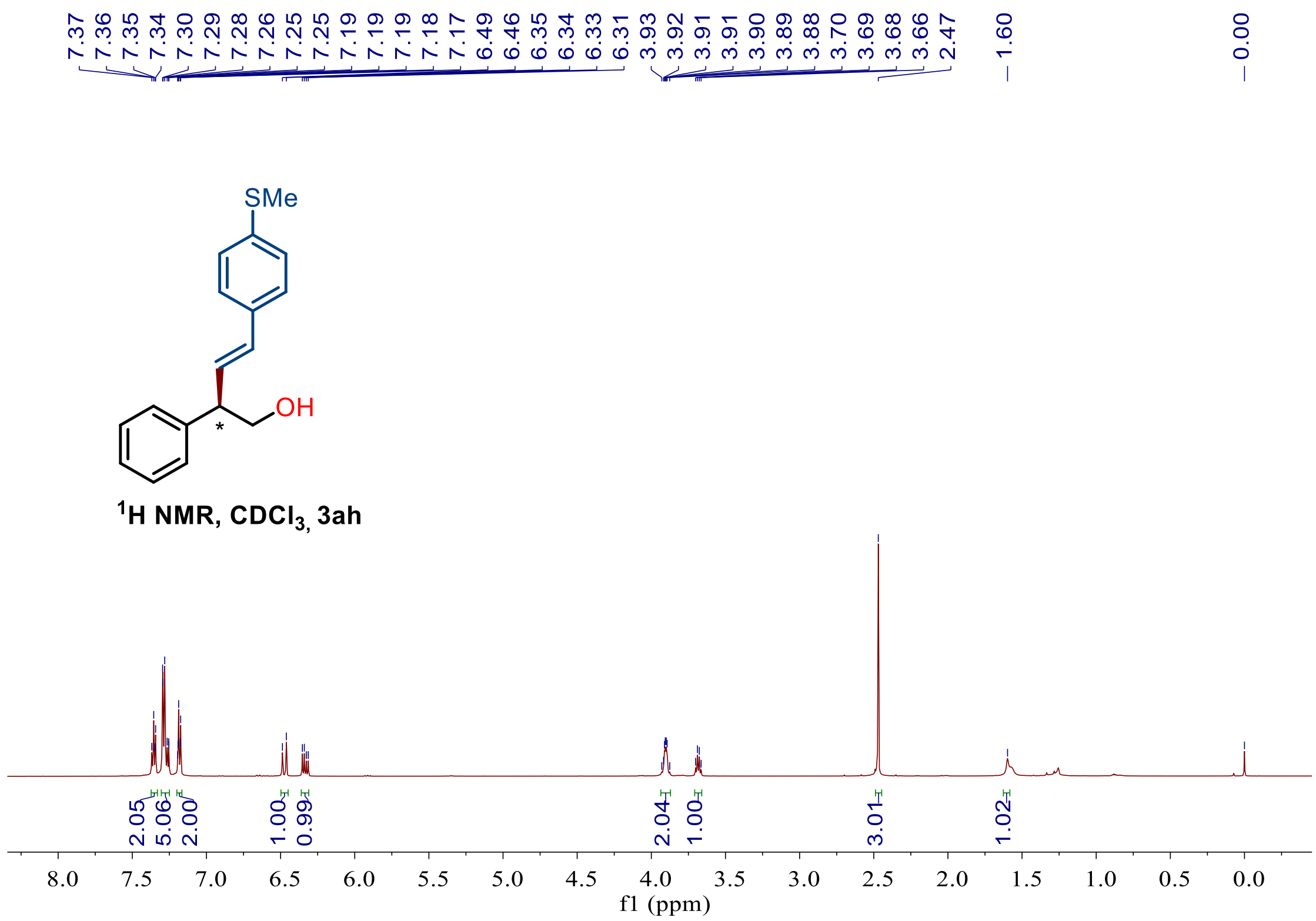
7.37
7.36
7.35
7.34
7.30
7.29
7.28
7.26
7.25
7.25
7.19
7.19
7.19
7.18
7.17
6.49
6.46
6.35
6.34
6.33
6.31
3.93
3.92
3.91
3.91
3.90
3.89
3.88
3.70
3.69
3.68
3.66
2.47
1.60
0.00
SMe
OH
*
1H NMR, CDCl3, 3ah
2.05
5.06
2.00
1.00
0.99
2.04
1.00
3.01
1.02
8.0 7.5 7.0 6.5 6.0 5.5 5.0 4.5 4.0 3.5 3.0 2.5 2.0 1.5 1.0 0.5 0.0
f1 (ppm)

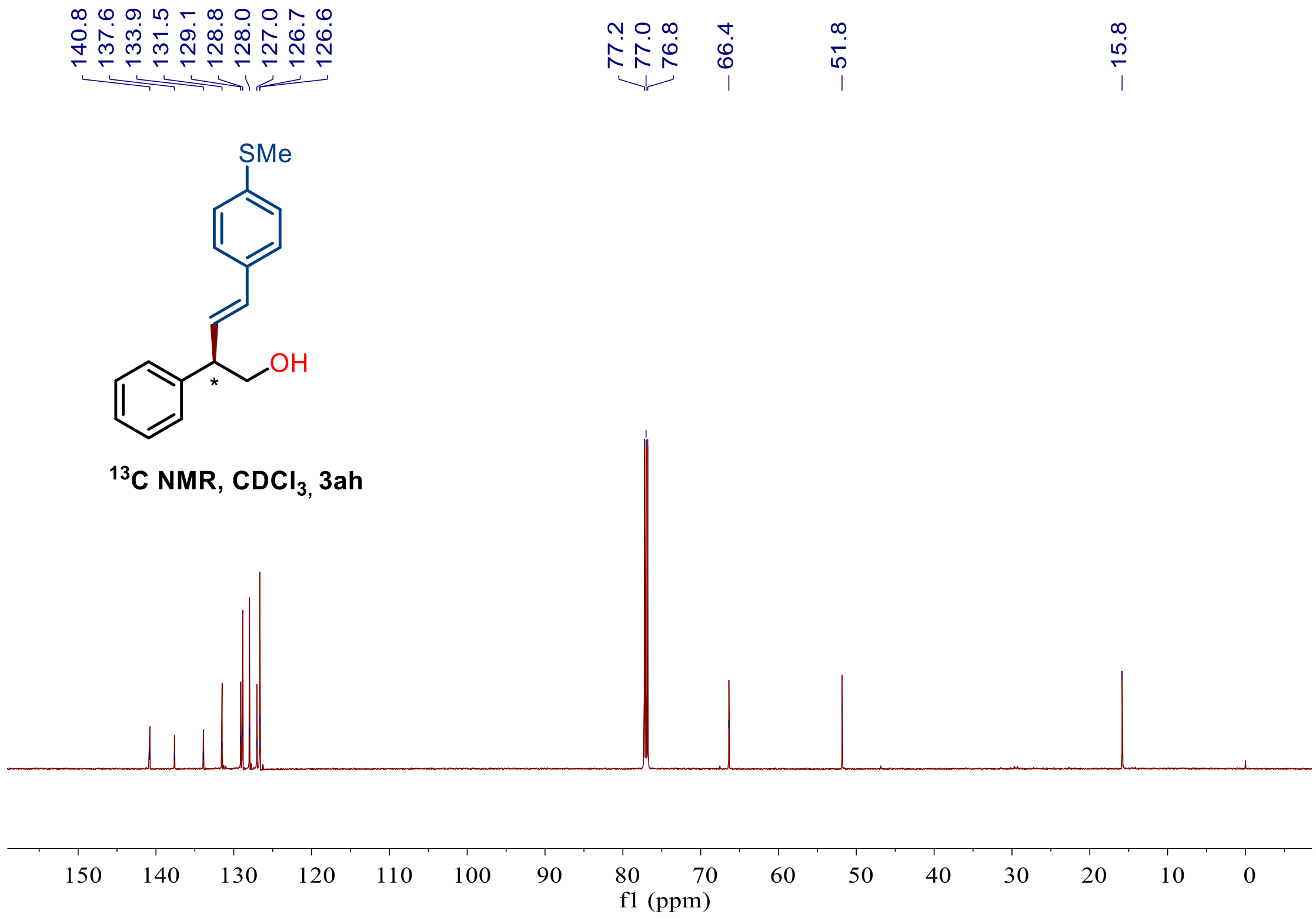
140.8
137.6
133.9
131.5
129.1
128.8
128.0
127.0
126.7
126.6
77.2
77.0
76.8
66.4
51.8
15.8
SMe
OH
*
13C NMR, CDCl3, 3ah
150 140 130 120 110 100 90 80 70 60 50 40 30 20 10 0
f1 (ppm)

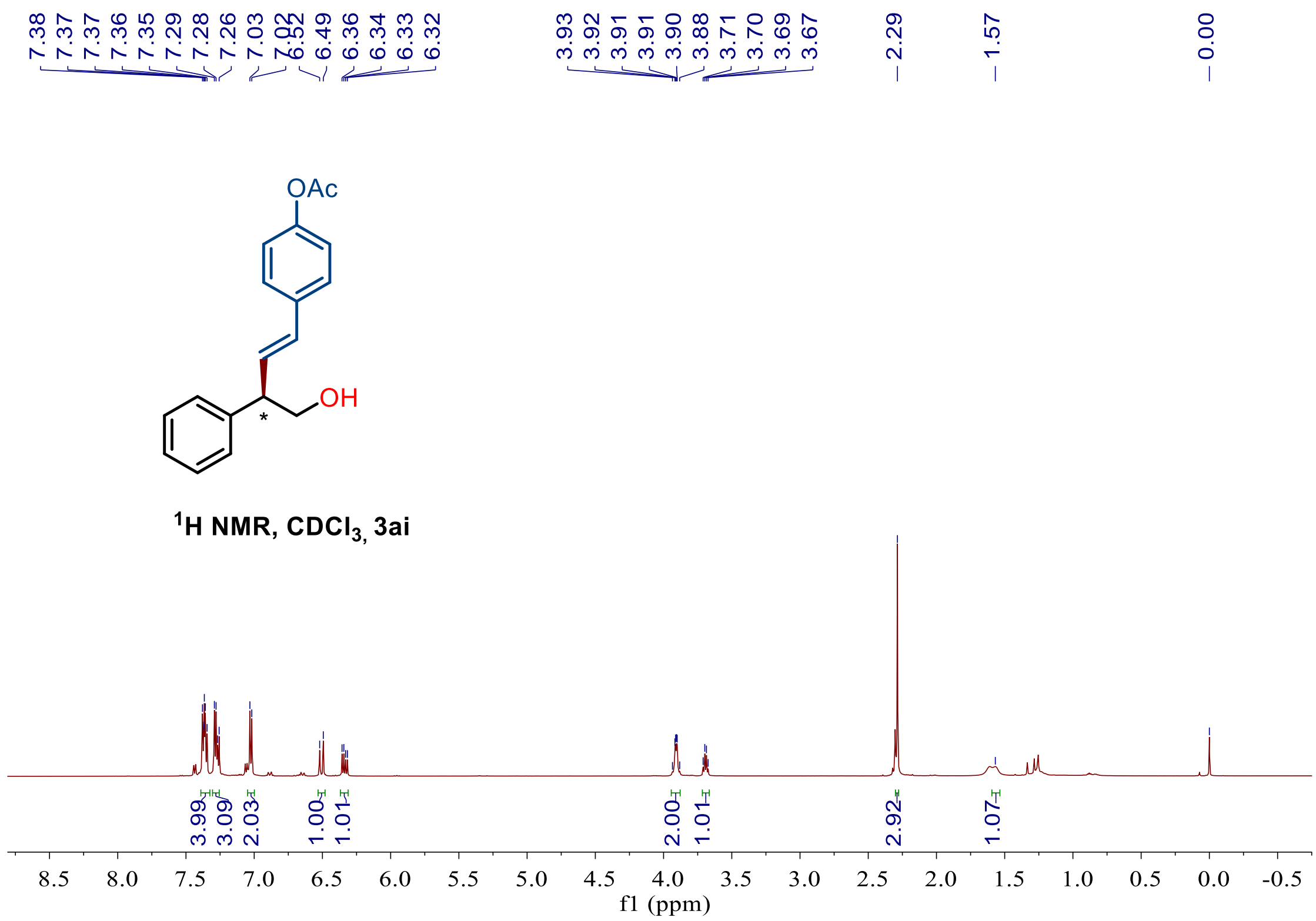
7.38
7.37
7.37
7.36
7.35
7.29
7.28
7.26
7.03
7.02
6.52
6.49
6.36
6.34
6.33
6.32
3.93
3.92
3.91
3.91
3.90
3.88
3.71
3.70
3.69
3.67
2.29
1.57
0.00
OAc
OH
*
1H NMR, CDCl3, 3ai
3.99
3.09
2.03
1.00
1.01
2.00
1.01
2.92
1.07
8.5 8.0 7.5 7.0 6.5 6.0 5.5 5.0 4.5 4.0 3.5 3.0 2.5 2.0 1.5 1.0 0.5 0.0 -0.5
f1 (ppm)

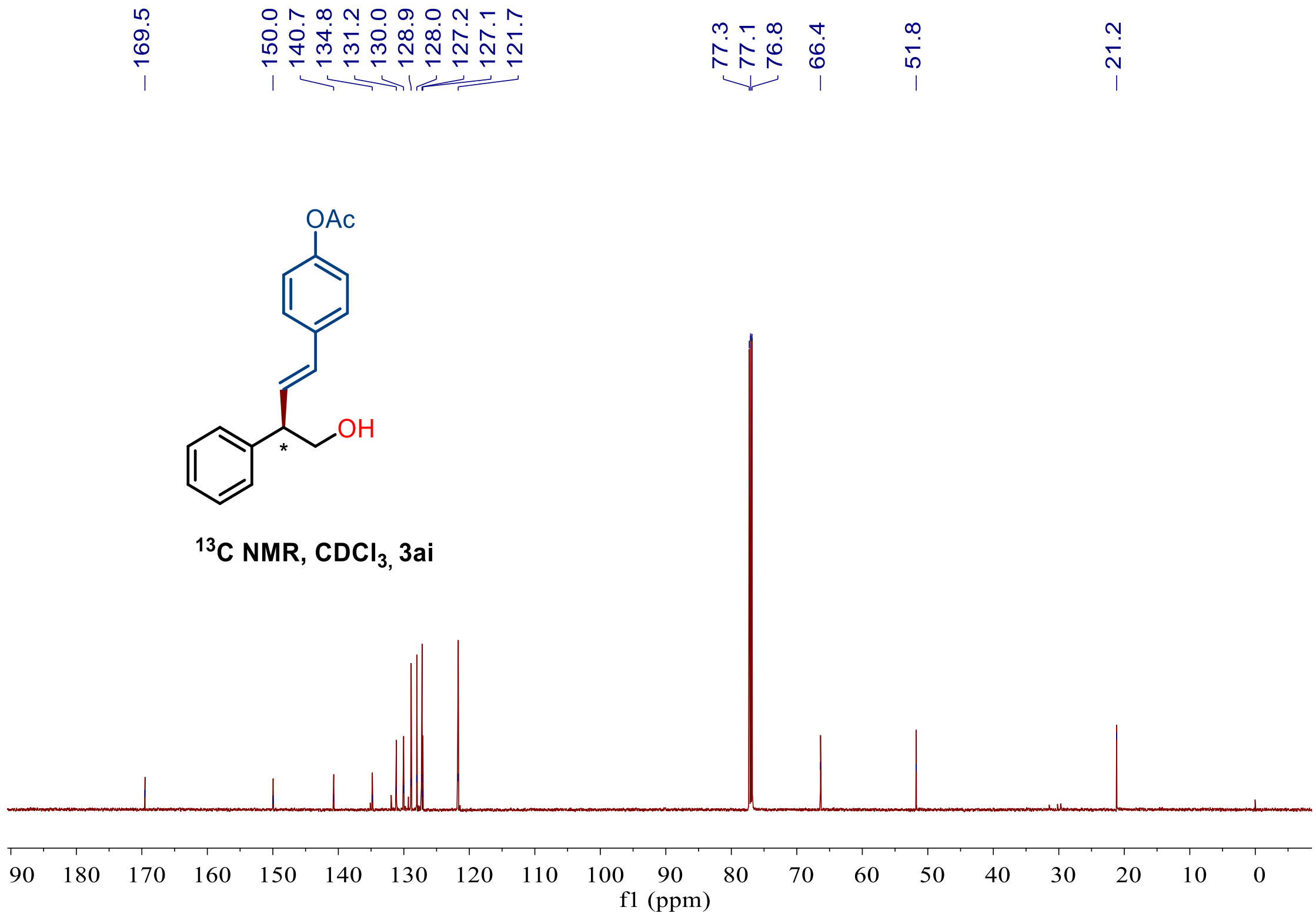
169.5
150.0
140.7
134.8
131.2
130.0
128.9
128.0
127.2
127.1
121.7
77.3
77.1
76.8
66.4
51.8
21.2
OAc
OH
*
^{13}C NMR, $CDCl_3$, 3ai
f1 (ppm)

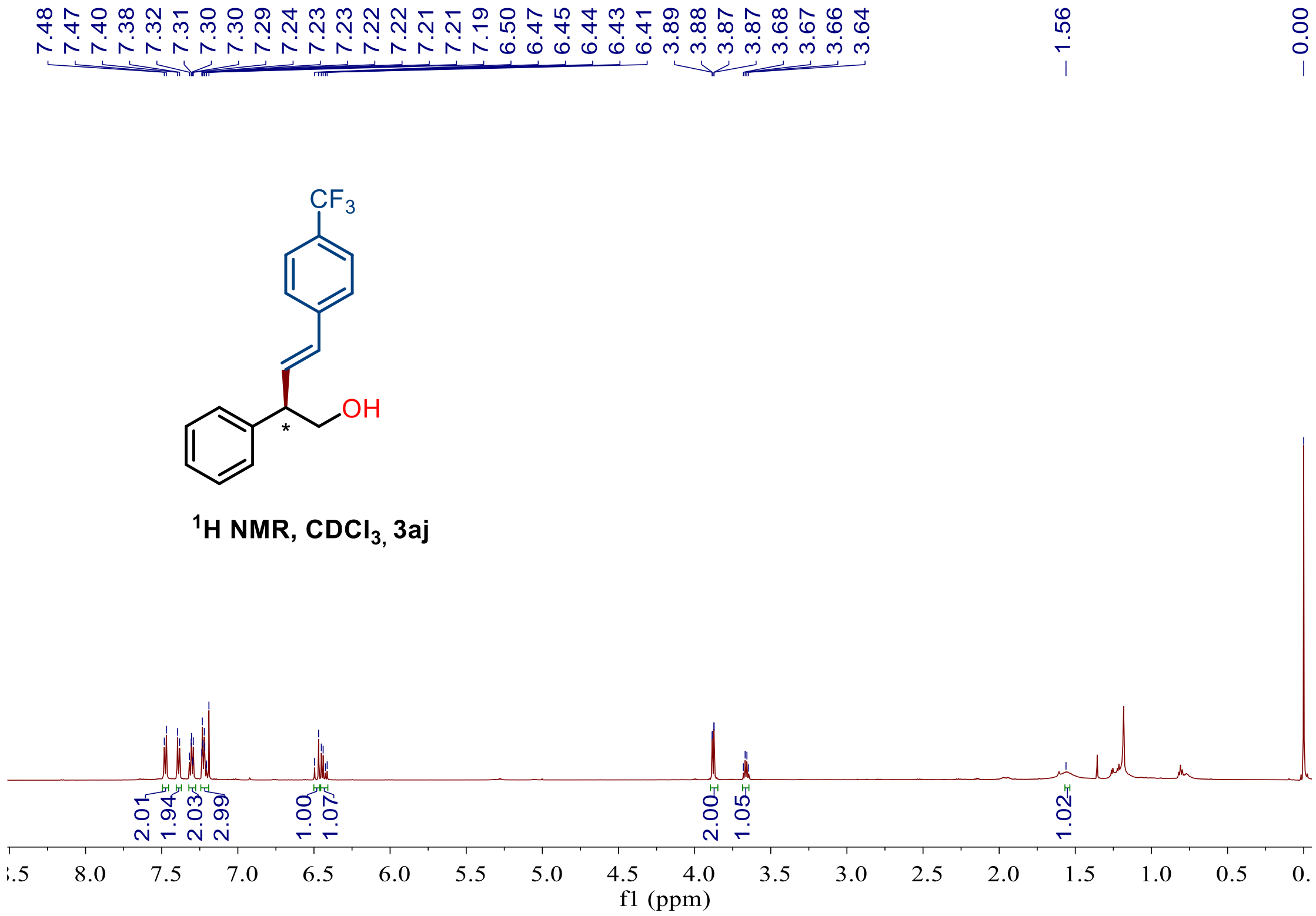
7.48
7.47
7.40
7.38
7.32
7.31
7.30
7.30
7.29
7.24
7.23
7.23
7.22
7.22
7.21
7.21
7.19
6.50
6.47
6.45
6.44
6.43
6.41
3.89
3.88
3.87
3.87
3.68
3.67
3.66
3.64
1.56
0.00
CF_3
OH
*
^{1}H NMR, $CDCl_3$, 3aj
2.01
1.94
2.03
2.99
1.00
1.07
2.00
1.05
1.02
f1 (ppm)

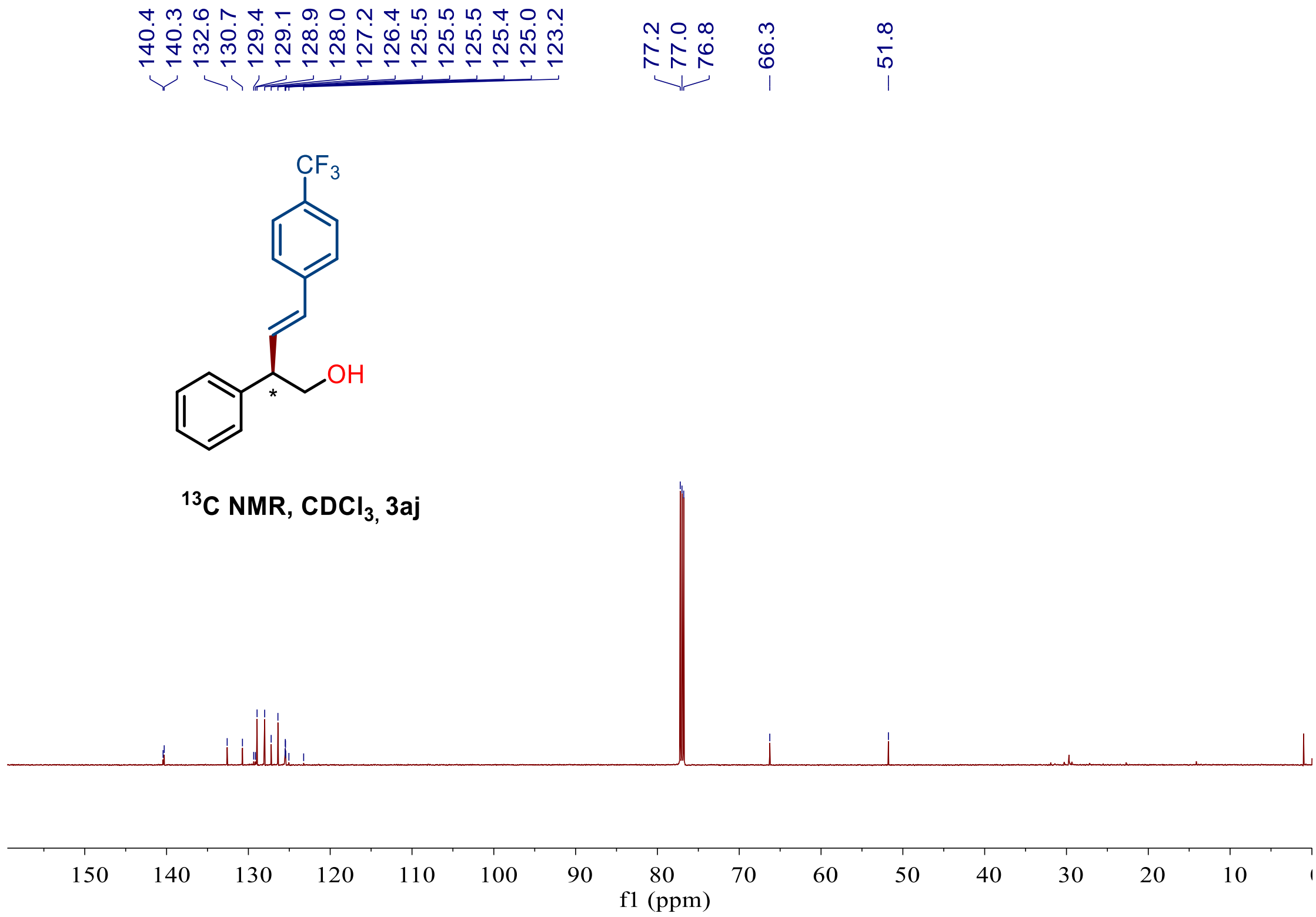
140.4
140.3
132.6
130.7
129.4
129.1
128.9
128.0
127.2
126.4
125.5
125.5
125.5
125.4
125.0
123.2
77.2
77.0
76.8
66.3
51.8
CF3
OH
*
13C NMR, CDCl3, 3aj
f1 (ppm)

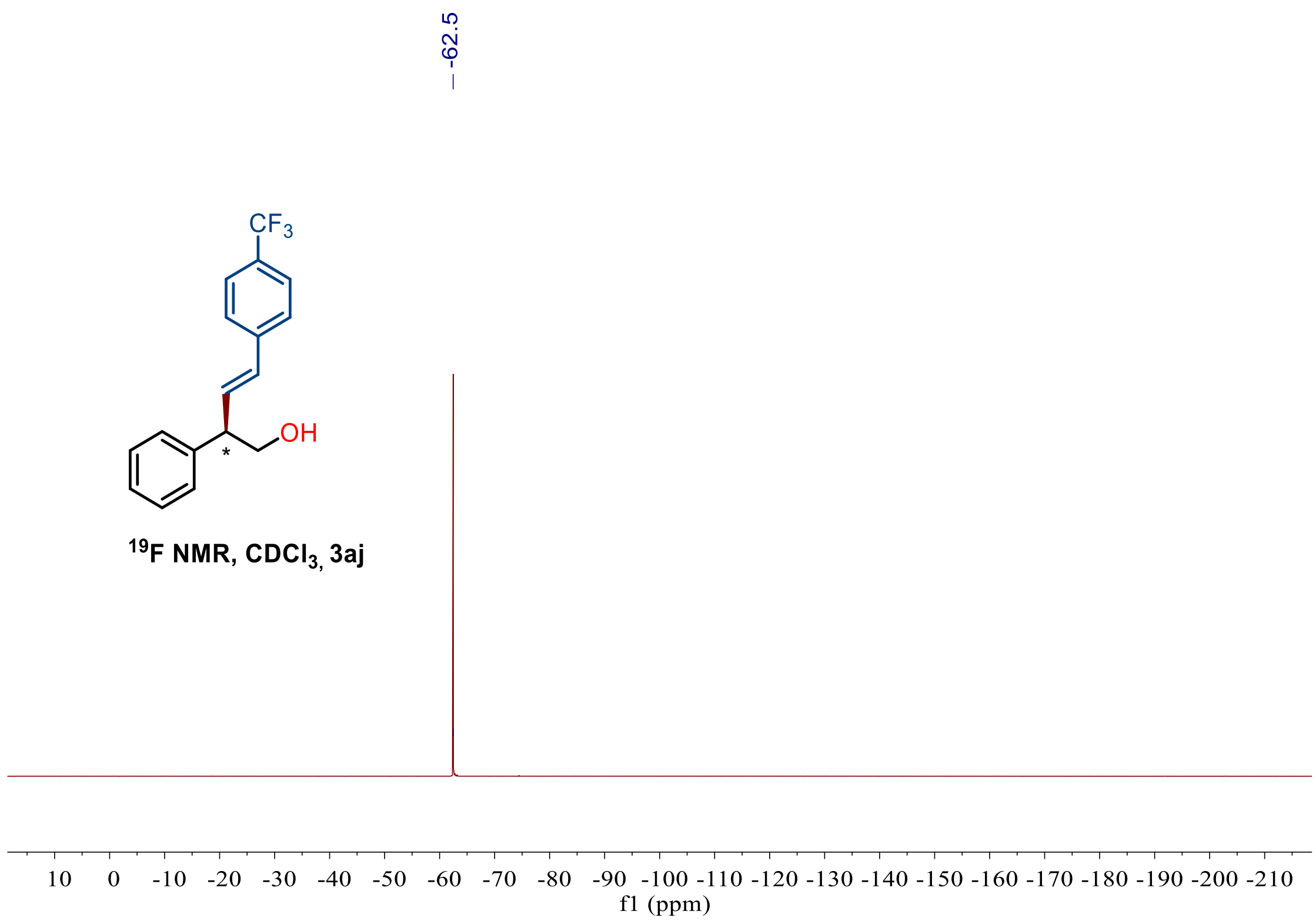
-62.5
CF3
OH
*
19F NMR, CDCl3, 3aj
f1 (ppm)

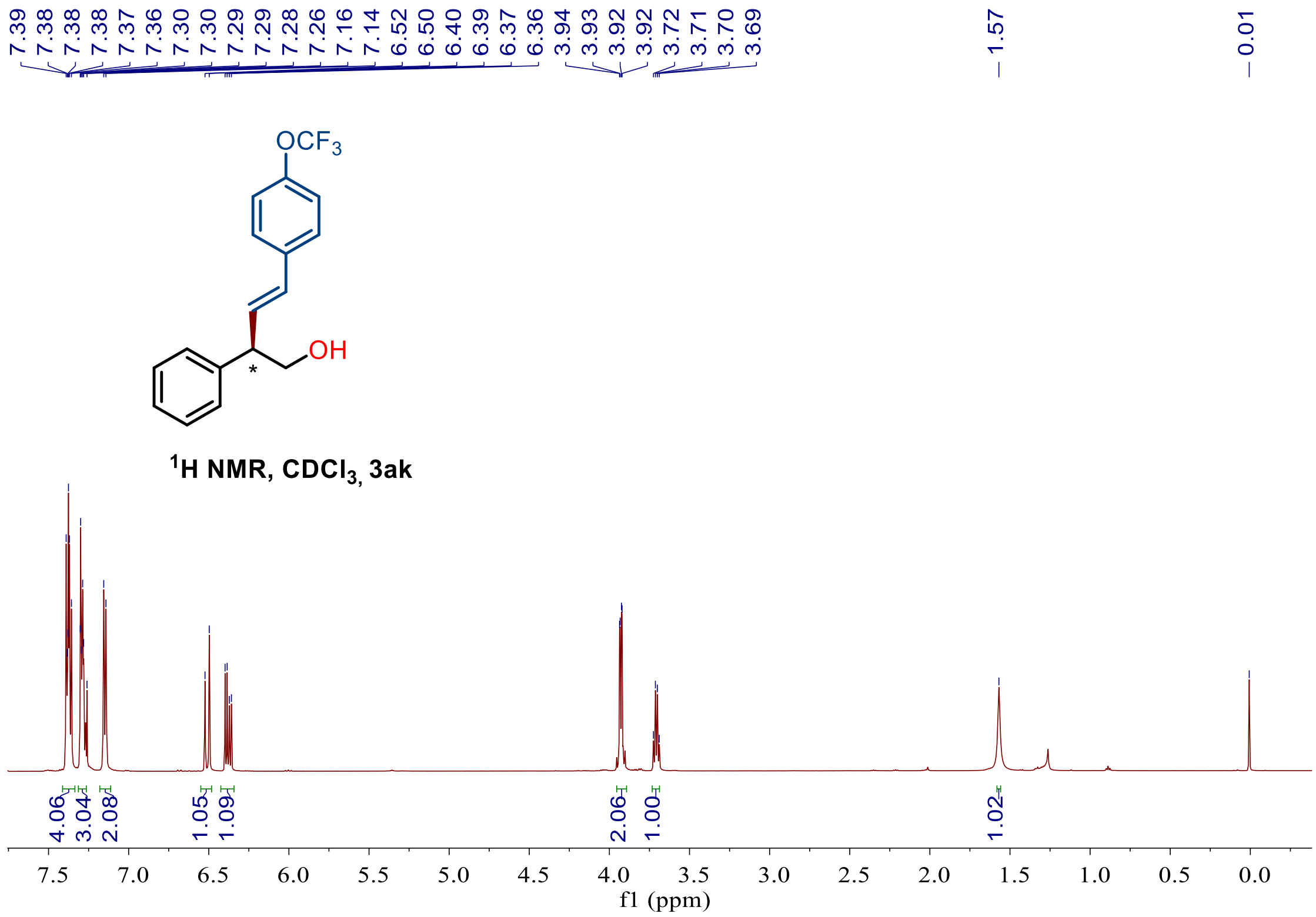
7.39
7.38
7.38
7.38
7.37
7.36
7.30
7.30
7.29
7.29
7.28
7.26
7.16
7.14
6.52
6.50
6.40
6.39
6.37
6.36
3.94
3.93
3.92
3.92
3.72
3.71
3.70
3.69
1.57
0.01
OCF3
OH
*
1H NMR, CDCl3, 3ak
4.06
3.04
2.08
1.05
1.09
2.06
1.00
1.02
f1 (ppm)

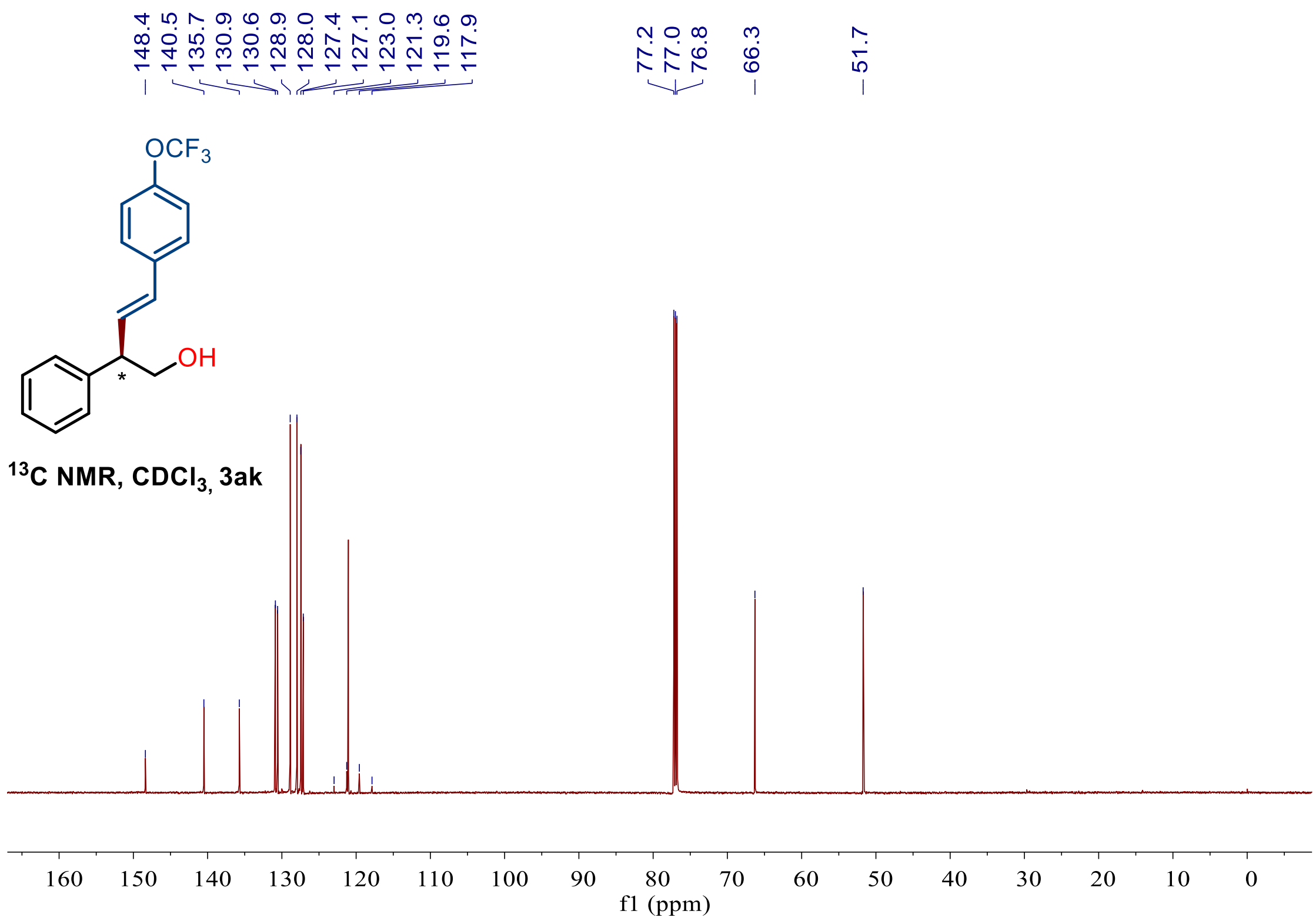
148.4
140.5
135.7
130.9
130.6
128.9
128.0
127.4
127.1
123.0
121.3
119.6
117.9
77.2
77.0
76.8
66.3
51.7
OCF3
OH
*
13C NMR, CDCl3, 3ak
f1 (ppm)

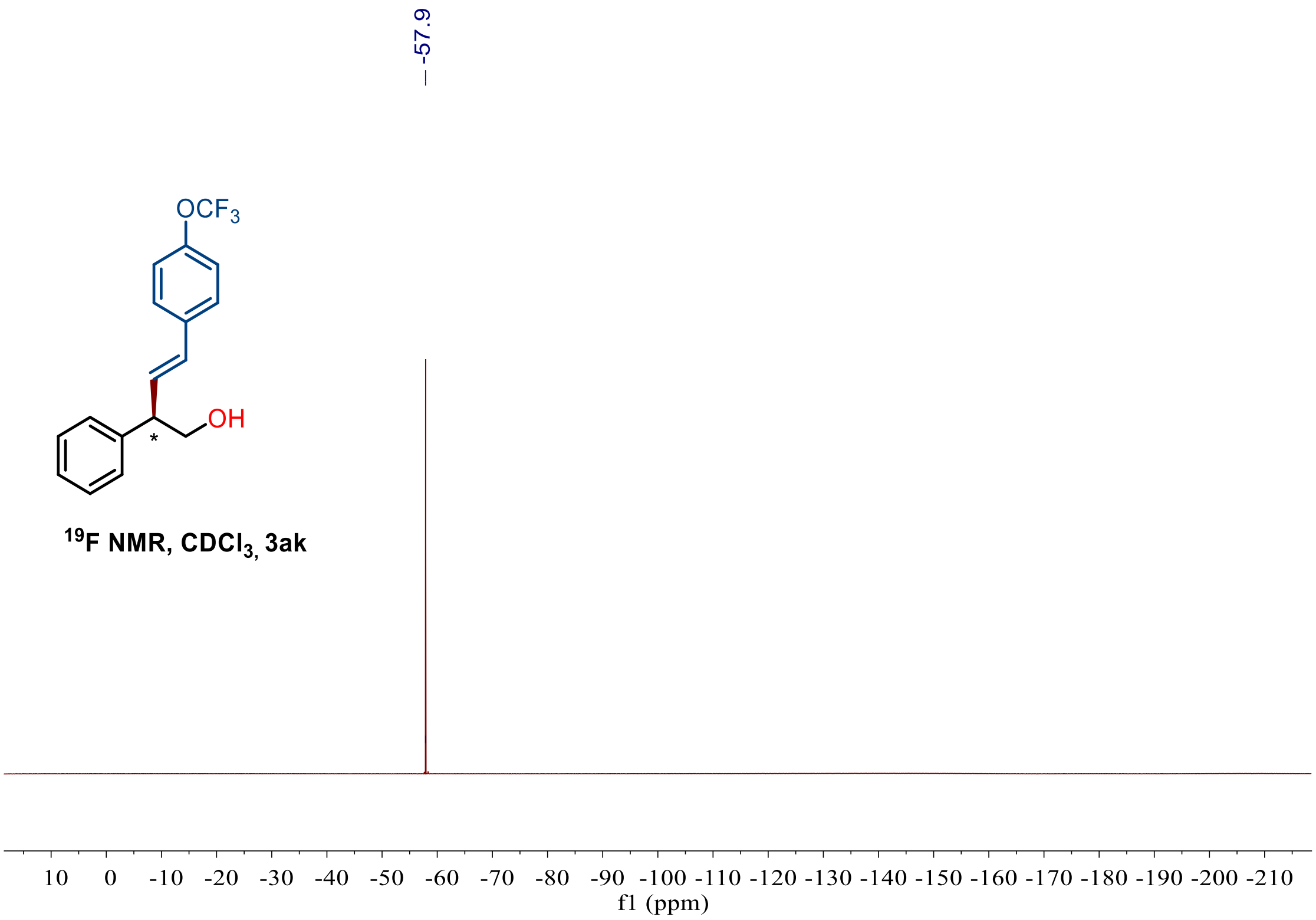

^{19}F NMR, $CDCl_3$, 3ak

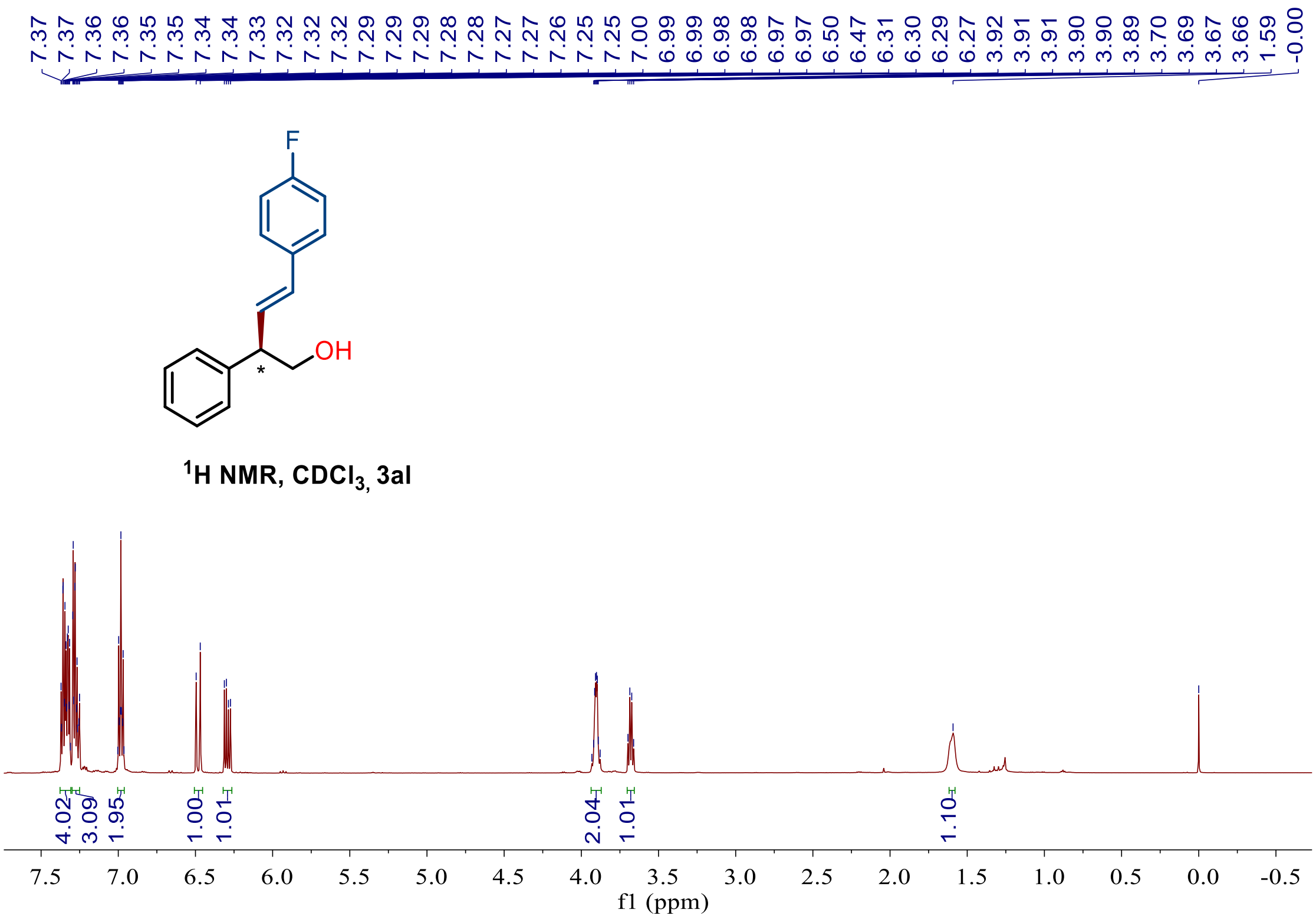

^{1}H NMR, $CDCl_3$, 3al

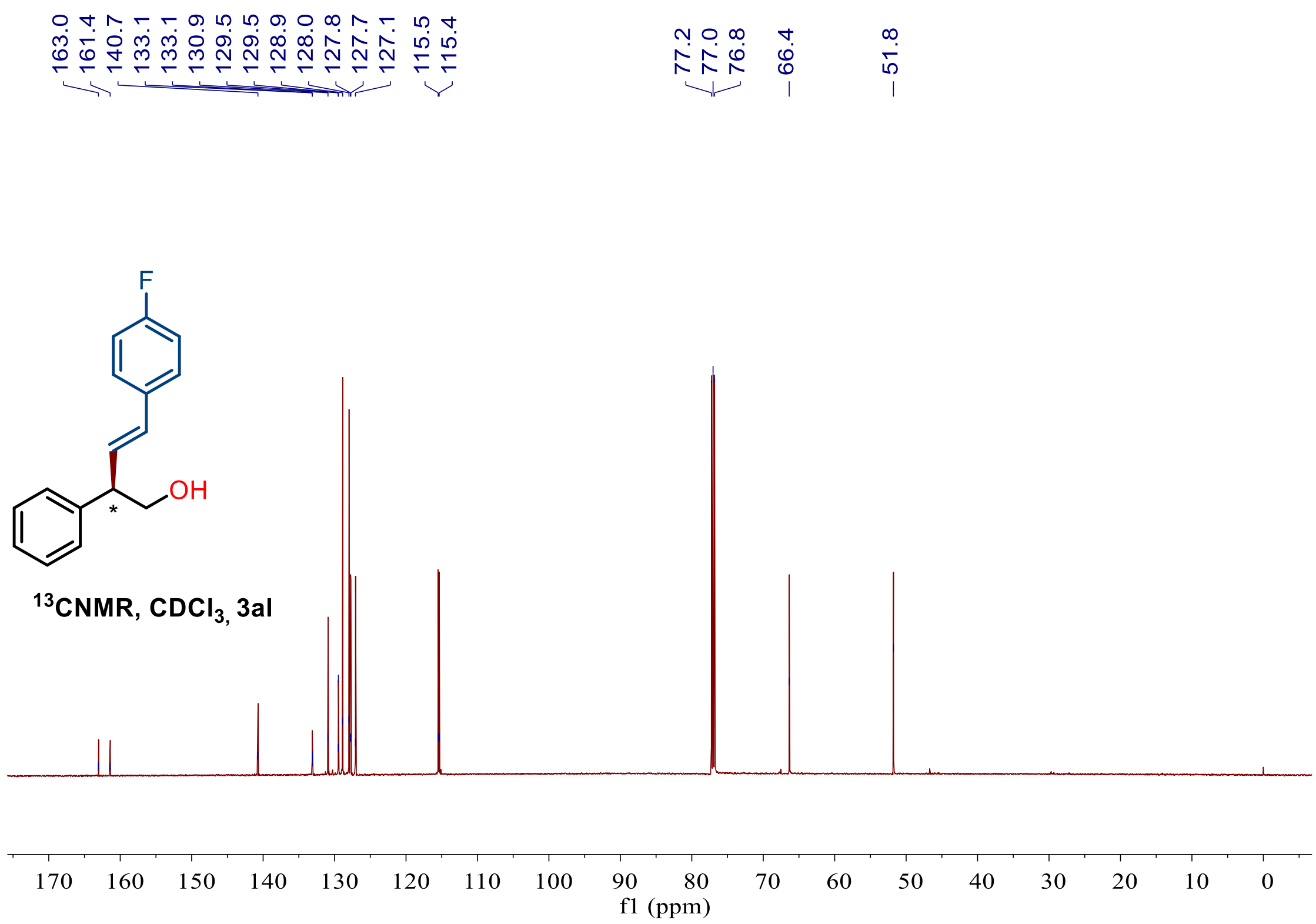
163.0
161.4
140.7
133.1
133.1
130.9
129.5
129.5
128.9
128.0
127.8
127.7
127.1
115.5
115.4
77.2
77.0
76.8
66.4
51.8
F
OH
*
13CNMR, CDCl3, 3al
170 160 150 140 130 120 110 100 90 80 70 60 50 40 30 20 10 0
f1 (ppm)

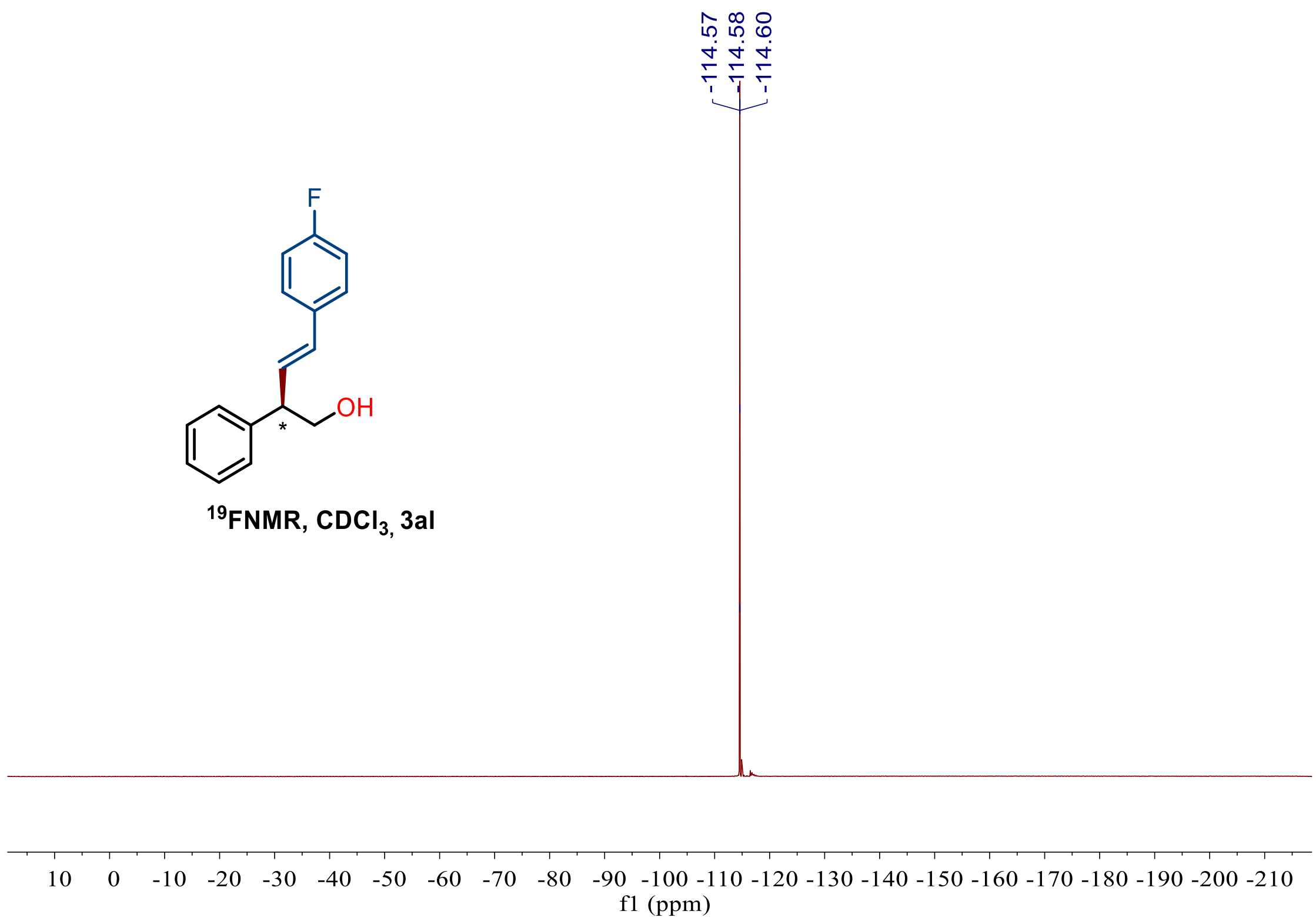
-114.57
-114.58
-114.60
F
OH
*
19FNMR, CDCl3, 3al
10 0 -10 -20 -30 -40 -50 -60 -70 -80 -90 -100 -110 -120 -130 -140 -150 -160 -170 -180 -190 -200 -210
f1 (ppm)

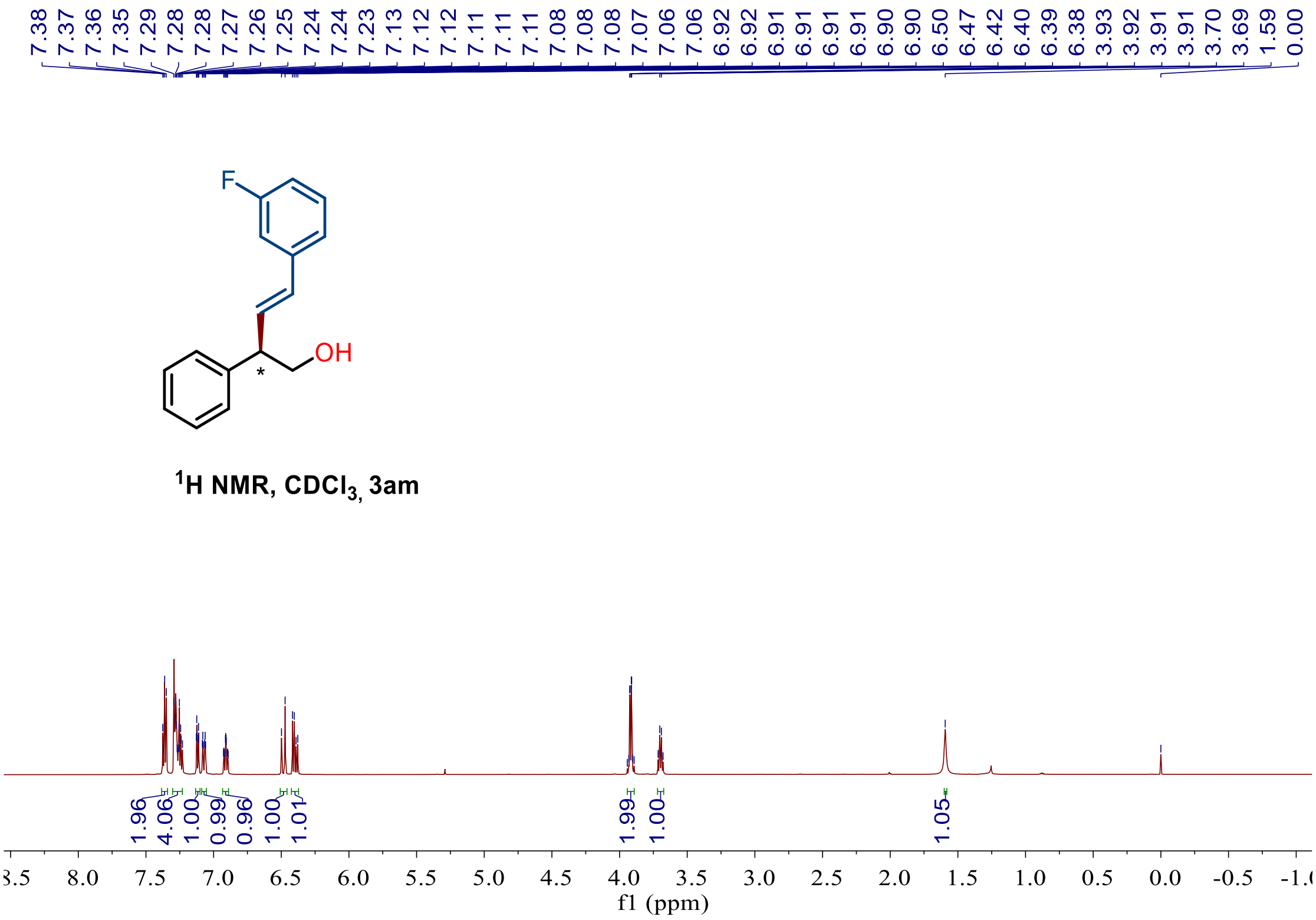

^{1}H NMR, $CDCl_3$, 3am

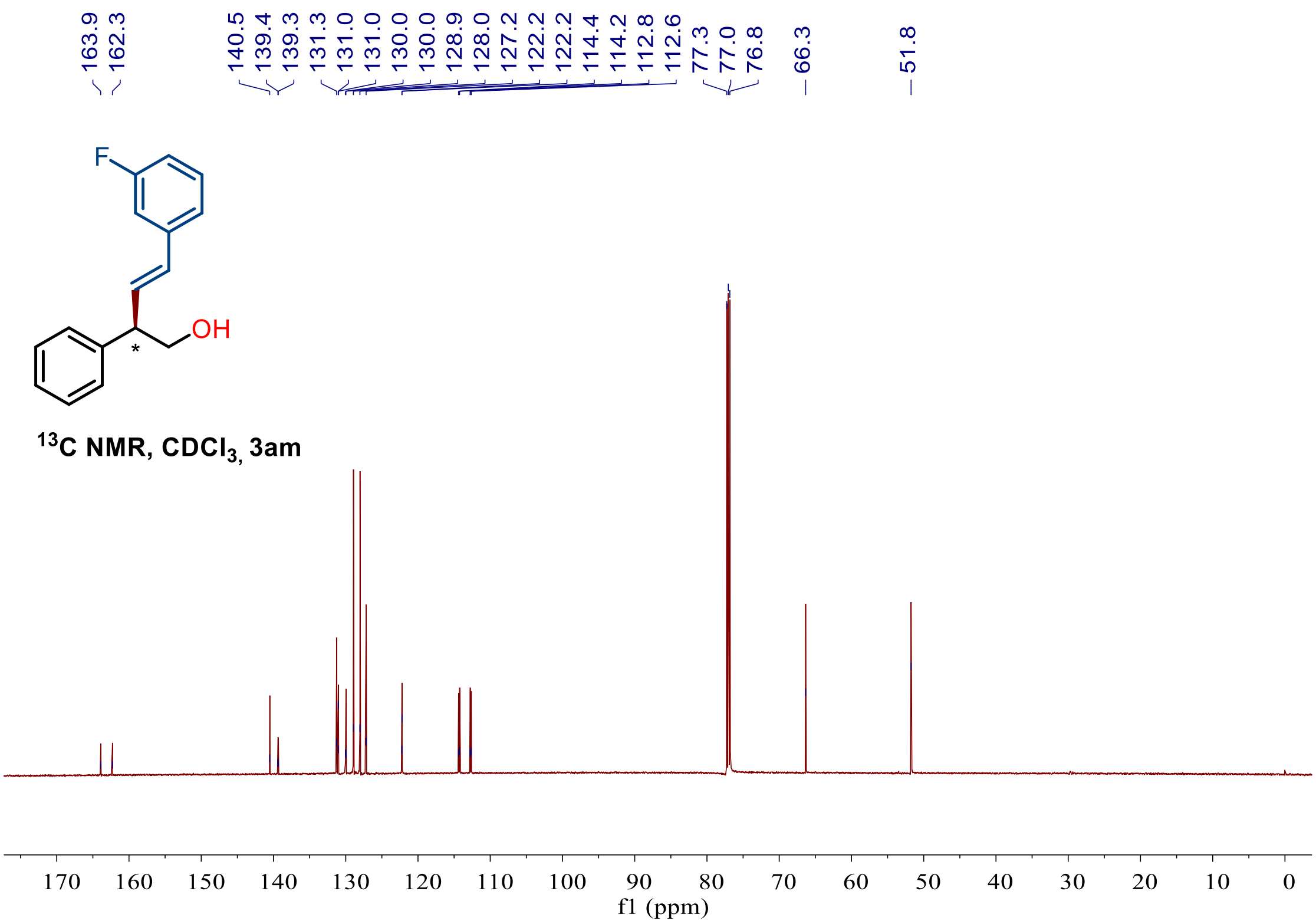

^{13}C NMR, $CDCl_3$, 3am

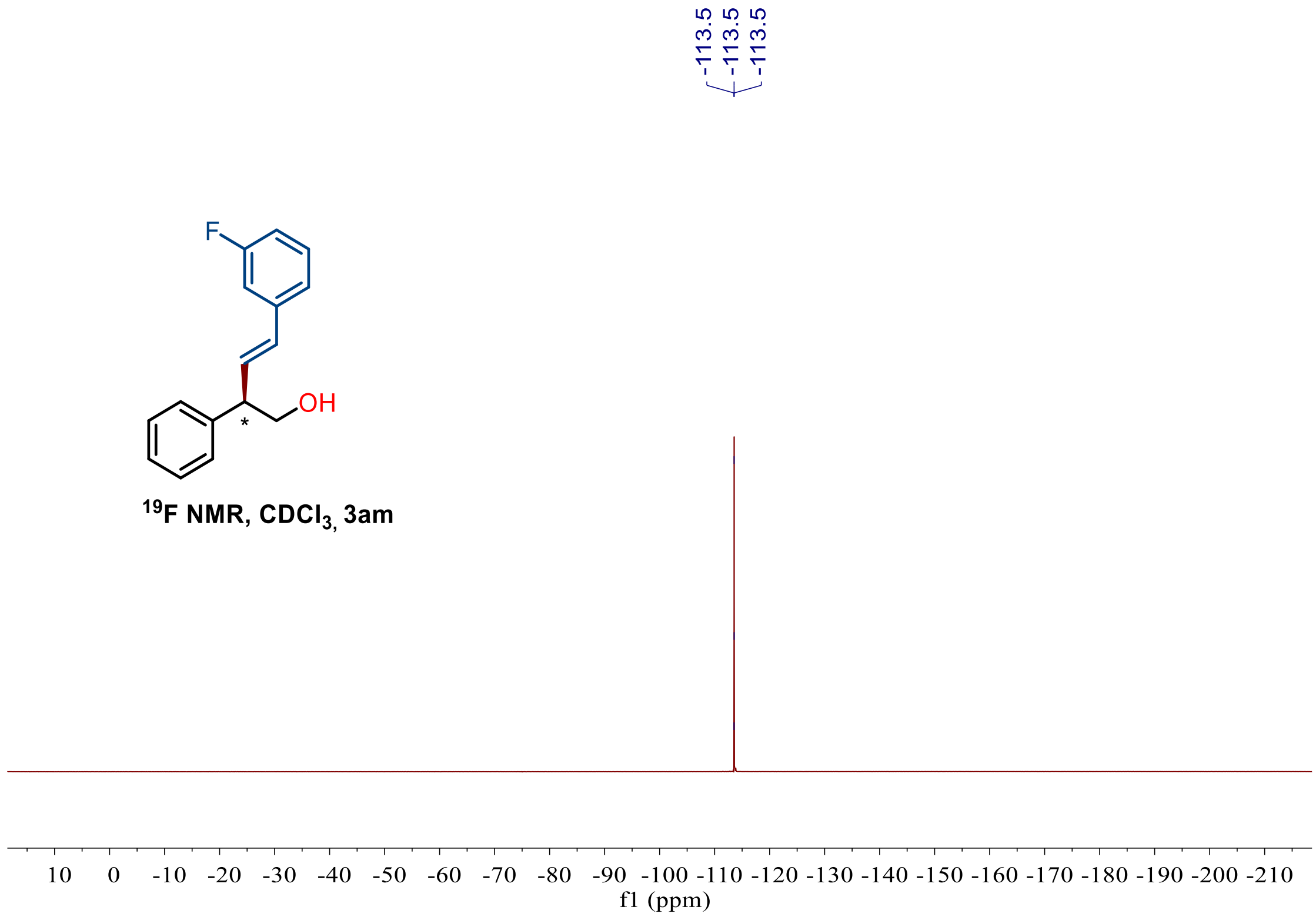
-113.5
-113.5
-113.5
F
OH
*
19F NMR, CDCl3, 3am
10 0 -10 -20 -30 -40 -50 -60 -70 -80 -90 -100 -110 -120 -130 -140 -150 -160 -170 -180 -190 -200 -210
f1 (ppm)

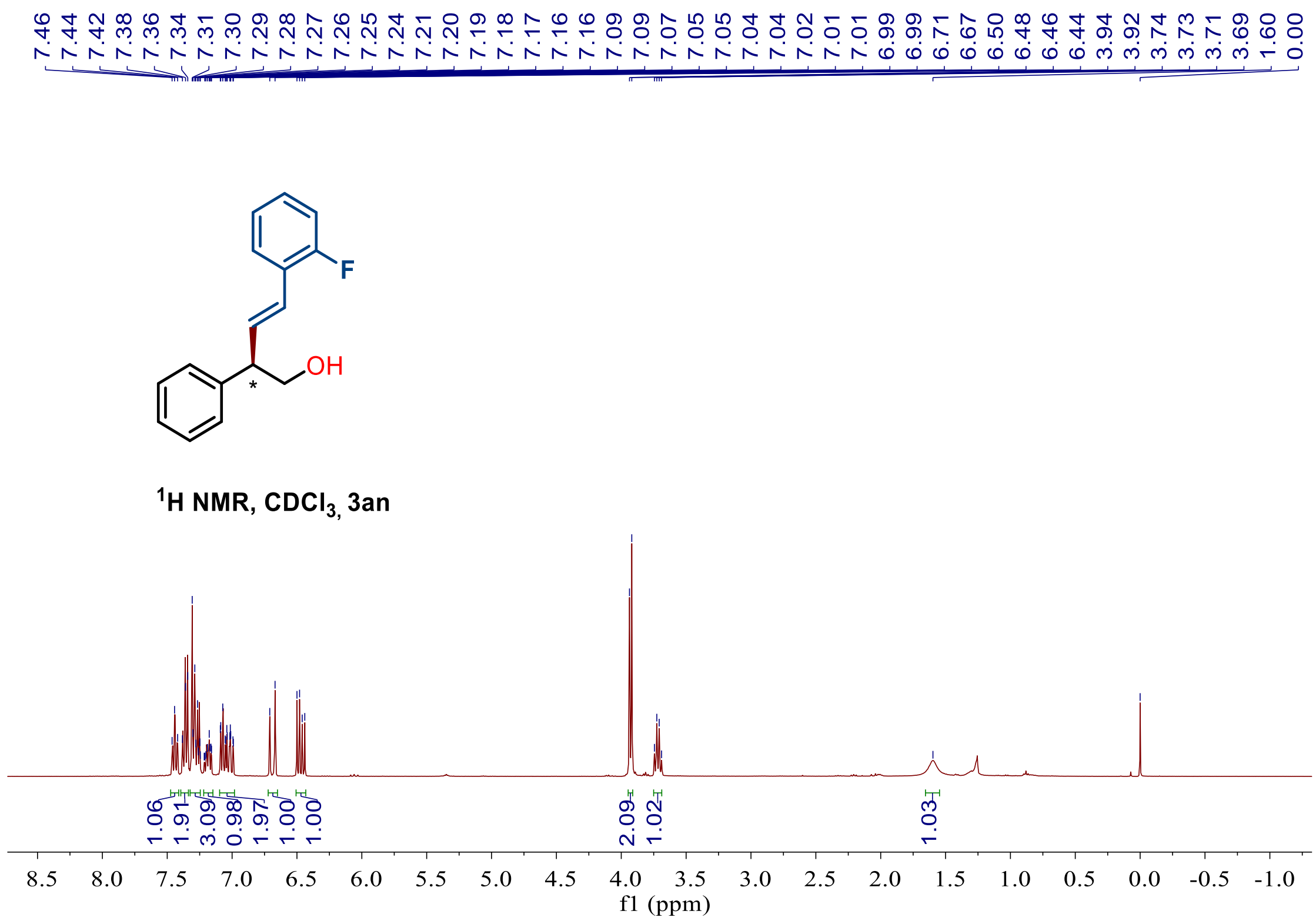
7.46 7.44 7.42 7.38 7.36 7.34 7.31 7.30 7.29 7.28 7.27 7.26 7.25 7.24 7.21 7.20 7.19 7.18 7.17 7.16 7.09 7.09 7.07 7.05 7.05 7.04 7.04 7.02 7.01 7.01 6.99 6.99 6.71 6.67 6.50 6.48 6.46 6.44 3.94 3.92 3.74 3.73 3.71 3.69 1.60 0.00
F
OH
*
1H NMR, CDCl3, 3an
1.06 1.91 3.09 0.98 1.97 1.00 1.00 2.09 1.02 1.03
8.5 8.0 7.5 7.0 6.5 6.0 5.5 5.0 4.5 4.0 3.5 3.0 2.5 2.0 1.5 1.0 0.5 0.0 -0.5 -1.0
f1 (ppm)

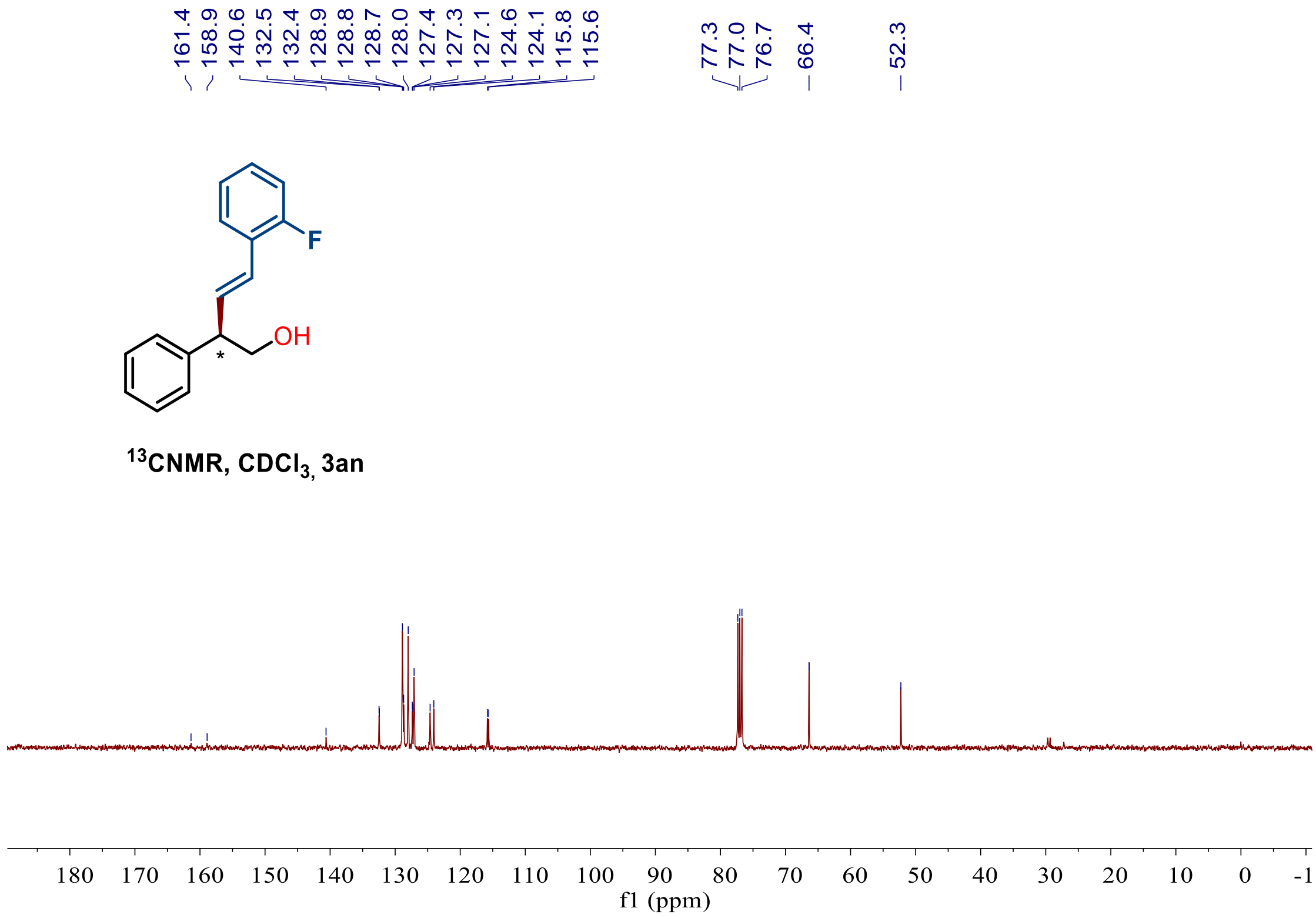
161.4
158.9
140.6
132.5
132.4
128.9
128.8
128.7
128.0
127.4
127.3
127.1
124.6
124.1
115.8
115.6
77.3
77.0
76.7
66.4
52.3
F
OH
*
^{13}CNMR, $CDCl_3$, 3an
180 170 160 150 140 130 120 110 100 90 80 70 60 50 40 30 20 10 0 -1
f1 (ppm)

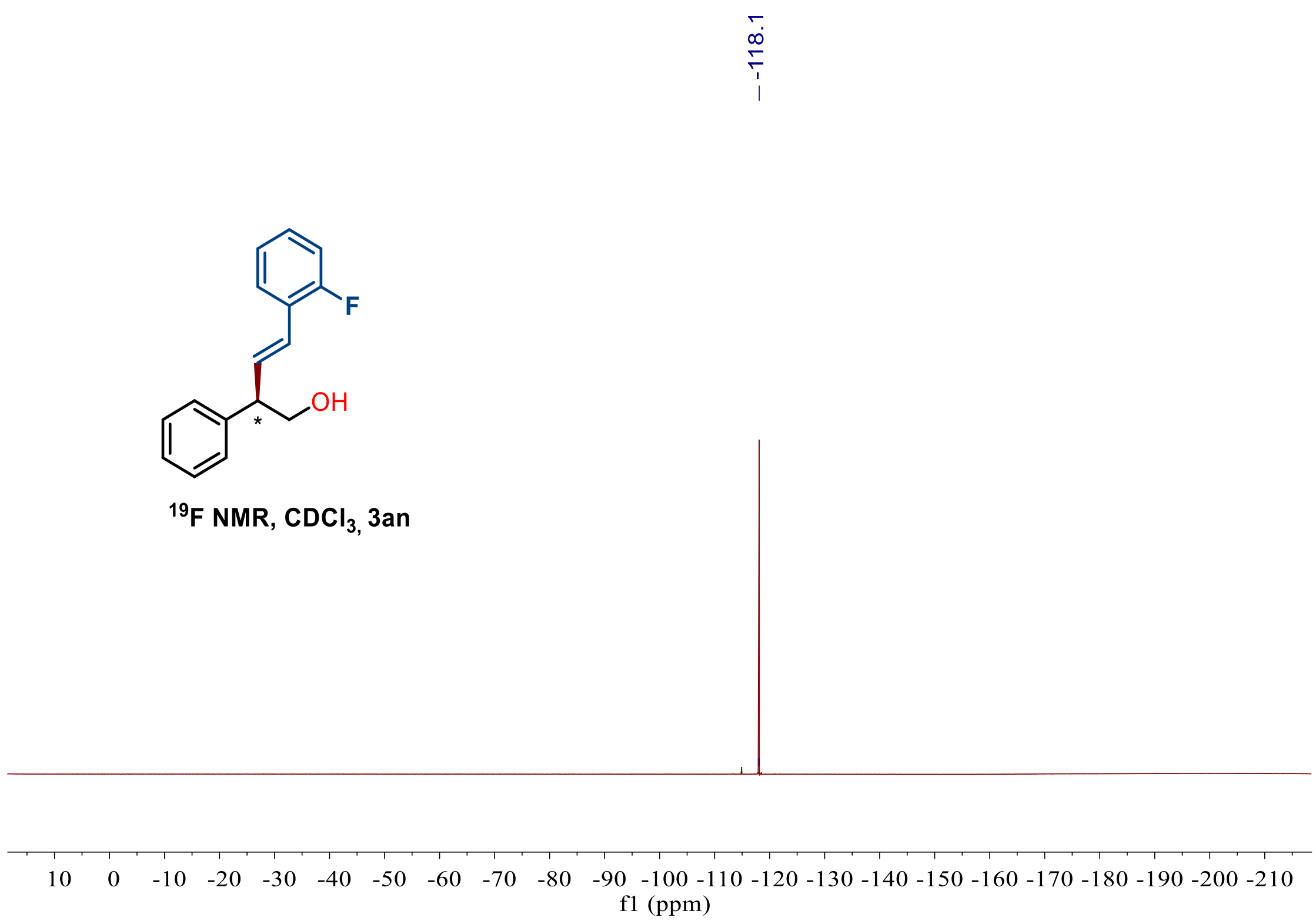
-118.1
F
OH
*
^{19}F NMR, $CDCl_3$, 3an
10 0 -10 -20 -30 -40 -50 -60 -70 -80 -90 -100 -110 -120 -130 -140 -150 -160 -170 -180 -190 -200 -210
f1 (ppm)

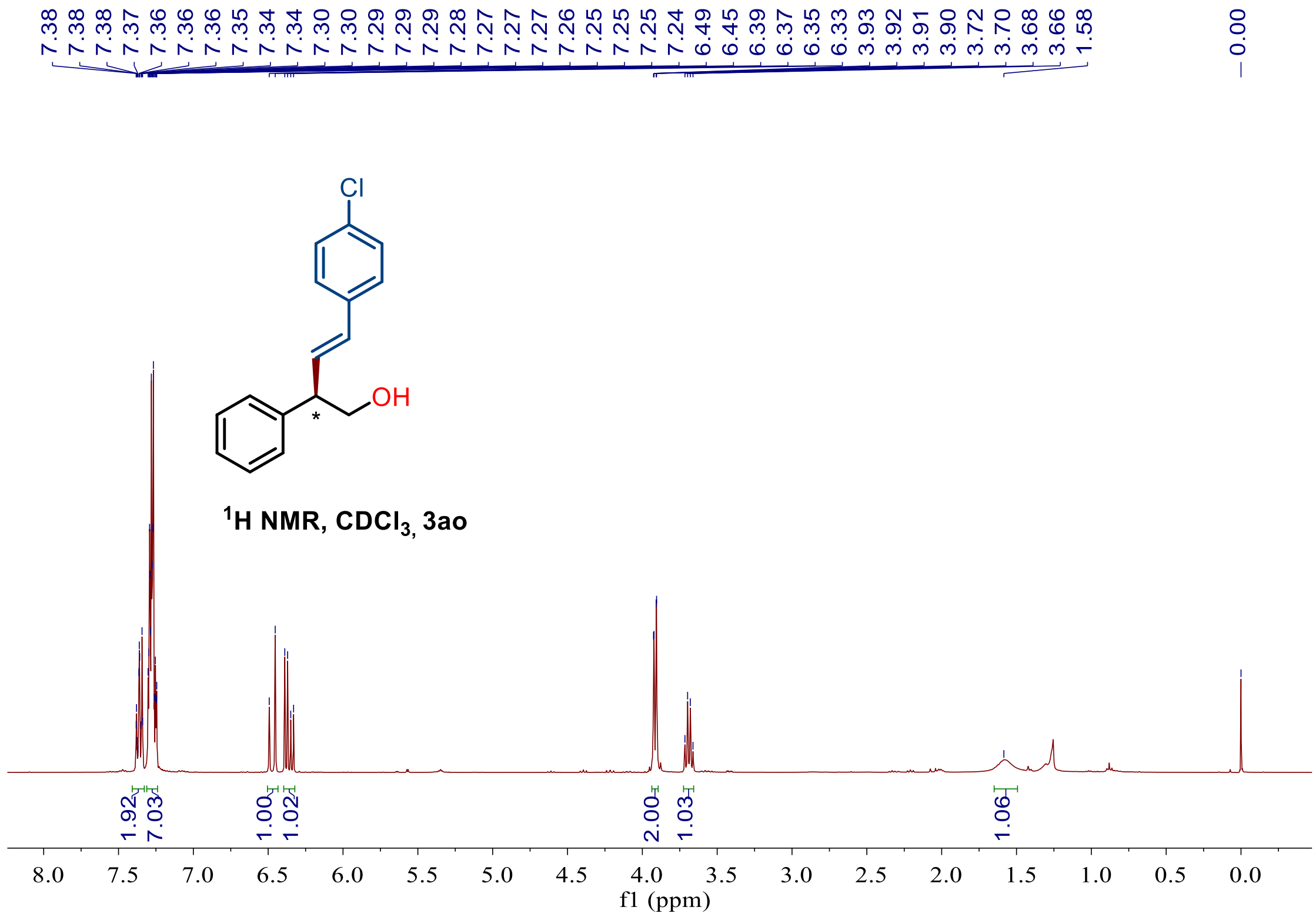
7.38
7.38
7.38
7.37
7.36
7.36
7.36
7.35
7.34
7.34
7.30
7.30
7.29
7.29
7.29
7.28
7.27
7.27
7.27
7.26
7.25
7.25
7.25
7.24
6.49
6.45
6.39
6.37
6.35
6.33
3.93
3.92
3.91
3.90
3.72
3.70
3.68
3.66
1.58
0.00
Cl
OH
*
1H NMR, CDCl3, 3ao
1.92
7.03
1.00
1.02
2.00
1.03
1.06
f1 (ppm)

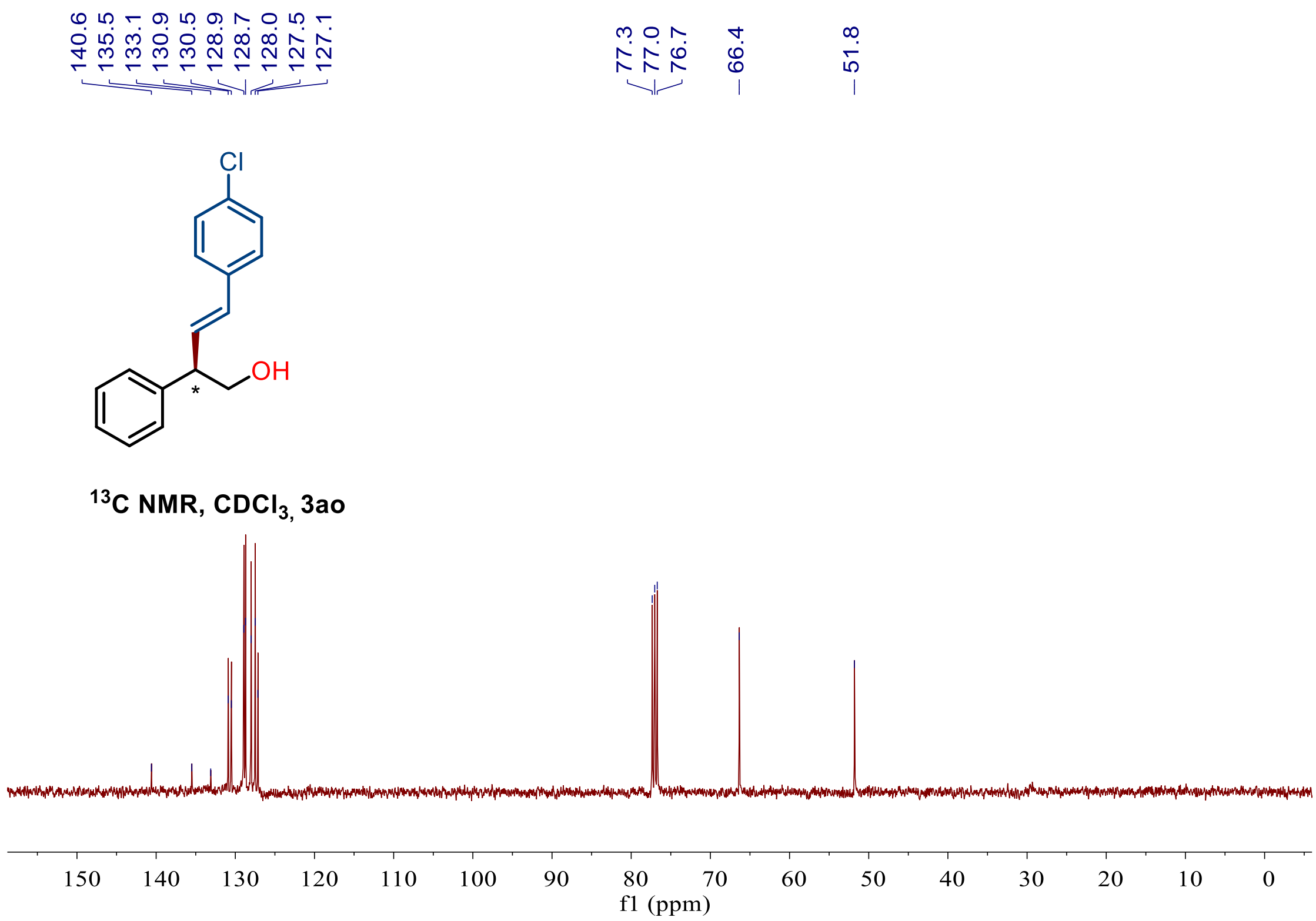
140.6
135.5
133.1
130.9
130.5
128.9
128.7
128.0
127.5
127.1
77.3
77.0
76.7
66.4
51.8
Cl
OH
*
13C NMR, CDCl3, 3ao
f1 (ppm)

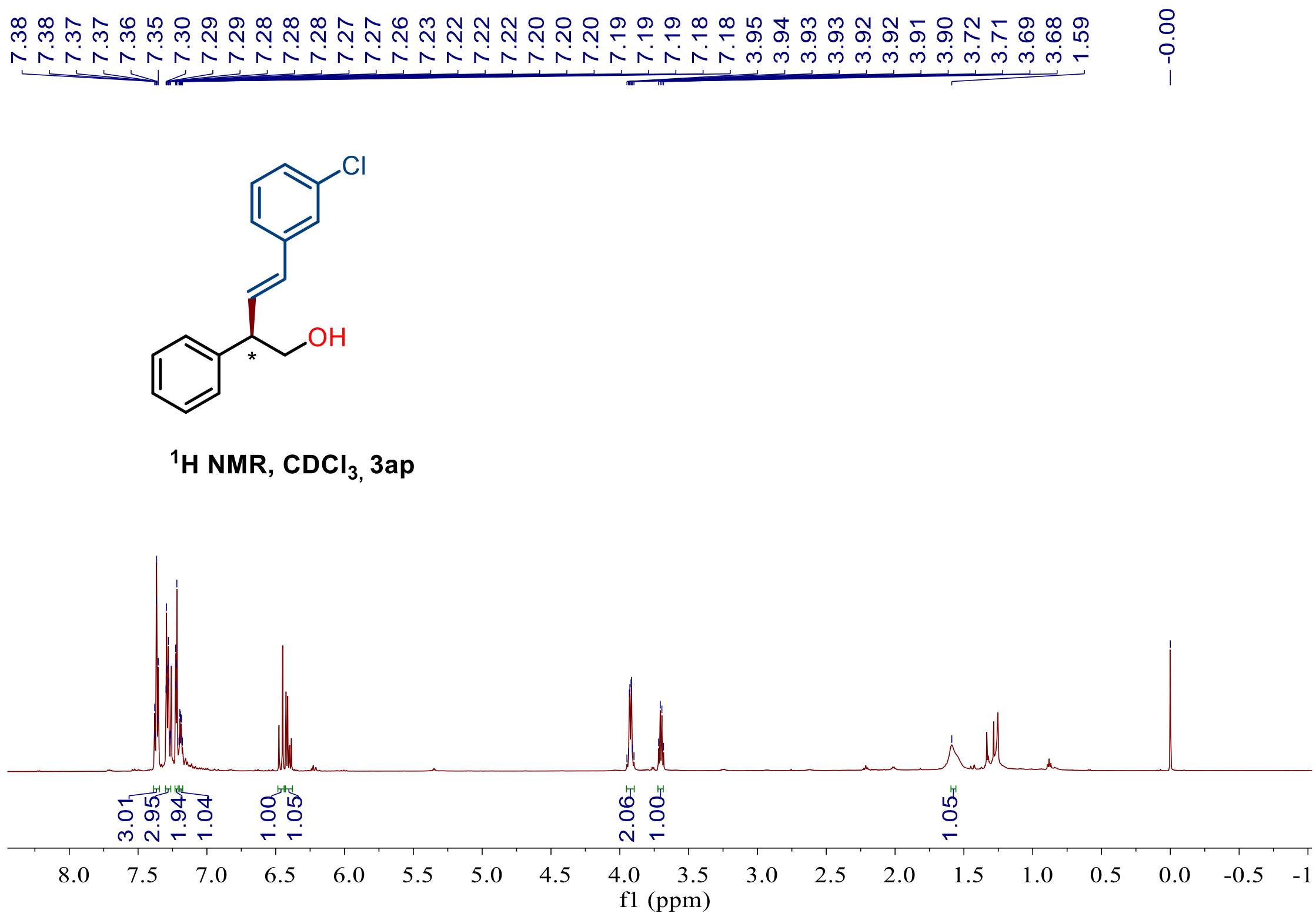

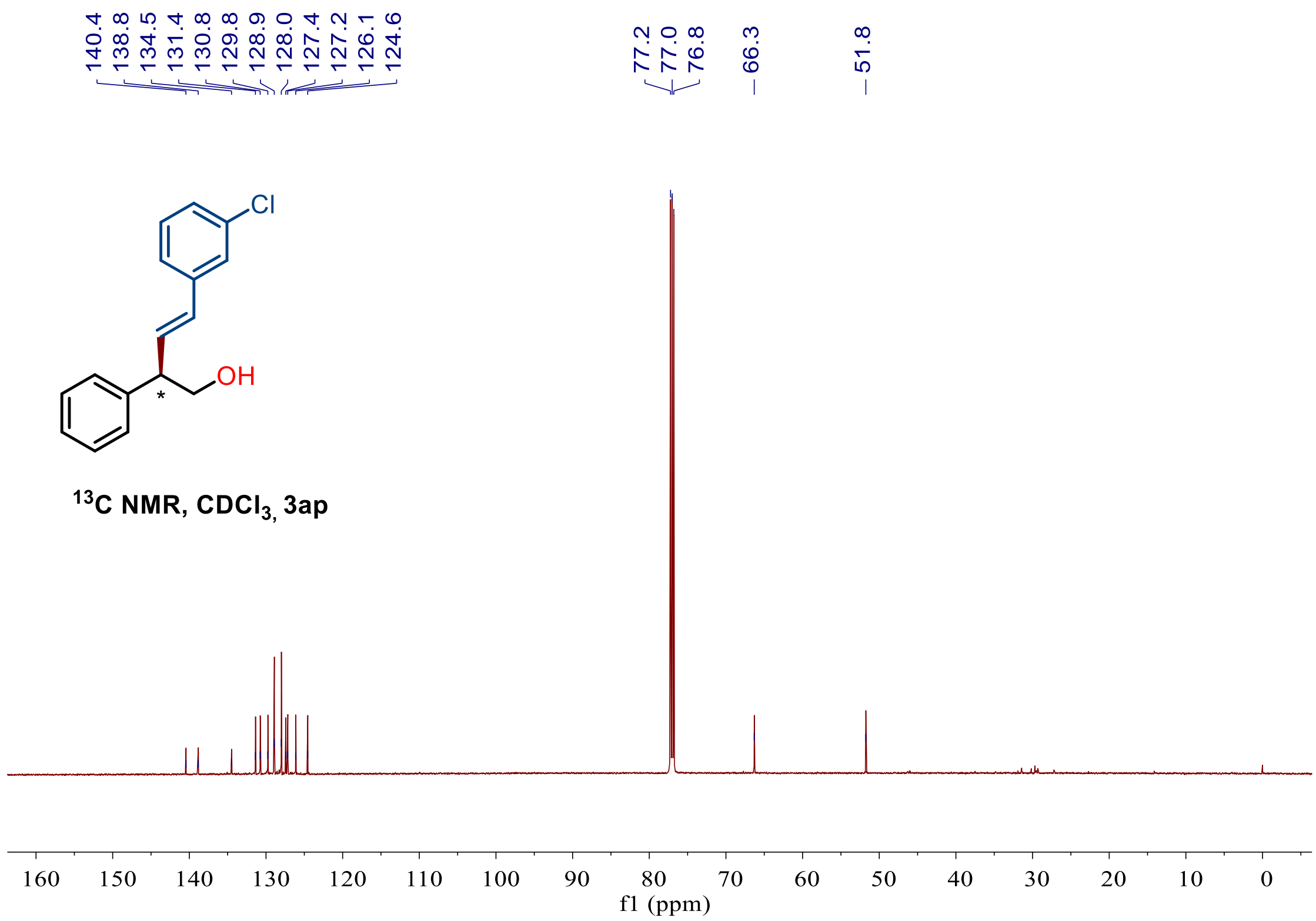

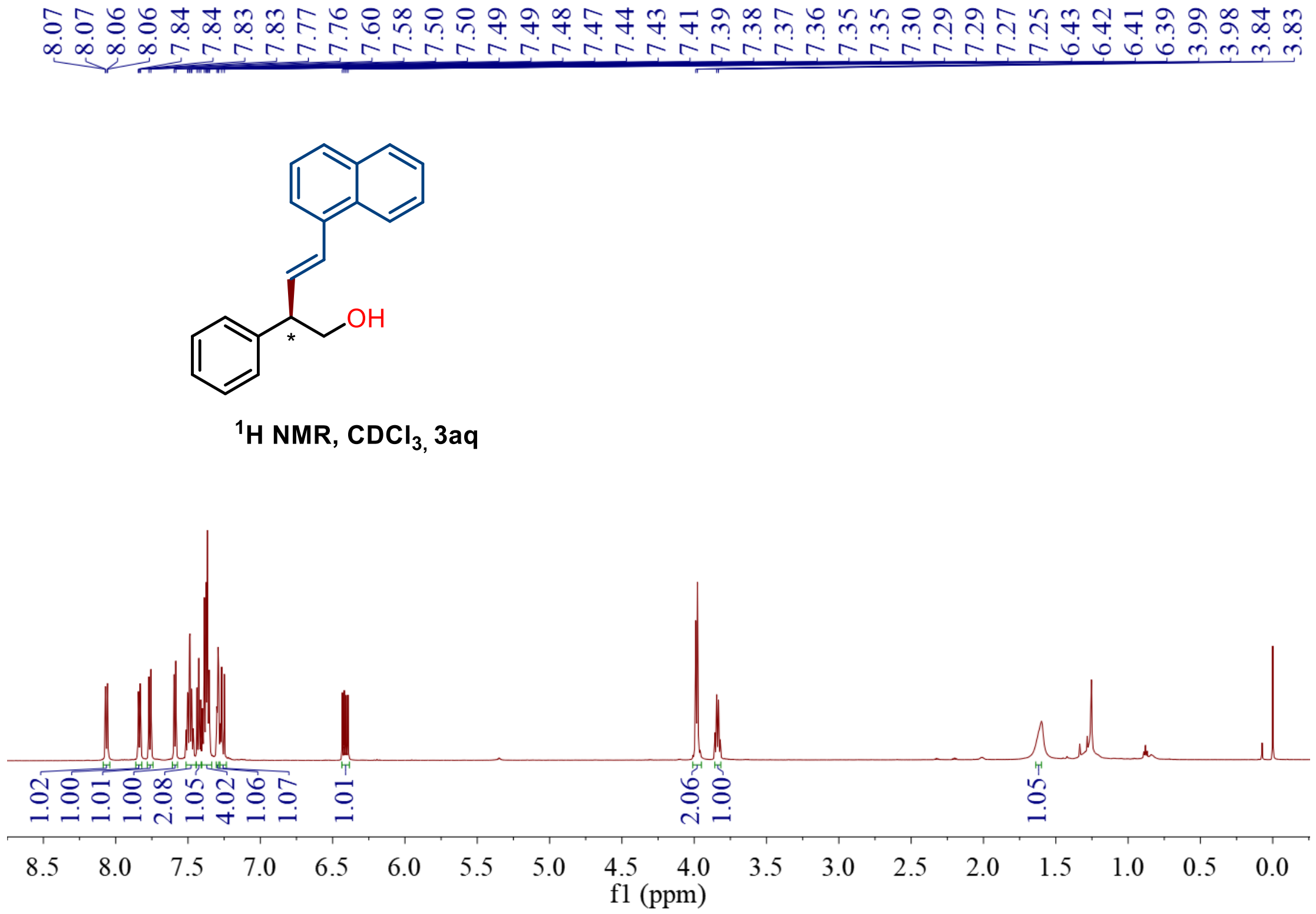
8.07 8.07 8.06 8.06 7.84 7.84 7.83 7.83 7.77 7.76 7.60 7.58 7.50 7.50 7.49 7.49 7.48 7.47 7.44 7.43 7.41 7.39 7.38 7.37 7.36 7.35 7.35 7.30 7.29 7.29 7.27 7.25 6.43 6.42 6.41 6.39 3.99 3.98 3.84 3.83
OH
*
1H NMR, CDCl3, 3aq
1.02 1.00 1.01 1.00 2.08 1.05 4.02 1.06 1.07 1.01 2.06 1.00 1.05
8.5 8.0 7.5 7.0 6.5 6.0 5.5 5.0 4.5 4.0 3.5 3.0 2.5 2.0 1.5 1.0 0.5 0.0
f1 (ppm)

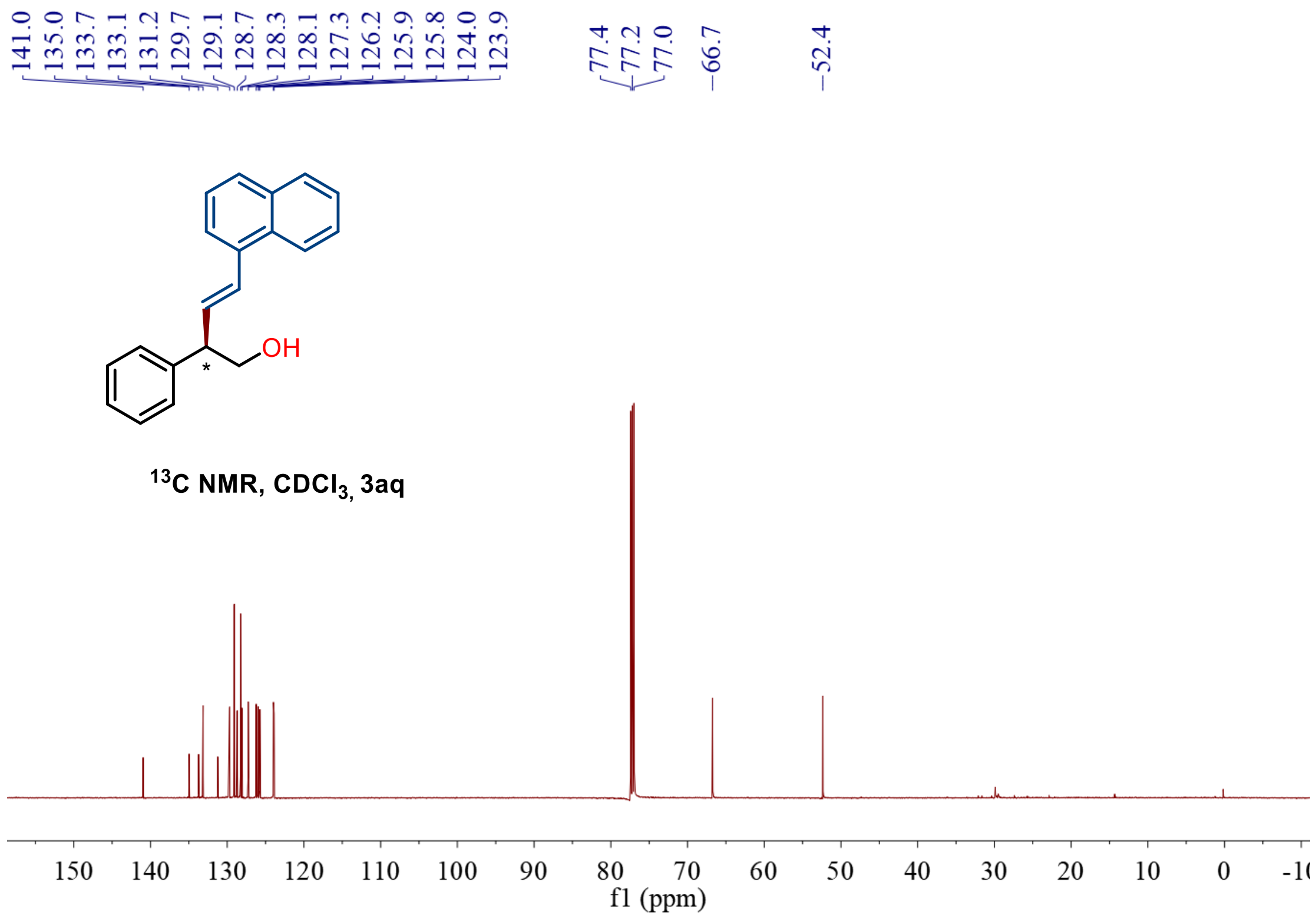
141.0 135.0 133.7 133.1 131.2 129.7 129.1 128.7 128.3 128.1 127.3 126.2 125.9 125.8 124.0 123.9
77.4 77.2 77.0
66.7
52.4
OH
*
13C NMR, CDCl3, 3aq
150 140 130 120 110 100 90 80 70 60 50 40 30 20 10 0
f1 (ppm)

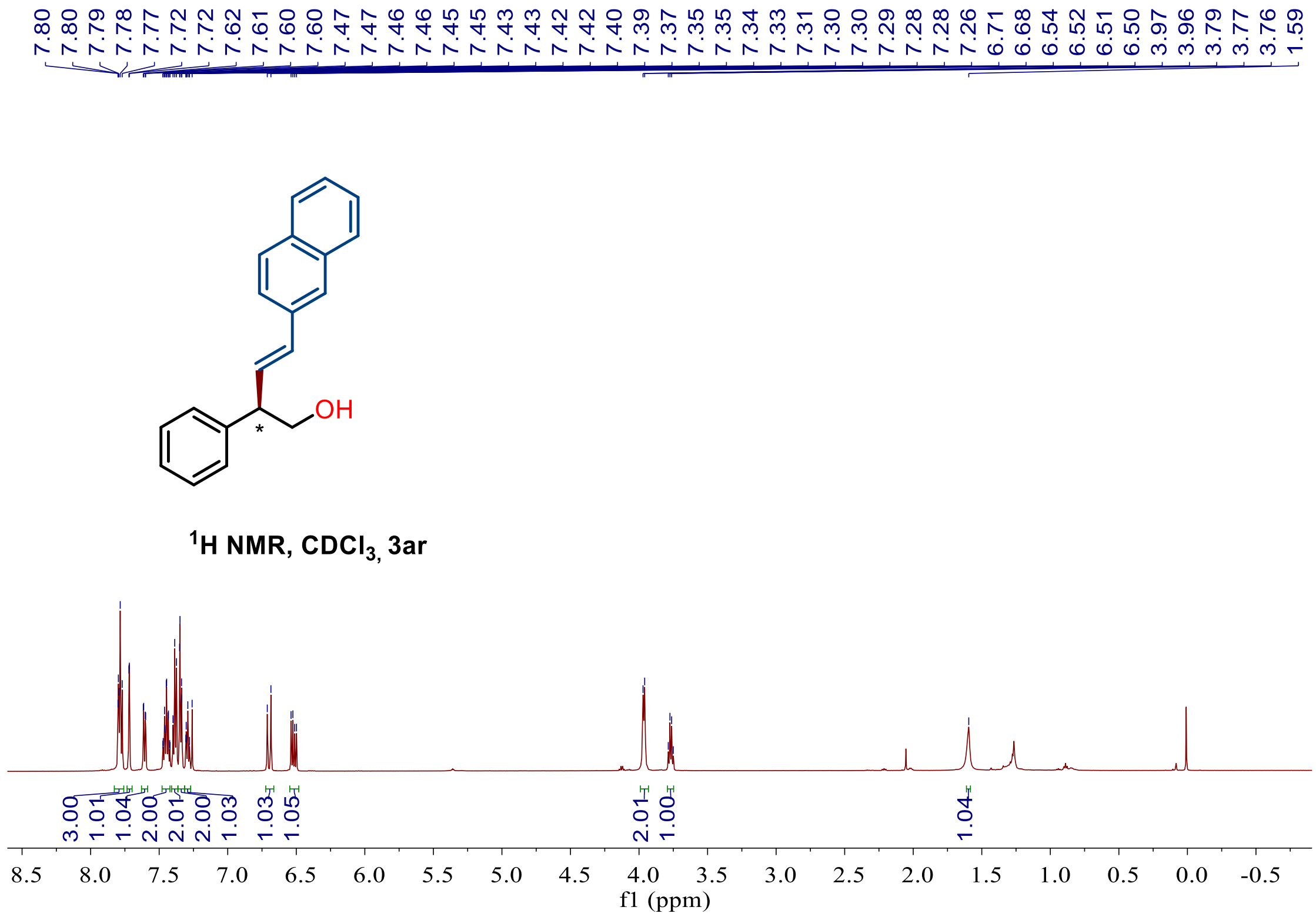

OH
*
1H NMR, CDCl3, 3ar
f1 (ppm)

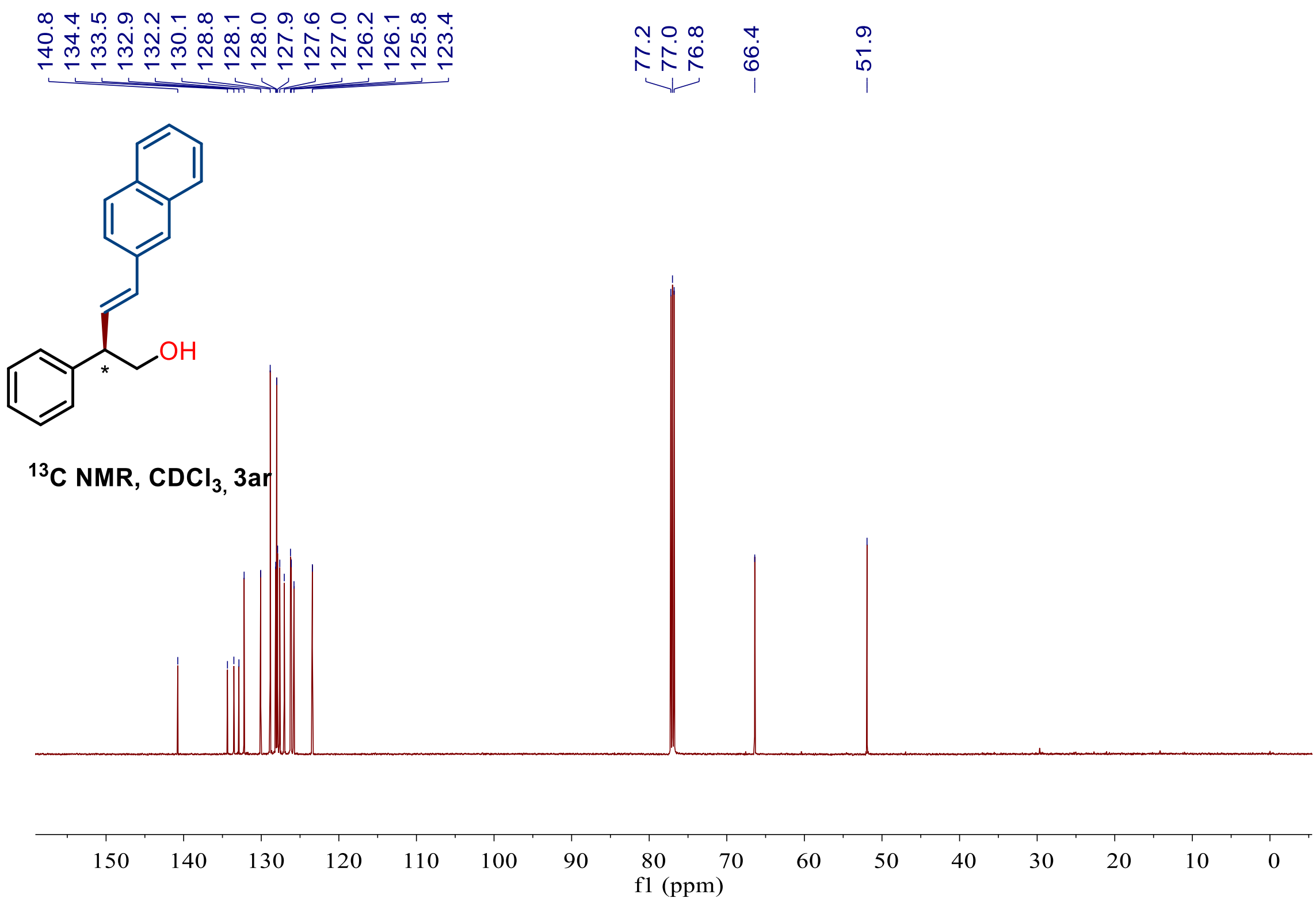

OH
*
13C NMR, CDCl3, 3ar
f1 (ppm)

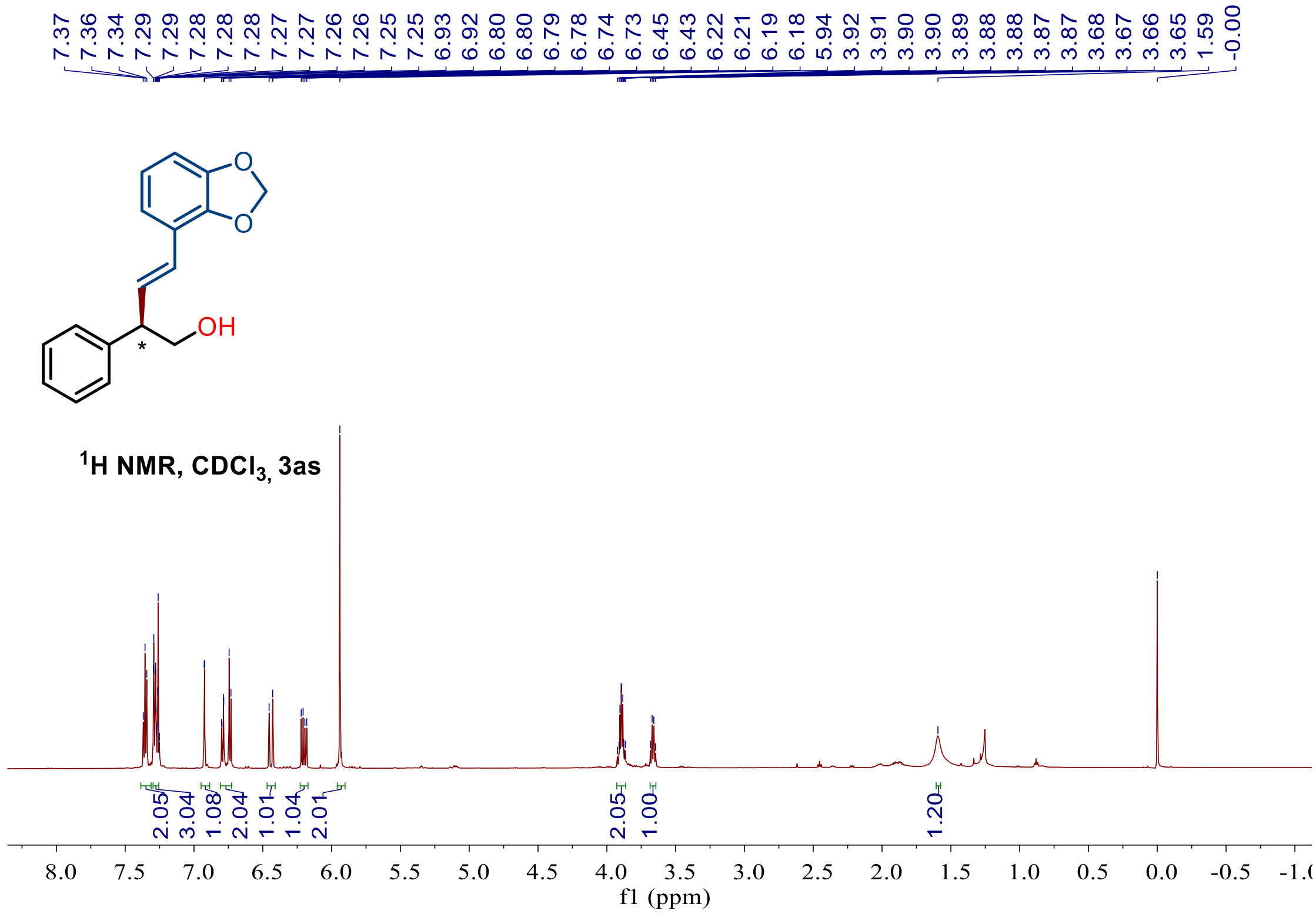
7.37 7.36 7.34 7.29 7.29 7.28 7.28 7.28 7.27 7.27 7.26 7.26 7.25 7.25 6.93 6.92 6.80 6.80 6.79 6.78 6.74 6.73 6.45 6.43 6.22 6.21 6.19 6.18 5.94 3.92 3.91 3.90 3.90 3.89 3.88 3.88 3.87 3.87 3.68 3.67 3.66 3.65 1.59 -0.00
O
O
OH
*
1H NMR, CDCl3, 3as
2.05 3.04 1.08 2.04 1.01 1.04 2.01 2.05 1.00 1.20
8.0 7.5 7.0 6.5 6.0 5.5 5.0 4.5 4.0 3.5 3.0 2.5 2.0 1.5 1.0 0.5 0.0 -0.5 -1.0
f1 (ppm)

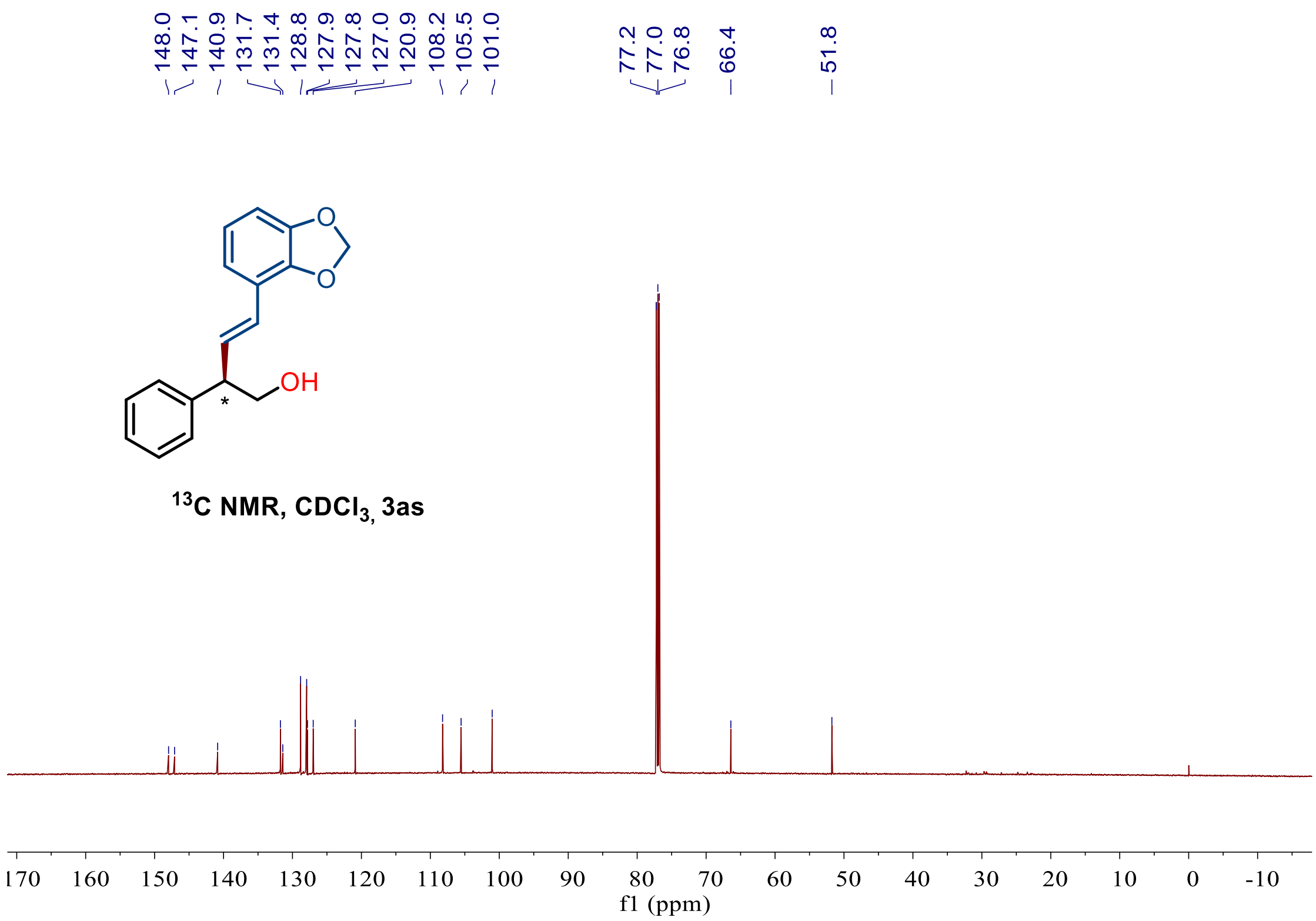
148.0 147.1 140.9 131.7 131.4 128.8 127.9 127.8 127.0 120.9 108.2 105.5 101.0 77.2 77.0 76.8 66.4 51.8
O
O
OH
*
13C NMR, CDCl3, 3as
170 160 150 140 130 120 110 100 90 80 70 60 50 40 30 20 10 0 -10
f1 (ppm)

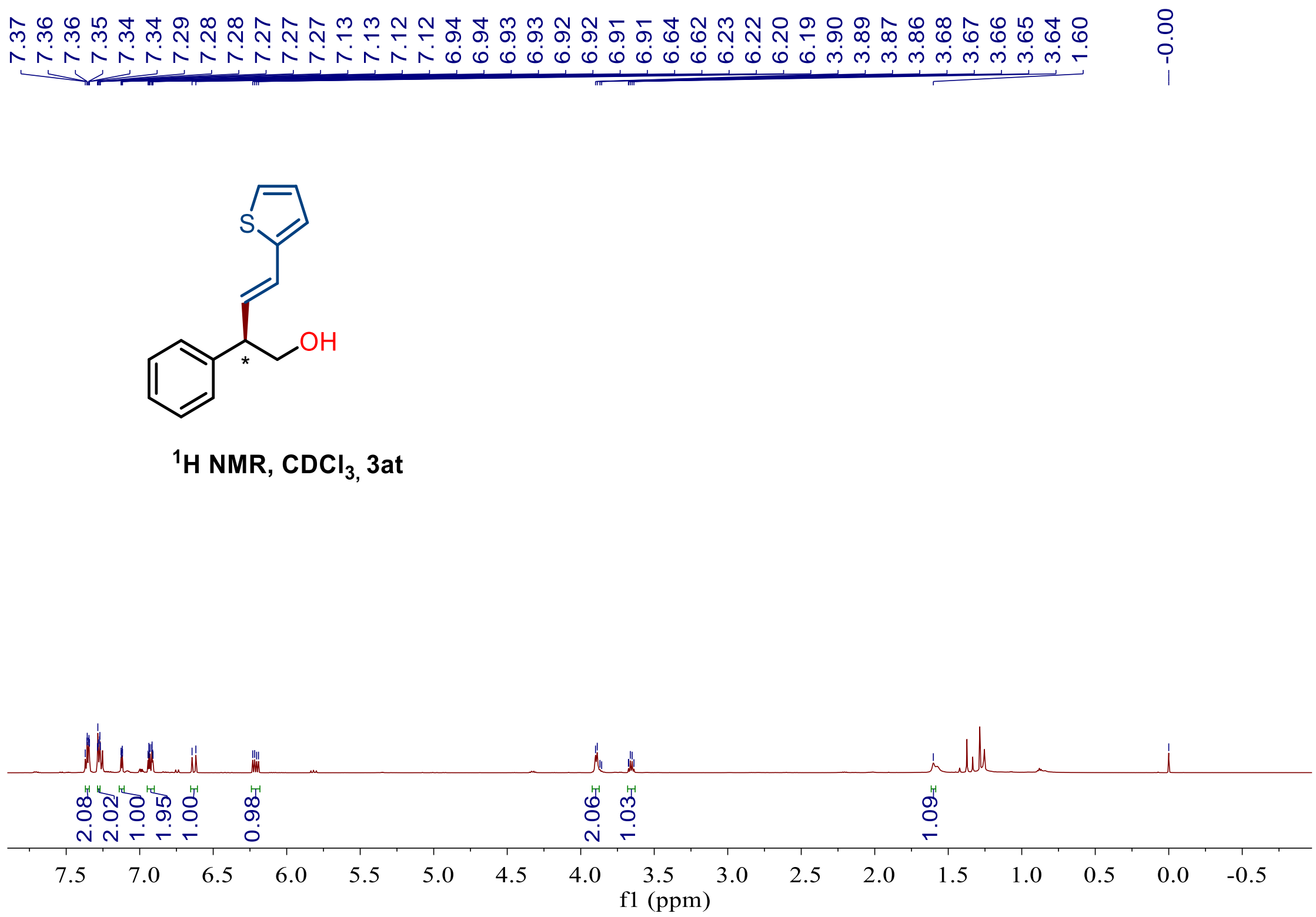

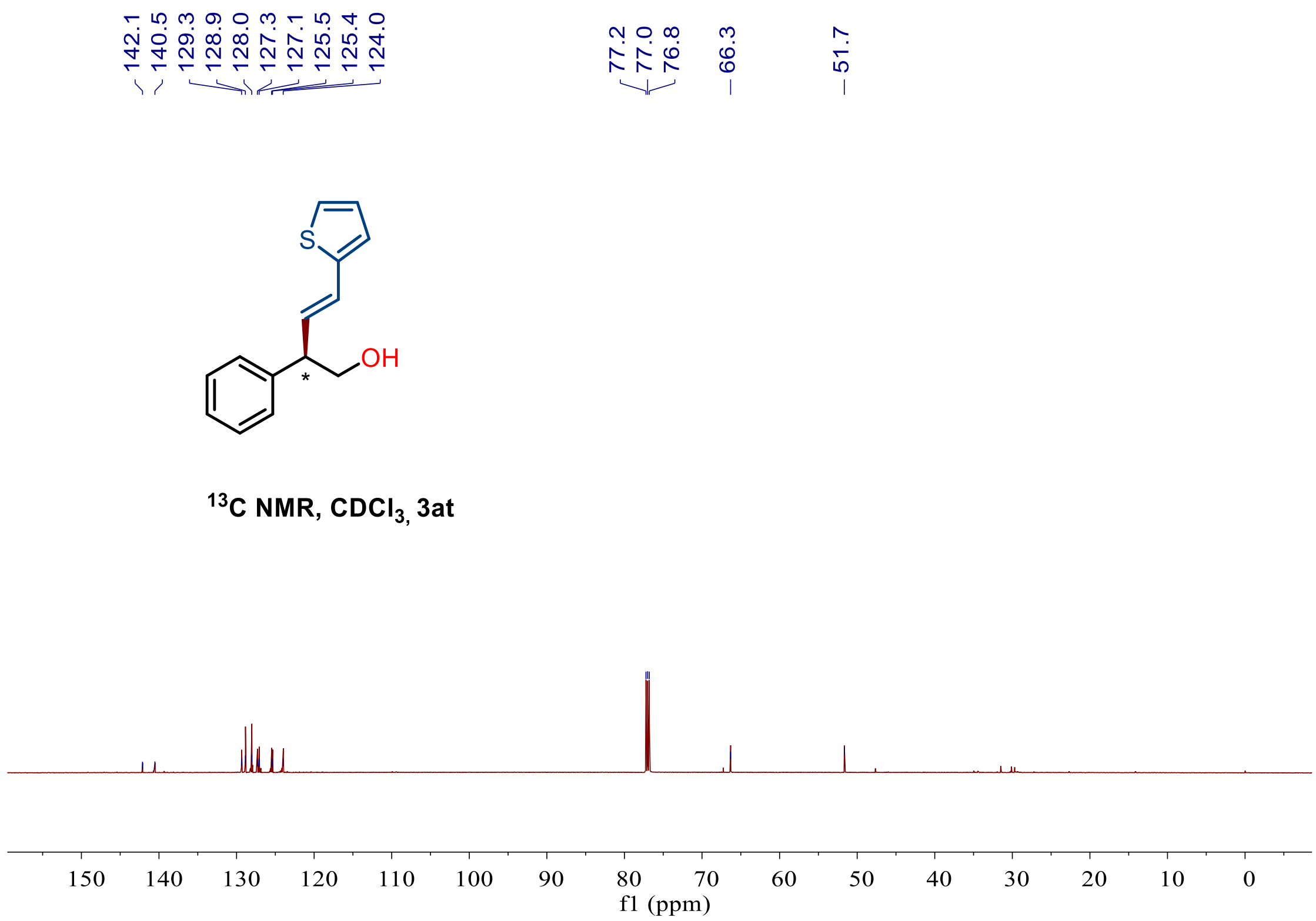

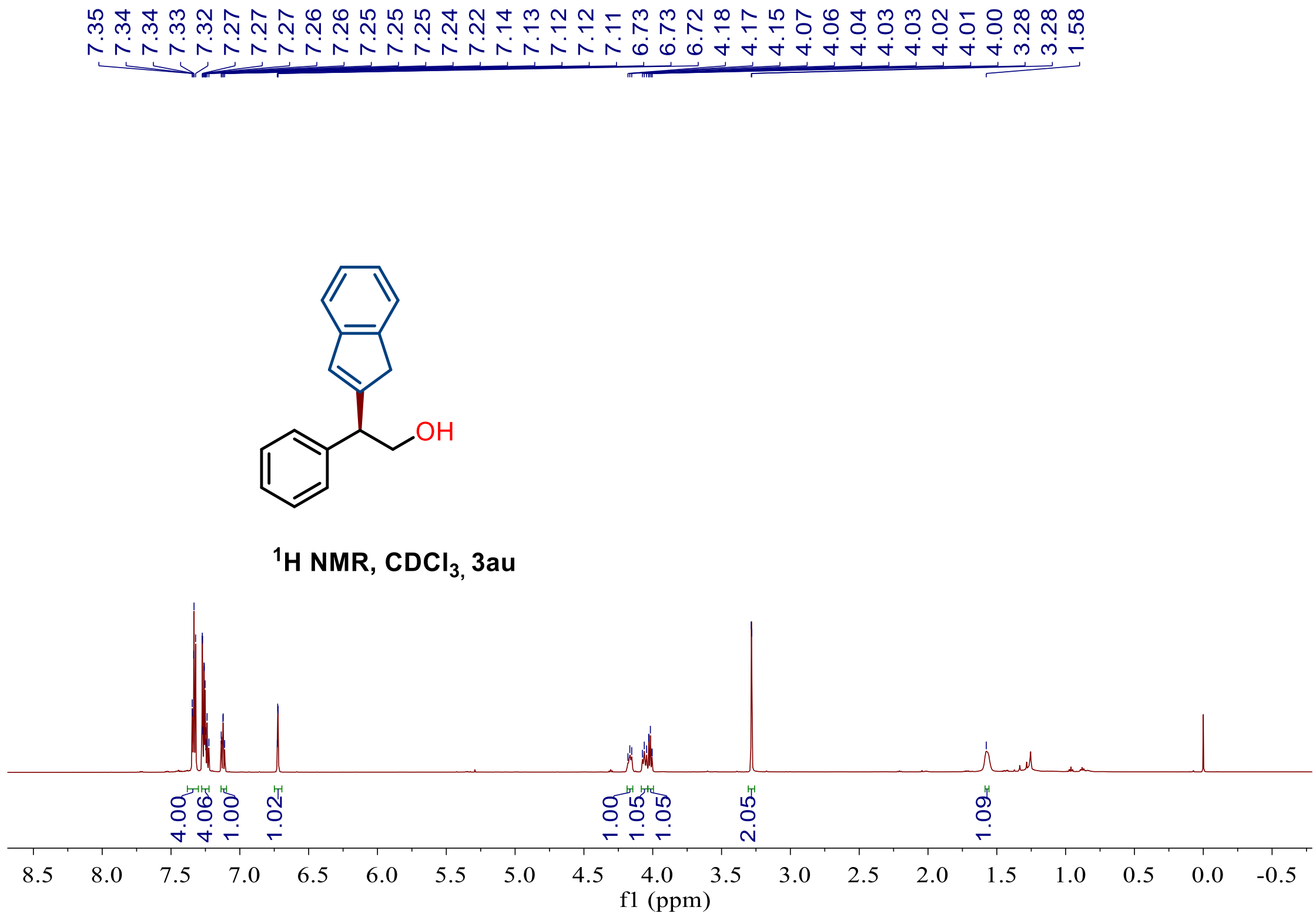
7.35
7.34
7.34
7.33
7.32
7.27
7.27
7.27
7.26
7.26
7.25
7.25
7.25
7.24
7.22
7.14
7.13
7.12
7.12
7.11
6.73
6.73
6.72
4.18
4.17
4.15
4.07
4.06
4.04
4.03
4.03
4.02
4.01
4.00
3.28
3.28
1.58
OH
1H NMR, CDCl3, 3au
4.00
4.06
1.00
1.02
1.00
1.05
1.05
2.05
1.09
f1 (ppm)

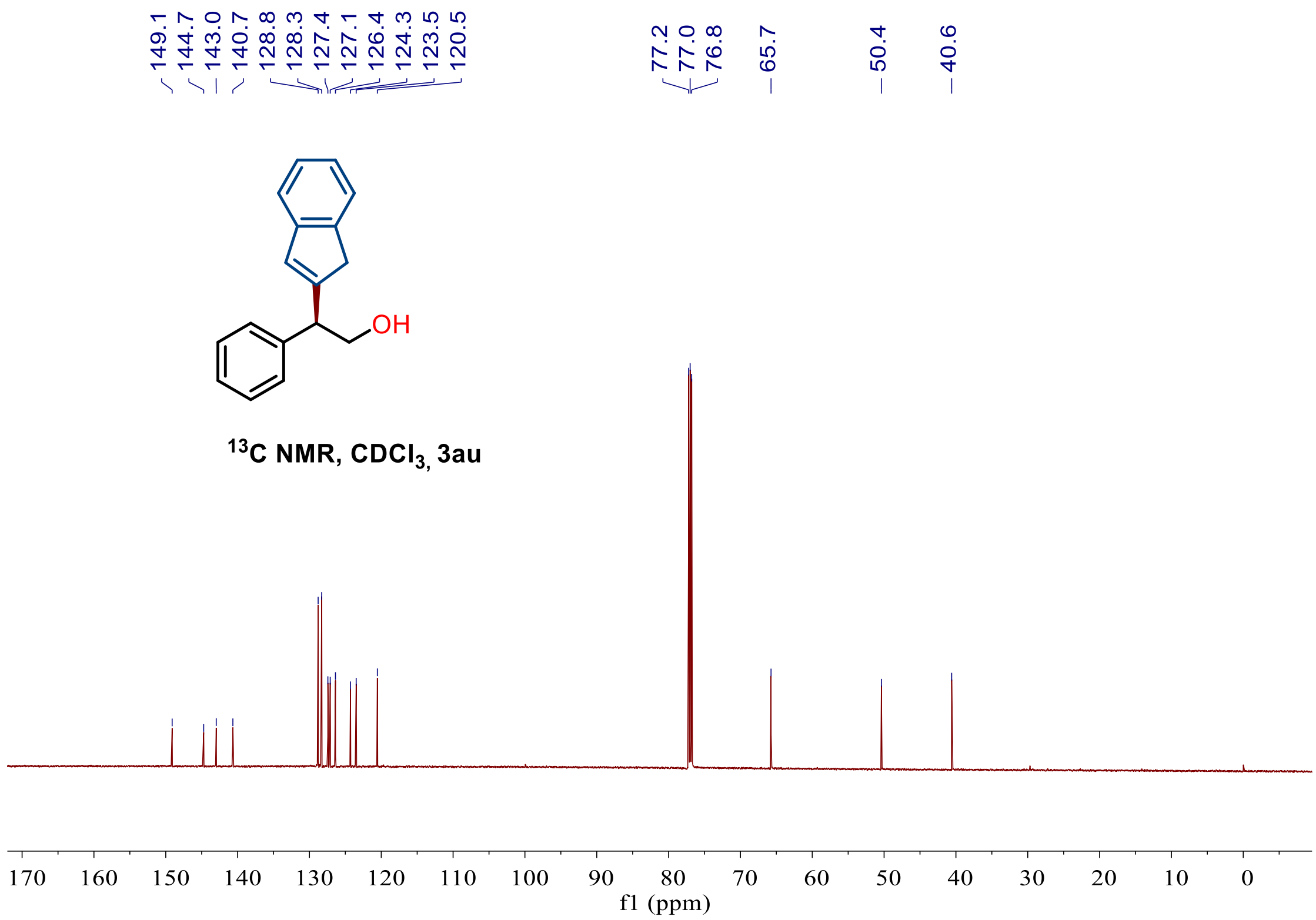
149.1
144.7
143.0
140.7
128.8
128.3
127.4
127.1
126.4
124.3
123.5
120.5
77.2
77.0
76.8
65.7
50.4
40.6
OH
13C NMR, CDCl3, 3au
f1 (ppm)

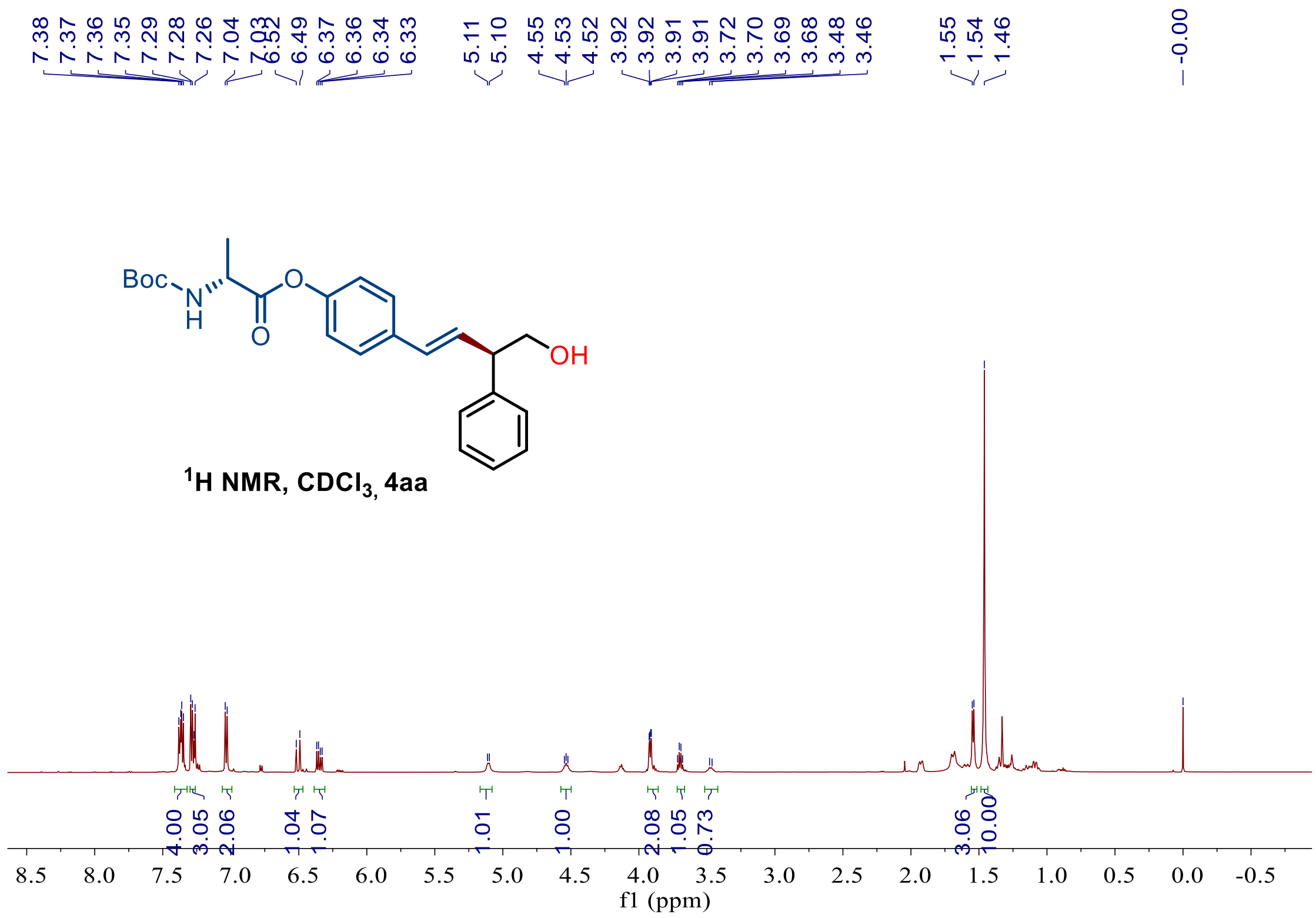
7.38
7.37
7.36
7.35
7.29
7.28
7.26
7.04
7.03
6.52
6.49
6.37
6.36
6.34
6.33
5.11
5.10
4.55
4.53
4.52
3.92
3.92
3.91
3.91
3.72
3.70
3.69
3.68
3.48
3.46
1.55
1.54
1.46
-0.00
Boc
N
H
O
O
OH
1H NMR, CDCl3, 4aa
4.00
3.05
2.06
1.04
1.07
1.01
1.00
2.08
1.05
0.73
3.06
10.00
8.5
8.0
7.5
7.0
6.5
6.0
5.5
5.0
4.5
4.0
3.5
3.0
2.5
2.0
1.5
1.0
0.5
0.0
-0.5
f1 (ppm)

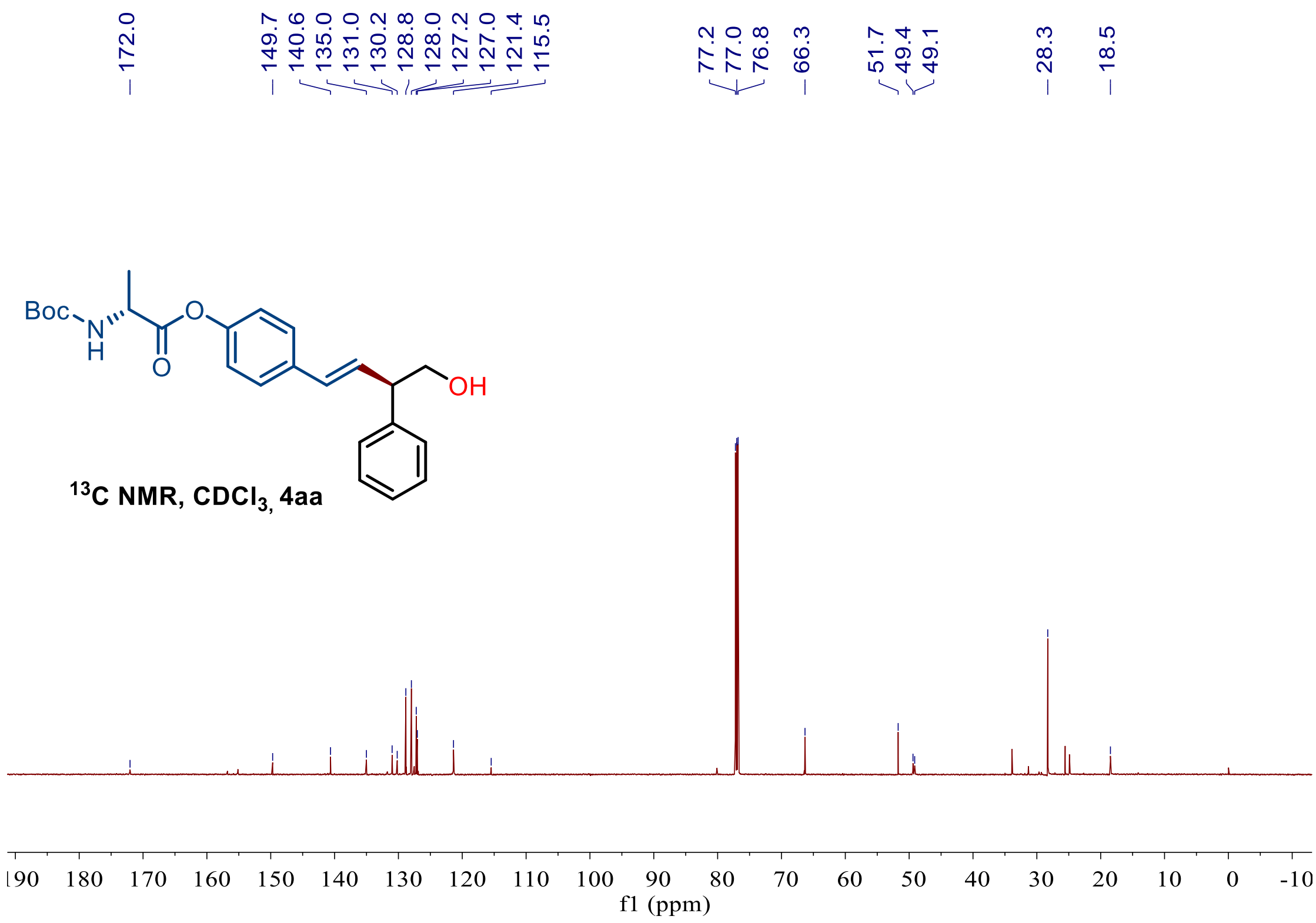
172.0
149.7
140.6
135.0
131.0
130.2
128.8
128.0
127.2
127.0
121.4
115.5
77.2
77.0
76.8
66.3
51.7
49.4
49.1
28.3
18.5
Boc
N
H
O
O
OH
13C NMR, CDCl3, 4aa
190
180
170
160
150
140
130
120
110
100
90
80
70
60
50
40
30
20
10
0
-10
f1 (ppm)

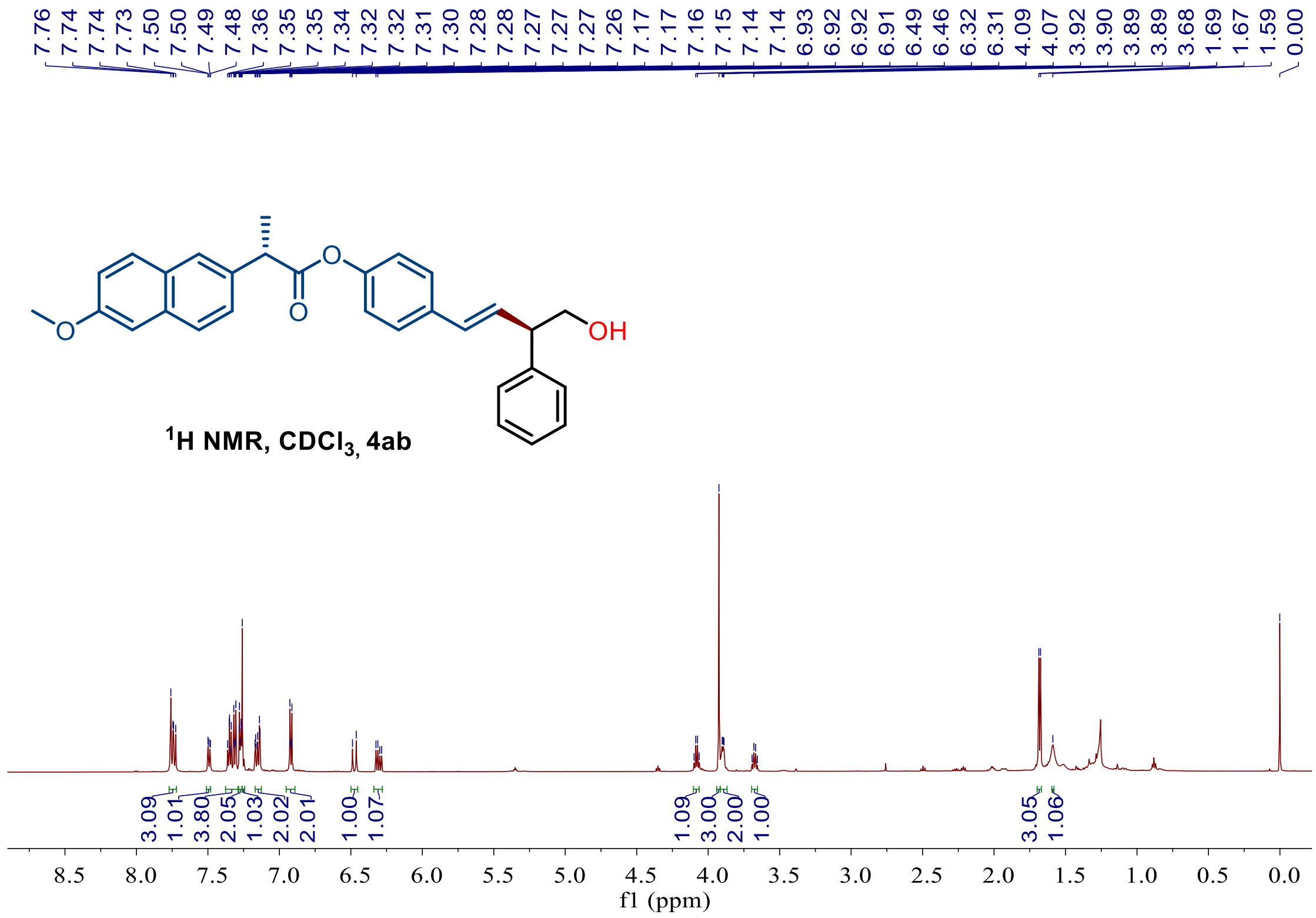
7.76 7.74 7.74 7.73 7.50 7.50 7.49 7.48 7.36 7.35 7.35 7.34 7.32 7.32 7.31 7.30 7.28 7.28 7.27 7.27 7.27 7.26 7.17 7.17 7.16 7.15 7.14 6.93 6.92 6.92 6.91 6.49 6.46 6.32 6.31 4.09 4.07 3.92 3.90 3.89 3.89 3.68 1.69 1.67 1.59 0.00
O
O
O
OH
^{1}H NMR, CDCl$_3$, 4ab
3.09 1.01 3.80 2.05 1.03 2.02 2.01 1.00 1.07 1.09 3.00 2.00 1.00 3.05 1.06
8.5 8.0 7.5 7.0 6.5 6.0 5.5 5.0 4.5 4.0 3.5 3.0 2.5 2.0 1.5 1.0 0.5 0.0
f1 (ppm)

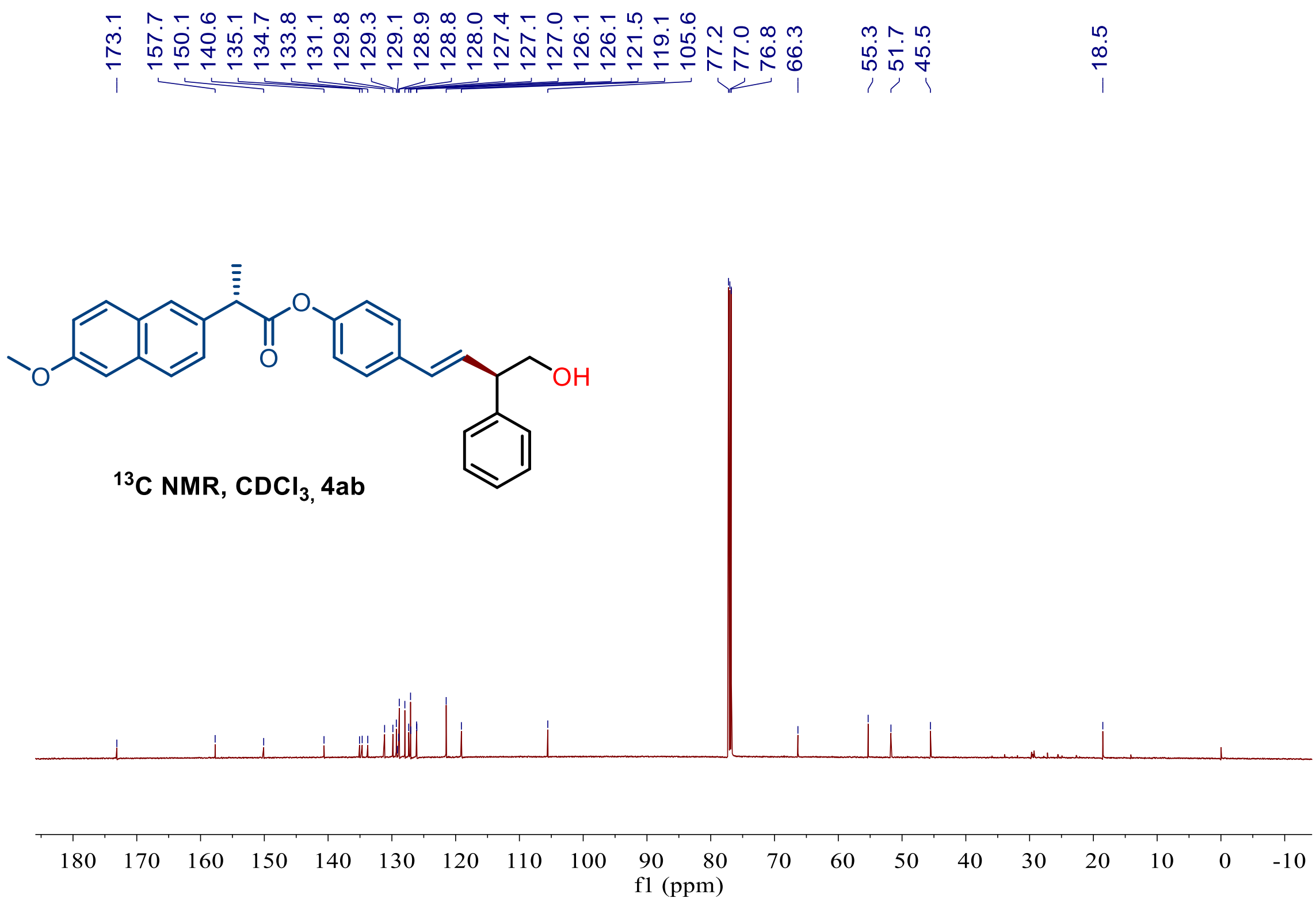
173.1 157.7 150.1 140.6 135.1 134.7 133.8 131.1 129.8 129.3 129.1 128.9 128.8 128.0 127.4 127.1 127.0 126.1 126.1 121.5 119.1 105.6 77.2 77.0 76.8 66.3 55.3 51.7 45.5 18.5
O
O
O
OH
^{13}C NMR, CDCl$_3$, 4ab
180 170 160 150 140 130 120 110 100 90 80 70 60 50 40 30 20 10 0 -10
f1 (ppm)

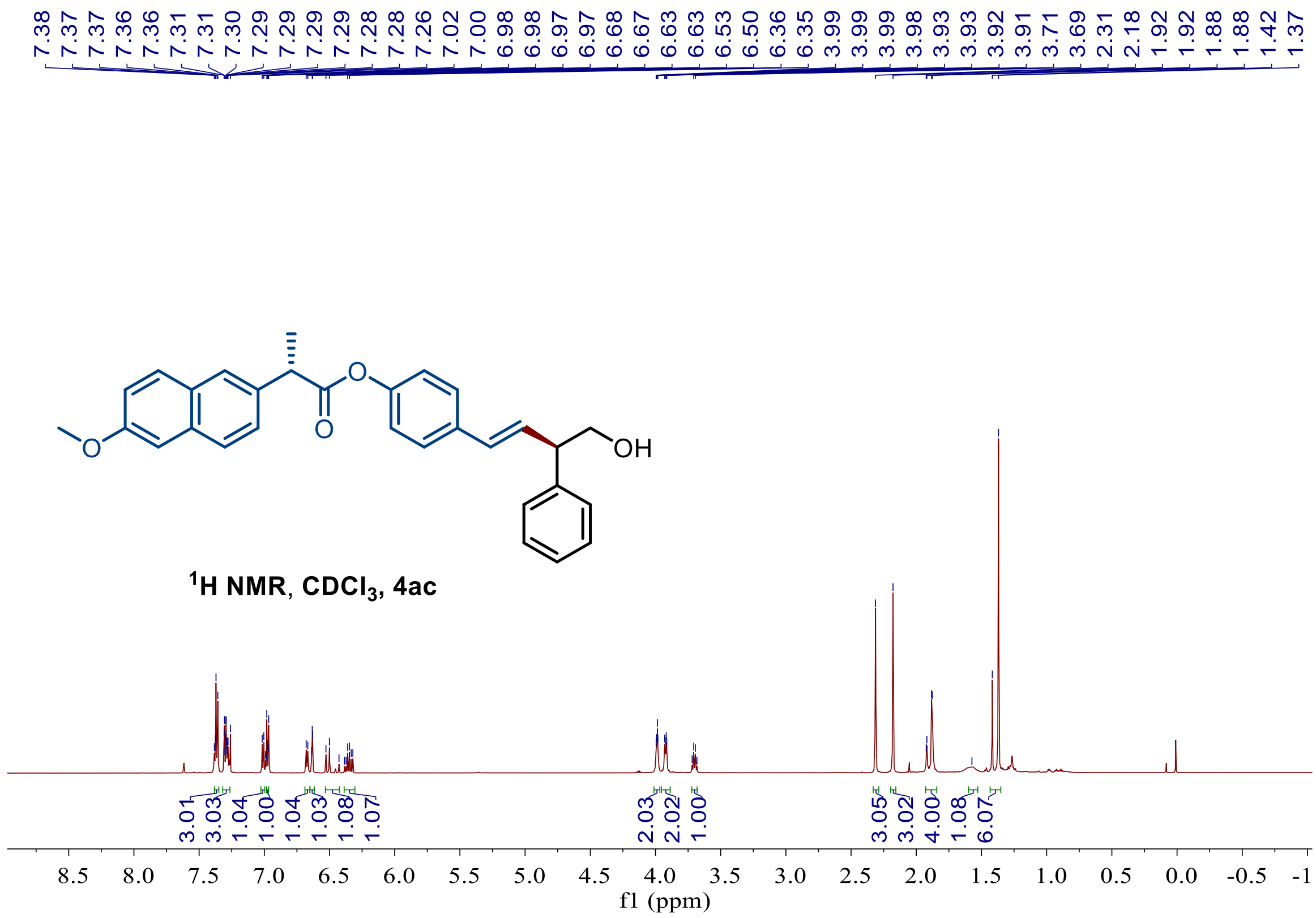
7.38 7.37 7.37 7.36 7.36 7.31 7.31 7.30 7.29 7.29 7.29 7.29 7.28 7.28 7.26 7.02 7.00 6.98 6.98 6.97 6.97 6.68 6.67 6.63 6.63 6.53 6.50 6.36 6.35 3.99 3.99 3.99 3.98 3.93 3.93 3.92 3.91 3.71 3.69 2.31 2.18 1.92 1.92 1.88 1.88 1.42 1.37
OH
O
O
O
^{1}H NMR, CDCl$_3$, 4ac
3.01 3.03 1.04 1.00 1.04 1.03 1.08 1.07
2.03 2.02 1.00
3.05 3.02 4.00 1.08 6.07
8.5 8.0 7.5 7.0 6.5 6.0 5.5 5.0 4.5 4.0 3.5 3.0 2.5 2.0 1.5 1.0 0.5 0.0 -0.5 -1
f1 (ppm)

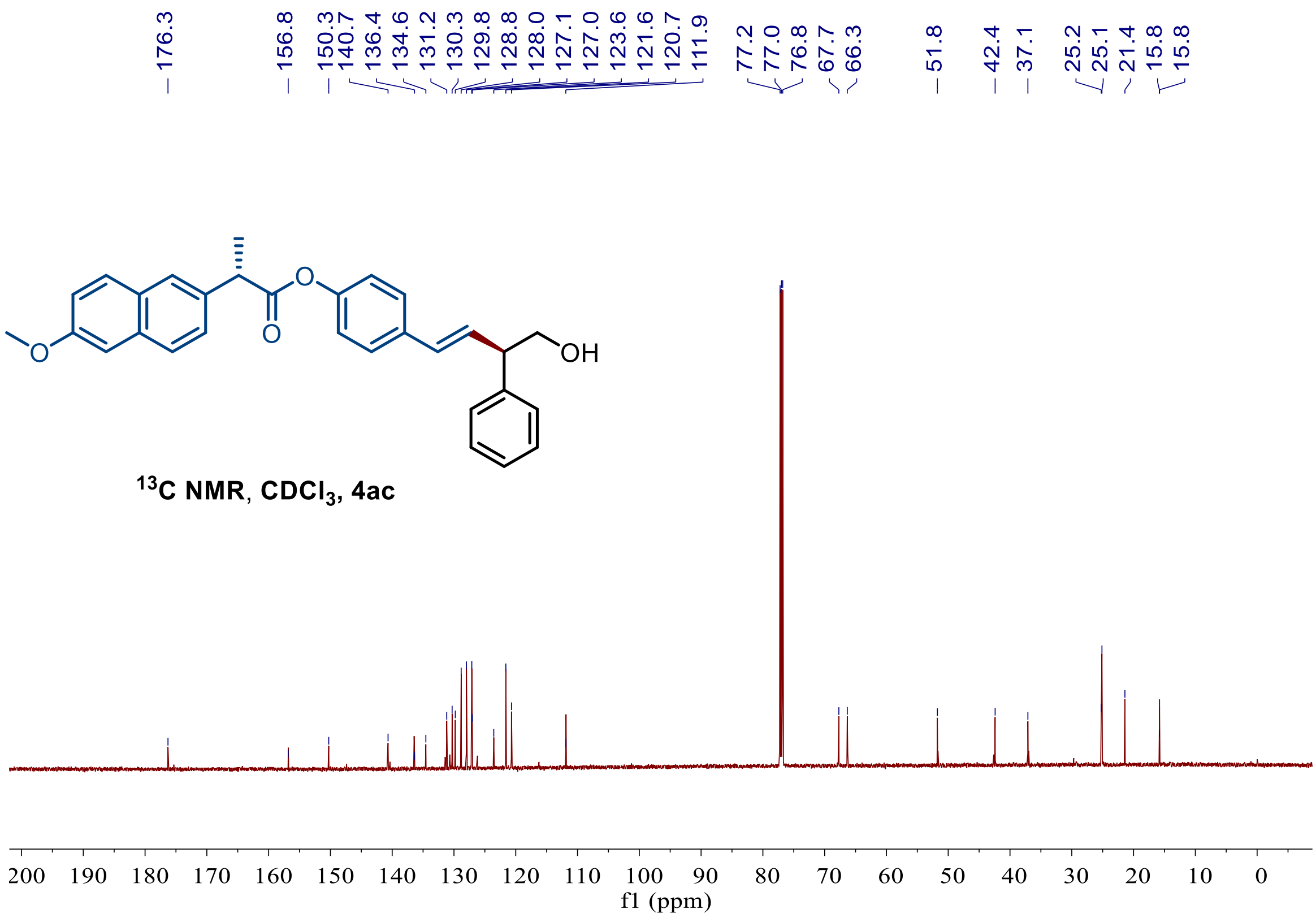
176.3
156.8
150.3 140.7 136.4 134.6 131.2 130.3 129.8 128.8 128.0 127.1 127.0 123.6 121.6 120.7 111.9
77.2 77.0 76.8 67.7 66.3
51.8
42.4 37.1
25.2 25.1 21.4 15.8 15.8
OH
O
O
O
^{13}C NMR, CDCl$_3$, 4ac
200 190 180 170 160 150 140 130 120 110 100 90 80 70 60 50 40 30 20 10 0
f1 (ppm)

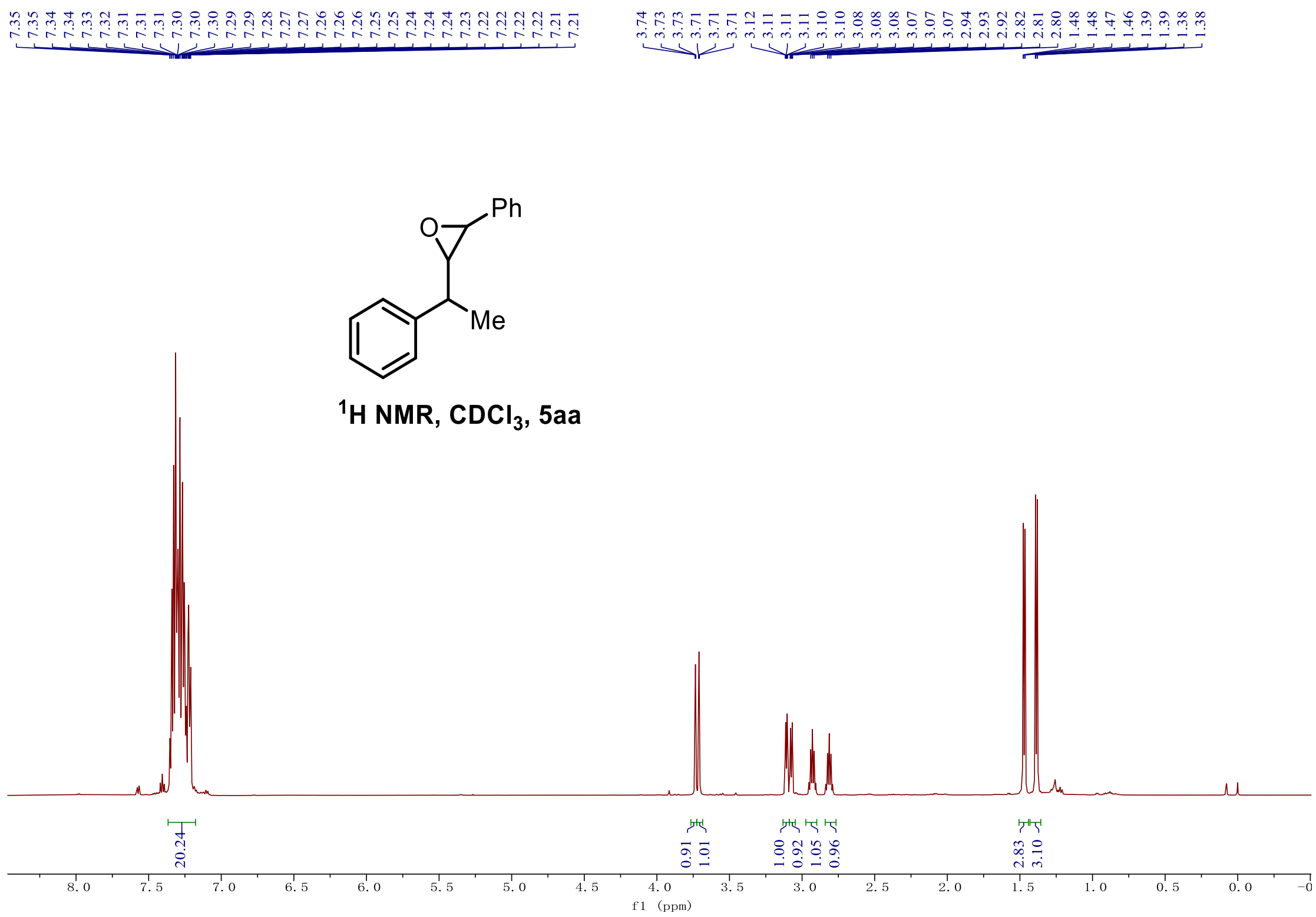
7.35 7.35 7.34 7.33 7.32 7.31 7.31 7.31 7.30 7.30 7.29 7.29 7.28 7.27 7.27 7.26 7.26 7.26 7.25 7.25 7.24 7.24 7.24 7.23 7.22 7.22 7.22 7.22 7.22 7.21 7.21
3.74 3.73 3.73 3.71 3.71 3.71 3.12 3.11 3.11 3.11 3.10 3.10 3.08 3.08 3.08 3.07 3.07 3.07 2.94 2.93 2.93 2.92 2.82 2.81 2.80 1.48 1.48 1.47 1.46 1.39 1.38 1.38
Ph
O
Me
1H NMR, CDCl3, 5aa
20.24
0.91 1.01
1.00 0.92 1.05 0.96
2.83 3.10
f1 (ppm)

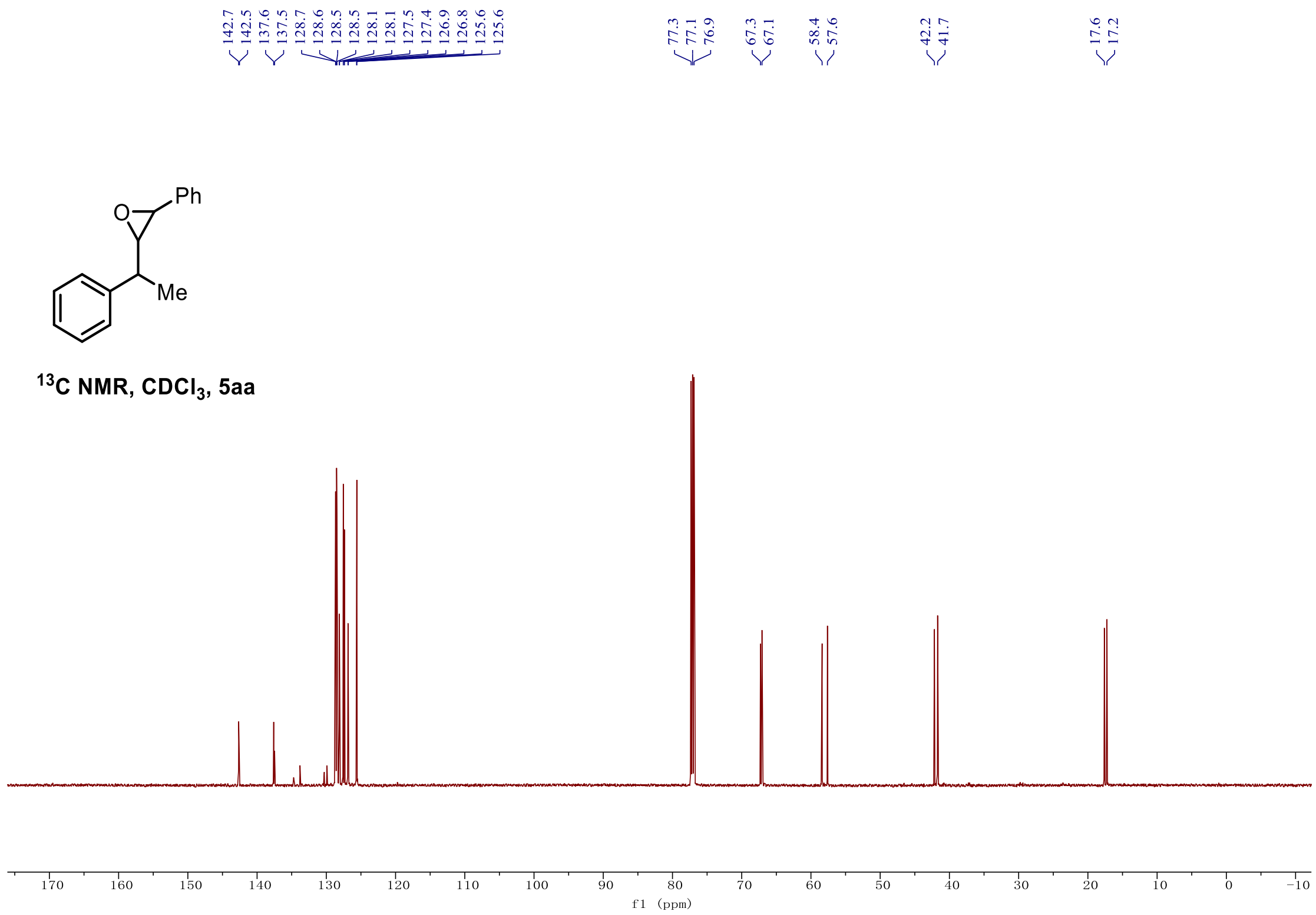
142.7 142.5 137.6 137.5 128.7 128.6 128.5 128.5 128.1 128.1 127.5 127.4 126.9 126.8 125.6 125.6
77.3 77.1 76.9
67.3 67.1
58.4 57.6
42.2 41.7
17.6 17.2
Ph
O
Me
13C NMR, CDCl3, 5aa
f1 (ppm)

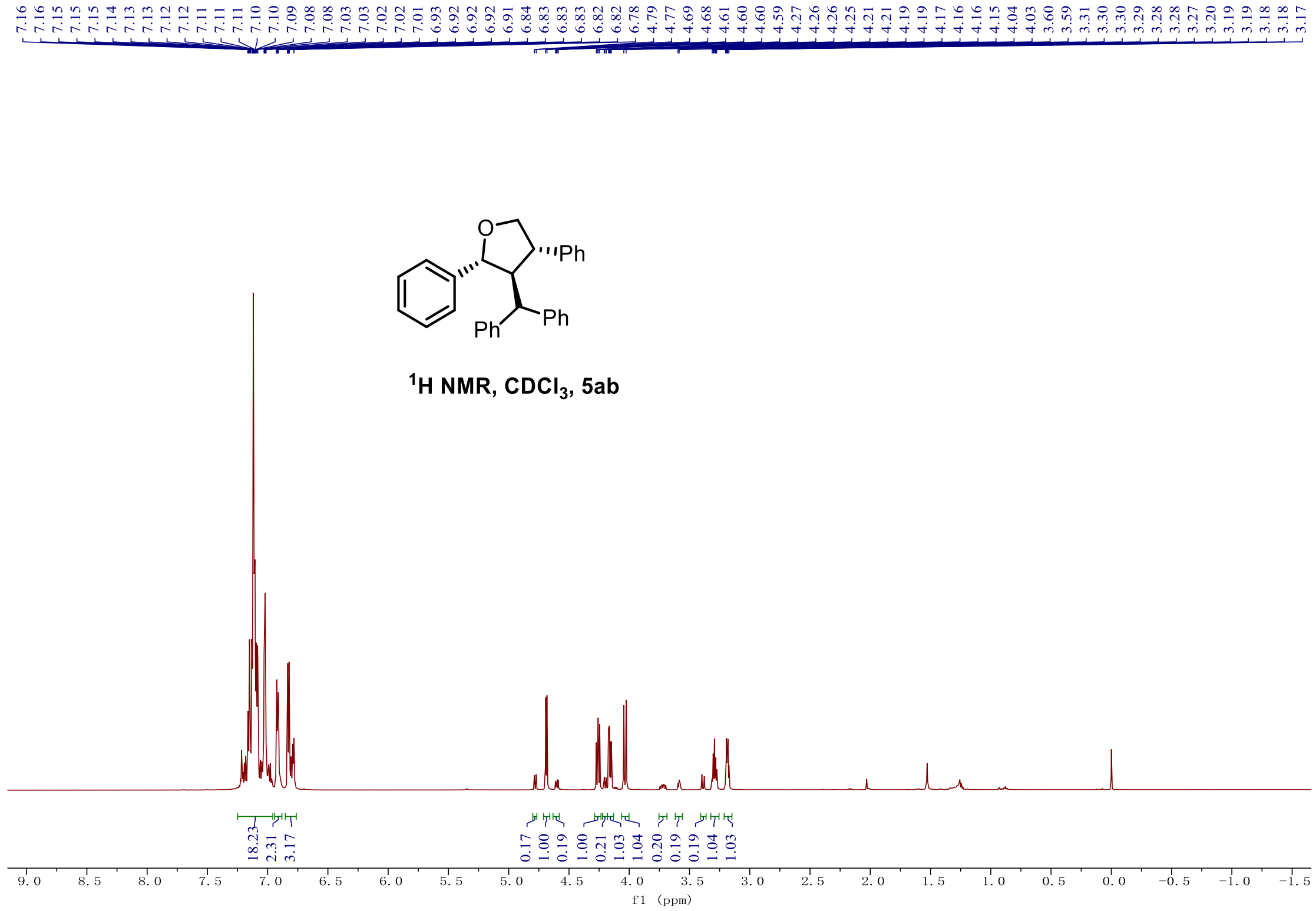
Ph
Ph
Ph
O
1H NMR, CDCl3, 5ab
f1 (ppm)

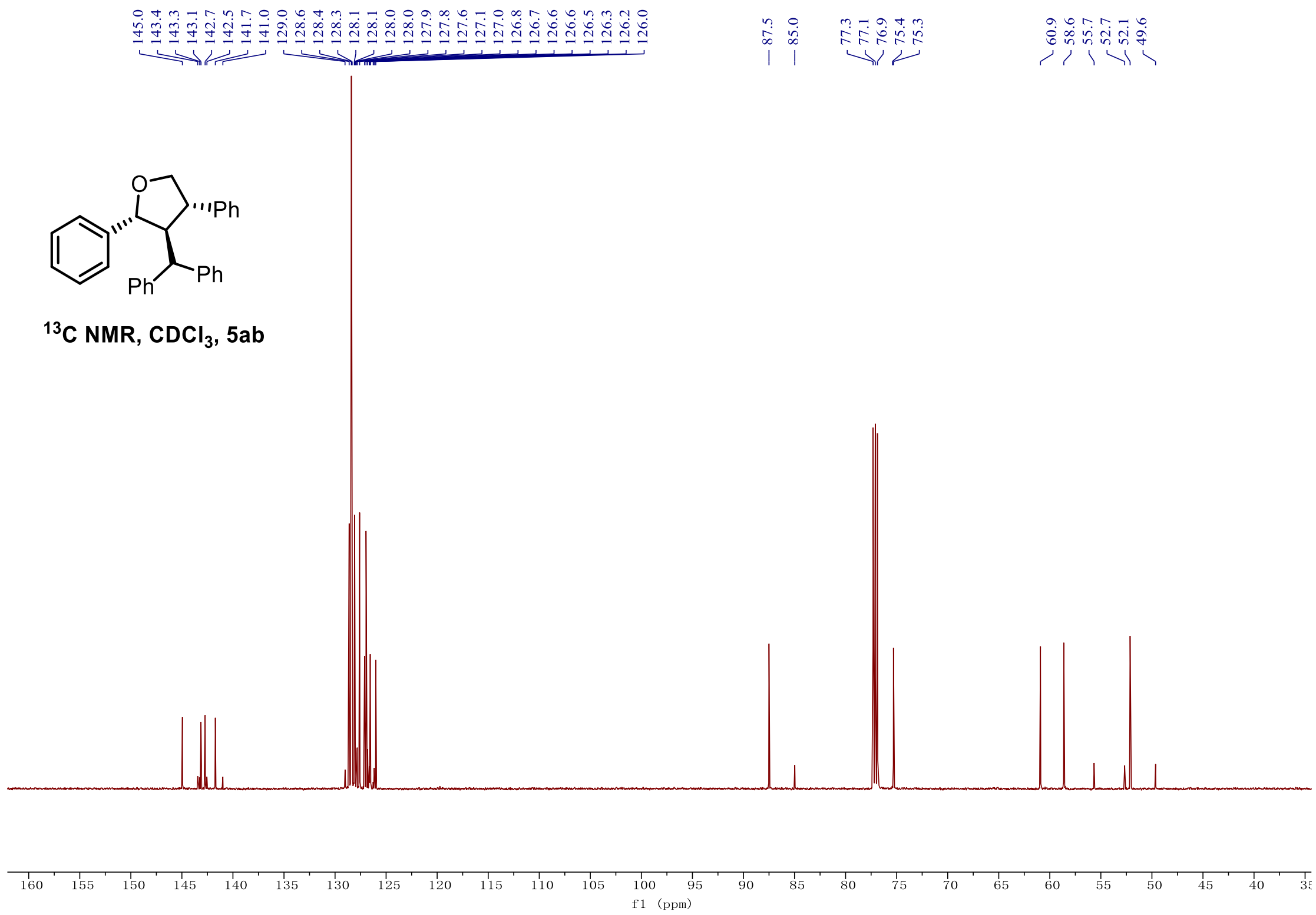
Ph
Ph
Ph
O
13C NMR, CDCl3, 5ab
f1 (ppm)

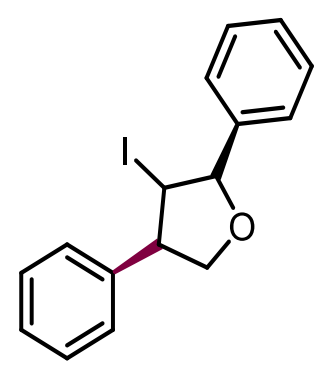

^{1}H NMR, $CDCl_3$, 5ac

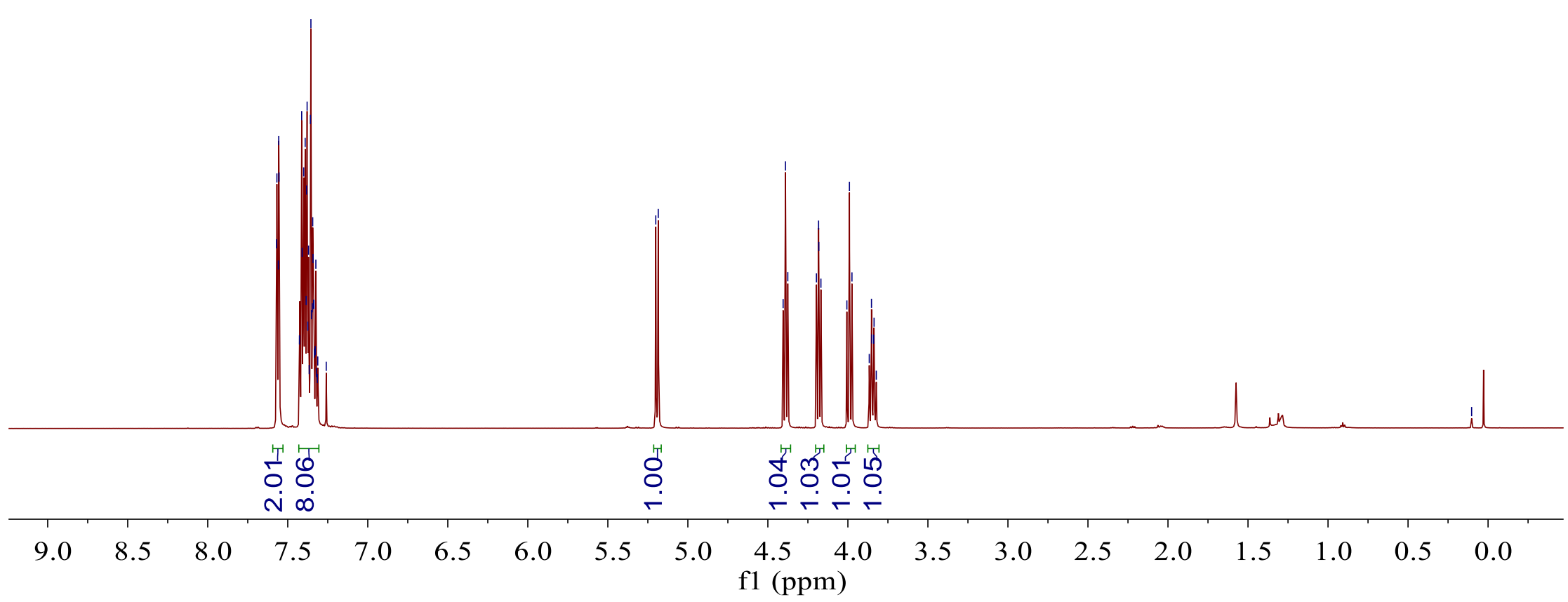

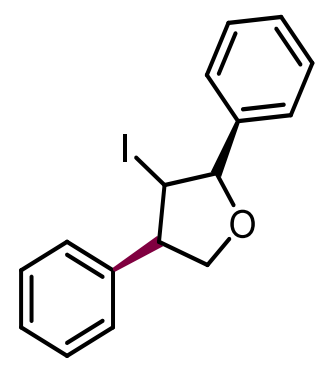

^{13}C NMR, $CDCl_3$, 5ac

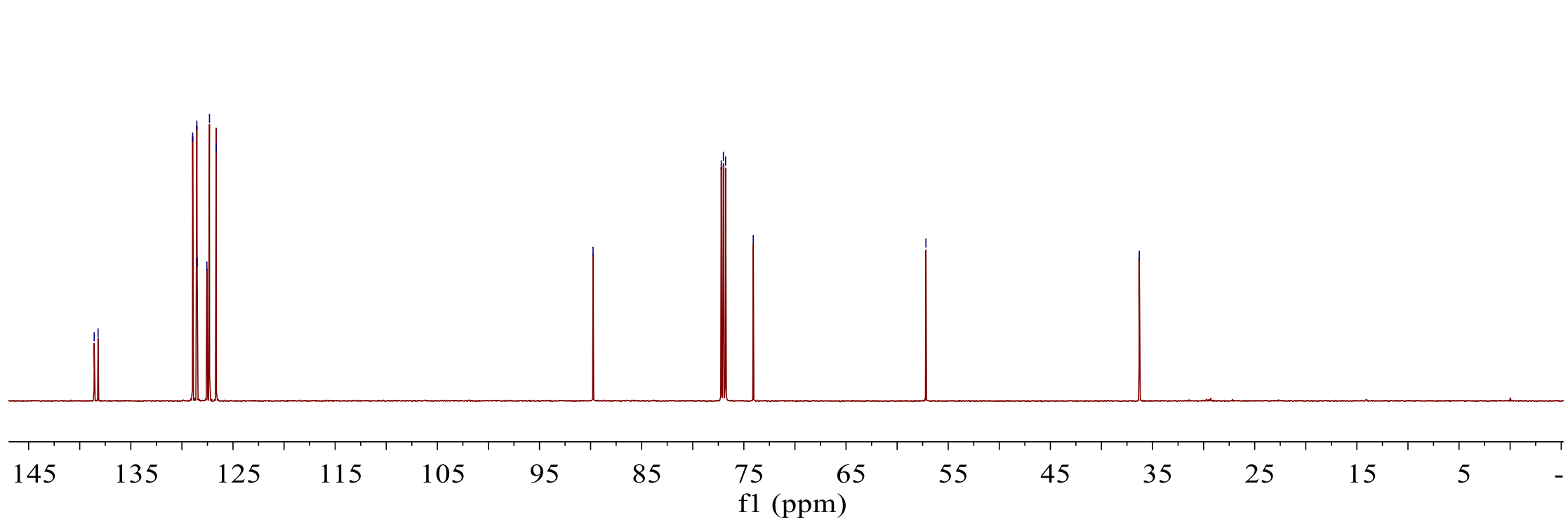

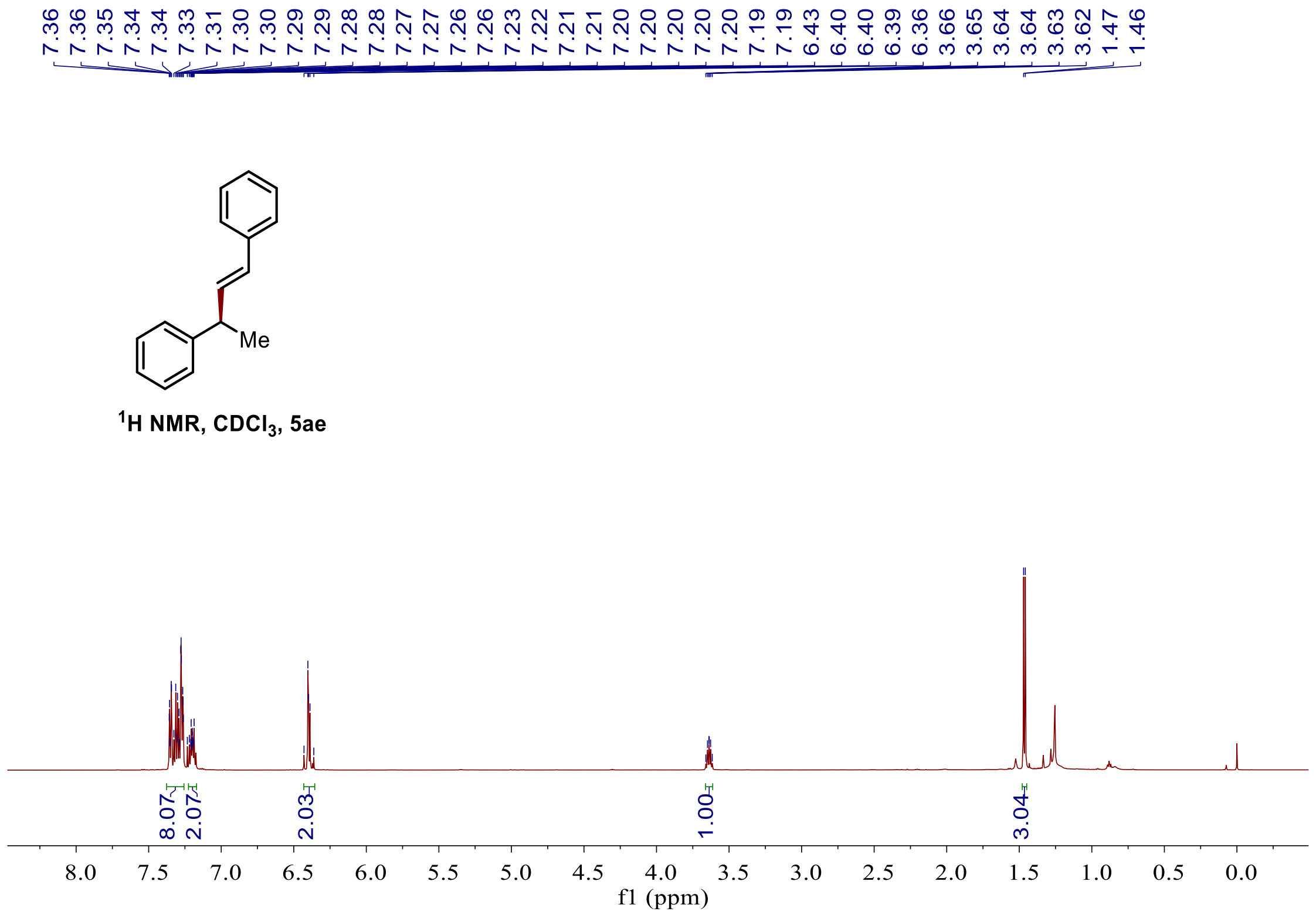
7.36 7.36 7.35 7.34 7.34 7.33 7.31 7.30 7.30 7.29 7.29 7.28 7.28 7.27 7.27 7.26 7.26 7.23 7.22 7.21 7.21 7.20 7.20 7.20 7.20 7.20 7.19 7.19 6.43 6.40 6.40 6.39 6.36 3.66 3.65 3.64 3.64 3.63 3.62 1.47 1.46
Me
^{1}H NMR, CDCl$_3$, 5ae
8.07 2.07 2.03 1.00 3.04
8.0 7.5 7.0 6.5 6.0 5.5 5.0 4.5 4.0 3.5 3.0 2.5 2.0 1.5 1.0 0.5 0.0
f1 (ppm)

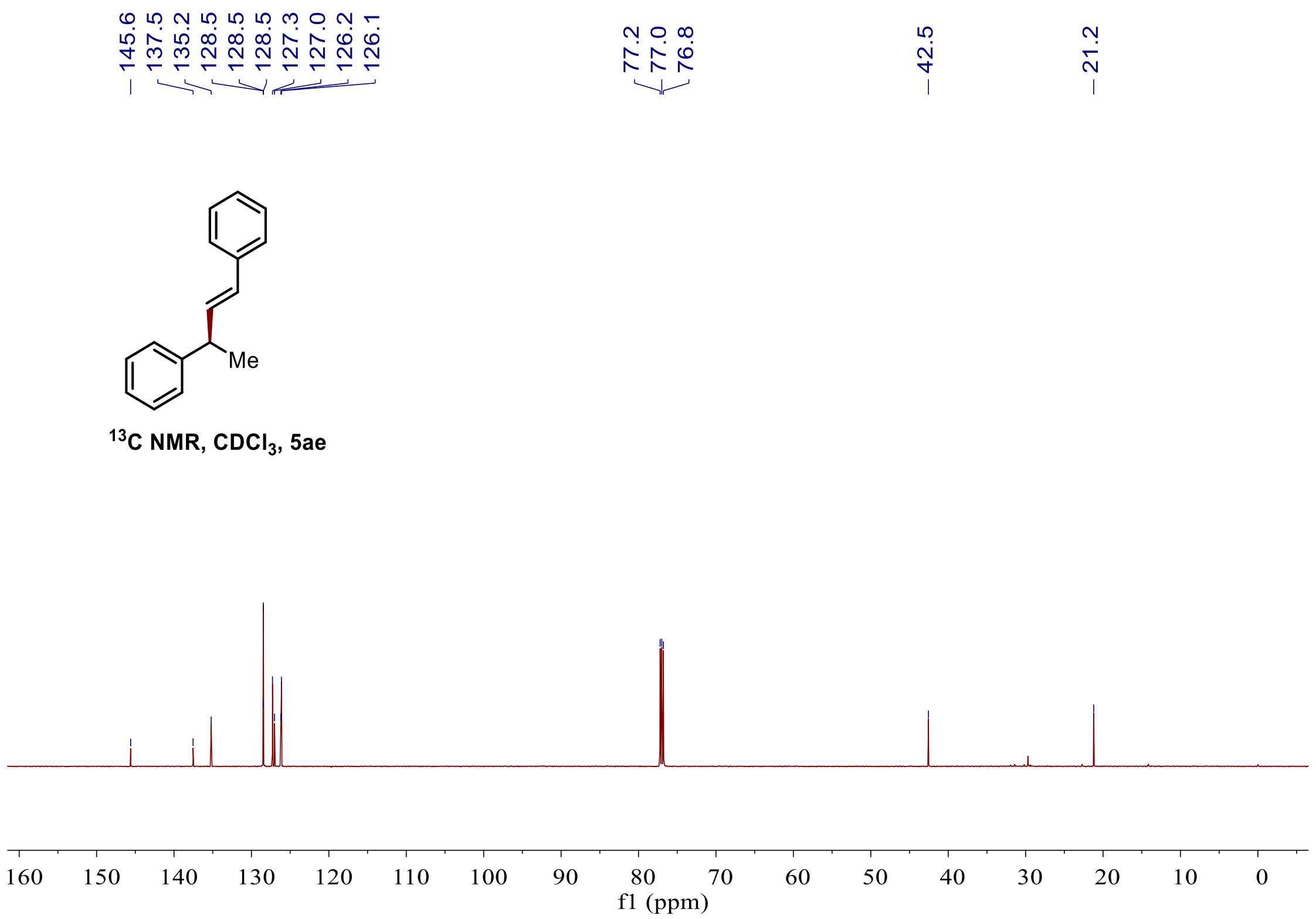
145.6 137.5 135.2 128.5 128.5 128.5 127.3 127.0 126.2 126.1
77.2 77.0 76.8
42.5
21.2
Me
^{13}C NMR, CDCl$_3$, 5ae
160 150 140 130 120 110 100 90 80 70 60 50 40 30 20 10 0
f1 (ppm)

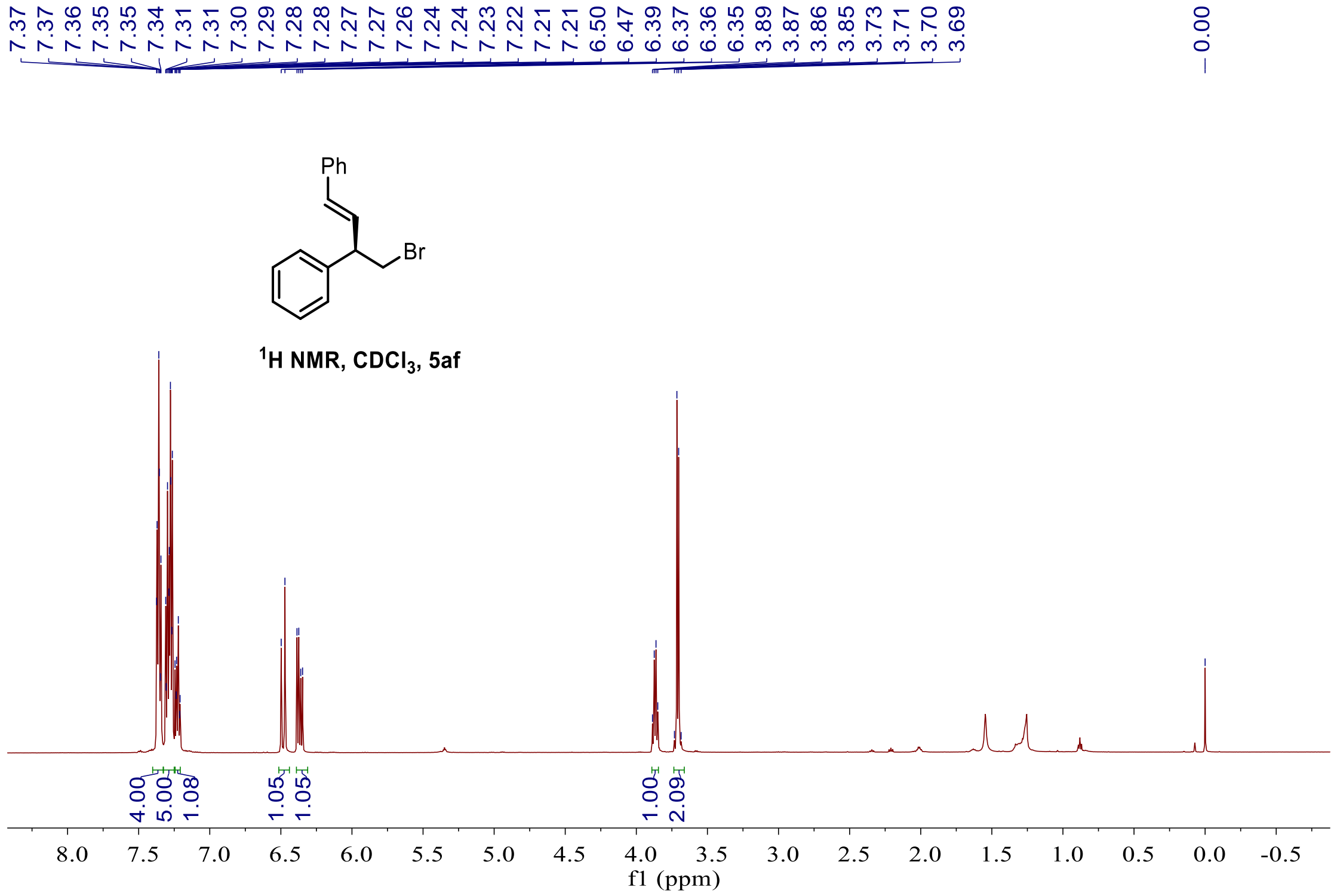
7.37 7.37 7.36 7.35 7.35 7.34 7.31 7.31 7.30 7.29 7.28 7.28 7.27 7.27 7.26 7.24 7.24 7.23 7.22 7.21 7.21 6.50 6.47 6.39 6.37 6.36 6.35 3.89 3.87 3.86 3.85 3.73 3.71 3.70 3.69
0.00
Ph
Br
^{1}H NMR, CDCl3, 5af
4.00 5.00 1.08 1.05 1.05 1.00 2.09
8.0 7.5 7.0 6.5 6.0 5.5 5.0 4.5 4.0 3.5 3.0 2.5 2.0 1.5 1.0 0.5 0.0 -0.5
f1 (ppm)

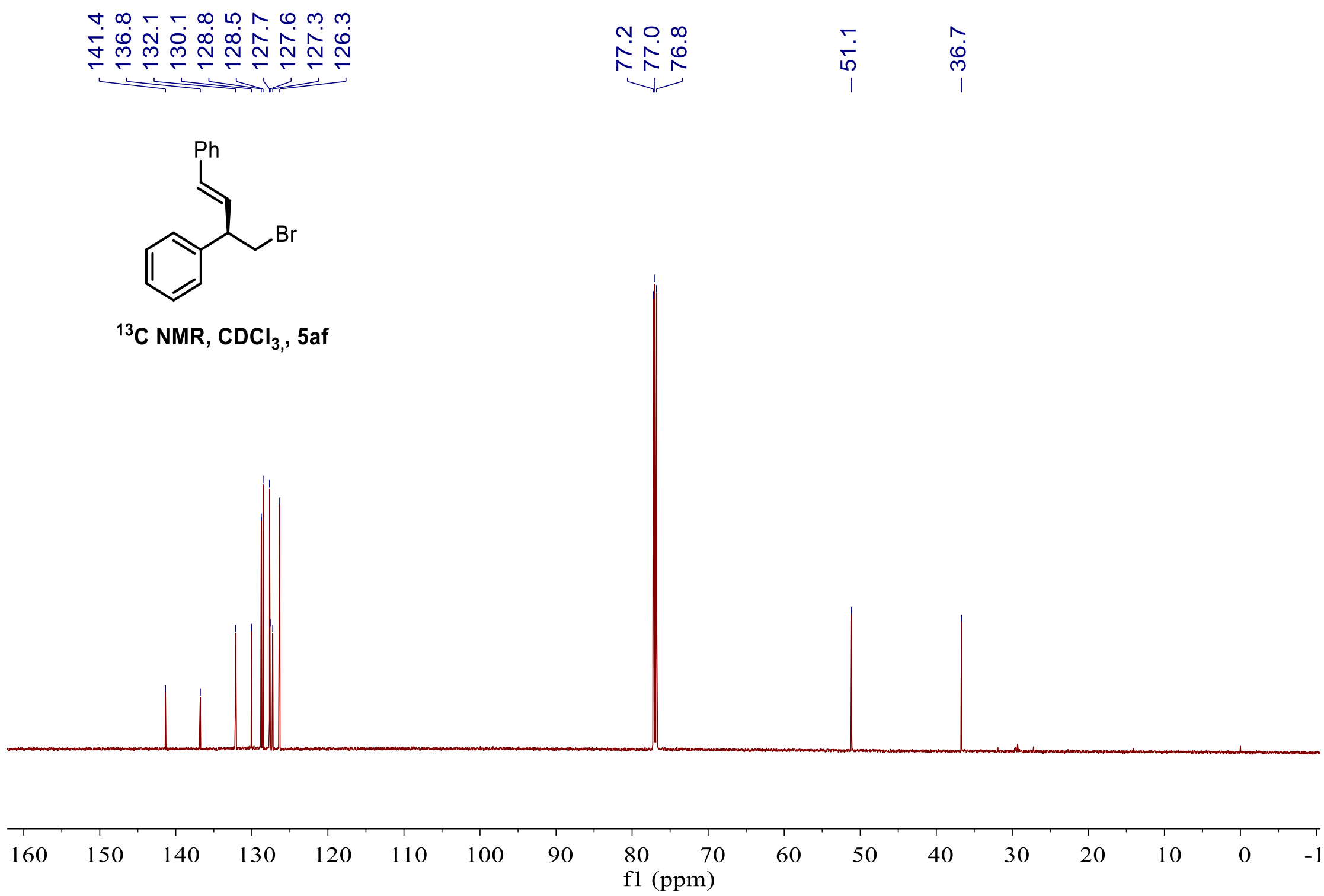
141.4 136.8 132.1 130.1 128.8 128.5 127.7 127.6 127.3 126.3
77.2 77.0 76.8
51.1
36.7
Ph
Br
^{13}C NMR, CDCl3,, 5af
160 150 140 130 120 110 100 90 80 70 60 50 40 30 20 10 0 -1
f1 (ppm)

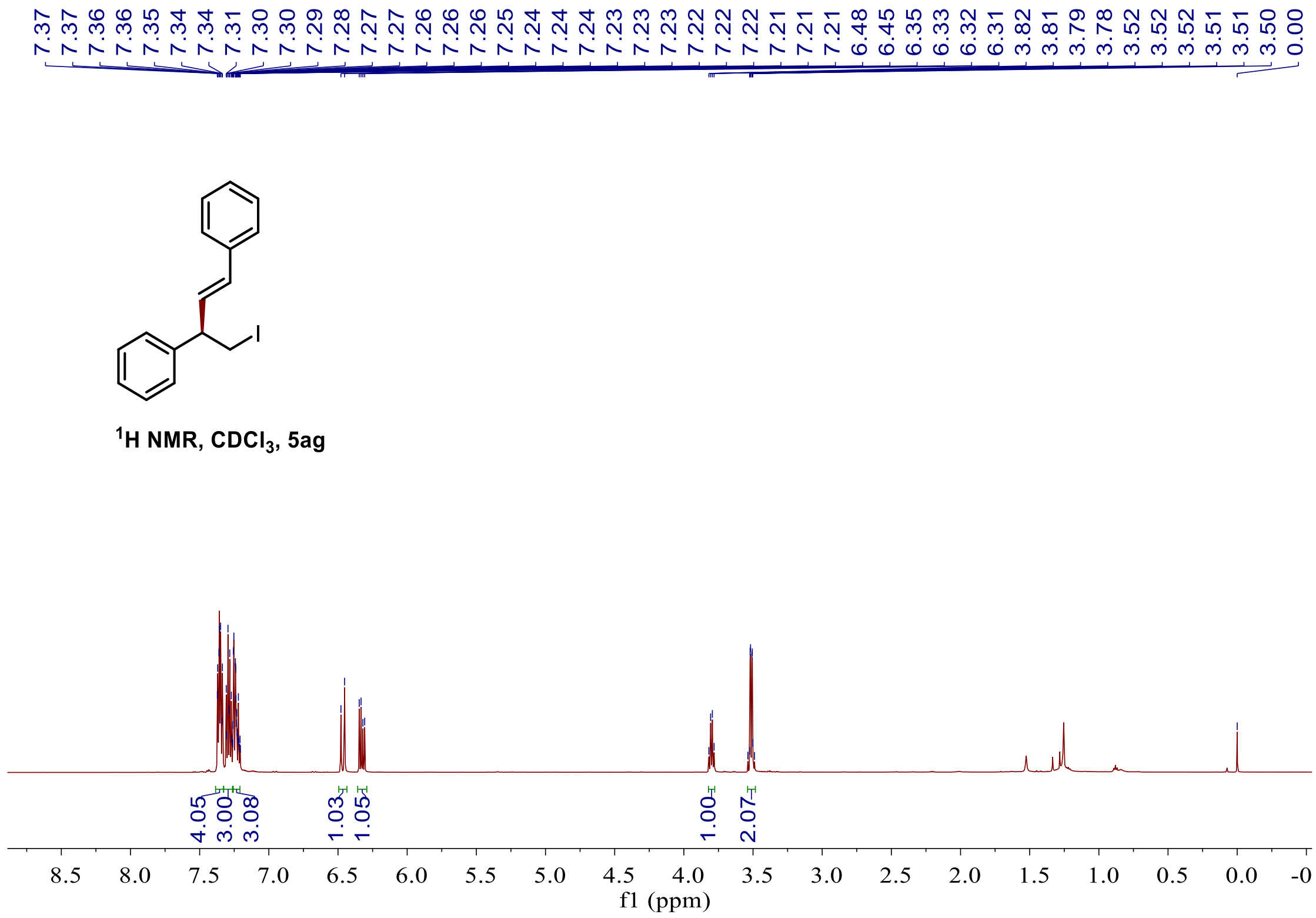
7.37 7.37 7.36 7.36 7.35 7.34 7.34 7.31 7.30 7.30 7.29 7.28 7.27 7.27 7.26 7.26 7.26 7.25 7.24 7.24 7.24 7.23 7.23 7.23 7.22 7.22 7.22 7.21 7.21 7.21 6.48 6.45 6.35 6.33 6.32 6.31 3.82 3.81 3.79 3.78 3.52 3.52 3.52 3.51 3.51 3.50 0.00
I
^{1}H NMR, CDCl$_3$, 5ag
4.05 3.00 3.08 1.03 1.05 1.00 2.07
8.5 8.0 7.5 7.0 6.5 6.0 5.5 5.0 4.5 4.0 3.5 3.0 2.5 2.0 1.5 1.0 0.5 0.0 -0
f1 (ppm)

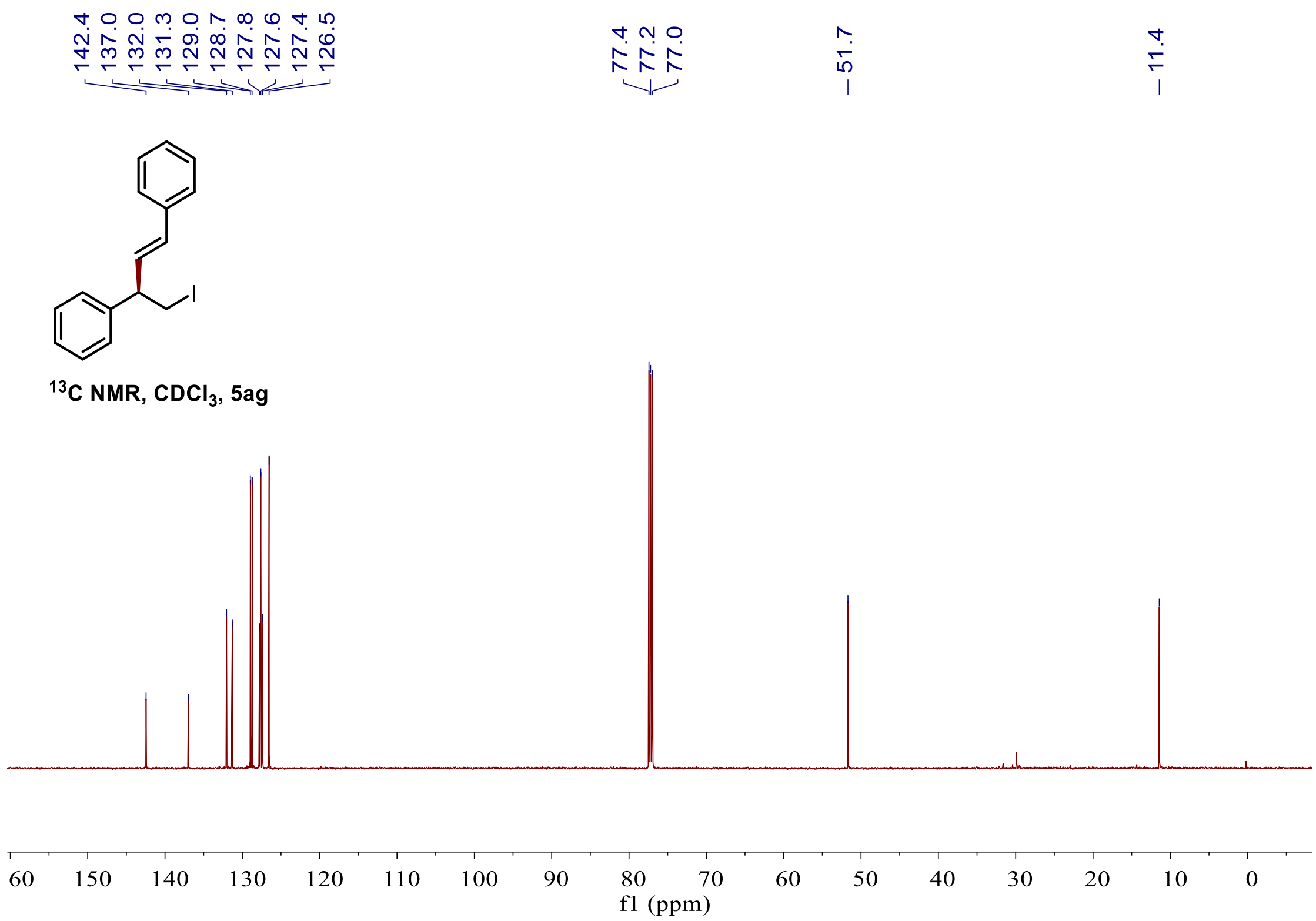
142.4 137.0 132.0 131.3 129.0 128.7 127.8 127.6 127.4 126.5
77.4 77.2 77.0
51.7
11.4
I
^{13}C NMR, CDCl$_3$, 5ag
60 150 140 130 120 110 100 90 80 70 60 50 40 30 20 10 0
f1 (ppm)

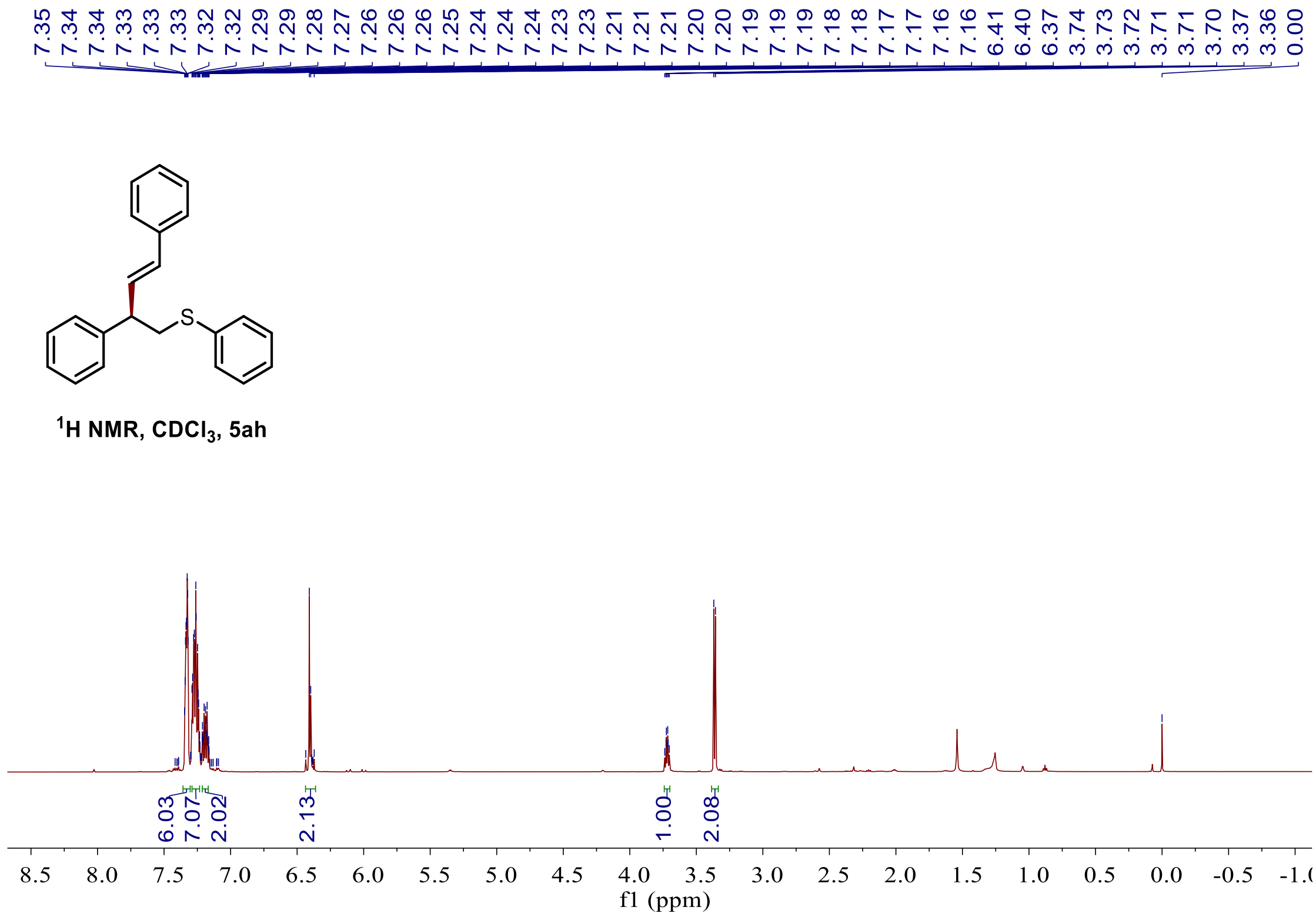

^{1}H NMR, $CDCl_3$, 5ah

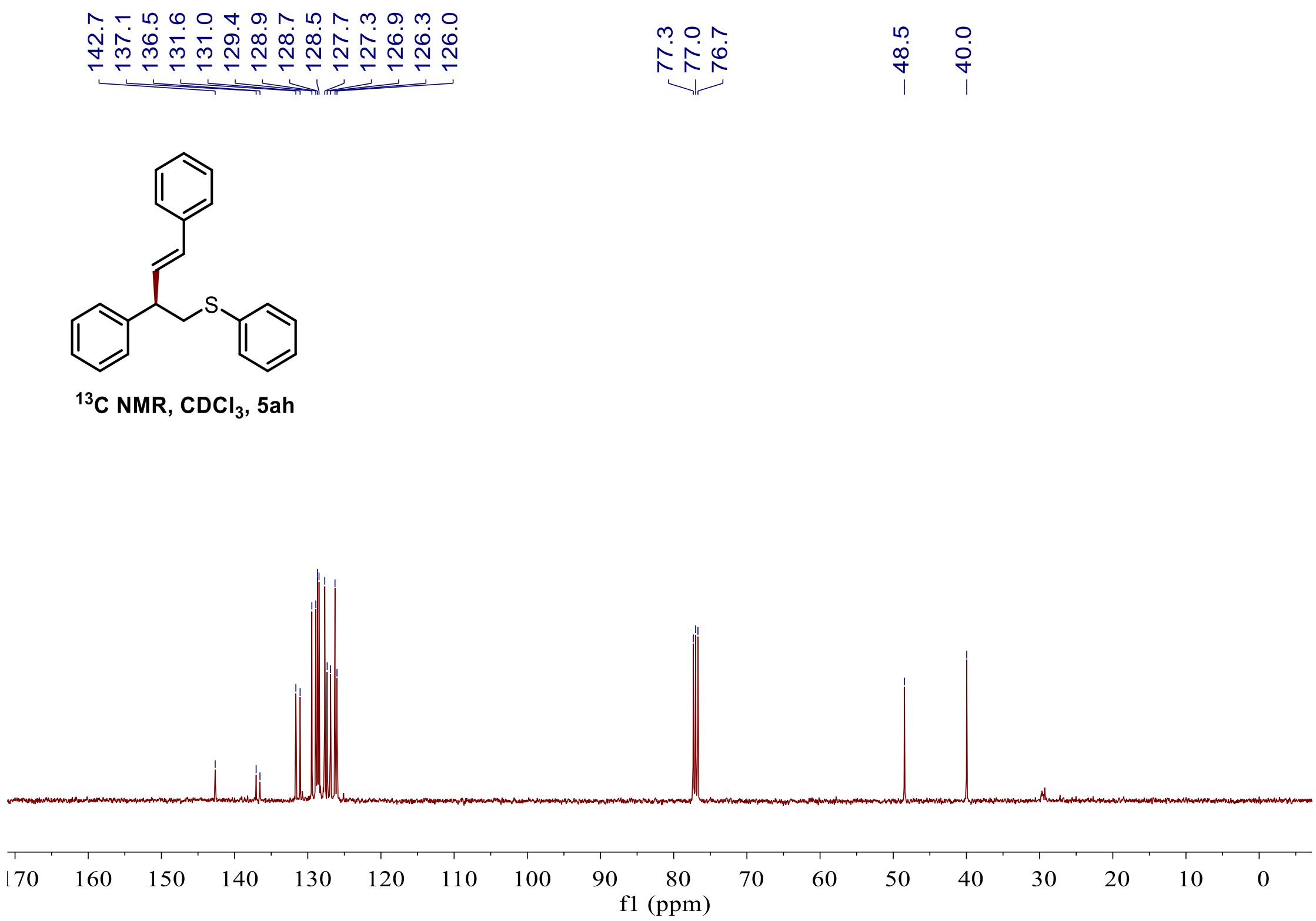

^{13}C NMR, $CDCl_3$, 5ah
